# Analyses of the Sequence and Structural Properties Corresponding to Pentapeptide and Large Palindromes in Proteins

**DOI:** 10.1371/journal.pone.0139568

**Published:** 2015-10-14

**Authors:** Settu Sridhar, Mallapragada Nagamruta, Kunchur Guruprasad

**Affiliations:** Bioinformatics, Centre for Cellular and Molecular Biology (CCMB), Uppal Road, Hyderabad, 500007, India; Russian Academy of Sciences, Institute for Biological Instrumentation, RUSSIAN FEDERATION

## Abstract

The analyses of 3967 representative proteins selected from the Protein Data Bank revealed the presence of 2803 pentapeptide and large palindrome sequences with known secondary structure conformation. These represent 2014 unique palindrome sequences. 60% palindromes are not associated with any regular secondary structure and 28% are in helix conformation, 11% in strand conformation and 1% in the coil conformation. The average solvent accessibility values are in the range between 0–155.28 Å^2^ suggesting that the palindromes in proteins can be either buried, exposed to the solvent or share an intermittent property. The number of residue neighborhood contacts defined by interactions ≤ 3.2 Ǻ is in the range between 0–29 residues. Palindromes of the same length in helix, strand and coil conformation are associated with different amino acid residue preferences at the individual positions. Nearly, 20% palindromes interact with catalytic/active site residues, ligand or metal ions in proteins and may therefore be important for function in the corresponding protein. The average hydrophobicity values for the pentapeptide and large palindromes range between -4.3 to +4.32 and the number of palindromes is almost equally distributed between the negative and positive hydrophobicity values. The palindromes represent 107 different protein families and the hydrolases, transferases, oxidoreductases and lyases contain relatively large number of palindromes.

## Introduction

A palindrome refers to a set of characters in a sequence that reads the same in both directions. Palindromes are present in nucleic acid and protein sequences. Nearly, 30% residues in a protein are members of peptide palindromes, tripeptidic and longer [1]. Palindromes exceeding 10 residues in length are not rare [2]. As the length of the palindrome sequence decreases, more number of palindromes is known to occur in proteins [3]. 26% protein sequences in the SwissProt database comprise at least one palindromic repeat [4]. Palindrome sequences have a high tendency to form α-helices [5]. Generally, the roles of palindrome in protein, is not clear.

In the present study, we have analyzed certain sequence and structural properties associated with palindromes in proteins, such as, probability of amino acid residue occurrence at individual positions in the palindrome sequences of specific length, secondary structure conformation, hydrophobicity, solvent accessibility, residue neighborhood contacts, interaction with catalytic site or active site residues, ligand or metal in proteins and identifying protein families comprising the palindromes. We discuss these features for pentapeptide and large palindromes identified in representative proteins of known three-dimensional structure. Further, we examine for certain illustrative examples, the ‘environment’ of palindromes characterized by the same length, sequence and secondary structure in different proteins.

## Materials and Methods

The representative proteins were selected from the Protein Data Bank (PDB) [6] according to the PDB_SELECT program [7]. They correspond to protein crystal structures determined at ≤ 2.5 Å resolutions and with pairwise sequence identities ≤ 25%. The pentapeptide and large sequences corresponding to the palindromes (or PALINs) were identified using a computer program developed by us for this purpose. We followed a procedure similar to that described in our recent work for the identification of inverted peptides (or INVPEPs) in proteins [8]. Accordingly, in order to identify the PALINs, we consider the first sequence in the dataset and starting with the N-terminal residue, define a ‘probe’ sequence that corresponds to the first five amino acid residues (or pentapeptide). We then generate a ‘target’ sequence by inverting the ‘probe’ sequence. The ‘target’ sequence was then searched starting from the N-terminal residue of the first sequence in the dataset and pentapeptide ‘hits’ that exactly matched the ‘target’ sequence along the protein sequence were identified by sliding one residue at a time until the end of the protein sequence was reached. Later, the ‘target’ sequence was searched for identifying the exact matching ‘hits’ in the next protein sequence and likewise in all the protein sequences in the dataset. Each ‘hit’ corresponds to a PALIN. For each PALIN that was identified in this manner, the PDB code, protein chain, protein name, palindrome sequence, start and end residue positions corresponding to the PALIN sequences in the different proteins were recorded. Then, a new pentapeptide sequence was defined as the ‘probe’ sequence. This new ‘probe’ sequence once again corresponds to the first protein sequence in the analysis dataset but was obtained by sliding along the protein sequence with respect to its previous position by one residue. Accordingly, the corresponding new ‘target’ sequence was generated and with the new ‘target’ sequence, once again PALINs were identified from all proteins in the dataset as described above. This process was repeated until all the pentapeptide ‘probe’ sequences from all the protein sequences in the dataset were examined in order to identify the PALINs. In the same manner, PALINs of varying sequence length, i.e., hexapeptide (6-mer), heptapeptide (7-mer), and so on, up to the length of the largest protein sequence (comprising 1045 amino acid residues) in the dataset were searched. The PALINs identified were sorted according to peptide sequence length. Redundant PALINs that were obtained, i.e., when the ‘target’ was defined as ‘probe’ were excluded from further analyses. The redundant PALINs were identified according to the protein name, PDB code, protein chain, palindrome sequence and location in the protein. Further, PALINs corresponding to the continuous single amino acid repeats (or CARPs) described previously [9] and PALINs corresponding to amino acid residues with missing atom records in the PDB file were excluded from further analysis as their secondary structure information was incomplete. The secondary structure conformations defined according to the hydrogen-bonding patterns as in the DSSP program [10] was retrieved from the PDB website (http://www.rcsb.org). Accordingly, the secondary structure conformations for the individual amino acid residues corresponding to the PALINs were assigned as; H (alpha helix), E (beta-strand), B (beta-bridge), T (turn), G (3/10-helix) and S (bend). The secondary structure conformation for amino acid residue(s) corresponding to a ‘coil’ conformation was assigned ‘C’ and those with missing ATOM records in the PDB was assigned ‘-‘ as described in PSSARD [11, 12]. The solvent accessibility values were derived according to the method described in [13] and obtained using the AREAIMOL program available in the CCP4 software program suite (version 6.4.0) [14]. Accordingly, the solvent accessibility values were calculated for all chains in the protein crystal structure complex by specifying the PDB code corresponding to the protein containing the palindrome sequence as input to the AREAIMOL program. Then, the solvent accessibility values corresponding to the amino acid residues in the palindrome sequence of interest were extracted by writing our own computer programs. The average solvent accessibility values were evaluated by summing up the solvent accessibility values corresponding to the individual amino acid residues and dividing by length of the palindrome peptide sequence. The total number of residue neighborhood contacts for amino acid residues constituting the PALINs was evaluated using the NCONT program available in the CCP4 software suite [14]. Accordingly, the coordinates corresponding to the palindrome sequence in the PDB file was separated as ‘target’ file and the remaining PDB file was considered as ‘source’ file. A distance cut-off value ≤ 3.2 Å was set to evaluate the number of interacting protein atoms between the ‘target’ and ‘source’ files. We developed a computer program in order to evaluate the total number of residue neighborhood interactions made by a palindrome peptide with rest of the protein. The interactions made with a particular amino acid residue in a protein in the specified range were counted only once, irrespective of which amino acid residue(s) in the palindrome peptide made with the protein. Further, all protein chains corresponding to a PDB file were considered for evaluating the average solvent accessibility and the total number of residue neighborhood contacts for a palindrome peptide. The PALINs were analyzed on the basis of sequence length, amino acid sequence, secondary structure, solvent accessibility, residue neighborhood contacts and protein families. The interactions of PALINs with metal, ligand, catalytic site, or a cysteine residue in the sequence involved in a disulphide bridge in the corresponding protein structure were inferred by consulting the PDBsum [15]. The probabilities of amino acid residues observed at individual positions for the PALINs based on sequence length and secondary structure were drawn using WebLogo 3.4 software tool [16] available at http://weblogo.threeplusone.com/create.cgi. PALINs associated with helix, strand or coil conformations were examined on the graphics in order to infer whether they interact when present either in the same protein chain or different chains of the same protein. The PyMol software [17] was used for molecular visualization and for preparing the figures. The average hydrophobicity values were computed based on peptide length for the palindrome sequences according to the hydrophobicity values defined for individual amino acid residues [18].

## Results and Discussion

### Distribution of PALINs in the analysis dataset based on the palindrome sequence length

**Fig 1:**
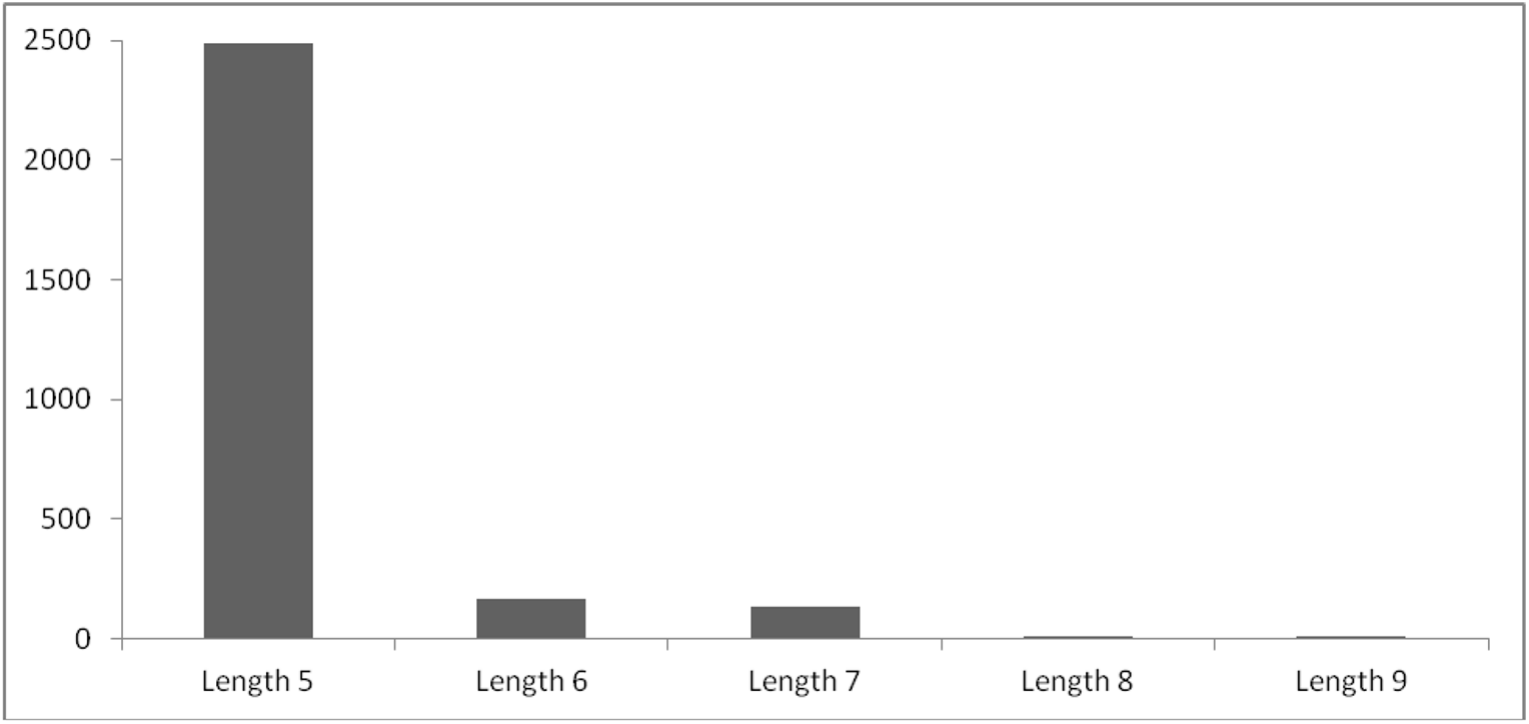
Histogram showing distribution of PALINs (length ≥ 5) in representative proteins of known three-dimensional structure selected from the Protein Data Bank.

The analysis dataset comprised 3967 representative protein chains selected from the Protein Data Bank as described in methods and a list of these proteins is provided in S1 Appendix. We observed 2803 pentapeptide and large PALINs varying in sequence length between five to nine amino acid residues in these proteins. A distribution of the PALINs based on sequence length is shown in Fig 1. Although palindrome sequences greater than nine amino acid residues were observed and have also been previously reported [1, 2], they were not considered for subsequent analyses in the present study as their secondary structure conformation were not available due to the missing ATOM records in the corresponding PDB files. Further, it is possible that the PDB code corresponding to protein containing the palindrome may not be among the representative proteins selected for the analyses. Our results are consistent with previous report that the number of palindromes in proteins increases as length of the palindrome decreases [3]. A list of the 2803 PALIN sequences along with the protein PDB code, protein description, location in protein, palindrome sequence, peptide sequence length, average solvent accessibility, secondary structure conformation, number of residue neighborhood contacts and average hydrophobicity values are provided in S2 Appendix.

### Secondary structure

**Fig 2:**
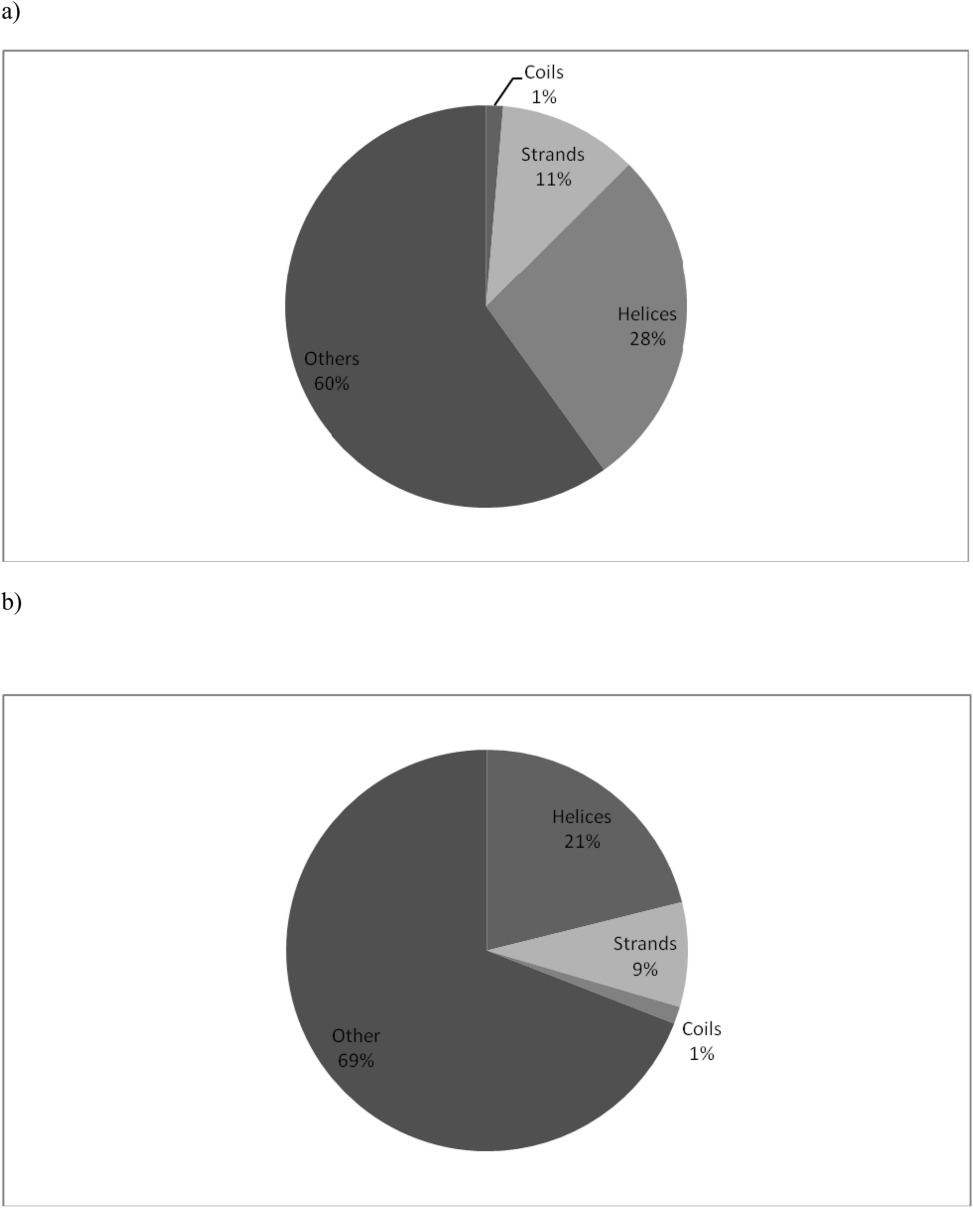
Pie-chart showing the secondary structure composition corresponding to (a) PALINs (length ≥ 5) in representative proteins of known three-dimensional structure and (b) non-PALINs.

Nearly 60% PALINs are neither in a helix or strand conformation demonstrating that a majority of the palindrome sequences in proteins are not associated with a regular secondary structure as shown in Fig 2(A). For example, the palindrome in the blood clotting protein (PDB code:3E1I:A) ‘RALAR’ located between residues 167–171 is in coil conformation. Another palindrome ‘PALGLAP’ in the protein of ‘unknown’ function (PDB code:2FZV:A) mainly comprises the residues in turn conformation. An example of a large palindrome not associated with a regular secondary structure is ‘GDNPRPNDG’ (323–331) in the hydrolase protein (PDB code:3B7E:A). However, among PALINs associated with a regular secondary structure, helix was the most preferred conformation. This observation is in support of a previous report where palindrome sequences were shown to have a relatively high tendency to form α-helices [5]. According to the pie chart shown in Fig 2(A), 28% PALINs are in helix conformation, 11% in strand conformation and 1% entirely in the coil conformation. In order to infer whether there is a significant difference in the secondary structure propensity among palindromes of size N compared with other sequences of size N, we considered another set of equivalent number of non-palindrome sequences (or non-PALINs) of size N in the representative protein dataset. The secondary structure for the non-PALINs was also evaluated as described for the PALINs. A distribution of the secondary structure propensity for non-PALINs is shown in Fig 2(B). The preference for regular secondary structure in PALINs is ~39%, i.e., ~9% higher than compared with non-PALINs, suggesting that there is a greater tendency for the PALIN sequences to adopt a regular secondary structure compared to the non-PALINs. Also, the percentage of helices and strands in PALINs is more than that in the non-PALINs although this preference is more pronounced in the case of helices. The dataset comprises relatively more number of helices (772) compared to the strands (313) and coils (38). The secondary structure conformations for all the palindromes analyzed is shown in S2 Appendix. These data provide information on the sequence-to-structure relatedness for palindromes in proteins that may be useful for prediction and design.

### Sequence conservation

**Fig 3:**
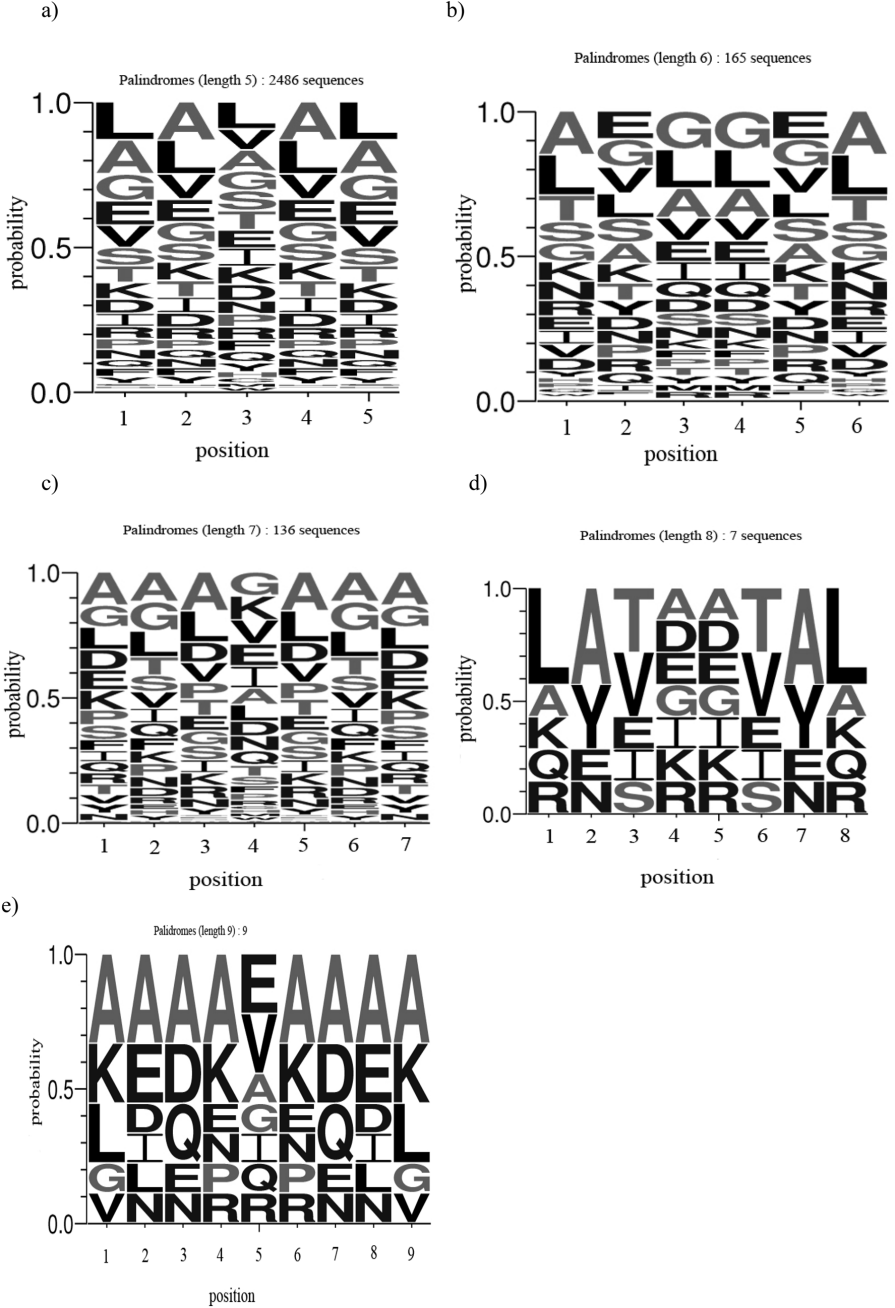
Weblogos for PALINs of different sequence lengths; (a) 5, (b) 6, (c) 7, (d) 8, and (e) 9.

**Fig 4:**
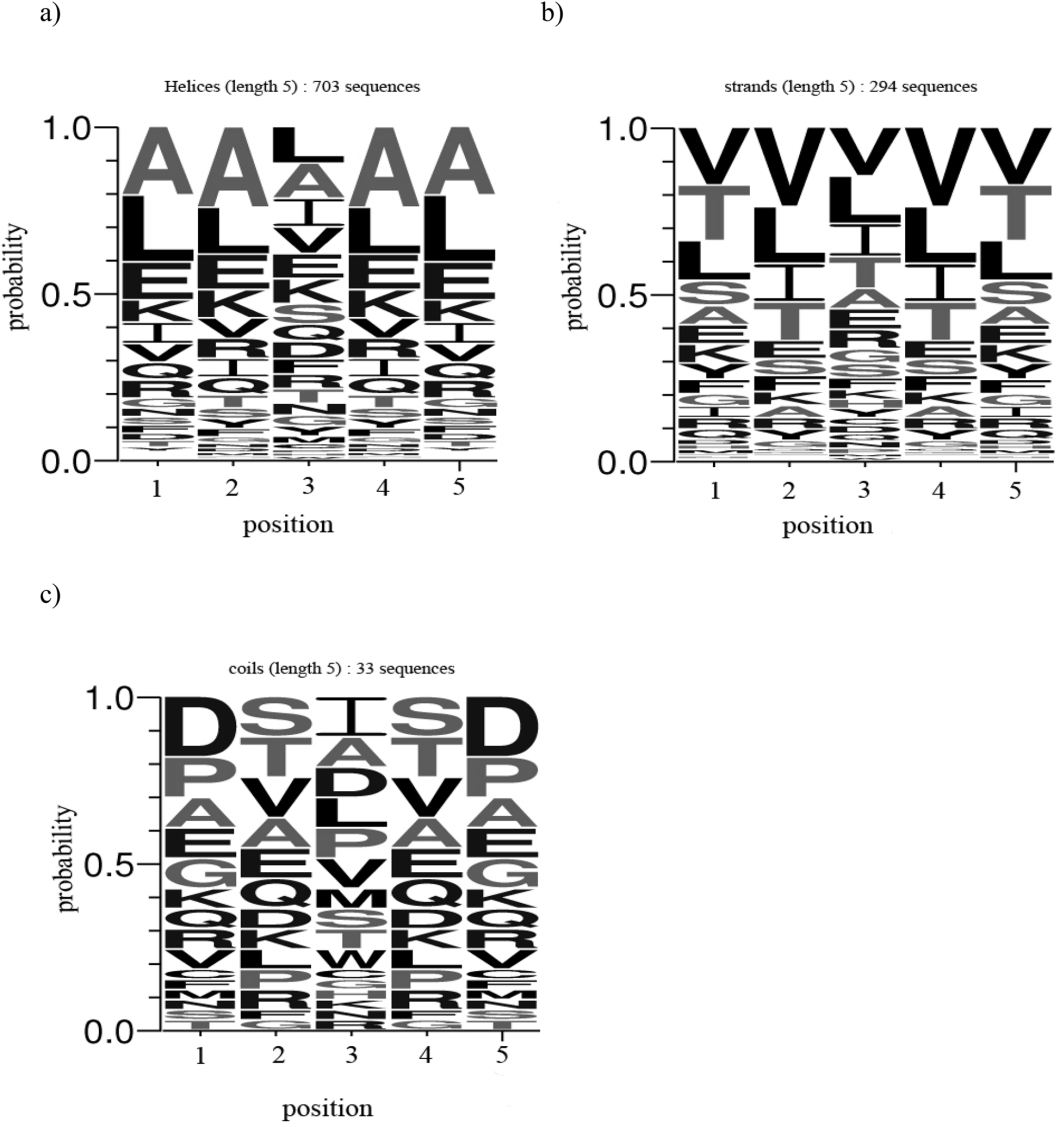
Weblogos for PALINs of length 5 in: (a) helix, (b) strand and (c) coil conformation.

In order to infer the amino acid preferences at individual positions in the PALIN sequences of varying length, weblogos [16] were generated according to the web application available at (http://weblogo.berkeley.edu/logo.cgi). The weblogos were generated by providing the individual PALIN sequences of a particular length in the FASTA format [19]. A logo represents each column of the alignment by a stack of letters, with the height of each letter proportional to the observed frequency of the corresponding amino acid. The weblogos for PALINs of varying sequence lengths are shown in Fig 3A–3E. The consensus sequence can be read from the top of the stack for the palindromes of varying sequence lengths. For instance, the amino acid residue preferences at individual positions for palindrome of length 5 are; ‘L, A, L, A, L’. However, the consensus sequence for palindrome sequence of the same length but different conformations are different as shown in Fig 4A–4C. For instance, the amino acid residue preferences at individual positions for palindrome sequences of length 5 in the helix conformation are; ‘A, A, L, A, A’, and in the strand conformation; ‘V, V, V, V, V’, and in the coil conformation; ‘D, S, I, S, D’.

### Solvent accessibility

**Fig 5:**
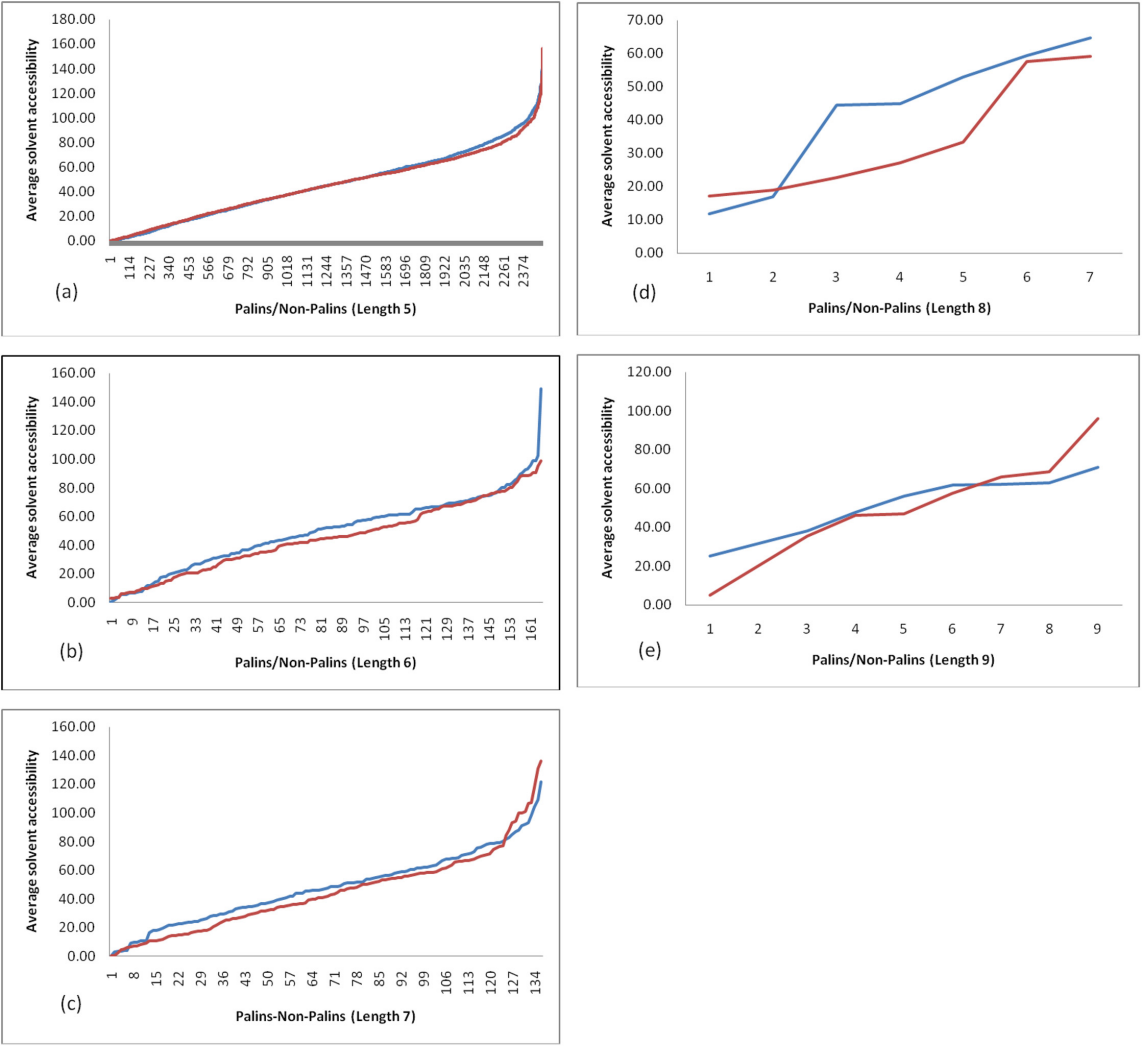
(a-e) Range of average solvent accessibility values corresponding to different sequence lengths for PALINs (blue color) and non-PALINs (red color).

The range of solvent accessibility values for PALINs of different sequence lengths is shown in Fig 5A–5E). This figure is useful to infer the minimum and maximum average solvent accessibility values for the PALINs of different length (shown in blue color). This figure also includes the average solvent accessibility values for equivalent number of non-PALINs of varying sequence length (shown in red color). The average solvent accessibility values for the non-PALINs was evaluated in the same manner as described for the PALINs. There is not much appreciable difference in the extreme values corresponding to the average solvent accessibility observed for PALINs and the non-PALINs of length 5 (Fig 5(A)). This is true also for PALINs of length 6 and 7 (Fig 5(B) and 5(C), respectively), although the average solvent accessibility values for most PALINs in these cases is generally higher than that for the non-PALINs of equivalent length. Our results demonstrate that it is possible to identify non-PALIN peptide sequences with comparable average solvent accessibility values as the PALINs of equivalent length. The PALINs of length 7 have a relatively lower maximum average solvent accessibility value compared to the PALINs of length 5 and 6, suggesting that as the size of the palindrome peptide increases certain larger palindromes tend to be relatively more buried in the protein structure. This observation is more pronounced in the case of certain PALINs of length 8 and 9 (Fig 5(D) and 5(E), respectively). Our analysis on the average solvent accessibility values corresponding to PALINs varying in lengths between 5 to 9 amino acid residues suggests that the PALINs may be either buried, exposed or have an intermittent property in protein three-dimensional structures. In general, as size of the PALIN increases, the maximum average solvent accessibility value decrease from ~155.28 Å^2^ for PALINs of length 5 to ~70.8 Å^2^ for PALINs of length 9, suggesting that the certain large PALINs tend to be relatively buried in protein structure. This is not necessarily true for non-PALINs. Further, the range of solvent accessibilities varies according to the secondary structure conformation. For instance, the solvent accessibility values for PALINs of length 5 in the coil conformation range between 10.06–140.82 Ǻ^2^, for helices between 0–133.06 Ǻ^2^ and for the strands between 0–97.78 Ǻ^2^ demonstrating that certain pentapeptide PALINs in the coil and helix conformations in proteins are relatively more exposed to the solvent compared to the pentapeptide PALINs in the strand conformation which has maximum solvent accessibility value ~98 Ǻ^2^. Likewise, certain pentapeptide PALINs in the strand and helix conformation are relatively more buried in protein three-dimensional structure compared to the pentapeptide PALINs in the coil conformation; the minimum solvent accessibility values for PALINs in the coil conformation is ~10 Ǻ^2^. The solvent accessibility values provided in S2 Appendix are useful to associate with sequence, length and secondary structure corresponding to the palindromes. The same palindrome sequence may be associated with different solvent accessibility values in different proteins and many such instances were observed. For example, the palindrome sequence ‘AAIAA’ in the transferase protein (PDB code:1EKQ_A) located between positions 217–221, has solvent accessibility value 0.3 Å^2^, whereas, in another protein of unknown function (PDB code:2RFR_A) the solvent accessibility is 37.88 Å^2^. This observation is true for the palindrome sequences associated with helix, strand or the coil conformation. While amino acid residues, such as, Glycine and Proline are usually known to reside in coils, they may also be associated with a helix conformation as in the palindrome ‘PGYGP’ (PDB code:2J8Q_B, residues 206–210) or the strand conformation as in the palindrome ‘RGPGR’ (PDB code:3OJN_D, residues 243–247). The supplementary data shown in S2 Appendix is useful to infer structural properties of several palindrome sequences, i.e., whether a given palindrome sequence is buried or exposed, and what is its associated secondary structure conformation as observed among proteins of known three-dimensional structure.

### Neighborhood interactions in proteins

The number of residue neighborhood interactions (defined by ≤ 3.2 Å cut-off value) for all the 2803 PALINs is in the range between 0 to 29 residues. PALINs with no residue neighborhood contacts are relatively exposed. For instance, the PALIN sequence ‘DNYND’ in the virus protein (PDB code:1AYM:1) located between residues 86–90 does not make any residue neighborhood contacts in the specified range and is also relatively exposed to the solvent with solvent accessibility value 79.16 Ǻ^2^. On the other hand, the PALIN sequence ‘VVLVV’ in the hydrolase protein (PDB code:1MJ5:A) located between residues 102–106 in strand conformation has 29 neighborhood residue contacts and therefore buried with the solvent accessibility value 0.34 Ǻ^2^. The PALIN sequence ‘KLTLK’ located between residues 41–45 in the fluorescent protein (PDB code:2WUR:A) that is in a strand conformation has an intermittent value of 15 residue neighborhood contacts. The residue neighborhood contact values for the palindromes provided in S2 Appendix are again useful to relate with length, sequence, secondary structure and the solvent accessibility.

### PALIN interactions in proteins

In order to infer whether the PALIN sequences may be involved in protein function, we consulted the PDBsum and examined whether the amino acid residues in the pentapeptide and large PALINs interact with ligand or the catalytic sites in proteins. The PDBsum has many useful features on protein annotation among which it has two large and diverse benchmark sets for predicting functional residues, based on the Catalytic Site Atlas by Thornton and coworkers [20] and the PDB SITE records, [CSA and SITE]. The PDB SITE entries contain records that indicate important sites such as the ligand binding sites. Site records are generated by the entry author and/or by software based on a distance cutoff from a ligand. The Catalytic Site Atlas (CSA) database documents enzyme active sites and catalytic residues in enzymes of 3D structure (ref: http://www.ebi.ac.uk/thornton-srv/databases/CSA/). Accordingly, we evaluated whether the PALINs are associated with any of these features i.e., does the palindrome sequence interact with residues in the catalytic site, ligand or metal, or does the palindrome sequence contain a cystine residue involved in a disulphide bridge in the protein. Out of 2803 palindrome sequences that were analyzed, ~20% were associated with one or more of the interaction types described above, suggesting that certain PALINs in proteins may be involved in the protein function. The interactions of PALINs with ligand and catalytic site are relatively more common. Few PALINs are characterized by more interactions, for instance, the PALIN sequence ‘SHSHS’ in the transferase protein (PDB code:2Q4H_B-chain) has four of the five interactions described.

### PALINs—unique sequences, commonly observed sequences and ‘chameleons’

There are 2014 unique PALIN sequences that correspond to the 2803 PALINs observed in our analyses. These are shown in S3 Appendix. They represent 1705 (length 5), 158 (length 6), 135 (length 7), 7 (length 8), and 9 (length 9) unique palindrome sequences. Some commonly observed PALIN sequences are: ‘AAEAA’, ‘AAIAA’, ‘AALAA’, ‘AAVAA’, ‘EKLKE’, ‘GGSGG’, ‘KEAEK’, ‘LAAAL’, ‘LASAL’, ‘LEKEL’, ‘LKEKL’. Identical palindrome sequences can adopt different conformations representing ‘chameleon’ PALINs in proteins. For instance, ‘GKVKG’ in the basement membrane protein (PDB code: 1GL4:A) located between 406–410 is mainly in strand conformation, whereas, in the oxidoreductase protein (PDB code:2JAE:A) located between 7–11, it is mainly in the coil conformation. A number of such ‘chameleon’ PALINs in proteins were observed in our analysis.

### PALIN hydrophobicity

**Fig 6:**
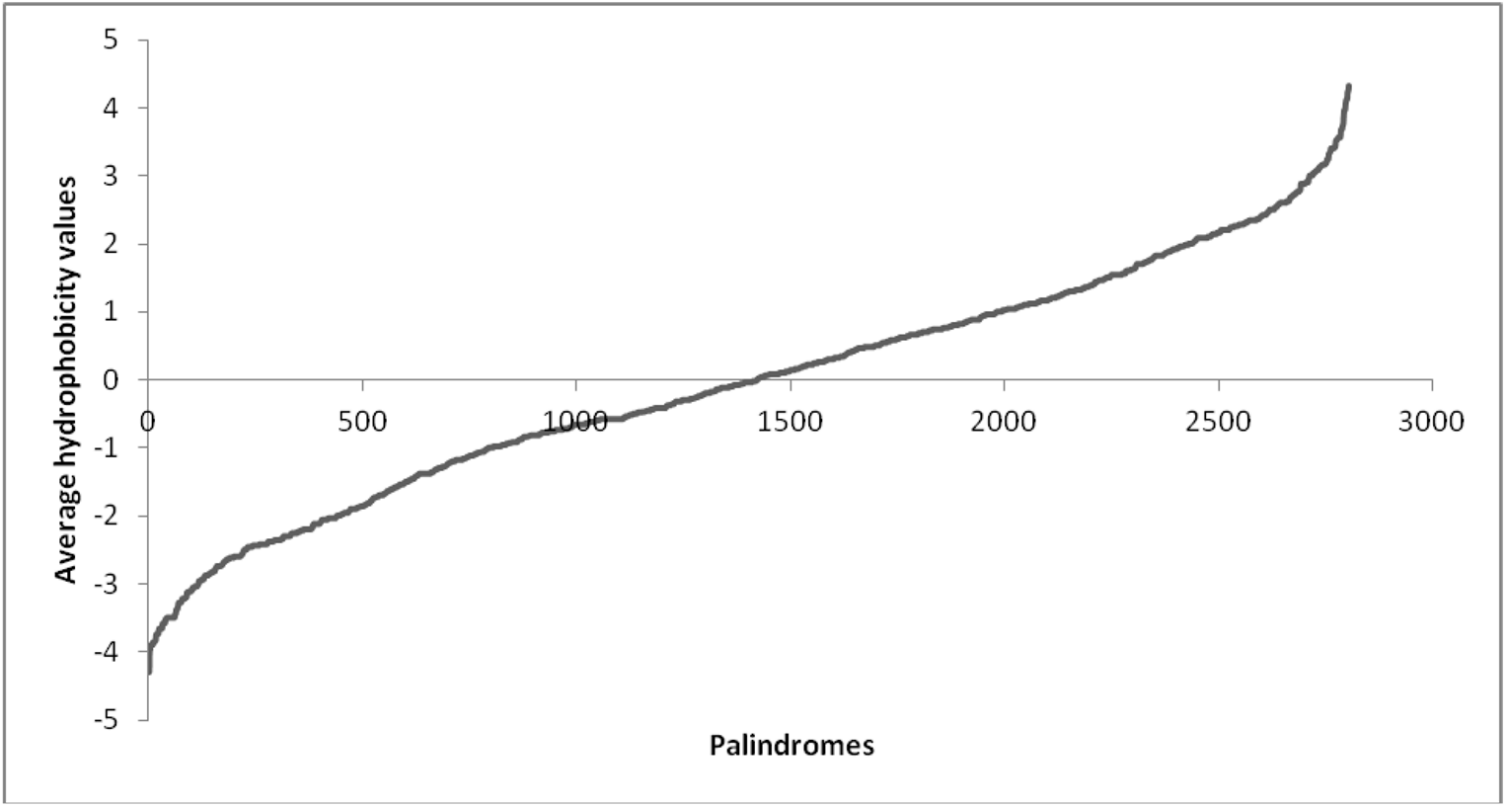
Average hydrophobicity values corresponding to the palindromes.

The average hydrophobicity values computed for all the PALINs in the present study range between -4.3 to +4.32 as shown in Fig 6. There is an almost equal distribution of the number of PALINs in proteins associated with negative and positive hydrophobicity values. The hydrophobicity values for the individual PALINs are shown in S2 Appendix. The palindrome sequence ‘RRNRR’ in the viral protein (PDB code:3LPH_D) is among the palindromes with the least hydrophobicity value -4.3. The palindrome sequence ‘VIVIV’ in the electron transport protein (PDB code:3WU2) is at the other extreme of hydrophobicity value 4.3. The palindrome sequence ‘KAVAK’ in the isomerase protein (PDB code:3A9S_C) has an intermittent average hydrophobicity value 0.

### PALINs with identical sequence and secondary structure conformation in different proteins

**Fig 7:**
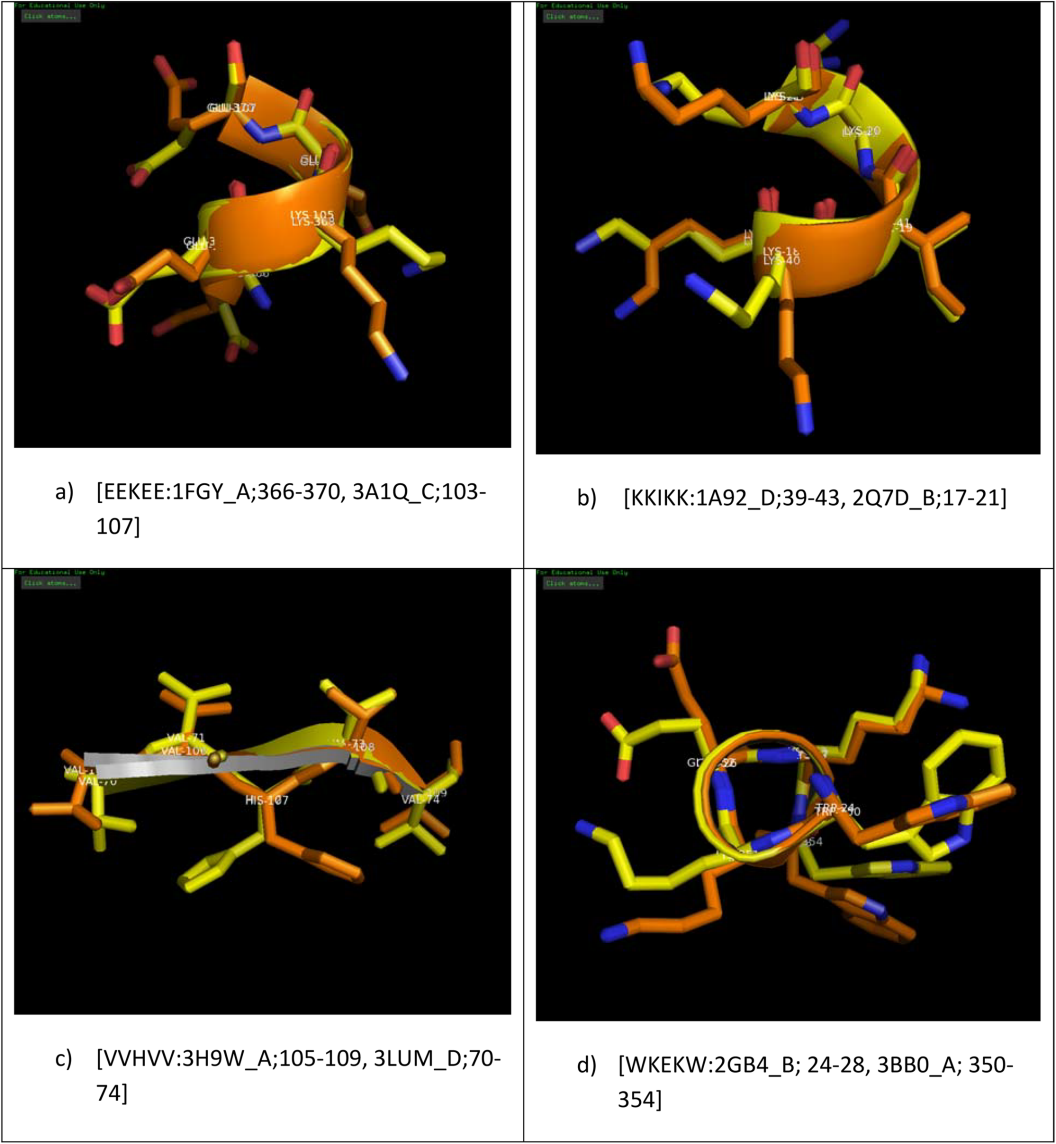
(a-d) Illustrative examples showing the structural overlay corresponding to the palindromes of same length, sequence and secondary structure in different proteins (brown and yellow colors). The palindrome sequence and location in the corresponding PDB code are indicated in each panel. The side-chain orientations for at least one or more equivalent residues is different in each case suggesting that the ‘environment’ representing the complementary charge distribution and shape of the proteins characterized by these palindromes need not be the same.

Palindrome sequences in proteins may comprise negatively charged residues, positively charged residues, hydrophobic residues or a combination of these types of residues, but the pattern of amino acid residue distribution remains the same on either side of the centre or central residue within a palindrome sequence. We wanted to examine, whether this property of a palindrome sequence is also reflected in different proteins comprising them, i.e., do different proteins containing palindromes of same length, sequence and secondary structure have the same ‘environment’ surrounding the palindrome. In other words, is there a complement-arty of charge and shape that is conserved in different protein three-dimensional structures in order to be able to accommodate the same palindrome peptide? To address the above, we examined four illustrative examples corresponding to palindrome peptides of same length, sequence and secondary structure in different proteins. The Cartesian co-ordinates corresponding to the palindrome sequences in the proteins were extracted from their corresponding PDB files and a structural superposition was performed using the PyMol software. Our analysis showed that although the main-chain conformation characterizing the secondary structure remained the same, the side-chain conformations at equivalent positions in the palindromes were different as shown in Fig 7A–7D). This was true of palindromes in the helix conformation (Fig 7(A), 7(B) and 7(D)) as well as for palindromes in the strand conformation (Fig 7(C)), suggesting that the ‘environment’ surrounding the palindrome in the corresponding protein need not be the same. This feature is also reflected in the significantly different average solvent accessibility values for the same palindrome sequence in different proteins (refer S2 Appendix).

### Distribution of PALINs in protein families and their mode of occurrence

The 2803 PALINs represent 107 different proteins identified according to the classification records available for the protein entries in the PDB (http://www.rcbs.org). A list of the number of PALINs and their associated protein families is provided in S4 Appendix. A relatively large number of PALINs were observed in the hydrolases (526), transferases (330), oxidoreductases (292) and lyases (145). A single protein chain may comprise several palindromes. For instance, the electron transport protein (PDB code:2J8C:L) has as many as nine pentapeptide and large palindromes; ‘VGFFGV’ (31–36), ‘FAFAF’ (119–123), ‘AFAFA’ (120–124), ‘GYTYG’ (161–165), ‘ALALA’ (184–188), ‘LALAL’ (185–189), ‘EKGKE’ (201–205), ‘ITGTI’ (250–254) and ‘WWQWW’ (262–266). Situations of palindrome within a palindrome were observed as in the oxidoreductase protein (PDB code:1N62:E); with ‘QKAKQ’ (463–467) in ‘AEQKAKQEA’ (461–469) that is associated with a helix conformation. Overlapping palindromes were observed, for example, in the allergen protein (PDB code:1L3P:A) ‘AATAA’ (168–172) and ‘ATAAATA’ (169–175) mainly associated with the helix conformation. Consecutive palindrome sequences were observed in the signaling protein (PDB code:1T0H:A) ‘REAER’ (42–46) and ‘QAQAQ’ (47–51) that are mainly associated with a helix conformation in the protein. In another example, observed in the apoptosis protein (PDB code: 2O71:A), the palindrome sequence ‘NHPHN’ (151–155) is in coil/turn conformation and ‘VQSQV’ (156–160) is in a helix conformation in the protein. The cell adhesion protein (PDB code:3M7P:A) contains the same palindrome sequence ‘GNSNG’ at two locations; (353–357) and (414–417).

## Conclusions

In this study, we have identified 2803 pentapeptide and large palindrome sequences with known secondary structure from 3967 representative proteins selected from the Protein Data Bank. These palindrome sequences comprise between 5 to 9 amino acid residues with the penta-peptides constituting ~89%. Among the palindromes analyzed, nearly, 71% represent unique palindrome sequences. A majority of the palindromes were not associated with any regular secondary structure, however, the helix conformation was more common among the regular secondary structures, although ~11% palindromes were also observed in strand conformation and ~1% in coil conformation. The consensus amino acid residue preferences for palindromes of the same length but different secondary structure were different. Palindromes can be either buried, exposed or have an intermittent property. The larger palindromes comprising 8 or 9 amino acid residues tend to be relatively more buried compared to the shorter palindromes comprising 5 to 7 amino acid residues in protein structures. Nearly 20% palindromes interact with ligand, metal ion or active site/catalytic site residues in proteins and therefore may be associated with a functional role. Palindromes with negative and positive average hydrophobicity values were observed to be almost equally distributed in proteins. Palindromes of the same length, sequence and secondary structure, need not necessarily share the same ‘structurally conserved environment’ in different proteins, as the side-chain conformations accompanying the equivalent amino acid residues in the palindromes could be different. Certain other aspects of palindromes in proteins related to; a single protein chain comprising a number of palindromes, same palindrome sequence repeated more than once along the protein chain, occurrence of palindromes within palindromes, overlapping palindromes, consecutive palindromes, or ‘chameleon’ palindromes that represent identical sequence with different conformation.

## Supporting Information

S1 Appendix List of Protein Data Bank (PDB) codes corresponding to representative proteins used in the analysis of palindrome peptides. (DOC)

S2 Appendix Palindromes in representative proteins selected from the Protein Data Bank and their associated structural properties. (DOC)

S3 Appendix List of 2014 unique palindrome sequences (orange) in representative proteins of known three-dimensional structure. (DOC)

S4 Appendix Distribution of the number of palindrome peptides in representative proteins. (DOC)
