# Supplementary material for: Analyses of the Sequence and Structural Properties Corresponding to Pentapeptide and Large Palindromes in Proteins
Source: PLoS One. 2015 Oct 14;10(10):e0139568. doi: 10.1371/journal.pone.0139568 (PMC4605511; doi:10.1371/journal.pone.0139568)
Supplement: S1 Appendix — (DOC) [file pone.0139568.s001.doc]

| **S1 Appendix. List of Protein Data Bank (PDB) codes corresponding to representative protein chains used in the analysis of palindrome peptides.** | | | |
| --- | --- | --- | --- |
| S.No. | PDB code | No. of residues | Resolution  (in Å units) |
|  |
| 1 | 1A17A | 159 | 2.45 |
| 2 | 1A1IA | 85 | 1.6 |
| 3 | 1A1XA | 106 | 2 |
| 4 | 1A2PC | 108 | 1.5 |
| 5 | 1A2XB | 31 | 2.3 |
| 6 | 1A34A | 147 | 1.81 |
| 7 | 1A78B | 134 | 2 |
| 8 | 1A79C | 171 | 2.28 |
| 9 | 1A7WA | 68 | 1.55 |
| 10 | 1A8VB | 116 | 2 |
| 11 | 1A92D | 49 | 1.8 |
| 12 | 1A99D | 341 | 2.2 |
| 13 | 1ABAA | 87 | 1.45 |
| 14 | 1AGQC | 91 | 1.9 |
| 15 | 1AH7A | 245 | 1.5 |
| 16 | 1ALUA | 157 | 1.9 |
| 17 | 1AOHA | 143 | 1.7 |
| 18 | 1APYA | 161 | 2 |
| 19 | 1AQ0B | 306 | 2 |
| 20 | 1AQZA | 142 | 1.7 |
| 21 | 1ARBA | 263 | 1.2 |
| 22 | 1AX8A | 130 | 2.4 |
| 23 | 1AXDB | 209 | 2.5 |
| 24 | 1AY7B | 89 | 1.7 |
| 25 | 1AYM1 | 285 | 2.15 |
| 26 | 1AYM3 | 238 | 2.15 |
| 27 | 1AYM4 | 29 | 2.15 |
| 28 | 1B0BA | 142 | 1.43 |
| 29 | 1B0NA | 103 | 1.9 |
| 30 | 1B0NB | 31 | 1.9 |
| 31 | 1B0UA | 258 | 1.5 |
| 32 | 1B1UA | 117 | 2.2 |
| 33 | 1B33O | 67 | 2.3 |
| 34 | 1B34A | 80 | 2.5 |
| 35 | 1B34B | 74 | 2.5 |
| 36 | 1B3AA | 67 | 1.6 |
| 37 | 1B5EA | 241 | 1.6 |
| 38 | 1B66B | 138 | 1.9 |
| 39 | 1B8OA | 280 | 1.5 |
| 40 | 1B93C | 144 | 1.9 |
| 41 | 1BB1C | 34 | 1.8 |
| 42 | 1BB9A | 83 | 2.2 |
| 43 | 1BC8C | 93 | 1.93 |
| 44 | 1BD3B | 224 | 1.93 |
| 45 | 1BDOA | 80 | 1.8 |
| 46 | 1BECA | 238 | 1.7 |
| 47 | 1BG8B | 76 | 2.2 |
| 48 | 1BGCA | 158 | 1.7 |
| 49 | 1BGFA | 124 | 1.45 |
| 50 | 1BHPA | 45 | 1.7 |
| 51 | 1BI5A | 389 | 1.56 |
| 52 | 1BJAA | 95 | 2.19 |
| 53 | 1BKPB | 278 | 1.7 |
| 54 | 1BKRA | 108 | 1.1 |
| 55 | 1BM8A | 99 | 1.71 |
| 56 | 1BNDB | 108 | 2.3 |
| 57 | 1BTEA | 92 | 1.5 |
| 58 | 1BTNA | 106 | 2 |
| 59 | 1BUOA | 121 | 1.9 |
| 60 | 1BVYF | 152 | 2.03 |
| 61 | 1BWNB | 161 | 2.1 |
| 62 | 1BX7A | 51 | 1.2 |
| 63 | 1BXOA | 323 | 0.95 |
| 64 | 1BYIA | 224 | 0.97 |
| 65 | 1BYRA | 152 | 2 |
| 66 | 1C44A | 123 | 1.8 |
| 67 | 1C4QB | 69 | 1.52 |
| 68 | 1C5EB | 95 | 1.1 |
| 69 | 1C75A | 71 | 0.97 |
| 70 | 1C94A | 37 | 2.08 |
| 71 | 1C9OA | 66 | 1.17 |
| 72 | 1CC8A | 72 | 1.02 |
| 73 | 1CCWA | 137 | 1.6 |
| 74 | 1CEWI | 108 | 2 |
| 75 | 1CFRA | 283 | 2.15 |
| 76 | 1CG5B | 141 | 1.6 |
| 77 | 1CKNB | 317 | 2.5 |
| 78 | 1CKSC | 78 | 2.1 |
| 79 | 1CKTA | 71 | 2.5 |
| 80 | 1CLVI | 32 | 2 |
| 81 | 1CMCB | 104 | 1.8 |
| 82 | 1CN3F | 29 | 2.2 |
| 83 | 1CNUA | 133 | 2.25 |
| 84 | 1CR5A | 178 | 2.3 |
| 85 | 1CSEI | 63 | 1.2 |
| 86 | 1CV8A | 173 | 1.75 |
| 87 | 1CXQA | 143 | 1.02 |
| 88 | 1CXYA | 81 | 1.65 |
| 89 | 1D0DA | 60 | 1.62 |
| 90 | 1D2SA | 170 | 1.55 |
| 91 | 1D3BK | 71 | 2 |
| 92 | 1D3BL | 90 | 2 |
| 93 | 1D4OA | 177 | 1.21 |
| 94 | 1D4VA | 117 | 2.2 |
| 95 | 1D8DA | 323 | 2 |
| 96 | 1D9CA | 121 | 2 |
| 97 | 1DCSA | 279 | 1.3 |
| 98 | 1DEBA | 54 | 2.4 |
| 99 | 1DEUA | 275 | 1.7 |
| 100 | 1DFMB | 218 | 1.5 |
| 101 | 1DG6A | 149 | 1.3 |
| 102 | 1DHNA | 121 | 1.65 |
| 103 | 1DI2B | 60 | 1.9 |
| 104 | 1DM1A | 146 | 1.99 |
| 105 | 1DM9B | 107 | 2 |
| 106 | 1DOIA | 128 | 1.9 |
| 107 | 1DOWB | 32 | 1.8 |
| 108 | 1DPJB | 29 | 1.8 |
| 109 | 1DQGA | 134 | 1.7 |
| 110 | 1DQPB | 230 | 1.75 |
| 111 | 1DQTA | 117 | 2 |
| 112 | 1DTDB | 61 | 1.65 |
| 113 | 1DTJB | 62 | 2 |
| 114 | 1DVKB | 150 | 2.15 |
| 115 | 1DY5A | 124 | 0.87 |
| 116 | 1DYOA | 156 | 2.1 |
| 117 | 1DYTA | 133 | 1.75 |
| 118 | 1DZKB | 148 | 1.48 |
| 119 | 1E29A | 135 | 1.21 |
| 120 | 1E3OC | 132 | 1.9 |
| 121 | 1E5KA | 188 | 1.35 |
| 122 | 1E7SA | 314 | 1.5 |
| 123 | 1E9GA | 284 | 1.15 |
| 124 | 1EAID | 61 | 2.4 |
| 125 | 1EAJB | 120 | 1.35 |
| 126 | 1EAZA | 103 | 1.4 |
| 127 | 1EB6A | 177 | 1 |
| 128 | 1ECAA | 136 | 1.4 |
| 129 | 1ECSB | 120 | 1.7 |
| 130 | 1EDMC | 39 | 1.5 |
| 131 | 1EDYA | 134 | 2.3 |
| 132 | 1EF1B | 294 | 1.9 |
| 133 | 1EF1D | 90 | 1.9 |
| 134 | 1EF8A | 256 | 1.85 |
| 135 | 1EGIB | 143 | 2.3 |
| 136 | 1EGWA | 71 | 1.5 |
| 137 | 1EJ0A | 180 | 1.5 |
| 138 | 1EJ2A | 167 | 1.9 |
| 139 | 1EJDA | 67 | 1.55 |
| 140 | 1EJFB | 110 | 2.49 |
| 141 | 1EJGA | 46 | 0.54 |
| 142 | 1EJXA | 100 | 1.6 |
| 143 | 1EK6A | 346 | 1.5 |
| 144 | 1EKQA | 254 | 1.5 |
| 145 | 1ELWA | 117 | 1.6 |
| 146 | 1EP3B | 261 | 2.1 |
| 147 | 1ES9A | 212 | 1.3 |
| 148 | 1ESCA | 302 | 2.1 |
| 149 | 1ET1A | 34 | 0.9 |
| 150 | 1EUVA | 221 | 1.6 |
| 151 | 1EUWA | 136 | 1.05 |
| 152 | 1EW0A | 130 | 1.4 |
| 153 | 1EW4A | 106 | 1.4 |
| 154 | 1EXRA | 146 | 1 |
| 155 | 1EXTB | 158 | 1.85 |
| 156 | 1EXZB | 140 | 2.3 |
| 157 | 1EYHA | 144 | 1.56 |
| 158 | 1EYQB | 212 | 1.85 |
| 159 | 1EZGB | 82 | 1.4 |
| 160 | 1F00I | 282 | 1.9 |
| 161 | 1F08A | 148 | 1.9 |
| 162 | 1F0LB | 522 | 1.55 |
| 163 | 1F1EA | 151 | 1.37 |
| 164 | 1F2LD | 70 | 2 |
| 165 | 1F35B | 162 | 2.3 |
| 166 | 1F3AB | 221 | 1.9 |
| 167 | 1F3MB | 70 | 2.3 |
| 168 | 1F3UB | 139 | 1.7 |
| 169 | 1F4PA | 147 | 1.3 |
| 170 | 1F5VB | 240 | 1.7 |
| 171 | 1F60B | 90 | 1.67 |
| 172 | 1F61A | 418 | 2 |
| 173 | 1F7LA | 118 | 1.5 |
| 174 | 1F86B | 115 | 1.1 |
| 175 | 1F8YB | 156 | 2.4 |
| 176 | 1F94A | 63 | 0.97 |
| 177 | 1FASA | 61 | 1.8 |
| 178 | 1FC3C | 116 | 2 |
| 179 | 1FD3A | 41 | 1.35 |
| 180 | 1FEWA | 173 | 2.2 |
| 181 | 1FG7A | 354 | 1.5 |
| 182 | 1FGYA | 127 | 1.5 |
| 183 | 1FIAA | 79 | 2 |
| 184 | 1FJ2A | 229 | 1.5 |
| 185 | 1FM0E | 142 | 1.45 |
| 186 | 1FNFA | 368 | 2 |
| 187 | 1FO0B | 112 | 2.5 |
| 188 | 1FQJC | 38 | 2.02 |
| 189 | 1FQJE | 141 | 2.02 |
| 190 | 1FR3H | 67 | 1.5 |
| 191 | 1FS1A | 41 | 1.8 |
| 192 | 1FSGA | 233 | 1.05 |
| 193 | 1FT5A | 211 | 1.6 |
| 194 | 1FVKA | 188 | 1.7 |
| 195 | 1FX4A | 231 | 1.9 |
| 196 | 1FXKC | 133 | 2.3 |
| 197 | 1FYHE | 201 | 2.04 |
| 198 | 1G1SA | 158 | 1.9 |
| 199 | 1G2BA | 62 | 1.12 |
| 200 | 1G2RA | 94 | 1.35 |
| 201 | 1G2YD | 30 | 1 |
| 202 | 1G31A | 107 | 2.3 |
| 203 | 1G4YB | 81 | 1.6 |
| 204 | 1G5HC | 405 | 1.95 |
| 205 | 1G60B | 228 | 1.74 |
| 206 | 1G61B | 225 | 1.3 |
| 207 | 1G66A | 207 | 0.9 |
| 208 | 1G6GA | 127 | 1.6 |
| 209 | 1G6XA | 58 | 0.86 |
| 210 | 1G8EA | 98 | 1.8 |
| 211 | 1G8MA | 590 | 1.75 |
| 212 | 1GA6A | 369 | 1 |
| 213 | 1GA8A | 278 | 2 |
| 214 | 1GCIA | 269 | 0.78 |
| 215 | 1GCQC | 69 | 1.68 |
| 216 | 1GD2I | 40 | 2 |
| 217 | 1GK4C | 70 | 2.3 |
| 218 | 1GK7A | 39 | 1.4 |
| 219 | 1GK8A | 469 | 1.4 |
| 220 | 1GK8I | 126 | 1.4 |
| 221 | 1GK9A | 208 | 1.3 |
| 222 | 1GKMA | 507 | 1 |
| 223 | 1GKPE | 458 | 1.29 |
| 224 | 1GL2B | 60 | 1.9 |
| 225 | 1GL2C | 60 | 1.9 |
| 226 | 1GL4A | 273 | 2 |
| 227 | 1GL4B | 89 | 2 |
| 228 | 1GMLA | 154 | 2.2 |
| 229 | 1GMWD | 138 | 1.5 |
| 230 | 1GMXA | 108 | 1.1 |
| 231 | 1GNYA | 153 | 1.63 |
| 232 | 1GO4G | 100 | 2.05 |
| 233 | 1GP0A | 133 | 1.4 |
| 234 | 1GPPA | 217 | 1.35 |
| 235 | 1GPQB | 128 | 1.6 |
| 236 | 1GQ1B | 559 | 1.4 |
| 237 | 1GQPA | 180 | 2.2 |
| 238 | 1GR3A | 132 | 2 |
| 239 | 1GTZB | 149 | 1.6 |
| 240 | 1GU2A | 124 | 1.19 |
| 241 | 1GUQA | 347 | 1.8 |
| 242 | 1GVDA | 52 | 1.45 |
| 243 | 1GVEB | 301 | 1.38 |
| 244 | 1GVNC | 85 | 1.95 |
| 245 | 1GVPA | 87 | 1.6 |
| 246 | 1GWEA | 498 | 0.88 |
| 247 | 1GWMA | 153 | 1.15 |
| 248 | 1GXMB | 332 | 1.32 |
| 249 | 1GXRB | 324 | 1.65 |
| 250 | 1GXUA | 88 | 1.27 |
| 251 | 1GXYB | 223 | 1.71 |
| 252 | 1GY6B | 123 | 1.6 |
| 253 | 1GYXB | 76 | 1.35 |
| 254 | 1GZ8A | 291 | 1.3 |
| 255 | 1GZRB | 28 | 2 |
| 256 | 1GZSB | 165 | 2.3 |
| 257 | 1H03Q | 125 | 1.7 |
| 258 | 1H0HL | 214 | 1.8 |
| 259 | 1H16A | 759 | 1.53 |
| 260 | 1H1NA | 305 | 1.12 |
| 261 | 1H2CA | 124 | 1.6 |
| 262 | 1H3LA | 75 | 2.37 |
| 263 | 1H3OA | 49 | 2.3 |
| 264 | 1H3OB | 74 | 2.3 |
| 265 | 1H41B | 701 | 1.5 |
| 266 | 1H4AX | 173 | 1.15 |
| 267 | 1H4XB | 111 | 1.16 |
| 268 | 1H64Z | 71 | 1.9 |
| 269 | 1H6HA | 143 | 1.7 |
| 270 | 1H72C | 296 | 1.8 |
| 271 | 1H7CA | 103 | 1.8 |
| 272 | 1H7EA | 245 | 1.83 |
| 273 | 1H8EH | 89 | 2 |
| 274 | 1H8PA | 88 | 1.82 |
| 275 | 1H8UA | 115 | 1.8 |
| 276 | 1H97A | 147 | 1.17 |
| 277 | 1HBKA | 89 | 2 |
| 278 | 1HBNA | 547 | 1.16 |
| 279 | 1HBNF | 247 | 1.16 |
| 280 | 1HCRA | 52 | 2.3 |
| 281 | 1HDKA | 139 | 1.8 |
| 282 | 1HDOA | 205 | 1.15 |
| 283 | 1HFES | 88 | 1.6 |
| 284 | 1HFOF | 113 | 1.65 |
| 285 | 1HL6B | 137 | 2.5 |
| 286 | 1HLQC | 74 | 1.45 |
| 287 | 1HMCA | 148 | 2.5 |
| 288 | 1HNJA | 317 | 1.46 |
| 289 | 1HQ1A | 76 | 1.52 |
| 290 | 1HQZ4 | 137 | 2.1 |
| 291 | 1HT6A | 404 | 1.5 |
| 292 | 1HTRP | 43 | 1.62 |
| 293 | 1HW7A | 229 | 2.2 |
| 294 | 1HX0A | 496 | 1.38 |
| 295 | 1HXAA | 528 | 2.32 |
| 296 | 1HXRB | 115 | 1.65 |
| 297 | 1HY5A | 120 | 2.25 |
| 298 | 1HZTA | 153 | 1.45 |
| 299 | 1I07A | 59 | 1.8 |
| 300 | 1I0VA | 104 | 1.23 |
| 301 | 1I12D | 157 | 1.3 |
| 302 | 1I1JB | 104 | 1.39 |
| 303 | 1I1KC | 298 | 2.1 |
| 304 | 1I1WA | 303 | 0.89 |
| 305 | 1I24A | 392 | 1.2 |
| 306 | 1I27A | 73 | 1.02 |
| 307 | 1I2HA | 138 | 1.8 |
| 308 | 1I2TA | 61 | 1.04 |
| 309 | 1I4DA | 188 | 2.5 |
| 310 | 1I4UA | 181 | 1.15 |
| 311 | 1I5NB | 125 | 2.14 |
| 312 | 1I71A | 83 | 1.45 |
| 313 | 1I7EA | 237 | 1.95 |
| 314 | 1I7NB | 308 | 1.9 |
| 315 | 1I7WD | 60 | 2 |
| 316 | 1I8NA | 89 | 2.2 |
| 317 | 1I9SA | 189 | 1.65 |
| 318 | 1IAKA | 182 | 1.9 |
| 319 | 1IARB | 188 | 2.3 |
| 320 | 1IAZA | 175 | 1.9 |
| 321 | 1ICFI | 65 | 2 |
| 322 | 1IDPA | 147 | 1.45 |
| 323 | 1IFCA | 131 | 1.19 |
| 324 | 1IFRA | 113 | 1.4 |
| 325 | 1IGQA | 54 | 1.7 |
| 326 | 1IJYA | 122 | 1.35 |
| 327 | 1IK7A | 52 | 2.3 |
| 328 | 1ILR1 | 145 | 2.1 |
| 329 | 1IM3P | 95 | 2.2 |
| 330 | 1IMJA | 208 | 2.2 |
| 331 | 1INLA | 285 | 1.5 |
| 332 | 1IO0A | 166 | 1.45 |
| 333 | 1IOOA | 196 | 1.55 |
| 334 | 1IQ4B | 179 | 1.8 |
| 335 | 1IQZA | 81 | 0.92 |
| 336 | 1IRQA | 48 | 1.5 |
| 337 | 1IS1A | 185 | 2.2 |
| 338 | 1ISIA | 250 | 2.1 |
| 339 | 1ISUA | 62 | 1.5 |
| 340 | 1IT2B | 146 | 1.6 |
| 341 | 1ITVA | 195 | 1.95 |
| 342 | 1ITXA | 419 | 1.1 |
| 343 | 1IX9A | 205 | 0.9 |
| 344 | 1IXLA | 130 | 1.94 |
| 345 | 1J0PA | 108 | 0.91 |
| 346 | 1J2LA | 68 | 1.7 |
| 347 | 1J3AA | 129 | 1.6 |
| 348 | 1J7DA | 140 | 1.85 |
| 349 | 1J98A | 154 | 1.2 |
| 350 | 1J9BA | 138 | 1.26 |
| 351 | 1JB0C | 80 | 2.5 |
| 352 | 1JB0E | 69 | 2.5 |
| 353 | 1JB0I | 38 | 2.5 |
| 354 | 1JB0J | 41 | 2.5 |
| 355 | 1JB0M | 31 | 2.5 |
| 356 | 1JB0X | 29 | 2.5 |
| 357 | 1JBEA | 126 | 1.08 |
| 358 | 1JC4D | 145 | 2 |
| 359 | 1JCDC | 50 | 1.3 |
| 360 | 1JDHB | 38 | 1.9 |
| 361 | 1JEKA | 40 | 1.5 |
| 362 | 1JEKB | 34 | 1.5 |
| 363 | 1JERA | 110 | 1.6 |
| 364 | 1JF8A | 130 | 1.12 |
| 365 | 1JFBA | 399 | 1 |
| 366 | 1JG1A | 216 | 1.2 |
| 367 | 1JH6A | 181 | 1.8 |
| 368 | 1JHGA | 101 | 1.3 |
| 369 | 1JI7C | 85 | 1.45 |
| 370 | 1JIFB | 122 | 1.6 |
| 371 | 1JIXA | 351 | 1.65 |
| 372 | 1JKGA | 139 | 1.9 |
| 373 | 1JKVF | 266 | 1.39 |
| 374 | 1JLYB | 299 | 2.2 |
| 375 | 1JM1A | 202 | 1.11 |
| 376 | 1JMVA | 140 | 1.85 |
| 377 | 1JNRD | 149 | 1.6 |
| 378 | 1JO0B | 96 | 1.37 |
| 379 | 1JO8A | 58 | 1.3 |
| 380 | 1JR7A | 306 | 2 |
| 381 | 1JR8B | 105 | 1.5 |
| 382 | 1JS3B | 464 | 2.25 |
| 383 | 1JSUC | 69 | 2.3 |
| 384 | 1JT2A | 255 | 1.8 |
| 385 | 1JUQD | 159 | 2.2 |
| 386 | 1JW9B | 240 | 1.7 |
| 387 | 1JX6A | 338 | 1.5 |
| 388 | 1JXOB | 264 | 2.3 |
| 389 | 1JY2N | 43 | 1.4 |
| 390 | 1JY2O | 51 | 1.4 |
| 391 | 1JY2P | 44 | 1.4 |
| 392 | 1JY5A | 203 | 2.05 |
| 393 | 1JYKA | 229 | 1.5 |
| 394 | 1JYOD | 130 | 1.9 |
| 395 | 1JZTB | 243 | 1.94 |
| 396 | 1K0DB | 231 | 2.2 |
| 397 | 1K1FE | 65 | 2.2 |
| 398 | 1K3IA | 651 | 1.4 |
| 399 | 1K3XA | 253 | 1.25 |
| 400 | 1K4IA | 216 | 0.98 |
| 401 | 1K4ZA | 157 | 2.3 |
| 402 | 1K52A | 72 | 1.8 |
| 403 | 1K55A | 245 | 1.39 |
| 404 | 1K5CA | 333 | 0.96 |
| 405 | 1K5NA | 276 | 1.09 |
| 406 | 1K5NB | 100 | 1.09 |
| 407 | 1K61D | 58 | 2.1 |
| 408 | 1K66B | 149 | 1.75 |
| 409 | 1K78I | 58 | 2.25 |
| 410 | 1K7CA | 233 | 1.12 |
| 411 | 1K94A | 165 | 1.7 |
| 412 | 1KAFA | 108 | 1.6 |
| 413 | 1KBAA | 66 | 2.3 |
| 414 | 1KCFB | 228 | 2.3 |
| 415 | 1KD8B | 35 | 1.9 |
| 416 | 1KGDA | 175 | 1.31 |
| 417 | 1KHXA | 203 | 1.8 |
| 418 | 1KJQB | 385 | 1.05 |
| 419 | 1KL9A | 168 | 1.9 |
| 420 | 1KLLA | 128 | 1.5 |
| 421 | 1KLXA | 133 | 1.95 |
| 422 | 1KMQA | 177 | 1.55 |
| 423 | 1KMVA | 185 | 1.05 |
| 424 | 1KN1A | 160 | 2.2 |
| 425 | 1KNGA | 144 | 1.14 |
| 426 | 1KNMA | 129 | 1.2 |
| 427 | 1KNQB | 171 | 2 |
| 428 | 1KP6A | 79 | 1.8 |
| 429 | 1KQ1W | 61 | 1.55 |
| 430 | 1KQ3A | 364 | 1.5 |
| 431 | 1KQ6A | 140 | 1.18 |
| 432 | 1KQPA | 271 | 1.03 |
| 433 | 1KT7A | 175 | 1.27 |
| 434 | 1KTHA | 58 | 0.95 |
| 435 | 1KVEA | 63 | 1.8 |
| 436 | 1KVEB | 77 | 1.8 |
| 437 | 1KW4A | 70 | 1.75 |
| 438 | 1KWFA | 363 | 0.94 |
| 439 | 1KWIA | 85 | 2.19 |
| 440 | 1KYFA | 247 | 1.22 |
| 441 | 1KZLA | 202 | 2.1 |
| 442 | 1KZQA | 253 | 1.7 |
| 443 | 1L2HA | 144 | 1.54 |
| 444 | 1L2UA | 224 | 2.5 |
| 445 | 1L3PA | 102 | 1.98 |
| 446 | 1L4IB | 189 | 2.2 |
| 447 | 1L5PC | 93 | 2.2 |
| 448 | 1L6PA | 121 | 1.65 |
| 449 | 1L8RA | 101 | 1.65 |
| 450 | 1L9LA | 74 | 0.92 |
| 451 | 1LBUA | 213 | 1.8 |
| 452 | 1LC5A | 355 | 1.46 |
| 453 | 1LCSB | 205 | 2.5 |
| 454 | 1LDDA | 74 | 2 |
| 455 | 1LGHK | 43 | 2.4 |
| 456 | 1LIHA | 160 | 2.2 |
| 457 | 1LKEA | 157 | 1.9 |
| 458 | 1LKKA | 105 | 1 |
| 459 | 1LM8C | 88 | 1.85 |
| 460 | 1LNIA | 96 | 1 |
| 461 | 1LO7A | 140 | 1.5 |
| 462 | 1LPBA | 85 | 2.46 |
| 463 | 1LQTA | 452 | 1.05 |
| 464 | 1LQVB | 173 | 1.6 |
| 465 | 1LR7A | 73 | 1.5 |
| 466 | 1LSHB | 174 | 1.9 |
| 467 | 1LSLA | 113 | 1.9 |
| 468 | 1LSTA | 239 | 1.8 |
| 469 | 1LTZA | 274 | 1.4 |
| 470 | 1LU0B | 29 | 1.03 |
| 471 | 1LU4A | 134 | 1.12 |
| 472 | 1LUCA | 326 | 1.5 |
| 473 | 1LUGA | 259 | 0.95 |
| 474 | 1LUZB | 83 | 1.8 |
| 475 | 1LVBA | 214 | 2.2 |
| 476 | 1LWBA | 122 | 1.05 |
| 477 | 1LY2A | 130 | 1.8 |
| 478 | 1LYVA | 283 | 1.36 |
| 479 | 1M0DA | 129 | 1.9 |
| 480 | 1M0KA | 222 | 1.43 |
| 481 | 1M15A | 356 | 1.2 |
| 482 | 1M1EB | 65 | 2.1 |
| 483 | 1M1FB | 105 | 1.4 |
| 484 | 1M1QA | 90 | 0.97 |
| 485 | 1M2DA | 101 | 1.05 |
| 486 | 1M2XA | 219 | 1.5 |
| 487 | 1M3SA | 181 | 1.95 |
| 488 | 1M40A | 263 | 0.85 |
| 489 | 1M45B | 25 | 1.65 |
| 490 | 1M4IA | 181 | 1.5 |
| 491 | 1M4LA | 307 | 1.25 |
| 492 | 1M4RA | 142 | 2 |
| 493 | 1M56C | 265 | 2.3 |
| 494 | 1M56D | 42 | 2.3 |
| 495 | 1M5Q2 | 127 | 2 |
| 496 | 1M70A | 190 | 1.25 |
| 497 | 1M8AB | 61 | 1.7 |
| 498 | 1M93A | 46 | 1.65 |
| 499 | 1M93C | 31 | 1.65 |
| 500 | 1M9ZA | 105 | 1.05 |
| 501 | 1MAIA | 119 | 1.9 |
| 502 | 1MB3A | 117 | 1.41 |
| 503 | 1MBYA | 75 | 2 |
| 504 | 1MC2A | 122 | 0.85 |
| 505 | 1MDOA | 365 | 1.7 |
| 506 | 1ME4A | 215 | 1.2 |
| 507 | 1MF7A | 194 | 1.25 |
| 508 | 1MFMA | 153 | 1.02 |
| 509 | 1MFTB | 51 | 2.5 |
| 510 | 1MG7B | 352 | 1.55 |
| 511 | 1MGTA | 169 | 1.8 |
| 512 | 1MHMB | 54 | 2.3 |
| 513 | 1MIDA | 91 | 1.71 |
| 514 | 1MIJA | 139 | 2.05 |
| 515 | 1MJ4A | 79 | 1.2 |
| 516 | 1MJ5A | 297 | 0.95 |
| 517 | 1MJUH | 212 | 1.22 |
| 518 | 1MJUL | 219 | 1.22 |
| 519 | 1MKFB | 371 | 2.1 |
| 520 | 1MKKA | 93 | 1.32 |
| 521 | 1MKYA | 407 | 1.9 |
| 522 | 1MMLA | 251 | 1.8 |
| 523 | 1MN8D | 97 | 1 |
| 524 | 1MNMB | 81 | 2.25 |
| 525 | 1MOFA | 53 | 1.7 |
| 526 | 1MOWG | 233 | 2.4 |
| 527 | 1MOXC | 49 | 2.5 |
| 528 | 1MSKA | 327 | 1.8 |
| 529 | 1MSPB | 122 | 2.5 |
| 530 | 1MUNA | 225 | 1.2 |
| 531 | 1MVFE | 44 | 1.65 |
| 532 | 1MVLA | 173 | 2 |
| 533 | 1MWPA | 96 | 1.8 |
| 534 | 1MWQA | 100 | 0.99 |
| 535 | 1MXEE | 25 | 1.7 |
| 536 | 1MXRB | 339 | 1.42 |
| 537 | 1MY7A | 107 | 1.49 |
| 538 | 1MZ9A | 45 | 1.7 |
| 539 | 1MZGB | 143 | 2 |
| 540 | 1MZWB | 31 | 2 |
| 541 | 1N0QB | 93 | 1.26 |
| 542 | 1N0WB | 33 | 1.7 |
| 543 | 1N12C | 136 | 1.87 |
| 544 | 1N1FA | 153 | 1.95 |
| 545 | 1N1JA | 87 | 1.67 |
| 546 | 1N1JB | 78 | 1.67 |
| 547 | 1N2FA | 142 | 2.01 |
| 548 | 1N4WA | 498 | 0.92 |
| 549 | 1N62E | 796 | 1.09 |
| 550 | 1N62F | 286 | 1.09 |
| 551 | 1N67A | 332 | 1.9 |
| 552 | 1N6JG | 26 | 2.2 |
| 553 | 1N7HB | 334 | 1.8 |
| 554 | 1N7OA | 721 | 1.5 |
| 555 | 1N7SA | 63 | 1.45 |
| 556 | 1N7SB | 68 | 1.45 |
| 557 | 1N7SD | 66 | 1.45 |
| 558 | 1N81A | 186 | 2.1 |
| 559 | 1NC7C | 116 | 1.55 |
| 560 | 1NCQD | 40 | 2.5 |
| 561 | 1NEZH | 120 | 2.1 |
| 562 | 1NF8A | 207 | 1.6 |
| 563 | 1NFPA | 228 | 1.6 |
| 564 | 1NH2C | 50 | 1.9 |
| 565 | 1NH9A | 80 | 2 |
| 566 | 1NKDA | 59 | 1.09 |
| 567 | 1NKIA | 134 | 0.95 |
| 568 | 1NKZF | 41 | 2 |
| 569 | 1NLQD | 94 | 1.5 |
| 570 | 1NLSA | 237 | 0.94 |
| 571 | 1NMLA | 316 | 2.2 |
| 572 | 1NNXA | 93 | 1.45 |
| 573 | 1NO1C | 67 | 2.4 |
| 574 | 1NOFA | 383 | 1.42 |
| 575 | 1NOWA | 480 | 2.2 |
| 576 | 1NOXA | 200 | 1.59 |
| 577 | 1NP6B | 169 | 1.9 |
| 578 | 1NPIA | 61 | 1.16 |
| 579 | 1NQJB | 114 | 1 |
| 580 | 1NQUD | 154 | 1.75 |
| 581 | 1NR4A | 67 | 1.72 |
| 582 | 1NRGA | 213 | 1.95 |
| 583 | 1NRVB | 100 | 1.65 |
| 584 | 1NTVA | 152 | 1.5 |
| 585 | 1NU4A | 91 | 1.8 |
| 586 | 1NUYA | 328 | 1.3 |
| 587 | 1NWAA | 168 | 1.5 |
| 588 | 1NWWA | 145 | 1.2 |
| 589 | 1NWZA | 125 | 0.82 |
| 590 | 1NXMA | 194 | 1.3 |
| 591 | 1NYCA | 111 | 1.4 |
| 592 | 1NYKA | 156 | 1.31 |
| 593 | 1NZ0A | 109 | 1.2 |
| 594 | 1NZIA | 155 | 1.5 |
| 595 | 1O04G | 494 | 1.42 |
| 596 | 1O3UA | 120 | 1.75 |
| 597 | 1O4YA | 270 | 1.48 |
| 598 | 1O50A | 141 | 1.87 |
| 599 | 1O58B | 285 | 1.8 |
| 600 | 1O63B | 200 | 2 |
| 601 | 1O6AA | 87 | 1.85 |
| 602 | 1O6UC | 64 | 2.05 |
| 603 | 1O6VB | 460 | 1.5 |
| 604 | 1O7IB | 114 | 1.2 |
| 605 | 1O7JC | 325 | 1 |
| 606 | 1O7QA | 287 | 1.3 |
| 607 | 1O7ZA | 60 | 1.92 |
| 608 | 1O82D | 70 | 1.46 |
| 609 | 1O8XA | 143 | 1.3 |
| 610 | 1O9GA | 249 | 1.5 |
| 611 | 1O9WA | 169 | 1.65 |
| 612 | 1OA8A | 128 | 1.7 |
| 613 | 1OAIA | 59 | 1 |
| 614 | 1OB9A | 124 | 2 |
| 615 | 1OC0B | 37 | 2.28 |
| 616 | 1OC7A | 364 | 1.11 |
| 617 | 1OD3A | 131 | 1 |
| 618 | 1OD6A | 155 | 1.5 |
| 619 | 1ODMA | 329 | 1.15 |
| 620 | 1OF8B | 344 | 1.5 |
| 621 | 1OFWB | 292 | 1.5 |
| 622 | 1OH4A | 174 | 1.35 |
| 623 | 1OHEA | 338 | 2.2 |
| 624 | 1OI0A | 108 | 1.5 |
| 625 | 1OI7A | 270 | 1.23 |
| 626 | 1OIHD | 244 | 1.89 |
| 627 | 1OJ5A | 105 | 2.2 |
| 628 | 1OJHL | 50 | 1.8 |
| 629 | 1OK0A | 74 | 0.93 |
| 630 | 1OKOA | 121 | 1.6 |
| 631 | 1OKSA | 53 | 1.8 |
| 632 | 1OLLA | 188 | 1.93 |
| 633 | 1OMZA | 253 | 2.1 |
| 634 | 1OOHB | 126 | 1.25 |
| 635 | 1OOTA | 58 | 1.39 |
| 636 | 1OPDA | 85 | 1.5 |
| 637 | 1OQJB | 89 | 1.55 |
| 638 | 1OQVA | 171 | 1.3 |
| 639 | 1OR7F | 64 | 2 |
| 640 | 1ORJD | 125 | 2.25 |
| 641 | 1ORUA | 182 | 1.8 |
| 642 | 1ORYB | 40 | 2.45 |
| 643 | 1OSYB | 112 | 1.7 |
| 644 | 1OTFA | 59 | 1.9 |
| 645 | 1OTKB | 244 | 2 |
| 646 | 1OU0A | 190 | 2.1 |
| 647 | 1OUWD | 151 | 1.37 |
| 648 | 1OW1A | 167 | 1.8 |
| 649 | 1OWFB | 94 | 1.95 |
| 650 | 1OXKE | 156 | 2.1 |
| 651 | 1OZ2A | 324 | 1.55 |
| 652 | 1P0ZJ | 131 | 1.6 |
| 653 | 1P1JB | 516 | 1.7 |
| 654 | 1P1MA | 405 | 1.5 |
| 655 | 1P1XA | 250 | 0.99 |
| 656 | 1P28A | 119 | 1.7 |
| 657 | 1P3QR | 36 | 1.7 |
| 658 | 1P4XA | 250 | 2.2 |
| 659 | 1P57A | 110 | 1.75 |
| 660 | 1P5UC | 130 | 1.99 |
| 661 | 1P5ZB | 229 | 1.6 |
| 662 | 1P6OB | 161 | 1.14 |
| 663 | 1P9GA | 41 | 0.84 |
| 664 | 1P9IA | 29 | 1.17 |
| 665 | 1PA2A | 306 | 1.45 |
| 666 | 1PBJA | 120 | 1.4 |
| 667 | 1PBYC | 79 | 1.7 |
| 668 | 1PE9A | 361 | 1.6 |
| 669 | 1PFBA | 55 | 1.4 |
| 670 | 1PI1A | 185 | 2 |
| 671 | 1PK3C | 78 | 1.85 |
| 672 | 1PLCA | 99 | 1.33 |
| 673 | 1PM4C | 117 | 1.75 |
| 674 | 1PMHX | 183 | 1.06 |
| 675 | 1PN4C | 247 | 2.35 |
| 676 | 1POCA | 134 | 2 |
| 677 | 1PP0A | 191 | 1.42 |
| 678 | 1PP7U | 114 | 2.45 |
| 679 | 1PPJJ | 32 | 2.1 |
| 680 | 1PQ5A | 224 | 0.85 |
| 681 | 1PQ9D | 219 | 2.1 |
| 682 | 1PQHB | 115 | 1.29 |
| 683 | 1PRZA | 252 | 1.8 |
| 684 | 1PSRB | 100 | 1.05 |
| 685 | 1PTQA | 50 | 1.95 |
| 686 | 1PV5A | 261 | 1.75 |
| 687 | 1PWGA | 345 | 1.07 |
| 688 | 1PYAE | 81 | 2.5 |
| 689 | 1PYBD | 107 | 2.5 |
| 690 | 1PYOB | 98 | 1.65 |
| 691 | 1PZ4A | 113 | 1.35 |
| 692 | 1PZXA | 277 | 2 |
| 693 | 1Q08A | 94 | 1.9 |
| 694 | 1Q0QA | 398 | 1.9 |
| 695 | 1Q1FA | 148 | 1.5 |
| 696 | 1Q33A | 292 | 1.81 |
| 697 | 1Q35A | 317 | 1.2 |
| 698 | 1Q40A | 165 | 1.95 |
| 699 | 1Q4UA | 140 | 1.6 |
| 700 | 1Q5YD | 80 | 1.4 |
| 701 | 1Q6OB | 215 | 1.2 |
| 702 | 1Q7LC | 190 | 1.4 |
| 703 | 1Q8BA | 93 | 1.9 |
| 704 | 1Q8CA | 132 | 2 |
| 705 | 1Q8DA | 100 | 1.8 |
| 706 | 1Q9BA | 43 | 1.5 |
| 707 | 1QADA | 104 | 1.8 |
| 708 | 1QAUA | 112 | 1.25 |
| 709 | 1QB5D | 99 | 1.9 |
| 710 | 1QBZB | 114 | 1.47 |
| 711 | 1QCAA | 211 | 2.2 |
| 712 | 1QCSA | 195 | 1.9 |
| 713 | 1QCXA | 359 | 1.7 |
| 714 | 1QDDA | 144 | 1.3 |
| 715 | 1QFOC | 114 | 1.85 |
| 716 | 1QFTB | 169 | 1.25 |
| 717 | 1QGKB | 44 | 2.5 |
| 718 | 1QGQA | 238 | 1.5 |
| 719 | 1QHHD | 96 | 2.5 |
| 720 | 1QHVA | 195 | 1.51 |
| 721 | 1QHWA | 300 | 2.2 |
| 722 | 1QIPD | 183 | 1.72 |
| 723 | 1QL0B | 241 | 1.1 |
| 724 | 1QLWB | 317 | 1.09 |
| 725 | 1QMPB | 121 | 2 |
| 726 | 1QNRA | 344 | 1.4 |
| 727 | 1QOPA | 265 | 1.4 |
| 728 | 1QOPB | 390 | 1.4 |
| 729 | 1QQ5B | 245 | 1.52 |
| 730 | 1QQFA | 277 | 1.45 |
| 731 | 1QQP4 | 46 | 1.9 |
| 732 | 1QR0A | 228 | 1.9 |
| 733 | 1QRVA | 73 | 2.2 |
| 734 | 1QSAA | 618 | 1.65 |
| 735 | 1QSDB | 102 | 2.2 |
| 736 | 1QTNB | 90 | 1.2 |
| 737 | 1QTWA | 285 | 1.02 |
| 738 | 1QV1A | 187 | 1.1 |
| 739 | 1QVEA | 126 | 1.54 |
| 740 | 1QVYA | 138 | 1.6 |
| 741 | 1QVZA | 236 | 1.85 |
| 742 | 1QW2A | 102 | 1.5 |
| 743 | 1QWYA | 234 | 1.3 |
| 744 | 1QWZA | 235 | 1.75 |
| 745 | 1QYSA | 92 | 2.5 |
| 746 | 1QZMA | 94 | 1.9 |
| 747 | 1QZQB | 437 | 2.4 |
| 748 | 1R0UA | 142 | 1.75 |
| 749 | 1R1QA | 97 | 1.8 |
| 750 | 1R1UA | 94 | 2 |
| 751 | 1R26A | 113 | 1.4 |
| 752 | 1R29A | 122 | 1.3 |
| 753 | 1R2QA | 170 | 1.05 |
| 754 | 1R45D | 200 | 1.57 |
| 755 | 1R4PA | 281 | 1.77 |
| 756 | 1R4VA | 151 | 1.9 |
| 757 | 1R6DA | 322 | 1.35 |
| 758 | 1R6JA | 82 | 0.73 |
| 759 | 1R75A | 110 | 1.86 |
| 760 | 1R77A | 99 | 1.75 |
| 761 | 1R7AB | 504 | 1.77 |
| 762 | 1R7JA | 90 | 1.47 |
| 763 | 1R7LA | 103 | 2 |
| 764 | 1R7MA | 223 | 2.25 |
| 765 | 1R8GA | 352 | 2.15 |
| 766 | 1R8HF | 83 | 1.9 |
| 767 | 1R8NA | 185 | 1.75 |
| 768 | 1R8SA | 160 | 1.46 |
| 769 | 1R9LA | 309 | 1.59 |
| 770 | 1R9WA | 138 | 1.8 |
| 771 | 1RA0A | 423 | 1.12 |
| 772 | 1RC9A | 221 | 1.6 |
| 773 | 1RCFA | 169 | 1.4 |
| 774 | 1REWC | 86 | 1.86 |
| 775 | 1RG8A | 141 | 1.1 |
| 776 | 1RH6B | 52 | 1.7 |
| 777 | 1RHFB | 176 | 1.96 |
| 778 | 1RHSA | 293 | 1.36 |
| 779 | 1RJUV | 36 | 1.44 |
| 780 | 1RK8C | 33 | 1.9 |
| 781 | 1RKIB | 97 | 1.6 |
| 782 | 1RLWA | 126 | 2.4 |
| 783 | 1RMDA | 116 | 2.1 |
| 784 | 1RO2A | 210 | 1.6 |
| 785 | 1ROCA | 155 | 1.5 |
| 786 | 1RREE | 191 | 1.75 |
| 787 | 1RSSA | 140 | 1.9 |
| 788 | 1RTQA | 291 | 0.95 |
| 789 | 1RTTA | 174 | 1.28 |
| 790 | 1RUTX | 157 | 1.3 |
| 791 | 1RW1A | 114 | 1.02 |
| 792 | 1RWJA | 81 | 1.7 |
| 793 | 1RY9D | 133 | 1.82 |
| 794 | 1RYLB | 149 | 1.6 |
| 795 | 1RYQA | 64 | 1.38 |
| 796 | 1RZ4A | 213 | 2.1 |
| 797 | 1S0PA | 176 | 1.4 |
| 798 | 1S29A | 92 | 1.6 |
| 799 | 1S35A | 211 | 2.4 |
| 800 | 1S3JB | 141 | 2.25 |
| 801 | 1S5DA | 218 | 1.75 |
| 802 | 1S5NA | 386 | 0.95 |
| 803 | 1S5PA | 225 | 1.96 |
| 804 | 1S5UH | 130 | 1.7 |
| 805 | 1S6CA | 167 | 2 |
| 806 | 1S7ZA | 106 | 1.83 |
| 807 | 1S99A | 186 | 1.65 |
| 808 | 1S9UA | 201 | 1.38 |
| 809 | 1SAUA | 114 | 1.12 |
| 810 | 1SBQB | 164 | 2.2 |
| 811 | 1SBYB | 254 | 1.1 |
| 812 | 1SC6A | 390 | 2.09 |
| 813 | 1SD4A | 122 | 2 |
| 814 | 1SDIA | 213 | 1.65 |
| 815 | 1SENA | 134 | 1.2 |
| 816 | 1SFSA | 213 | 1.07 |
| 817 | 1SFUB | 70 | 2 |
| 818 | 1SFXB | 104 | 1.55 |
| 819 | 1SG4C | 250 | 1.3 |
| 820 | 1SHXB | 138 | 2.1 |
| 821 | 1SJ1B | 66 | 1.5 |
| 822 | 1SJWA | 142 | 1.35 |
| 823 | 1SKNP | 74 | 2.5 |
| 824 | 1SLUA | 131 | 1.8 |
| 825 | 1SMBA | 149 | 1.55 |
| 826 | 1SMOB | 110 | 1.47 |
| 827 | 1SO2D | 365 | 2.4 |
| 828 | 1SO7A | 361 | 1.49 |
| 829 | 1SPBP | 71 | 2 |
| 830 | 1SQ9A | 378 | 1.9 |
| 831 | 1SQWA | 176 | 1.9 |
| 832 | 1SR4C | 154 | 2 |
| 833 | 1SRRA | 119 | 1.9 |
| 834 | 1ST9A | 137 | 1.5 |
| 835 | 1STMA | 141 | 1.9 |
| 836 | 1SU7A | 633 | 1.12 |
| 837 | 1SVFB | 38 | 1.4 |
| 838 | 1SVFC | 62 | 1.4 |
| 839 | 1SVIA | 182 | 1.95 |
| 840 | 1SVPB | 160 | 2 |
| 841 | 1SX5A | 244 | 1.5 |
| 842 | 1SYQB | 25 | 2.42 |
| 843 | 1SYXF | 62 | 2.35 |
| 844 | 1T0BH | 241 | 1.7 |
| 845 | 1T0HA | 96 | 1.97 |
| 846 | 1T0HB | 187 | 1.97 |
| 847 | 1T0IB | 177 | 2 |
| 848 | 1T15A | 211 | 1.85 |
| 849 | 1T1DA | 100 | 1.51 |
| 850 | 1T1JB | 125 | 1.7 |
| 851 | 1T1VA | 93 | 1.6 |
| 852 | 1T3UD | 91 | 2.5 |
| 853 | 1T3YA | 131 | 1.15 |
| 854 | 1T4OA | 81 | 2.5 |
| 855 | 1T4WA | 196 | 2.1 |
| 856 | 1T61A | 223 | 1.5 |
| 857 | 1T6FA | 37 | 1.47 |
| 858 | 1T6UA | 117 | 1.3 |
| 859 | 1T82D | 136 | 1.7 |
| 860 | 1T8KA | 77 | 1.1 |
| 861 | 1T92A | 108 | 1.6 |
| 862 | 1TAFA | 68 | 2 |
| 863 | 1TC1B | 186 | 1.41 |
| 864 | 1TC3C | 51 | 2.45 |
| 865 | 1TCAA | 317 | 1.55 |
| 866 | 1TG0A | 66 | 0.97 |
| 867 | 1TGXC | 60 | 1.55 |
| 868 | 1TH8A | 132 | 2.4 |
| 869 | 1TH8B | 115 | 2.4 |
| 870 | 1TIFA | 76 | 1.8 |
| 871 | 1TIGA | 88 | 2 |
| 872 | 1TKEA | 224 | 1.46 |
| 873 | 1TL2A | 235 | 2 |
| 874 | 1TO2I | 63 | 1.3 |
| 875 | 1TOJA | 396 | 1.9 |
| 876 | 1TP6A | 126 | 1.5 |
| 877 | 1TQ5A | 234 | 1.76 |
| 878 | 1TQ8B | 127 | 2.4 |
| 879 | 1TQGA | 105 | 0.98 |
| 880 | 1TQHA | 242 | 1.63 |
| 881 | 1TQJC | 218 | 1.6 |
| 882 | 1TS9A | 98 | 1.7 |
| 883 | 1TT8A | 164 | 1 |
| 884 | 1TU7A | 208 | 1.5 |
| 885 | 1TUHA | 131 | 1.85 |
| 886 | 1TUKA | 67 | 1.12 |
| 887 | 1TULA | 102 | 2.2 |
| 888 | 1TVDA | 116 | 1.9 |
| 889 | 1TVGA | 136 | 1.6 |
| 890 | 1TVXD | 71 | 1.75 |
| 891 | 1TWFF | 84 | 2.3 |
| 892 | 1TWFI | 122 | 2.3 |
| 893 | 1TWFL | 46 | 2.3 |
| 894 | 1TXJA | 157 | 2 |
| 895 | 1TXNA | 259 | 1.7 |
| 896 | 1TXUA | 254 | 2.35 |
| 897 | 1TY0B | 207 | 1.75 |
| 898 | 1TZDB | 242 | 2.2 |
| 899 | 1U02A | 229 | 1.92 |
| 900 | 1U07A | 90 | 1.13 |
| 901 | 1U0AD | 214 | 1.64 |
| 902 | 1U2MC | 143 | 2.3 |
| 903 | 1U4GA | 298 | 1.4 |
| 904 | 1U58A | 222 | 1.9 |
| 905 | 1U5FA | 119 | 1.9 |
| 906 | 1U5KA | 242 | 2 |
| 907 | 1U69D | 147 | 1.6 |
| 908 | 1U7GA | 383 | 1.4 |
| 909 | 1U7IA | 134 | 1.4 |
| 910 | 1U7LA | 364 | 1.75 |
| 911 | 1U83A | 225 | 2.2 |
| 912 | 1U9DB | 122 | 1.7 |
| 913 | 1UADD | 92 | 2.1 |
| 914 | 1UAIA | 223 | 1.2 |
| 915 | 1UASA | 362 | 1.5 |
| 916 | 1UCDA | 190 | 1.3 |
| 917 | 1UCSA | 64 | 0.62 |
| 918 | 1UF5B | 303 | 1.6 |
| 919 | 1UFOD | 238 | 1.6 |
| 920 | 1UFYA | 121 | 0.96 |
| 921 | 1UG6A | 426 | 0.99 |
| 922 | 1UGIB | 83 | 1.55 |
| 923 | 1UI0A | 192 | 1.5 |
| 924 | 1UIXA | 69 | 1.8 |
| 925 | 1UJ8A | 73 | 1.75 |
| 926 | 1UNQA | 117 | 0.98 |
| 927 | 1UOCA | 263 | 2.3 |
| 928 | 1UOWA | 156 | 1.04 |
| 929 | 1UOYA | 64 | 1.5 |
| 930 | 1URQC | 68 | 2 |
| 931 | 1URRA | 97 | 1.5 |
| 932 | 1URSA | 366 | 1.45 |
| 933 | 1US0A | 313 | 0.66 |
| 934 | 1USCB | 178 | 1.24 |
| 935 | 1USEA | 40 | 1.3 |
| 936 | 1USMA | 77 | 1.2 |
| 937 | 1UTGA | 70 | 1.34 |
| 938 | 1UUQA | 410 | 1.5 |
| 939 | 1UUZA | 129 | 1.8 |
| 940 | 1UV7B | 74 | 1.7 |
| 941 | 1UVQB | 181 | 1.8 |
| 942 | 1UWCA | 261 | 1.08 |
| 943 | 1UWWB | 179 | 1.4 |
| 944 | 1UX6A | 335 | 1.9 |
| 945 | 1UZ3A | 102 | 1.1 |
| 946 | 1UZKA | 152 | 1.35 |
| 947 | 1V05A | 96 | 1.43 |
| 948 | 1V2ZA | 106 | 1.8 |
| 949 | 1V4PA | 151 | 1.45 |
| 950 | 1V5IB | 76 | 1.5 |
| 951 | 1V6PA | 62 | 0.87 |
| 952 | 1V76B | 91 | 2 |
| 953 | 1V7PC | 193 | 1.9 |
| 954 | 1V8DC | 188 | 2.16 |
| 955 | 1V96A | 146 | 1.75 |
| 956 | 1V9YB | 103 | 1.32 |
| 957 | 1VBWA | 68 | 0.93 |
| 958 | 1VCCA | 77 | 1.6 |
| 959 | 1VCLA | 432 | 1.7 |
| 960 | 1VF6D | 48 | 2.1 |
| 961 | 1VGWE | 213 | 2.35 |
| 962 | 1VH5A | 138 | 1.34 |
| 963 | 1VH6A | 102 | 2.5 |
| 964 | 1VHFA | 101 | 1.54 |
| 965 | 1VHTC | 189 | 1.59 |
| 966 | 1VHUA | 192 | 1.34 |
| 967 | 1VI4A | 162 | 1.87 |
| 968 | 1VJNA | 194 | 2 |
| 969 | 1VJQB | 73 | 2.1 |
| 970 | 1VK1A | 232 | 1.2 |
| 971 | 1VKED | 101 | 1.56 |
| 972 | 1VKKA | 137 | 1.35 |
| 973 | 1VL0A | 281 | 2.05 |
| 974 | 1VL7A | 135 | 1.5 |
| 975 | 1VMGA | 83 | 1.46 |
| 976 | 1VMHA | 129 | 1.31 |
| 977 | 1VPMC | 154 | 1.66 |
| 978 | 1VQ3D | 84 | 1.9 |
| 979 | 1VQO1 | 56 | 2.2 |
| 980 | 1VQO2 | 46 | 2.2 |
| 981 | 1VQO3 | 92 | 2.2 |
| 982 | 1VQOG | 29 | 2.2 |
| 983 | 1VQOK | 132 | 2.2 |
| 984 | 1VQOO | 115 | 2.2 |
| 985 | 1VQOP | 143 | 2.2 |
| 986 | 1VQOS | 81 | 2.2 |
| 987 | 1VQOZ | 73 | 2.2 |
| 988 | 1VQSD | 110 | 1.5 |
| 989 | 1VR7A | 120 | 1.2 |
| 990 | 1VR9B | 121 | 1.7 |
| 991 | 1VYIA | 111 | 1.5 |
| 992 | 1VYKA | 129 | 1.49 |
| 993 | 1VYRA | 363 | 0.9 |
| 994 | 1VZIA | 125 | 1.15 |
| 995 | 1VZJH | 32 | 2.35 |
| 996 | 1W0HA | 200 | 1.59 |
| 997 | 1W0NA | 120 | 0.8 |
| 998 | 1W0UB | 55 | 1.8 |
| 999 | 1W1DA | 143 | 1.5 |
| 1000 | 1W1OA | 479 | 1.7 |
| 1001 | 1W23A | 360 | 1.08 |
| 1002 | 1W2LA | 97 | 1.3 |
| 1003 | 1W2WJ | 191 | 1.75 |
| 1004 | 1W4SA | 146 | 1.55 |
| 1005 | 1W5QA | 320 | 1.4 |
| 1006 | 1W66A | 218 | 1.08 |
| 1007 | 1W6SB | 72 | 1.2 |
| 1008 | 1W70A | 60 | 1.46 |
| 1009 | 1W7CA | 737 | 1.23 |
| 1010 | 1W94A | 155 | 2 |
| 1011 | 1W98B | 270 | 2.15 |
| 1012 | 1W9AA | 142 | 1.8 |
| 1013 | 1W9HA | 398 | 1.95 |
| 1014 | 1W9MA | 553 | 1.35 |
| 1015 | 1WC2A | 180 | 1.2 |
| 1016 | 1WDCA | 64 | 2 |
| 1017 | 1WDGB | 74 | 2.06 |
| 1018 | 1WHZA | 70 | 1.52 |
| 1019 | 1WK2A | 84 | 2.5 |
| 1020 | 1WKOA | 165 | 1.8 |
| 1021 | 1WKQB | 155 | 1.17 |
| 1022 | 1WL8A | 187 | 1.45 |
| 1023 | 1WLZA | 85 | 1.6 |
| 1024 | 1WM3A | 72 | 1.2 |
| 1025 | 1WMAA | 275 | 1.24 |
| 1026 | 1WMGF | 87 | 2.1 |
| 1027 | 1WMIA | 88 | 2.3 |
| 1028 | 1WMZD | 140 | 1.7 |
| 1029 | 1WOCD | 99 | 2 |
| 1030 | 1WOUA | 119 | 1.8 |
| 1031 | 1WP5A | 323 | 1.79 |
| 1032 | 1WPAA | 107 | 1.5 |
| 1033 | 1WPUB | 147 | 1.48 |
| 1034 | 1WQ6A | 59 | 2 |
| 1035 | 1WQJB | 80 | 1.6 |
| 1036 | 1WRIA | 93 | 1.2 |
| 1037 | 1WT6D | 63 | 1.6 |
| 1038 | 1WU3I | 161 | 2.15 |
| 1039 | 1WUIL | 534 | 1.04 |
| 1040 | 1WUIS | 267 | 1.04 |
| 1041 | 1WV9A | 87 | 2 |
| 1042 | 1WW7A | 160 | 1.9 |
| 1043 | 1WWCA | 105 | 1.9 |
| 1044 | 1WWIA | 148 | 1.58 |
| 1045 | 1WWZB | 157 | 1.75 |
| 1046 | 1WY3A | 35 | 0.95 |
| 1047 | 1WZ3B | 84 | 1.8 |
| 1048 | 1WZDA | 209 | 1.35 |
| 1049 | 1X0GD | 104 | 2.5 |
| 1050 | 1X2IA | 68 | 1.45 |
| 1051 | 1X3KA | 152 | 1.64 |
| 1052 | 1X46A | 150 | 1.5 |
| 1053 | 1X6IB | 87 | 1.2 |
| 1054 | 1X6OA | 143 | 1.6 |
| 1055 | 1X6ZA | 119 | 0.78 |
| 1056 | 1X82A | 190 | 1.5 |
| 1057 | 1X8DD | 104 | 1.8 |
| 1058 | 1X8QA | 184 | 0.85 |
| 1059 | 1X91A | 149 | 1.5 |
| 1060 | 1X9DA | 451 | 1.41 |
| 1061 | 1XAUA | 104 | 1.8 |
| 1062 | 1XDNA | 265 | 1.2 |
| 1063 | 1XE0A | 108 | 1.7 |
| 1064 | 1XEBF | 145 | 2.35 |
| 1065 | 1XEDC | 105 | 1.9 |
| 1066 | 1XERA | 103 | 2 |
| 1067 | 1XG0B | 67 | 0.97 |
| 1068 | 1XG0C | 174 | 0.97 |
| 1069 | 1XG5C | 257 | 1.53 |
| 1070 | 1XG8A | 108 | 2.1 |
| 1071 | 1XIWF | 63 | 1.9 |
| 1072 | 1XIYA | 172 | 1.8 |
| 1073 | 1XJUA | 156 | 1.07 |
| 1074 | 1XK4A | 87 | 1.8 |
| 1075 | 1XKPC | 126 | 1.7 |
| 1076 | 1XL3A | 202 | 2.2 |
| 1077 | 1XLQC | 106 | 1.45 |
| 1078 | 1XM8B | 254 | 1.74 |
| 1079 | 1XMKA | 79 | 0.97 |
| 1080 | 1XMTA | 95 | 1.15 |
| 1081 | 1XODA | 105 | 1.15 |
| 1082 | 1XPJD | 123 | 2.3 |
| 1083 | 1XPPD | 101 | 1.6 |
| 1084 | 1XQAB | 111 | 1.8 |
| 1085 | 1XQOA | 253 | 1.03 |
| 1086 | 1XRXD | 35 | 2.15 |
| 1087 | 1XS1B | 193 | 1.8 |
| 1088 | 1XSVA | 106 | 1.7 |
| 1089 | 1XT5A | 135 | 1.15 |
| 1090 | 1XU1D | 137 | 1.9 |
| 1091 | 1XU1T | 39 | 1.9 |
| 1092 | 1XVSA | 124 | 2.01 |
| 1093 | 1XVXA | 311 | 1.53 |
| 1094 | 1XWTA | 404 | 1.3 |
| 1095 | 1Y0HA | 101 | 1.6 |
| 1096 | 1Y0NA | 71 | 2 |
| 1097 | 1Y2IE | 109 | 2.3 |
| 1098 | 1Y42X | 371 | 1.95 |
| 1099 | 1Y43B | 171 | 1.4 |
| 1100 | 1Y55X | 120 | 1 |
| 1101 | 1Y5MB | 265 | 2.3 |
| 1102 | 1Y62D | 56 | 2.45 |
| 1103 | 1Y63A | 168 | 1.7 |
| 1104 | 1Y66C | 44 | 1.65 |
| 1105 | 1Y71B | 112 | 1.95 |
| 1106 | 1Y7RA | 133 | 1.7 |
| 1107 | 1Y7TB | 327 | 1.65 |
| 1108 | 1Y7YB | 66 | 1.69 |
| 1109 | 1Y8XB | 92 | 2.4 |
| 1110 | 1Y93A | 158 | 1.03 |
| 1111 | 1YA5T | 89 | 2.44 |
| 1112 | 1YB0A | 157 | 1.86 |
| 1113 | 1YBKB | 52 | 1.45 |
| 1114 | 1YBXB | 92 | 1.8 |
| 1115 | 1YCCA | 108 | 1.23 |
| 1116 | 1YD3A | 90 | 1.6 |
| 1117 | 1YD7A | 169 | 2.3 |
| 1118 | 1YDLA | 71 | 2.3 |
| 1119 | 1YDXA | 374 | 2.3 |
| 1120 | 1YFND | 107 | 1.8 |
| 1121 | 1YFQA | 342 | 1.1 |
| 1122 | 1YFUA | 174 | 1.9 |
| 1123 | 1YG9A | 330 | 1.3 |
| 1124 | 1YISA | 421 | 2.4 |
| 1125 | 1YJ7A | 154 | 1.8 |
| 1126 | 1YK4A | 52 | 0.69 |
| 1127 | 1YKSA | 431 | 1.8 |
| 1128 | 1YKWA | 414 | 2 |
| 1129 | 1YLFA | 117 | 2.5 |
| 1130 | 1YM0B | 27 | 2.06 |
| 1131 | 1YMTA | 235 | 1.2 |
| 1132 | 1YN9A | 168 | 1.5 |
| 1133 | 1YODB | 27 | 1.8 |
| 1134 | 1YOZA | 113 | 2 |
| 1135 | 1YPFA | 295 | 1.8 |
| 1136 | 1YPHC | 131 | 1.34 |
| 1137 | 1YQHA | 104 | 1.7 |
| 1138 | 1YRBA | 259 | 1.75 |
| 1139 | 1YREA | 183 | 2.15 |
| 1140 | 1YSQA | 181 | 1.75 |
| 1141 | 1YU0A | 376 | 1.56 |
| 1142 | 1YUZB | 202 | 1.4 |
| 1143 | 1YW5A | 177 | 1.6 |
| 1144 | 1YYMS | 26 | 2.2 |
| 1145 | 1Z0PA | 73 | 1.7 |
| 1146 | 1Z27A | 175 | 2.08 |
| 1147 | 1Z2UA | 150 | 1.1 |
| 1148 | 1Z3EB | 67 | 1.5 |
| 1149 | 1Z4RA | 163 | 1.74 |
| 1150 | 1Z4VA | 444 | 2.3 |
| 1151 | 1Z6MA | 175 | 1.3 |
| 1152 | 1Z6OA | 212 | 1.91 |
| 1153 | 1Z7CA | 161 | 2 |
| 1154 | 1Z7KB | 62 | 1.9 |
| 1155 | 1Z8HB | 199 | 2.02 |
| 1156 | 1Z91A | 137 | 2.5 |
| 1157 | 1Z96A | 38 | 1.8 |
| 1158 | 1Z9FA | 89 | 2.3 |
| 1159 | 1Z9MB | 105 | 2.4 |
| 1160 | 1Z9TA | 240 | 1.54 |
| 1161 | 1Z9WA | 108 | 2.5 |
| 1162 | 1ZB1B | 367 | 1.95 |
| 1163 | 1ZBA3 | 221 | 2 |
| 1164 | 1ZBFA | 132 | 1.5 |
| 1165 | 1ZCBA | 318 | 2 |
| 1166 | 1ZCEA | 146 | 1.3 |
| 1167 | 1ZD8A | 212 | 1.48 |
| 1168 | 1ZEEA | 363 | 2.31 |
| 1169 | 1ZGKA | 288 | 1.35 |
| 1170 | 1ZGZA | 121 | 1.8 |
| 1171 | 1ZHVA | 134 | 1.5 |
| 1172 | 1ZJRA | 197 | 1.85 |
| 1173 | 1ZJZA | 251 | 1.1 |
| 1174 | 1ZL0A | 306 | 1.1 |
| 1175 | 1ZLDA | 103 | 1.65 |
| 1176 | 1ZLHB | 74 | 1.7 |
| 1177 | 1ZMAA | 118 | 1.25 |
| 1178 | 1ZMED | 70 | 2.5 |
| 1179 | 1ZMIA | 29 | 1.15 |
| 1180 | 1ZMMA | 31 | 1.6 |
| 1181 | 1ZMTA | 252 | 1.7 |
| 1182 | 1ZOQC | 47 | 2.37 |
| 1183 | 1ZPSB | 128 | 1.7 |
| 1184 | 1ZPVC | 88 | 1.9 |
| 1185 | 1ZR3A | 186 | 1.66 |
| 1186 | 1ZRHA | 263 | 2.1 |
| 1187 | 1ZS4D | 73 | 1.7 |
| 1188 | 1ZUUA | 56 | 0.97 |
| 1189 | 1ZUYA | 58 | 1.39 |
| 1190 | 1ZVAA | 75 | 1.5 |
| 1191 | 1ZVDA | 373 | 2.1 |
| 1192 | 1ZVTA | 245 | 1.7 |
| 1193 | 1ZW0H | 66 | 1.8 |
| 1194 | 1ZWXA | 288 | 1.9 |
| 1195 | 1ZWYA | 165 | 1.9 |
| 1196 | 1ZX3A | 86 | 2.5 |
| 1197 | 1ZZKA | 80 | 0.95 |
| 1198 | 1ZZWA | 147 | 1.6 |
| 1199 | 256BA | 106 | 1.4 |
| 1200 | 2A06H | 66 | 2.1 |
| 1201 | 2A0BA | 118 | 1.57 |
| 1202 | 2A0JA | 146 | 2.5 |
| 1203 | 2A0MA | 298 | 1.6 |
| 1204 | 2A1LA | 269 | 2.18 |
| 1205 | 2A25A | 146 | 2.2 |
| 1206 | 2A26A | 48 | 1.2 |
| 1207 | 2A2KA | 171 | 1.52 |
| 1208 | 2A40B | 260 | 1.8 |
| 1209 | 2A5ZC | 244 | 2.02 |
| 1210 | 2A6CA | 76 | 1.9 |
| 1211 | 2A6QD | 58 | 2.05 |
| 1212 | 2A6SD | 83 | 1.77 |
| 1213 | 2A6ZA | 222 | 1 |
| 1214 | 2A7KA | 229 | 2.24 |
| 1215 | 2AA3A | 316 | 2.05 |
| 1216 | 2ABKA | 211 | 1.85 |
| 1217 | 2ABSA | 340 | 1.1 |
| 1218 | 2ABWA | 216 | 1.62 |
| 1219 | 2ACAB | 174 | 2.25 |
| 1220 | 2ACFD | 179 | 1.4 |
| 1221 | 2AD7A | 571 | 1.5 |
| 1222 | 2ADVB | 28 | 2.24 |
| 1223 | 2AEBB | 309 | 1.29 |
| 1224 | 2AENA | 164 | 1.6 |
| 1225 | 2AG4B | 164 | 1.8 |
| 1226 | 2AGKA | 233 | 1.3 |
| 1227 | 2AH5A | 210 | 1.74 |
| 1228 | 2AIBA | 98 | 1.1 |
| 1229 | 2AJ6A | 120 | 1.63 |
| 1230 | 2AJ7B | 148 | 1.67 |
| 1231 | 2AKFC | 32 | 1.2 |
| 1232 | 2AMLB | 361 | 1.5 |
| 1233 | 2ANUA | 224 | 2.4 |
| 1234 | 2ANXA | 146 | 1.04 |
| 1235 | 2AO9F | 110 | 1.9 |
| 1236 | 2APLA | 149 | 2.01 |
| 1237 | 2APOB | 55 | 1.95 |
| 1238 | 2AQMA | 154 | 1.1 |
| 1239 | 2AR5A | 117 | 1.8 |
| 1240 | 2ARCA | 161 | 1.5 |
| 1241 | 2ARPA | 105 | 2 |
| 1242 | 2AS9A | 207 | 1.7 |
| 1243 | 2ASKB | 101 | 1.55 |
| 1244 | 2ATMA | 324 | 2 |
| 1245 | 2ATPD | 112 | 2.4 |
| 1246 | 2ATRA | 132 | 2.01 |
| 1247 | 2AUAB | 210 | 2.35 |
| 1248 | 2AUWA | 151 | 1.85 |
| 1249 | 2AXIA | 92 | 1.4 |
| 1250 | 2AXWB | 134 | 1.05 |
| 1251 | 2AYDA | 76 | 1.6 |
| 1252 | 2AZWA | 146 | 1.9 |
| 1253 | 2B06A | 150 | 1.4 |
| 1254 | 2B0AA | 186 | 1.45 |
| 1255 | 2B18A | 155 | 1.8 |
| 1256 | 2B1YA | 101 | 1.8 |
| 1257 | 2B4AA | 116 | 2.42 |
| 1258 | 2B5GA | 164 | 1.7 |
| 1259 | 2B5IB | 196 | 2.3 |
| 1260 | 2B69A | 312 | 1.21 |
| 1261 | 2B7JC | 158 | 2.3 |
| 1262 | 2B7UA | 251 | 1.6 |
| 1263 | 2B82A | 211 | 1.25 |
| 1264 | 2B8MA | 109 | 1.7 |
| 1265 | 2B97A | 70 | 0.75 |
| 1266 | 2B9DB | 52 | 1.6 |
| 1267 | 2B9SB | 52 | 2.27 |
| 1268 | 2BBAA | 185 | 1.65 |
| 1269 | 2BBRA | 189 | 1.2 |
| 1270 | 2BCXB | 27 | 2 |
| 1271 | 2BDQB | 210 | 2.3 |
| 1272 | 2BE6D | 29 | 2 |
| 1273 | 2BF6A | 448 | 0.97 |
| 1274 | 2BF9A | 36 | 0.99 |
| 1275 | 2BFFA | 392 | 1.46 |
| 1276 | 2BH1X | 68 | 2.4 |
| 1277 | 2BH4X | 121 | 1.55 |
| 1278 | 2BHUA | 580 | 1.1 |
| 1279 | 2BI7A | 383 | 2 |
| 1280 | 2BJ7B | 138 | 2.1 |
| 1281 | 2BJDB | 90 | 1.27 |
| 1282 | 2BJIB | 274 | 1.3 |
| 1283 | 2BJNB | 148 | 1.7 |
| 1284 | 2BK8A | 97 | 1.69 |
| 1285 | 2BK9A | 153 | 1.2 |
| 1286 | 2BKFA | 86 | 1.56 |
| 1287 | 2BKMB | 128 | 1.5 |
| 1288 | 2BKRA | 211 | 1.9 |
| 1289 | 2BKYY | 86 | 1.7 |
| 1290 | 2BL0A | 63 | 1.75 |
| 1291 | 2BL8B | 81 | 1.6 |
| 1292 | 2BLAA | 81 | 2.5 |
| 1293 | 2BLFB | 81 | 1.8 |
| 1294 | 2BNLF | 129 | 2 |
| 1295 | 2BO9B | 217 | 1.6 |
| 1296 | 2BOPA | 85 | 1.7 |
| 1297 | 2BOUA | 137 | 1.9 |
| 1298 | 2BQ4A | 114 | 1.68 |
| 1299 | 2BS2F | 254 | 1.78 |
| 1300 | 2BSYA | 249 | 1.5 |
| 1301 | 2BT6B | 104 | 1.5 |
| 1302 | 2BT9C | 88 | 0.94 |
| 1303 | 2BTIA | 58 | 2 |
| 1304 | 2BU3B | 204 | 1.4 |
| 1305 | 2BUEA | 179 | 1.7 |
| 1306 | 2BV2A | 83 | 1.55 |
| 1307 | 2BW3B | 84 | 2 |
| 1308 | 2BW4A | 334 | 0.9 |
| 1309 | 2BWQA | 122 | 1.41 |
| 1310 | 2BZ6L | 53 | 1.6 |
| 1311 | 2BZVA | 146 | 1.15 |
| 1312 | 2BZWB | 27 | 2.3 |
| 1313 | 2BZYB | 62 | 2.5 |
| 1314 | 2C0HA | 353 | 1.6 |
| 1315 | 2C1MB | 46 | 2.2 |
| 1316 | 2C29F | 326 | 1.81 |
| 1317 | 2C2JA | 165 | 2.05 |
| 1318 | 2C2UA | 178 | 1.1 |
| 1319 | 2C3AA | 252 | 2.5 |
| 1320 | 2C3HH | 92 | 2.24 |
| 1321 | 2C3VB | 94 | 1.39 |
| 1322 | 2C5KT | 89 | 2.05 |
| 1323 | 2C5SA | 372 | 2.5 |
| 1324 | 2C60A | 85 | 1.25 |
| 1325 | 2C6UA | 122 | 1.6 |
| 1326 | 2C71A | 205 | 1.05 |
| 1327 | 2C7NE | 48 | 2.1 |
| 1328 | 2C9LZ | 62 | 2.25 |
| 1329 | 2CA5A | 62 | 2.1 |
| 1330 | 2CAKA | 154 | 1.27 |
| 1331 | 2CARB | 194 | 1.09 |
| 1332 | 2CB8A | 86 | 1.4 |
| 1333 | 2CB9A | 212 | 1.8 |
| 1334 | 2CBPA | 96 | 1.8 |
| 1335 | 2CCVA | 99 | 1.3 |
| 1336 | 2CDOC | 136 | 1.64 |
| 1337 | 2CE0A | 99 | 1.24 |
| 1338 | 2CF5A | 352 | 2 |
| 1339 | 2CG7A | 90 | 1.2 |
| 1340 | 2CHHA | 113 | 1 |
| 1341 | 2CHPA | 148 | 2 |
| 1342 | 2CI1A | 275 | 1.08 |
| 1343 | 2CISA | 288 | 1.62 |
| 1344 | 2CJSC | 54 | 1.78 |
| 1345 | 2CKKA | 120 | 1.45 |
| 1346 | 2CKWA | 487 | 2.3 |
| 1347 | 2CKXA | 83 | 1.9 |
| 1348 | 2CLBA | 169 | 2.4 |
| 1349 | 2CMPA | 56 | 1.58 |
| 1350 | 2CNQA | 301 | 1 |
| 1351 | 2CO3B | 135 | 1.78 |
| 1352 | 2COQA | 108 | 2.1 |
| 1353 | 2COVI | 88 | 1.25 |
| 1354 | 2CPGA | 43 | 1.6 |
| 1355 | 2CS7C | 54 | 1.2 |
| 1356 | 2CVDA | 198 | 1.45 |
| 1357 | 2CVIA | 83 | 1.5 |
| 1358 | 2CW9A | 182 | 1.9 |
| 1359 | 2CWRA | 97 | 1.7 |
| 1360 | 2CWSA | 227 | 1 |
| 1361 | 2CWZD | 137 | 1.85 |
| 1362 | 2CX7A | 129 | 1.75 |
| 1363 | 2CXYA | 114 | 1.6 |
| 1364 | 2CYJA | 118 | 1.5 |
| 1365 | 2CZQA | 205 | 1.05 |
| 1366 | 2D0BA | 308 | 2.1 |
| 1367 | 2D0OD | 108 | 2 |
| 1368 | 2D1PF | 95 | 2.15 |
| 1369 | 2D1SA | 539 | 1.3 |
| 1370 | 2D37A | 155 | 1.7 |
| 1371 | 2D39B | 200 | 1.9 |
| 1372 | 2D3DA | 83 | 1.6 |
| 1373 | 2D42B | 249 | 2.07 |
| 1374 | 2D48A | 129 | 1.65 |
| 1375 | 2D5VB | 135 | 2 |
| 1376 | 2D7CD | 42 | 1.75 |
| 1377 | 2D7DB | 38 | 2.1 |
| 1378 | 2D7VA | 155 | 1.97 |
| 1379 | 2DDXA | 324 | 0.86 |
| 1380 | 2DFBA | 190 | 1.11 |
| 1381 | 2DJFA | 118 | 2 |
| 1382 | 2DKOA | 146 | 1.06 |
| 1383 | 2DKOB | 103 | 1.06 |
| 1384 | 2DLBA | 70 | 1.2 |
| 1385 | 2DQAB | 123 | 1.6 |
| 1386 | 2DRVA | 191 | 1.6 |
| 1387 | 2DS2C | 28 | 1.7 |
| 1388 | 2DS2D | 68 | 1.7 |
| 1389 | 2DS5A | 43 | 1.5 |
| 1390 | 2DTJB | 163 | 1.58 |
| 1391 | 2DVTC | 323 | 1.7 |
| 1392 | 2DWUC | 266 | 1.6 |
| 1393 | 2DY0B | 181 | 1.25 |
| 1394 | 2DYJB | 90 | 1.84 |
| 1395 | 2DYNA | 111 | 2.3 |
| 1396 | 2DYOB | 36 | 1.97 |
| 1397 | 2E0NA | 229 | 2 |
| 1398 | 2E10B | 222 | 1.35 |
| 1399 | 2E1FA | 94 | 2 |
| 1400 | 2E2RA | 227 | 1.6 |
| 1401 | 2E3HA | 81 | 1.45 |
| 1402 | 2E4TA | 509 | 0.96 |
| 1403 | 2E56A | 144 | 2 |
| 1404 | 2E6FA | 312 | 1.26 |
| 1405 | 2E6XA | 69 | 2 |
| 1406 | 2E8EA | 132 | 1.7 |
| 1407 | 2E9XA | 144 | 2.3 |
| 1408 | 2EA7A | 390 | 1.8 |
| 1409 | 2EB4B | 265 | 1.6 |
| 1410 | 2ECUA | 149 | 1.3 |
| 1411 | 2EEYA | 160 | 1.94 |
| 1412 | 2EFJA | 349 | 2 |
| 1413 | 2EGJB | 126 | 1.8 |
| 1414 | 2EHPB | 124 | 1.3 |
| 1415 | 2EHZA | 299 | 1.35 |
| 1416 | 2EIXA | 243 | 1.56 |
| 1417 | 2EJ8A | 133 | 1.84 |
| 1418 | 2ENDA | 137 | 1.45 |
| 1419 | 2EQ7C | 37 | 1.8 |
| 1420 | 2ERLA | 40 | 1 |
| 1421 | 2EUCA | 108 | 2.5 |
| 1422 | 2EV1B | 181 | 1.6 |
| 1423 | 2EWRA | 156 | 1.6 |
| 1424 | 2EWTA | 71 | 1.81 |
| 1425 | 2EX4B | 221 | 1.75 |
| 1426 | 2F0CA | 147 | 1.65 |
| 1427 | 2F15A | 89 | 2 |
| 1428 | 2F3CI | 46 | 2.5 |
| 1429 | 2F3LA | 132 | 2.11 |
| 1430 | 2F42A | 138 | 2.5 |
| 1431 | 2F4MB | 61 | 1.85 |
| 1432 | 2F5JA | 157 | 2.2 |
| 1433 | 2F5TX | 233 | 1.45 |
| 1434 | 2F5VA | 577 | 1.41 |
| 1435 | 2F69A | 244 | 1.3 |
| 1436 | 2F6EA | 125 | 1.85 |
| 1437 | 2F6MB | 107 | 2.1 |
| 1438 | 2F6UB | 231 | 1.55 |
| 1439 | 2F9HA | 121 | 1.57 |
| 1440 | 2FA5B | 142 | 1.8 |
| 1441 | 2FA8A | 86 | 1.9 |
| 1442 | 2FAUA | 288 | 2.1 |
| 1443 | 2FB0A | 94 | 2.1 |
| 1444 | 2FB5B | 204 | 1.99 |
| 1445 | 2FB6A | 116 | 1.46 |
| 1446 | 2FBIA | 136 | 2.1 |
| 1447 | 2FCWA | 106 | 1.26 |
| 1448 | 2FCWB | 78 | 1.26 |
| 1449 | 2FDNA | 55 | 0.94 |
| 1450 | 2FE5A | 94 | 1.1 |
| 1451 | 2FEAB | 224 | 2 |
| 1452 | 2FFGB | 79 | 2.31 |
| 1453 | 2FFUA | 494 | 1.64 |
| 1454 | 2FG1A | 157 | 1.25 |
| 1455 | 2FGCA | 161 | 2.3 |
| 1456 | 2FHPA | 183 | 1.6 |
| 1457 | 2FHZA | 106 | 1.15 |
| 1458 | 2FHZB | 93 | 1.15 |
| 1459 | 2FI0A | 79 | 2.1 |
| 1460 | 2FJ8A | 120 | 1.19 |
| 1461 | 2FJCP | 151 | 2.5 |
| 1462 | 2FKKA | 184 | 1.2 |
| 1463 | 2FL4A | 147 | 1.6 |
| 1464 | 2FL7A | 183 | 1.85 |
| 1465 | 2FLHB | 153 | 1.2 |
| 1466 | 2FM8A | 134 | 2.2 |
| 1467 | 2FMAA | 59 | 0.85 |
| 1468 | 2FN9A | 280 | 1.4 |
| 1469 | 2FNOB | 234 | 2 |
| 1470 | 2FOMB | 150 | 1.5 |
| 1471 | 2FP7A | 40 | 1.68 |
| 1472 | 2FP8B | 303 | 2.3 |
| 1473 | 2FPEA | 62 | 1.75 |
| 1474 | 2FPWB | 163 | 1.75 |
| 1475 | 2FQMA | 65 | 2.3 |
| 1476 | 2FQPD | 94 | 1.8 |
| 1477 | 2FSQA | 232 | 1.4 |
| 1478 | 2FTBA | 125 | 2 |
| 1479 | 2FTXB | 59 | 1.9 |
| 1480 | 2FU2A | 78 | 2.15 |
| 1481 | 2FU4A | 81 | 1.8 |
| 1482 | 2FUEA | 246 | 1.75 |
| 1483 | 2FUFA | 126 | 1.45 |
| 1484 | 2FURA | 193 | 1.8 |
| 1485 | 2FVVA | 135 | 1.25 |
| 1486 | 2FVYA | 305 | 0.92 |
| 1487 | 2FXAB | 180 | 2.4 |
| 1488 | 2FYGA | 128 | 1.8 |
| 1489 | 2FYUI | 57 | 2.26 |
| 1490 | 2FYUK | 53 | 2.26 |
| 1491 | 2FYZD | 31 | 2.2 |
| 1492 | 2FZVA | 235 | 1.7 |
| 1493 | 2G0CA | 68 | 1.7 |
| 1494 | 2G1UA | 137 | 1.5 |
| 1495 | 2G38D | 173 | 2.2 |
| 1496 | 2G3AA | 137 | 1.9 |
| 1497 | 2G3RA | 119 | 1.25 |
| 1498 | 2G3VA | 161 | 2.3 |
| 1499 | 2G5RA | 114 | 1.6 |
| 1500 | 2G6YB | 215 | 1.6 |
| 1501 | 2G7OA | 68 | 1.4 |
| 1502 | 2G7SA | 190 | 1.4 |
| 1503 | 2G9WB | 119 | 1.8 |
| 1504 | 2G9ZB | 295 | 1.96 |
| 1505 | 2GB4B | 230 | 1.25 |
| 1506 | 2GB7D | 292 | 1.7 |
| 1507 | 2GDQB | 373 | 1.8 |
| 1508 | 2GE7A | 107 | 2 |
| 1509 | 2GEFA | 198 | 2.2 |
| 1510 | 2GEYB | 133 | 1.8 |
| 1511 | 2GFFA | 97 | 1.75 |
| 1512 | 2GFQC | 278 | 1.75 |
| 1513 | 2GGCA | 263 | 1 |
| 1514 | 2GHCX | 249 | 1.25 |
| 1515 | 2GHVE | 183 | 2.2 |
| 1516 | 2GIAD | 146 | 1.89 |
| 1517 | 2GIAG | 155 | 1.89 |
| 1518 | 2GIBA | 97 | 1.75 |
| 1519 | 2GIXD | 205 | 2.02 |
| 1520 | 2GJ3B | 119 | 1.04 |
| 1521 | 2GJ4A | 803 | 1.6 |
| 1522 | 2GJVF | 136 | 2.39 |
| 1523 | 2GKGA | 122 | 1 |
| 1524 | 2GKMB | 126 | 1.73 |
| 1525 | 2GKPA | 164 | 1.35 |
| 1526 | 2GKTI | 51 | 1.23 |
| 1527 | 2GLZA | 149 | 1.45 |
| 1528 | 2GMWB | 184 | 1.5 |
| 1529 | 2GMYA | 147 | 1.6 |
| 1530 | 2GN4A | 329 | 1.9 |
| 1531 | 2GNCB | 55 | 1.8 |
| 1532 | 2GNPA | 262 | 1.65 |
| 1533 | 2GOMA | 61 | 1.25 |
| 1534 | 2GPED | 48 | 1.9 |
| 1535 | 2GPIA | 91 | 1.6 |
| 1536 | 2GQ0A | 256 | 1.9 |
| 1537 | 2GRCA | 121 | 1.5 |
| 1538 | 2GSCC | 110 | 2.45 |
| 1539 | 2GUKB | 112 | 1.91 |
| 1540 | 2GVGA | 461 | 2.2 |
| 1541 | 2GWMA | 200 | 1.5 |
| 1542 | 2GYQB | 157 | 1.4 |
| 1543 | 2GZ4A | 200 | 1.5 |
| 1544 | 2GZGA | 83 | 1.7 |
| 1545 | 2GZHB | 57 | 2.47 |
| 1546 | 2GZQA | 191 | 1.3 |
| 1547 | 2GZVA | 91 | 1.12 |
| 1548 | 2H00C | 203 | 2 |
| 1549 | 2H09A | 127 | 2.1 |
| 1550 | 2H0EB | 114 | 2.2 |
| 1551 | 2H28A | 109 | 2.1 |
| 1552 | 2H29A | 188 | 2 |
| 1553 | 2H3LB | 98 | 1 |
| 1554 | 2H5CA | 198 | 0.82 |
| 1555 | 2H5ND | 123 | 2.01 |
| 1556 | 2H7ZA | 75 | 1.5 |
| 1557 | 2H88C | 139 | 1.74 |
| 1558 | 2H8GB | 244 | 1.5 |
| 1559 | 2H98A | 216 | 1.8 |
| 1560 | 2H9DA | 84 | 1.95 |
| 1561 | 2H9EC | 52 | 2.2 |
| 1562 | 2HALA | 212 | 1.35 |
| 1563 | 2HBGA | 147 | 1.5 |
| 1564 | 2HC1A | 290 | 1.3 |
| 1565 | 2HCJA | 33 | 2.12 |
| 1566 | 2HCRB | 308 | 2.2 |
| 1567 | 2HD3K | 95 | 2.4 |
| 1568 | 2HD9A | 145 | 1.35 |
| 1569 | 2HDDA | 55 | 1.9 |
| 1570 | 2HDSB | 358 | 1.16 |
| 1571 | 2HDVA | 103 | 2 |
| 1572 | 2HEKB | 369 | 2 |
| 1573 | 2HEUA | 391 | 1.04 |
| 1574 | 2HEWF | 128 | 1.45 |
| 1575 | 2HEYR | 138 | 2 |
| 1576 | 2HF1A | 61 | 1.9 |
| 1577 | 2HFED | 43 | 2.25 |
| 1578 | 2HFNF | 139 | 1.8 |
| 1579 | 2HFTA | 205 | 1.69 |
| 1580 | 2HHGA | 132 | 1.2 |
| 1581 | 2HINA | 66 | 1.05 |
| 1582 | 2HIPB | 71 | 2.5 |
| 1583 | 2HJ1B | 80 | 2.1 |
| 1584 | 2HJ3A | 101 | 2.5 |
| 1585 | 2HJDA | 89 | 2.1 |
| 1586 | 2HKXB | 148 | 2.3 |
| 1587 | 2HL0A | 143 | 1.86 |
| 1588 | 2HLJA | 154 | 2 |
| 1589 | 2HLRA | 67 | 1.2 |
| 1590 | 2HNFA | 130 | 1.8 |
| 1591 | 2HNUE | 81 | 2 |
| 1592 | 2HO2A | 33 | 1.33 |
| 1593 | 2HPJA | 99 | 1.7 |
| 1594 | 2HQLF | 96 | 2 |
| 1595 | 2HQSE | 107 | 1.5 |
| 1596 | 2HQTH | 111 | 1.9 |
| 1597 | 2HSBA | 126 | 1.95 |
| 1598 | 2HTDB | 124 | 1.6 |
| 1599 | 2HTIA | 126 | 2.5 |
| 1600 | 2HTSA | 88 | 1.83 |
| 1601 | 2HUEB | 75 | 1.7 |
| 1602 | 2HUEC | 82 | 1.7 |
| 1603 | 2HUHA | 147 | 1.54 |
| 1604 | 2HUJA | 125 | 1.74 |
| 1605 | 2HVWC | 146 | 1.67 |
| 1606 | 2HW4A | 116 | 1.9 |
| 1607 | 2HWVA | 101 | 1.9 |
| 1608 | 2HX5A | 144 | 1.5 |
| 1609 | 2HY5C | 101 | 1.72 |
| 1610 | 2HY7A | 373 | 1.9 |
| 1611 | 2HYKA | 237 | 1.3 |
| 1612 | 2HZ5A | 87 | 2.1 |
| 1613 | 2HZYA | 416 | 1.35 |
| 1614 | 2I02A | 140 | 1.8 |
| 1615 | 2I04B | 85 | 2.15 |
| 1616 | 2I15A | 122 | 2.4 |
| 1617 | 2I3DA | 218 | 1.5 |
| 1618 | 2I3SF | 36 | 1.9 |
| 1619 | 2I45I | 97 | 2.5 |
| 1620 | 2I4AA | 107 | 1 |
| 1621 | 2I52D | 117 | 2.08 |
| 1622 | 2I53A | 254 | 1.5 |
| 1623 | 2I5FA | 99 | 1.35 |
| 1624 | 2I5HA | 178 | 1.74 |
| 1625 | 2I5IB | 261 | 1.7 |
| 1626 | 2I5UA | 77 | 1.5 |
| 1627 | 2I5VO | 246 | 1.1 |
| 1628 | 2I6CA | 160 | 1.3 |
| 1629 | 2I6JA | 161 | 1.66 |
| 1630 | 2I74A | 180 | 1.75 |
| 1631 | 2I7DA | 193 | 1.2 |
| 1632 | 2I7HD | 187 | 2.3 |
| 1633 | 2I8BA | 127 | 2 |
| 1634 | 2I8DA | 121 | 1.69 |
| 1635 | 2I9DC | 193 | 2.3 |
| 1636 | 2I9FD | 59 | 2 |
| 1637 | 2I9XA | 86 | 1.8 |
| 1638 | 2IA0A | 156 | 2.37 |
| 1639 | 2IA1A | 169 | 1.59 |
| 1640 | 2IA7A | 111 | 1.44 |
| 1641 | 2IAYA | 114 | 1.2 |
| 1642 | 2IBLA | 108 | 1.32 |
| 1643 | 2IBPB | 407 | 1.6 |
| 1644 | 2IC2B | 114 | 1.3 |
| 1645 | 2IC6B | 76 | 1.15 |
| 1646 | 2ICTA | 94 | 1.63 |
| 1647 | 2IDLB | 116 | 1.7 |
| 1648 | 2IDOD | 76 | 2.1 |
| 1649 | 2IECA | 113 | 2.33 |
| 1650 | 2IFTA | 176 | 2.3 |
| 1651 | 2IG6A | 143 | 1.8 |
| 1652 | 2IGIB | 179 | 1.7 |
| 1653 | 2II3A | 234 | 2.17 |
| 1654 | 2IIMA | 62 | 1 |
| 1655 | 2IIZA | 306 | 2.3 |
| 1656 | 2IJ2A | 450 | 1.2 |
| 1657 | 2IJLB | 109 | 2.3 |
| 1658 | 2ILKA | 155 | 1.6 |
| 1659 | 2ILNI | 53 | 2 |
| 1660 | 2IMFA | 203 | 1.3 |
| 1661 | 2IMJD | 153 | 1.5 |
| 1662 | 2IMQX | 280 | 1.3 |
| 1663 | 2IMSA | 163 | 1.48 |
| 1664 | 2IMZB | 140 | 1.7 |
| 1665 | 2IN5B | 188 | 2.3 |
| 1666 | 2IP1A | 371 | 1.8 |
| 1667 | 2IP6A | 87 | 1.35 |
| 1668 | 2IQYA | 190 | 1.4 |
| 1669 | 2ISBA | 184 | 1.66 |
| 1670 | 2IU1A | 178 | 1.8 |
| 1671 | 2IU5A | 179 | 1.6 |
| 1672 | 2IUWA | 205 | 1.5 |
| 1673 | 2IVYA | 88 | 1.4 |
| 1674 | 2IWOB | 99 | 1.7 |
| 1675 | 2IX7C | 58 | 2.5 |
| 1676 | 2IXDB | 230 | 1.8 |
| 1677 | 2IY2A | 69 | 1.9 |
| 1678 | 2IYBE | 64 | 2.35 |
| 1679 | 2IYVA | 179 | 1.35 |
| 1680 | 2IZXB | 41 | 1.3 |
| 1681 | 2J05A | 64 | 1.5 |
| 1682 | 2J0ST | 44 | 2.21 |
| 1683 | 2J2JD | 182 | 1.5 |
| 1684 | 2J3TC | 141 | 2.4 |
| 1685 | 2J3WB | 167 | 2.1 |
| 1686 | 2J43B | 218 | 1.6 |
| 1687 | 2J45B | 297 | 1.14 |
| 1688 | 2J4WD | 34 | 2.5 |
| 1689 | 2J5IA | 247 | 1.8 |
| 1690 | 2J67A | 141 | 2.2 |
| 1691 | 2J6BA | 109 | 1.3 |
| 1692 | 2J6ZA | 86 | 1.95 |
| 1693 | 2J73A | 103 | 1.4 |
| 1694 | 2J8BA | 78 | 1.15 |
| 1695 | 2J8CH | 240 | 1.87 |
| 1696 | 2J8CL | 281 | 1.87 |
| 1697 | 2J8KA | 181 | 1.5 |
| 1698 | 2J8QB | 195 | 2.3 |
| 1699 | 2J97A | 98 | 1.75 |
| 1700 | 2J9OD | 275 | 1.5 |
| 1701 | 2J9UB | 47 | 2 |
| 1702 | 2J9WA | 101 | 1.3 |
| 1703 | 2JAEA | 478 | 1.25 |
| 1704 | 2JBAA | 125 | 1.45 |
| 1705 | 2JC9A | 467 | 1.5 |
| 1706 | 2JCQA | 149 | 1.25 |
| 1707 | 2JDAA | 139 | 1.35 |
| 1708 | 2JDII | 25 | 1.9 |
| 1709 | 2JDQE | 65 | 2.2 |
| 1710 | 2JE6I | 207 | 1.6 |
| 1711 | 2JENA | 233 | 1.4 |
| 1712 | 2JERH | 365 | 1.65 |
| 1713 | 2JFRA | 234 | 0.83 |
| 1714 | 2JGBA | 187 | 1.7 |
| 1715 | 2JHFA | 374 | 1 |
| 1716 | 2JILA | 95 | 1.5 |
| 1717 | 2JJSC | 116 | 1.85 |
| 1718 | 2JJUA | 107 | 1.19 |
| 1719 | 2JK1A | 138 | 2.1 |
| 1720 | 2JKSA | 284 | 1.9 |
| 1721 | 2JKUA | 35 | 1.5 |
| 1722 | 2JL1A | 287 | 1.96 |
| 1723 | 2LISA | 131 | 1.35 |
| 1724 | 2MCMA | 112 | 1.5 |
| 1725 | 2MHRA | 118 | 1.3 |
| 1726 | 2MLTB | 26 | 2 |
| 1727 | 2MSBB | 113 | 1.7 |
| 1728 | 2NL9A | 142 | 1.55 |
| 1729 | 2NLRA | 222 | 1.2 |
| 1730 | 2NLSA | 36 | 0.98 |
| 1731 | 2NLVA | 112 | 1.3 |
| 1732 | 2NMLA | 100 | 1.55 |
| 1733 | 2NN4A | 62 | 2.1 |
| 1734 | 2NN5A | 171 | 1.45 |
| 1735 | 2NNUA | 201 | 1.59 |
| 1736 | 2NOGA | 157 | 2 |
| 1737 | 2NPNA | 238 | 1.6 |
| 1738 | 2NPSD | 63 | 2.5 |
| 1739 | 2NPTA | 102 | 1.75 |
| 1740 | 2NQDA | 109 | 1.75 |
| 1741 | 2NQWA | 87 | 1.3 |
| 1742 | 2NR5F | 57 | 1.9 |
| 1743 | 2NR7A | 194 | 1.3 |
| 1744 | 2NRKA | 165 | 1.65 |
| 1745 | 2NRRA | 139 | 1.2 |
| 1746 | 2NS6A | 183 | 2.1 |
| 1747 | 2NSQA | 140 | 1.85 |
| 1748 | 2NSZA | 129 | 1.15 |
| 1749 | 2NT0D | 497 | 1.79 |
| 1750 | 2NTXA | 314 | 2.2 |
| 1751 | 2NUHA | 104 | 1.39 |
| 1752 | 2NUTC | 135 | 2.3 |
| 1753 | 2NVNA | 121 | 2.5 |
| 1754 | 2NW0A | 189 | 1.6 |
| 1755 | 2NW2A | 196 | 1.4 |
| 1756 | 2NWFA | 141 | 1.1 |
| 1757 | 2NX4A | 194 | 1.7 |
| 1758 | 2NXVB | 249 | 1.1 |
| 1759 | 2NXYB | 181 | 2 |
| 1760 | 2NZCA | 80 | 1.95 |
| 1761 | 2O16A | 132 | 1.9 |
| 1762 | 2O1CD | 142 | 1.8 |
| 1763 | 2O1KA | 43 | 1.67 |
| 1764 | 2O1MB | 224 | 2 |
| 1765 | 2O5HB | 118 | 1.9 |
| 1766 | 2O6PB | 119 | 1.5 |
| 1767 | 2O71A | 91 | 2 |
| 1768 | 2O7AA | 116 | 0.84 |
| 1769 | 2O7TA | 188 | 2.1 |
| 1770 | 2O8GJ | 58 | 2.5 |
| 1771 | 2O90A | 115 | 1.07 |
| 1772 | 2O9AA | 182 | 1.8 |
| 1773 | 2O9SA | 67 | 0.83 |
| 1774 | 2O9UX | 96 | 1.15 |
| 1775 | 2OA5A | 92 | 2.1 |
| 1776 | 2OAAA | 247 | 1.5 |
| 1777 | 2OAFB | 144 | 2 |
| 1778 | 2OB0C | 166 | 1.8 |
| 1779 | 2OB3B | 330 | 1.04 |
| 1780 | 2OB5A | 151 | 1.6 |
| 1781 | 2OC5A | 222 | 1.68 |
| 1782 | 2OCTA | 97 | 1.4 |
| 1783 | 2OCZA | 218 | 1.85 |
| 1784 | 2OD4A | 101 | 1.7 |
| 1785 | 2OD6D | 108 | 1.85 |
| 1786 | 2ODBB | 35 | 2.4 |
| 1787 | 2ODIA | 238 | 1.45 |
| 1788 | 2ODKA | 51 | 1.4 |
| 1789 | 2ODMA | 79 | 2.24 |
| 1790 | 2OEEB | 93 | 1.96 |
| 1791 | 2OEGA | 482 | 2.3 |
| 1792 | 2OFCA | 141 | 1.11 |
| 1793 | 2OFWC | 204 | 2.05 |
| 1794 | 2OFYB | 80 | 1.7 |
| 1795 | 2OFZA | 132 | 1.17 |
| 1796 | 2OH1A | 175 | 1.46 |
| 1797 | 2OIDA | 266 | 2.3 |
| 1798 | 2OIKD | 139 | 1.65 |
| 1799 | 2OIWD | 130 | 2 |
| 1800 | 2OIZA | 360 | 1.05 |
| 1801 | 2OIZH | 122 | 1.05 |
| 1802 | 2OJ6A | 162 | 1.85 |
| 1803 | 2OKFA | 129 | 1.6 |
| 1804 | 2OKGB | 251 | 1.65 |
| 1805 | 2OKQB | 118 | 1.8 |
| 1806 | 2OKTA | 342 | 1.3 |
| 1807 | 2OKVD | 149 | 2 |
| 1808 | 2OLMA | 133 | 1.48 |
| 1809 | 2OMKB | 203 | 1.8 |
| 1810 | 2OO9A | 46 | 2.1 |
| 1811 | 2OPCA | 115 | 1.43 |
| 1812 | 2OPEC | 111 | 2.4 |
| 1813 | 2OPIA | 203 | 2.5 |
| 1814 | 2OR7B | 115 | 1.5 |
| 1815 | 2ORDB | 393 | 1.4 |
| 1816 | 2ORWB | 173 | 1.5 |
| 1817 | 2OS0A | 179 | 1.3 |
| 1818 | 2OS5A | 118 | 1.6 |
| 1819 | 2OSAA | 196 | 1.8 |
| 1820 | 2OSXA | 449 | 1.1 |
| 1821 | 2OUID | 360 | 1.77 |
| 1822 | 2OV0A | 105 | 0.75 |
| 1823 | 2OVCA | 30 | 2.07 |
| 1824 | 2OVGA | 58 | 1.35 |
| 1825 | 2OWAB | 126 | 2 |
| 1826 | 2OX0B | 348 | 1.95 |
| 1827 | 2OX8A | 129 | 2.5 |
| 1828 | 2OXCA | 205 | 1.3 |
| 1829 | 2OXLA | 62 | 1.8 |
| 1830 | 2OY9A | 85 | 1.6 |
| 1831 | 2OYAB | 102 | 1.77 |
| 1832 | 2OYYB | 71 | 2.5 |
| 1833 | 2OZFA | 92 | 1.5 |
| 1834 | 2OZJB | 109 | 1.6 |
| 1835 | 2P02A | 380 | 1.21 |
| 1836 | 2P04B | 105 | 2.11 |
| 1837 | 2P09A | 69 | 1.65 |
| 1838 | 2P0BA | 142 | 1.74 |
| 1839 | 2P0LA | 266 | 2.04 |
| 1840 | 2P0NB | 158 | 1.41 |
| 1841 | 2P0WA | 319 | 1.9 |
| 1842 | 2P13B | 83 | 1.65 |
| 1843 | 2P14A | 186 | 1.5 |
| 1844 | 2P1OA | 127 | 1.9 |
| 1845 | 2P25A | 126 | 1.7 |
| 1846 | 2P26A | 280 | 1.75 |
| 1847 | 2P2EA | 119 | 2.48 |
| 1848 | 2P2SA | 333 | 1.25 |
| 1849 | 2P39A | 142 | 1.5 |
| 1850 | 2P3PA | 197 | 1.76 |
| 1851 | 2P4FA | 191 | 1.4 |
| 1852 | 2P58B | 38 | 1.8 |
| 1853 | 2P5KA | 63 | 1 |
| 1854 | 2P5MC | 79 | 1.95 |
| 1855 | 2P64B | 52 | 2.5 |
| 1856 | 2P6WA | 206 | 1.6 |
| 1857 | 2P7IB | 229 | 1.74 |
| 1858 | 2P7OA | 127 | 1.44 |
| 1859 | 2P8GA | 162 | 1.36 |
| 1860 | 2P8IA | 117 | 1.4 |
| 1861 | 2P8JA | 206 | 2 |
| 1862 | 2P92B | 106 | 1.73 |
| 1863 | 2P9RA | 102 | 2.3 |
| 1864 | 2P9WA | 333 | 1.35 |
| 1865 | 2P9XA | 98 | 1.65 |
| 1866 | 2PC1A | 173 | 1.28 |
| 1867 | 2PD1D | 101 | 1.86 |
| 1868 | 2PFIB | 148 | 1.6 |
| 1869 | 2PGOB | 587 | 1.26 |
| 1870 | 2PH7B | 219 | 2.4 |
| 1871 | 2PIEA | 132 | 1.35 |
| 1872 | 2PIHA | 123 | 2.1 |
| 1873 | 2PKDF | 107 | 2.04 |
| 1874 | 2PKFB | 333 | 1.5 |
| 1875 | 2PKHH | 142 | 1.95 |
| 1876 | 2PLXB | 26 | 1.56 |
| 1877 | 2PMRA | 76 | 1.32 |
| 1878 | 2PMUF | 93 | 1.78 |
| 1879 | 2PMYA | 73 | 2.3 |
| 1880 | 2PN0C | 122 | 1.7 |
| 1881 | 2PNDA | 119 | 1 |
| 1882 | 2PNLJ | 203 | 2.21 |
| 1883 | 2PNTB | 95 | 2.15 |
| 1884 | 2PNVB | 39 | 2.1 |
| 1885 | 2POCD | 340 | 1.8 |
| 1886 | 2POFB | 220 | 1.4 |
| 1887 | 2PPLA | 449 | 2.2 |
| 1888 | 2PQ5A | 169 | 2.3 |
| 1889 | 2PQ8A | 260 | 1.45 |
| 1890 | 2PQRD | 45 | 1.88 |
| 1891 | 2PR5B | 126 | 1.45 |
| 1892 | 2PRGC | 32 | 2.3 |
| 1893 | 2PRXB | 114 | 1.5 |
| 1894 | 2PSPB | 106 | 1.95 |
| 1895 | 2PTHA | 193 | 1.2 |
| 1896 | 2PTTB | 108 | 1.63 |
| 1897 | 2PTVA | 96 | 1.66 |
| 1898 | 2PTZA | 431 | 1.65 |
| 1899 | 2PU3A | 207 | 1.5 |
| 1900 | 2PU9A | 110 | 1.65 |
| 1901 | 2PU9B | 74 | 1.65 |
| 1902 | 2PV2D | 103 | 1.3 |
| 1903 | 2PVBA | 107 | 0.91 |
| 1904 | 2PVQA | 201 | 1.8 |
| 1905 | 2PW8I | 62 | 1.84 |
| 1906 | 2PY2A | 127 | 1.7 |
| 1907 | 2PY5A | 564 | 1.6 |
| 1908 | 2Q00B | 122 | 2.4 |
| 1909 | 2Q0IA | 300 | 1.57 |
| 1910 | 2Q0SA | 215 | 1.5 |
| 1911 | 2Q22C | 129 | 2.11 |
| 1912 | 2Q30B | 105 | 1.94 |
| 1913 | 2Q37A | 142 | 2.5 |
| 1914 | 2Q3EA | 460 | 2 |
| 1915 | 2Q3GA | 89 | 1.11 |
| 1916 | 2Q3PA | 103 | 1.9 |
| 1917 | 2Q3TA | 121 | 1.6 |
| 1918 | 2Q3WA | 109 | 1.48 |
| 1919 | 2Q4HB | 305 | 1.83 |
| 1920 | 2Q4IA | 174 | 1.71 |
| 1921 | 2Q4MA | 162 | 1.7 |
| 1922 | 2Q4NA | 153 | 1.32 |
| 1923 | 2Q4PB | 109 | 2.32 |
| 1924 | 2Q52A | 206 | 1.38 |
| 1925 | 2Q5RC | 303 | 2.3 |
| 1926 | 2Q5WD | 77 | 2 |
| 1927 | 2Q7BA | 164 | 2 |
| 1928 | 2Q7DB | 336 | 1.6 |
| 1929 | 2Q87A | 107 | 1.7 |
| 1930 | 2Q8VA | 53 | 2.5 |
| 1931 | 2Q9KA | 147 | 1.59 |
| 1932 | 2QC1B | 212 | 1.94 |
| 1933 | 2QCPX | 80 | 1 |
| 1934 | 2QDQA | 34 | 2.2 |
| 1935 | 2QDXA | 257 | 1.55 |
| 1936 | 2QE8B | 336 | 1.35 |
| 1937 | 2QE9B | 162 | 1.9 |
| 1938 | 2QEAA | 160 | 2.46 |
| 1939 | 2QEBA | 145 | 2 |
| 1940 | 2QF4B | 167 | 1.2 |
| 1941 | 2QFAA | 137 | 1.4 |
| 1942 | 2QFAB | 62 | 1.4 |
| 1943 | 2QFAC | 45 | 1.4 |
| 1944 | 2QG1A | 89 | 1.4 |
| 1945 | 2QG6A | 182 | 1.5 |
| 1946 | 2QGSA | 209 | 2 |
| 1947 | 2QHKA | 148 | 1.91 |
| 1948 | 2QHLA | 110 | 1.56 |
| 1949 | 2QIFB | 68 | 1.5 |
| 1950 | 2QIKA | 269 | 1.35 |
| 1951 | 2QIMA | 157 | 1.35 |
| 1952 | 2QIYB | 133 | 1.69 |
| 1953 | 2QIYD | 25 | 1.69 |
| 1954 | 2QJLA | 99 | 1.44 |
| 1955 | 2QJVB | 263 | 1.9 |
| 1956 | 2QKHB | 32 | 1.9 |
| 1957 | 2QKIF | 295 | 2.4 |
| 1958 | 2QKLB | 90 | 2.33 |
| 1959 | 2QKPA | 138 | 1.75 |
| 1960 | 2QKVB | 92 | 1.55 |
| 1961 | 2QL2D | 57 | 2.5 |
| 1962 | 2QL8A | 141 | 1.5 |
| 1963 | 2QMLA | 193 | 1.55 |
| 1964 | 2QMQA | 278 | 1.7 |
| 1965 | 2QNTA | 126 | 1.4 |
| 1966 | 2QOLA | 273 | 1.07 |
| 1967 | 2QPXA | 376 | 1.4 |
| 1968 | 2QQBA | 214 | 1.92 |
| 1969 | 2QQRA | 118 | 1.8 |
| 1970 | 2QRDC | 114 | 2.41 |
| 1971 | 2QSAA | 101 | 1.68 |
| 1972 | 2QSBA | 85 | 1.3 |
| 1973 | 2QSJA | 120 | 2.1 |
| 1974 | 2QSKA | 95 | 1 |
| 1975 | 2QSQB | 109 | 1.95 |
| 1976 | 2QSWA | 90 | 1.5 |
| 1977 | 2QSXB | 185 | 1.64 |
| 1978 | 2QTIA | 67 | 2.3 |
| 1979 | 2QTQD | 198 | 1.85 |
| 1980 | 2QTVD | 36 | 2.5 |
| 1981 | 2QTXL | 57 | 2.5 |
| 1982 | 2QUXK | 122 | 2.44 |
| 1983 | 2QV3A | 447 | 2.4 |
| 1984 | 2QV8A | 144 | 2 |
| 1985 | 2QVKA | 121 | 1.45 |
| 1986 | 2QVPA | 266 | 2 |
| 1987 | 2QX3B | 330 | 2 |
| 1988 | 2QXVB | 29 | 1.82 |
| 1989 | 2QYZA | 127 | 2.04 |
| 1990 | 2QZID | 98 | 2.2 |
| 1991 | 2QZTB | 110 | 1.7 |
| 1992 | 2QZUA | 465 | 1.7 |
| 1993 | 2R01A | 195 | 1.15 |
| 1994 | 2R2AA | 191 | 1.82 |
| 1995 | 2R2YA | 109 | 1.7 |
| 1996 | 2R31A | 236 | 1 |
| 1997 | 2R4GA | 250 | 1.71 |
| 1998 | 2R4IB | 121 | 1.6 |
| 1999 | 2R5OB | 166 | 1.3 |
| 2000 | 2R5XA | 115 | 2.04 |
| 2001 | 2R6QA | 138 | 1.43 |
| 2002 | 2R6VA | 182 | 1.25 |
| 2003 | 2R78A | 114 | 1.6 |
| 2004 | 2R8OA | 662 | 1.47 |
| 2005 | 2R8UA | 131 | 1.35 |
| 2006 | 2R9GP | 187 | 2.09 |
| 2007 | 2RA2F | 53 | 1.9 |
| 2008 | 2RA4A | 65 | 1.7 |
| 2009 | 2RA9A | 127 | 1.4 |
| 2010 | 2RAFC | 190 | 1.6 |
| 2011 | 2RB5A | 261 | 1.03 |
| 2012 | 2RB8A | 93 | 1.45 |
| 2013 | 2RBGA | 124 | 1.75 |
| 2014 | 2RC3A | 127 | 1.6 |
| 2015 | 2RCIA | 188 | 1.8 |
| 2016 | 2RCZB | 81 | 1.7 |
| 2017 | 2RDEB | 225 | 1.92 |
| 2018 | 2RDGA | 192 | 1.6 |
| 2019 | 2RE9C | 164 | 2.1 |
| 2020 | 2RFRA | 154 | 1.16 |
| 2021 | 2RG9B | 263 | 1.95 |
| 2022 | 2RGQC | 134 | 1.8 |
| 2023 | 2RH0A | 133 | 1.95 |
| 2024 | 2RH2A | 57 | 0.96 |
| 2025 | 2RH3A | 121 | 1.7 |
| 2026 | 2RHFA | 76 | 1.1 |
| 2027 | 2RHKA | 119 | 1.95 |
| 2028 | 2RHKC | 63 | 1.95 |
| 2029 | 2RI0B | 234 | 1.6 |
| 2030 | 2RIKA | 283 | 1.6 |
| 2031 | 2RILA | 95 | 1.26 |
| 2032 | 2RK3A | 187 | 1.05 |
| 2033 | 2RK5A | 86 | 1.5 |
| 2034 | 2RKLB | 50 | 1.5 |
| 2035 | 2RLDE | 108 | 1.7 |
| 2036 | 2SAKA | 121 | 1.8 |
| 2037 | 2SASA | 185 | 2.4 |
| 2038 | 2SCPB | 174 | 2 |
| 2039 | 2SN3A | 65 | 1.2 |
| 2040 | 2TNFC | 148 | 1.4 |
| 2041 | 2TPSA | 226 | 1.25 |
| 2042 | 2UUIA | 155 | 2 |
| 2043 | 2UUKA | 28 | 1.39 |
| 2044 | 2UURA | 210 | 1.8 |
| 2045 | 2UUYB | 52 | 1.15 |
| 2046 | 2UUZA | 89 | 2.3 |
| 2047 | 2UV4A | 143 | 1.33 |
| 2048 | 2UVPD | 176 | 1.7 |
| 2049 | 2UWAA | 274 | 1.8 |
| 2050 | 2UWIB | 127 | 2 |
| 2051 | 2UX0F | 134 | 2.46 |
| 2052 | 2UX9E | 67 | 1.4 |
| 2053 | 2UXYA | 341 | 1.25 |
| 2054 | 2UY2A | 287 | 1.6 |
| 2055 | 2V14A | 134 | 2.2 |
| 2056 | 2V1MA | 163 | 1 |
| 2057 | 2V1OA | 149 | 1.78 |
| 2058 | 2V1QA | 60 | 1.2 |
| 2059 | 2V1TA | 73 | 1.92 |
| 2060 | 2V33B | 91 | 1.55 |
| 2061 | 2V3GA | 273 | 1.2 |
| 2062 | 2V3IA | 434 | 1.05 |
| 2063 | 2V4XA | 131 | 1.5 |
| 2064 | 2V51F | 28 | 2.35 |
| 2065 | 2V52B | 360 | 1.45 |
| 2066 | 2V52M | 30 | 1.45 |
| 2067 | 2V5TA | 184 | 2 |
| 2068 | 2V6KA | 214 | 1.3 |
| 2069 | 2V6VB | 135 | 1.5 |
| 2070 | 2V6XA | 82 | 1.98 |
| 2071 | 2V6XB | 41 | 1.98 |
| 2072 | 2V6YB | 72 | 2.4 |
| 2073 | 2V76B | 102 | 1.6 |
| 2074 | 2V7FA | 139 | 1.15 |
| 2075 | 2V7QJ | 43 | 2.1 |
| 2076 | 2V8QB | 73 | 2.1 |
| 2077 | 2V9LA | 274 | 1.23 |
| 2078 | 2V9VA | 135 | 1.1 |
| 2079 | 2VA1F | 226 | 2.5 |
| 2080 | 2VACA | 134 | 1.7 |
| 2081 | 2VB1A | 129 | 0.65 |
| 2082 | 2VBKA | 511 | 1.25 |
| 2083 | 2VBWB | 147 | 2.2 |
| 2084 | 2VC8A | 72 | 1.31 |
| 2085 | 2VCLA | 205 | 1.55 |
| 2086 | 2VDJA | 268 | 2 |
| 2087 | 2VDUD | 376 | 2.4 |
| 2088 | 2VE8E | 66 | 1.4 |
| 2089 | 2VFOA | 555 | 1.5 |
| 2090 | 2VFRA | 418 | 1.1 |
| 2091 | 2VFXL | 203 | 1.95 |
| 2092 | 2VGOA | 269 | 1.7 |
| 2093 | 2VGOC | 41 | 1.7 |
| 2094 | 2VGXA | 134 | 1.95 |
| 2095 | 2VH3A | 112 | 1.16 |
| 2096 | 2VHAB | 276 | 1 |
| 2097 | 2VIFA | 131 | 1.45 |
| 2098 | 2VKJA | 106 | 1.65 |
| 2099 | 2VKNA | 66 | 2.05 |
| 2100 | 2VLAA | 280 | 1.3 |
| 2101 | 2VLGA | 93 | 1.7 |
| 2102 | 2VLQB | 134 | 1.6 |
| 2103 | 2VN5A | 142 | 1.9 |
| 2104 | 2VN5B | 56 | 1.9 |
| 2105 | 2VOGA | 145 | 1.9 |
| 2106 | 2VPAA | 204 | 1.2 |
| 2107 | 2VPBA | 57 | 1.59 |
| 2108 | 2VPBB | 35 | 1.59 |
| 2109 | 2VPKA | 115 | 2 |
| 2110 | 2VPTA | 201 | 1.4 |
| 2111 | 2VQ4A | 106 | 1.25 |
| 2112 | 2VQCA | 70 | 2.3 |
| 2113 | 2VQEN | 60 | 2.5 |
| 2114 | 2VQEP | 83 | 2.5 |
| 2115 | 2VQEQ | 104 | 2.5 |
| 2116 | 2VQES | 80 | 2.5 |
| 2117 | 2VQPA | 254 | 1.6 |
| 2118 | 2VRNA | 75 | 2.15 |
| 2119 | 2VSHB | 222 | 2 |
| 2120 | 2VSMA | 413 | 1.8 |
| 2121 | 2VT3A | 185 | 2 |
| 2122 | 2VTWF | 205 | 2 |
| 2123 | 2VU4A | 148 | 1.98 |
| 2124 | 2VU6A | 207 | 0.95 |
| 2125 | 2VU9A | 425 | 1.6 |
| 2126 | 2VUVA | 129 | 1.3 |
| 2127 | 2VUWA | 329 | 1.8 |
| 2128 | 2VVPE | 158 | 1.65 |
| 2129 | 2VVWA | 150 | 1.9 |
| 2130 | 2VWRA | 95 | 1.3 |
| 2131 | 2VWSA | 256 | 1.39 |
| 2132 | 2VXNA | 249 | 0.82 |
| 2133 | 2VXTI | 156 | 1.49 |
| 2134 | 2VXXD | 172 | 2.4 |
| 2135 | 2VY1A | 163 | 2.1 |
| 2136 | 2VY8A | 149 | 1.2 |
| 2137 | 2VYND | 334 | 2.2 |
| 2138 | 2VYOA | 206 | 1.5 |
| 2139 | 2VZCA | 127 | 1.05 |
| 2140 | 2VZPB | 127 | 1.05 |
| 2141 | 2W0IA | 135 | 1.8 |
| 2142 | 2W0PA | 93 | 1.9 |
| 2143 | 2W15A | 202 | 1.05 |
| 2144 | 2W1VB | 274 | 1.49 |
| 2145 | 2W2GB | 256 | 2.22 |
| 2146 | 2W2XD | 105 | 2.3 |
| 2147 | 2W39A | 298 | 1.1 |
| 2148 | 2W3QA | 229 | 1.34 |
| 2149 | 2W3ZA | 238 | 1.45 |
| 2150 | 2W4JA | 276 | 1.3 |
| 2151 | 2W4SA | 86 | 2.45 |
| 2152 | 2W6PB | 387 | 1.85 |
| 2153 | 2W72A | 141 | 1.07 |
| 2154 | 2W7AA | 100 | 1.4 |
| 2155 | 2W7VA | 82 | 2.3 |
| 2156 | 2W7ZA | 207 | 1.6 |
| 2157 | 2W8MA | 165 | 2.4 |
| 2158 | 2W8TA | 397 | 1.25 |
| 2159 | 2W8XB | 66 | 1.6 |
| 2160 | 2W91A | 635 | 1.4 |
| 2161 | 2WAGA | 217 | 1.4 |
| 2162 | 2WAOA | 325 | 1.8 |
| 2163 | 2WASB | 118 | 1.9 |
| 2164 | 2WAXD | 28 | 2.3 |
| 2165 | 2WB6A | 114 | 1.95 |
| 2166 | 2WBMA | 234 | 1.75 |
| 2167 | 2WBNA | 178 | 1.9 |
| 2168 | 2WCJA | 141 | 1.4 |
| 2169 | 2WCRA | 147 | 1.7 |
| 2170 | 2WCWB | 122 | 1.58 |
| 2171 | 2WF7A | 218 | 1.05 |
| 2172 | 2WFHB | 181 | 1.8 |
| 2173 | 2WFIA | 172 | 0.75 |
| 2174 | 2WH7A | 152 | 1.6 |
| 2175 | 2WHLA | 294 | 1.4 |
| 2176 | 2WJ5A | 96 | 1.12 |
| 2177 | 2WJ6D | 273 | 2 |
| 2178 | 2WJ9A | 138 | 1.62 |
| 2179 | 2WJRA | 204 | 1.8 |
| 2180 | 2WK1A | 242 | 1.4 |
| 2181 | 2WKBA | 106 | 1.78 |
| 2182 | 2WKDA | 79 | 2.1 |
| 2183 | 2WKJC | 298 | 1.45 |
| 2184 | 2WL1A | 191 | 1.35 |
| 2185 | 2WL8A | 110 | 2.05 |
| 2186 | 2WLCA | 211 | 1.95 |
| 2187 | 2WLVB | 144 | 1.25 |
| 2188 | 2WM3A | 296 | 1.85 |
| 2189 | 2WM9A | 407 | 2.2 |
| 2190 | 2WN3A | 254 | 1.59 |
| 2191 | 2WNFA | 272 | 1.25 |
| 2192 | 2WNKA | 223 | 1.55 |
| 2193 | 2WNOA | 119 | 2.3 |
| 2194 | 2WNVD | 133 | 1.25 |
| 2195 | 2WOJC | 284 | 1.99 |
| 2196 | 2WOYA | 333 | 1.5 |
| 2197 | 2WQ1A | 32 | 1.08 |
| 2198 | 2WQ4B | 130 | 1.42 |
| 2199 | 2WQIA | 45 | 1.7 |
| 2200 | 2WQRA | 319 | 1.9 |
| 2201 | 2WT7B | 90 | 2.3 |
| 2202 | 2WTGA | 158 | 1.5 |
| 2203 | 2WUHA | 163 | 1.6 |
| 2204 | 2WUJB | 50 | 1.4 |
| 2205 | 2WURA | 226 | 0.9 |
| 2206 | 2WUXA | 213 | 1.84 |
| 2207 | 2WWEA | 111 | 1.25 |
| 2208 | 2WX3A | 43 | 2.31 |
| 2209 | 2WY3A | 173 | 1.8 |
| 2210 | 2WY3D | 133 | 1.8 |
| 2211 | 2WY4A | 139 | 1.35 |
| 2212 | 2WY8Q | 66 | 1.7 |
| 2213 | 2WZ1A | 197 | 1.63 |
| 2214 | 2WZ8A | 135 | 1.5 |
| 2215 | 2WZVB | 223 | 1.75 |
| 2216 | 2X1FA | 94 | 1.6 |
| 2217 | 2X27X | 209 | 2.4 |
| 2218 | 2X32B | 176 | 1.55 |
| 2219 | 2X3GA | 116 | 1.8 |
| 2220 | 2X3HC | 498 | 1.6 |
| 2221 | 2X46A | 144 | 1 |
| 2222 | 2X49A | 333 | 1.5 |
| 2223 | 2X4IB | 103 | 2.2 |
| 2224 | 2X4JA | 109 | 1.62 |
| 2225 | 2X55A | 274 | 1.85 |
| 2226 | 2X5NA | 189 | 1.3 |
| 2227 | 2X5PA | 104 | 1.6 |
| 2228 | 2X5XA | 342 | 1.2 |
| 2229 | 2X5YA | 171 | 1.05 |
| 2230 | 2X61A | 245 | 1.95 |
| 2231 | 2X6VB | 172 | 2.2 |
| 2232 | 2X6WA | 596 | 1.35 |
| 2233 | 2X9GA | 251 | 1.1 |
| 2234 | 2X9ZA | 262 | 1.3 |
| 2235 | 2XBLC | 193 | 1.62 |
| 2236 | 2XC8A | 121 | 2.35 |
| 2237 | 2XCJB | 84 | 1.8 |
| 2238 | 2XCME | 74 | 2.2 |
| 2239 | 2XDGA | 89 | 1.95 |
| 2240 | 2XDJF | 62 | 1.82 |
| 2241 | 2XE5A | 343 | 2.28 |
| 2242 | 2XETB | 88 | 1.6 |
| 2243 | 2XEUA | 64 | 1.5 |
| 2244 | 2XF3A | 423 | 1.55 |
| 2245 | 2XFAB | 143 | 2.1 |
| 2246 | 2XFDA | 109 | 1.19 |
| 2247 | 2XFGA | 446 | 1.68 |
| 2248 | 2XFRA | 487 | 0.97 |
| 2249 | 2XGTA | 427 | 1.9 |
| 2250 | 2XGUA | 143 | 1.5 |
| 2251 | 2XHFB | 154 | 1.3 |
| 2252 | 2XHGA | 457 | 1.5 |
| 2253 | 2XHQA | 163 | 1.45 |
| 2254 | 2XI8A | 66 | 1.21 |
| 2255 | 2XIGA | 146 | 1.85 |
| 2256 | 2XIWA | 64 | 1.5 |
| 2257 | 2XJPA | 258 | 0.95 |
| 2258 | 2XMJA | 63 | 1.08 |
| 2259 | 2XMWA | 67 | 1.8 |
| 2260 | 2XNQA | 78 | 1.3 |
| 2261 | 2XODA | 118 | 0.96 |
| 2262 | 2XOLA | 168 | 1.35 |
| 2263 | 2XOMA | 145 | 0.95 |
| 2264 | 2XQQD | 85 | 1.31 |
| 2265 | 2XR6A | 130 | 1.35 |
| 2266 | 2XRHA | 100 | 1.5 |
| 2267 | 2XS2A | 87 | 1.35 |
| 2268 | 2XSTA | 149 | 1.63 |
| 2269 | 2XT1A | 86 | 1.32 |
| 2270 | 2XTCA | 74 | 2.22 |
| 2271 | 2XTMB | 199 | 1.7 |
| 2272 | 2XTSC | 389 | 1.33 |
| 2273 | 2XTSD | 204 | 1.33 |
| 2274 | 2XTTA | 35 | 0.93 |
| 2275 | 2XTYB | 207 | 1.8 |
| 2276 | 2XUSB | 40 | 1.91 |
| 2277 | 2XVCA | 56 | 2.15 |
| 2278 | 2XVTF | 79 | 2.05 |
| 2279 | 2XWVA | 309 | 1.05 |
| 2280 | 2XXNA | 143 | 1.6 |
| 2281 | 2XXQA | 373 | 1.77 |
| 2282 | 2XZ4A | 165 | 1.72 |
| 2283 | 2XZZA | 97 | 2.3 |
| 2284 | 2Y0OA | 171 | 1.23 |
| 2285 | 2Y1BA | 86 | 2 |
| 2286 | 2Y1KA | 149 | 2.5 |
| 2287 | 2Y1TD | 128 | 1.89 |
| 2288 | 2Y27A | 425 | 1.6 |
| 2289 | 2Y2ZA | 238 | 1.95 |
| 2290 | 2Y3CA | 278 | 1.4 |
| 2291 | 2Y3MB | 138 | 2.3 |
| 2292 | 2Y3NB | 60 | 1.9 |
| 2293 | 2Y3YA | 83 | 2.39 |
| 2294 | 2Y43A | 88 | 1.8 |
| 2295 | 2Y4SA | 863 | 2.1 |
| 2296 | 2Y4ZA | 135 | 2 |
| 2297 | 2Y5FL | 54 | 1.29 |
| 2298 | 2Y5PA | 72 | 1.3 |
| 2299 | 2Y6HA | 167 | 1.08 |
| 2300 | 2Y6UA | 371 | 1.9 |
| 2301 | 2Y6XA | 109 | 1.6 |
| 2302 | 2Y72A | 85 | 1.18 |
| 2303 | 2Y78A | 122 | 0.91 |
| 2304 | 2Y7LA | 312 | 1.49 |
| 2305 | 2Y8DA | 281 | 1.84 |
| 2306 | 2Y8NB | 86 | 1.75 |
| 2307 | 2Y8TE | 36 | 1.95 |
| 2308 | 2Y9UA | 67 | 1.6 |
| 2309 | 2Y9WD | 135 | 2.3 |
| 2310 | 2YADA | 77 | 2.2 |
| 2311 | 2YB1A | 284 | 1.9 |
| 2312 | 2YC3A | 219 | 1.4 |
| 2313 | 2YD6A | 195 | 1.35 |
| 2314 | 2YEOA | 65 | 1.08 |
| 2315 | 2YFOA | 719 | 1.35 |
| 2316 | 2YFVC | 60 | 2.32 |
| 2317 | 2YG2A | 162 | 1.7 |
| 2318 | 2YGGA | 66 | 2.23 |
| 2319 | 2YH5A | 121 | 1.25 |
| 2320 | 2YH6A | 108 | 1.55 |
| 2321 | 2YHGA | 411 | 1.08 |
| 2322 | 2YHOA | 67 | 2.1 |
| 2323 | 2YILD | 129 | 1.95 |
| 2324 | 2YKZA | 126 | 0.84 |
| 2325 | 2YLNA | 240 | 1.12 |
| 2326 | 2YV0X | 155 | 1.4 |
| 2327 | 2YVEB | 172 | 1.4 |
| 2328 | 2YVQA | 134 | 1.98 |
| 2329 | 2YVRB | 45 | 1.8 |
| 2330 | 2YVTA | 257 | 1.6 |
| 2331 | 2YWKA | 84 | 1.54 |
| 2332 | 2YWNA | 150 | 1.6 |
| 2333 | 2YWWA | 141 | 2 |
| 2334 | 2YX8A | 81 | 2.4 |
| 2335 | 2YXMA | 92 | 1.51 |
| 2336 | 2YXTA | 304 | 2 |
| 2337 | 2YY3C | 91 | 2.5 |
| 2338 | 2YZYA | 163 | 1.6 |
| 2339 | 2Z0BA | 111 | 2 |
| 2340 | 2Z0TA | 109 | 1.8 |
| 2341 | 2Z0UA | 128 | 2.2 |
| 2342 | 2Z1EA | 298 | 1.55 |
| 2343 | 2Z30B | 65 | 1.65 |
| 2344 | 2Z3QD | 77 | 1.85 |
| 2345 | 2Z3XA | 56 | 2.1 |
| 2346 | 2Z4UA | 261 | 1.1 |
| 2347 | 2Z72A | 338 | 1.1 |
| 2348 | 2Z7FI | 50 | 1.7 |
| 2349 | 2Z80A | 321 | 1.8 |
| 2350 | 2ZAYA | 123 | 2 |
| 2351 | 2ZC2B | 75 | 2.1 |
| 2352 | 2ZCMA | 177 | 1.33 |
| 2353 | 2ZD7B | 212 | 1.85 |
| 2354 | 2ZDPA | 110 | 1.5 |
| 2355 | 2ZEXA | 147 | 1.2 |
| 2356 | 2ZFIA | 329 | 1.55 |
| 2357 | 2ZHJA | 315 | 1.35 |
| 2358 | 2ZK9X | 185 | 1.15 |
| 2359 | 2ZKMX | 708 | 1.62 |
| 2360 | 2ZKOB | 70 | 1.7 |
| 2361 | 2ZKZD | 82 | 2 |
| 2362 | 2ZNRA | 178 | 1.2 |
| 2363 | 2ZOUB | 140 | 1.45 |
| 2364 | 2ZP1A | 309 | 1.7 |
| 2365 | 2ZPTX | 288 | 1.15 |
| 2366 | 2ZPUA | 320 | 1.7 |
| 2367 | 2ZQOB | 130 | 1.8 |
| 2368 | 2ZRRA | 83 | 1.8 |
| 2369 | 2ZS0C | 147 | 1.6 |
| 2370 | 2ZSIB | 60 | 1.8 |
| 2371 | 2ZU0D | 42 | 2.2 |
| 2372 | 2ZUXB | 582 | 1.32 |
| 2373 | 2ZW2A | 85 | 1.55 |
| 2374 | 2ZX2B | 195 | 1.8 |
| 2375 | 2ZXEB | 265 | 2.4 |
| 2376 | 2ZXEG | 39 | 2.4 |
| 2377 | 2ZXKA | 266 | 2.5 |
| 2378 | 2ZXYA | 86 | 1.15 |
| 2379 | 2ZYZC | 96 | 1.7 |
| 2380 | 2ZZDB | 153 | 1.78 |
| 2381 | 3A02A | 51 | 1 |
| 2382 | 3A07B | 118 | 1.19 |
| 2383 | 3A0MC | 25 | 1.02 |
| 2384 | 3A0SA | 96 | 1.47 |
| 2385 | 3A1BA | 148 | 2.29 |
| 2386 | 3A1GC | 73 | 1.7 |
| 2387 | 3A1QC | 45 | 2.2 |
| 2388 | 3A2OB | 99 | 0.88 |
| 2389 | 3A2VJ | 243 | 1.65 |
| 2390 | 3A2ZA | 190 | 1.5 |
| 2391 | 3A35B | 184 | 1.42 |
| 2392 | 3A38A | 83 | 0.7 |
| 2393 | 3A4RB | 75 | 1 |
| 2394 | 3A4UB | 65 | 1.84 |
| 2395 | 3A57A | 154 | 1.5 |
| 2396 | 3A5FB | 291 | 1.19 |
| 2397 | 3A72A | 353 | 1.04 |
| 2398 | 3A7OA | 59 | 2.5 |
| 2399 | 3A8RA | 166 | 2.4 |
| 2400 | 3A9FB | 77 | 1.3 |
| 2401 | 3A9JB | 77 | 1.18 |
| 2402 | 3A9JC | 32 | 1.18 |
| 2403 | 3A9SC | 589 | 1.6 |
| 2404 | 3AA0A | 270 | 1.7 |
| 2405 | 3AAIB | 77 | 2.1 |
| 2406 | 3AAYA | 272 | 1.9 |
| 2407 | 3ABDB | 195 | 1.9 |
| 2408 | 3ACXA | 284 | 1.31 |
| 2409 | 3ACZA | 387 | 1.97 |
| 2410 | 3AD8D | 91 | 2.2 |
| 2411 | 3AEIB | 94 | 1.7 |
| 2412 | 3AG3B | 227 | 1.8 |
| 2413 | 3AG3G | 84 | 1.8 |
| 2414 | 3AG3H | 79 | 1.8 |
| 2415 | 3AG3J | 58 | 1.8 |
| 2416 | 3AG3K | 49 | 1.8 |
| 2417 | 3AG3L | 46 | 1.8 |
| 2418 | 3AG3V | 73 | 1.8 |
| 2419 | 3AGNA | 114 | 0.96 |
| 2420 | 3AIAB | 200 | 1.4 |
| 2421 | 3AJ4A | 112 | 1 |
| 2422 | 3AJ6B | 284 | 1.48 |
| 2423 | 3AJBB | 26 | 2.5 |
| 2424 | 3AJDA | 254 | 1.27 |
| 2425 | 3AJFD | 90 | 2 |
| 2426 | 3AJID | 73 | 2.05 |
| 2427 | 3AJMA | 201 | 2.3 |
| 2428 | 3AK0B | 132 | 1.59 |
| 2429 | 3AK9J | 155 | 1.3 |
| 2430 | 3AKHA | 447 | 1.7 |
| 2431 | 3ALJA | 369 | 1.48 |
| 2432 | 3ALNC | 183 | 2.3 |
| 2433 | 3ALRD | 55 | 2.1 |
| 2434 | 3ALUA | 157 | 1.65 |
| 2435 | 3AMNB | 256 | 1.47 |
| 2436 | 3AMRA | 352 | 1.25 |
| 2437 | 3ANUA | 367 | 1.9 |
| 2438 | 3AONA | 188 | 2 |
| 2439 | 3AOWC | 404 | 1.56 |
| 2440 | 3APSA | 114 | 1.9 |
| 2441 | 3AQIB | 359 | 1.7 |
| 2442 | 3ARCF | 34 | 1.9 |
| 2443 | 3ARCH | 65 | 1.9 |
| 2444 | 3ARCI | 38 | 1.9 |
| 2445 | 3ARCJ | 38 | 1.9 |
| 2446 | 3ARCK | 37 | 1.9 |
| 2447 | 3ARCL | 37 | 1.9 |
| 2448 | 3ARCM | 34 | 1.9 |
| 2449 | 3ARCt | 30 | 1.9 |
| 2450 | 3ARCu | 97 | 1.9 |
| 2451 | 3ARCx | 39 | 1.9 |
| 2452 | 3ARCy | 29 | 1.9 |
| 2453 | 3ASLA | 68 | 1.41 |
| 2454 | 3AU4B | 32 | 1.9 |
| 2455 | 3AWGC | 308 | 2.39 |
| 2456 | 3AWUA | 278 | 1.16 |
| 2457 | 3AWUB | 78 | 1.16 |
| 2458 | 3AZDA | 30 | 0.98 |
| 2459 | 3B08B | 58 | 1.7 |
| 2460 | 3B0BC | 75 | 2.15 |
| 2461 | 3B0DB | 95 | 2.2 |
| 2462 | 3B0DC | 75 | 2.2 |
| 2463 | 3B0FA | 43 | 1.4 |
| 2464 | 3B1FA | 286 | 2.1 |
| 2465 | 3B21A | 192 | 2.01 |
| 2466 | 3B33A | 109 | 1.83 |
| 2467 | 3B4UA | 287 | 1.2 |
| 2468 | 3B5NH | 63 | 1.6 |
| 2469 | 3B5NI | 58 | 1.6 |
| 2470 | 3B5NK | 65 | 1.6 |
| 2471 | 3B5OA | 230 | 1.35 |
| 2472 | 3B64A | 112 | 1.03 |
| 2473 | 3B79A | 125 | 1.37 |
| 2474 | 3B7CA | 121 | 1.7 |
| 2475 | 3B7EA | 385 | 1.45 |
| 2476 | 3B7FA | 368 | 2.2 |
| 2477 | 3B7HA | 76 | 2 |
| 2478 | 3B93A | 123 | 2.2 |
| 2479 | 3B9CC | 134 | 1.9 |
| 2480 | 3B9WA | 362 | 1.3 |
| 2481 | 3BALD | 149 | 1.95 |
| 2482 | 3BB0A | 576 | 1.5 |
| 2483 | 3BB6A | 109 | 2.3 |
| 2484 | 3BB7A | 314 | 1.5 |
| 2485 | 3BB9F | 120 | 1.8 |
| 2486 | 3BBDA | 204 | 2.15 |
| 2487 | 3BC1F | 51 | 1.8 |
| 2488 | 3BCWA | 118 | 1.6 |
| 2489 | 3BD1C | 64 | 1.4 |
| 2490 | 3BDIA | 207 | 1.45 |
| 2491 | 3BDWD | 116 | 2.5 |
| 2492 | 3BEEB | 91 | 2.15 |
| 2493 | 3BEMA | 207 | 1.65 |
| 2494 | 3BF4A | 115 | 2.1 |
| 2495 | 3BF5A | 284 | 1.91 |
| 2496 | 3BF7A | 255 | 1.1 |
| 2497 | 3BFQG | 132 | 1.34 |
| 2498 | 3BGUB | 104 | 1.5 |
| 2499 | 3BGYA | 226 | 1.65 |
| 2500 | 3BH2A | 244 | 2.4 |
| 2501 | 3BHDA | 211 | 1.5 |
| 2502 | 3BHWA | 182 | 1.5 |
| 2503 | 3BI1A | 694 | 1.5 |
| 2504 | 3BJ5A | 130 | 2.2 |
| 2505 | 3BJ91 | 116 | 2 |
| 2506 | 3BJQA | 296 | 2.05 |
| 2507 | 3BKHA | 268 | 2.5 |
| 2508 | 3BL2B | 131 | 2.3 |
| 2509 | 3BL4A | 120 | 2.2 |
| 2510 | 3BLNA | 142 | 1.31 |
| 2511 | 3BLZA | 124 | 1.75 |
| 2512 | 3BMXA | 617 | 1.4 |
| 2513 | 3BNJA | 471 | 1.3 |
| 2514 | 3BNWB | 157 | 2.4 |
| 2515 | 3BNYD | 297 | 1.89 |
| 2516 | 3BOEA | 209 | 1.4 |
| 2517 | 3BONA | 416 | 1.2 |
| 2518 | 3BPJD | 69 | 1.85 |
| 2519 | 3BPQA | 41 | 2.2 |
| 2520 | 3BPTA | 362 | 1.5 |
| 2521 | 3BPUA | 86 | 1.6 |
| 2522 | 3BQAA | 146 | 2 |
| 2523 | 3BQPB | 80 | 1.3 |
| 2524 | 3BRCA | 155 | 1.6 |
| 2525 | 3BS1A | 103 | 1.6 |
| 2526 | 3BS4A | 245 | 1.6 |
| 2527 | 3BSUA | 48 | 2.1 |
| 2528 | 3BT2U | 259 | 2.5 |
| 2529 | 3BT4A | 85 | 2.1 |
| 2530 | 3BT5A | 151 | 1.35 |
| 2531 | 3BUTA | 125 | 1.91 |
| 2532 | 3BUUA | 224 | 1.2 |
| 2533 | 3BUXB | 305 | 1.35 |
| 2534 | 3BV8A | 85 | 1.75 |
| 2535 | 3BVFF | 172 | 1.5 |
| 2536 | 3BVPB | 130 | 2.1 |
| 2537 | 3BVUA | 1015 | 1.12 |
| 2538 | 3BW1A | 86 | 2.5 |
| 2539 | 3BWLA | 125 | 1.73 |
| 2540 | 3BWUD | 121 | 1.76 |
| 2541 | 3BWVA | 167 | 1.55 |
| 2542 | 3BWYA | 215 | 1.3 |
| 2543 | 3BWZA | 171 | 1.2 |
| 2544 | 3BX4D | 123 | 1.7 |
| 2545 | 3BXUA | 71 | 1.35 |
| 2546 | 3BY4A | 172 | 1.55 |
| 2547 | 3BY9A | 259 | 1.7 |
| 2548 | 3BYJA | 440 | 2.1 |
| 2549 | 3BYPA | 82 | 1.7 |
| 2550 | 3BZNA | 430 | 2 |
| 2551 | 3BZYB | 83 | 1.2 |
| 2552 | 3C0FB | 85 | 1.8 |
| 2553 | 3C1DB | 154 | 1.8 |
| 2554 | 3C1QA | 115 | 1.7 |
| 2555 | 3C24B | 274 | 1.62 |
| 2556 | 3C26A | 257 | 2 |
| 2557 | 3C2EA | 267 | 1.9 |
| 2558 | 3C2UB | 537 | 1.3 |
| 2559 | 3C3MA | 123 | 1.7 |
| 2560 | 3C3YA | 225 | 1.37 |
| 2561 | 3C4SB | 55 | 1.7 |
| 2562 | 3C5CB | 167 | 1.85 |
| 2563 | 3C5TB | 25 | 2.1 |
| 2564 | 3C64A | 152 | 2.4 |
| 2565 | 3C68A | 758 | 1.5 |
| 2566 | 3C6AA | 198 | 1.16 |
| 2567 | 3C6KC | 346 | 1.95 |
| 2568 | 3C6VA | 143 | 1.9 |
| 2569 | 3C6WC | 52 | 1.75 |
| 2570 | 3C70A | 256 | 1.05 |
| 2571 | 3C7XA | 196 | 1.7 |
| 2572 | 3C8CA | 240 | 1.5 |
| 2573 | 3C8GD | 161 | 2.5 |
| 2574 | 3C8LB | 119 | 1.22 |
| 2575 | 3C8ZB | 401 | 1.6 |
| 2576 | 3C9FA | 531 | 1.9 |
| 2577 | 3C9QA | 195 | 1.5 |
| 2578 | 3CA7A | 50 | 1.5 |
| 2579 | 3CB0A | 166 | 1.6 |
| 2580 | 3CBNA | 144 | 1.63 |
| 2581 | 3CBWA | 336 | 1.27 |
| 2582 | 3CC2M | 194 | 2.4 |
| 2583 | 3CDLB | 188 | 2.36 |
| 2584 | 3CDXD | 322 | 2.1 |
| 2585 | 3CEGA | 292 | 2.01 |
| 2586 | 3CEWD | 118 | 2.31 |
| 2587 | 3CFUA | 145 | 2.4 |
| 2588 | 3CG6B | 128 | 1.7 |
| 2589 | 3CG7A | 296 | 2.5 |
| 2590 | 3CGLF | 60 | 2.09 |
| 2591 | 3CH0A | 272 | 1.5 |
| 2592 | 3CHBH | 103 | 1.25 |
| 2593 | 3CI0I | 83 | 2.2 |
| 2594 | 3CI3A | 188 | 1.11 |
| 2595 | 3CI9A | 44 | 1.8 |
| 2596 | 3CIJB | 291 | 1.07 |
| 2597 | 3CIPG | 128 | 1.6 |
| 2598 | 3CJJA | 219 | 1.85 |
| 2599 | 3CJSA | 58 | 1.37 |
| 2600 | 3CJSC | 68 | 1.37 |
| 2601 | 3CJWA | 207 | 1.48 |
| 2602 | 3CKMA | 318 | 1.35 |
| 2603 | 3CLMA | 352 | 1.14 |
| 2604 | 3CM3A | 164 | 1.32 |
| 2605 | 3CNED | 174 | 1.99 |
| 2606 | 3CNHA | 200 | 1.66 |
| 2607 | 3CNRA | 91 | 1.9 |
| 2608 | 3CNYA | 300 | 1.85 |
| 2609 | 3CO5B | 132 | 2.4 |
| 2610 | 3CP5A | 116 | 1.24 |
| 2611 | 3CP7A | 216 | 1.39 |
| 2612 | 3CPXA | 309 | 2.39 |
| 2613 | 3CQ0B | 323 | 1.9 |
| 2614 | 3CQLB | 243 | 1.5 |
| 2615 | 3CS5A | 48 | 2.2 |
| 2616 | 3CT5A | 159 | 1.37 |
| 2617 | 3CT6B | 128 | 1.1 |
| 2618 | 3CT9B | 347 | 2.31 |
| 2619 | 3CTPA | 266 | 1.41 |
| 2620 | 3CTZA | 617 | 1.6 |
| 2621 | 3CU2B | 236 | 1.91 |
| 2622 | 3CU4A | 79 | 1.3 |
| 2623 | 3CU9A | 314 | 1.06 |
| 2624 | 3CWRA | 193 | 1.5 |
| 2625 | 3CX5H | 93 | 1.9 |
| 2626 | 3CX5I | 57 | 1.9 |
| 2627 | 3CX5Q | 74 | 1.9 |
| 2628 | 3CXGB | 122 | 2 |
| 2629 | 3CXKB | 131 | 1.7 |
| 2630 | 3CYPB | 129 | 1.6 |
| 2631 | 3CZ1A | 117 | 1.5 |
| 2632 | 3CZ6A | 151 | 1.85 |
| 2633 | 3CZ8A | 302 | 2.2 |
| 2634 | 3CZQA | 288 | 2.23 |
| 2635 | 3CZTX | 91 | 1.4 |
| 2636 | 3CZZA | 101 | 1.36 |
| 2637 | 3D02A | 300 | 1.3 |
| 2638 | 3D06A | 179 | 1.2 |
| 2639 | 3D0JA | 138 | 1.53 |
| 2640 | 3D1BC | 108 | 1.7 |
| 2641 | 3D1PA | 120 | 0.98 |
| 2642 | 3D2QD | 69 | 1.5 |
| 2643 | 3D2WA | 72 | 1.65 |
| 2644 | 3D32B | 116 | 1.3 |
| 2645 | 3D33A | 94 | 1.7 |
| 2646 | 3D36C | 42 | 2.03 |
| 2647 | 3D3BA | 139 | 1.3 |
| 2648 | 3D3BJ | 87 | 1.3 |
| 2649 | 3D3MA | 161 | 1.9 |
| 2650 | 3D3OA | 176 | 2.46 |
| 2651 | 3D3SD | 145 | 1.87 |
| 2652 | 3D59A | 372 | 1.5 |
| 2653 | 3D5PA | 133 | 1.45 |
| 2654 | 3D6IB | 107 | 1.5 |
| 2655 | 3D6MA | 173 | 1.8 |
| 2656 | 3D7AB | 134 | 1.9 |
| 2657 | 3D7RB | 295 | 2.01 |
| 2658 | 3D89A | 136 | 2.07 |
| 2659 | 3D9NB | 140 | 1.6 |
| 2660 | 3D9RA | 132 | 2.4 |
| 2661 | 3D9SD | 246 | 2 |
| 2662 | 3D9TB | 95 | 1.5 |
| 2663 | 3D9XA | 114 | 1.13 |
| 2664 | 3DA5A | 121 | 1.94 |
| 2665 | 3DA8B | 205 | 1.3 |
| 2666 | 3DALB | 166 | 1.65 |
| 2667 | 3DAOA | 265 | 1.8 |
| 2668 | 3DASA | 334 | 1.6 |
| 2669 | 3DAUA | 159 | 1.5 |
| 2670 | 3DB2A | 347 | 1.7 |
| 2671 | 3DB7A | 127 | 1.4 |
| 2672 | 3DCMX | 189 | 2 |
| 2673 | 3DCZA | 170 | 1.65 |
| 2674 | 3DDCB | 133 | 1.8 |
| 2675 | 3DDOB | 244 | 1.5 |
| 2676 | 3DDTA | 48 | 1.9 |
| 2677 | 3DEDF | 86 | 2.14 |
| 2678 | 3DF6D | 98 | 2.05 |
| 2679 | 3DF8A | 109 | 1.65 |
| 2680 | 3DHAA | 254 | 0.95 |
| 2681 | 3DHXA | 99 | 2.1 |
| 2682 | 3DJ9A | 107 | 1.75 |
| 2683 | 3DK9A | 462 | 0.95 |
| 2684 | 3DL1A | 210 | 2.2 |
| 2685 | 3DLCA | 219 | 1.15 |
| 2686 | 3DLQR | 201 | 1.9 |
| 2687 | 3DLVA | 92 | 1.87 |
| 2688 | 3DM8A | 135 | 1.8 |
| 2689 | 3DMLA | 95 | 1.9 |
| 2690 | 3DMOD | 133 | 1.6 |
| 2691 | 3DNJB | 81 | 1.15 |
| 2692 | 3DNSB | 129 | 2.1 |
| 2693 | 3DNTA | 415 | 1.66 |
| 2694 | 3DO8B | 123 | 1.6 |
| 2695 | 3DQGA | 148 | 1.72 |
| 2696 | 3DQYA | 106 | 1.2 |
| 2697 | 3DR0C | 93 | 1.23 |
| 2698 | 3DR5A | 216 | 2.25 |
| 2699 | 3DRAA | 302 | 1.8 |
| 2700 | 3DRFA | 539 | 1.3 |
| 2701 | 3DSBA | 152 | 1.48 |
| 2702 | 3DSOA | 66 | 1.55 |
| 2703 | 3DTNB | 213 | 2.09 |
| 2704 | 3DTZB | 221 | 1.81 |
| 2705 | 3DXEB | 27 | 2 |
| 2706 | 3DXLA | 299 | 1.3 |
| 2707 | 3DXRB | 69 | 2.5 |
| 2708 | 3DXYA | 208 | 1.5 |
| 2709 | 3DY0A | 327 | 1.55 |
| 2710 | 3DY0B | 28 | 1.55 |
| 2711 | 3E0XA | 245 | 1.45 |
| 2712 | 3E10B | 167 | 1.4 |
| 2713 | 3E19C | 77 | 2 |
| 2714 | 3E1IA | 57 | 2.3 |
| 2715 | 3E1RB | 42 | 2 |
| 2716 | 3E21A | 40 | 1.73 |
| 2717 | 3E3MD | 270 | 1.6 |
| 2718 | 3E3UA | 196 | 1.56 |
| 2719 | 3E3VA | 154 | 2.04 |
| 2720 | 3E4HA | 29 | 1.8 |
| 2721 | 3E5AB | 33 | 2.3 |
| 2722 | 3E5UA | 215 | 1.83 |
| 2723 | 3E7KG | 52 | 2.01 |
| 2724 | 3E7RL | 40 | 1 |
| 2725 | 3E8MA | 164 | 1.1 |
| 2726 | 3E8OA | 102 | 1.4 |
| 2727 | 3E8TA | 216 | 1.3 |
| 2728 | 3E8YX | 30 | 1.1 |
| 2729 | 3E98B | 174 | 2.43 |
| 2730 | 3E9FA | 99 | 1.8 |
| 2731 | 3E9TA | 112 | 1.6 |
| 2732 | 3E9VA | 120 | 1.7 |
| 2733 | 3EA6A | 83 | 0.92 |
| 2734 | 3EA6A | 132 | 0.92 |
| 2735 | 3EABA | 89 | 2.5 |
| 2736 | 3EABG | 40 | 2.5 |
| 2737 | 3EARB | 50 | 2.3 |
| 2738 | 3EATX | 278 | 2.5 |
| 2739 | 3EBTA | 131 | 1.3 |
| 2740 | 3EC6A | 129 | 1.6 |
| 2741 | 3ECOB | 129 | 2.4 |
| 2742 | 3ED1C | 301 | 1.9 |
| 2743 | 3EDOB | 149 | 1.2 |
| 2744 | 3EDVB | 321 | 1.95 |
| 2745 | 3EE4A | 289 | 1.9 |
| 2746 | 3EEHA | 116 | 1.95 |
| 2747 | 3EEIA | 231 | 1.78 |
| 2748 | 3EF4C | 124 | 1.18 |
| 2749 | 3EF8A | 149 | 1.5 |
| 2750 | 3EFGA | 51 | 2 |
| 2751 | 3EFYB | 182 | 1.7 |
| 2752 | 3EG3A | 63 | 1.4 |
| 2753 | 3EGAA | 219 | 1.8 |
| 2754 | 3EGGD | 66 | 1.85 |
| 2755 | 3EGNA | 120 | 2.5 |
| 2756 | 3EGWC | 225 | 1.9 |
| 2757 | 3EHCC | 127 | 2.12 |
| 2758 | 3EHGA | 125 | 1.74 |
| 2759 | 3EIKA | 178 | 1.9 |
| 2760 | 3EINA | 207 | 1.13 |
| 2761 | 3EIXA | 290 | 1.35 |
| 2762 | 3EJ9E | 64 | 1.5 |
| 2763 | 3EJ9F | 58 | 1.5 |
| 2764 | 3EJJX | 272 | 2.4 |
| 2765 | 3ELFA | 332 | 1.31 |
| 2766 | 3ELKB | 103 | 1.7 |
| 2767 | 3ELNA | 188 | 1.42 |
| 2768 | 3EMFC | 113 | 2 |
| 2769 | 3EMUA | 144 | 2.3 |
| 2770 | 3EN0C | 260 | 1.5 |
| 2771 | 3ENGA | 213 | 1.9 |
| 2772 | 3ENUA | 114 | 1.86 |
| 2773 | 3EO6A | 106 | 0.97 |
| 2774 | 3EOIB | 123 | 1.52 |
| 2775 | 3EOJA | 358 | 1.3 |
| 2776 | 3EP6B | 57 | 1.7 |
| 2777 | 3EPBA | 253 | 1.75 |
| 2778 | 3EPWA | 326 | 1.3 |
| 2779 | 3EPZB | 223 | 2.31 |
| 2780 | 3EQAA | 458 | 1.9 |
| 2781 | 3EQZB | 125 | 2.15 |
| 2782 | 3ERBA | 188 | 1.8 |
| 2783 | 3ERJB | 111 | 1.8 |
| 2784 | 3ERME | 58 | 2.45 |
| 2785 | 3ES4B | 116 | 1.64 |
| 2786 | 3ESLA | 195 | 1.74 |
| 2787 | 3ESSA | 199 | 1.19 |
| 2788 | 3ETIA | 168 | 2.2 |
| 2789 | 3ETQA | 194 | 1.9 |
| 2790 | 3EUDA | 99 | 2.4 |
| 2791 | 3EULD | 72 | 1.9 |
| 2792 | 3EURA | 140 | 1.3 |
| 2793 | 3EVFA | 262 | 1.45 |
| 2794 | 3EVZA | 197 | 2.2 |
| 2795 | 3EW0A | 77 | 1.4 |
| 2796 | 3EWMB | 291 | 1.9 |
| 2797 | 3EY6A | 118 | 1.05 |
| 2798 | 3EYEA | 153 | 1.45 |
| 2799 | 3EYIA | 64 | 1.45 |
| 2800 | 3EYPB | 459 | 1.9 |
| 2801 | 3EZIB | 99 | 1.7 |
| 2802 | 3F02A | 322 | 1.8 |
| 2803 | 3F0DD | 157 | 1.2 |
| 2804 | 3F0HA | 362 | 1.7 |
| 2805 | 3F14A | 112 | 1.45 |
| 2806 | 3F1LB | 244 | 0.95 |
| 2807 | 3F1PA | 114 | 1.17 |
| 2808 | 3F1PB | 111 | 1.17 |
| 2809 | 3F1TC | 135 | 2.2 |
| 2810 | 3F2KB | 183 | 1.85 |
| 2811 | 3F2ZA | 149 | 1.3 |
| 2812 | 3F3BA | 115 | 2.5 |
| 2813 | 3F42B | 84 | 1.78 |
| 2814 | 3F52A | 77 | 1.75 |
| 2815 | 3F5HB | 57 | 1.75 |
| 2816 | 3F5OH | 138 | 1.7 |
| 2817 | 3F65E | 194 | 2.29 |
| 2818 | 3F6CB | 128 | 1.45 |
| 2819 | 3F6GA | 121 | 2 |
| 2820 | 3F6QB | 72 | 1.6 |
| 2821 | 3F75P | 75 | 1.99 |
| 2822 | 3F7EB | 126 | 1.23 |
| 2823 | 3F7QA | 214 | 1.75 |
| 2824 | 3F7SA | 142 | 2.11 |
| 2825 | 3F8BB | 105 | 2 |
| 2826 | 3F8XA | 131 | 1.55 |
| 2827 | 3F95A | 175 | 1.8 |
| 2828 | 3F9FB | 299 | 2.3 |
| 2829 | 3F9SA | 143 | 1.76 |
| 2830 | 3F9TB | 392 | 2.11 |
| 2831 | 3F9XA | 160 | 1.25 |
| 2832 | 3FA2A | 203 | 2.2 |
| 2833 | 3FAUA | 71 | 1.9 |
| 2834 | 3FAVC | 90 | 2.15 |
| 2835 | 3FBGB | 332 | 1.6 |
| 2836 | 3FCDA | 117 | 1.92 |
| 2837 | 3FCIA | 223 | 1.27 |
| 2838 | 3FCNA | 158 | 1.45 |
| 2839 | 3FCXA | 268 | 1.5 |
| 2840 | 3FDEB | 206 | 1.41 |
| 2841 | 3FDJA | 276 | 1.8 |
| 2842 | 3FDLB | 25 | 1.78 |
| 2843 | 3FDRA | 89 | 1.75 |
| 2844 | 3FDWB | 130 | 2.2 |
| 2845 | 3FETD | 164 | 2.05 |
| 2846 | 3FF7D | 112 | 1.8 |
| 2847 | 3FFYA | 112 | 2 |
| 2848 | 3FGHA | 67 | 1.35 |
| 2849 | 3FGVA | 94 | 1.3 |
| 2850 | 3FH3B | 147 | 2.1 |
| 2851 | 3FILA | 56 | 0.88 |
| 2852 | 3FIQA | 154 | 1.6 |
| 2853 | 3FJ2A | 170 | 1.85 |
| 2854 | 3FJUB | 65 | 1.6 |
| 2855 | 3FK8A | 131 | 1.3 |
| 2856 | 3FKAA | 119 | 1.69 |
| 2857 | 3FKMX | 120 | 2.5 |
| 2858 | 3FLEB | 243 | 2.01 |
| 2859 | 3FLPE | 217 | 2.3 |
| 2860 | 3FM5D | 138 | 2 |
| 2861 | 3FM8B | 95 | 2.3 |
| 2862 | 3FMBB | 103 | 1.85 |
| 2863 | 3FMFD | 220 | 2.05 |
| 2864 | 3FMYA | 66 | 1.4 |
| 2865 | 3FNCB | 161 | 1.75 |
| 2866 | 3FO3B | 519 | 1.4 |
| 2867 | 3FOTA | 505 | 1.75 |
| 2868 | 3FPNB | 94 | 1.8 |
| 2869 | 3FQ4A | 109 | 1.49 |
| 2870 | 3FQGA | 313 | 2 |
| 2871 | 3FRHA | 242 | 1.2 |
| 2872 | 3FSAA | 122 | 0.98 |
| 2873 | 3FT7B | 46 | 2 |
| 2874 | 3FTDA | 240 | 1.44 |
| 2875 | 3FTTA | 188 | 1.6 |
| 2876 | 3FUYC | 156 | 2 |
| 2877 | 3FVVA | 223 | 2.1 |
| 2878 | 3FWBB | 54 | 2.5 |
| 2879 | 3FWKA | 291 | 1.2 |
| 2880 | 3FX7B | 87 | 1.65 |
| 2881 | 3FXHA | 113 | 1.84 |
| 2882 | 3FY5B | 85 | 2.4 |
| 2883 | 3FYFA | 149 | 2.2 |
| 2884 | 3FYMA | 82 | 1 |
| 2885 | 3FYNA | 152 | 1.45 |
| 2886 | 3FYQA | 179 | 1.95 |
| 2887 | 3FZ4A | 119 | 1.38 |
| 2888 | 3FZWA | 127 | 1.32 |
| 2889 | 3G0KA | 129 | 1.3 |
| 2890 | 3G14A | 176 | 1.75 |
| 2891 | 3G15B | 306 | 1.7 |
| 2892 | 3G16B | 152 | 1.45 |
| 2893 | 3G1JB | 87 | 1.7 |
| 2894 | 3G27A | 81 | 2.1 |
| 2895 | 3G2BA | 90 | 1.66 |
| 2896 | 3G36D | 51 | 1.2 |
| 2897 | 3G3BG | 71 | 2.4 |
| 2898 | 3G3KB | 255 | 1.24 |
| 2899 | 3G3LA | 291 | 2.2 |
| 2900 | 3G3ZA | 142 | 2.1 |
| 2901 | 3G43F | 45 | 2.1 |
| 2902 | 3G46A | 146 | 0.91 |
| 2903 | 3G48A | 110 | 1.5 |
| 2904 | 3G5OB | 81 | 2 |
| 2905 | 3G5TA | 299 | 1.12 |
| 2906 | 3G62A | 375 | 0.98 |
| 2907 | 3G73A | 90 | 2.21 |
| 2908 | 3G74H | 85 | 2.43 |
| 2909 | 3G7PA | 145 | 2 |
| 2910 | 3G7RB | 202 | 1.38 |
| 2911 | 3G8KA | 127 | 2 |
| 2912 | 3G8ZA | 129 | 1.9 |
| 2913 | 3G91A | 260 | 1.23 |
| 2914 | 3G9KS | 166 | 1.79 |
| 2915 | 3G9OB | 75 | 1.65 |
| 2916 | 3G9YA | 29 | 1.4 |
| 2917 | 3GA3A | 133 | 1.45 |
| 2918 | 3GA4A | 156 | 1.3 |
| 2919 | 3GA7A | 308 | 1.55 |
| 2920 | 3GA8A | 67 | 1.7 |
| 2921 | 3GAEB | 253 | 1.6 |
| 2922 | 3GBWA | 161 | 1.32 |
| 2923 | 3GCGB | 152 | 2.3 |
| 2924 | 3GDCC | 286 | 1.8 |
| 2925 | 3GE2A | 89 | 2.2 |
| 2926 | 3GE3B | 304 | 1.52 |
| 2927 | 3GE3C | 83 | 1.52 |
| 2928 | 3GE3E | 102 | 1.52 |
| 2929 | 3GE6B | 210 | 1.85 |
| 2930 | 3GFFB | 316 | 2.12 |
| 2931 | 3GFPA | 180 | 1.8 |
| 2932 | 3GGYA | 186 | 1.7 |
| 2933 | 3GI7A | 103 | 1.85 |
| 2934 | 3GIUA | 212 | 1.25 |
| 2935 | 3GIXA | 141 | 1.33 |
| 2936 | 3GIYA | 353 | 1.6 |
| 2937 | 3GJ3B | 27 | 1.79 |
| 2938 | 3GJ4D | 25 | 2.15 |
| 2939 | 3GJUA | 458 | 1.55 |
| 2940 | 3GK5A | 100 | 2.4 |
| 2941 | 3GKMA | 157 | 1.53 |
| 2942 | 3GL6A | 52 | 1.9 |
| 2943 | 3GLAA | 97 | 1.64 |
| 2944 | 3GM5A | 151 | 2 |
| 2945 | 3GMIA | 356 | 1.91 |
| 2946 | 3GMOA | 268 | 1.6 |
| 2947 | 3GMXA | 154 | 1.05 |
| 2948 | 3GNEB | 242 | 1.2 |
| 2949 | 3GNJA | 111 | 1.99 |
| 2950 | 3GNZP | 211 | 1.35 |
| 2951 | 3GODD | 299 | 2.17 |
| 2952 | 3GOEA | 80 | 0.97 |
| 2953 | 3GP6A | 155 | 1.4 |
| 2954 | 3GPQD | 148 | 2 |
| 2955 | 3GQHA | 163 | 1.8 |
| 2956 | 3GQQF | 164 | 1.95 |
| 2957 | 3GR0A | 186 | 2.3 |
| 2958 | 3GRDB | 132 | 1.25 |
| 2959 | 3GREA | 408 | 1.8 |
| 2960 | 3GS2B | 30 | 1.7 |
| 2961 | 3GSZB | 558 | 1.9 |
| 2962 | 3GTZC | 115 | 2.5 |
| 2963 | 3GV3A | 62 | 1.6 |
| 2964 | 3GVOA | 342 | 1.6 |
| 2965 | 3GWKC | 98 | 1.3 |
| 2966 | 3GWLB | 102 | 2.1 |
| 2967 | 3GWNA | 113 | 1.78 |
| 2968 | 3GWRA | 126 | 2.01 |
| 2969 | 3GX8A | 111 | 1.67 |
| 2970 | 3GXBB | 177 | 1.9 |
| 2971 | 3GXHB | 151 | 1.4 |
| 2972 | 3GXRA | 185 | 1.7 |
| 2973 | 3GXVD | 26 | 2.2 |
| 2974 | 3GZ7A | 99 | 2.15 |
| 2975 | 3GZED | 182 | 1.98 |
| 2976 | 3GZRB | 142 | 1.4 |
| 2977 | 3GZYB | 186 | 1.62 |
| 2978 | 3H05B | 163 | 1.65 |
| 2979 | 3H09B | 964 | 1.75 |
| 2980 | 3H0DB | 151 | 2.4 |
| 2981 | 3H11A | 212 | 1.9 |
| 2982 | 3H16D | 130 | 2.5 |
| 2983 | 3H2DB | 154 | 1.86 |
| 2984 | 3H31A | 74 | 1 |
| 2985 | 3H36A | 78 | 1.8 |
| 2986 | 3H3HA | 118 | 1.6 |
| 2987 | 3H3LA | 232 | 1.59 |
| 2988 | 3H3MB | 82 | 2.5 |
| 2989 | 3H43A | 76 | 2.1 |
| 2990 | 3H4TA | 390 | 1.15 |
| 2991 | 3H4XA | 315 | 1.23 |
| 2992 | 3H5JB | 167 | 1.2 |
| 2993 | 3H5ZA | 411 | 1.49 |
| 2994 | 3H6NA | 104 | 2 |
| 2995 | 3H6PA | 60 | 1.91 |
| 2996 | 3H6PC | 56 | 1.91 |
| 2997 | 3H79A | 115 | 1.5 |
| 2998 | 3H7HA | 118 | 1.55 |
| 2999 | 3H7HB | 95 | 1.55 |
| 3000 | 3H7IA | 302 | 1.5 |
| 3001 | 3H87A | 136 | 1.49 |
| 3002 | 3H87C | 72 | 1.49 |
| 3003 | 3H8AF | 25 | 1.9 |
| 3004 | 3H8DD | 119 | 2.2 |
| 3005 | 3H8DH | 37 | 2.2 |
| 3006 | 3H8KB | 28 | 1.8 |
| 3007 | 3H8TA | 182 | 1.8 |
| 3008 | 3H8UB | 115 | 1.8 |
| 3009 | 3H8ZA | 112 | 1.92 |
| 3010 | 3H96B | 137 | 2 |
| 3011 | 3H9GA | 346 | 2.2 |
| 3012 | 3H9MA | 399 | 1.57 |
| 3013 | 3H9WA | 109 | 1.9 |
| 3014 | 3HA2A | 168 | 1.8 |
| 3015 | 3HA4F | 129 | 2.4 |
| 3016 | 3HCTA | 104 | 2.1 |
| 3017 | 3HCWA | 283 | 2.2 |
| 3018 | 3HD5C | 181 | 2.35 |
| 3019 | 3HE1C | 147 | 2.1 |
| 3020 | 3HE4B | 44 | 2.46 |
| 3021 | 3HE5A | 47 | 1.75 |
| 3022 | 3HF5A | 115 | 1.4 |
| 3023 | 3HFEA | 27 | 1.7 |
| 3024 | 3HFOC | 66 | 1.3 |
| 3025 | 3HHTA | 202 | 1.16 |
| 3026 | 3HHTB | 227 | 1.16 |
| 3027 | 3HIAB | 83 | 2.38 |
| 3028 | 3HIEB | 163 | 2 |
| 3029 | 3HILA | 63 | 2 |
| 3030 | 3HIMA | 190 | 2.2 |
| 3031 | 3HINA | 258 | 2 |
| 3032 | 3HKLB | 153 | 2.1 |
| 3033 | 3HLXA | 254 | 1.3 |
| 3034 | 3HM2H | 167 | 2.21 |
| 3035 | 3HMSA | 91 | 1.7 |
| 3036 | 3HMZA | 191 | 1.5 |
| 3037 | 3HNAB | 260 | 1.5 |
| 3038 | 3HNXA | 108 | 1.37 |
| 3039 | 3HP4A | 183 | 1.35 |
| 3040 | 3HPCX | 155 | 1.47 |
| 3041 | 3HPWA | 101 | 1.45 |
| 3042 | 3HPWC | 33 | 1.45 |
| 3043 | 3HQCA | 140 | 1.8 |
| 3044 | 3HQXA | 105 | 1.66 |
| 3045 | 3HRGA | 257 | 1.85 |
| 3046 | 3HROA | 37 | 1.9 |
| 3047 | 3HRQB | 319 | 1.8 |
| 3048 | 3HRYA | 67 | 2.25 |
| 3049 | 3HT1A | 142 | 1.2 |
| 3050 | 3HTKB | 73 | 2.31 |
| 3051 | 3HTKC | 254 | 2.31 |
| 3052 | 3HTMD | 152 | 2.5 |
| 3053 | 3HTNA | 143 | 1.5 |
| 3054 | 3HTUH | 36 | 2 |
| 3055 | 3HTYP | 93 | 1.95 |
| 3056 | 3HUGD | 62 | 2.35 |
| 3057 | 3HULB | 269 | 2.19 |
| 3058 | 3HVZB | 64 | 2.2 |
| 3059 | 3HWUA | 144 | 1.3 |
| 3060 | 3HXIA | 182 | 1.8 |
| 3061 | 3HXWA | 441 | 1.93 |
| 3062 | 3HYNA | 186 | 1.2 |
| 3063 | 3HZBH | 87 | 1.74 |
| 3064 | 3HZEA | 107 | 2 |
| 3065 | 3HZSA | 209 | 2.1 |
| 3066 | 3I10A | 278 | 1.35 |
| 3067 | 3I1AB | 329 | 1.7 |
| 3068 | 3I26B | 364 | 1.8 |
| 3069 | 3I2VA | 124 | 1.25 |
| 3070 | 3I38J | 101 | 2.3 |
| 3071 | 3I4OB | 67 | 1.47 |
| 3072 | 3I4PA | 153 | 2.3 |
| 3073 | 3I5RA | 81 | 1.7 |
| 3074 | 3I5WA | 32 | 1.63 |
| 3075 | 3I6XD | 164 | 2.5 |
| 3076 | 3I7DA | 157 | 2.3 |
| 3077 | 3I7MA | 134 | 1.46 |
| 3078 | 3I84A | 79 | 2 |
| 3079 | 3I94A | 243 | 1.04 |
| 3080 | 3IARA | 360 | 1.52 |
| 3081 | 3IB7A | 295 | 1.6 |
| 3082 | 3IBWA | 79 | 1.93 |
| 3083 | 3IC3A | 98 | 1.8 |
| 3084 | 3IEZB | 95 | 1.5 |
| 3085 | 3IF4A | 100 | 2.18 |
| 3086 | 3IFWA | 223 | 2.4 |
| 3087 | 3IG2B | 197 | 2.09 |
| 3088 | 3IGMB | 62 | 2.2 |
| 3089 | 3IGRB | 183 | 2 |
| 3090 | 3IGSB | 232 | 1.5 |
| 3091 | 3IHSB | 84 | 1.15 |
| 3092 | 3IHXB | 129 | 2.5 |
| 3093 | 3IISM | 151 | 1.4 |
| 3094 | 3IJDB | 285 | 2 |
| 3095 | 3IJMA | 146 | 1.7 |
| 3096 | 3IJWB | 264 | 1.9 |
| 3097 | 3IKKA | 125 | 2.5 |
| 3098 | 3ILYA | 142 | 2.2 |
| 3099 | 3IM3A | 50 | 2 |
| 3100 | 3IM4C | 27 | 2.29 |
| 3101 | 3IMAD | 85 | 2.03 |
| 3102 | 3IMHA | 332 | 1.76 |
| 3103 | 3IMMA | 197 | 2 |
| 3104 | 3INGA | 319 | 1.95 |
| 3105 | 3IOFA | 227 | 1.44 |
| 3106 | 3IOLA | 100 | 2.1 |
| 3107 | 3IP0A | 158 | 0.89 |
| 3108 | 3IPFB | 65 | 1.99 |
| 3109 | 3IQ2A | 123 | 1.7 |
| 3110 | 3IQUA | 236 | 1.05 |
| 3111 | 3IRBA | 137 | 1.8 |
| 3112 | 3IRSA | 281 | 1.76 |
| 3113 | 3IS6B | 217 | 1.95 |
| 3114 | 3ISXA | 335 | 1.4 |
| 3115 | 3IT7B | 182 | 2.14 |
| 3116 | 3IU0A | 354 | 1.9 |
| 3117 | 3IU5A | 114 | 1.63 |
| 3118 | 3IU6A | 143 | 1.79 |
| 3119 | 3IUFA | 32 | 1.8 |
| 3120 | 3IUWA | 79 | 1.58 |
| 3121 | 3IVEA | 495 | 1.7 |
| 3122 | 3IVVA | 140 | 1.25 |
| 3123 | 3IWFA | 89 | 1.4 |
| 3124 | 3IX0A | 87 | 2.3 |
| 3125 | 3IXSG | 99 | 1.7 |
| 3126 | 3IXSL | 32 | 1.7 |
| 3127 | 3JQLA | 119 | 1.2 |
| 3128 | 3JRNA | 151 | 2 |
| 3129 | 3JRVA | 142 | 1.6 |
| 3130 | 3JSTB | 94 | 2.1 |
| 3131 | 3JSYA | 213 | 1.6 |
| 3132 | 3JTWA | 176 | 1.9 |
| 3133 | 3JU4A | 670 | 0.98 |
| 3134 | 3JUDA | 144 | 0.98 |
| 3135 | 3JVDA | 255 | 2.3 |
| 3136 | 3JVMA | 116 | 1.2 |
| 3137 | 3JVOM | 96 | 2.1 |
| 3138 | 3JWIA | 195 | 2.2 |
| 3139 | 3JXOA | 84 | 1.55 |
| 3140 | 3JYBB | 138 | 2.04 |
| 3141 | 3JZ0A | 266 | 2 |
| 3142 | 3K06B | 95 | 1.58 |
| 3143 | 3K0BA | 380 | 1.5 |
| 3144 | 3K0LB | 140 | 2.35 |
| 3145 | 3K0XA | 99 | 1.7 |
| 3146 | 3K12A | 115 | 1.49 |
| 3147 | 3K1EA | 124 | 1.85 |
| 3148 | 3K1RB | 74 | 2.3 |
| 3149 | 3K1SI | 104 | 2.3 |
| 3150 | 3K1WB | 337 | 1.5 |
| 3151 | 3K2AA | 55 | 1.95 |
| 3152 | 3K2VA | 130 | 1.95 |
| 3153 | 3K3CB | 152 | 1.62 |
| 3154 | 3K4IA | 202 | 1.69 |
| 3155 | 3K5IB | 376 | 2 |
| 3156 | 3K65A | 80 | 1.85 |
| 3157 | 3K67B | 156 | 1.25 |
| 3158 | 3K6CJ | 90 | 2.2 |
| 3159 | 3K6GC | 93 | 1.95 |
| 3160 | 3K6GE | 36 | 1.95 |
| 3161 | 3K6IA | 99 | 1.13 |
| 3162 | 3K6TD | 46 | 2.04 |
| 3163 | 3K7CD | 108 | 2 |
| 3164 | 3K7IB | 157 | 1.44 |
| 3165 | 3K94A | 215 | 2.1 |
| 3166 | 3KA5B | 59 | 1.8 |
| 3167 | 3KA8A | 172 | 1.35 |
| 3168 | 3KAEA | 225 | 2.3 |
| 3169 | 3KALA | 470 | 1.9 |
| 3170 | 3KANC | 117 | 1.13 |
| 3171 | 3KB2B | 167 | 2.2 |
| 3172 | 3KB5A | 193 | 1.5 |
| 3173 | 3KBRA | 231 | 1.66 |
| 3174 | 3KBYA | 145 | 1.8 |
| 3175 | 3KC2B | 319 | 1.55 |
| 3176 | 3KCCB | 198 | 1.66 |
| 3177 | 3KCUC | 239 | 2.24 |
| 3178 | 3KDEC | 74 | 1.74 |
| 3179 | 3KDFC | 118 | 1.98 |
| 3180 | 3KE7B | 133 | 1.45 |
| 3181 | 3KEPB | 148 | 1.82 |
| 3182 | 3KEVA | 184 | 1.3 |
| 3183 | 3KF6A | 136 | 1.65 |
| 3184 | 3KF8D | 123 | 2.4 |
| 3185 | 3KFFA | 152 | 0.96 |
| 3186 | 3KG4A | 184 | 1.95 |
| 3187 | 3KGKA | 92 | 1.4 |
| 3188 | 3KGRA | 99 | 1.8 |
| 3189 | 3KGZB | 145 | 1.85 |
| 3190 | 3KH0B | 105 | 2.1 |
| 3191 | 3KH1B | 189 | 1.37 |
| 3192 | 3KHFB | 97 | 1.2 |
| 3193 | 3KIKE | 27 | 2.1 |
| 3194 | 3KIZB | 371 | 1.5 |
| 3195 | 3KK4D | 125 | 1.95 |
| 3196 | 3KKFA | 105 | 1.3 |
| 3197 | 3KKZB | 250 | 1.68 |
| 3198 | 3KL2L | 200 | 2.3 |
| 3199 | 3KLQB | 130 | 1.9 |
| 3200 | 3KLRA | 125 | 0.88 |
| 3201 | 3KMHB | 223 | 1.58 |
| 3202 | 3KNBA | 98 | 1.4 |
| 3203 | 3KNVA | 123 | 1.9 |
| 3204 | 3KOJB | 90 | 1.9 |
| 3205 | 3KOPE | 165 | 1.9 |
| 3206 | 3KOSA | 200 | 1.83 |
| 3207 | 3KP1F | 109 | 2.01 |
| 3208 | 3KP7B | 142 | 2.3 |
| 3209 | 3KPEA | 49 | 1.47 |
| 3210 | 3KPEB | 35 | 1.47 |
| 3211 | 3KT7A | 558 | 1.77 |
| 3212 | 3KT9A | 102 | 1.65 |
| 3213 | 3KTAB | 171 | 1.63 |
| 3214 | 3KTBD | 104 | 2.1 |
| 3215 | 3KU3B | 172 | 1.6 |
| 3216 | 3KUCB | 76 | 1.92 |
| 3217 | 3KUPC | 59 | 1.77 |
| 3218 | 3KUUC | 167 | 1.41 |
| 3219 | 3KUVB | 133 | 1.5 |
| 3220 | 3KV1A | 253 | 1.7 |
| 3221 | 3KVPB | 43 | 2.4 |
| 3222 | 3KW8A | 273 | 2.29 |
| 3223 | 3KWOD | 148 | 1.99 |
| 3224 | 3KWSB | 265 | 1.68 |
| 3225 | 3KWUA | 135 | 1.37 |
| 3226 | 3KX6D | 343 | 2.1 |
| 3227 | 3KXTA | 56 | 1.6 |
| 3228 | 3KYJA | 129 | 1.4 |
| 3229 | 3KYZA | 112 | 1.5 |
| 3230 | 3KZ5E | 48 | 1.58 |
| 3231 | 3KZ9D | 199 | 2.1 |
| 3232 | 3KZPB | 232 | 2 |
| 3233 | 3KZTA | 132 | 2.1 |
| 3234 | 3KZXA | 205 | 1.9 |
| 3235 | 3L0AA | 266 | 2.19 |
| 3236 | 3L0FA | 162 | 1.35 |
| 3237 | 3L0VA | 255 | 1.75 |
| 3238 | 3L11A | 104 | 2.12 |
| 3239 | 3L1NA | 155 | 1.3 |
| 3240 | 3L2AA | 126 | 1.71 |
| 3241 | 3L2HA | 158 | 1.85 |
| 3242 | 3L32A | 44 | 1.5 |
| 3243 | 3L41A | 214 | 1.45 |
| 3244 | 3L46A | 90 | 1.48 |
| 3245 | 3L4AA | 121 | 1.5 |
| 3246 | 3L4HA | 109 | 1.8 |
| 3247 | 3L4QD | 163 | 2.3 |
| 3248 | 3L4RA | 151 | 1.45 |
| 3249 | 3L5WJ | 86 | 2 |
| 3250 | 3L77A | 235 | 1.6 |
| 3251 | 3L80A | 273 | 2 |
| 3252 | 3L8WA | 295 | 1 |
| 3253 | 3L9AX | 81 | 1.3 |
| 3254 | 3LA7B | 205 | 1.9 |
| 3255 | 3LAXA | 106 | 1.43 |
| 3256 | 3LB2B | 137 | 1.06 |
| 3257 | 3LBEA | 122 | 1.7 |
| 3258 | 3LCNC | 25 | 2 |
| 3259 | 3LCZA | 53 | 2.06 |
| 3260 | 3LD7A | 87 | 1.55 |
| 3261 | 3LDDA | 82 | 1.45 |
| 3262 | 3LE0A | 142 | 1.91 |
| 3263 | 3LE4A | 55 | 1.7 |
| 3264 | 3LEDB | 375 | 1.45 |
| 3265 | 3LF5B | 87 | 1.25 |
| 3266 | 3LFJB | 166 | 1.56 |
| 3267 | 3LFPA | 96 | 2 |
| 3268 | 3LGBB | 193 | 1.54 |
| 3269 | 3LGIC | 215 | 1.65 |
| 3270 | 3LHIA | 231 | 1.33 |
| 3271 | 3LHKA | 146 | 2.2 |
| 3272 | 3LHOA | 254 | 1.8 |
| 3273 | 3LHQA | 211 | 1.56 |
| 3274 | 3LJDB | 126 | 1.38 |
| 3275 | 3LJKA | 543 | 1.48 |
| 3276 | 3LJMA | 29 | 1.36 |
| 3277 | 3LJWB | 120 | 1.5 |
| 3278 | 3LK49 | 29 | 1.99 |
| 3279 | 3LKKA | 232 | 2 |
| 3280 | 3LKMA | 247 | 1.6 |
| 3281 | 3LKXB | 54 | 2.5 |
| 3282 | 3LL6B | 271 | 2.1 |
| 3283 | 3LL8A | 357 | 2 |
| 3284 | 3LLPA | 472 | 1.8 |
| 3285 | 3LLRD | 143 | 2.3 |
| 3286 | 3LLTA | 341 | 2.5 |
| 3287 | 3LLUA | 177 | 1.4 |
| 3288 | 3LMBA | 164 | 2.1 |
| 3289 | 3LNLA | 307 | 2 |
| 3290 | 3LO3Q | 94 | 2.38 |
| 3291 | 3LO8A | 302 | 1.05 |
| 3292 | 3LOFA | 84 | 2.4 |
| 3293 | 3LPHD | 57 | 2.5 |
| 3294 | 3LPWB | 195 | 1.65 |
| 3295 | 3LQ9A | 128 | 2 |
| 3296 | 3LQBA | 198 | 1.1 |
| 3297 | 3LQKA | 195 | 2.1 |
| 3298 | 3LQWA | 129 | 1.3 |
| 3299 | 3LR2B | 129 | 1.7 |
| 3300 | 3LRQD | 84 | 2.29 |
| 3301 | 3LRTA | 284 | 1.53 |
| 3302 | 3LRUA | 160 | 1.85 |
| 3303 | 3LU9F | 25 | 1.8 |
| 3304 | 3LUMD | 262 | 1.7 |
| 3305 | 3LUUA | 89 | 1.93 |
| 3306 | 3LVKB | 76 | 2.44 |
| 3307 | 3LVYD | 179 | 2.1 |
| 3308 | 3LW6A | 241 | 1.81 |
| 3309 | 3LWCA | 104 | 1.4 |
| 3310 | 3LWXA | 199 | 1.1 |
| 3311 | 3LX3A | 163 | 1.55 |
| 3312 | 3LXRF | 181 | 1.68 |
| 3313 | 3LYEA | 285 | 1.3 |
| 3314 | 3LYGA | 120 | 1.61 |
| 3315 | 3LYHB | 122 | 1.6 |
| 3316 | 3LYIA | 120 | 2.1 |
| 3317 | 3LYUF | 131 | 2.3 |
| 3318 | 3M0FA | 203 | 1.6 |
| 3319 | 3M0ZA | 248 | 1.2 |
| 3320 | 3M1EA | 87 | 1.8 |
| 3321 | 3M1IB | 131 | 2 |
| 3322 | 3M1XA | 126 | 1.2 |
| 3323 | 3M3GA | 120 | 1.39 |
| 3324 | 3M3PA | 249 | 1.3 |
| 3325 | 3M4WG | 36 | 2.3 |
| 3326 | 3M5QA | 357 | 0.93 |
| 3327 | 3M73A | 313 | 1.15 |
| 3328 | 3M7AB | 140 | 1.22 |
| 3329 | 3M7KA | 142 | 1.92 |
| 3330 | 3M7OA | 137 | 1.65 |
| 3331 | 3M7PA | 301 | 2.5 |
| 3332 | 3M7VB | 401 | 2 |
| 3333 | 3M8JA | 90 | 1.4 |
| 3334 | 3M91B | 31 | 1.8 |
| 3335 | 3M9JA | 105 | 1.1 |
| 3336 | 3M9QA | 89 | 1.29 |
| 3337 | 3M9ZA | 124 | 1.7 |
| 3338 | 3MA2B | 117 | 2.05 |
| 3339 | 3MB2F | 54 | 2.41 |
| 3340 | 3MBKB | 264 | 1.35 |
| 3341 | 3MBRX | 236 | 1.44 |
| 3342 | 3MCBB | 58 | 1.9 |
| 3343 | 3MCIC | 175 | 1.7 |
| 3344 | 3MD1B | 79 | 1.6 |
| 3345 | 3MD7A | 270 | 1.27 |
| 3346 | 3MD9A | 253 | 1.5 |
| 3347 | 3MDPA | 132 | 1.9 |
| 3348 | 3MDYA | 320 | 2.05 |
| 3349 | 3ME5A | 414 | 1.75 |
| 3350 | 3MEAA | 162 | 1.26 |
| 3351 | 3MEZA | 111 | 1.94 |
| 3352 | 3MFXC | 112 | 2.4 |
| 3353 | 3MGDA | 153 | 1.9 |
| 3354 | 3MH9C | 205 | 1.79 |
| 3355 | 3MHPC | 26 | 1.7 |
| 3356 | 3MI9C | 49 | 2.1 |
| 3357 | 3MJ0A | 118 | 2.31 |
| 3358 | 3MJGA | 97 | 2.3 |
| 3359 | 3MJHB | 34 | 2.03 |
| 3360 | 3MJOA | 296 | 1.36 |
| 3361 | 3MK1A | 481 | 1.57 |
| 3362 | 3MK6C | 351 | 1.98 |
| 3363 | 3MKOA | 103 | 1.8 |
| 3364 | 3ML1B | 109 | 1.6 |
| 3365 | 3MM1A | 439 | 1.42 |
| 3366 | 3MM5B | 363 | 1.8 |
| 3367 | 3MMHA | 167 | 1.25 |
| 3368 | 3MMYB | 51 | 1.65 |
| 3369 | 3MN2B | 108 | 1.8 |
| 3370 | 3MNLA | 188 | 1.8 |
| 3371 | 3MNMA | 112 | 1.73 |
| 3372 | 3MOEA | 618 | 1.25 |
| 3373 | 3MOLB | 174 | 1.2 |
| 3374 | 3MPCA | 96 | 1.6 |
| 3375 | 3MQ2A | 215 | 1.69 |
| 3376 | 3MQDA | 407 | 1.25 |
| 3377 | 3MQHB | 189 | 1.43 |
| 3378 | 3MQPB | 25 | 2.24 |
| 3379 | 3MSEB | 168 | 2.1 |
| 3380 | 3MSTA | 233 | 1.35 |
| 3381 | 3MSWA | 139 | 1.9 |
| 3382 | 3MSXB | 192 | 1.65 |
| 3383 | 3MTSC | 58 | 2.2 |
| 3384 | 3MTUF | 67 | 2.1 |
| 3385 | 3MUDD | 48 | 2.2 |
| 3386 | 3MUJB | 133 | 1.92 |
| 3387 | 3MVCB | 154 | 1.4 |
| 3388 | 3MVSA | 210 | 1.1 |
| 3389 | 3MW4A | 178 | 2 |
| 3390 | 3MW6B | 94 | 2.21 |
| 3391 | 3MWZA | 115 | 1.52 |
| 3392 | 3MX7A | 90 | 1.76 |
| 3393 | 3MXOA | 188 | 1.7 |
| 3394 | 3MYBC | 259 | 1.55 |
| 3395 | 3MYUB | 334 | 1.95 |
| 3396 | 3MZ0A | 337 | 1.54 |
| 3397 | 3N0RA | 258 | 1.25 |
| 3398 | 3N0UA | 209 | 1.5 |
| 3399 | 3N1FC | 94 | 1.6 |
| 3400 | 3N3FB | 53 | 2 |
| 3401 | 3N3MA | 328 | 1.47 |
| 3402 | 3N44A | 54 | 2.35 |
| 3403 | 3N4JA | 161 | 1.47 |
| 3404 | 3N53B | 93 | 2.2 |
| 3405 | 3N5BB | 86 | 1.9 |
| 3406 | 3N6YA | 124 | 1.5 |
| 3407 | 3N72B | 152 | 1.77 |
| 3408 | 3N79A | 183 | 1.5 |
| 3409 | 3N8BA | 75 | 1.9 |
| 3410 | 3NA3A | 302 | 2.5 |
| 3411 | 3NBCB | 148 | 1.01 |
| 3412 | 3NBMA | 104 | 1.3 |
| 3413 | 3NCEA | 186 | 2 |
| 3414 | 3NCEB | 206 | 2 |
| 3415 | 3NDDB | 35 | 1.5 |
| 3416 | 3NDHB | 208 | 1.3 |
| 3417 | 3NDIA | 405 | 1.5 |
| 3418 | 3NE0A | 208 | 1 |
| 3419 | 3NE8A | 226 | 1.24 |
| 3420 | 3NEDA | 228 | 0.95 |
| 3421 | 3NFKB | 91 | 1.43 |
| 3422 | 3NFWA | 179 | 1.6 |
| 3423 | 3NGGB | 48 | 1.33 |
| 3424 | 3NGWA | 195 | 2.31 |
| 3425 | 3NGXB | 275 | 2.3 |
| 3426 | 3NJAA | 106 | 2.37 |
| 3427 | 3NJEA | 156 | 1.85 |
| 3428 | 3NKEB | 183 | 1.4 |
| 3429 | 3NKLA | 122 | 1.9 |
| 3430 | 3NMRA | 175 | 1.85 |
| 3431 | 3NNGB | 151 | 2.18 |
| 3432 | 3NO0C | 276 | 1.3 |
| 3433 | 3NO3A | 238 | 1.89 |
| 3434 | 3NO6A | 235 | 1.65 |
| 3435 | 3NO8B | 157 | 2.2 |
| 3436 | 3NPDA | 113 | 1.6 |
| 3437 | 3NPFB | 297 | 1.72 |
| 3438 | 3NPHB | 131 | 1.85 |
| 3439 | 3NPKA | 265 | 1.5 |
| 3440 | 3NQAB | 218 | 1.4 |
| 3441 | 3NREA | 291 | 1.59 |
| 3442 | 3NRHA | 158 | 1.8 |
| 3443 | 3NRVD | 142 | 2 |
| 3444 | 3NS6A | 99 | 1.25 |
| 3445 | 3NSUA | 109 | 2 |
| 3446 | 3NSWG | 94 | 1.75 |
| 3447 | 3NTVA | 211 | 1.55 |
| 3448 | 3NUAB | 237 | 1.4 |
| 3449 | 3NUFB | 112 | 1.38 |
| 3450 | 3NULA | 130 | 1.6 |
| 3451 | 3NUQA | 268 | 1.7 |
| 3452 | 3NV0A | 196 | 1.84 |
| 3453 | 3NVSA | 426 | 1.02 |
| 3454 | 3NVWA | 164 | 1.6 |
| 3455 | 3NW4A | 363 | 2 |
| 3456 | 3NY3A | 70 | 1.6 |
| 3457 | 3NYCA | 382 | 1.06 |
| 3458 | 3NZLA | 73 | 1.2 |
| 3459 | 3NZNB | 103 | 1.1 |
| 3460 | 3O0AB | 214 | 1.77 |
| 3461 | 3O0LA | 109 | 1.81 |
| 3462 | 3O0PA | 194 | 1.3 |
| 3463 | 3O12A | 185 | 1.5 |
| 3464 | 3O1CA | 115 | 1.08 |
| 3465 | 3O1NB | 246 | 1.03 |
| 3466 | 3O22A | 157 | 1.4 |
| 3467 | 3O2EA | 86 | 1.95 |
| 3468 | 3O2IC | 93 | 2.2 |
| 3469 | 3O2QE | 192 | 2.4 |
| 3470 | 3O2RB | 143 | 1.25 |
| 3471 | 3O2TA | 304 | 1.4 |
| 3472 | 3O3MA | 399 | 1.82 |
| 3473 | 3O3XA | 198 | 1.45 |
| 3474 | 3O46A | 84 | 1.3 |
| 3475 | 3O48A | 129 | 1.75 |
| 3476 | 3O4PA | 314 | 0.85 |
| 3477 | 3O5NG | 95 | 1.83 |
| 3478 | 3O5UB | 256 | 2.35 |
| 3479 | 3O5YA | 142 | 2.45 |
| 3480 | 3O6QD | 44 | 2.5 |
| 3481 | 3O79A | 97 | 1.6 |
| 3482 | 3O7AA | 52 | 1.67 |
| 3483 | 3O7IB | 147 | 1.5 |
| 3484 | 3O8QA | 270 | 1.45 |
| 3485 | 3OA2D | 298 | 1.5 |
| 3486 | 3OABC | 242 | 2.3 |
| 3487 | 3OAJB | 310 | 1.4 |
| 3488 | 3OAKC | 31 | 2.15 |
| 3489 | 3OBHB | 67 | 1.89 |
| 3490 | 3OBLA | 132 | 1.2 |
| 3491 | 3OBQA | 141 | 1.4 |
| 3492 | 3OD3A | 488 | 1.1 |
| 3493 | 3OD8H | 88 | 2.4 |
| 3494 | 3ODTB | 296 | 1.35 |
| 3495 | 3ODVA | 38 | 0.95 |
| 3496 | 3OENA | 251 | 1.8 |
| 3497 | 3OF4C | 208 | 1.9 |
| 3498 | 3OFGA | 91 | 1.37 |
| 3499 | 3OG4A | 140 | 2.16 |
| 3500 | 3OGHB | 151 | 1.65 |
| 3501 | 3OHEA | 137 | 1.2 |
| 3502 | 3OIGA | 260 | 1.25 |
| 3503 | 3OIIA | 216 | 1.85 |
| 3504 | 3OIOA | 112 | 1.65 |
| 3505 | 3OIPA | 197 | 2.5 |
| 3506 | 3OIQB | 30 | 2.4 |
| 3507 | 3OISD | 268 | 1.65 |
| 3508 | 3OIZA | 92 | 1.65 |
| 3509 | 3OJ0A | 138 | 1.65 |
| 3510 | 3OJND | 359 | 1.65 |
| 3511 | 3OK8A | 219 | 2.25 |
| 3512 | 3OKFB | 352 | 2.5 |
| 3513 | 3OKXA | 156 | 1.8 |
| 3514 | 3OLJA | 286 | 2.1 |
| 3515 | 3OLOA | 111 | 2.09 |
| 3516 | 3OLQA | 306 | 1.82 |
| 3517 | 3OMDA | 145 | 1.5 |
| 3518 | 3OMTB | 67 | 1.65 |
| 3519 | 3OMYA | 51 | 1.3 |
| 3520 | 3ON9B | 160 | 1.57 |
| 3521 | 3ONHA | 113 | 1.6 |
| 3522 | 3ONRA | 71 | 1.8 |
| 3523 | 3OOPA | 139 | 1.78 |
| 3524 | 3OOUA | 105 | 1.57 |
| 3525 | 3OP6B | 141 | 2 |
| 3526 | 3OP8A | 85 | 1.9 |
| 3527 | 3OQ2A | 99 | 1.35 |
| 3528 | 3OQIA | 222 | 1.7 |
| 3529 | 3ORKA | 274 | 1.6 |
| 3530 | 3ORUA | 231 | 1.11 |
| 3531 | 3OS4B | 365 | 1.6 |
| 3532 | 3OSEA | 100 | 1.7 |
| 3533 | 3OTMA | 205 | 1.5 |
| 3534 | 3OV5A | 84 | 1.04 |
| 3535 | 3OV8A | 91 | 1.85 |
| 3536 | 3OYVA | 354 | 1.25 |
| 3537 | 3OZYA | 386 | 1.3 |
| 3538 | 3OZZB | 82 | 1.7 |
| 3539 | 3P02A | 309 | 1.55 |
| 3540 | 3P04A | 77 | 2.2 |
| 3541 | 3P0TB | 136 | 1.9 |
| 3542 | 3P1VB | 404 | 1.93 |
| 3543 | 3P1XA | 70 | 1.9 |
| 3544 | 3P2UB | 250 | 1.48 |
| 3545 | 3P3CA | 272 | 1.25 |
| 3546 | 3P42D | 229 | 1.91 |
| 3547 | 3P4HA | 118 | 1.1 |
| 3548 | 3P57P | 105 | 2.19 |
| 3549 | 3P6BB | 186 | 2 |
| 3550 | 3P6DA | 139 | 1.06 |
| 3551 | 3P7XA | 166 | 1.96 |
| 3552 | 3P8AB | 252 | 1.95 |
| 3553 | 3P8BD | 147 | 1.8 |
| 3554 | 3P8KA | 268 | 1.7 |
| 3555 | 3P94A | 204 | 1.93 |
| 3556 | 3PA6C | 92 | 1.5 |
| 3557 | 3PA8B | 242 | 2 |
| 3558 | 3PB6X | 313 | 1.05 |
| 3559 | 3PBTA | 501 | 1.64 |
| 3560 | 3PC7A | 81 | 1.65 |
| 3561 | 3PCTC | 255 | 1.85 |
| 3562 | 3PD7B | 94 | 1.26 |
| 3563 | 3PDYB | 205 | 2.22 |
| 3564 | 3PE9D | 85 | 1.69 |
| 3565 | 3PG6D | 134 | 1.7 |
| 3566 | 3PGZB | 114 | 2.1 |
| 3567 | 3PH0C | 53 | 2.4 |
| 3568 | 3PHXB | 77 | 1.6 |
| 3569 | 3PI6A | 297 | 1.5 |
| 3570 | 3PIKA | 429 | 2.3 |
| 3571 | 3PISA | 39 | 2 |
| 3572 | 3PIWA | 154 | 1.49 |
| 3573 | 3PJ0D | 359 | 1.8 |
| 3574 | 3PJPB | 195 | 1.6 |
| 3575 | 3PKVA | 221 | 1.34 |
| 3576 | 3PKZG | 122 | 1.8 |
| 3577 | 3PL0A | 254 | 1.91 |
| 3578 | 3PLUB | 74 | 1.4 |
| 3579 | 3PLWA | 110 | 1.4 |
| 3580 | 3PMCB | 132 | 1.49 |
| 3581 | 3PMTA | 55 | 1.8 |
| 3582 | 3PN3B | 181 | 1.3 |
| 3583 | 3PNAB | 125 | 1.5 |
| 3584 | 3PNXF | 157 | 1.92 |
| 3585 | 3PP2A | 112 | 1.42 |
| 3586 | 3PP5A | 63 | 1.5 |
| 3587 | 3PPLB | 422 | 1.25 |
| 3588 | 3PPQA | 271 | 1.91 |
| 3589 | 3PQKF | 100 | 2.09 |
| 3590 | 3PQSA | 504 | 2.1 |
| 3591 | 3PR6A | 145 | 1.8 |
| 3592 | 3PROD | 152 | 1.8 |
| 3593 | 3PSMB | 47 | 0.98 |
| 3594 | 3PT3A | 88 | 1.97 |
| 3595 | 3PT5A | 320 | 1.6 |
| 3596 | 3PT8A | 151 | 1.76 |
| 3597 | 3PUCA | 98 | 0.96 |
| 3598 | 3PW3F | 359 | 2.23 |
| 3599 | 3PWKA | 357 | 1.5 |
| 3600 | 3PWTA | 562 | 1.9 |
| 3601 | 3PXLA | 499 | 1.2 |
| 3602 | 3PYIA | 142 | 2.1 |
| 3603 | 3PZ7A | 86 | 2.44 |
| 3604 | 3Q0HA | 109 | 1.7 |
| 3605 | 3Q1PB | 197 | 1.8 |
| 3606 | 3Q2BA | 120 | 1.6 |
| 3607 | 3Q39A | 315 | 1.25 |
| 3608 | 3Q46A | 178 | 0.99 |
| 3609 | 3Q49B | 132 | 1.54 |
| 3610 | 3Q62A | 170 | 1.4 |
| 3611 | 3Q63A | 132 | 2 |
| 3612 | 3Q64A | 155 | 1.5 |
| 3613 | 3Q6AH | 135 | 1.8 |
| 3614 | 3Q6XB | 241 | 1.3 |
| 3615 | 3Q72A | 138 | 1.66 |
| 3616 | 3Q7CA | 209 | 1.5 |
| 3617 | 3Q7HN | 192 | 2.5 |
| 3618 | 3Q7RB | 95 | 1.6 |
| 3619 | 3Q7ZA | 252 | 1.87 |
| 3620 | 3Q90B | 130 | 1.7 |
| 3621 | 3QA9A | 142 | 1.9 |
| 3622 | 3QAOA | 140 | 1.87 |
| 3623 | 3QAPA | 219 | 1.9 |
| 3624 | 3QB8B | 197 | 1.5 |
| 3625 | 3QBMA | 199 | 1.8 |
| 3626 | 3QC2B | 357 | 2.3 |
| 3627 | 3QEXA | 901 | 1.73 |
| 3628 | 3QFHB | 425 | 2.05 |
| 3629 | 3QFTA | 433 | 1.4 |
| 3630 | 3QHPB | 151 | 1.5 |
| 3631 | 3QI5A | 206 | 2.2 |
| 3632 | 3QITA | 281 | 1.68 |
| 3633 | 3QJGL | 170 | 2.04 |
| 3634 | 3QL9A | 125 | 0.93 |
| 3635 | 3QM9A | 145 | 0.91 |
| 3636 | 3QMDA | 53 | 1.9 |
| 3637 | 3QMXA | 99 | 1.82 |
| 3638 | 3QN1B | 295 | 1.8 |
| 3639 | 3QNAF | 118 | 2.5 |
| 3640 | 3QO4A | 163 | 2.2 |
| 3641 | 3QOOA | 135 | 1.25 |
| 3642 | 3QORB | 119 | 1.75 |
| 3643 | 3QOUA | 282 | 1.8 |
| 3644 | 3QPAA | 197 | 0.85 |
| 3645 | 3QQ8B | 80 | 2 |
| 3646 | 3QQQB | 149 | 1.84 |
| 3647 | 3QR5B | 154 | 2.3 |
| 3648 | 3QR7B | 115 | 0.94 |
| 3649 | 3QRAA | 152 | 1.8 |
| 3650 | 3QSDA | 253 | 1.3 |
| 3651 | 3QSQA | 216 | 1.8 |
| 3652 | 3QSZB | 179 | 2.39 |
| 3653 | 3QT9A | 424 | 2.05 |
| 3654 | 3QTEA | 32 | 1.95 |
| 3655 | 3QU5B | 229 | 1.24 |
| 3656 | 3QUVA | 216 | 1.7 |
| 3657 | 3QVEC | 129 | 2.04 |
| 3658 | 3QWOP | 53 | 1.9 |
| 3659 | 3QWWA | 431 | 1.8 |
| 3660 | 3QXHA | 224 | 1.36 |
| 3661 | 3QXVE | 113 | 2.5 |
| 3662 | 3QY3A | 130 | 1.75 |
| 3663 | 3QY9D | 241 | 1.8 |
| 3664 | 3QYJB | 290 | 1.78 |
| 3665 | 3QZBA | 131 | 1.1 |
| 3666 | 3QZMB | 123 | 1.25 |
| 3667 | 3QZRA | 180 | 1.04 |
| 3668 | 3QZXA | 190 | 1.3 |
| 3669 | 3R0RA | 194 | 2.35 |
| 3670 | 3R0VA | 257 | 1.38 |
| 3671 | 3R1FA | 123 | 2.5 |
| 3672 | 3R24A | 292 | 2 |
| 3673 | 3R27A | 84 | 2.04 |
| 3674 | 3R2QA | 202 | 1.05 |
| 3675 | 3R2RA | 154 | 1.65 |
| 3676 | 3R3QA | 153 | 1.45 |
| 3677 | 3R3RA | 184 | 1.2 |
| 3678 | 3R46F | 30 | 1.75 |
| 3679 | 3R5GB | 196 | 1.5 |
| 3680 | 3R5ZA | 140 | 1.5 |
| 3681 | 3R62A | 125 | 1.52 |
| 3682 | 3R6AA | 118 | 1.76 |
| 3683 | 3R84W | 73 | 2.05 |
| 3684 | 3R8JA | 180 | 1.6 |
| 3685 | 3R9FB | 180 | 1.2 |
| 3686 | 3R9ZA | 154 | 1.75 |
| 3687 | 3RAZA | 135 | 2 |
| 3688 | 3RD5A | 268 | 1.5 |
| 3689 | 3RD7A | 275 | 1.95 |
| 3690 | 3REAC | 125 | 2 |
| 3691 | 3RF7A | 362 | 2.12 |
| 3692 | 3RH3B | 237 | 2.1 |
| 3693 | 3RHBA | 100 | 1.2 |
| 3694 | 3RHEA | 114 | 2.05 |
| 3695 | 3RL5A | 279 | 1.26 |
| 3696 | 3RLGA | 278 | 1.6 |
| 3697 | 3RLSB | 144 | 1.7 |
| 3698 | 3RMHB | 133 | 1.9 |
| 3699 | 3RMIA | 105 | 2.4 |
| 3700 | 3RNQA | 112 | 1.6 |
| 3701 | 3RNQB | 184 | 1.6 |
| 3702 | 3RO3A | 159 | 1.1 |
| 3703 | 3ROBD | 128 | 1.48 |
| 3704 | 3ROFA | 158 | 1.03 |
| 3705 | 3RPCD | 262 | 1.49 |
| 3706 | 3RPDB | 341 | 1.5 |
| 3707 | 3RPFB | 146 | 1.9 |
| 3708 | 3RPFC | 74 | 1.9 |
| 3709 | 3RPPA | 216 | 1.8 |
| 3710 | 3RPWA | 333 | 1.65 |
| 3711 | 3RQ5A | 270 | 1.7 |
| 3712 | 3RQ7A | 212 | 1.55 |
| 3713 | 3RQ9B | 84 | 1 |
| 3714 | 3RQAD | 169 | 2.1 |
| 3715 | 3RQTA | 466 | 1.5 |
| 3716 | 3RQZC | 245 | 1.95 |
| 3717 | 3RSNA | 165 | 2.1 |
| 3718 | 3RT2A | 171 | 1.5 |
| 3719 | 3RTLA | 114 | 1.45 |
| 3720 | 3RWNC | 153 | 1 |
| 3721 | 3RY2B | 122 | 0.95 |
| 3722 | 3RY4A | 170 | 1.5 |
| 3723 | 3RYCB | 432 | 2.1 |
| 3724 | 3RYDA | 261 | 2.37 |
| 3725 | 3RYKB | 175 | 1.63 |
| 3726 | 3RZNA | 206 | 1.1 |
| 3727 | 3S0AA | 119 | 1.15 |
| 3728 | 3S0RB | 30 | 2.45 |
| 3729 | 3S25A | 295 | 1.88 |
| 3730 | 3S40D | 264 | 2.1 |
| 3731 | 3S46B | 367 | 2 |
| 3732 | 3S5BA | 236 | 1.8 |
| 3733 | 3S6EA | 113 | 0.95 |
| 3734 | 3S6LD | 158 | 2.3 |
| 3735 | 3S6MA | 163 | 1.65 |
| 3736 | 3S6NG | 55 | 2.5 |
| 3737 | 3S7OA | 315 | 1.24 |
| 3738 | 3S81A | 232 | 1.8 |
| 3739 | 3S83A | 256 | 1.34 |
| 3740 | 3S8GC | 31 | 1.8 |
| 3741 | 3S8KA | 183 | 1.7 |
| 3742 | 3S8SA | 107 | 1.3 |
| 3743 | 3S90D | 26 | 1.97 |
| 3744 | 3S95B | 290 | 1.65 |
| 3745 | 3S9CA | 234 | 1.8 |
| 3746 | 3S9DA | 127 | 2 |
| 3747 | 3S9JA | 362 | 1.75 |
| 3748 | 3S9XA | 159 | 1.35 |
| 3749 | 3SAOA | 150 | 1.8 |
| 3750 | 3SC0A | 237 | 1.95 |
| 3751 | 3SD2A | 86 | 1.4 |
| 3752 | 3SD4A | 69 | 1.93 |
| 3753 | 3SE2A | 186 | 2.3 |
| 3754 | 3SE8G | 344 | 1.9 |
| 3755 | 3SEBA | 238 | 1.48 |
| 3756 | 3SEEA | 214 | 1.25 |
| 3757 | 3SF6A | 387 | 1.7 |
| 3758 | 3SG0A | 361 | 1.2 |
| 3759 | 3SG8A | 298 | 1.8 |
| 3760 | 3SGGA | 512 | 1.25 |
| 3761 | 3SGRF | 25 | 2.17 |
| 3762 | 3SHGA | 198 | 1.5 |
| 3763 | 3SHGB | 61 | 1.5 |
| 3764 | 3SIBA | 203 | 1.9 |
| 3765 | 3SIQD | 97 | 2.4 |
| 3766 | 3SK2A | 132 | 1.01 |
| 3767 | 3SKJF | 84 | 2.5 |
| 3768 | 3SLZB | 114 | 1.4 |
| 3769 | 3SM4C | 229 | 1.88 |
| 3770 | 3SNKA | 119 | 2.02 |
| 3771 | 3SO0E | 86 | 1.93 |
| 3772 | 3SOJB | 115 | 1 |
| 3773 | 3SOKB | 142 | 2.3 |
| 3774 | 3SOVA | 306 | 1.27 |
| 3775 | 3SOYA | 142 | 2 |
| 3776 | 3SQFA | 95 | 1.63 |
| 3777 | 3SQNB | 381 | 2.31 |
| 3778 | 3SSBC | 35 | 1.8 |
| 3779 | 3SSBI | 30 | 1.8 |
| 3780 | 3SWFC | 51 | 2.14 |
| 3781 | 3SWNA | 76 | 2.5 |
| 3782 | 3SWNB | 71 | 2.5 |
| 3783 | 3SWNF | 78 | 2.5 |
| 3784 | 3SX2H | 249 | 1.5 |
| 3785 | 3SZ7A | 151 | 1.72 |
| 3786 | 3SZYA | 413 | 1.35 |
| 3787 | 3T0HA | 208 | 1.2 |
| 3788 | 3T2CA | 389 | 1.3 |
| 3789 | 3T47B | 70 | 1.3 |
| 3790 | 3T49A | 70 | 1.45 |
| 3791 | 3T5SA | 97 | 2.3 |
| 3792 | 3T5XA | 195 | 2.12 |
| 3793 | 3T5XB | 30 | 2.12 |
| 3794 | 3T6OA | 118 | 2.1 |
| 3795 | 3T7KA | 233 | 2.03 |
| 3796 | 3T7LA | 74 | 1.09 |
| 3797 | 3T8BB | 201 | 1.65 |
| 3798 | 3T98A | 39 | 2.5 |
| 3799 | 3TBNA | 87 | 1.15 |
| 3800 | 3TC7A | 251 | 1.5 |
| 3801 | 3TC8B | 292 | 1.06 |
| 3802 | 3TD3E | 120 | 1.59 |
| 3803 | 3TDNB | 236 | 1.4 |
| 3804 | 3TDQB | 77 | 2.1 |
| 3805 | 3TDUD | 76 | 1.5 |
| 3806 | 3TE8A | 115 | 1.7 |
| 3807 | 3TFWB | 220 | 1.88 |
| 3808 | 3TGNA | 135 | 2 |
| 3809 | 3THRD | 285 | 2 |
| 3810 | 3TIWA | 160 | 1.8 |
| 3811 | 3TJ5B | 27 | 1.99 |
| 3812 | 3TJ8B | 152 | 1.59 |
| 3813 | 3TJYA | 78 | 1.7 |
| 3814 | 3TK9A | 226 | 2.2 |
| 3815 | 3TKTA | 411 | 2.2 |
| 3816 | 3TMPE | 169 | 1.91 |
| 3817 | 3TNJA | 130 | 2 |
| 3818 | 3TOWA | 152 | 1.34 |
| 3819 | 3TQEA | 316 | 1.5 |
| 3820 | 3TR3B | 81 | 2.46 |
| 3821 | 3TRTB | 73 | 2.3 |
| 3822 | 3TS3A | 203 | 1.49 |
| 3823 | 3TS9A | 128 | 2 |
| 3824 | 3TT8D | 30 | 1.12 |
| 3825 | 3TU8A | 210 | 1.04 |
| 3826 | 3TUFA | 115 | 2.26 |
| 3827 | 3TUOA | 104 | 1.7 |
| 3828 | 3TWEB | 25 | 1.36 |
| 3829 | 3TX2A | 247 | 1.5 |
| 3830 | 3TXSA | 89 | 1.81 |
| 3831 | 3TYSA | 75 | 1.12 |
| 3832 | 3TYTA | 204 | 1.6 |
| 3833 | 3U01A | 104 | 1.12 |
| 3834 | 3U02C | 226 | 2.4 |
| 3835 | 3U12B | 103 | 2.08 |
| 3836 | 3U1IA | 39 | 2.3 |
| 3837 | 3U23A | 56 | 1.11 |
| 3838 | 3U28B | 48 | 1.9 |
| 3839 | 3U2BC | 76 | 2.4 |
| 3840 | 3U2KA | 194 | 1.64 |
| 3841 | 3U2UB | 263 | 1.45 |
| 3842 | 3U3GA | 140 | 1.4 |
| 3843 | 3U3LC | 230 | 1.57 |
| 3844 | 3U4VA | 116 | 1.8 |
| 3845 | 3U52C | 318 | 1.95 |
| 3846 | 3U5RH | 184 | 2.05 |
| 3847 | 3U5VA | 62 | 1.7 |
| 3848 | 3U62A | 253 | 1.45 |
| 3849 | 3U7ID | 218 | 1.75 |
| 3850 | 3U7QA | 477 | 1 |
| 3851 | 3U7QD | 522 | 1 |
| 3852 | 3U7ZB | 96 | 1.3 |
| 3853 | 3U80A | 127 | 1.6 |
| 3854 | 3U97A | 77 | 1.1 |
| 3855 | 3U9GA | 217 | 1.8 |
| 3856 | 3U9HB | 209 | 1.75 |
| 3857 | 3U9QA | 257 | 1.52 |
| 3858 | 3UAWA | 233 | 1.2 |
| 3859 | 3UFEB | 101 | 1.5 |
| 3860 | 3UI4A | 101 | 0.8 |
| 3861 | 3UJCA | 258 | 1.19 |
| 3862 | 3ULTA | 114 | 1.4 |
| 3863 | 3UNPA | 193 | 2.39 |
| 3864 | 3UO3B | 147 | 1.85 |
| 3865 | 3UPVA | 125 | 1.6 |
| 3866 | 3US4A | 97 | 1.5 |
| 3867 | 3US6A | 148 | 1.45 |
| 3868 | 3UTMC | 32 | 2 |
| 3869 | 3UUWD | 303 | 1.63 |
| 3870 | 3UV4A | 125 | 1.89 |
| 3871 | 3UXJD | 261 | 1.4 |
| 3872 | 3UZED | 74 | 2.04 |
| 3873 | 3UZPA | 292 | 1.94 |
| 3874 | 3V0SA | 287 | 1.77 |
| 3875 | 3V1AA | 48 | 0.98 |
| 3876 | 3V2UD | 514 | 2.1 |
| 3877 | 3V3LA | 83 | 1.65 |
| 3878 | 3V46A | 160 | 1.55 |
| 3879 | 3V4HA | 131 | 2.1 |
| 3880 | 3V4KA | 186 | 1.38 |
| 3881 | 3V5AA | 335 | 1.44 |
| 3882 | 3V5UA | 297 | 1.9 |
| 3883 | 3V7PA | 409 | 1.35 |
| 3884 | 3V7QB | 97 | 1.55 |
| 3885 | 3V8HA | 312 | 1.65 |
| 3886 | 3V9OA | 121 | 1.45 |
| 3887 | 3VA4B | 108 | 1.54 |
| 3888 | 3VAAB | 176 | 1.7 |
| 3889 | 3VBLE | 188 | 1.9 |
| 3890 | 3VC8A | 81 | 2 |
| 3891 | 3VCXB | 120 | 1.39 |
| 3892 | 3VEJA | 40 | 1.23 |
| 3893 | 3VHVA | 255 | 1.35 |
| 3894 | 3VJZB | 163 | 1.8 |
| 3895 | 3VK6A | 96 | 1.9 |
| 3896 | 3VKEA | 76 | 1.77 |
| 3897 | 3VMKB | 368 | 1.48 |
| 3898 | 3VNYA | 466 | 1.5 |
| 3899 | 3YGSP | 97 | 2.5 |
| 3900 | 3ZQIA | 201 | 1.5 |
| 3901 | 3ZQOJ | 58 | 1.68 |
| 3902 | 3ZQUA | 207 | 1.5 |
| 3903 | 3ZR8X | 65 | 0.9 |
| 3904 | 3ZRGB | 65 | 1.75 |
| 3905 | 3ZRIA | 166 | 1.8 |
| 3906 | 3ZRXA | 111 | 1.25 |
| 3907 | 3ZS9C | 31 | 2.1 |
| 3908 | 3ZSJA | 138 | 0.86 |
| 3909 | 3ZTPA | 141 | 1.37 |
| 3910 | 3ZUCA | 153 | 1 |
| 3911 | 3ZUDA | 228 | 1.25 |
| 3912 | 3ZVKE | 57 | 2.5 |
| 3913 | 3ZVLA | 380 | 1.65 |
| 3914 | 3ZW5B | 123 | 1.6 |
| 3915 | 3ZWFA | 259 | 1.7 |
| 3916 | 3ZWLE | 39 | 2.2 |
| 3917 | 3ZX3A | 399 | 1.7 |
| 3918 | 3ZXOB | 125 | 1.9 |
| 3919 | 3ZY7B | 116 | 1.09 |
| 3920 | 3ZYLA | 260 | 1.7 |
| 3921 | 3ZZPA | 74 | 0.96 |
| 3922 | 3ZZSF | 65 | 1.49 |
| 3923 | 3ZZYA | 106 | 1.4 |
| 3924 | 451CA | 82 | 1.6 |
| 3925 | 4A02A | 166 | 0.95 |
| 3926 | 4A1UA | 150 | 1.54 |
| 3927 | 4A2VA | 122 | 1.44 |
| 3928 | 4A34T | 140 | 2.5 |
| 3929 | 4A3PA | 210 | 1.4 |
| 3930 | 4A3XA | 227 | 1.65 |
| 3931 | 4A49A | 77 | 2.21 |
| 3932 | 4A4YA | 128 | 1.57 |
| 3933 | 4A56A | 93 | 1.24 |
| 3934 | 4A69C | 69 | 2.06 |
| 3935 | 4A8JF | 262 | 2.1 |
| 3936 | 4A8TA | 317 | 1.59 |
| 3937 | 4A8XA | 88 | 1.9 |
| 3938 | 4A8XB | 27 | 1.9 |
| 3939 | 4A94D | 51 | 1.7 |
| 3940 | 4ACJA | 167 | 0.97 |
| 3941 | 4ADNB | 207 | 1.65 |
| 3942 | 4ADUB | 274 | 2.44 |
| 3943 | 4AEQA | 90 | 1.89 |
| 3944 | 4AFFA | 110 | 1.05 |
| 3945 | 4AFLD | 95 | 2.28 |
| 3946 | 4AJJA | 330 | 1.75 |
| 3947 | 4CPAI | 37 | 2.5 |
| 3948 | 4DBBA | 150 | 1.9 |
| 3949 | 4DDJA | 80 | 1.9 |
| 3950 | 4DEMF | 346 | 1.85 |
| 3951 | 4DFAA | 129 | 1.4 |
| 3952 | 4DGQC | 277 | 1.85 |
| 3953 | 4DM5A | 87 | 1.5 |
| 3954 | 4DNUA | 372 | 1.76 |
| 3955 | 4DOVA | 157 | 1.7 |
| 3956 | 4DS7H | 44 | 2.15 |
| 3957 | 4DZIC | 388 | 1.6 |
| 3958 | 4E0QB | 110 | 2.5 |
| 3959 | 4FIVA | 113 | 1.8 |
| 3960 | 4MT2A | 61 | 2 |
| 3961 | 4SGBI | 51 | 2.1 |
| 3962 | 4UBPB | 122 | 1.55 |
| 3963 | 5CSMA | 251 | 2 |
| 3964 | 6RLXD | 25 | 1.5 |
| 3965 | 7FD1A | 106 | 1.3 |
| 3966 | 7ODCA | 387 | 1.6 |
| 3967 | 8A3HA | 300 | 0.97 |
