# Supplementary material for: Analyses of the Sequence and Structural Properties Corresponding to Pentapeptide and Large Palindromes in Proteins
Source: PLoS One. 2015 Oct 14;10(10):e0139568. doi: 10.1371/journal.pone.0139568 (PMC4605511; doi:10.1371/journal.pone.0139568)
Supplement: S2 Appendix — (DOC) [file pone.0139568.s002.doc]

| **S2 Appendix. Palindromes in representative protein chains selected from the Protein Data Bank and their associated structural properties.** | | | | | | | | | | |
| --- | --- | --- | --- | --- | --- | --- | --- | --- | --- | --- |
| S. No. | PDB code | Protein name classification | Start position | End position | Palindrome sequence | Peptide length | Average solvent accessibility  (Å^2^) | Secondary structure conformation | Number of residue contacts | Average hydrophobicity values |
| 1 | 3NUL:A | actin binding protein | 64 | 68 | GLFLG | 5 | 25.66 | CEEET | 8 | 1.92 |
| 2 | 1V05:A | actin-binding protein | 2694 | 2698 | VTYTV | 5 | 10.26 | EEEEC | 9 | 1.14 |
| 3 | 1BJA:A | activation domain | 21 | 25 | TILIT | 5 | 5.54 | HHHHH | 7 | 2.28 |
| 4 | 1NLS:A | agglutinin | 113 | 117 | SKLKS | 5 | 39.90 | EEEEE | 10 | -1.12 |
| 5 | 1NLS:A | agglutinin | 185 | 190 | SAVVAS | 6 | 44.73 | TEEEEE | 6 | 1.73 |
| 6 | 1L3P:A | allergen | 169 | 175 | ATAAATA | 7 | 51.56 | HHHHHTS | 6 | 1.09 |
| 7 | 1L3P:A | allergen | 168 | 172 | AATAA | 5 | 30.40 | HHHHH | 7 | 1.30 |
| 8 | 1L3P:A | allergen | 170 | 174 | TAAAT | 5 | 65.98 | HHHHT | 6 | 0.80 |
| 9 | 3L4R:A | allergen, lipid binding protein | 100 | 104 | YLILY | 5 | 19.22 | EEEEE | 9 | 1.90 |
| 10 | 1GK9:A | antibiotic resistance | 121 | 125 | TFGFT | 5 | 57.16 | HHTCC | 5 | 0.76 |
| 11 | 4ADN:B | antibiotic resistance | 108 | 112 | KKVKK | 5 | 127.82 | SSCSS | 6 | -2.28 |
| 12 | 4ADN:B | antibiotic resistance | 60 | 64 | IDDDI | 5 | 61.24 | CCHHH | 6 | -0.30 |
| 13 | 1GK9:A | antibiotic resistance | 29 | 33 | YGYGY | 5 | 4.96 | HHHHH | 14 | -0.94 |
| 14 | 1BX7:A | anti-coagulant | 29 | 33 | CRIRC | 5 | 127.38 | CCCCC | 5 | 0.10 |
| 15 | 1P9G:A | antifungal protein | 36 | 40 | RCQCR | 5 | 90.82 | EECTT | 12 | -1.50 |
| 16 | 1P9G:A | antifungal protein | 7 | 11 | CPRPC | 5 | 65.56 | TTCCS | 12 | -0.54 |
| 17 | 1B3A:A | anti-hiv protein | 17 | 21 | RPLPR | 5 | 82.68 | SCCCG | 6 | -1.68 |
| 18 | 1B3A:A | anti-hiv protein | 16 | 22 | ARPLPRA | 7 | 78.60 | SSCCCGG | 6 | -0.69 |
| 19 | 1KLL:A | antimicrobial protein | 73 | 77 | FAIAF | 5 | 54.48 | CEEEE | 14 | 2.74 |
| 20 | 3E7R:L | antimicrobial protein | 15 | 19 | CHNHC | 5 | 41.82 | HHHHH | 10 | -0.98 |
| 21 | 2MCM:A | apoprotein | 98 | 102 | GSDSG | 5 | 71.18 | ECTTS | 3 | -1.18 |
| 22 | 1FEW:A | apoptosis | 167 | 171 | EEGEE | 5 | 65.72 | HHHHH | 9 | -2.88 |
| 23 | 1FEW:A | apoptosis | 112 | 116 | AAEAA | 5 | 26.22 | HHHHH | 9 | 0.74 |
| 24 | 1WMG:F | apoptosis | 923 | 927 | LASAL | 5 | 17.80 | HHHHH | 6 | 2.08 |
| 25 | 2A26:A | apoptosis | 36 | 40 | IETEI | 5 | 46.40 | HHHHH | 9 | 0.26 |
| 26 | 2NL9:A | apoptosis | 216 | 220 | VGDGV | 5 | 14.42 | HHHHH | 10 | 0.82 |
| 27 | 2O71:A | apoptosis | 151 | 155 | NHPHN | 5 | 96.00 | HCTTC | 6 | -3.00 |
| 28 | 2O71:A | apoptosis | 129 | 133 | PEWEP | 5 | 54.80 | TTHHH | 6 | -2.22 |
| 29 | 2O71:A | apoptosis | 156 | 160 | VQSQV | 5 | 41.74 | HHHHH | 7 | 0.12 |
| 30 | 2O71:A | apoptosis | 137 | 141 | SLGLS | 5 | 37.18 | HTTCC | 6 | 1.12 |
| 31 | 2VOG:A | apoptosis | 46 | 50 | KEVEK | 5 | 76.00 | HHHHH | 7 | -2.12 |
| 32 | 3AJM:A | apoptosis | 134 | 138 | IASAI | 5 | 22.08 | HHHHH | 8 | 2.36 |
| 33 | 3MX7:A | apoptosis | 59 | 63 | AKTKA | 5 | 66.12 | TTEEE | 5 | -0.98 |
| 34 | 3QO4:A | apoptosis | 152 | 156 | VGWGV | 5 | 36.24 | TTEEE | 7 | 1.34 |
| 35 | 3R8J:A | apoptosis | 32 | 36 | GPQPG | 5 | 77.18 | CCCCT | 3 | -1.50 |
| 36 | 3R8J:A | apoptosis | 95 | 99 | PGSGP | 5 | 72.02 | CTTST | 3 | -0.96 |
| 37 | 3R8J:A | apoptosis | 81 | 85 | MKIKM | 5 | 85.52 | CCCCC | 5 | 0.10 |
| 38 | 3R8J:A | apoptosis | 49 | 53 | VSTSV | 5 | 1.64 | EEEEE | 5 | 1.22 |
| 39 | 3YGS:P | apoptosis | 7 | 12 | RRLLRR | 6 | 66.45 | HHHHHH | 8 | -1.73 |
| 40 | 2A26:A | apoptosis | 35 | 41 | KIETEIK | 7 | 60.50 | HHHHHHH | 9 | -0.93 |
| 41 | 1GL4:A | basement membrane | 406 | 410 | GKVKG | 5 | 43.96 | EEEEE | 10 | -0.88 |
| 42 | 1GL4:A | basement membrane | 552 | 556 | TSSST | 5 | 48.56 | EEEEE | 10 | -0.76 |
| 43 | 1GL4:A | basement membrane | 500 | 504 | KLVLK | 5 | 53.86 | CEEEE | 8 | 0.80 |
| 44 | 3HRQ:B | biosynthetic protein | 1516 | 1520 | AENEA | 5 | 35.08 | GGTEE | 7 | -1.38 |
| 45 | 1I24:A | biosynthetic protein | 226 | 230 | LRNRL | 5 | 10.84 | GCCCC | 12 | -0.98 |
| 46 | 1I24:A | biosynthetic protein | 344 | 348 | ELGLE | 5 | 62.76 | HTTCC | 12 | 0.04 |
| 47 | 1I24:A | biosynthetic protein | 352 | 356 | LSDSL | 5 | 55.98 | CCHHH | 12 | 0.50 |
| 48 | 1RZ4:A | biosynthetic protein | 12 | 17 | GKLLKG | 6 | 68.72 | HHHHHS | 6 | -0.17 |
| 49 | 2EEY:A | biosynthetic protein | 33 | 37 | SVTVS | 5 | 18.36 | EEEEC | 8 | 1.22 |
| 50 | 3CKM:A | biosynthetic protein | 471 | 475 | TNTNT | 5 | 65.56 | HHTCH | 9 | -1.82 |
| 51 | 3CKM:A | biosynthetic protein | 466 | 470 | ASASA | 5 | 32.96 | GCCHH | 9 | 0.76 |
| 52 | 3G2B:A | biosynthetic protein | 21 | 25 | DRARD | 5 | 127.24 | EGGGT | 2 | -2.84 |
| 53 | 3G2B:A | biosynthetic protein | 80 | 84 | LTTTL | 5 | 35.40 | HHHHH | 9 | 1.10 |
| 54 | 3HRQ:B | biosynthetic protein | 1514 | 1518 | NEAEN | 5 | 42.84 | EGGGT | 6 | -2.44 |
| 55 | 3NGW:A | biosynthetic protein | 106 | 110 | KEGEK | 5 | 75.44 | HHHHH | 8 | -3.04 |
| 56 | 3PL0:A | biosynthetic protein | 239 | 243 | TLVLT | 5 | 10.32 | EEEEE | 9 | 2.08 |
| 57 | 3LUU:A | biosynthetic protein | 65 | 69 | VVEVV | 5 | 61.90 | EEEEE | 4 | 2.66 |
| 58 | 3E1I:A | blood clotting | 167 | 171 | RALAR | 5 | 68.04 | CCCCC | 4 | -0.32 |
| 59 | 3DY0:A | blood clotting, hydrolase inhibitor | 28 | 32 | DFTFD | 5 | 14.30 | CHHHH | 7 | -0.42 |
| 60 | 1DYO:A | carbohydrate-binding module | 153 | 157 | GAVAG | 5 | 27.32 | EEETT | 7 | 1.40 |
| 61 | 1F00:I | cell adhesion | 872 | 876 | NELEN | 5 | 60.46 | HHHHH | 7 | -2.04 |
| 62 | 1F00:I | cell adhesion | 738 | 742 | DVAVD | 5 | 84.74 | TCCCC | 4 | 0.64 |
| 63 | 1F00:I | cell adhesion | 764 | 768 | VGTGV | 5 | 50.66 | TTTCC | 4 | 1.38 |
| 64 | 1N67:A | cell adhesion | 555 | 559 | EPIPE | 5 | 57.30 | CSSCC | 5 | -1.14 |
| 65 | 1N67:A | cell adhesion | 532 | 536 | GSGSG | 5 | 49.30 | SSCSC | 5 | -0.56 |
| 66 | 1O6V:B | cell adhesion | 321 | 325 | NLELN | 5 | 16.20 | EEECC | 6 | -0.58 |
| 67 | 1O6V:B | cell adhesion | 66 | 70 | VTDTV | 5 | 44.20 | TTCEE | 6 | 0.70 |
| 68 | 1O6V:B | cell adhesion | 343 | 347 | YLTLY | 5 | 25.34 | EEECC | 6 | 0.86 |
| 69 | 1OQV:A | cell adhesion | 101 | 105 | NAAAN | 5 | 63.52 | TTEEE | 5 | -0.32 |
| 70 | 1OQV:A | cell adhesion | 60 | 64 | TADAT | 5 | 58.40 | CSSHH | 5 | -0.26 |
| 71 | 1OQV:A | cell adhesion | 59 | 65 | ATADATA | 7 | 56.41 | CCSSHHH | 5 | 0.33 |
| 72 | 1QVE:A | cell adhesion | 112 | 116 | TLTLT | 5 | 21.64 | EEEEE | 8 | 1.10 |
| 73 | 1UX6:A | cell adhesion | 906 | 910 | DGRGD | 5 | 58.04 | SSSCG | 8 | -2.46 |
| 74 | 1UX6:A | cell adhesion | 828 | 832 | DSDSD | 5 | 38.28 | CTTSS | 8 | -2.42 |
| 75 | 1UX6:A | cell adhesion | 870 | 874 | DGKGD | 5 | 52.26 | SSSCG | 8 | -2.34 |
| 76 | 1UX6:A | cell adhesion | 1006 | 1010 | GFVFG | 5 | 0.88 | EEEEE | 7 | 1.80 |
| 77 | 1WPA:A | cell adhesion | 469 | 473 | EESEE | 5 | 106.04 | TTSHH | 4 | -2.96 |
| 78 | 1WPA:A | cell adhesion | 503 | 507 | LKSKL | 5 | 48.24 | HHHHH | 8 | -0.20 |
| 79 | 1Z9M:B | cell adhesion | 104 | 108 | SISIS | 5 | 13.98 | EEEES | 9 | 1.32 |
| 80 | 1Z9M:B | cell adhesion | 133 | 137 | LVTVL | 5 | 41.36 | EEEEE | 8 | 3.06 |
| 81 | 2H3L:B | cell adhesion | 1338 | 1342 | GGVGG | 5 | 36.62 | ECTTS | 6 | 0.52 |
| 82 | 2P26:A | cell adhesion | 431 | 435 | SRDRS | 5 | 137.56 | TTCTT | 11 | -2.82 |
| 83 | 2P26:A | cell adhesion | 376 | 380 | GDCDG | 5 | 28.32 | EEESS | 11 | -1.06 |
| 84 | 2V5T:A | cell adhesion | 297 | 301 | QVFVQ | 5 | 96.84 | EEECC | 5 | 0.84 |
| 85 | 2VN5:A | cell adhesion | 29 | 33 | TVPVT | 5 | 29.28 | EEEEE | 7 | 1.08 |
| 86 | 2VZC:A | cell adhesion | 284 | 288 | TELET | 5 | 62.30 | CCTTT | 5 | -0.92 |
| 87 | 2X9Z:A | cell adhesion | 253 | 257 | TVKVT | 5 | 42.86 | CCEEE | 7 | 0.62 |
| 88 | 2X9Z:A | cell adhesion | 357 | 361 | APIPA | 5 | 68.34 | CBCCC | 7 | 0.98 |
| 89 | 2XJP:A | cell adhesion | 61 | 65 | AYGYA | 5 | 28.34 | HTGGG | 17 | 0.12 |
| 90 | 2XJP:A | cell adhesion | 78 | 82 | DISID | 5 | 42.38 | BCCEE | 17 | 0.24 |
| 91 | 2Y7L:A | cell adhesion | 99 | 103 | SVSVS | 5 | 92.64 | CCSSC | 2 | 1.20 |
| 92 | 3D9X:A | cell adhesion | 392 | 396 | NNDNN | 5 | 88.62 | TTCCC | 6 | -3.50 |
| 93 | 3FYQ:A | cell adhesion | 2044 | 2049 | TGAAGT | 6 | 52.03 | HHTSSC | 8 | 0.23 |
| 94 | 3K6I:A | cell adhesion | 6 | 10 | PILIP | 5 | 54.00 | CEEEE | 5 | 1.92 |
| 95 | 3M7P:A | cell adhesion | 559 | 563 | DQCQD | 5 | 40.56 | CEEEC | 6 | -2.30 |
| 96 | 3M7P:A | cell adhesion | 353 | 357 | GNSNG | 5 | 55.50 | TTTTS | 6 | -1.72 |
| 97 | 3M7P:A | cell adhesion | 414 | 417 | GNSNG | 5 | 55.50 | TTTTS | 6 | -1.72 |
| 98 | 3MVS:A | cell adhesion | 163 | 167 | IVTVI | 5 | 34.32 | EEEEC | 10 | 3.34 |
| 99 | 3MW4:A | cell adhesion | 215 | 219 | GKDKG | 5 | 71.06 | SGGGT | 4 | -2.42 |
| 100 | 3MW4:A | cell adhesion | 246 | 250 | NIKIN | 5 | 80.56 | TEEEE | 4 | -0.38 |
| 101 | 3S6L:D | cell adhesion | 70 | 74 | SGSGS | 5 | 29.94 | CSTTC | 7 | -0.64 |
| 102 | 3S6L:D | cell adhesion | 21 | 25 | AGNGA | 5 | 53.78 | ESTTC | 7 | -0.14 |
| 103 | 3SOJ:B | cell adhesion | 72 | 76 | GSTSG | 5 | 44.10 | EETTE | 5 | -0.62 |
| 104 | 3TDQ:B | cell adhesion | 32 | 36 | EVQVE | 5 | 84.26 | EEEGG | 5 | -0.42 |
| 105 | 2QSQ:B | cell adhesion | 17 | 21 | VLLLV | 5 | 23.42 | EEEEE | 6 | 3.96 |
| 106 | 2Y7L:A | cell adhesion | 270 | 274 | YTLTY | 5 | 19.18 | EEEEE | 9 | -0.04 |
| 107 | 3KLQ:B | cell adhesion | 36 | 41 | TIEEIT | 6 | 66.95 | EEEEEE | 6 | 0.10 |
| 108 | 3M7P:A | cell adhesion | 297 | 301 | QPHPQ | 5 | 99.88 | CCCCC | 2 | -2.68 |
| 109 | 3M7P:A | cell adhesion | 332 | 336 | LCTCL | 5 | 21.84 | EEEEE | 2 | 2.38 |
| 110 | 2X9Z:A | cell adhesion | 356 | 362 | GAPIPAG | 7 | 58.54 | SCBCCCC | 5 | 0.59 |
| 111 | 1QVE:A | cell adhesion | 113 | 117 | LTLTL | 5 | 16.28 | EEEEE | 8 | 2.00 |
| 112 | 1O6V:B | cell adhesion | 187 | 192 | SLQQLS | 6 | 34.03 | TCSEEE | 13 | -0.17 |
| 113 | 1FNF:A | cell adhesion protein | 1183 | 1188 | NGQQGN | 6 | 98.85 | TTCSSC | 7 | -2.47 |
| 114 | 1EYH:A | cell cycle | 135 | 139 | RDEDR | 5 | 107.56 | HCHHH | 7 | -3.90 |
| 115 | 1EYH:A | cell cycle | 56 | 60 | IMSMI | 5 | 19.72 | HHHHH | 8 | 2.40 |
| 116 | 1G6G:A | cell cycle | 98 | 102 | NLLLN | 5 | 21.52 | CEEEE | 11 | 0.88 |
| 117 | 1G6G:A | cell cycle | 132 | 136 | VGVGV | 5 | 36.62 | ECTTS | 11 | 2.36 |
| 118 | 1GO4:G | cell cycle | 490 | 494 | SFLFS | 5 | 57.66 | HHHHH | 8 | 1.56 |
| 119 | 1T6F:A | cell cycle | 7 | 11 | KENEK | 5 | 93.08 | HHHHH | 9 | -3.66 |
| 120 | 1TVG:A | cell cycle | 75 | 81 | EKSTSKE | 7 | 61.39 | EEECSSS | 7 | -2.44 |
| 121 | 1TVG:A | cell cycle | 76 | 80 | KSTSK | 5 | 62.94 | EECSS | 7 | -2.02 |
| 122 | 2I53:A | cell cycle | 241 | 245 | DVPVD | 5 | 70.92 | SCCHH | 7 | -0.04 |
| 123 | 2WM9:A | cell cycle | 334 | 338 | KLQLK | 5 | 16.66 | HHHHH | 10 | -0.74 |
| 124 | 2WM9:A | cell cycle | 161 | 165 | FGQGF | 5 | 43.06 | ECHHH | 5 | 0.26 |
| 125 | 2WM9:A | cell cycle | 160 | 166 | FFGQGFF | 7 | 33.11 | EECHHHH | 5 | 0.99 |
| 126 | 2WUJ:B | cell cycle | 11 | 15 | KTFTK | 5 | 78.24 | CCCCE | 6 | -1.28 |
| 127 | 3EFY:B | cell cycle | 139 | 143 | EQLQE | 5 | 87.02 | HHHHH | 5 | -2.04 |
| 128 | 3ESL:A | cell cycle | 59 | 63 | DLFLD | 5 | 30.88 | HHHHH | 7 | 0.68 |
| 129 | 3L41:A | cell cycle | 792 | 796 | VISIV | 5 | 22.64 | HHHHH | 9 | 3.32 |
| 130 | 3PA6:C | cell cycle | 70 | 74 | LVSVL | 5 | 31.34 | EECHH | 19 | 3.04 |
| 131 | 3RYC:B | cell cycle | 437 | 441 | DATAD | 5 | 71.82 | HCCTT | 4 | -0.82 |
| 132 | 3RYC:B | cell cycle | 216 | 220 | TLKLT | 5 | 77.70 | TSCCS | 4 | 0.46 |
| 133 | 2WM9:A | cell cycle | 336 | 340 | QLKLQ | 5 | 6.44 | HHHHH | 12 | -0.66 |
| 134 | 1EYH:A | cell cycle | 134 | 140 | LRDEDRL | 7 | 79.16 | HHCHHHH | 8 | -1.70 |
| 135 | 1T6F:A | cell cycle | 6 | 12 | LKENEKL | 7 | 81.91 | HHHHHHH | 9 | -1.53 |
| 136 | 3BY4:A | cell cycle, hydrolase | 249 | 253 | ALQLA | 5 | 28.80 | HHHHH | 8 | 1.54 |
| 137 | 2QFA:A | cell cycle/cell cycle/cell cycle | 69 | 73 | PDDDP | 5 | 51.50 | TTCCH | 6 | -2.74 |
| 138 | 1H4X:B | cell differentiation | 61 | 65 | GLVLG | 5 | 20.08 | HHHHH | 7 | 2.20 |
| 139 | 3QRA:A | cell invasion | 149 | 155 | FNPHPNF | 7 | 68.04 | ECSSTTE | 6 | -1.11 |
| 140 | 1XL3:A | cell invasion | 106 | 110 | LLSLL | 5 | 50.00 | HHHHH | 8 | 2.88 |
| 141 | 2AXW:B | cell invasion | 123 | 127 | LAAAL | 5 | 64.84 | ECCCE | 15 | 2.60 |
| 142 | 3QRA:A | cell invasion | 150 | 154 | NPHPN | 5 | 61.28 | CSSTT | 5 | -2.68 |
| 143 | 3QRA:A | cell invasion | 160 | 164 | SYEYS | 5 | 33.22 | EEEEE | 12 | -1.54 |
| 144 | 2AXW:B | cell invasion | 2 | 6 | ELHLE | 5 | 58.04 | EEEEE | 21 | -0.52 |
| 145 | 1AOH:A | cellulosome subunit | 143 | 147 | GVNVG | 5 | 28.00 | EEEEC | 8 | 0.82 |
| 146 | 1EJF:B | chaperone | 9 | 14 | YDRRDY | 6 | 57.00 | EECSSE | 8 | -3.10 |
| 147 | 1H7C:A | chaperone | 89 | 93 | EEAEE | 5 | 78.24 | TTSHH | 7 | -2.44 |
| 148 | 1HW7:A | chaperone | 161 | 165 | GDVDG | 5 | 87.54 | EEETT | 5 | -0.72 |
| 149 | 1HW7:A | chaperone | 57 | 61 | LTATL | 5 | 18.28 | HHTTC | 5 | 1.60 |
| 150 | 1ORJ:D | chaperone | 4047 | 4051 | EKRKE | 5 | 84.44 | HHHHH | 9 | -3.86 |
| 151 | 1ORJ:D | chaperone | 4073 | 4077 | EKGKE | 5 | 88.10 | TTSHH | 7 | -3.04 |
| 152 | 1ORJ:D | chaperone | 4120 | 4124 | VKKKV | 5 | 77.62 | HHHHH | 9 | -0.66 |
| 153 | 1ORY:B | chaperone | 2511 | 2515 | QLVLQ | 5 | 70.12 | HHHHH | 5 | 0.96 |
| 154 | 1RY9:D | chaperone | 42 | 46 | AINIA | 5 | 14.46 | EEEEE | 6 | 1.82 |
| 155 | 2D0O:D | chaperone | 78 | 82 | PASAP | 5 | 47.50 | CTTCC | 9 | -0.08 |
| 156 | 2D0O:D | chaperone | 9 | 13 | AIAIA | 5 | 9.00 | CEEEE | 9 | 2.88 |
| 157 | 2P4F:A | chaperone | 108 | 112 | DKIKD | 5 | 108.48 | HHHTT | 3 | -2.06 |
| 158 | 2P4F:A | chaperone | 107 | 113 | LDKIKDL | 7 | 92.96 | HHHHTTS | 3 | -0.39 |
| 159 | 2QIF:B | chaperone | 50 | 54 | KVSVK | 5 | 72.86 | TSCHH | 6 | -0.04 |
| 160 | 2QIF:B | chaperone | 56 | 60 | IADAI | 5 | 16.00 | HHHHH | 7 | 1.82 |
| 161 | 2QSA:A | chaperone | 115 | 119 | YYQYY | 5 | 64.66 | HHHHH | 8 | -1.74 |
| 162 | 2QSA:A | chaperone | 58 | 62 | RALAR | 5 | 68.88 | HHHHH | 8 | -0.32 |
| 163 | 2R31:A | chaperone | 48 | 52 | ALALA | 5 | 26.44 | HHHHH | 5 | 2.60 |
| 164 | 2WJ5:A | chaperone | 89 | 93 | SAQAS | 5 | 117.58 | CCCCS | 2 | -0.30 |
| 165 | 2XMJ:A | chaperone | 13 | 17 | EACAE | 5 | 47.90 | HHHHH | 5 | -0.18 |
| 166 | 3AEI:B | chaperone | 8 | 12 | ELQLE | 5 | 41.90 | HHHHH | 9 | -0.58 |
| 167 | 3DQG:A | chaperone | 542 | 546 | KEAEK | 5 | 105.16 | HHHHH | 6 | -2.60 |
| 168 | 3DQG:A | chaperone | 523 | 527 | QIVIQ | 5 | 75.24 | EEEEE | 6 | 1.24 |
| 169 | 3I38:J | chaperone | 269 | 273 | VIKIV | 5 | 35.52 | EEEEC | 8 | 2.70 |
| 170 | 3T0H:A | chaperone | 134 | 138 | FGVGF | 5 | 19.30 | GTCGG | 24 | 1.80 |
| 171 | 3UO3:B | chaperone | 89 | 93 | TQEQT | 5 | 123.40 | TSTTT | 4 | -2.38 |
| 172 | 2WJ5:A | chaperone | 35 | 39 | HVEVH | 5 | 24.36 | EEEEE | 9 | -0.30 |
| 173 | 2QIF:B | chaperone | 49 | 55 | DKVSVKD | 7 | 82.96 | TTSCHHH | 6 | -1.03 |
| 174 | 2D0O:D | chaperone | 77 | 83 | LPASAPL | 7 | 39.07 | SCTTCCS | 5 | 1.03 |
| 175 | 2R31:A | chaperone | 186 | 190 | IRGRI | 5 | 55.26 | HTTSS | 4 | -0.08 |
| 176 | 2R31:A | chaperone | 41 | 45 | PLRLP | 5 | 37.84 | BCCBS | 4 | -0.02 |
| 177 | 3AEI:B | chaperone | 10 | 14 | QLELQ | 5 | 58.42 | HHHHH | 9 | -0.58 |
| 178 | 1FXK:C | chaperone | 80 | 84 | VGAGV | 5 | 20.56 | EETTE | 4 | 1.88 |
| 179 | 3N72:B | chaperone activator | 136 | 140 | EKIKE | 5 | 58.54 | HHHHH | 9 | -2.06 |
| 180 | 3SZ7:A | chaperone regulator | 243 | 247 | DDVDD | 5 | 140.82 | CCCCC | 2 | -1.96 |
| 181 | 3SZ7:A | chaperone regulator | 242 | 248 | ADDVDDA | 7 | 121.63 | CCCCCCS | 2 | -0.89 |
| 182 | 1P5U:C | chaperone, structural protein | 142 | 146 | VTVTV | 5 | 18.46 | EEEEE | 9 | 2.24 |
| 183 | 2FM8:A | chaperone/cell invasion | 42 | 46 | SICIS | 5 | 15.06 | CEEEE | 7 | 1.98 |
| 184 | 3AJI:D | chaperone/protein binding | 346 | 350 | STITS | 5 | 56.54 | HHHHT | 6 | 0.30 |
| 185 | 1GR3:A | collagen | 636 | 640 | ASGSA | 5 | 28.02 | EEEEE | 7 | 0.32 |
| 186 | 1AY7:B | complex (enzyme/inhibitor) | 76 | 80 | EAKAE | 5 | 74.32 | HHHHT | 9 | -1.46 |
| 187 | 5CSM:A | complex (isomerase/peptide) | 244 | 248 | EVEVE | 5 | 32.16 | HHHHH | 10 | -0.42 |
| 188 | 5CSM:A | complex (isomerase/peptide) | 27 | 31 | IFKFI | 5 | 34.44 | HHHHH | 10 | 2.14 |
| 189 | 1CNU:A | contractile | 112 | 116 | QVEVQ | 5 | 65.76 | CEEEE | 7 | -0.42 |
| 190 | 1CNU:A | contractile | 35 | 39 | EVVVE | 5 | 37.44 | EEEEE | 8 | 1.12 |
| 191 | 1CV8:A | cysteine protease | 74 | 78 | GQTQG | 5 | 48.36 | HHHTT | 7 | -1.70 |
| 192 | 1ALU:A | cytokine | 138 | 142 | TPDPT | 5 | 84.40 | CCCHH | 4 | -1.62 |
| 193 | 1BGC:A | cytokine | 63 | 67 | SSCSS | 5 | 39.74 | GGGST | 8 | -0.14 |
| 194 | 1WU3:I | cytokine | 18 | 23 | QELLEQ | 6 | 52.35 | HHHHHT | 6 | -1.07 |
| 195 | 1WU3:I | cytokine | 5 | 9 | QLQLQ | 5 | 55.20 | HHHHH | 8 | -0.58 |
| 196 | 2D48:A | cytokine | 40 | 44 | TEKET | 5 | 72.54 | CHHHH | 6 | -2.46 |
| 197 | 2HEW:F | cytokine | 126 | 130 | PISIP | 5 | 53.34 | CEEEC | 5 | 1.00 |
| 198 | 2ILK:A | cytokine | 101 | 105 | LRLRL | 5 | 40.50 | HHHHH | 10 | 0.48 |
| 199 | 2TNF:C | cytokine | 34 | 39 | NALLAN | 6 | 40.83 | TCEEET | 8 | 0.70 |
| 200 | 2VXT:I | cytokine | 171 | 175 | KLILK | 5 | 30.20 | EEEEE | 10 | 0.86 |
| 201 | 3GV3:A | cytokine | 8 | 12 | RCPCR | 5 | 89.92 | SCSCS | 4 | -1.12 |
| 202 | 3PIW:A | cytokine | 56 | 60 | VAKAV | 5 | 17.84 | HHHHH | 9 | 1.62 |
| 203 | 2ILK:A | cytokine | 102 | 106 | RLRLR | 5 | 56.14 | HHHHH | 11 | -1.18 |
| 204 | 1ALU:A | cytokine | 137 | 143 | TTPDPTT | 7 | 87.99 | CCCCHHH | 4 | -1.36 |
| 205 | 2HEW:F | cytokine | 101 | 106 | LYIIYL | 6 | 31.75 | EEEEEE | 10 | 2.33 |
| 206 | 2RE9:C | cytokine, hormone/growth factor | 33 | 37 | QTPTQ | 5 | 95.32 | CCCCC | 4 | -2.00 |
| 207 | 1XU1:D | cytokine, hormone/growth factor receptor | 108 | 112 | VLHLV | 5 | 4.64 | EEEEE | 10 | 2.56 |
| 208 | 2B5I:B | cytokine/cytokine receptor | 88 | 92 | VRWRV | 5 | 117.88 | TSCEE | 5 | -0.30 |
| 209 | 3DLQ:R | cytokine/cytokine receptor | 141 | 145 | PTPTP | 5 | 39.96 | CCBCS | 7 | -1.24 |
| 210 | 3OG4:A | cytokine/cytokine receptor | 72 | 76 | LEAEL | 5 | 15.64 | HHHHH | 12 | 0.48 |
| 211 | 3OG4:A | cytokine/cytokine receptor | 71 | 77 | ALEAELA | 7 | 18.00 | HHHHHHH | 12 | 0.86 |
| 212 | 3KAN:C | cytokine/inhibitor | 94 | 98 | RILIR | 5 | 52.62 | GEEEE | 7 | 0.76 |
| 213 | 3KAN:C | cytokine/inhibitor | 45 | 49 | LAMAL | 5 | 52.64 | CCCCB | 7 | 2.62 |
| 214 | 1IAR:B | cytokine/receptor | 126 | 130 | NYLYN | 5 | 35.62 | STTGG | 8 | -1.16 |
| 215 | 1IAR:B | cytokine/receptor | 174 | 178 | ARVRA | 5 | 26.04 | EEEEE | 10 | -0.24 |
| 216 | 1IAR:B | cytokine/receptor | 113 | 117 | TLLLT | 5 | 41.18 | CEEEE | 8 | 2.00 |
| 217 | 3S0R:B | de novo protein | 17 | 23 | AQLALQA | 7 | 59.21 | HHHHHHH | 10 | 0.86 |
| 218 | 1QYS:A | de novo protein | 58 | 62 | KEAEK | 5 | 66.56 | HHHHH | 8 | -2.60 |
| 219 | 2I5V:O | de novo protein | 209 | 214 | ATKKTA | 6 | 54.53 | TTCEEE | 6 | -0.93 |
| 220 | 2I5V:O | de novo protein | 208 | 215 | AATKKTAA | 8 | 59.51 | TTTCEEEE | 6 | -0.25 |
| 221 | 2I5V:O | de novo protein | 196 | 200 | EVSVE | 5 | 59.04 | CEEEE | 6 | 0.12 |
| 222 | 2P09:A | de novo protein | 24 | 28 | VKCKV | 5 | 88.32 | TTTSS | 3 | 0.62 |
| 223 | 3HE4:B | de novo protein | 34 | 38 | LEFEL | 5 | 56.60 | HHHHH | 7 | 0.68 |
| 224 | 3HE5:A | de novo protein | 14 | 18 | ENENE | 5 | 73.48 | HHHHH | 10 | -3.50 |
| 225 | 3R46:F | de novo protein | 11 | 15 | KAIAK | 5 | 63.98 | HHHHH | 8 | 0.06 |
| 226 | 3S0R:B | de novo protein | 18 | 22 | QLALQ | 5 | 62.84 | HHHHH | 10 | 0.48 |
| 227 | 3TDN:B | de novo protein | 71 | 76 | PLTTLP | 6 | 45.67 | GGCSSC | 11 | 0.50 |
| 228 | 3TDN:B | de novo protein | 3 | 7 | AVVVA | 5 | 3.38 | TEEEE | 11 | 3.24 |
| 229 | 1KD8:B | de novo protein | 12 | 16 | LKSKL | 5 | 53.52 | HHHHH | 9 | -0.20 |
| 230 | 2I5V:O | de novo protein | 119 | 125 | KIIIIIK | 7 | 63.86 | CCEEEEE | 10 | 2.10 |
| 231 | 1BB1:C | de novo protein design | 23 | 28 | EIAAIE | 6 | 38.43 | HHHHHH | 9 | 0.93 |
| 232 | 1BB1:C | de novo protein design | 22 | 29 | QEIAAIEQ | 8 | 53.11 | HHHHHHHH | 9 | -0.18 |
| 233 | 1FVK:A | disulfide oxidoreductase | 25 | 29 | FFSFF | 5 | 13.74 | EECTT | 7 | 2.08 |
| 234 | 2CXY:A | dna binding protein | 82 | 87 | SSAASS | 6 | 48.95 | HHHHHH | 5 | 0.07 |
| 235 | 1G5H:C | dna binding protein | 230 | 234 | LRHRL | 5 | 34.94 | HHHHH | 11 | -0.92 |
| 236 | 1N1J:B | dna binding protein | 101 | 105 | IAMAI | 5 | 9.64 | HHHHH | 5 | 2.90 |
| 237 | 1NH9:A | dna binding protein | 41 | 45 | AVDVA | 5 | 27.94 | HHHHH | 8 | 1.70 |
| 238 | 2NOG:A | dna binding protein | 813 | 817 | EELEE | 5 | 74.08 | HHHHH | 6 | -2.04 |
| 239 | 2OKF:A | dna binding protein | 115 | 119 | EIIIE | 5 | 53.90 | HHHHH | 7 | 1.30 |
| 240 | 2P5K:A | dna binding protein | 59 | 63 | SYKYS | 5 | 51.82 | EEEEE | 16 | -1.62 |
| 241 | 3B0B:C | dna binding protein | 48 | 52 | AARAA | 5 | 11.64 | HHHHH | 9 | 0.54 |
| 242 | 3FPN:B | dna binding protein | 222 | 226 | ERIRE | 5 | 41.48 | EEEEE | 10 | -2.30 |
| 243 | 3M9Q:A | dna binding protein | 80 | 84 | RQLQR | 5 | 60.50 | HHHHH | 12 | -2.44 |
| 244 | 3R9Z:A | dna binding protein | 184 | 188 | TVPVT | 5 | 32.32 | EEEEE | 9 | 1.08 |
| 245 | 3R9Z:A | dna binding protein | 198 | 202 | AFSFA | 5 | 50.70 | HHHHH | 9 | 1.68 |
| 246 | 1G5H:C | dna binding protein | 378 | 383 | NELLEN | 6 | 51.48 | HHHHHT | 7 | -1.07 |
| 247 | 1KW4:A | dna binding protein | 18 | 23 | SVDDVS | 6 | 45.22 | CHHHHH | 5 | -0.03 |
| 248 | 1YDX:A | dna binding protein | 30 | 34 | LEKEL | 5 | 100.16 | CCGGG | 2 | -0.66 |
| 249 | 2CXY:A | dna binding protein | 108 | 112 | EPPPE | 5 | 104.72 | CCCCS | 2 | -2.36 |
| 250 | 2G9W:B | dna binding protein | 120 | 124 | AELEA | 5 | 105.64 | HHHHC | 4 | 0.08 |
| 251 | 2OKF:A | dna binding protein | 129 | 133 | VEQEV | 5 | 87.50 | TTTTE | 3 | -0.42 |
| 252 | 2P5M:C | dna binding protein | 145 | 149 | LLELL | 5 | 64.26 | HHHTC | 4 | 2.34 |
| 253 | 3F6C:B | dna binding protein | 51 | 55 | IDVDI | 5 | 15.84 | EETTC | 8 | 1.24 |
| 254 | 3OMY:A | dna binding protein | 8 | 12 | VNNNV | 5 | 91.00 | CCHHH | 5 | -0.42 |
| 255 | 3SIB:A | dna binding protein | 96 | 100 | FDTDF | 5 | 53.08 | HCTTC | 8 | -0.42 |
| 256 | 3OMY:A | dna binding protein | 7 | 13 | YVNNNVY | 7 | 109.00 | CCCHHHH | 6 | -0.67 |
| 257 | 2BW3:B | dna recombination | 155 | 159 | AKEKA | 5 | 128.00 | HHTCC | 4 | -1.54 |
| 258 | 1MUN:A | dna repair | 126 | 130 | LSLSL | 5 | 20.84 | HHHHH | 8 | 1.96 |
| 259 | 2JK1:A | dna-binding | 128 | 132 | RENER | 5 | 90.38 | HHHHH | 8 | -3.90 |
| 260 | 1FIA:A | dna-binding protein | 90 | 94 | KKLKK | 5 | 95.02 | HHHHH | 8 | -2.36 |
| 261 | 1GVP:A | dna-binding protein | 54 | 58 | PAYAP | 5 | 54.18 | CCCCS | 6 | -0.18 |
| 262 | 2VQC:A | dna-binding protein | 23 | 27 | ELTLE | 5 | 46.28 | EECHH | 7 | -0.02 |
| 263 | 3ZQO:J | dna-binding protein | 84 | 88 | QEKEQ | 5 | 66.30 | CCEEE | 7 | -3.58 |
| 264 | 1RCF:A | electron transfer(flavoprotein) | 9 | 13 | GTQTG | 5 | 48.52 | CCSSS | 11 | -1.14 |
| 265 | 1E29:A | electron transport | 69 | 73 | VLALV | 5 | 26.28 | HHHHH | 9 | 3.56 |
| 266 | 1FT5:A | electron transport | 42 | 46 | AKQKA | 5 | 56.04 | HHHHT | 10 | -1.54 |
| 267 | 1IQZ:A | electron transport | 48 | 52 | DILID | 5 | 97.30 | GGGHH | 5 | 1.16 |
| 268 | 1M1Q:A | electron transport | 29 | 33 | AFEFA | 5 | 54.06 | HHHHH | 8 | 1.14 |
| 269 | 1M2D:A | electron transport | 80 | 84 | EDVDE | 5 | 61.42 | GGHHH | 8 | -1.96 |
| 270 | 1M70:A | electron transport | 126 | 130 | NGVGN | 5 | 40.88 | TSCCB | 9 | -0.72 |
| 271 | 1NYK:A | electron transport | 47 | 51 | PEKEP | 5 | 82.94 | GGGCC | 5 | -2.82 |
| 272 | 1O8X:A | electron transport | 71 | 75 | DEEED | 5 | 111.76 | CCSHH | 7 | -3.50 |
| 273 | 1OFW:B | electron transport | 22 | 26 | KPNPK | 5 | 87.82 | SCCTT | 5 | -2.90 |
| 274 | 1OFW:B | electron transport | 184 | 188 | DALAD | 5 | 69.98 | CTTCS | 5 | 0.08 |
| 275 | 1RWJ:A | electron transport | 43 | 47 | KAGAK | 5 | 83.58 | STTSS | 3 | -0.92 |
| 276 | 1XER:A | electron transport | 89 | 93 | CVNVC | 5 | 53.98 | HHHHC | 4 | 1.98 |
| 277 | 256B:A | electron transport | 10 | 14 | LNDNL | 5 | 44.84 | HHHHH | 8 | -0.58 |
| 278 | 256B:A | electron transport | 75 | 79 | ALKLA | 5 | 39.76 | HHHHH | 8 | 1.46 |
| 279 | 2CAK:A | electron transport | 71 | 75 | TVDVT | 5 | 17.86 | EEEEE | 10 | 0.70 |
| 280 | 2CAK:A | electron transport | 41 | 45 | VAAAV | 5 | 3.60 | EEEES | 8 | 2.76 |
| 281 | 2CBP:A | electron transport | 6 | 10 | GGSGG | 5 | 52.46 | TGGGC | 6 | -0.48 |
| 282 | 2CBP:A | electron transport | 40 | 45 | NVVVVN | 6 | 26.97 | CEEEEC | 6 | 1.63 |
| 283 | 2FDN:A | electron transport | 14 | 18 | CEPEC | 5 | 59.90 | TGGGC | 10 | -0.72 |
| 284 | 2FDN:A | electron transport | 41 | 45 | GACAG | 5 | 30.40 | CHHHH | 10 | 1.06 |
| 285 | 2J8C:H | electron transport | 179 | 183 | LEVEL | 5 | 17.68 | EEEEE | 14 | 0.96 |
| 286 | 2J8C:L | electron transport | 201 | 205 | EKGKE | 5 | 78.82 | STTCC | 8 | -3.04 |
| 287 | 2J8C:L | electron transport | 262 | 266 | WWQWW | 5 | 85.34 | HTHHH | 8 | -1.42 |
| 288 | 2J8C:L | electron transport | 161 | 165 | GYTYG | 5 | 45.62 | HHHTS | 8 | -0.82 |
| 289 | 2J8C:L | electron transport | 250 | 254 | ITGTI | 5 | 42.94 | TBTTT | 8 | 1.44 |
| 290 | 2J8C:L | electron transport | 119 | 123 | FAFAF | 5 | 55.96 | HHHHH | 7 | 2.40 |
| 291 | 2J8C:L | electron transport | 184 | 188 | ALALA | 5 | 27.20 | HHHHH | 7 | 2.60 |
| 292 | 2OV0:A | electron transport | 6 | 10 | PSESP | 5 | 72.00 | SCSSC | 3 | -1.66 |
| 293 | 2P0B:A | electron transport | 59 | 63 | QPSPQ | 5 | 95.20 | CCCTT | 4 | -2.20 |
| 294 | 2P0B:A | electron transport | 149 | 153 | PNFNP | 5 | 82.06 | TTCCT | 4 | -1.48 |
| 295 | 3H31:A | electron transport | 49 | 54 | QCGGCQ | 6 | 61.33 | SCBEES | 10 | -0.47 |
| 296 | 3N79:A | electron transport | 150 | 154 | AVTVA | 5 | 16.54 | HHHHH | 8 | 2.26 |
| 297 | 7FD1:A | electron transport | 86 | 90 | DPLPD | 5 | 87.66 | CCCTT | 16 | -1.28 |
| 298 | 7FD1:A | electron transport | 45 | 49 | CEPEC | 5 | 56.70 | TGGGC | 16 | -0.72 |
| 299 | 7FD1:A | electron transport | 16 | 20 | CVEVC | 5 | 36.46 | HHHHC | 16 | 1.98 |
| 300 | 1C75:A | electron transport | 79 | 83 | AEAEA | 5 | 52.28 | HHHHH | 14 | -0.32 |
| 301 | 3N79:A | electron transport | 154 | 158 | ASESA | 5 | 25.78 | HHHHH | 10 | -0.30 |
| 302 | 2J8C:L | electron transport | 120 | 124 | AFAFA | 5 | 40.82 | HHHHH | 8 | 2.20 |
| 303 | 2J8C:L | electron transport | 185 | 189 | LALAL | 5 | 34.80 | HHHHH | 8 | 3.00 |
| 304 | 2J8C:L | electron transport | 31 | 36 | VGFFGV | 6 | 32.32 | CHHHHH | 7 | 2.20 |
| 305 | 1JM1:A | electron transport, oxidoreductase | 65 | 69 | QQVQQ | 5 | 75.80 | HHHHH | 12 | -1.96 |
| 306 | 1JM1:A | electron transport, oxidoreductase | 169 | 173 | HCPCH | 5 | 51.22 | ECTTT | 15 | -0.60 |
| 307 | 3ARC:J | electron transport, photosynthesis | 31 | 35 | GAYAG | 5 | 46.82 | HHSSS | 6 | 0.30 |
| 308 | 3ARC:J/3WU2 | electron transport, photosynthesis | 21 | 25 | VIVIV | 5 | 82.08 | HHHHH | 8 | 4.32 |
| 309 | 3ARC:K | electron transport, photosynthesis | 21 | 25 | LVDVL | 5 | 24.92 | HHHHG | 8 | 2.50 |
| 310 | 3ARC:u | electron transport, photosynthesis | 85 | 89 | TEVET | 5 | 62.72 | CCCCH | 8 | -0.84 |
| 311 | 3ZY7:B | endocytosis | 16 | 20 | EFTFE | 5 | 54.26 | EEEEE | 9 | -0.42 |
| 312 | 1CR5:A | endocytosis/exocytosis | 117 | 121 | IDIDI | 5 | 16.58 | EEEEE | 9 | 1.30 |
| 313 | 1KYF:A | endocytosis/exocytosis | 916 | 920 | RLTLR | 5 | 26.94 | EEEEE | 22 | -0.42 |
| 314 | 1CR5:A | endocytosis/exocytosis | 116 | 122 | SIDIDIS | 7 | 16.49 | EEEEEEE | 12 | 0.70 |
| 315 | 1A79:C | endonuclease | 43 | 47 | LSLSL | 5 | 2.20 | EEEEH | 12 | 1.96 |
| 316 | 2ABK:A | endonuclease | 49 | 53 | KATAK | 5 | 63.58 | HHHHH | 6 | -0.98 |
| 317 | 2END:A | endonuclease | 110 | 114 | SIAIS | 5 | 40.12 | HHHHH | 7 | 1.84 |
| 318 | 1E7S:A | epimerase/reductase | 245 | 249 | VGTGV | 5 | 27.74 | ESCCC | 5 | 1.38 |
| 319 | 1H4A:X | eye lens protein | 83 | 88 | HSGSH | 5 | 79.10 | CCSCC | 4 | -1.68 |
| 320 | 1NOX:A | flavoenzyme | 111 | 115 | AQKQA | 5 | 62.18 | HHHHH | 8 | -1.46 |
| 321 | 2XOD:A | flavoprotein | 111 | 115 | ERVRE | 5 | 86.52 | HHHHH | 9 | -2.36 |
| 322 | 3EDO:B | flavoprotein | 72 | 77 | DYNNYD | 6 | 61.28 | CGGGCS | 6 | -2.77 |
| 323 | 2PR5:B | flavoprotein, signaling protein | 130 | 134 | KEYEK | 5 | 104.42 | HHHHH | 8 | -3.22 |
| 324 | 2WUR:A | fluorescent protein | 31 | 35 | GEGEG | 5 | 47.10 | EEEEE | 15 | -1.64 |
| 325 | 2WUR:A | fluorescent protein | 41 | 45 | KLTLK | 5 | 40.70 | EEEEE | 15 | -0.18 |
| 326 | 3NED:A | fluorescent protein | 30 | 34 | EGEGE | 5 | 48.54 | EEEEE | 8 | -2.26 |
| 327 | 3S7O:A | fluorescent protein | 28 | 32 | PIHIP | 5 | 21.32 | CTTCC | 19 | 0.52 |
| 328 | 3NED:A | fluorescent protein | 31 | 35 | GEGEG | 5 | 30.82 | CCCCC | 8 | -1.64 |
| 329 | 2FUR:A | fmn-binding protein | 170 | 174 | DTSTD | 5 | 82.86 | CCCCS | 4 | -1.84 |
| 330 | 1MG7:B | gene regulation | 328 | 332 | AIAIA | 5 | 15.74 | HHHHH | 8 | 2.88 |
| 331 | 1K0D:B | gene regulation | 264 | 269 | AERREA | 6 | 72.65 | HHHHHH | 10 | -2.07 |
| 332 | 1K0D:B | gene regulation | 263 | 270 | LAERREAL | 8 | 64.80 | HHHHHHHH | 10 | -0.60 |
| 333 | 1MG7:B | gene regulation | 338 | 343 | SVSSVS | 6 | 25.70 | CCCEEE | 7 | 0.87 |
| 334 | 1S7Z:A | gene regulation | 106 | 110 | EEVEE | 5 | 155.28 | TTCCC | 4 | -1.96 |
| 335 | 2CPG:A | gene regulation | 5 | 9 | LTITL | 5 | 31.04 | EEEEE | 7 | 2.14 |
| 336 | 2OXL:A | gene regulation | 54 | 58 | ILSLI | 5 | 44.14 | HHHHH | 8 | 3.16 |
| 337 | 4DNU:A | gene regulation | 195 | 199 | GWGWG | 5 | 6.68 | EEECC | 9 | -0.60 |
| 338 | 4DNU:A | gene regulation | 21 | 25 | SAGAS | 5 | 10.50 | EECSS | 9 | 0.32 |
| 339 | 1MG7:B | gene regulation | 327 | 331 | IAIAI | 5 | 16.54 | HHHHH | 8 | 3.42 |
| 340 | 3A1Q:C | gene regulation/signaling protein | 103 | 107 | EEKEE | 5 | 94.84 | HHHHH | 8 | -3.58 |
| 341 | 1UOW:A | glycoprotein | 409 | 413 | VEEEV | 5 | 72.36 | CHHHH | 5 | -0.42 |
| 342 | 1UZK:A | glycoprotein | 1605 | 1609 | EDIDE | 5 | 25.28 | EECCH | 12 | -1.90 |
| 343 | 1UZK:A | glycoprotein | 1561 | 1565 | SCCCS | 5 | 3.12 | HHHTT | 12 | 1.18 |
| 344 | 2WNO:A | glycoprotein | 178 | 182 | LDFDL | 5 | 43.98 | EEEEC | 8 | 0.68 |
| 345 | 3SK2:A | griseoluteate-binding protein | 7 | 11 | PTITP | 5 | 35.20 | CCCCC | 4 | -0.02 |
| 346 | 1AGQ:C | growth factor | 69 | 73 | CSGSC | 5 | 31.46 | EESCC | 7 | 0.60 |
| 347 | 3MOL:B | heme binding protein, transport protein | 2 | 6 | SISIS | 5 | 43.04 | CCEEE | 6 | 1.32 |
| 348 | 3H8T:A | heme-binding protein | 37 | 41 | VTKTV | 5 | 72.90 | EEEEE | 7 | 0.62 |
| 349 | 3H8T:A | heme-binding protein | 90 | 94 | GKGKG | 5 | 58.64 | CSSSC | 7 | -1.80 |
| 350 | 3H8T:A | heme-binding protein | 178 | 183 | RVFFVR | 6 | 21.65 | CEEEEE | 7 | 0.83 |
| 351 | 1EXZ:B | hormone/growth factor | 252 | 256 | LSDSL | 5 | 19.64 | HHHHH | 8 | 0.50 |
| 352 | 1I1J:B | hormone/growth factor | 94 | 98 | KVDVK | 5 | 79.90 | CEEEE | 8 | -0.58 |
| 353 | 1MKK:A | hormone/growth factor | 86 | 90 | HQGQH | 5 | 108.84 | TSCCE | 4 | -2.76 |
| 354 | 1VKK:A | hormone/growth factor | 134 | 138 | LKEKL | 5 | 54.44 | HHHHH | 6 | -0.74 |
| 355 | 1VKK:A | hormone/growth factor | 56 | 60 | ELKLE | 5 | 71.90 | HHHHH | 6 | -0.66 |
| 356 | 1Z7C:A | hormone/growth factor | 168 | 172 | KDMDK | 5 | 34.78 | HHHHH | 13 | -2.58 |
| 357 | 1Z7C:A | hormone/growth factor | 51 | 55 | SFCFS | 5 | 63.38 | CCCGG | 5 | 1.30 |
| 358 | 2ARP:A | hormone/growth factor | 40 | 44 | CEGEC | 5 | 56.28 | EESCC | 6 | -0.48 |
| 359 | 2ASK:B | hormone/growth factor | 43 | 47 | CSGSC | 5 | 12.60 | EECBC | 10 | 0.60 |
| 360 | 1VKK:A | hormone/growth factor | 45 | 49 | LEDEL | 5 | 65.16 | EEEEE | 6 | -0.58 |
| 361 | 2H8G:B | hydrolase | 55 | 61 | PLGKGLP | 7 | 58.00 | TTCTTCC | 4 | -0.04 |
| 362 | 3R0V:A | hydrolase | 81 | 86 | AAGGAA | 6 | 29.25 | HTTSCE | 8 | 1.07 |
| 363 | 1H41:B | hydrolase | 293 | 299 | GQPGPQG | 7 | 42.13 | TBCCGGG | 7 | -1.63 |
| 364 | 2WOJ:C | hydrolase | 23 | 29 | VGGKGGV | 7 | 11.04 | EEESTTS | 10 | 0.41 |
| 365 | 3Q7H:N | hydrolase | 101 | 107 | GALLLAG | 7 | 4.01 | HHHHHHT | 6 | 2.03 |
| 366 | 2XFR:A | hydrolase | 360 | 365 | LVQQVL | 6 | 6.53 | HHHHHH | 11 | 1.50 |
| 367 | 2BSY:A | hydrolase | 152 | 156 | VTLTV | 5 | 60.14 | EEECS | 6 | 2.16 |
| 368 | 1I1W:A | hydrolase | 211 | 217 | SAGQGAS | 7 | 46.21 | CTTTHHH | 10 | -0.33 |
| 369 | 1XWT:A | hydrolase | 207 | 213 | AFYDYFA | 7 | 3.89 | HHHHHHH | 10 | 0.44 |
| 370 | 2OSX:A | hydrolase | 483 | 487 | TVTVT | 5 | 25.02 | EEEEE | 8 | 1.26 |
| 371 | 3JU4:A | hydrolase | 700 | 706 | SGVGVGS | 7 | 0.50 | CCSEEEE | 10 | 0.80 |
| 372 | 2IMZ:B | hydrolase | 61 | 66 | IAGGAI | 6 | 43.50 | ETTSCE | 4 | 1.97 |
| 373 | 3EQA:A | hydrolase | 96 | 101 | TSLLST | 6 | 54.50 | GGGHHH | 6 | 0.77 |
| 374 | 3BI1:A | hydrolase | 677 | 683 | DPLGLPD | 7 | 75.70 | CTTCBTT | 4 | -0.43 |
| 375 | 1Z4V:A | hydrolase | 346 | 351 | SSYYSS | 6 | 34.60 | HTTEET | 13 | -0.97 |
| 376 | 2VSM:A | hydrolase | 197 | 203 | ILKPKLI | 7 | 54.01 | CCCCEEE | 5 | 1.03 |
| 377 | 1APY:A | hydrolase | 25 | 30 | ASGGSA | 6 | 52.70 | HTTCCH | 8 | 0.20 |
| 378 | 1APY:A | hydrolase | 24 | 31 | LASGGSAL | 8 | 45.00 | HHTTCCHH | 8 | 1.10 |
| 379 | 1BXO:A | hydrolase | 201 | 205 | GSQSG | 5 | 65.76 | TTEEE | 9 | -1.18 |
| 380 | 1BXO:A | hydrolase | 277 | 281 | SGDGS | 5 | 76.52 | CSSSS | 9 | -1.18 |
| 381 | 1DEU:A | hydrolase | 35 | 39 | ASTSA | 5 | 2.24 | HHHHH | 8 | 0.26 |
| 382 | 1DEU:A | hydrolase | 133 | 137 | GSLSG | 5 | 37.30 | EEECS | 7 | 0.28 |
| 383 | 1EB6:A | hydrolase | 119 | 123 | AQDQA | 5 | 25.80 | CCCHH | 8 | -1.38 |
| 384 | 1EB6:A | hydrolase | 67 | 71 | GSTSG | 5 | 55.82 | TCSBC | 8 | -0.62 |
| 385 | 1EB6:A | hydrolase | 28 | 32 | AAEAA | 5 | 23.26 | HHHHH | 8 | 0.74 |
| 386 | 1ES9:A | hydrolase | 212 | 216 | LLRLL | 5 | 58.48 | HHHHC | 4 | 2.14 |
| 387 | 1EUW:A | hydrolase | 75 | 79 | GHKHG | 5 | 87.42 | HHHHC | 20 | -2.22 |
| 388 | 1EUW:A | hydrolase | 56 | 60 | AIHIA | 5 | 26.48 | EEECC | 20 | 1.88 |
| 389 | 1G66:A | hydrolase | 68 | 72 | VASAV | 5 | 19.46 | HHHHH | 19 | 2.24 |
| 390 | 1GKP:E | hydrolase | 446 | 450 | KGWGK | 5 | 63.62 | TTCCC | 7 | -1.90 |
| 391 | 1GKP:E | hydrolase | 248 | 252 | AAMAA | 5 | 18.70 | HHHHH | 8 | 1.82 |
| 392 | 1H41:B | hydrolase | 294 | 298 | QPGPQ | 5 | 30.82 | BCCGG | 8 | -2.12 |
| 393 | 1H41:B | hydrolase | 275 | 279 | IYSYI | 5 | 32.54 | HHHHC | 8 | 1.12 |
| 394 | 1H41:B | hydrolase | 477 | 481 | AATAA | 5 | 20.76 | HHHHH | 7 | 1.30 |
| 395 | 1H41:B | hydrolase | 311 | 315 | LAAAL | 5 | 10.58 | HHHHH | 7 | 2.60 |
| 396 | 1HT6:A | hydrolase | 371 | 375 | KVVVK | 5 | 21.70 | TEEEE | 13 | 0.96 |
| 397 | 1HX0:A | hydrolase | 280 | 284 | WGEGW | 5 | 49.26 | TTGGG | 4 | -1.22 |
| 398 | 1HX0:A | hydrolase | 106 | 110 | GAAAG | 5 | 53.56 | TCCCB | 4 | 0.92 |
| 399 | 1I1W:A | hydrolase | 102 | 106 | NTLTN | 5 | 58.68 | HHHHH | 13 | -0.92 |
| 400 | 1I1W:A | hydrolase | 212 | 216 | AGQGA | 5 | 43.80 | TTTHH | 10 | -0.14 |
| 401 | 1ITX:A | hydrolase | 300 | 304 | AASAA | 5 | 46.94 | HHHHT | 7 | 1.28 |
| 402 | 1ITX:A | hydrolase | 174 | 178 | AATAA | 5 | 47.02 | HTSHH | 7 | 1.30 |
| 403 | 1JY5:A | hydrolase | 9 | 13 | TLALT | 5 | 3.64 | EEEEE | 12 | 1.60 |
| 404 | 1K55:A | hydrolase | 45 | 49 | KSSSK | 5 | 73.44 | ESSTT | 6 | -2.04 |
| 405 | 1K5C:A | hydrolase | 196 | 200 | GISIG | 5 | 0.68 | CEEEE | 10 | 1.48 |
| 406 | 1KWF:A | hydrolase | 274 | 278 | DYKYD | 5 | 51.30 | SBSTT | 12 | -2.70 |
| 407 | 1KWF:A | hydrolase | 368 | 372 | YYGYY | 5 | 63.68 | GGTTH | 12 | -1.12 |
| 408 | 1LBU:A | hydrolase | 12 | 17 | EGSSGE | 6 | 66.60 | TTCBSH | 7 | -1.57 |
| 409 | 1LWB:A | hydrolase | 52 | 56 | PFGFP | 5 | 75.02 | TTCCC | 10 | 0.40 |
| 410 | 1LYV:A | hydrolase | 192 | 196 | EARAE | 5 | 70.40 | HHHHH | 7 | -1.58 |
| 411 | 1LYV:A | hydrolase | 263 | 267 | LQSQL | 5 | 52.10 | CGGGH | 5 | -0.04 |
| 412 | 1ME4:A | hydrolase | 130 | 134 | VAVAV | 5 | 2.54 | EEEEE | 7 | 3.24 |
| 413 | 1MJ5:A | hydrolase | 102 | 106 | VVLVV | 5 | 0.34 | EEEEE | 29 | 4.12 |
| 414 | 1NF8:A | hydrolase | 175 | 179 | DAIAD | 5 | 3.44 | EEEEC | 11 | 0.22 |
| 415 | 1NOW:A | hydrolase | 475 | 479 | QKQKQ | 5 | 108.56 | HHHHH | 7 | -3.66 |
| 416 | 1NQJ:B | hydrolase | 895 | 899 | EKLKE | 5 | 107.02 | CCHHH | 6 | -2.20 |
| 417 | 1NUY:A | hydrolase | 1196 | 1200 | VDRDV | 5 | 80.16 | EECSC | 5 | -0.62 |
| 418 | 1NWW:A | hydrolase | 16 | 20 | AAGAA | 5 | 31.44 | TTTCC | 3 | 1.36 |
| 419 | 1NZ0:A | hydrolase | 10 | 14 | RLRLR | 5 | 68.90 | CCCHH | 5 | -1.18 |
| 420 | 1NZ0:A | hydrolase | 108 | 112 | LLNLL | 5 | 19.48 | HHHHH | 6 | 2.34 |
| 421 | 1O4Y:A | hydrolase | 225 | 229 | RTVTR | 5 | 65.02 | EEECH | 8 | -1.24 |
| 422 | 1O4Y:A | hydrolase | 124 | 128 | IKAKI | 5 | 29.40 | EEEEC | 8 | 0.60 |
| 423 | 1O7J:C | hydrolase | 248 | 252 | AGMGA | 5 | 5.00 | EEBTT | 22 | 0.94 |
| 424 | 1OB9:A | hydrolase | 60 | 64 | KVKVK | 5 | 67.56 | EEEEC | 8 | -0.66 |
| 425 | 1OC7:A | hydrolase | 309 | 313 | ANYNA | 5 | 11.64 | TCCCC | 13 | -0.94 |
| 426 | 1OD3:A | hydrolase | 94 | 98 | GSPSG | 5 | 52.62 | SSTTS | 4 | -0.80 |
| 427 | 1OI0:A | hydrolase | 116 | 120 | VELEV | 5 | 66.30 | ECCEE | 7 | 1.04 |
| 428 | 1PQ5:A | hydrolase | 82 | 86 | SSLSS | 5 | 40.38 | ECEEE | 12 | 0.12 |
| 429 | 1PQ5:A | hydrolase | 136 | 140 | AGWGA | 5 | 3.56 | EESCC | 12 | 0.38 |
| 430 | 1Q33:A | hydrolase | 159 | 164 | RGLLGR | 6 | 20.00 | EETCSS | 8 | -0.37 |
| 431 | 1QHH:D | hydrolase | 592 | 596 | EDDDE | 5 | 92.38 | TCHHH | 7 | -3.50 |
| 432 | 1QNR:A | hydrolase | 253 | 257 | WGNGW | 5 | 17.54 | HHHHH | 10 | -1.22 |
| 433 | 1QQ5:B | hydrolase | 155 | 160 | LVEEVL | 6 | 51.02 | HHHHHH | 6 | 1.50 |
| 434 | 1QWY:A | hydrolase | 131 | 135 | QYGYQ | 5 | 79.12 | HHCCC | 5 | -2.00 |
| 435 | 1QWY:A | hydrolase | 98 | 102 | NANAN | 5 | 65.04 | SCSSC | 5 | -1.38 |
| 436 | 1QWZ:A | hydrolase | 174 | 178 | KTTTK | 5 | 77.10 | EEESS | 5 | -1.98 |
| 437 | 1QZM:A | hydrolase | 528 | 532 | IIGII | 5 | 10.96 | HHHHH | 7 | 3.52 |
| 438 | 1RA0:A | hydrolase | 128 | 132 | ATLTA | 5 | 37.46 | TTCHH | 4 | 1.20 |
| 439 | 1RRE:E | hydrolase | 719 | 723 | LKEKL | 5 | 49.14 | HHHHH | 6 | -0.74 |
| 440 | 1RTQ:A | hydrolase | 115 | 119 | ADDDA | 5 | 1.86 | TTTTH | 13 | -1.38 |
| 441 | 1RTQ:A | hydrolase | 8 | 12 | ATVTA | 5 | 52.66 | HHHHH | 10 | 1.28 |
| 442 | 1RTQ:A | hydrolase | 22 | 26 | ITGTI | 5 | 13.06 | HHHHH | 10 | 1.44 |
| 443 | 1RTQ:A | hydrolase | 56 | 60 | LSASL | 5 | 50.04 | HHTTS | 13 | 1.56 |
| 444 | 1S5P:A | hydrolase | 238 | 242 | ELNLE | 5 | 52.96 | EEESS | 11 | -0.58 |
| 445 | 1SO2:D | hydrolase | 821 | 825 | HDYDH | 5 | 15.64 | TTTTC | 12 | -2.94 |
| 446 | 1SO2:D | hydrolase | 906 | 910 | NAKAN | 5 | 43.80 | HHHTT | 12 | -1.46 |
| 447 | 1TQH:A | hydrolase | 92 | 96 | GLSLG | 5 | 6.10 | EETHH | 10 | 1.20 |
| 448 | 1U0A:D | hydrolase | 1005 | 1009 | QIDIQ | 5 | 10.60 | EEEEE | 9 | -0.30 |
| 449 | 1U4G:A | hydrolase | 263 | 267 | SNYNS | 5 | 34.96 | CCHHH | 9 | -1.98 |
| 450 | 1U4G:A | hydrolase | 4 | 8 | GGPGG | 5 | 6.52 | BEEEE | 9 | -0.64 |
| 451 | 1U4G:A | hydrolase | 292 | 296 | TVGVT | 5 | 34.88 | HTTCC | 9 | 1.32 |
| 452 | 1UAS:A | hydrolase | 130 | 134 | DNCND | 5 | 33.64 | ECCCC | 10 | -2.30 |
| 453 | 1UAS:A | hydrolase | 87 | 91 | GLKLG | 5 | 26.64 | TCEEE | 10 | 0.58 |
| 454 | 1UAS:A | hydrolase | 311 | 315 | ATITA | 5 | 28.24 | EEEEE | 8 | 1.34 |
| 455 | 1UCD:A | hydrolase | 61 | 65 | LQSQL | 5 | 48.56 | GHHHH | 7 | -0.04 |
| 456 | 1UF5:B | hydrolase | 260 | 264 | LTTTL | 5 | 61.62 | ECCSS | 5 | 1.10 |
| 457 | 1UFO:D | hydrolase | 63 | 67 | GEREG | 5 | 93.46 | TTSSC | 6 | -2.46 |
| 458 | 1UFO:D | hydrolase | 77 | 82 | YVEEVY | 6 | 26.62 | HHHHHH | 7 | -0.20 |
| 459 | 1UFO:D | hydrolase | 25 | 29 | ALLLA | 5 | 0.50 | EEEEE | 7 | 3.00 |
| 460 | 1UFO:D | hydrolase | 76 | 83 | RYVEEVYR | 8 | 44.65 | THHHHHHH | 12 | -1.28 |
| 461 | 1UG6:A | hydrolase | 197 | 201 | LGHGL | 5 | 5.80 | HHHHH | 7 | 0.72 |
| 462 | 1UG6:A | hydrolase | 230 | 234 | AVDVA | 5 | 20.82 | HHHHH | 7 | 1.70 |
| 463 | 1UG6:A | hydrolase | 138 | 142 | AFAFA | 5 | 17.56 | HHHHH | 7 | 2.20 |
| 464 | 1UI0:A | hydrolase | 41 | 45 | EGPGE | 5 | 41.74 | SCCCH | 7 | -1.88 |
| 465 | 1UI0:A | hydrolase | 7 | 11 | QAQAQ | 5 | 52.74 | HHHHT | 7 | -1.38 |
| 466 | 1UOC:A | hydrolase | 170 | 174 | LINIL | 5 | 16.92 | HHHHH | 7 | 2.62 |
| 467 | 1UOC:A | hydrolase | 252 | 256 | LLMLL | 5 | 4.94 | HHHHH | 7 | 3.42 |
| 468 | 1UUQ:A | hydrolase | 100 | 104 | NGFGN | 5 | 57.80 | SSTTC | 2 | -1.00 |
| 469 | 1UWC:A | hydrolase | 107 | 111 | VQDQV | 5 | 42.30 | HHHHH | 8 | -0.42 |
| 470 | 1VJN:A | hydrolase | 195 | 199 | EKPKE | 5 | 111.40 | SCCCS | 5 | -3.28 |
| 471 | 1VJN:A | hydrolase | 162 | 166 | YKTKY | 5 | 99.90 | CCCSS | 5 | -2.22 |
| 472 | 1VJN:A | hydrolase | 177 | 181 | FLKLF | 5 | 50.56 | HHTTS | 5 | 1.86 |
| 473 | 1VMG:A | hydrolase | 39 | 43 | LAEAL | 5 | 57.94 | HHHHH | 7 | 1.54 |
| 474 | 1W0N:A | hydrolase | 79 | 83 | GVTVG | 5 | 55.52 | TEEEE | 4 | 1.38 |
| 475 | 1X2I:A | hydrolase | 49 | 53 | EGIGE | 5 | 66.46 | TTCCH | 4 | -0.66 |
| 476 | 1X2I:A | hydrolase | 2 | 6 | ALTLA | 5 | 73.00 | CCCHH | 4 | 2.10 |
| 477 | 1X9D:A | hydrolase | 390 | 394 | TSDST | 5 | 24.62 | CSEEE | 10 | -1.30 |
| 478 | 1XJU:A | hydrolase | 171 | 175 | REKER | 5 | 49.72 | HHHHH | 12 | -3.98 |
| 479 | 1XM8:B | hydrolase | 92 | 96 | KDGDK | 5 | 86.24 | CTTCE | 6 | -3.04 |
| 480 | 1XMK:A | hydrolase | 299 | 303 | IKEKI | 5 | 52.66 | HHHHH | 8 | -0.46 |
| 481 | 1XMK:A | hydrolase | 315 | 319 | ALNLA | 5 | 42.86 | HHHHH | 8 | 1.54 |
| 482 | 1XWT:A | hydrolase | 208 | 212 | FYDYF | 5 | 5.40 | HHHHH | 10 | -0.10 |
| 483 | 1Y43:B | hydrolase | 47 | 51 | DYAYD | 5 | 91.52 | SCCEE | 7 | -1.56 |
| 484 | 1Y43:B | hydrolase | 135 | 140 | TSGGST | 6 | 59.32 | EETTEE | 7 | -0.63 |
| 485 | 1YB0:A | hydrolase | 79 | 83 | NGPGN | 5 | 17.34 | SSHHH | 9 | -1.88 |
| 486 | 1YKS:A | hydrolase | 467 | 471 | RNPNR | 5 | 80.80 | CCTTC | 6 | -3.52 |
| 487 | 1YKS:A | hydrolase | 475 | 479 | SYYYS | 5 | 18.14 | EEEEC | 6 | -1.10 |
| 488 | 1YRB:A | hydrolase | 122 | 126 | LPYPL | 5 | 46.04 | SSSCE | 5 | 0.62 |
| 489 | 1Z4V:A | hydrolase | 545 | 549 | LSSSL | 5 | 68.36 | EECSS | 9 | 1.04 |
| 490 | 1ZPS:B | hydrolase | 65 | 69 | KLWLK | 5 | 53.64 | EEEET | 7 | -0.22 |
| 491 | 1ZPS:B | hydrolase | 81 | 85 | DVLVD | 5 | 14.14 | EEEEC | 7 | 1.04 |
| 492 | 1ZWX:A | hydrolase | 204 | 208 | EQMQE | 5 | 34.36 | HHHHH | 12 | -2.42 |
| 493 | 2A0M:A | hydrolase | 71 | 75 | NLELN | 5 | 108.10 | ETTTT | 3 | -0.58 |
| 494 | 2AS9:A | hydrolase | 33 | 38 | NTIITN | 6 | 0.33 | TEEEEC | 5 | 0.10 |
| 495 | 2AS9:A | hydrolase | 104 | 108 | AFNFA | 5 | 39.38 | CCCBC | 5 | 1.14 |
| 496 | 2BF6:A | hydrolase | 370 | 374 | YLCLY | 5 | 21.68 | EEEEE | 8 | 1.50 |
| 497 | 2BHU:A | hydrolase | 340 | 344 | GEQEG | 5 | 73.76 | CCCSG | 8 | -2.26 |
| 498 | 2BJI:B | hydrolase | 2204 | 2208 | AAGAA | 5 | 13.06 | HTTSS | 6 | 1.36 |
| 499 | 2BO9:B | hydrolase | 35 | 39 | QKVKQ | 5 | 62.44 | EEEEE | 8 | -2.12 |
| 500 | 2BSY:A | hydrolase | 149 | 153 | TVSVT | 5 | 52.18 | EEEEE | 8 | 1.24 |
| 501 | 2C0H:A | hydrolase | 55 | 59 | SKGKS | 5 | 84.90 | HHHHH | 7 | -1.96 |
| 502 | 2C0H:A | hydrolase | 186 | 190 | ESSSE | 5 | 77.78 | CCCSS | 5 | -1.88 |
| 503 | 2C0H:A | hydrolase | 244 | 248 | ADTDA | 5 | 23.38 | GBCSS | 5 | -0.82 |
| 504 | 2CDO:C | hydrolase | 126 | 130 | NWQWN | 5 | 36.54 | SBCCE | 14 | -2.46 |
| 505 | 2CI1:A | hydrolase | 24 | 29 | LAQQAL | 6 | 65.00 | HHHHSC | 5 | 0.70 |
| 506 | 2CI1:A | hydrolase | 59 | 64 | LQVVQL | 6 | 59.40 | CEEEEE | 5 | 1.50 |
| 507 | 2CKW:A | hydrolase | 386 | 390 | PTNTP | 5 | 69.14 | CCSSC | 9 | -1.62 |
| 508 | 2CKW:A | hydrolase | 163 | 167 | KLALK | 5 | 23.88 | EEEEC | 9 | 0.32 |
| 509 | 2CKW:A | hydrolase | 224 | 228 | VQMQV | 5 | 50.80 | HHHHH | 6 | 0.66 |
| 510 | 2CWZ:D | hydrolase | 56 | 60 | EEGEE | 5 | 69.98 | CTTEE | 6 | -2.88 |
| 511 | 2CWZ:D | hydrolase | 71 | 75 | LASAL | 5 | 37.54 | CSCCC | 6 | 2.08 |
| 512 | 2D0B:A | hydrolase | 58 | 62 | AEQEA | 5 | 47.54 | HHHHH | 9 | -1.38 |
| 513 | 2D0B:A | hydrolase | 56 | 64 | KAAEQEAAK | 9 | 70.80 | TTHHHHHHT | 8 | -1.23 |
| 514 | 2D0B:A | hydrolase | 194 | 198 | AEPEA | 5 | 50.20 | CCCSE | 8 | -1.00 |
| 515 | 2D0B:A | hydrolase | 277 | 282 | ARIIRA | 6 | 52.10 | HHHHHH | 9 | 0.60 |
| 516 | 2D0B:A | hydrolase | 233 | 237 | SVHVS | 5 | 25.24 | GTCHH | 8 | 0.72 |
| 517 | 2D0B:A | hydrolase | 142 | 146 | IAPAI | 5 | 19.72 | HHHHH | 9 | 2.20 |
| 518 | 2DFB:A | hydrolase | 163 | 167 | GLTLG | 5 | 34.20 | TCCCC | 7 | 1.22 |
| 519 | 2E4T:A | hydrolase | 11 | 15 | DIRID | 5 | 51.62 | EEEEE | 10 | -0.50 |
| 520 | 2E4T:A | hydrolase | 433 | 437 | SAYAS | 5 | 7.72 | EEEEE | 10 | 0.14 |
| 521 | 2E4T:A | hydrolase | 36 | 40 | ATVTA | 5 | 22.98 | SCCSE | 11 | 1.28 |
| 522 | 2FVV:A | hydrolase | 55 | 59 | PEEEP | 5 | 88.62 | TTCCH | 6 | -2.74 |
| 523 | 2GEF:A | hydrolase | 696 | 700 | ALALA | 5 | 2.40 | HHHHH | 7 | 2.60 |
| 524 | 2GMW:B | hydrolase | 89 | 93 | ETLTE | 5 | 65.52 | HHHHH | 8 | -0.92 |
| 525 | 2GMW:B | hydrolase | 163 | 167 | AAVAA | 5 | 31.74 | HHHHT | 6 | 2.28 |
| 526 | 2GRC:A | hydrolase | 1460 | 1464 | KKMKK | 5 | 72.84 | HHHHH | 8 | -2.74 |
| 527 | 2H0E:B | hydrolase | 98 | 102 | ADPDA | 5 | 66.44 | CCTTS | 4 | -1.00 |
| 528 | 2H5C:A | hydrolase | 207 | 211 | GQAQG | 5 | 7.84 | CBEEE | 20 | -1.20 |
| 529 | 2H8G:B | hydrolase | 56 | 60 | LGKGL | 5 | 61.94 | TCTTC | 4 | 0.58 |
| 530 | 2HC1:A | hydrolase | 1895 | 1899 | PGAGP | 5 | 49.96 | SCCCC | 7 | -0.44 |
| 531 | 2HC1:A | hydrolase | 1777 | 1781 | GPLPG | 5 | 37.36 | CCCTT | 7 | -0.04 |
| 532 | 2HDS:B | hydrolase | 140 | 144 | PAWAP | 5 | 62.78 | CSSCT | 8 | -0.10 |
| 533 | 2HDS:B | hydrolase | 110 | 114 | ATYTA | 5 | 1.22 | HHTCS | 8 | 0.18 |
| 534 | 2HYK:A | hydrolase | 22 | 26 | APDPA | 5 | 55.16 | CCCTT | 4 | -0.62 |
| 535 | 2HYK:A | hydrolase | 238 | 242 | YVRVY | 5 | 30.70 | EEEEE | 11 | 0.26 |
| 536 | 2HZY:A | hydrolase | 198 | 202 | MELEM | 5 | 1.46 | CBEEE | 13 | 0.12 |
| 537 | 2HZY:A | hydrolase | 299 | 303 | LSVSL | 5 | 10.80 | EEEEE | 8 | 2.04 |
| 538 | 2I5I:B | hydrolase | 166 | 170 | YLELY | 5 | 58.08 | GGGGT | 7 | 0.30 |
| 539 | 2I5I:B | hydrolase | 64 | 68 | LTLTL | 5 | 2.40 | ECSCC | 7 | 2.00 |
| 540 | 2I5I:B | hydrolase | 143 | 147 | ALALA | 5 | 9.16 | HHHHH | 9 | 2.60 |
| 541 | 2I7D:A | hydrolase | 26 | 30 | FRRRF | 5 | 94.46 | HHHHS | 5 | -1.58 |
| 542 | 2IXD:B | hydrolase | 89 | 93 | IVKVI | 5 | 16.04 | HHHHH | 9 | 2.70 |
| 543 | 2J9O:D | hydrolase | 22 | 26 | QQIQQ | 5 | 83.00 | HHHHH | 8 | -1.90 |
| 544 | 2J9O:D | hydrolase | 124 | 128 | NTATN | 5 | 4.12 | HHHHH | 8 | -1.32 |
| 545 | 2JC9:A | hydrolase | 442 | 446 | RSGSR | 5 | 44.00 | EETTE | 2 | -2.20 |
| 546 | 2JC9:A | hydrolase | 269 | 273 | GPKPG | 5 | 71.70 | SSSTT | 2 | -1.58 |
| 547 | 2JC9:A | hydrolase | 315 | 319 | GTYTG | 5 | 63.80 | SCCCS | 2 | -0.70 |
| 548 | 2JE6:I | hydrolase | 161 | 165 | PVKVP | 5 | 32.18 | GGGHH | 7 | 0.26 |
| 549 | 2JE6:I | hydrolase | 205 | 209 | EILIE | 5 | 73.84 | CCTHH | 7 | 1.16 |
| 550 | 2NRR:A | hydrolase | 471 | 475 | PHDHP | 5 | 86.50 | CTTCH | 6 | -2.62 |
| 551 | 2NT0:D | hydrolase | 382 | 386 | NLALN | 5 | 8.46 | ESCBC | 5 | 0.48 |
| 552 | 2NT0:D | hydrolase | 164 | 168 | ALQLA | 5 | 53.64 | HHHHC | 5 | 1.54 |
| 553 | 2OB3:B | hydrolase | 232 | 236 | DDTDD | 5 | 43.46 | GGCCC | 10 | -2.94 |
| 554 | 2OB3:B | hydrolase | 266 | 270 | ASASA | 5 | 44.90 | HHHHH | 6 | 0.76 |
| 555 | 2OIK:D | hydrolase | 96 | 100 | VHWHV | 5 | 13.38 | CEEEE | 9 | 0.22 |
| 556 | 2OKV:D | hydrolase | 23 | 27 | IGRGI | 5 | 35.58 | ESSEE | 8 | 0.74 |
| 557 | 2OS0:A | hydrolase | 109 | 113 | GEGEG | 5 | 49.18 | TTCCC | 5 | -1.64 |
| 558 | 2OS0:A | hydrolase | 117 | 121 | VDRDV | 5 | 76.40 | CCSCC | 5 | -0.62 |
| 559 | 2OSX:A | hydrolase | 220 | 224 | ADNDA | 5 | 66.14 | TTCTT | 4 | -1.38 |
| 560 | 2OXC:A | hydrolase | 250 | 254 | LANAL | 5 | 24.40 | HHHHH | 7 | 1.54 |
| 561 | 2OXC:A | hydrolase | 66 | 70 | SLLLS | 5 | 37.90 | GGTCC | 7 | 1.96 |
| 562 | 2PNL:J | hydrolase | 674 | 678 | AAIAA | 5 | 15.10 | HHHHH | 7 | 2.34 |
| 563 | 2POF:B | hydrolase | 45 | 49 | NQQQN | 5 | 86.06 | HHHHH | 7 | -3.50 |
| 564 | 2PTH:A | hydrolase | 108 | 112 | GGHGG | 5 | 60.16 | CCCTT | 3 | -0.96 |
| 565 | 2PW8:I | hydrolase | 41 | 45 | TGEGT | 5 | 82.04 | ESCCE | 6 | -1.14 |
| 566 | 2Q0S:A | hydrolase | 169 | 173 | SALAS | 5 | 24.98 | HHHHH | 8 | 1.16 |
| 567 | 2QE8:B | hydrolase | 137 | 141 | DPAPD | 5 | 26.64 | ECCSG | 11 | -1.68 |
| 568 | 2QSW:A | hydrolase | 337 | 341 | VETEV | 5 | 37.14 | CEEEE | 9 | 0.14 |
| 569 | 2QVP:A | hydrolase | 176 | 180 | TYVYT | 5 | 7.72 | BEEEE | 5 | 0.04 |
| 570 | 2QVP:A | hydrolase | 103 | 107 | VLPLV | 5 | 2.86 | EECCS | 5 | 2.88 |
| 571 | 2R9G:P | hydrolase | 319 | 323 | DVVVD | 5 | 4.88 | HHHHH | 10 | 1.12 |
| 572 | 2RI0:B | hydrolase | 221 | 225 | VILIV | 5 | 3.70 | EEEEE | 9 | 4.24 |
| 573 | 2UXY:A | hydrolase | 203 | 207 | MAKAM | 5 | 34.30 | HHHHH | 6 | 0.70 |
| 574 | 2UY2:A | hydrolase | 157 | 161 | ENNNE | 5 | 71.68 | CSSCC | 7 | -3.50 |
| 575 | 2V3G:A | hydrolase | 195 | 199 | SQWQS | 5 | 61.46 | CCCCC | 8 | -1.90 |
| 576 | 2V3I:A | hydrolase | 167 | 171 | YGTGY | 5 | 11.22 | GTCCC | 13 | -0.82 |
| 577 | 2V3I:A | hydrolase | 319 | 323 | GSYSG | 5 | 58.86 | TTEEE | 13 | -0.74 |
| 578 | 2VB1:A | hydrolase | 43 | 47 | TNRNT | 5 | 100.78 | EEECT | 11 | -2.58 |
| 579 | 2VFO:A | hydrolase | 459 | 463 | AGSGA | 5 | 1.58 | SSCSE | 5 | 0.40 |
| 580 | 2VFO:A | hydrolase | 124 | 128 | SLKLS | 5 | 47.86 | EEEGG | 5 | 0.42 |
| 581 | 2VFO:A | hydrolase | 136 | 140 | AASAA | 5 | 24.90 | HHHHC | 5 | 1.28 |
| 582 | 2VPT:A | hydrolase | 128 | 132 | LNGNL | 5 | 79.68 | HHCCC | 6 | 0.04 |
| 583 | 2VSM:A | hydrolase | 198 | 202 | LKPKL | 5 | 70.84 | CCCEE | 4 | -0.36 |
| 584 | 2VYO:A | hydrolase | 202 | 206 | GKDKG | 5 | 63.12 | HHHHT | 12 | -2.42 |
| 585 | 2VYO:A | hydrolase | 56 | 60 | TFSFT | 5 | 4.50 | EEEEC | 12 | 0.68 |
| 586 | 2VZP:B | hydrolase | 97 | 101 | TVRVT | 5 | 58.46 | EEEEE | 6 | 0.50 |
| 587 | 2W39:A | hydrolase | 246 | 250 | NIIIN | 5 | 5.78 | EEEEE | 10 | 1.30 |
| 588 | 2W3Z:A | hydrolase | 166 | 170 | HSFSH | 5 | 23.42 | CCSSC | 10 | -1.04 |
| 589 | 2W91:A | hydrolase | 230 | 234 | SNTNS | 5 | 19.04 | ECCSC | 14 | -1.86 |
| 590 | 2WAO:A | hydrolase | 167 | 171 | NASAN | 5 | 29.84 | TEEEE | 5 | -0.84 |
| 591 | 2WAO:A | hydrolase | 106 | 110 | GFDFG | 5 | 43.30 | EEECT | 5 | 0.26 |
| 592 | 2WAO:A | hydrolase | 226 | 230 | FSTSF | 5 | 59.04 | HSSSC | 5 | 0.66 |
| 593 | 2WHL:A | hydrolase | 94 | 98 | DSRSD | 5 | 61.34 | CCHHH | 7 | -2.62 |
| 594 | 2WHL:A | hydrolase | 153 | 157 | LTHTL | 5 | 13.56 | CCSCE | 7 | 0.60 |
| 595 | 2WOJ:C | hydrolase | 24 | 28 | GGKGG | 5 | 15.40 | EESTT | 7 | -1.10 |
| 596 | 2X5X:A | hydrolase | 29 | 33 | GGFGG | 5 | 3.68 | CEEEC | 9 | 0.24 |
| 597 | 2XF3:A | hydrolase | 248 | 253 | REVVER | 6 | 70.87 | HHHHHH | 8 | -1.27 |
| 598 | 2XF3:A | hydrolase | 14 | 18 | AALAA | 5 | 43.80 | HHHHH | 8 | 2.20 |
| 599 | 2XFR:A | hydrolase | 461 | 465 | EISIE | 5 | 80.00 | CCCHH | 12 | 0.24 |
| 600 | 2XFR:A | hydrolase | 9 | 13 | YVQVY | 5 | 3.66 | CCEEE | 12 | 0.46 |
| 601 | 2XXN:A | hydrolase | 70 | 74 | TFQFT | 5 | 60.24 | EEEEE | 7 | 0.14 |
| 602 | 2Y1K:A | hydrolase | 172 | 176 | QLALQ | 5 | 25.52 | HHHHH | 8 | 0.48 |
| 603 | 2Y4S:A | hydrolase | 541 | 545 | DRIRD | 5 | 20.64 | HHHHH | 9 | -2.30 |
| 604 | 2Y4S:A | hydrolase | 249 | 253 | DSFSD | 5 | 48.34 | SCGGG | 4 | -1.16 |
| 605 | 2Y6H:A | hydrolase | 85 | 89 | TYTYT | 5 | 22.22 | EEEEE | 11 | -0.94 |
| 606 | 2Y6H:A | hydrolase | 62 | 66 | NGVGN | 5 | 53.02 | CCCCS | 7 | -0.72 |
| 607 | 2Y6H:A | hydrolase | 129 | 133 | TFEFT | 5 | 55.30 | EEEEE | 11 | 0.14 |
| 608 | 2Y6U:A | hydrolase | 48 | 52 | RTATR | 5 | 65.58 | TTCEE | 12 | -1.72 |
| 609 | 2Y6U:A | hydrolase | 303 | 307 | QLFLQ | 5 | 50.38 | HHHHH | 6 | 0.68 |
| 610 | 2YB1:A | hydrolase | 25 | 29 | RAAAR | 5 | 64.22 | HHHTT | 6 | -0.72 |
| 611 | 2YD6:A | hydrolase | 59 | 63 | KKGKK | 5 | 125.84 | ETTEE | 2 | -3.20 |
| 612 | 2YFO:A | hydrolase | 361 | 366 | DMVVMD | 6 | 7.52 | CEEEEC | 12 | 0.87 |
| 613 | 2YHG:A | hydrolase | 761 | 765 | GGWGG | 5 | 2.62 | CCTTC | 7 | -0.50 |
| 614 | 2Z72:A | hydrolase | 263 | 267 | ETFTE | 5 | 92.86 | TTCCH | 8 | -1.12 |
| 615 | 2Z72:A | hydrolase | 314 | 319 | ELLLLE | 6 | 17.60 | CEEEEE | 8 | 1.37 |
| 616 | 2Z72:A | hydrolase | 86 | 90 | LLTLL | 5 | 15.08 | HHHHH | 9 | 2.90 |
| 617 | 2ZEX:A | hydrolase | 137 | 142 | YVDDVY | 6 | 22.62 | EEEEEE | 12 | -0.20 |
| 618 | 2ZEX:A | hydrolase | 136 | 143 | LYVDDVYL | 8 | 17.09 | EEEEEEEE | 15 | 0.80 |
| 619 | 2ZKM:X | hydrolase | 556 | 560 | SFEFS | 5 | 53.14 | CHHHH | 10 | 0.10 |
| 620 | 2ZKM:X | hydrolase | 389 | 393 | EAIAE | 5 | 24.46 | HHHHH | 10 | 0.22 |
| 621 | 2ZKM:X | hydrolase | 457 | 461 | KILIK | 5 | 14.12 | CEEEE | 10 | 1.00 |
| 622 | 2ZKM:X | hydrolase | 130 | 134 | VLALV | 5 | 23.56 | HHHHH | 10 | 3.56 |
| 623 | 3A72:A | hydrolase | 367 | 371 | VSVSV | 5 | 1.24 | EEEEE | 12 | 2.20 |
| 624 | 3AKH:A | hydrolase | 385 | 389 | EVWVE | 5 | 34.04 | EEEEE | 13 | 0.10 |
| 625 | 3AKH:A | hydrolase | 226 | 230 | SASAS | 5 | 38.64 | EEETT | 5 | 0.24 |
| 626 | 3AMN:B | hydrolase | 96 | 100 | SVEVS | 5 | 13.94 | EEEEE | 10 | 0.66 |
| 627 | 3AON:A | hydrolase | 88 | 92 | VSISV | 5 | 17.92 | CEEEE | 7 | 2.26 |
| 628 | 3B7E:A | hydrolase | 325 | 329 | NPRPN | 5 | 37.64 | SSCCS | 8 | -2.94 |
| 629 | 3B7E:A | hydrolase | 323 | 331 | GDNPRPNDG | 9 | 31.41 | CSSSCCSSC | 8 | -2.50 |
| 630 | 3B7E:A | hydrolase | 105 | 109 | GIRIG | 5 | 41.36 | HHHHH | 9 | 0.74 |
| 631 | 3B7E:A | hydrolase | 211 | 215 | ITDTI | 5 | 24.10 | EEEEE | 9 | 0.82 |
| 632 | 3B7F:A | hydrolase | 64 | 68 | REPER | 5 | 65.38 | SSTTC | 7 | -3.52 |
| 633 | 3BB7:A | hydrolase | 321 | 325 | NWGWN | 5 | 24.20 | ECSST | 9 | -1.84 |
| 634 | 3BB7:A | hydrolase | 326 | 330 | GDVDG | 5 | 22.88 | TTTCE | 9 | -0.72 |
| 635 | 3BB7:A | hydrolase | 156 | 160 | ATATA | 5 | 0.86 | HHHHH | 9 | 0.80 |
| 636 | 3BF7:A | hydrolase | 111 | 115 | AIDIA | 5 | 0.96 | EESCC | 11 | 1.82 |
| 637 | 3BI1:A | hydrolase | 212 | 216 | NKVKN | 5 | 23.32 | HHHHH | 12 | -2.12 |
| 638 | 3BI1:A | hydrolase | 678 | 682 | PLGLP | 5 | 69.58 | TTCBT | 4 | 0.80 |
| 639 | 3BMX:A | hydrolase | 245 | 249 | ERLRE | 5 | 52.94 | HHHHH | 8 | -2.44 |
| 640 | 3BMX:A | hydrolase | 260 | 264 | DAGAD | 5 | 34.20 | HTTCC | 10 | -0.76 |
| 641 | 3BMX:A | hydrolase | 630 | 634 | LGYGL | 5 | 34.40 | TTCCB | 10 | 1.10 |
| 642 | 3BPT:A | hydrolase | 357 | 361 | PKWKP | 5 | 50.68 | CCCSS | 6 | -2.38 |
| 643 | 3BPT:A | hydrolase | 233 | 237 | KSPSK | 5 | 61.86 | SSCCH | 6 | -2.20 |
| 644 | 3BVU:A | hydrolase | 1041 | 1045 | SSHSS | 5 | 40.36 | EEECC | 7 | -1.28 |
| 645 | 3BVU:A | hydrolase | 705 | 709 | HVPVH | 5 | 51.88 | CEEEE | 7 | 0.08 |
| 646 | 3BVU:A | hydrolase | 498 | 502 | QMVMQ | 5 | 19.00 | HHHHH | 11 | 0.20 |
| 647 | 3C7X:A | hydrolase | 339 | 343 | RWFWR | 5 | 57.64 | TEEEE | 9 | -1.60 |
| 648 | 3C7X:A | hydrolase | 406 | 410 | LGRGL | 5 | 33.94 | TCBSC | 9 | 0.46 |
| 649 | 3CDX:D | hydrolase | 196 | 200 | STFTS | 5 | 30.72 | CCHHH | 7 | -0.04 |
| 650 | 3CM3:A | hydrolase | 1082 | 1086 | DTTTD | 5 | 70.40 | SSSCC | 8 | -1.82 |
| 651 | 3CM3:A | hydrolase | 1008 | 1012 | LYKYL | 5 | 92.48 | HHHHH | 7 | 0.22 |
| 652 | 3CNE:D | hydrolase | 52 | 57 | IVDDVI | 6 | 30.10 | ECSEEG | 9 | 1.73 |
| 653 | 3CP7:A | hydrolase | 166 | 171 | GGSSGG | 6 | 12.92 | TTCTTC | 6 | -0.53 |
| 654 | 3CP7:A | hydrolase | 105 | 109 | VQQQV | 5 | 48.66 | HHHHH | 4 | -0.42 |
| 655 | 3CP7:A | hydrolase | 131 | 135 | PAAAP | 5 | 58.66 | CCSTT | 6 | 0.44 |
| 656 | 3CPX:A | hydrolase | 294 | 298 | LYKYL | 5 | 32.70 | HHHHH | 11 | 0.22 |
| 657 | 3CPX:A | hydrolase | 154 | 158 | ALELA | 5 | 11.36 | HHHHT | 7 | 1.54 |
| 658 | 3CT5:A | hydrolase | 63 | 67 | LGFGL | 5 | 8.96 | SCBTT | 9 | 1.92 |
| 659 | 3CTZ:A | hydrolase | 485 | 489 | HGTGH | 5 | 18.90 | SCSEE | 5 | -1.58 |
| 660 | 3CTZ:A | hydrolase | 107 | 111 | LVSVL | 5 | 40.04 | HHHHC | 5 | 3.04 |
| 661 | 3CU9:A | hydrolase | 95 | 99 | YYLYY | 5 | 4.66 | EEEEE | 12 | -0.28 |
| 662 | 3CZ8:A | hydrolase | 378 | 382 | RSMSR | 5 | 54.32 | HHHHH | 10 | -1.74 |
| 663 | 3CZ8:A | hydrolase | 257 | 261 | GYDYG | 5 | 11.48 | TCCHH | 7 | -1.38 |
| 664 | 3CZ8:A | hydrolase | 229 | 233 | LRDRL | 5 | 44.56 | HHHHH | 10 | -0.98 |
| 665 | 3D59:A | hydrolase | 420 | 424 | TNINT | 5 | 57.70 | CSCCC | 5 | -0.78 |
| 666 | 3D59:A | hydrolase | 395 | 399 | HLGLH | 5 | 56.32 | HHTCC | 5 | 0.16 |
| 667 | 3D7R:B | hydrolase | 68 | 72 | RFNFR | 5 | 48.46 | EEEST | 8 | -1.38 |
| 668 | 3DAO:A | hydrolase | 216 | 220 | DNLND | 5 | 61.16 | CSGGG | 9 | -2.04 |
| 669 | 3DMO:D | hydrolase | 9 | 13 | AAKAA | 5 | 23.12 | HHHHH | 7 | 0.66 |
| 670 | 3EAR:B | hydrolase | 414 | 418 | LLALL | 5 | 31.32 | HHHHH | 6 | 3.40 |
| 671 | 3EEI:A | hydrolase | 83 | 87 | LGKGL | 5 | 59.40 | CSTTC | 4 | 0.58 |
| 672 | 3EMU:A | hydrolase | 406 | 410 | AIVIA | 5 | 1.00 | HHHHH | 5 | 3.36 |
| 673 | 3EN0:C | hydrolase | 247 | 251 | NLRLN | 5 | 77.06 | SCEEE | 5 | -0.78 |
| 674 | 3EN0:C | hydrolase | 38 | 42 | IIGII | 5 | 3.68 | EEEEE | 9 | 3.52 |
| 675 | 3EPW:A | hydrolase | 282 | 286 | DVVVD | 5 | 43.06 | EECCS | 7 | 1.12 |
| 676 | 3ERB:A | hydrolase | 144 | 148 | GFRFG | 5 | 77.62 | CCCCS | 7 | 0.06 |
| 677 | 3ERJ:B | hydrolase | 21 | 25 | AVQVA | 5 | 2.42 | HHHHH | 7 | 1.70 |
| 678 | 3F5O:H | hydrolase | 59 | 63 | LTATL | 5 | 0.74 | HHHHH | 10 | 1.60 |
| 679 | 3F9F:B | hydrolase | 120 | 124 | GSPSG | 5 | 29.46 | TEEEE | 6 | -0.80 |
| 680 | 3FAU:A | hydrolase | 74 | 78 | SFRFS | 5 | 84.72 | TCCEE | 6 | -0.10 |
| 681 | 3FCX:A | hydrolase | 217 | 221 | DILID | 5 | 12.96 | CEEEE | 10 | 1.16 |
| 682 | 3G91:A | hydrolase | 122 | 126 | LKYKL | 5 | 38.90 | HHHHH | 14 | -0.30 |
| 683 | 3G91:A | hydrolase | 253 | 257 | LEIEL | 5 | 30.86 | EEEEC | 9 | 1.02 |
| 684 | 3G9K:S | hydrolase | 388 | 392 | NQLQN | 5 | 71.20 | CCCCC | 9 | -2.04 |
| 685 | 3G9K:S | hydrolase | 488 | 492 | VKKKV | 5 | 89.40 | EEECC | 4 | -0.66 |
| 686 | 3GA3:A | hydrolase | 987 | 991 | FVVVF | 5 | 17.42 | EEEEE | 9 | 3.64 |
| 687 | 3GA7:A | hydrolase | 172 | 176 | LASAL | 5 | 2.08 | HHHHH | 7 | 2.08 |
| 688 | 3GFP:A | hydrolase | 338 | 343 | ATKKTA | 6 | 60.33 | SCHHHH | 7 | -0.93 |
| 689 | 3H09:B | hydrolase | 463 | 467 | EGKGE | 5 | 44.60 | CCBSB | 6 | -2.34 |
| 690 | 3H09:B | hydrolase | 663 | 667 | GEEEG | 5 | 43.94 | SCCTT | 6 | -2.26 |
| 691 | 3H09:B | hydrolase | 421 | 425 | TSDST | 5 | 62.36 | SSTTC | 6 | -1.30 |
| 692 | 3H09:B | hydrolase | 901 | 905 | SGNGS | 5 | 30.46 | EEEEE | 11 | -1.18 |
| 693 | 3H09:B | hydrolase | 111 | 115 | GNLNG | 5 | 12.56 | SSSCC | 6 | -0.80 |
| 694 | 3H09:B | hydrolase | 735 | 739 | EVVVE | 5 | 18.96 | CCCCT | 6 | 1.12 |
| 695 | 3H43:A | hydrolase | 105 | 109 | KVVVK | 5 | 38.58 | EEEEE | 8 | 0.96 |
| 696 | 3I10:A | hydrolase | 201 | 205 | KEAEK | 5 | 84.22 | TTHHH | 8 | -2.60 |
| 697 | 3I10:A | hydrolase | 112 | 116 | KLKLK | 5 | 67.00 | TSCBB | 8 | -0.82 |
| 698 | 3I26:B | hydrolase | 36 | 40 | DSRSD | 5 | 4.56 | SGGGC | 11 | -2.62 |
| 699 | 3I26:B | hydrolase | 61 | 65 | SSKSS | 5 | 34.26 | EECTT | 11 | -1.42 |
| 700 | 3I7M:A | hydrolase | 48 | 52 | PDQDP | 5 | 60.28 | SSSCC | 7 | -2.74 |
| 701 | 3I7M:A | hydrolase | 41 | 45 | VLALV | 5 | 6.44 | CCEEE | 7 | 3.56 |
| 702 | 3IAR:A | hydrolase | 31 | 35 | GRRRG | 5 | 94.18 | HHHHT | 9 | -2.86 |
| 703 | 3IAR:A | hydrolase | 55 | 59 | PLTLP | 5 | 54.18 | CCCHH | 9 | 0.74 |
| 704 | 3IAR:A | hydrolase | 14 | 18 | LHVHL | 5 | 4.10 | CCBBG | 9 | 1.08 |
| 705 | 3IAR:A | hydrolase | 343 | 347 | LLDLL | 5 | 29.26 | HHHHH | 8 | 2.34 |
| 706 | 3IB7:A | hydrolase | 138 | 142 | GHHHG | 5 | 55.54 | TCCSB | 7 | -2.08 |
| 707 | 3IOF:A | hydrolase | 223 | 231 | LKEKL | 5 | 58.56 | SCSSC | 9 | -0.74 |
| 708 | 3IOF:A | hydrolase | 168 | 172 | PLVLP | 5 | 45.30 | CCCCC | 4 | 1.72 |
| 709 | 3IVE:A | hydrolase | 67 | 71 | AKNKA | 5 | 85.34 | HHCSS | 6 | -1.54 |
| 710 | 3JQL:A | hydrolase | 53 | 57 | NEAEN | 5 | 84.38 | HHHTT | 9 | -2.44 |
| 711 | 3JQL:A | hydrolase | 26 | 30 | GCYCG | 5 | 4.60 | TTTBS | 9 | 0.58 |
| 712 | 3JU4:A | hydrolase | 561 | 565 | NSPSN | 5 | 72.76 | TCTTC | 3 | -2.04 |
| 713 | 3JU4:A | hydrolase | 701 | 705 | GVGVG | 5 | 0.18 | CSEEE | 3 | 1.44 |
| 714 | 3K1W:B | hydrolase | 207 | 211 | GVSVG | 5 | 14.84 | EEEET | 9 | 1.36 |
| 715 | 3KZX:A | hydrolase | 164 | 168 | DSISD | 5 | 46.34 | SSHHH | 8 | -0.82 |
| 716 | 3LGI:C | hydrolase | 166 | 170 | QTITQ | 5 | 11.96 | SEEEE | 24 | -0.78 |
| 717 | 3LHI:A | hydrolase | 19 | 23 | DAVAD | 5 | 41.40 | HHHHH | 5 | 0.16 |
| 718 | 3LHI:A | hydrolase | 10 | 14 | AAEAA | 5 | 54.50 | HHHHH | 5 | 0.74 |
| 719 | 3LQB:A | hydrolase | 180 | 184 | GQRQG | 5 | 68.24 | CCCSS | 5 | -2.46 |
| 720 | 3LQB:A | hydrolase | 38 | 42 | IANAI | 5 | 33.46 | HHHHH | 9 | 1.82 |
| 721 | 3LQW:A | hydrolase | 75 | 79 | VGAGV | 5 | 50.40 | ECCCE | 6 | 1.88 |
| 722 | 3LUM:D | hydrolase | 87 | 91 | IQSQI | 5 | 18.68 | HHHHH | 9 | 0.24 |
| 723 | 3LUM:D | hydrolase | 70 | 74 | VVHVV | 5 | 0.40 | EEEEE | 9 | 2.72 |
| 724 | 3LYE:A | hydrolase | 113 | 117 | TGAGT | 5 | 6.82 | CHHHH | 7 | -0.08 |
| 725 | 3LYE:A | hydrolase | 269 | 273 | AAVAA | 5 | 33.16 | HHHHH | 8 | 2.28 |
| 726 | 3MK1:A | hydrolase | 94 | 98 | ATATA | 5 | 0.66 | HHHHH | 9 | 0.80 |
| 727 | 3MXO:A | hydrolase | 214 | 218 | RADAR | 5 | 110.14 | CCCTT | 5 | -1.78 |
| 728 | 3NE8:A | hydrolase | 372 | 376 | KEDEK | 5 | 70.06 | HHHHH | 9 | -3.66 |
| 729 | 3NO6:A | hydrolase | 101 | 105 | VKEKV | 5 | 101.48 | CCSCC | 3 | -0.58 |
| 730 | 3NUQ:A | hydrolase | 22 | 26 | AKNKA | 5 | 74.56 | HHHHH | 11 | -1.54 |
| 731 | 3NUQ:A | hydrolase | 248 | 252 | VENEV | 5 | 109.78 | CSCCC | 4 | -0.42 |
| 732 | 3O1C:A | hydrolase | 67 | 71 | EDADE | 5 | 102.98 | CGGGH | 6 | -2.44 |
| 733 | 3O1C:A | hydrolase | 111 | 115 | VHLHV | 5 | 10.10 | CCEEE | 6 | 1.16 |
| 734 | 3O2R:B | hydrolase | 46 | 50 | VLDLV | 5 | 7.94 | HHHHH | 9 | 2.50 |
| 735 | 3OYV:A | hydrolase | 238 | 242 | NNSNN | 5 | 85.38 | CGGGS | 5 | -2.96 |
| 736 | 3P1V:B | hydrolase | 66 | 70 | ELPLE | 5 | 58.30 | CCSSC | 6 | -0.20 |
| 737 | 3P1V:B | hydrolase | 366 | 370 | IPTPI | 5 | 61.98 | SSCCG | 6 | 1.02 |
| 738 | 3P1V:B | hydrolase | 124 | 128 | VEVEV | 5 | 12.30 | EEEEE | 8 | 1.12 |
| 739 | 3P2U:B | hydrolase | 3 | 7 | LTLTL | 5 | 18.68 | EEEEE | 8 | 2.00 |
| 740 | 3PCT:C | hydrolase | 53 | 57 | KKGKK | 5 | 85.30 | CTTCE | 5 | -3.20 |
| 741 | 3PCT:C | hydrolase | 180 | 184 | DNLND | 5 | 20.34 | SSGGG | 5 | -2.04 |
| 742 | 3PCT:C | hydrolase | 88 | 92 | KTWTK | 5 | 49.26 | HHHHH | 8 | -2.02 |
| 743 | 3PI6:A | hydrolase | 267 | 271 | GGHGG | 5 | 27.04 | CSTTC | 8 | -0.96 |
| 744 | 3PT5:A | hydrolase | 271 | 275 | PDEDP | 5 | 62.24 | GGGSC | 8 | -2.74 |
| 745 | 3PW3:F | hydrolase | 65 | 69 | LESEL | 5 | 6.76 | HHHHH | 12 | -0.04 |
| 746 | 3Q46:A | hydrolase | 8 | 12 | EPGPE | 5 | 60.90 | CSCSB | 5 | -2.12 |
| 747 | 3Q7H:N | hydrolase | 102 | 106 | ALLLA | 5 | 3.32 | HHHHH | 6 | 3.00 |
| 748 | 3QC2:B | hydrolase | 188 | 192 | FAKAF | 5 | 55.22 | TSSHH | 4 | 1.06 |
| 749 | 3QFH:B | hydrolase | 74 | 78 | NALAN | 5 | 44.48 | HHHTT | 5 | 0.08 |
| 750 | 3QIT:A | hydrolase | 43 | 47 | ALPLA | 5 | 23.78 | HHHHH | 6 | 1.92 |
| 751 | 3QPA:A | hydrolase | 123 | 127 | AALAA | 5 | 1.10 | HHHHH | 24 | 2.20 |
| 752 | 3QT9:A | hydrolase | 279 | 283 | EGFGE | 5 | 75.60 | TTTEE | 4 | -1.00 |
| 753 | 3QT9:A | hydrolase | 340 | 345 | GKAAKG | 6 | 58.85 | CSSCEE | 4 | -0.83 |
| 754 | 3QU5:B | hydrolase | 202 | 206 | DAGAD | 5 | 47.86 | HTTCS | 5 | -0.76 |
| 755 | 3QU5:B | hydrolase | 201 | 207 | LDAGADL | 7 | 51.51 | HHTTCSE | 5 | 0.54 |
| 756 | 3QU5:B | hydrolase | 200 | 208 | LLDAGADLL | 9 | 47.60 | HHHTTCSEE | 5 | 1.27 |
| 757 | 3QYJ:B | hydrolase | 22 | 26 | AGHGA | 5 | 57.88 | ECSSE | 4 | -0.08 |
| 758 | 3R0V:A | hydrolase | 125 | 129 | PPVPP | 5 | 81.00 | CCCCT | 3 | -0.44 |
| 759 | 3RL5:A | hydrolase | 66 | 70 | TRSRT | 5 | 34.06 | CTTCC | 11 | -2.24 |
| 760 | 3RLG:A | hydrolase | 119 | 123 | NNGNN | 5 | 72.20 | GGGSS | 6 | -2.88 |
| 761 | 3RQZ:C | hydrolase | 48 | 52 | LVRVL | 5 | 9.32 | HHHHH | 6 | 2.30 |
| 762 | 3RYD:A | hydrolase | 52 | 56 | QQFQQ | 5 | 63.48 | HHHHH | 10 | -2.24 |
| 763 | 3S5B:A | hydrolase | 207 | 211 | PLYLP | 5 | 18.92 | EECCE | 6 | 0.62 |
| 764 | 3SGG:A | hydrolase | 256 | 260 | KDLDK | 5 | 88.14 | HTSCT | 8 | -2.20 |
| 765 | 3SZY:A | hydrolase | 137 | 141 | LGKGL | 5 | 26.90 | HTTTC | 6 | 0.58 |
| 766 | 3TK9:A | hydrolase | 121 | 125 | PLRLP | 5 | 61.74 | CCBCC | 5 | -0.02 |
| 767 | 3TX2:A | hydrolase | 10 | 14 | ADTDA | 5 | 74.38 | SSHHH | 6 | -0.82 |
| 768 | 3TX2:A | hydrolase | 42 | 46 | TGGGT | 5 | 31.34 | CCSHH | 6 | -0.52 |
| 769 | 3TX2:A | hydrolase | 220 | 224 | PVDVP | 5 | 32.58 | TTTSG | 6 | 0.34 |
| 770 | 3TX2:A | hydrolase | 211 | 215 | VAAAV | 5 | 27.70 | HHHHH | 6 | 2.76 |
| 771 | 3U3G:A | hydrolase | 48 | 52 | AEYEA | 5 | 12.30 | HHHHH | 11 | -0.94 |
| 772 | 3V7P:A | hydrolase | 244 | 248 | FLELF | 5 | 25.90 | HHHTT | 8 | 1.94 |
| 773 | 3VNY:A | hydrolase | 138 | 142 | GLNLG | 5 | 5.64 | EECTT | 9 | 0.66 |
| 774 | 3VNY:A | hydrolase | 477 | 481 | LAAAL | 5 | 103.44 | TTCTT | 9 | 2.60 |
| 775 | 3ZWF:A | hydrolase | 28 | 32 | CEGEC | 5 | 51.50 | ETTEE | 3 | -0.48 |
| 776 | 3ZWF:A | hydrolase | 186 | 190 | SFGFS | 5 | 0.60 | EEEEE | 11 | 0.72 |
| 777 | 3ZX3:A | hydrolase | 129 | 133 | GATAG | 5 | 4.52 | EECHH | 9 | 0.42 |
| 778 | 4A3P:A | hydrolase | 161 | 165 | IEKEI | 5 | 42.60 | HHHHH | 10 | -0.38 |
| 779 | 4DFA:A | hydrolase | 90 | 94 | AYAYA | 5 | 8.54 | EEEEE | 10 | 0.56 |
| 780 | 4DZI:C | hydrolase | 393 | 397 | GVQVG | 5 | 99.32 | CCCTT | 9 | 0.82 |
| 781 | 4DZI:C | hydrolase | 223 | 227 | GVPVG | 5 | 2.76 | TCCEE | 9 | 1.20 |
| 782 | 8A3H:A | hydrolase | 78 | 82 | VKEKV | 5 | 43.48 | GHHHH | 8 | -0.58 |
| 783 | 8A3H:A | hydrolase | 90 | 94 | IDLDI | 5 | 36.70 | HHHTC | 8 | 1.16 |
| 784 | 1KWF:A | hydrolase | 378 | 382 | LLTLL | 5 | 1.00 | HHHHH | 8 | 2.90 |
| 785 | 1ME4:A | hydrolase | 160 | 165 | GVLLVG | 6 | 2.17 | EEEEEE | 7 | 2.53 |
| 786 | 1MJ5:A | hydrolase | 284 | 288 | AAIAA | 5 | 19.06 | HHHHH | 29 | 2.34 |
| 787 | 1O7J:C | hydrolase | 138 | 142 | AVRVA | 5 | 14.14 | HHHHH | 23 | 1.50 |
| 788 | 1UOC:A | hydrolase | 251 | 257 | SLLMLLS | 7 | 3.53 | HHHHHHH | 7 | 2.21 |
| 789 | 2I74:A | hydrolase | 611 | 615 | LEAEL | 5 | 17.08 | EEEEE | 10 | 0.48 |
| 790 | 2OSX:A | hydrolase | 482 | 486 | VTVTV | 5 | 20.10 | EEEEE | 8 | 2.24 |
| 791 | 2UWA:A | hydrolase | 94 | 98 | EIDIE | 5 | 11.82 | EEEEE | 9 | -0.30 |
| 792 | 2V3I:A | hydrolase | 360 | 364 | MVLVM | 5 | 0.06 | EEEEE | 9 | 3.20 |
| 793 | 2VU9:A | hydrolase | 941 | 945 | FSTSF | 5 | 2.20 | EEEEE | 9 | 0.66 |
| 794 | 2ZKM:X | hydrolase | 775 | 779 | HLCLH | 5 | 5.94 | EEEEE | 10 | 0.74 |
| 795 | 2ZKM:X | hydrolase | 791 | 795 | LFIFL | 5 | 7.30 | EEEEE | 10 | 3.54 |
| 796 | 3AMN:B | hydrolase | 95 | 101 | FSVEVSF | 7 | 10.94 | EEEEEEE | 10 | 1.27 |
| 797 | 3CTZ:A | hydrolase | 539 | 543 | VVLVV | 5 | 7.94 | EEEEE | 10 | 4.12 |
| 798 | 3I26:B | hydrolase | 226 | 230 | YYGYY | 5 | 30.54 | EEEEE | 7 | -1.12 |
| 799 | 3KUV:B | hydrolase | 85 | 89 | TVTVT | 5 | 23.42 | EEEEE | 8 | 1.26 |
| 800 | 3LHI:A | hydrolase | 17 | 25 | LADAVADAL | 9 | 25.04 | HHHHHHHHH | 5 | 1.33 |
| 801 | 3RL5:A | hydrolase | 11 | 15 | VTITV | 5 | 81.02 | CCCCC | 1 | 2.30 |
| 802 | 3ZX3:A | hydrolase | 134 | 139 | MRLLRM | 6 | 20.68 | HHHHHH | 7 | 0.40 |
| 803 | 1NWW:A | hydrolase | 15 | 21 | SAAGAAS | 7 | 51.93 | TTTTCCC | 3 | 0.74 |
| 804 | 3RL5:A | hydrolase | 65 | 71 | DTRSRTD | 7 | 41.00 | CCTTCCT | 14 | -2.60 |
| 805 | 3B7E:A | hydrolase | 324 | 330 | DNPRPND | 7 | 35.89 | SSSCCSS | 11 | -3.10 |
| 806 | 1EB6:A | hydrolase | 69 | 74 | TSGGST | 6 | 52.75 | SBCSSC | 7 | -0.63 |
| 807 | 2ADV:B | hydrolase | 12 | 16 | ANGNA | 5 | 46.24 | SSSCC | 6 | -0.76 |
| 808 | 3SZY:A | hydrolase | 366 | 371 | DLAALD | 6 | 86.43 | CBCCCS | 5 | 0.70 |
| 809 | 1ZPS:B | hydrolase | 64 | 70 | GKLWLKG | 7 | 46.21 | TEEEETT | 7 | -0.27 |
| 810 | 3D59:A | hydrolase | 394 | 400 | KHLGLHK | 7 | 71.89 | HHHTCCS | 5 | -1.00 |
| 811 | 3LGI:C | hydrolase | 165 | 171 | GQTITQG | 7 | 11.03 | SSEEEEE | 26 | -0.67 |
| 812 | 8A3H:A | hydrolase | 80 | 84 | EKVKE | 5 | 50.06 | HHHHH | 10 | -2.12 |
| 813 | 1BXO:A | hydrolase | 91 | 96 | TVGGVT | 6 | 27.95 | EETTEE | 18 | 1.03 |
| 814 | 3P2U:B | hydrolase | 4 | 8 | TLTLT | 5 | 16.48 | EEEEE | 9 | 1.10 |
| 815 | 1VJN:A | hydrolase | 176 | 182 | DFLKLFD | 7 | 67.86 | HHHTTSS | 5 | 0.33 |
| 816 | 1LYV:A | hydrolase | 200 | 205 | RLTTLR | 6 | 59.88 | HHHHHH | 14 | -0.47 |
| 817 | 3LHI:A | hydrolase | 18 | 24 | ADAVADA | 7 | 31.53 | HHHHHHH | 9 | 0.63 |
| 818 | 3AKH:A | hydrolase | 227 | 231 | ASASA | 5 | 40.52 | EETTS | 5 | 0.76 |
| 819 | 3CZ8:A | hydrolase | 228 | 234 | QLRDRLQ | 7 | 56.87 | HHHHHHH | 10 | -1.70 |
| 820 | 2D0B:A | hydrolase | 57 | 63 | AAEQEAA | 7 | 47.00 | THHHHHH | 8 | -0.47 |
| 821 | 1NZ0:A | hydrolase | 107 | 113 | KLLNLLK | 7 | 33.97 | HHHHHHT | 7 | 0.56 |
| 822 | 2XF3:A | hydrolase | 220 | 224 | RVTVR | 5 | 57.00 | EEEHH | 8 | -0.26 |
| 823 | 2XF3:A | hydrolase | 113 | 117 | LIRIL | 5 | 34.88 | CEEEE | 8 | 2.42 |
| 824 | 3LUM:D | hydrolase | 76 | 81 | NEEEEN | 6 | 72.15 | SSGGGS | 4 | -3.50 |
| 825 | 3I7M:A | hydrolase | 47 | 53 | FPDQDPF | 7 | 43.74 | CSSSCCE | 5 | -1.16 |
| 826 | 2AS9:A | hydrolase | 32 | 39 | KNTIITNK | 8 | 11.86 | TTEEEECH | 23 | -0.90 |
| 827 | 1ESC:A | hydrolase (serine esterase) | 247 | 251 | AGTGA | 5 | 47.62 | GGCTT | 5 | 0.42 |
| 828 | 3PP2:A | hydrolase activator | 513 | 517 | KRLRK | 5 | 125.62 | EECSS | 6 | -2.60 |
| 829 | 3PP2:A | hydrolase activator | 520 | 524 | WSASW | 5 | 37.90 | CEEEE | 6 | -0.32 |
| 830 | 1R8N:A | hydrolase inhibitor | 37 | 41 | GRTRG | 5 | 83.30 | ECCTT | 5 | -2.10 |
| 831 | 1R8N:A | hydrolase inhibitor | 181 | 185 | SETES | 5 | 116.40 | SCCCC | 5 | -1.86 |
| 832 | 1UGI:B | hydrolase inhibitor | 27 | 31 | EEVEE | 5 | 81.12 | HHHHH | 5 | -1.96 |
| 833 | 2IQY:A | hydrolase inhibitor | 65 | 69 | TLVLT | 5 | 0.04 | EEEEE | 11 | 2.08 |
| 834 | 3F02:A | hydrolase inhibitor | 157 | 161 | NNTNN | 5 | 64.64 | HTTTT | 7 | -2.94 |
| 835 | 3MWZ:A | hydrolase inhibitor | 84 | 88 | ANANA | 5 | 76.26 | CCTTS | 2 | -0.32 |
| 836 | 3S8K:A | hydrolase inhibitor | 84 | 88 | NITIN | 5 | 86.36 | SSCCS | 4 | 0.26 |
| 837 | 3F02:A | hydrolase inhibitor | 201 | 205 | TFSFT | 5 | 50.24 | EEEEE | 7 | 0.68 |
| 838 | 1B1U:A | hydrolase inhibitor | 87 | 91 | RQVQR | 5 | 55.06 | HHHHH | 12 | -2.36 |
| 839 | 3ED1:C | hydrolase receptor | 127 | 131 | SSASS | 5 | 7.22 | CCTTB | 9 | -0.28 |
| 840 | 3Q7Z:A | hydrolase regulator/antibiotic | 357 | 361 | GSNSG | 5 | 47.24 | TTCEE | 8 | -1.18 |
| 841 | 3Q7Z:A | hydrolase regulator/antibiotic | 509 | 513 | LSSSL | 5 | 19.18 | HHHHH | 8 | 1.04 |
| 842 | 1QLW:B | hydrolase(carboxylic esterase) | 16 | 20 | SGQGS | 5 | 27.22 | EEEEE | 9 | -1.18 |
| 843 | 1QLW:B | hydrolase(carboxylic esterase) | 182 | 186 | TPNPT | 5 | 43.52 | SSCHH | 5 | -1.62 |
| 844 | 1QLW:B | hydrolase(carboxylic esterase) | 311 | 315 | DLILD | 5 | 41.84 | HHHHH | 9 | 1.02 |
| 845 | 1TCA:A | hydrolase(carboxylic esterase) | 190 | 194 | VQPQV | 5 | 39.24 | SCCCC | 9 | -0.04 |
| 846 | 1ARB:A | hydrolase(serine protease) | 97 | 101 | SQTQS | 5 | 55.78 | CCEEE | 6 | -1.86 |
| 847 | 3CG7:A | hydrolase, apoptosis | 239 | 243 | VLPLV | 5 | 5.62 | HSSCE | 8 | 2.88 |
| 848 | 1QZQ:B | hydrolase, dna binding protein | 236 | 240 | AHLHA | 5 | 63.98 | HHHHH | 7 | 0.20 |
| 849 | 3N0U:A | hydrolase, lyase | 9 | 13 | ERIRE | 5 | 78.42 | HHHHH | 15 | -2.30 |
| 850 | 3N0U:A | hydrolase, lyase | 201 | 205 | VKGKV | 5 | 59.54 | HHSCC | 11 | 0.04 |
| 851 | 2RG9:B | hydrolase, toxin | 67 | 71 | TYGYT | 5 | 67.56 | ESCSS | 5 | -0.88 |
| 852 | 2RG9:B | hydrolase, toxin | 202 | 206 | VINIV | 5 | 36.32 | BCEEE | 5 | 2.78 |
| 853 | 3BGY:A | hydrolase, viral protein | 210 | 214 | ELELE | 5 | 18.64 | EEEEE | 9 | -0.58 |
| 854 | 3BGY:A | hydrolase, viral protein | 224 | 228 | LESEL | 5 | 21.24 | HHHHH | 9 | -0.04 |
| 855 | 3BGY:A | hydrolase, viral protein | 211 | 215 | LELEL | 5 | 8.34 | EEEEE | 8 | 0.88 |
| 856 | 3PBT:A | hydrolase/antibiotic | 243 | 247 | GAKAG | 5 | 32.80 | TCSEE | 5 | -0.22 |
| 857 | 3PBT:A | hydrolase/antibiotic | 161 | 165 | VAHAV | 5 | 3.30 | GHHHH | 5 | 1.76 |
| 858 | 3PBT:A | hydrolase/antibiotic | 99 | 103 | LAAAL | 5 | 30.78 | HHHHT | 5 | 2.60 |
| 859 | 3Q6X:B | hydrolase/antibiotic | 213 | 217 | SKAKS | 5 | 107.24 | TTCSC | 3 | -1.52 |
| 860 | 3Q6X:B | hydrolase/antibiotic | 171 | 175 | PATAP | 5 | 61.64 | GGGST | 3 | -0.06 |
| 861 | 3Q6X:B | hydrolase/antibiotic | 113 | 117 | VALAV | 5 | 14.84 | EEEEE | 7 | 3.16 |
| 862 | 3DDC:B | hydrolase/apoptosis | 229 | 233 | GTYTG | 5 | 23.88 | GCEEE | 8 | -0.70 |
| 863 | 3DDC:B | hydrolase/apoptosis | 206 | 210 | IKQKI | 5 | 56.20 | HHHHH | 9 | -0.46 |
| 864 | 3DDC:B | hydrolase/apoptosis | 244 | 248 | PVTVP | 5 | 110.22 | EECCC | 8 | 0.90 |
| 865 | 3LL8:A | hydrolase/calcium binding protein | 257 | 261 | SYFYS | 5 | 11.72 | SEEEC | 11 | -0.28 |
| 866 | 3LL8:A | hydrolase/calcium binding protein | 365 | 369 | LVNVL | 5 | 23.40 | HHHHH | 6 | 2.50 |
| 867 | 3A2O:B | hydrolase/hydrolase inhibitor | 148 | 152 | GGIGG | 5 | 28.74 | EETTE | 13 | 0.58 |
| 868 | 1GA6:A | hydrolase/hydrolase inhibitor | 140 | 144 | DANAD | 5 | 61.44 | HHHHT | 7 | -1.38 |
| 869 | 1GA6:A | hydrolase/hydrolase inhibitor | 335 | 340 | GYGGYG | 6 | 61.47 | SSTTCS | 7 | -0.70 |
| 870 | 2AEB:B | hydrolase/hydrolase inhibitor | 38 | 42 | EKLKE | 5 | 83.86 | HHHHH | 15 | -2.20 |
| 871 | 2AEB:B | hydrolase/hydrolase inhibitor | 106 | 110 | GSISG | 5 | 2.22 | HHHHH | 15 | 0.42 |
| 872 | 2AEB:B | hydrolase/hydrolase inhibitor | 289 | 293 | VTRTV | 5 | 35.56 | HHHHH | 15 | 0.50 |
| 873 | 2FP7:A | hydrolase/hydrolase inhibitor | 74 | 78 | RVDVR | 5 | 69.36 | EEEEE | 6 | -0.82 |
| 874 | 2Z7F:I | hydrolase/hydrolase inhibitor | 93 | 97 | CMGMC | 5 | 32.66 | EEETT | 5 | 1.68 |
| 875 | 3AMR:A | hydrolase/hydrolase inhibitor | 291 | 295 | LGFGL | 5 | 18.70 | ECSCC | 6 | 1.92 |
| 876 | 3AMR:A | hydrolase/hydrolase inhibitor | 123 | 127 | IATAI | 5 | 36.30 | EECCC | 6 | 2.38 |
| 877 | 3D6M:A | hydrolase/hydrolase inhibitor | 226 | 230 | TSDST | 5 | 15.24 | GCSCE | 12 | -1.30 |
| 878 | 3QSD:A | hydrolase/hydrolase inhibitor | 137 | 141 | CGLGC | 5 | 32.58 | SBCTT | 10 | 1.60 |
| 879 | 3QZR:A | hydrolase/hydrolase inhibitor | 159 | 163 | GIHIG | 5 | 3.68 | EEEEE | 15 | 1.00 |
| 880 | 3QZR:A | hydrolase/hydrolase inhibitor | 53 | 57 | LVNVL | 5 | 47.44 | EEEEE | 15 | 2.50 |
| 881 | 3SLZ:B | hydrolase/hydrolase inhibitor | 11 | 15 | EPPPE | 5 | 85.56 | CCCCS | 4 | -2.36 |
| 882 | 3HNA:B | hydrolase/hydrolase regulator | 1093 | 1097 | LRARL | 5 | 50.84 | CCSCE | 5 | 0.08 |
| 883 | 1V5I:B | hydrolase/protein binding | 5 | 9 | FIVIF | 5 | 4.78 | EEEEE | 10 | 3.76 |
| 884 | 3TMP:E | hydrolase/protein binding | 302 | 306 | STGTS | 5 | 74.84 | CTTCS | 2 | -0.68 |
| 885 | 1V5I:B | hydrolase/protein binding | 4 | 10 | KFIVIFK | 7 | 26.34 | EEEEEEC | 11 | 1.57 |
| 886 | 2XFG:A | hydrolase/sugar binding protein | 320 | 324 | ATTTA | 5 | 0.52 | HHHHH | 25 | 0.30 |
| 887 | 3ZVL:A | hydrolase/transferase | 350 | 354 | PLYLP | 5 | 51.70 | CSSBS | 6 | 0.62 |
| 888 | 2ZXE:B | hydrolase/transport protein | 244 | 248 | YYPYY | 5 | 8.28 | GCSBC | 8 | -1.36 |
| 889 | 2ZXE:B | hydrolase/transport protein | 293 | 297 | FRGRF | 5 | 24.30 | TSSBC | 8 | -0.76 |
| 890 | 2ZXE:B | hydrolase/transport protein | 89 | 93 | ISFSI | 5 | 23.66 | EEECT | 8 | 2.04 |
| 891 | 2ZXE:B | hydrolase/transport protein | 49 | 53 | GIFIG | 5 | 55.04 | HHHHH | 8 | 2.20 |
| 892 | 1D9C:A | immune system | 45 | 49 | IQSQI | 5 | 4.54 | HHHHH | 12 | 0.24 |
| 893 | 1DQT:A | immune system | 98 | 102 | YPPPY | 5 | 88.46 | ESSSC | 5 | -1.48 |
| 894 | 1FO0:B | immune system | 39 | 43 | LQKQL | 5 | 76.16 | TTCCE | 5 | -0.66 |
| 895 | 1FYH:E | immune system | 212 | 216 | EKSKE | 5 | 72.88 | CCCCC | 7 | -3.12 |
| 896 | 1IFR:A | immune system | 500 | 504 | AGAGA | 5 | 44.36 | TTSSC | 5 | 0.92 |
| 897 | 1K5N:A | immune system | 223 | 227 | DQTQD | 5 | 104.96 | ECGGG | 3 | -2.94 |
| 898 | 1K5N:A | immune system | 266 | 270 | LPKPL | 5 | 101.78 | CSSCE | 3 | 0.10 |
| 899 | 1KZQ:A | immune system | 124 | 128 | VTVTV | 5 | 16.38 | EEEEE | 8 | 2.24 |
| 900 | 1LY2:A | immune system | 57 | 61 | KPAPK | 5 | 60.02 | SCCCE | 4 | -1.84 |
| 901 | 1MJU:H | immune system | 73 | 77 | TSSST | 5 | 57.06 | TTTTE | 3 | -0.76 |
| 902 | 1MJU:L | immune system | 103 | 107 | KLELK | 5 | 52.20 | EEEEC | 5 | -0.74 |
| 903 | 1MJU:L | immune system | 63 | 67 | SGSGS | 5 | 39.92 | EEEEC | 5 | -0.64 |
| 904 | 1MJU:L | immune system | 178 | 182 | TLTLT | 5 | 31.94 | EEEEE | 8 | 1.10 |
| 905 | 1QFO:C | immune system | 113 | 117 | TVTVT | 5 | 14.14 | EEEEE | 11 | 1.26 |
| 906 | 1QQF:A | immune system | 1262 | 1266 | YGGGY | 5 | 89.90 | CTTST | 3 | -0.76 |
| 907 | 1QQF:A | immune system | 1277 | 1281 | QALAQ | 5 | 22.24 | HHHHH | 8 | 0.08 |
| 908 | 1QQF:A | immune system | 1237 | 1241 | LLALL | 5 | 2.68 | HHHHH | 8 | 3.40 |
| 909 | 1TY0:B | immune system | 54 | 58 | SEVES | 5 | 40.66 | EECCT | 7 | -0.88 |
| 910 | 1TY0:B | immune system | 178 | 182 | TSSST | 5 | 62.52 | CTTCC | 7 | -0.76 |
| 911 | 1U58:A | immune system | 212 | 216 | DVYVD | 5 | 52.14 | EEEEC | 6 | 0.02 |
| 912 | 1XT5:A | immune system | 93 | 97 | TLRLT | 5 | 31.86 | CEEEC | 8 | 0.34 |
| 913 | 2BOU:A | immune system | 87 | 91 | NESEN | 5 | 75.26 | SGGGC | 6 | -2.96 |
| 914 | 2J4W:D | immune system | 448 | 452 | VCNCV | 5 | 38.40 | ECCSB | 9 | 1.98 |
| 915 | 2JJU:A | immune system | 41 | 45 | GAGAG | 5 | 50.64 | CSSTT | 4 | 0.48 |
| 916 | 2JJU:A | immune system | 75 | 79 | SISIS | 5 | 18.38 | CEEEC | 4 | 1.32 |
| 917 | 2JKS:A | immune system | 32 | 36 | SLTLS | 5 | 29.14 | EEEEB | 8 | 1.06 |
| 918 | 2JKS:A | immune system | 302 | 306 | TVLVT | 5 | 9.70 | EEEEE | 9 | 2.16 |
| 919 | 2PTV:A | immune system | 54 | 58 | FESEF | 5 | 62.60 | CSSTT | 5 | -0.44 |
| 920 | 2Q87:A | immune system | 101 | 105 | VEVEV | 5 | 31.54 | EEEEE | 10 | 1.12 |
| 921 | 2QHL:A | immune system | 27 | 31 | KDKDK | 5 | 83.92 | SCGGG | 6 | -3.74 |
| 922 | 2QHL:A | immune system | 94 | 98 | GEVEG | 5 | 34.56 | EEEET | 6 | -0.72 |
| 923 | 2WCR:A | immune system | 101 | 105 | IKQKI | 5 | 72.58 | CCSCC | 7 | -0.46 |
| 924 | 2WQR:A | immune system | 263 | 267 | TIQIT | 5 | 59.66 | CEEEE | 5 | 0.82 |
| 925 | 2WY8:Q | immune system | 62 | 66 | LQKQL | 5 | 50.70 | HHHHH | 9 | -0.66 |
| 926 | 2XTM:B | immune system | 108 | 112 | VLLLV | 5 | 2.00 | EEEEE | 8 | 3.96 |
| 927 | 2XZ4:A | immune system | 151 | 155 | ISVSI | 5 | 20.70 | SCEEE | 8 | 2.32 |
| 928 | 2Z80:A | immune system | 232 | 236 | TDLDT | 5 | 39.72 | CBCTT | 11 | -0.92 |
| 929 | 2Z80:A | immune system | 56 | 60 | SLDLS | 5 | 13.48 | EEECT | 11 | 0.50 |
| 930 | 3BJ9:1 | immune system | 20 | 24 | LTCTL | 5 | 10.30 | EEEEE | 1 | 1.74 |
| 931 | 3BT2:U | immune system | 33 | 37 | EEGEE | 5 | 89.98 | ETTEE | 2 | -2.88 |
| 932 | 3GMO:A | immune system | 21 | 25 | RSWSR | 5 | 97.70 | TTEEE | 6 | -2.30 |
| 933 | 3GOD:D | immune system | 96 | 114 | EVDVE | 5 | 66.60 | CCCCC | 6 | -0.42 |
| 934 | 3GOD:D | immune system | 78 | 82 | GVLVG | 5 | 16.28 | TCEEE | 7 | 2.28 |
| 935 | 3NKE:B | immune system | 107 | 111 | LDEDL | 5 | 45.46 | HSHHH | 7 | -0.58 |
| 936 | 3OQ2:A | immune system | 66 | 70 | DKEKD | 5 | 84.58 | CTTTC | 10 | -3.66 |
| 937 | 3OQ2:A | immune system | 24 | 28 | RRLRR | 5 | 63.82 | HHHHH | 20 | -2.84 |
| 938 | 3Q0H:A | immune system | 116 | 120 | GTYTG | 5 | 43.96 | CCEEE | 7 | -0.70 |
| 939 | 3QXV:E | immune system | 19 | 23 | SLRLS | 5 | 58.10 | CEEEE | 7 | 0.30 |
| 940 | 3QXV:E | immune system | 34 | 38 | WAMAW | 5 | 16.68 | CEEEE | 7 | 0.74 |
| 941 | 3RY4:A | immune system | 168 | 172 | VTITV | 5 | 33.20 | EEEEE | 9 | 2.30 |
| 942 | 4ACJ:A | immune system | 764 | 768 | LDGDL | 5 | 26.74 | EETTS | 13 | 0.04 |
| 943 | 4ACJ:A | immune system | 722 | 726 | TFLFT | 5 | 1.06 | CEEEE | 13 | 1.60 |
| 944 | 1MKF:B | immune system | 104 | 108 | LVRVL | 5 | 8.10 | EEEEE | 10 | 2.30 |
| 945 | 2Z80:A | immune system | 202 | 206 | HLILH | 5 | 15.46 | EEEEE | 11 | 1.14 |
| 946 | 1MJU:L | immune system | 64 | 68 | GSGSG | 5 | 35.18 | EEECS | 5 | -0.56 |
| 947 | 1K5N:A | immune system | 107 | 112 | GRLLRG | 6 | 70.63 | CCEEEE | 7 | -0.37 |
| 948 | 2XZ4:A | immune system | 150 | 156 | AISVSIA | 7 | 21.67 | CSCEEEE | 10 | 2.17 |
| 949 | 1QFO:C | immune system | 112 | 118 | TTVTVTT | 7 | 23.64 | EEEEEEC | 14 | 0.70 |
| 950 | 2JJU:A | immune system | 40 | 46 | RGAGAGR | 7 | 59.40 | ECSSTTC | 7 | -0.94 |
| 951 | 1G1S:A | immune system, membrane protein | 71 | 75 | NEAEN | 5 | 83.72 | TTTCC | 5 | -2.44 |
| 952 | 1G1S:A | immune system, membrane protein | 44 | 49 | YYSSYY | 6 | 53.18 | CCTTCE | 6 | -1.13 |
| 953 | 2ZX2:B | immune system, sugar binding protein | 19 | 23 | KIHIK | 5 | 65.72 | EEEEE | 7 | -0.40 |
| 954 | 2ZX2:B | immune system, sugar binding protein | 172 | 176 | VSNSV | 5 | 39.60 | CSHHH | 6 | 0.66 |
| 955 | 2WY3:D | immune system/viral protein | 118 | 122 | TVPVT | 5 | 38.18 | SSSEE | 8 | 1.08 |
| 956 | 2WY3:D | immune system/viral protein | 60 | 64 | CISIC | 5 | 6.88 | CEEEE | 8 | 2.64 |
| 957 | 2W7Z:A | inhibitor | 202 | 206 | LASAL | 5 | 29.56 | HHHHT | 6 | 2.08 |
| 958 | 1M0K:A | ion transport | 62 | 66 | LGYGL | 5 | 37.64 | TTTTC | 7 | 1.10 |
| 959 | 1M0K:A | ion transport | 116 | 120 | GIMIG | 5 | 19.30 | HHHHH | 9 | 2.02 |
| 960 | 1EYQ:B | isomerase | 134 | 140 | KFAEAFK | 7 | 46.74 | HHHHHHT | 9 | -0.30 |
| 961 | 3A9S:C | isomerase | 72 | 78 | EAAEAAE | 7 | 54.90 | HHHHHHH | 9 | -0.47 |
| 962 | 1EK6:A | isomerase | 270 | 274 | GTGTG | 5 | 31.22 | CCSCC | 5 | -0.52 |
| 963 | 1EYQ:B | isomerase | 127 | 131 | AEAEA | 5 | 45.46 | HHHHH | 7 | -0.32 |
| 964 | 1EYQ:B | isomerase | 135 | 139 | FAEAF | 5 | 23.30 | HHHHH | 7 | 1.14 |
| 965 | 1HZT:A | isomerase | 113 | 117 | VENEV | 5 | 47.06 | EEEEE | 10 | -0.42 |
| 966 | 1OU0:A | isomerase | 25 | 29 | IDPDI | 5 | 57.26 | SCTTC | 4 | 0.08 |
| 967 | 1P1J:B | isomerase | 57 | 61 | KLDLK | 5 | 52.42 | EEECC | 7 | -0.74 |
| 968 | 1P1J:B | isomerase | 47 | 51 | VTPTV | 5 | 53.78 | EEEEE | 6 | 1.08 |
| 969 | 1TQJ:C | isomerase | 60 | 65 | LTKKTL | 6 | 61.87 | GCCSEE | 9 | -0.27 |
| 970 | 1W2W:J | isomerase | 340 | 344 | VTGTV | 5 | 19.54 | EEEEE | 8 | 1.32 |
| 971 | 1WP5:A | isomerase | 314 | 318 | LAAAL | 5 | 33.44 | HHHHH | 8 | 2.60 |
| 972 | 1ZVT:A | isomerase | 613 | 617 | ARNRA | 5 | 79.32 | CCSTT | 8 | -1.78 |
| 973 | 2BI7:A | isomerase | 241 | 245 | GYQYG | 5 | 72.62 | TTTTC | 5 | -1.38 |
| 974 | 2BI7:A | isomerase | 311 | 316 | IPYYPI | 6 | 34.28 | CCCEEC | 5 | 0.53 |
| 975 | 2DWU:C | isomerase | 79 | 84 | TAAAAT | 6 | 7.07 | HHHHHH | 9 | 0.97 |
| 976 | 2Q4I:A | isomerase | 174 | 178 | EPAPE | 5 | 7.51 | CCCHH | 13 | -1.68 |
| 977 | 2QJV:B | isomerase | 6 | 10 | STCTS | 5 | 65.84 | ECCSC | 6 | -0.10 |
| 978 | 2QJV:B | isomerase | 124 | 128 | EVGVE | 5 | 62.04 | GSEEE | 6 | 0.20 |
| 979 | 2V6K:A | isomerase | 203 | 207 | AAPAA | 5 | 38.64 | HSGGG | 5 | 1.12 |
| 980 | 2VXN:A | isomerase | 181 | 185 | EQAQE | 5 | 61.26 | HHHHH | 7 | -2.44 |
| 981 | 2VXN:A | isomerase | 216 | 220 | AANAA | 5 | 42.04 | TTTHH | 6 | 0.74 |
| 982 | 2WF7:A | isomerase | 133 | 137 | DAIAD | 5 | 44.70 | SEECC | 7 | 0.22 |
| 983 | 2WF7:A | isomerase | 157 | 161 | AVGVA | 5 | 42.18 | HTTCC | 7 | 2.32 |
| 984 | 2WFI:A | isomerase | 48 | 52 | KGTGK | 5 | 79.06 | TCBCT | 7 | -1.86 |
| 985 | 2XHG:A | isomerase | 184 | 188 | QSWSQ | 5 | 29.50 | HHHHH | 8 | -1.90 |
| 986 | 2XHG:A | isomerase | 266 | 270 | ALGLA | 5 | 3.90 | HHHHH | 8 | 2.16 |
| 987 | 2Y0O:A | isomerase | 91 | 95 | VYLYV | 5 | 6.04 | EEEEE | 10 | 1.92 |
| 988 | 2ZPU:A | isomerase | 182 | 187 | LGGGGL | 6 | 5.57 | CSSSHH | 9 | 1.00 |
| 989 | 3A9S:C | isomerase | 71 | 79 | KEAAEAAEK | 9 | 62.06 | HHHHHHHHH | 8 | -1.23 |
| 990 | 3A9S:C | isomerase | 40 | 44 | KAVAK | 5 | 54.74 | HHHHH | 8 | 0.00 |
| 991 | 3A9S:C | isomerase | 73 | 77 | AAEAA | 5 | 47.26 | HHHHH | 8 | 0.74 |
| 992 | 3BZN:A | isomerase | 122 | 126 | TLRLT | 5 | 14.28 | EEEEE | 11 | 0.34 |
| 993 | 3CU2:B | isomerase | 83 | 87 | VAKAV | 5 | 29.46 | HHHHH | 9 | 1.62 |
| 994 | 3EY6:A | isomerase | 40 | 44 | LGNGL | 5 | 60.96 | SSSSS | 5 | 0.66 |
| 995 | 3GM5:A | isomerase | 58 | 62 | KARAK | 5 | 51.00 | CCCEE | 5 | -1.74 |
| 996 | 3IGS:B | isomerase | 69 | 73 | IIGII | 5 | 5.24 | EEEEC | 10 | 3.52 |
| 997 | 3IMH:A | isomerase | 218 | 222 | RKTKR | 5 | 97.64 | SSCEE | 6 | -3.50 |
| 998 | 3IMH:A | isomerase | 93 | 97 | NDGDN | 5 | 62.98 | CBTTB | 6 | -2.88 |
| 999 | 3L2H:A | isomerase | 100 | 104 | HAAAH | 5 | 32.46 | TSCCE | 7 | -0.20 |
| 1000 | 3LJK:A | isomerase | 127 | 131 | GRWRG | 5 | 55.88 | TCSBC | 9 | -2.14 |
| 1001 | 3LJK:A | isomerase | 417 | 421 | AQSQA | 5 | 41.12 | HHHHH | 6 | -0.84 |
| 1002 | 3LJK:A | isomerase | 272 | 276 | IAFAI | 5 | 2.68 | HHHHH | 6 | 3.08 |
| 1003 | 3NO0:C | isomerase | 511 | 515 | ELPLE | 5 | 106.82 | GCCSS | 4 | -0.20 |
| 1004 | 3NRE:A | isomerase | 213 | 217 | EQPQE | 5 | 49.54 | EEGGG | 7 | -3.12 |
| 1005 | 3NRE:A | isomerase | 277 | 282 | ESTTSE | 6 | 56.03 | CEEEEE | 7 | -1.67 |
| 1006 | 3NRE:A | isomerase | 127 | 131 | LTVTL | 5 | 18.14 | EEEEE | 9 | 2.08 |
| 1007 | 3PWT:A | isomerase | 206 | 210 | ERERE | 5 | 64.66 | HHHHH | 13 | -3.90 |
| 1008 | 3PWT:A | isomerase | 484 | 488 | KELEK | 5 | 76.58 | HHHHH | 13 | -2.20 |
| 1009 | 3PWT:A | isomerase | 489 | 493 | RGIGR | 5 | 36.00 | TTCCC | 11 | -1.06 |
| 1010 | 3RMI:A | isomerase | 60 | 64 | KRLRK | 5 | 70.08 | HHHHH | 8 | -2.60 |
| 1011 | 3RMI:A | isomerase | 71 | 75 | FDPDF | 5 | 83.50 | CCHHH | 7 | -0.60 |
| 1012 | 3RMI:A | isomerase | 24 | 28 | LIHIL | 5 | 95.26 | HHHHH | 8 | 2.68 |
| 1013 | 3RYK:B | isomerase | 83 | 87 | DVIVD | 5 | 0.04 | EEEEE | 15 | 1.18 |
| 1014 | 3S46:B | isomerase | 200 | 204 | ASNSA | 5 | 7.42 | EECHH | 7 | -0.30 |
| 1015 | 3S46:B | isomerase | 48 | 52 | AVAVA | 5 | 13.32 | HHHHH | 6 | 2.76 |
| 1016 | 1GYX:B | isomerase | 18 | 22 | AALAA | 5 | 31.02 | HHHHH | 8 | 2.20 |
| 1017 | 2Y0O:A | isomerase | 141 | 146 | AGEEGA | 6 | 51.70 | EEEEEE | 10 | -0.70 |
| 1018 | 3BZN:A | isomerase | 208 | 213 | AAMMAA | 6 | 12.00 | HHHHHH | 11 | 1.83 |
| 1019 | 3CU2:B | isomerase | 10 | 15 | LKQQKL | 6 | 48.78 | HTTCCE | 9 | -1.20 |
| 1020 | 3NO8:B | isomerase regulator | 506 | 510 | GNNNG | 5 | 47.46 | SSSTT | 7 | -2.26 |
| 1021 | 3NO8:B | isomerase regulator | 466 | 470 | TACAT | 5 | 10.32 | EEEEE | 10 | 0.94 |
| 1022 | 3NO8:B | isomerase regulator | 498 | 502 | CFTFC | 5 | 30.34 | EEEEE | 10 | 1.98 |
| 1023 | 1JLY:B | lectin | 168 | 173 | KGNNGK | 6 | 46.70 | ECTTSC | 6 | -2.60 |
| 1024 | 1JLY:B | lectin | 191 | 195 | DNLND | 5 | 86.84 | CCTTC | 6 | -2.04 |
| 1025 | 1JLY:B | lectin | 294 | 299 | LEIIEL | 6 | 52.77 | EEEEEC | 6 | 1.60 |
| 1026 | 1A78:B | lectin | 96 | 100 | KIIIK | 5 | 28.34 | EEEEE | 8 | 1.14 |
| 1027 | 2CHH:A | lectin | 41 | 45 | TGSGT | 5 | 51.34 | EEEEE | 7 | -0.60 |
| 1028 | 1A92:D | leucine zipper | 39 | 43 | KKIKK | 5 | 99.26 | HHHHH | 8 | -2.22 |
| 1029 | 1A92:D | leucine zipper | 55 | 60 | KGIIGK | 6 | 69.33 | HHHTTC | 7 | 0.07 |
| 1030 | 1LKE:A | ligand binding protein | 53 | 57 | SVKVS | 5 | 29.36 | EEEEE | 7 | 0.58 |
| 1031 | 1LKE:A | ligand binding protein | 127 | 131 | LVWVL | 5 | 14.46 | EEEEE | 7 | 3.02 |
| 1032 | 1MKY:A | ligand binding protein | 279 | 283 | RQDQR | 5 | 89.96 | HHHHH | 5 | -3.90 |
| 1033 | 1MKY:A | ligand binding protein | 124 | 128 | REFER | 5 | 78.90 | HHHHH | 5 | -2.64 |
| 1034 | 1X8Q:A | ligand binding protein | 42 | 46 | AALAA | 5 | 6.26 | EEEEE | 8 | 2.20 |
| 1035 | 2QEB:A | ligand binding protein | 136 | 140 | KDYDK | 5 | 79.38 | HHHHH | 9 | -3.22 |
| 1036 | 2QEB:A | ligand binding protein | 49 | 53 | DGRGD | 5 | 33.52 | TCCBC | 9 | -2.46 |
| 1037 | 1V4P:A | ligase | 63 | 69 | NEKVKEN | 7 | 77.30 | HHHHHHT | 9 | -2.51 |
| 1038 | 3CEG:A | ligase | 4560 | 4564 | SLPLS | 5 | 37.64 | HCCCC | 8 | 0.88 |
| 1039 | 3FMF:D | ligase | 8 | 12 | GTGTG | 5 | 33.48 | ESSSS | 4 | -0.52 |
| 1040 | 3FMF:D | ligase | 128 | 132 | AVDVA | 5 | 52.18 | HHHTT | 6 | 1.70 |
| 1041 | 1BYI:A | ligase | 25 | 29 | AAKAA | 5 | 58.18 | HHHHT | 6 | 0.66 |
| 1042 | 1JW9:B | ligase | 123 | 127 | DLVLD | 5 | 15.96 | SEEEE | 10 | 0.96 |
| 1043 | 1V4P:A | ligase | 56 | 60 | REIER | 5 | 68.88 | HHHHH | 10 | -2.30 |
| 1044 | 1V4P:A | ligase | 64 | 68 | EKVKE | 5 | 83.92 | HHHHH | 10 | -2.12 |
| 1045 | 1V4P:A | ligase | 62 | 70 | ANEKVKENA | 9 | 61.73 | HHHHHHHTC | 9 | -1.56 |
| 1046 | 1WL8:A | ligase | 2 | 6 | MIVIM | 5 | 4.86 | EEEEE | 10 | 3.40 |
| 1047 | 1Y42:X | ligase | 60 | 64 | KKGKK | 5 | 112.58 | HTTSS | 6 | -3.20 |
| 1048 | 1Y42:X | ligase | 160 | 164 | TMNMT | 5 | 61.84 | HHHHH | 6 | -0.22 |
| 1049 | 1Y42:X | ligase | 345 | 349 | FYGYF | 5 | 1.00 | HHHHH | 6 | 0.52 |
| 1050 | 1Z2U:A | ligase | 112 | 116 | DPNPD | 5 | 58.88 | SCCTT | 6 | -2.74 |
| 1051 | 1ZVD:A | ligase | 654 | 658 | KWFWK | 5 | 60.52 | HHHHH | 8 | -1.36 |
| 1052 | 2A25:A | ligase | 247 | 251 | IATAI | 5 | 32.14 | SHHHH | 5 | 2.38 |
| 1053 | 2E10:B | ligase | 126 | 130 | LGIGL | 5 | 6.66 | EEEEE | 9 | 2.26 |
| 1054 | 2IP1:A | ligase | 365 | 369 | KQIQK | 5 | 59.70 | HHHHH | 6 | -2.06 |
| 1055 | 2IP1:A | ligase | 200 | 204 | KFLFK | 5 | 71.12 | HHHHC | 7 | 0.32 |
| 1056 | 2P64:B | ligase | 131 | 135 | EKEKE | 5 | 79.36 | HHHHH | 7 | -3.66 |
| 1057 | 2PIE:A | ligase | 119 | 123 | EYEYE | 5 | 32.26 | SEEEE | 8 | -2.62 |
| 1058 | 2W6P:B | ligase | 276 | 280 | EFLFE | 5 | 19.60 | EEEEE | 13 | 0.48 |
| 1059 | 2W6P:B | ligase | 260 | 264 | CAKAC | 5 | 22.02 | HHHHH | 13 | 0.94 |
| 1060 | 2XGT:A | ligase | 273 | 277 | VQTQV | 5 | 23.90 | ESCCS | 6 | 0.14 |
| 1061 | 2Y27:A | ligase | 375 | 379 | EPCPE | 5 | 61.62 | CBCTT | 10 | -1.54 |
| 1062 | 2YVQ:A | ligase | 1370 | 1374 | FRPRF | 5 | 63.90 | GHHHH | 9 | -1.00 |
| 1063 | 2YVQ:A | ligase | 1361 | 1365 | GILIG | 5 | 3.94 | EEEEE | 8 | 2.40 |
| 1064 | 2ZP1:A | ligase | 295 | 299 | LIKIL | 5 | 41.34 | HHHHH | 6 | 2.54 |
| 1065 | 3C8Z:B | ligase | 13 | 17 | PGRGP | 5 | 71.84 | SSCCS | 3 | -1.70 |
| 1066 | 3C8Z:B | ligase | 33 | 37 | TPGPT | 5 | 70.58 | CCCSS | 3 | -1.00 |
| 1067 | 3CEG:A | ligase | 4557 | 4561 | LSTSL | 5 | 48.84 | HHHHC | 5 | 1.06 |
| 1068 | 3FMF:D | ligase | 7 | 11 | TGTGT | 5 | 16.50 | EESSS | 5 | -0.58 |
| 1069 | 3FMF:D | ligase | 126 | 130 | DVAVD | 5 | 36.58 | HHHHH | 7 | 0.64 |
| 1070 | 3HXW:A | ligase | 310 | 315 | RRIIRR | 6 | 18.08 | HHHHHH | 13 | -1.50 |
| 1071 | 3IVV:A | ligase | 83 | 87 | YLSLY | 5 | 11.18 | EEEEE | 11 | 0.84 |
| 1072 | 3KIZ:B | ligase | 196 | 200 | YETEY | 5 | 66.12 | TCSSC | 6 | -2.06 |
| 1073 | 3NUA:B | ligase | 231 | 235 | NRLRN | 5 | 90.26 | HHHTC | 7 | -2.44 |
| 1074 | 3O0A:B | ligase | 319 | 323 | TRERT | 5 | 77.32 | HHHHH | 7 | -2.78 |
| 1075 | 3O0A:B | ligase | 385 | 389 | KVVVK | 5 | 37.38 | CCCEE | 11 | 0.96 |
| 1076 | 3O0A:B | ligase | 288 | 292 | LVPVL | 5 | 17.88 | HHHHH | 7 | 2.88 |
| 1077 | 3ONH:A | ligase | 487 | 491 | FDYDF | 5 | 65.08 | EETTB | 4 | -0.54 |
| 1078 | 3OQI:A | ligase | 157 | 161 | GRARG | 5 | 55.78 | HHHHH | 4 | -1.60 |
| 1079 | 3PG6:D | ligase | 632 | 636 | TIVIT | 5 | 1.10 | EEEEE | 11 | 2.36 |
| 1080 | 3PT3:A | ligase | 2783 | 2787 | LKQKL | 5 | 50.92 | HHHHH | 8 | -0.74 |
| 1081 | 3V3L:A | ligase | 163 | 167 | RKIKR | 5 | 70.14 | EEEEE | 7 | -2.46 |
| 1082 | 1JW9:B | ligase | 38 | 43 | GLGGLG | 6 | 4.20 | CCSHHH | 9 | 1.00 |
| 1083 | 2ZP1:A | ligase | 294 | 300 | ELIKILE | 7 | 48.59 | HHHHHHH | 8 | 0.81 |
| 1084 | 2AXI:A | ligase/ligase inhibitor | 34 | 38 | LLKLL | 5 | 31.78 | HHHHH | 7 | 2.26 |
| 1085 | 3BUX:B | ligase/signaling protein | 74 | 78 | KLALK | 5 | 69.42 | GGCCC | 6 | 0.32 |
| 1086 | 1C44:A | lipid binding protein | 14 | 18 | KEIEK | 5 | 83.44 | HHHHH | 8 | -2.06 |
| 1087 | 1MID:A | lipid binding protein | 60 | 64 | NLNLN | 5 | 80.84 | TCCHH | 6 | -0.58 |
| 1088 | 1PZX:A | lipid binding protein | 268 | 272 | GAHAG | 5 | 7.04 | HHHHC | 8 | -0.08 |
| 1089 | 1PZX:A | lipid binding protein | 124 | 128 | LGQGL | 5 | 1.00 | HHHHH | 9 | 0.66 |
| 1090 | 2AG4:B | lipid binding protein | 94 | 98 | EPCPE | 5 | 74.54 | SCCCT | 7 | -1.54 |
| 1091 | 2AG4:B | lipid binding protein | 106 | 110 | PCHCP | 5 | 43.90 | CCSSC | 7 | -0.28 |
| 1092 | 2IN5:B | lipid binding protein | 82 | 86 | LNNNL | 5 | 51.06 | SSSCE | 7 | -0.58 |
| 1093 | 2YH6:A | lipid binding protein | 133 | 137 | YRGRY | 5 | 34.90 | EEEEE | 11 | -2.40 |
| 1094 | 3BQP:B | lipid binding protein | 58 | 62 | LIEIL | 5 | 33.68 | HHHHH | 6 | 2.62 |
| 1095 | 3MH9:C | lipid binding protein | 103 | 108 | DFVVFD | 6 | 42.65 | EEEEET | 8 | 1.17 |
| 1096 | 3PQS:A | lipid binding protein | 165 | 169 | PNYNP | 5 | 73.78 | CSBCS | 5 | -2.30 |
| 1097 | 3PQS:A | lipid binding protein | 71 | 75 | DTVTD | 5 | 54.86 | EEECC | 5 | -0.84 |
| 1098 | 3PQS:A | lipid binding protein | 158 | 162 | ADLDA | 5 | 38.50 | EETTS | 5 | 0.08 |
| 1099 | 2YH6:A | lipid binding protein | 132 | 138 | QYRGRYQ | 7 | 36.74 | CEEEEEE | 15 | -2.71 |
| 1100 | 3BQP:B | lipid binding protein | 57 | 63 | VLIEILV | 7 | 39.64 | HHHHHHT | 5 | 3.07 |
| 1101 | 2QZT:B | lipid transport | 61 | 65 | VTVTV | 5 | 15.52 | EEEEE | 7 | 2.24 |
| 1102 | 2RA2:F | lipoprotein | 24 | 28 | TDNDT | 5 | 29.66 | ECTTT | 11 | -2.38 |
| 1103 | 3CFU:A | lipoprotein | 193 | 198 | TNSSNT | 6 | 39.68 | CCSTTS | 7 | -1.67 |
| 1104 | 3GE2:A | lipoprotein | 106 | 110 | QKVKQ | 5 | 97.78 | EEEEE | 8 | -2.12 |
| 1105 | 3GE2:A | lipoprotein | 75 | 79 | GTYTG | 5 | 33.96 | EEEEE | 8 | -0.70 |
| 1106 | 3GE2:A | lipoprotein | 91 | 96 | TGTTGT | 6 | 53.12 | ETTEEE | 6 | -0.60 |
| 1107 | 2G6Y:B | luminescent protein | 153 | 159 | SFTRTFS | 7 | 9.50 | EEEEEET | 12 | -0.27 |
| 1108 | 3A35:B | luminescent protein | 146 | 151 | SIEEIS | 6 | 60.17 | ECCEEE | 8 | 0.07 |
| 1109 | 2G6Y:B | luminescent protein | 154 | 158 | FTRTF | 5 | 9.00 | EEEEE | 7 | -0.06 |
| 1110 | 2Y8N:B | lyase | 59 | 65 | LGTCTGL | 7 | 34.86 | EEEECSS | 10 | 1.13 |
| 1111 | 2PTZ:A | lyase | 19 | 25 | TVEVEVT | 7 | 9.80 | EEEEEEE | 12 | 0.60 |
| 1112 | 3ELF:A | lyase | 149 | 155 | KAAAAAK | 7 | 55.66 | HHHHHTT | 5 | 0.17 |
| 1113 | 1N7O:A | lyase | 462 | 466 | RSISR | 5 | 21.10 | GGGGC | 10 | -1.22 |
| 1114 | 1F61:A | lyase | 251 | 257 | GERTREG | 7 | 98.57 | EEECTTS | 3 | -2.50 |
| 1115 | 1LC5:A | lyase | 154 | 159 | TPNNPT | 6 | 5.52 | SSCTTT | 9 | -1.93 |
| 1116 | 1F61:A | lyase | 252 | 256 | ERTRE | 5 | 119.60 | EECTT | 5 | -3.34 |
| 1117 | 1F61:A | lyase | 362 | 366 | AYGYA | 5 | 22.88 | HHHHH | 10 | 0.12 |
| 1118 | 1FX4:A | lyase | 1082 | 1086 | VPEPV | 5 | 79.34 | CSSCE | 5 | 0.34 |
| 1119 | 1GKM:A | lyase | 399 | 403 | SHPHS | 5 | 56.06 | CSTTC | 6 | -1.92 |
| 1120 | 1GKM:A | lyase | 91 | 95 | LDDDL | 5 | 31.34 | CCHHH | 6 | -0.58 |
| 1121 | 1GKM:A | lyase | 85 | 89 | AGIGA | 5 | 61.54 | CCEEE | 6 | 1.46 |
| 1122 | 1GKM:A | lyase | 78 | 82 | SLVLS | 5 | 46.70 | HHHHH | 6 | 2.04 |
| 1123 | 1GXM:B | lyase | 545 | 549 | TQPQT | 5 | 30.42 | TSCCC | 10 | -2.00 |
| 1124 | 1JS3:B | lyase | 112 | 116 | TELET | 5 | 2.50 | HHHHH | 14 | -0.92 |
| 1125 | 1JS3:B | lyase | 43 | 47 | PATAP | 5 | 50.80 | CSSCC | 5 | -0.06 |
| 1126 | 1LC5:A | lyase | 222 | 226 | GLRLG | 5 | 36.80 | TTCCE | 9 | 0.46 |
| 1127 | 1LC5:A | lyase | 319 | 323 | RILIR | 5 | 24.52 | TEECE | 9 | 0.76 |
| 1128 | 1LC5:A | lyase | 178 | 182 | LNINL | 5 | 39.08 | HTCEE | 9 | 1.02 |
| 1129 | 1LC5:A | lyase | 250 | 255 | ALAALA | 6 | 15.12 | HHHHHH | 7 | 2.47 |
| 1130 | 1LC5:A | lyase | 149 | 153 | CLFLC | 5 | 4.84 | EEEEE | 7 | 3.08 |
| 1131 | 1N7O:A | lyase | 459 | 463 | SRGRS | 5 | 9.66 | GSGGG | 7 | -2.20 |
| 1132 | 1N7O:A | lyase | 172 | 176 | DTYTD | 5 | 50.86 | SHHHH | 7 | -1.94 |
| 1133 | 1N7O:A | lyase | 214 | 218 | SSISS | 5 | 60.58 | HHSCC | 7 | 0.26 |
| 1134 | 1N7O:A | lyase | 561 | 565 | GFGFG | 5 | 3.66 | TEEEE | 7 | 0.88 |
| 1135 | 1Q6O:B | lyase | 40 | 44 | VGEGV | 5 | 17.40 | HHHCT | 11 | 0.82 |
| 1136 | 1QCX:A | lyase | 228 | 232 | NYFYN | 5 | 18.18 | CEEES | 5 | -1.36 |
| 1137 | 1QCX:A | lyase | 319 | 325 | SGSMSGS | 7 | 50.73 | CCCCCCB | 5 | -0.30 |
| 1138 | 1QCX:A | lyase | 320 | 324 | GSMSG | 5 | 49.30 | CCCCC | 4 | -0.10 |
| 1139 | 1QCX:A | lyase | 48 | 52 | TFDFT | 5 | 45.20 | EEECT | 5 | 0.14 |
| 1140 | 1QIP:D | lyase | 155 | 159 | GKMKG | 5 | 45.28 | SSSTT | 8 | -1.34 |
| 1141 | 1QIP:D | lyase | 97 | 101 | TLELT | 5 | 4.92 | EEEEE | 11 | 0.54 |
| 1142 | 1QOP:A | lyase | 198 | 202 | EKLKE | 5 | 78.46 | HHHHH | 8 | -2.20 |
| 1143 | 1QOP:A | lyase | 169 | 173 | YGRGY | 5 | 33.62 | HCCSC | 8 | -1.58 |
| 1144 | 1QOP:A | lyase | 42 | 46 | DAGAD | 5 | 34.84 | HTTCS | 8 | -0.76 |
| 1145 | 1QOP:A | lyase | 226 | 231 | AGAAGA | 6 | 11.73 | TTCSEE | 8 | 1.07 |
| 1146 | 1QOP:B | lyase | 41 | 45 | FQAQF | 5 | 46.54 | HHHHH | 9 | 0.08 |
| 1147 | 1QOP:B | lyase | 353 | 357 | HALAH | 5 | 6.22 | HHHHH | 9 | 0.20 |
| 1148 | 1QOP:B | lyase | 119 | 123 | SALAS | 5 | 1.74 | HHHHH | 9 | 1.16 |
| 1149 | 1QOP:B | lyase | 118 | 124 | ASALASA | 7 | 9.89 | HHHHHHH | 9 | 1.34 |
| 1150 | 1R6D:A | lyase | 228 | 232 | ALVLA | 5 | 21.72 | HHHHH | 8 | 3.08 |
| 1151 | 1SJW:A | lyase | 134 | 138 | DPWPD | 5 | 87.64 | SCCCC | 7 | -2.22 |
| 1152 | 1SJW:A | lyase | 80 | 84 | YLVLY | 5 | 42.04 | EEEEE | 10 | 1.84 |
| 1153 | 1TT8:A | lyase | 44 | 48 | TVSVT | 5 | 50.26 | CEEEE | 15 | 1.24 |
| 1154 | 1U83:A | lyase | 161 | 165 | EAGAE | 5 | 56.10 | HHTEE | 6 | -0.76 |
| 1155 | 1U83:A | lyase | 242 | 246 | RLGLR | 5 | 47.38 | HTTCS | 6 | -0.36 |
| 1156 | 1UAI:A | lyase | 86 | 90 | ASWSA | 5 | 27.70 | CCBCS | 8 | 0.22 |
| 1157 | 1YIS:A | lyase | 86 | 90 | AHNHA | 5 | 25.76 | HHHHH | 9 | -1.26 |
| 1158 | 1YIS:A | lyase | 429 | 433 | VRDRV | 5 | 84.16 | THHHH | 6 | -0.82 |
| 1159 | 1YIS:A | lyase | 37 | 41 | LWIWL | 5 | 13.78 | HHHHH | 9 | 2.06 |
| 1160 | 1YIS:A | lyase | 204 | 208 | FLTLF | 5 | 41.24 | HHHHT | 6 | 2.50 |
| 1161 | 1Z9W:A | lyase | 116 | 120 | RRSRR | 5 | 120.04 | EEECC | 7 | -3.76 |
| 1162 | 1Z9W:A | lyase | 92 | 96 | AVEVA | 5 | 16.16 | EEEEE | 9 | 1.70 |
| 1163 | 1ZMT:A | lyase | 143 | 147 | STYTS | 5 | 3.32 | HHHHH | 6 | -0.86 |
| 1164 | 1ZMT:A | lyase | 184 | 188 | PYFYP | 5 | 40.48 | CSSCB | 8 | -0.60 |
| 1165 | 1ZMT:A | lyase | 42 | 46 | EAFAE | 5 | 61.32 | HHHHH | 6 | -0.12 |
| 1166 | 1ZMT:A | lyase | 155 | 159 | LANAL | 5 | 6.94 | HHHHH | 6 | 1.54 |
| 1167 | 2CWS:A | lyase | 134 | 139 | TTGGTT | 6 | 36.88 | TTSCBC | 9 | -0.60 |
| 1168 | 2DVT:C | lyase | 253 | 257 | FNENF | 5 | 24.62 | HHHHE | 10 | -0.98 |
| 1169 | 2DVT:C | lyase | 73 | 77 | AIEIA | 5 | 37.48 | HHHHH | 8 | 1.82 |
| 1170 | 2EB4:B | lyase | 33 | 37 | EITIE | 5 | 70.98 | TCCHH | 7 | 0.26 |
| 1171 | 2EV1:B | lyase | 127 | 131 | VVLVV | 5 | 26.40 | HHHHH | 8 | 4.12 |
| 1172 | 2FP8:B | lyase | 309 | 313 | EQIQE | 5 | 15.54 | CEEEE | 11 | -1.90 |
| 1173 | 2GN4:A | lyase | 51 | 55 | EMAME | 5 | 64.38 | HHHHH | 12 | -0.28 |
| 1174 | 2GN4:A | lyase | 242 | 246 | LAKAL | 5 | 30.30 | HHHHH | 12 | 1.46 |
| 1175 | 2GN4:A | lyase | 329 | 333 | LLKLL | 5 | 95.82 | HHTTC | 5 | 2.26 |
| 1176 | 2GN4:A | lyase | 12 | 16 | TILIT | 5 | 2.58 | EEEEE | 12 | 2.28 |
| 1177 | 2H9D:A | lyase | 20 | 24 | IDLDI | 5 | 19.78 | HHHHH | 10 | 1.16 |
| 1178 | 2J5I:A | lyase | 118 | 122 | FGGGF | 5 | 10.22 | EGGGH | 6 | 0.88 |
| 1179 | 2OPI:A | lyase | 41 | 45 | SGTGS | 5 | 13.52 | EBTTC | 6 | -0.62 |
| 1180 | 2PTZ:A | lyase | 128 | 132 | LYRYL | 5 | 29.96 | HHHHH | 9 | 0.10 |
| 1181 | 2PTZ:A | lyase | 118 | 124 | KAAAAAK | 7 | 34.67 | HHHHHHH | 9 | 0.17 |
| 1182 | 2PTZ:A | lyase | 20 | 24 | VEVEV | 5 | 7.52 | EEEEE | 9 | 1.12 |
| 1183 | 2PTZ:A | lyase | 366 | 370 | SVMVS | 5 | 3.58 | EEEEE | 9 | 1.74 |
| 1184 | 2QX3:B | lyase | 192 | 196 | NYVYN | 5 | 30.64 | CEEEE | 13 | -1.08 |
| 1185 | 2QX3:B | lyase | 337 | 341 | VKAKV | 5 | 40.10 | HHHHH | 8 | 0.48 |
| 1186 | 2V9L:A | lyase | 195 | 199 | QATAQ | 5 | 46.10 | HHHHH | 15 | -0.82 |
| 1187 | 2V9L:A | lyase | 268 | 272 | LASAL | 5 | 34.86 | CHHHH | 15 | 2.08 |
| 1188 | 2VWS:A | lyase | 180 | 184 | LSASL | 5 | 17.50 | HHHHT | 6 | 1.56 |
| 1189 | 2WZ1:A | lyase | 458 | 462 | DTLTD | 5 | 37.50 | HHHHC | 9 | -0.92 |
| 1190 | 2X3H:C | lyase | 167 | 171 | GSYSG | 5 | 30.08 | EEECS | 9 | -0.74 |
| 1191 | 2X3H:C | lyase | 393 | 397 | GSMSG | 5 | 9.60 | CCTTS | 9 | -0.10 |
| 1192 | 2X3H:C | lyase | 19 | 23 | LYEYL | 5 | 46.80 | HHHHH | 13 | 0.30 |
| 1193 | 2X3H:C | lyase | 268 | 272 | DVLVD | 5 | 0.96 | EEEEE | 13 | 1.04 |
| 1194 | 2Y8N:B | lyase | 60 | 64 | GTCTG | 5 | 27.86 | EEECS | 8 | 0.06 |
| 1195 | 2ZUX:B | lyase | 146 | 150 | SYTYS | 5 | 50.12 | EECEE | 7 | -0.98 |
| 1196 | 2ZUX:B | lyase | 157 | 161 | GDVDG | 5 | 21.44 | ECSSS | 7 | -0.72 |
| 1197 | 3ANU:A | lyase | 27 | 31 | ERMRE | 5 | 69.10 | HHHHH | 9 | -2.82 |
| 1198 | 3ANU:A | lyase | 153 | 157 | ALELA | 5 | 35.98 | HHHHH | 9 | 1.54 |
| 1199 | 3AQI:B | lyase | 594 | 598 | LDRDL | 5 | 49.04 | TCTTT | 9 | -0.78 |
| 1200 | 3C70:A | lyase | 145 | 149 | GLKLG | 5 | 17.60 | EEECC | 22 | 0.58 |
| 1201 | 3C70:A | lyase | 35 | 39 | ALDLA | 5 | 18.96 | EECCT | 22 | 1.54 |
| 1202 | 3CLM:A | lyase | 298 | 302 | TAKAT | 5 | 57.26 | CCCCC | 8 | -0.34 |
| 1203 | 3CLM:A | lyase | 19 | 23 | LSRSL | 5 | 31.90 | CCHHH | 8 | 0.30 |
| 1204 | 3EPB:A | lyase | 241 | 245 | TIHIT | 5 | 6.54 | EEEEE | 11 | 0.88 |
| 1205 | 3F0D:D | lyase | 123 | 127 | DLPLD | 5 | 80.42 | TCCGG | 7 | -0.20 |
| 1206 | 3F9S:A | lyase | 78 | 82 | ADGDA | 5 | 36.36 | EETTE | 8 | -0.76 |
| 1207 | 3F9S:A | lyase | 86 | 90 | TWLWT | 5 | 32.98 | EEEEE | 9 | 0.12 |
| 1208 | 3F9T:B | lyase | 363 | 367 | VCNCV | 5 | 35.66 | ECSSS | 4 | 1.98 |
| 1209 | 3FCD:A | lyase | 73 | 77 | SDIDS | 5 | 61.12 | SCHHH | 5 | -0.82 |
| 1210 | 3H9M:A | lyase | 374 | 378 | NFDFN | 5 | 10.74 | CEEEE | 13 | -0.98 |
| 1211 | 3HHT:A | lyase | 15 | 19 | PHHHP | 5 | 82.62 | GGGSC | 3 | -2.56 |
| 1212 | 3HHT:A | lyase | 91 | 96 | LGYYGL | 6 | 36.63 | HTCCST | 3 | 0.70 |
| 1213 | 3HT1:A | lyase | 55 | 59 | HEWEH | 5 | 45.08 | CSSCE | 8 | -2.86 |
| 1214 | 3KUU:C | lyase | 131 | 135 | AANAA | 5 | 20.10 | HHHHH | 9 | 0.74 |
| 1215 | 3KUU:C | lyase | 90 | 94 | LVPVL | 5 | 53.24 | SSCEE | 5 | 2.88 |
| 1216 | 3KX6:D | lyase | 64 | 68 | KGLGK | 5 | 54.00 | TTGGG | 4 | -0.96 |
| 1217 | 3M0Z:A | lyase | 119 | 123 | PTGTP | 5 | 60.60 | CCSST | 3 | -1.00 |
| 1218 | 3M0Z:A | lyase | 171 | 175 | AEFEA | 5 | 30.80 | HHHHH | 10 | -0.12 |
| 1219 | 3M0Z:A | lyase | 47 | 51 | VASAV | 5 | 36.28 | HHHHH | 10 | 2.24 |
| 1220 | 3MOE:A | lyase | 453 | 457 | GVFVG | 5 | 2.42 | HHHHH | 7 | 2.08 |
| 1221 | 3N3M:A | lyase | 35 | 39 | NEKEN | 5 | 71.14 | HHHHT | 9 | -3.58 |
| 1222 | 3N3M:A | lyase | 79 | 83 | FFYFF | 5 | 14.34 | HHHHH | 7 | 1.98 |
| 1223 | 3O7I:B | lyase | 97 | 103 | RLAQALR | 7 | 78.51 | HHHHHHH | 8 | -0.19 |
| 1224 | 3O7I:B | lyase | 98 | 102 | LAQAL | 5 | 51.34 | HHHHH | 8 | 1.54 |
| 1225 | 3OKF:B | lyase | 356 | 360 | IAQAI | 5 | 24.24 | HHHHH | 8 | 1.82 |
| 1226 | 3PJ0:D | lyase | 354 | 358 | LEKEL | 5 | 57.78 | HHHHC | 6 | -0.66 |
| 1227 | 3PJ0:D | lyase | 200 | 204 | SVYVS | 5 | 6.12 | EEEEE | 11 | 1.10 |
| 1228 | 3PJ0:D | lyase | 212 | 216 | IAGAI | 5 | 1.86 | SSCEE | 6 | 2.44 |
| 1229 | 3R5G:B | lyase | 364 | 368 | ESASE | 5 | 49.84 | HHHCC | 10 | -1.36 |
| 1230 | 3TC7:A | lyase | 241 | 245 | EKIKE | 5 | 64.56 | THHHH | 6 | -2.06 |
| 1231 | 3V9O:A | lyase | 20 | 24 | RLYLR | 5 | 93.26 | EEEEE | 5 | -0.54 |
| 1232 | 3ZQU:A | lyase | 70 | 74 | GAAAG | 5 | 55.36 | TCCTT | 4 | 0.92 |
| 1233 | 3ZW5:B | lyase | 133 | 137 | RDPDR | 5 | 48.26 | ECTTC | 7 | -3.52 |
| 1234 | 7ODC:A | lyase | 203 | 207 | TDPDT | 5 | 68.46 | CCTHH | 5 | -2.00 |
| 1235 | 1PRZ:A | lyase | 223 | 227 | RLRLR | 5 | 54.26 | EEEEE | 9 | -1.18 |
| 1236 | 1XQO:A | lyase | 104 | 109 | ARKKRA | 6 | 47.05 | HHHHHH | 14 | -2.20 |
| 1237 | 2FP8:B | lyase | 33 | 37 | EILIE | 5 | 23.94 | EEEEE | 11 | 1.16 |
| 1238 | 3M0Z:A | lyase | 36 | 40 | LVGVL | 5 | 13.30 | EEEEE | 10 | 3.12 |
| 1239 | 3N3M:A | lyase | 39 | 43 | NNYNN | 5 | 65.40 | TTTHH | 7 | -3.06 |
| 1240 | 1GXM:B | lyase | 378 | 383 | NGGGGN | 6 | 76.88 | CCCCCS | 5 | -1.43 |
| 1241 | 2QX3:B | lyase | 191 | 197 | YNYVYNY | 7 | 24.34 | SCEEEEE | 21 | -1.14 |
| 1242 | 1QCX:A | lyase | 1 | 6 | AGVVGA | 6 | 69.30 | CCCCSC | 2 | 1.87 |
| 1243 | 3KUU:C | lyase | 134 | 139 | AALLAA | 6 | 14.13 | HHHHHH | 7 | 2.47 |
| 1244 | 1QIP:D | lyase | 106 | 111 | TEDDET | 6 | 96.05 | GGGCTT | 5 | -2.57 |
| 1245 | 1PRZ:A | lyase | 224 | 228 | LRLRL | 5 | 47.54 | EEEEE | 8 | 0.48 |
| 1246 | 2FP8:B | lyase | 111 | 116 | LQNNQL | 6 | 69.92 | TTTTEE | 3 | -1.07 |
| 1247 | 3PKV:A | lyase | 62 | 66 | AINIA | 5 | 2.64 | EEEEC | 13 | 1.82 |
| 1248 | 3CLM:A | lyase | 173 | 178 | AYAAYA | 6 | 6.58 | HHHHHH | 9 | 0.77 |
| 1249 | 1GK8:A | lyase | 314 | 318 | LAKAL | 5 | 1.56 | HHHHH | 9 | 1.46 |
| 1250 | 1GK8:A | lyase | 55 | 59 | AAVAA | 5 | 3.18 | HHHHH | 9 | 2.28 |
| 1251 | 1QOP:B | lyase | 121 | 125 | LASAL | 5 | 28.80 | HHHHH | 7 | 2.08 |
| 1252 | 1QOP:B | lyase | 355 | 359 | LAHAL | 5 | 6.92 | HHHHH | 8 | 1.60 |
| 1253 | 3NQA:B | lyase/lyase inhibitor | 59 | 63 | FRKRF | 5 | 85.86 | HHHHH | 8 | -1.46 |
| 1254 | 3RQ5:A | lyase/lyase substrate | 46 | 50 | AGLGA | 5 | 5.14 | HHHHH | 8 | 1.32 |
| 1255 | 3RQ5:A | lyase/lyase substrate | 30 | 34 | ALLLA | 5 | 0.00 | EEEEC | 10 | 3.00 |
| 1256 | 1URS:A | maltose-binding protein | 270 | 274 | TPWPT | 5 | 14.28 | ECCCC | 6 | -1.10 |
| 1257 | 1URS:A | maltose-binding protein | 186 | 190 | GGYGG | 5 | 23.58 | HHTTC | 6 | -0.58 |
| 1258 | 1URS:A | maltose-binding protein | 135 | 139 | SVPVS | 5 | 1.82 | SEEEE | 6 | 1.04 |
| 1259 | 1K4Z:A | membrane protein | 1423 | 1427 | SETES | 5 | 47.42 | EEEEE | 9 | -1.86 |
| 1260 | 1K4Z:A | membrane protein | 1481 | 1485 | TSCST | 5 | 58.48 | EESCE | 9 | -0.10 |
| 1261 | 1K4Z:A | membrane protein | 1408 | 1412 | QVLVQ | 5 | 42.88 | EEEEE | 9 | 1.04 |
| 1262 | 1NKZ:F | membrane protein | 1 | 5 | ATLTA | 5 | 63.12 | CCCCH | 7 | 1.20 |
| 1263 | 1S0P:A | membrane protein | 142 | 146 | ISESI | 5 | 11.10 | HHTSG | 8 | 0.78 |
| 1264 | 1T1D:A | membrane protein | 157 | 161 | ERYRE | 5 | 75.26 | HHHHH | 9 | -3.46 |
| 1265 | 1T1D:A | membrane protein | 134 | 138 | PVNVP | 5 | 69.66 | CTTSC | 4 | 0.34 |
| 1266 | 2HFE:D | membrane protein | 117 | 121 | REQER | 5 | 133.06 | HHHHH | 5 | -3.90 |
| 1267 | 2JIL:A | membrane protein | 150 | 154 | TVEVT | 5 | 61.40 | EEEEE | 5 | 0.70 |
| 1268 | 2VKN:A | membrane protein | 15 | 20 | ADDDDA | 6 | 90.60 | CCSSST | 4 | -1.73 |
| 1269 | 2X27:X | membrane protein | 23 | 27 | DSSSD | 5 | 45.82 | CEECC | 8 | -1.88 |
| 1270 | 2X27:X | membrane protein | 193 | 197 | VDVDV | 5 | 28.46 | EEEEC | 8 | 1.12 |
| 1271 | 2Y8D:A | membrane protein | 2332 | 2336 | NKYKN | 5 | 62.88 | HHHHH | 5 | -3.22 |
| 1272 | 2Y8D:A | membrane protein | 2452 | 2456 | GDTDG | 5 | 71.38 | TCCTT | 6 | -1.70 |
| 1273 | 3D9S:D | membrane protein | 229 | 233 | SLSLS | 5 | 84.98 | CCCHH | 10 | 1.04 |
| 1274 | 3D9S:D | membrane protein | 97 | 101 | GAIAG | 5 | 17.54 | HHHHH | 10 | 1.46 |
| 1275 | 3D9S:D | membrane protein | 56 | 60 | LAQAL | 5 | 6.04 | HHHHH | 10 | 1.54 |
| 1276 | 3D9S:D | membrane protein | 220 | 224 | LYFYL | 5 | 46.36 | HHHHT | 10 | 1.56 |
| 1277 | 3O79:A | membrane protein | 170 | 174 | SNQNS | 5 | 79.62 | SSHHH | 4 | -2.42 |
| 1278 | 2WNK:A | membrane protein | 192 | 196 | ASRSA | 5 | 41.64 | CCCCC | 8 | -0.50 |
| 1279 | 2X27:X | membrane protein | 194 | 198 | DVDVD | 5 | 30.84 | EEECC | 11 | -0.42 |
| 1280 | 3CJJ:A | membrane protein, signaling protein | 206 | 210 | FSCSF | 5 | 6.80 | EEEEE | 8 | 1.30 |
| 1281 | 2CLB:A | metal binding protein | 40 | 45 | TYYYYT | 6 | 6.75 | HHHHHH | 14 | -1.10 |
| 1282 | 1QDD:A | metal binding protein | 50 | 54 | LVSVL | 5 | 10.60 | ECCCC | 8 | 3.04 |
| 1283 | 1RJU:V | metal binding protein | 3 | 7 | CQCQC | 5 | 55.16 | CCCCS | 5 | 0.10 |
| 1284 | 1X0G:D | metal binding protein | 67 | 71 | AAEAA | 5 | 20.40 | CTTHH | 5 | 0.74 |
| 1285 | 1Z6O:A | metal binding protein | 126 | 130 | LAKAL | 5 | 2.96 | HHHHH | 8 | 1.46 |
| 1286 | 2ANU:A | metal binding protein | 127 | 131 | EKLKE | 5 | 77.98 | HHHHH | 8 | -2.20 |
| 1287 | 2CLB:A | metal binding protein | 84 | 88 | LGGGL | 5 | 27.96 | TTCCC | 5 | 1.28 |
| 1288 | 2PVB:A | metal binding protein | 89 | 93 | GDKDG | 5 | 74.24 | HCTTC | 7 | -2.34 |
| 1289 | 2PVB:A | metal binding protein | 13 | 17 | AALAA | 5 | 43.02 | HHHHH | 6 | 2.20 |
| 1290 | 2Q0I:A | metal binding protein | 10 | 14 | LDDDL | 5 | 43.82 | EETTE | 6 | -0.58 |
| 1291 | 2Q0I:A | metal binding protein | 225 | 229 | LRGRL | 5 | 56.96 | EETHH | 6 | -0.36 |
| 1292 | 2Q0I:A | metal binding protein | 241 | 245 | CLRLC | 5 | 48.76 | HHHHH | 8 | 1.62 |
| 1293 | 2YWW:A | metal binding protein | 51 | 55 | KKGKK | 5 | 83.86 | TTEEE | 7 | -3.20 |
| 1294 | 2ZHJ:A | metal binding protein | 128 | 132 | YQQQY | 5 | 85.12 | HHHHH | 8 | -2.62 |
| 1295 | 2ZHJ:A | metal binding protein | 109 | 113 | LIEIL | 5 | 2.80 | HHHHH | 8 | 2.62 |
| 1296 | 3CZT:X | metal binding protein | 85 | 90 | HEFFEH | 6 | 149.40 | HHHSCC | 4 | -1.30 |
| 1297 | 3FSA:A | metal binding protein | 113 | 117 | AAHAA | 5 | 39.94 | SSSTT | 5 | 0.80 |
| 1298 | 3R2R:A | metal binding protein | 69 | 73 | PRMRP | 5 | 93.60 | CCCCC | 6 | -2.06 |
| 1299 | 3V5A:A | metal binding protein | 407 | 411 | LVPVL | 5 | 13.54 | CEEEE | 6 | 2.88 |
| 1300 | 2PVB:A | metal binding protein | 88 | 94 | DGDKDGD | 7 | 68.91 | HHCTTCS | 9 | -2.67 |
| 1301 | 3D2Q:D | metal binding, rna binding protein | 213 | 217 | TNDNT | 5 | 71.44 | TTTTE | 6 | -2.38 |
| 1302 | 2BDQ:B | metal transport | 183 | 187 | VGGGV | 5 | 22.60 | ECSSC | 6 | 1.44 |
| 1303 | 3RTL:A | metal transport | 432 | 436 | KVHVK | 5 | 63.94 | EEEEG | 10 | -0.52 |
| 1304 | 3V5U:A | metal transport | 58 | 62 | SAYAS | 5 | 33.82 | HHHHH | 11 | 0.14 |
| 1305 | 3V5U:A | metal transport | 68 | 72 | GISIG | 5 | 9.96 | HHHHH | 11 | 1.48 |
| 1306 | 3V5U:A | metal transport | 83 | 87 | GLVLG | 5 | 5.86 | HTHHH | 7 | 2.20 |
| 1307 | 3V5U:A | metal transport | 174 | 178 | GVLVG | 5 | 24.02 | HHHHH | 11 | 2.28 |
| 1308 | 4MT2:A | metallothionein | 33 | 37 | CCSCC | 5 | 44.04 | SSTTS | 3 | 1.84 |
| 1309 | 1HBN:F | methanogenesis | 60 | 64 | DEPED | 5 | 61.20 | CCCCC | 6 | -3.12 |
| 1310 | 1HBN:F | methanogenesis | 75 | 79 | GAKAG | 5 | 21.92 | HHHHT | 7 | -0.22 |
| 1311 | 1HBN:F | methanogenesis | 111 | 115 | GADAG | 5 | 2.08 | SCEEE | 7 | -0.14 |
| 1312 | 1HBN:A | methanogenesis | 413 | 417 | TAFAT | 5 | 2.96 | HHHHH | 9 | 1.00 |
| 1313 | 1HBN:A | methanogenesis | 402 | 407 | AAVVAA | 6 | 1.25 | CCCCCC | 7 | 2.60 |
| 1314 | 1HBN:F | methanogenesis | 74 | 80 | DGAKAGD | 7 | 29.34 | CCCCCCC | 8 | -1.16 |
| 1315 | 1MSK:A | methyltransferase | 1097 | 1101 | EAFAE | 5 | 17.64 | HHHHH | 5 | -0.12 |
| 1316 | 1MSK:A | methyltransferase | 913 | 917 | DFAFD | 5 | 64.64 | CCCCC | 5 | 0.08 |
| 1317 | 1MSK:A | methyltransferase | 1070 | 1074 | DALAD | 5 | 51.94 | HHHHH | 5 | 0.08 |
| 1318 | 3H8D:D | motor protein/signaling protein | 1208 | 1212 | ELNLE | 5 | 67.80 | CSCHH | 4 | -0.58 |
| 1319 | 1W4S:A | nuclear protein | 1088 | 1092 | VPLPV | 5 | 89.34 | SCCCC | 3 | 1.80 |
| 1320 | 1W9H:A | nuclear protein | 324 | 328 | LLTLL | 5 | 20.20 | EECCB | 9 | 2.90 |
| 1321 | 2CKX:A | nuclear protein | 583 | 587 | AEVEA | 5 | 42.58 | HHHHH | 10 | 0.16 |
| 1322 | 2J8Q:B | nuclear protein | 206 | 210 | PGYGP | 5 | 54.86 | HHHHH | 6 | -1.06 |
| 1323 | 2RH0:A | nuclear protein | 62 | 66 | LKGKL | 5 | 44.16 | EEEEB | 8 | -0.12 |
| 1324 | 2RH0:A | nuclear protein | 52 | 56 | VALAV | 5 | 11.70 | EEEEE | 10 | 3.16 |
| 1325 | 3D1B:C | nuclear protein | 476 | 480 | VLQLV | 5 | 13.10 | HHHHH | 8 | 2.50 |
| 1326 | 3MMY:B | nuclear protein | 202 | 206 | ELRLE | 5 | 15.66 | HHHHH | 12 | -0.78 |
| 1327 | 2J45:B | nucleotide binding | 53 | 57 | ERVRE | 5 | 71.46 | HHHHH | 14 | -2.36 |
| 1328 | 2J45:B | nucleotide binding | 14 | 18 | GRLRG | 5 | 77.36 | HTTTT | 13 | -1.20 |
| 1329 | 2J45:B | nucleotide binding | 17 | 21 | RGRGR | 5 | 113.98 | TTSSS | 13 | -2.86 |
| 1330 | 3B79:A | nucleotide binding protein | 102 | 106 | PISIP | 5 | 53.80 | CEEEE | 8 | 1.00 |
| 1331 | 3MDP:A | nucleotide binding protein | 132 | 136 | VAAAV | 5 | 43.42 | HHHHH | 6 | 2.76 |
| 1332 | 1GUQ:A | nucleotidyltransferase | 69 | 73 | YTGTY | 5 | 48.28 | CSSCE | 8 | -0.88 |
| 1333 | 1GUQ:A | nucleotidyltransferase | 255 | 259 | LALAL | 5 | 12.98 | HHHHH | 7 | 3.00 |
| 1334 | 1H7E:A | nucleotidyltransferase | 131 | 135 | AAEAA | 5 | 60.64 | HHHHT | 3 | 0.74 |
| 1335 | 3GIY:A | oxidoreductase | 151 | 157 | TTLVLTT | 7 | 17.89 | CEEEEEC | 10 | 1.29 |
| 1336 | 3BAL:D | oxidoreductase | 81 | 87 | GGEQEGG | 7 | 75.96 | TCGGGTS | 4 | -1.73 |
| 1337 | 1GVE:B | oxidoreductase | 58 | 62 | GLGLG | 5 | 32.54 | CCCTT | 3 | 1.28 |
| 1338 | 1SBY:B | oxidoreductase | 72 | 77 | KKLLKK | 6 | 78.58 | HHHHHH | 8 | -1.33 |
| 1339 | 1JF8:A | oxidoreductase | 66 | 72 | LIDNDIL | 7 | 68.13 | BCCHHHH | 5 | 0.87 |
| 1340 | 3OD3:A | oxidoreductase | 162 | 167 | IEDDEI | 6 | 43.58 | EECHHH | 10 | -0.83 |
| 1341 | 1N62:E | oxidoreductase | 462 | 468 | EQKAKQE | 7 | 60.76 | HHHHHHH | 12 | -2.86 |
| 1342 | 1SC6:A | oxidoreductase | 248 | 254 | ALADALA | 7 | 31.13 | HHHHHHH | 7 | 1.61 |
| 1343 | 3QY9:D | oxidoreductase | 25 | 31 | EIVGVIE | 7 | 21.77 | EEEEEEC | 10 | 1.43 |
| 1344 | 1K3I:A | oxidoreductase | 287 | 293 | GGSWSGG | 7 | 45.94 | CCCCCSS | 7 | -0.59 |
| 1345 | 3OJ0:A | oxidoreductase | 15 | 20 | KNGGNK | 6 | 70.82 | HHCCCE | 8 | -2.60 |
| 1346 | 3DK9:A | oxidoreductase | 417 | 423 | CVMKMVC | 7 | 3.79 | EEEEEEE | 15 | 1.90 |
| 1347 | 1YPF:A | oxidoreductase | 181 | 185 | TGFGT | 5 | 67.58 | HSCSS | 7 | 0.12 |
| 1348 | 1Y7T:B | oxidoreductase | 245 | 249 | AANAA | 5 | 2.62 | HHHHH | 9 | 0.74 |
| 1349 | 1JFB:A | oxidoreductase | 102 | 108 | EPTFTPE | 7 | 61.59 | GGGGSHH | 17 | -1.26 |
| 1350 | 1N2F:A | oxidoreductase | 10 | 14 | ATATA | 5 | 14.14 | EEEEE | 11 | 0.80 |
| 1351 | 1N2F:A | oxidoreductase | 9 | 15 | TATATAT | 7 | 28.07 | EEEEEEE | 11 | 0.37 |
| 1352 | 1EP3:B | oxidoreductase | 204 | 208 | KAVAK | 5 | 50.42 | HHHHH | 7 | 0.00 |
| 1353 | 1EP3:B | oxidoreductase | 116 | 120 | IGGGI | 5 | 18.32 | EEEGG | 9 | 1.56 |
| 1354 | 1F5V:B | oxidoreductase | 154 | 158 | GLCLG | 5 | 0.04 | EEEEE | 9 | 1.86 |
| 1355 | 1GVE:B | oxidoreductase | 57 | 61 | LGLGL | 5 | 39.18 | SCCCT | 4 | 2.12 |
| 1356 | 1GWE:A | oxidoreductase | 394 | 398 | DSWSD | 5 | 83.80 | CCCCC | 6 | -1.90 |
| 1357 | 1IX9:A | oxidoreductase | 189 | 194 | WNVVNW | 6 | 24.03 | HHHBCH | 12 | -0.07 |
| 1358 | 1JF8:A | oxidoreductase | 67 | 71 | IDNDI | 5 | 73.24 | CCHHH | 6 | -0.30 |
| 1359 | 1JFB:A | oxidoreductase | 103 | 107 | PTFTP | 5 | 46.02 | GGGSH | 15 | -0.36 |
| 1360 | 1JFB:A | oxidoreductase | 7 | 11 | SFPFS | 5 | 42.80 | BSSCC | 15 | 0.48 |
| 1361 | 1JFB:A | oxidoreductase | 344 | 348 | GFGFG | 5 | 29.90 | TTCCG | 15 | 0.88 |
| 1362 | 1JKV:F | oxidoreductase | 190 | 194 | EELEE | 5 | 58.38 | HHHHH | 10 | -2.04 |
| 1363 | 1JKV:F | oxidoreductase | 57 | 61 | DLLLD | 5 | 0.40 | HHHHH | 10 | 0.88 |
| 1364 | 1JR8:B | oxidoreductase | 32 | 36 | EPTPE | 5 | 94.02 | SCCHH | 6 | -2.18 |
| 1365 | 1K3I:A | oxidoreductase | 76 | 80 | GNQNG | 5 | 46.20 | SCCTT | 7 | -2.26 |
| 1366 | 1K3I:A | oxidoreductase | 254 | 258 | DSSSD | 5 | 71.16 | EGGGT | 7 | -1.88 |
| 1367 | 1K3I:A | oxidoreductase | 531 | 535 | NYLYN | 5 | 43.84 | GGGBC | 7 | -1.16 |
| 1368 | 1K3I:A | oxidoreductase | 288 | 292 | GSWSG | 5 | 56.06 | CCCCS | 7 | -0.66 |
| 1369 | 1K3I:A | oxidoreductase | 218 | 222 | TVTVT | 5 | 61.04 | EEEEC | 7 | 1.26 |
| 1370 | 1KMV:A | oxidoreductase | 89 | 93 | LSRSL | 5 | 50.20 | EESSH | 8 | 0.30 |
| 1371 | 1KQ3:A | oxidoreductase | 2 | 6 | ITTTI | 5 | 56.32 | CBCCC | 8 | 1.38 |
| 1372 | 1L5P:C | oxidoreductase | 46 | 50 | KCICK | 5 | 46.02 | CCEEE | 8 | 0.34 |
| 1373 | 1LQT:A | oxidoreductase | 61 | 65 | KSISK | 5 | 57.00 | GGGHH | 17 | -0.98 |
| 1374 | 1LTZ:A | oxidoreductase | 29 | 33 | LPQPL | 5 | 52.48 | BCCCG | 7 | 0.18 |
| 1375 | 1M56:C | oxidoreductase | 76 | 80 | RLGLR | 5 | 39.16 | HHHHH | 8 | -0.36 |
| 1376 | 1M56:C | oxidoreductase | 190 | 194 | AFGFA | 5 | 50.66 | SSCSS | 5 | 1.76 |
| 1377 | 1M56:C | oxidoreductase | 170 | 174 | LAIAL | 5 | 45.28 | HHHHH | 8 | 3.14 |
| 1378 | 1M56:C | oxidoreductase | 48 | 52 | LVVVL | 5 | 64.98 | HHHHH | 8 | 4.04 |
| 1379 | 1MXR:B | oxidoreductase | 189 | 193 | LKKKL | 5 | 20.18 | HHHHH | 14 | -0.82 |
| 1380 | 1N2F:A | oxidoreductase | 40 | 44 | GGQGG | 5 | 49.64 | TCSCC | 4 | -1.02 |
| 1381 | 1N2F:A | oxidoreductase | 9 | 13 | TATAT | 5 | 26.40 | EEEEE | 9 | 0.30 |
| 1382 | 1N2F:A | oxidoreductase | 9 | 13 | TATAT | 5 | 26.40 | EEEEE | 9 | 0.30 |
| 1383 | 1N2F:A | oxidoreductase | 97 | 101 | LEVEL | 5 | 23.32 | EEEEE | 9 | 0.96 |
| 1384 | 1N4W:A | oxidoreductase | 363 | 367 | APMPA | 5 | 12.38 | ECCCC | 26 | 0.46 |
| 1385 | 1N4W:A | oxidoreductase | 108 | 112 | VGRGV | 5 | 16.28 | EECST | 26 | 0.62 |
| 1386 | 1N4W:A | oxidoreductase | 233 | 237 | LAAAL | 5 | 33.60 | HHHHH | 18 | 2.60 |
| 1387 | 1N62:E | oxidoreductase | 461 | 469 | AEQKAKQEA | 9 | 55.92 | HHHHHHHHH | 8 | -1.82 |
| 1388 | 1N62:E | oxidoreductase | 343 | 347 | DACAD | 5 | 10.06 | CCCCC | 8 | -0.18 |
| 1389 | 1N62:F | oxidoreductase | 114 | 119 | GNAANG | 6 | 19.72 | HHHHSC | 8 | -0.70 |
| 1390 | 1NRG:A | oxidoreductase | 100 | 104 | KGFGK | 5 | 61.06 | CCEET | 4 | -1.16 |
| 1391 | 1O04:G | oxidoreductase | 434 | 438 | KDLDK | 5 | 55.26 | SBHHH | 7 | -2.20 |
| 1392 | 1O04:G | oxidoreductase | 222 | 226 | PGFGP | 5 | 45.10 | CBCTT | 7 | -0.24 |
| 1393 | 1O04:G | oxidoreductase | 65 | 69 | AARAA | 5 | 26.88 | HHHHH | 9 | 0.54 |
| 1394 | 1OIH:D | oxidoreductase | 147 | 151 | LPEPL | 5 | 34.86 | SCHHH | 6 | 0.18 |
| 1395 | 1PA2:A | oxidoreductase | 138 | 142 | IPSPI | 5 | 37.34 | SCCTT | 5 | 1.00 |
| 1396 | 1QAU:A | oxidoreductase | 112 | 116 | TGDGT | 5 | 85.04 | CTTCC | 2 | -1.14 |
| 1397 | 1SAU:A | oxidoreductase | 21 | 26 | DWEEWD | 6 | 66.88 | SGGGCC | 11 | -2.63 |
| 1398 | 1SBY:B | oxidoreductase | 127 | 131 | GGPGG | 5 | 43.00 | TCCCE | 7 | -0.64 |
| 1399 | 1SC6:A | oxidoreductase | 249 | 253 | LADAL | 5 | 27.04 | HHHHH | 9 | 1.54 |
| 1400 | 1SU7:A | oxidoreductase | 87 | 91 | AAGAA | 5 | 16.36 | HHHHH | 10 | 1.36 |
| 1401 | 1SU7:A | oxidoreductase | 253 | 257 | AVNVA | 5 | 0.54 | SEEEE | 11 | 1.70 |
| 1402 | 1T0I:B | oxidoreductase | 54 | 58 | EDDDE | 5 | 81.48 | CCCCC | 4 | -3.50 |
| 1403 | 1T0I:B | oxidoreductase | 76 | 80 | RSWSR | 5 | 50.84 | HHHHH | 4 | -2.30 |
| 1404 | 1T0I:B | oxidoreductase | 125 | 129 | GGHGG | 5 | 41.30 | ETTCC | 7 | -0.96 |
| 1405 | 1US0:A | oxidoreductase | 98 | 102 | DLKLD | 5 | 50.14 | HHTCS | 7 | -0.66 |
| 1406 | 1US0:A | oxidoreductase | 103 | 107 | YLDLY | 5 | 9.48 | CBSEE | 7 | 0.30 |
| 1407 | 1US0:A | oxidoreductase | 30 | 34 | AVKVA | 5 | 15.74 | HHHHH | 9 | 1.62 |
| 1408 | 1VYR:A | oxidoreductase | 229 | 233 | RIGIR | 5 | 15.86 | GEEEE | 29 | -0.08 |
| 1409 | 1W7C:A | oxidoreductase | 186 | 190 | LNKNL | 5 | 55.16 | HHHHH | 16 | -0.66 |
| 1410 | 1W7C:A | oxidoreductase | 456 | 460 | ASYSA | 5 | 60.92 | TTEEE | 11 | 0.14 |
| 1411 | 1W7C:A | oxidoreductase | 536 | 540 | VDLDV | 5 | 1.14 | EEECT | 11 | 1.04 |
| 1412 | 1W9A:A | oxidoreductase | 91 | 95 | PAAAP | 5 | 44.92 | CCCST | 5 | 0.44 |
| 1413 | 1W9M:A | oxidoreductase | 46 | 50 | EKLKE | 5 | 62.78 | HHHHH | 8 | -2.20 |
| 1414 | 1W9M:A | oxidoreductase | 112 | 116 | EPLPE | 5 | 78.84 | CCCCG | 6 | -1.28 |
| 1415 | 1W9M:A | oxidoreductase | 501 | 505 | LLALL | 5 | 11.12 | HHHHH | 8 | 3.40 |
| 1416 | 1WMA:A | oxidoreductase | 160 | 164 | ETITE | 5 | 65.14 | SSCCH | 8 | -0.78 |
| 1417 | 1WUI:L | oxidoreductase | 40 | 44 | EVEVE | 5 | 41.18 | EEEEE | 11 | -0.42 |
| 1418 | 1WUI:L | oxidoreductase | 256 | 260 | AEFEA | 5 | 40.48 | HHHHH | 11 | -0.12 |
| 1419 | 1WUI:S | oxidoreductase | 55 | 59 | AAEAA | 5 | 22.92 | HHHHH | 11 | 0.74 |
| 1420 | 1WUI:S | oxidoreductase | 144 | 148 | AINIA | 5 | 3.28 | CEEEC | 9 | 1.82 |
| 1421 | 1WZD:A | oxidoreductase | 211 | 215 | LGKGL | 5 | 82.16 | HHTTC | 3 | 0.58 |
| 1422 | 1XG5:C | oxidoreductase | 109 | 113 | GSTSG | 5 | 26.32 | CCHHH | 9 | -0.62 |
| 1423 | 1XG5:C | oxidoreductase | 232 | 236 | VAEAV | 5 | 16.86 | HHHHH | 8 | 1.70 |
| 1424 | 1XIY:A | oxidoreductase | 67 | 71 | YEEEY | 5 | 48.70 | HHHTH | 15 | -2.62 |
| 1425 | 1Y7T:B | oxidoreductase | 132 | 136 | ANTNA | 5 | 2.28 | HHHHH | 12 | -0.82 |
| 1426 | 1Y7T:B | oxidoreductase | 242 | 246 | AASAA | 5 | 3.60 | HHHHH | 12 | 1.28 |
| 1427 | 1YD7:A | oxidoreductase | 69 | 73 | IAAAI | 5 | 26.32 | HHHHH | 8 | 2.88 |
| 1428 | 1YFU:A | oxidoreductase | 7 | 11 | PFNFP | 5 | 83.28 | CCCHH | 6 | -0.22 |
| 1429 | 1YPF:A | oxidoreductase | 177 | 181 | TKIKT | 5 | 91.72 | HHHHH | 5 | -0.94 |
| 1430 | 1Z9T:A | oxidoreductase | 219 | 223 | TENET | 5 | 79.08 | TCTTT | 5 | -2.38 |
| 1431 | 1ZJZ:A | oxidoreductase | 16 | 20 | LGIGL | 5 | 37.58 | SHHHH | 5 | 2.26 |
| 1432 | 2AA3:A | oxidoreductase | 317 | 321 | EAVAE | 5 | 38.42 | HHHHH | 8 | 0.16 |
| 1433 | 2AD7:A | oxidoreductase | 545 | 549 | AGLGA | 5 | 17.58 | TGGGH | 6 | 1.32 |
| 1434 | 2BFF:A | oxidoreductase | 268 | 272 | ARRRA | 5 | 44.20 | HHHHH | 10 | -1.98 |
| 1435 | 2BFF:A | oxidoreductase | 227 | 231 | STPTS | 5 | 47.70 | TEEGG | 6 | -0.92 |
| 1436 | 2BFF:A | oxidoreductase | 174 | 178 | AAYAA | 5 | 6.80 | HHHHH | 10 | 1.18 |
| 1437 | 2BLA:A | oxidoreductase | 138 | 142 | KTLTK | 5 | 99.52 | SSCCT | 5 | -1.08 |
| 1438 | 2BLA:A | oxidoreductase | 145 | 149 | VKEKV | 5 | 80.54 | CCSCC | 5 | -0.58 |
| 1439 | 2BS2:F | oxidoreductase | 149 | 153 | TQPQT | 5 | 17.26 | HCGGG | 6 | -2.00 |
| 1440 | 2BS2:F | oxidoreductase | 34 | 38 | GLFLG | 5 | 37.70 | HHHHH | 7 | 1.92 |
| 1441 | 2BS2:F | oxidoreductase | 84 | 88 | AFVFA | 5 | 48.32 | HHHHH | 7 | 2.68 |
| 1442 | 2BW4:A | oxidoreductase | 300 | 304 | VYAYV | 5 | 29.32 | EEEEE | 9 | 1.52 |
| 1443 | 2C29:F | oxidoreductase | 7 | 11 | TVCVT | 5 | 6.06 | EEEES | 13 | 1.90 |
| 1444 | 2D1S:A | oxidoreductase | 474 | 478 | VPDPV | 5 | 89.66 | EEETT | 4 | 0.34 |
| 1445 | 2D37:A | oxidoreductase | 86 | 90 | ERFRE | 5 | 69.08 | GGGGS | 6 | -2.64 |
| 1446 | 2E6F:A | oxidoreductase | 285 | 289 | LEDEL | 5 | 38.40 | HHHHH | 11 | -0.58 |
| 1447 | 2E6F:A | oxidoreductase | 231 | 235 | ANVNA | 5 | 0.50 | HHHHH | 11 | 0.16 |
| 1448 | 2E6F:A | oxidoreductase | 218 | 222 | GGLGG | 5 | 3.18 | EEEES | 8 | 0.44 |
| 1449 | 2ECU:A | oxidoreductase | 103 | 107 | LRCRL | 5 | 37.60 | EEEEE | 7 | 0.22 |
| 1450 | 2ECU:A | oxidoreductase | 45 | 49 | VALAV | 5 | 1.56 | EEEEE | 7 | 3.16 |
| 1451 | 2F5V:A | oxidoreductase | 558 | 562 | DEKED | 5 | 94.98 | CTTTT | 6 | -3.58 |
| 1452 | 2F5V:A | oxidoreductase | 187 | 191 | DADAD | 5 | 55.10 | CHHHH | 6 | -1.38 |
| 1453 | 2F5V:A | oxidoreductase | 293 | 297 | SEIES | 5 | 36.30 | SCEEE | 6 | -0.82 |
| 1454 | 2F5V:A | oxidoreductase | 220 | 224 | NLVLN | 5 | 29.58 | HHHHH | 8 | 0.96 |
| 1455 | 2GHC:X | oxidoreductase | 124 | 128 | EPPPE | 5 | 76.70 | SCCCS | 4 | -2.36 |
| 1456 | 2GHC:X | oxidoreductase | 197 | 201 | GEKEG | 5 | 94.80 | CCCTT | 4 | -2.34 |
| 1457 | 2GHC:X | oxidoreductase | 226 | 230 | ADEDA | 5 | 73.62 | HCHHH | 4 | -1.38 |
| 1458 | 2GHC:X | oxidoreductase | 50 | 54 | GTKTG | 5 | 77.10 | TTTBC | 4 | -1.22 |
| 1459 | 2GHC:X | oxidoreductase | 229 | 234 | DAFFAD | 6 | 44.02 | HHHHHH | 8 | 0.37 |
| 1460 | 2H88:C | oxidoreductase | 76 | 80 | SLSLS | 5 | 72.68 | HTCCC | 8 | 1.04 |
| 1461 | 2I7H:D | oxidoreductase | 102 | 106 | RQIQR | 5 | 73.30 | SHHHH | 7 | -2.30 |
| 1462 | 2IG6:A | oxidoreductase | 24 | 28 | PRVRP | 5 | 21.34 | EEEEE | 9 | -1.60 |
| 1463 | 2IG6:A | oxidoreductase | 87 | 91 | ELALE | 5 | 52.36 | HHHHH | 9 | 0.48 |
| 1464 | 2IJ2:A | oxidoreductase | 290 | 295 | AAEEAA | 6 | 32.30 | HHHHHH | 11 | 0.03 |
| 1465 | 2IUW:A | oxidoreductase | 152 | 156 | PHWHP | 5 | 86.44 | SSCCH | 6 | -2.10 |
| 1466 | 2JAE:A | oxidoreductase | 7 | 11 | GKVKG | 5 | 84.24 | CCCCS | 4 | -0.88 |
| 1467 | 2JHF:A | oxidoreductase | 213 | 217 | AAGAA | 5 | 35.30 | HTTCS | 6 | 1.36 |
| 1468 | 2JHF:A | oxidoreductase | 183 | 187 | AVKVA | 5 | 30.82 | HHTTT | 6 | 1.62 |
| 1469 | 2NWF:A | oxidoreductase | 171 | 175 | LPIPL | 5 | 29.60 | CCBCS | 19 | 1.78 |
| 1470 | 2OX0:B | oxidoreductase | 60 | 64 | DDIDD | 5 | 81.68 | TTCTT | 6 | -1.90 |
| 1471 | 2OX0:B | oxidoreductase | 67 | 71 | IPAPI | 5 | 26.64 | ESSCE | 6 | 1.52 |
| 1472 | 2P25:A | oxidoreductase | 44 | 48 | KLDLK | 5 | 21.64 | EEEEE | 10 | -0.74 |
| 1473 | 2P2S:A | oxidoreductase | 207 | 211 | HHPHH | 5 | 87.88 | SCTTS | 7 | -2.88 |
| 1474 | 2P8I:A | oxidoreductase | 7 | 11 | SAIAS | 5 | 29.72 | GGCCE | 6 | 1.30 |
| 1475 | 2Q3E:A | oxidoreductase | 350 | 354 | SIYIS | 5 | 19.58 | HHHHH | 8 | 1.22 |
| 1476 | 2Q3E:A | oxidoreductase | 281 | 285 | VLNLV | 5 | 6.24 | HHHHH | 8 | 2.50 |
| 1477 | 2QL8:A | oxidoreductase | 114 | 118 | NEIEN | 5 | 65.20 | HHHHH | 8 | -1.90 |
| 1478 | 2R01:A | oxidoreductase | 86 | 90 | GPEPG | 5 | 50.42 | SCCTT | 5 | -1.50 |
| 1479 | 2RAF:C | oxidoreductase | 94 | 98 | LQQQL | 5 | 42.08 | HHHHC | 9 | -0.58 |
| 1480 | 2RAF:C | oxidoreductase | 50 | 55 | ALAALA | 6 | 36.78 | HHHHHH | 8 | 2.47 |
| 1481 | 2VFR:A | oxidoreductase | 80 | 86 | RVGGGVR | 7 | 38.33 | EEETTCB | 9 | -0.26 |
| 1482 | 2VFR:A | oxidoreductase | 240 | 244 | AAPAA | 5 | 54.92 | SEECS | 9 | 1.12 |
| 1483 | 2VFR:A | oxidoreductase | 395 | 399 | GALAG | 5 | 30.58 | HHHHH | 7 | 1.32 |
| 1484 | 2VFR:A | oxidoreductase | 81 | 85 | VGGGV | 5 | 11.16 | EETTC | 9 | 1.44 |
| 1485 | 2VPA:A | oxidoreductase | 100 | 104 | PSNSP | 5 | 36.36 | CCSSG | 8 | -1.66 |
| 1486 | 2VPA:A | oxidoreductase | 99 | 105 | LPSNSPL | 7 | 51.33 | ECCSSGG | 8 | -0.10 |
| 1487 | 2VYN:D | oxidoreductase | 260 | 264 | KAAAK | 5 | 65.34 | HHHHH | 12 | -0.48 |
| 1488 | 2VYN:D | oxidoreductase | 13 | 17 | RLVLR | 5 | 5.26 | HHHHH | 12 | 0.56 |
| 1489 | 2WJ6:D | oxidoreductase | 147 | 151 | ERWRE | 5 | 102.86 | TTHHH | 7 | -3.38 |
| 1490 | 2WJ6:D | oxidoreductase | 179 | 183 | DYGYD | 5 | 50.28 | TCCHH | 7 | -2.00 |
| 1491 | 2WZV:B | oxidoreductase | 73 | 77 | LAEAL | 5 | 22.66 | HHHHH | 9 | 1.54 |
| 1492 | 2X9G:A | oxidoreductase | 231 | 235 | EASAE | 5 | 40.92 | CCCHH | 24 | -0.84 |
| 1493 | 2X9G:A | oxidoreductase | 189 | 193 | ALELA | 5 | 7.74 | HHHHG | 24 | 1.54 |
| 1494 | 2XHF:B | oxidoreductase | 89 | 93 | YLSLY | 5 | 44.02 | HHHTH | 20 | 0.84 |
| 1495 | 2ZDP:A | oxidoreductase | 46 | 50 | EDTDE | 5 | 68.18 | CSSEE | 9 | -2.94 |
| 1496 | 2ZXK:A | oxidoreductase | 99 | 103 | SAEAS | 5 | 15.92 | SEEEE | 7 | -0.30 |
| 1497 | 2ZXK:A | oxidoreductase | 77 | 81 | LQSQL | 5 | 58.72 | TTTTB | 7 | -0.04 |
| 1498 | 3A2V:J | oxidoreductase | 97 | 101 | IPFPI | 5 | 13.66 | CCSCE | 7 | 1.72 |
| 1499 | 3AG3:B | oxidoreductase | 72 | 76 | ILILI | 5 | 46.52 | HHHHH | 13 | 4.22 |
| 1500 | 3AG3:L | oxidoreductase | 29 | 33 | FGSGF | 5 | 16.06 | HHHHH | 7 | 0.80 |
| 1501 | 3ALJ:A | oxidoreductase | 89 | 93 | KSVSK | 5 | 69.70 | EEEEE | 10 | -1.04 |
| 1502 | 3ALJ:A | oxidoreductase | 243 | 247 | EVWVE | 5 | 53.40 | HHHHH | 10 | 0.10 |
| 1503 | 3B1F:A | oxidoreductase | 38 | 42 | DRSRD | 5 | 75.10 | HHHHH | 7 | -3.36 |
| 1504 | 3BAL:D | oxidoreductase | 82 | 86 | GEQEG | 5 | 97.70 | CGGGT | 4 | -2.26 |
| 1505 | 3BB0:A | oxidoreductase | 350 | 354 | WKEKW | 5 | 10.54 | HHHHH | 10 | -2.62 |
| 1506 | 3BB0:A | oxidoreductase | 121 | 125 | PNPNP | 5 | 63.78 | TCCBT | 9 | -2.36 |
| 1507 | 3BB0:A | oxidoreductase | 18 | 22 | YNTNY | 5 | 20.40 | GGGSH | 9 | -2.06 |
| 1508 | 3BB0:A | oxidoreductase | 36 | 40 | VTHTV | 5 | 15.94 | HHHHH | 10 | 0.76 |
| 1509 | 3BB0:A | oxidoreductase | 260 | 264 | AVRVA | 5 | 27.54 | HHHHH | 10 | 1.50 |
| 1510 | 3BEM:A | oxidoreductase | 135 | 139 | ASLSA | 5 | 20.60 | HHHHH | 8 | 1.16 |
| 1511 | 3BNJ:A | oxidoreductase | 428 | 432 | LVKVL | 5 | 44.02 | HHHHH | 9 | 2.42 |
| 1512 | 3BVF:F | oxidoreductase | 97 | 101 | ISESI | 5 | 28.04 | HHHHH | 11 | 0.78 |
| 1513 | 3C24:B | oxidoreductase | 88 | 92 | PRVRP | 5 | 75.18 | GGSCT | 9 | -1.60 |
| 1514 | 3C24:B | oxidoreductase | 39 | 43 | AIEIA | 5 | 49.40 | EECCC | 9 | 1.82 |
| 1515 | 3CB0:A | oxidoreductase | 156 | 160 | AEEEA | 5 | 74.40 | CCSEE | 6 | -1.38 |
| 1516 | 3CX5:I | oxidoreductase | 22 | 26 | FAGAF | 5 | 54.94 | HHHHH | 7 | 1.76 |
| 1517 | 3CXG:B | oxidoreductase | 74 | 78 | VDIDV | 5 | 13.48 | EEEET | 10 | 1.18 |
| 1518 | 3CXK:B | oxidoreductase | 19 | 23 | LRRRL | 5 | 88.04 | HHHHS | 7 | -1.18 |
| 1519 | 3DAS:A | oxidoreductase | 329 | 333 | DGRGD | 5 | 76.18 | SSSSC | 8 | -2.46 |
| 1520 | 3DB2:A | oxidoreductase | 291 | 295 | DVKVD | 5 | 81.58 | EEECC | 5 | -0.50 |
| 1521 | 3DB2:A | oxidoreductase | 170 | 174 | PATAP | 5 | 49.62 | TTTST | 5 | -0.06 |
| 1522 | 3DK9:A | oxidoreductase | 411 | 415 | TKRKT | 5 | 71.58 | CSSCC | 7 | -2.74 |
| 1523 | 3DK9:A | oxidoreductase | 28 | 32 | GGSGG | 5 | 9.22 | CSHHH | 7 | -0.48 |
| 1524 | 3DK9:A | oxidoreductase | 418 | 422 | VMKMV | 5 | 5.10 | EEEEE | 12 | 1.66 |
| 1525 | 3DML:A | oxidoreductase | 59 | 63 | FTPTF | 5 | 28.90 | SSSEE | 8 | 0.52 |
| 1526 | 3EAT:X | oxidoreductase | 132 | 136 | GDSDG | 5 | 66.88 | SCCSB | 6 | -1.72 |
| 1527 | 3EAT:X | oxidoreductase | 147 | 151 | ALQLA | 5 | 43.32 | HHHHC | 6 | 1.54 |
| 1528 | 3EAT:X | oxidoreductase | 88 | 92 | VLELV | 5 | 60.92 | CEEEE | 6 | 2.50 |
| 1529 | 3EGW:C | oxidoreductase | 11 | 15 | IYPYI | 5 | 56.76 | THHHH | 9 | 0.96 |
| 1530 | 3ELN:A | oxidoreductase | 27 | 31 | EVNVE | 5 | 80.80 | SCCHH | 5 | -0.42 |
| 1531 | 3ELN:A | oxidoreductase | 15 | 19 | LIRIL | 5 | 27.36 | HHHHH | 8 | 2.42 |
| 1532 | 3F1L:B | oxidoreductase | 135 | 139 | LLPLL | 5 | 19.78 | HHHHH | 21 | 2.72 |
| 1533 | 3FZ4:A | oxidoreductase | 69 | 73 | LKDKL | 5 | 74.64 | HHHHG | 4 | -0.74 |
| 1534 | 3G14:A | oxidoreductase | 59 | 63 | IANAI | 5 | 30.08 | HHHHB | 7 | 1.82 |
| 1535 | 3GDC:C | oxidoreductase | 29 | 33 | RDFDR | 5 | 74.52 | TCEEC | 11 | -2.64 |
| 1536 | 3GDC:C | oxidoreductase | 176 | 180 | MVMVM | 5 | 0.46 | EEEEE | 9 | 2.82 |
| 1537 | 3GE3:B | oxidoreductase | 246 | 250 | LLPLL | 5 | 19.70 | HHHHH | 7 | 2.72 |
| 1538 | 3GKM:A | oxidoreductase | 155 | 159 | KAHAK | 5 | 97.42 | HHHHC | 7 | -1.48 |
| 1539 | 3GKM:A | oxidoreductase | 64 | 68 | KAGAK | 5 | 67.10 | HTTCE | 7 | -0.92 |
| 1540 | 3GWL:B | oxidoreductase | 177 | 181 | NNVNN | 5 | 43.82 | HHHHH | 9 | -1.96 |
| 1541 | 3HD5:C | oxidoreductase | 154 | 158 | VQTQV | 5 | 42.76 | HHHHH | 10 | 0.14 |
| 1542 | 3HLX:A | oxidoreductase | 224 | 228 | AMTMA | 5 | 8.26 | HHHHH | 7 | 1.34 |
| 1543 | 3I94:A | oxidoreductase | 1232 | 1236 | EAWAE | 5 | 82.82 | HHHHH | 9 | -0.86 |
| 1544 | 3KA8:A | oxidoreductase | 53 | 57 | EHSHE | 5 | 50.80 | HHHHH | 11 | -2.84 |
| 1545 | 3KA8:A | oxidoreductase | 27 | 31 | SYTYS | 5 | 33.20 | HHHHH | 11 | -0.98 |
| 1546 | 3L77:A | oxidoreductase | 146 | 150 | YGGGY | 5 | 36.82 | TCHHH | 9 | -0.76 |
| 1547 | 3L77:A | oxidoreductase | 19 | 23 | RALAR | 5 | 40.92 | HHHHH | 9 | -0.32 |
| 1548 | 3L8W:A | oxidoreductase | 63 | 67 | TIYIT | 5 | 45.72 | HHHHH | 18 | 1.26 |
| 1549 | 3LQK:A | oxidoreductase | 40 | 44 | VTHTV | 5 | 61.26 | CSSCS | 4 | 0.76 |
| 1550 | 3M5Q:A | oxidoreductase | 59 | 63 | AGGGA | 5 | 5.26 | GCCSS | 8 | 0.48 |
| 1551 | 3MM1:A | oxidoreductase | 295 | 299 | DTLTD | 5 | 62.76 | TSTTC | 9 | -0.92 |
| 1552 | 3NFW:A | oxidoreductase | 125 | 130 | HDGGDH | 6 | 32.45 | EEETTE | 7 | -2.37 |
| 1553 | 3NW4:A | oxidoreductase | 85 | 89 | ALGLA | 5 | 32.12 | EEECC | 7 | 2.16 |
| 1554 | 3NYC:A | oxidoreductase | 1009 | 1013 | IGAGI | 5 | 7.92 | ECCSH | 10 | 2.00 |
| 1555 | 3NYC:A | oxidoreductase | 1224 | 1228 | AFIFA | 5 | 9.16 | EEEEC | 10 | 2.74 |
| 1556 | 3NZN:B | oxidoreductase | 36 | 40 | KKTKK | 5 | 81.78 | HHHHH | 8 | -3.26 |
| 1557 | 3O8Q:A | oxidoreductase | 219 | 223 | YGKGY | 5 | 76.40 | CCSSC | 4 | -1.46 |
| 1558 | 3OA2:D | oxidoreductase | 91 | 95 | GLRLG | 5 | 23.78 | HHHTT | 8 | 0.46 |
| 1559 | 3OD3:A | oxidoreductase | 231 | 235 | LRLRL | 5 | 4.42 | EEEEE | 13 | 0.48 |
| 1560 | 3OD3:A | oxidoreductase | 282 | 286 | EVLVE | 5 | 18.14 | EEEEE | 13 | 1.04 |
| 1561 | 3OJ0:A | oxidoreductase | 61 | 65 | YEYEY | 5 | 93.16 | HTCEE | 6 | -2.18 |
| 1562 | 3OJN:D | oxidoreductase | 243 | 247 | RGPGR | 5 | 7.34 | EEEEE | 7 | -2.28 |
| 1563 | 3OJN:D | oxidoreductase | 345 | 349 | GDEDG | 5 | 75.26 | TCSTT | 12 | -2.26 |
| 1564 | 3OJN:D | oxidoreductase | 127 | 131 | EFFFE | 5 | 23.60 | EEECC | 12 | 0.28 |
| 1565 | 3OLJ:A | oxidoreductase | 287 | 291 | ERVRE | 5 | 80.76 | HHHHH | 7 | -2.36 |
| 1566 | 3OLJ:A | oxidoreductase | 236 | 240 | FSGSF | 5 | 4.80 | THHHH | 8 | 0.72 |
| 1567 | 3P7X:A | oxidoreductase | 30 | 34 | LDNDL | 5 | 47.14 | ECTTS | 4 | -0.58 |
| 1568 | 3P7X:A | oxidoreductase | 156 | 160 | AALAA | 5 | 27.38 | HHHHH | 6 | 2.20 |
| 1569 | 3QJG:L | oxidoreductase | 58 | 62 | DEFED | 5 | 96.34 | CTTTC | 12 | -2.24 |
| 1570 | 3QJG:L | oxidoreductase | 15 | 19 | SINIS | 5 | 17.32 | GGGHH | 12 | 0.78 |
| 1571 | 3QZB:A | oxidoreductase | 62 | 66 | PDGDP | 5 | 62.40 | ETTCS | 5 | -2.12 |
| 1572 | 3RF7:A | oxidoreductase | 262 | 266 | YGLGY | 5 | 26.22 | HHHHH | 6 | 0.08 |
| 1573 | 3RF7:A | oxidoreductase | 211 | 215 | FAKAF | 5 | 58.46 | HHHHH | 6 | 1.06 |
| 1574 | 3RHE:A | oxidoreductase | 45 | 49 | GLRLG | 5 | 49.58 | SCEEE | 9 | 0.46 |
| 1575 | 3SF6:A | oxidoreductase | 203 | 207 | PTDTP | 5 | 64.82 | ETTST | 4 | -1.62 |
| 1576 | 3SF6:A | oxidoreductase | 342 | 346 | AIEIA | 5 | 21.32 | HHHHH | 9 | 1.82 |
| 1577 | 3SX2:H | oxidoreductase | 21 | 25 | RGQGR | 5 | 31.16 | SHHHH | 8 | -2.66 |
| 1578 | 3SX2:H | oxidoreductase | 247 | 251 | VANAV | 5 | 4.42 | HHHHH | 9 | 1.70 |
| 1579 | 3TKT:A | oxidoreductase | 176 | 180 | PEDEP | 5 | 81.90 | GGGHH | 7 | -2.74 |
| 1580 | 3TKT:A | oxidoreductase | 214 | 218 | VAGAV | 5 | 25.54 | HHHHH | 8 | 2.32 |
| 1581 | 3TKT:A | oxidoreductase | 282 | 286 | ALALA | 5 | 4.30 | HHHHH | 8 | 2.60 |
| 1582 | 3U52:C | oxidoreductase | 312 | 316 | EARAE | 5 | 53.12 | HHHHH | 11 | -1.58 |
| 1583 | 3U52:C | oxidoreductase | 119 | 123 | LLRLL | 5 | 18.48 | HHHHT | 11 | 2.14 |
| 1584 | 3U7I:D | oxidoreductase | 65 | 69 | QGNGQ | 5 | 85.78 | HHHTC | 5 | -2.26 |
| 1585 | 3U7Q:A | oxidoreductase | 254 | 258 | SGDGS | 5 | 0.90 | ETTCC | 11 | -1.18 |
| 1586 | 3U7Q:A | oxidoreductase | 421 | 425 | IGSGI | 5 | 3.94 | EEECH | 11 | 1.48 |
| 1587 | 3U7Q:D | oxidoreductase | 179 | 183 | FPVPF | 5 | 17.82 | SCCCB | 6 | 1.32 |
| 1588 | 3UUW:D | oxidoreductase | 172 | 176 | ALWLA | 5 | 16.46 | HHHHH | 7 | 2.06 |
| 1589 | 3V0S:A | oxidoreductase | 186 | 190 | IEDEI | 5 | 65.52 | GGTTH | 12 | -0.30 |
| 1590 | 3VMK:B | oxidoreductase | 145 | 149 | GKPKG | 5 | 59.32 | CSCCE | 7 | -2.04 |
| 1591 | 3VMK:B | oxidoreductase | 356 | 360 | IAQAI | 5 | 28.82 | HHHHH | 7 | 1.82 |
| 1592 | 4DGQ:C | oxidoreductase | 58 | 62 | RGHGR | 5 | 16.14 | TTSTT | 10 | -2.60 |
| 1593 | 4DGQ:C | oxidoreductase | 70 | 74 | HDMDH | 5 | 40.02 | CSHHH | 10 | -2.30 |
| 1594 | 1DCS:A | oxidoreductase | 131 | 135 | RAVAR | 5 | 28.98 | HHHHH | 9 | -0.24 |
| 1595 | 1KQ3:A | oxidoreductase | 21 | 25 | LEEEL | 5 | 26.52 | HHHHH | 8 | -0.58 |
| 1596 | 1N62:E | oxidoreductase | 463 | 467 | QKAKQ | 5 | 52.98 | HHHHH | 8 | -2.60 |
| 1597 | 1N62:F | oxidoreductase | 281 | 285 | RAKAR | 5 | 65.50 | HHHHH | 12 | -1.86 |
| 1598 | 1PA2:A | oxidoreductase | 273 | 277 | QAFAQ | 5 | 50.32 | HHHHH | 8 | -0.12 |
| 1599 | 1ST9:A | oxidoreductase | 151 | 155 | VVKVV | 5 | 52.16 | EEEEE | 6 | 2.58 |
| 1600 | 1VL7:A | oxidoreductase | 66 | 70 | LVNVL | 5 | 10.86 | EEEEE | 10 | 2.50 |
| 1601 | 3AG3:B | oxidoreductase | 210 | 214 | VLELV | 5 | 0.04 | EEEEE | 13 | 2.50 |
| 1602 | 3CXK:B | oxidoreductase | 99 | 103 | RVEVR | 5 | 28.60 | EEEEE | 13 | -0.82 |
| 1603 | 3GIY:A | oxidoreductase | 152 | 156 | TLVLT | 5 | 4.64 | EEEEE | 9 | 2.08 |
| 1604 | 3HA2:A | oxidoreductase | 159 | 164 | AYQQYA | 6 | 37.12 | HHHHHH | 12 | -1.00 |
| 1605 | 3PXL:A | oxidoreductase | 379 | 383 | SIEIS | 5 | 9.14 | EEEEE | 24 | 0.78 |
| 1606 | 3QY9:D | oxidoreductase | 203 | 207 | TIQIT | 5 | 12.28 | EEEEE | 7 | 0.82 |
| 1607 | 3QY9:D | oxidoreductase | 26 | 30 | IVGVI | 5 | 7.34 | EEEEE | 7 | 3.40 |
| 1608 | 3SX2:H | oxidoreductase | 20 | 26 | ARGQGRA | 7 | 23.33 | CCCCCCC | 9 | -1.39 |
| 1609 | 3CXG:B | oxidoreductase | 76 | 80 | IDVDI | 5 | 48.40 | EETTT | 5 | 1.24 |
| 1610 | 2F5V:A | oxidoreductase | 186 | 192 | DDADADD | 7 | 68.37 | SCHHHHH | 8 | -1.99 |
| 1611 | 2BS2:F | oxidoreductase | 36 | 40 | FLGLF | 5 | 54.58 | HHHHH | 7 | 2.56 |
| 1612 | 3KT7:A | oxidoreductase | 74 | 78 | IETEI | 5 | 53.76 | HHHHC | 6 | 0.26 |
| 1613 | 3KT7:A | oxidoreductase | 537 | 541 | TLCLT | 5 | 1.96 | EEEEC | 6 | 1.74 |
| 1614 | 2VFR:A | oxidoreductase | 397 | 401 | LAGAL | 5 | 32.06 | HHHHH | 5 | 2.16 |
| 1615 | 1JR7:A | oxidoreductase, lyase | 275 | 279 | VPVPV | 5 | 31.74 | ECCCT | 5 | 1.88 |
| 1616 | 2XTS:D | oxidoreductase/electron transport | 113 | 117 | VYDYV | 5 | 18.14 | HHHHH | 8 | 0.46 |
| 1617 | 4AJJ:A | oxidoreductase/inhibitor | 115 | 119 | IFKFI | 5 | 57.00 | HHHHH | 8 | 2.14 |
| 1618 | 3AWU:B | oxidoreductase/metal transport | 107 | 111 | AARAA | 5 | 23.88 | HHHHH | 9 | 0.54 |
| 1619 | 3AWU:A | oxidoreductase/metal transport | 44 | 48 | SDTDS | 5 | 37.06 | HCCTT | 6 | -1.86 |
| 1620 | 3AWU:A | oxidoreductase/metal transport | 237 | 242 | PTGGTP | 6 | 61.38 | CCSCCT | 6 | -0.90 |
| 1621 | 3AWU:A | oxidoreductase/metal transport | 8 | 12 | ATLTA | 5 | 60.64 | GGCCH | 6 | 1.20 |
| 1622 | 3AWU:B | oxidoreductase/metal transport | 105 | 109 | RAAAR | 5 | 42.72 | HHHHH | 7 | -0.72 |
| 1623 | 3PWK:A | oxidoreductase/oxidoreductase inhibitor | 160 | 164 | AGMGA | 5 | 36.84 | GCHHH | 6 | 0.94 |
| 1624 | 3PWK:A | oxidoreductase/oxidoreductase inhibitor | 271 | 275 | AAIAA | 5 | 40.90 | HHHHH | 5 | 2.34 |
| 1625 | 3UXJ:D | oxidoreductase/oxidoreductase substrate | 185 | 189 | LHSHL | 5 | 14.76 | EEEEE | 10 | 0.08 |
| 1626 | 3QQQ:B | oxygen binding, metal binding protein | 65 | 69 | PKLKP | 5 | 74.96 | GGGHH | 6 | -1.44 |
| 1627 | 3QZX:A | oxygen binding, oxygen transport | 8 | 12 | GYTYG | 5 | 37.92 | TCCTT | 7 | -0.82 |
| 1628 | 1DM1:A | oxygen storage/transport | 19 | 23 | ANKNA | 5 | 76.44 | HTHHH | 6 | -1.46 |
| 1629 | 1DM1:A | oxygen storage/transport | 96 | 100 | VGFGV | 5 | 64.54 | HHTTC | 6 | 2.08 |
| 1630 | 1Q1F:A | oxygen storage/transport | 128 | 132 | ATRTA | 5 | 44.54 | HHHHH | 6 | -0.46 |
| 1631 | 1X46:A | oxygen storage/transport | 68 | 72 | IVSVI | 5 | 38.84 | HHHHH | 8 | 3.32 |
| 1632 | 2GKM:B | oxygen storage/transport | 110 | 114 | ETITE | 5 | 66.50 | HHHHH | 8 | -0.78 |
| 1633 | 2GKM:B | oxygen storage/transport | 26 | 30 | EVVVE | 5 | 53.96 | HHHHH | 8 | 1.12 |
| 1634 | 2GKM:B | oxygen storage/transport | 98 | 102 | LADAL | 5 | 21.50 | HHHHH | 8 | 1.54 |
| 1635 | 2BK9:A | oxygen transport | 56 | 60 | ARFRA | 5 | 78.32 | HHHHH | 15 | -0.52 |
| 1636 | 2HBG:A | oxygen transport | 108 | 113 | ASLLSA | 6 | 30.85 | HHHHHH | 8 | 1.60 |
| 1637 | 2W72:A | oxygen transport | 56 | 60 | KGQGK | 5 | 70.16 | HHHHH | 9 | -2.42 |
| 1638 | 2W72:A | oxygen transport | 18 | 22 | GAHAG | 5 | 49.10 | GGGHH | 8 | -0.08 |
| 1639 | 2WTG:A | oxygen transport | 34 | 38 | FYRYF | 5 | 25.86 | HHHHH | 10 | -0.30 |
| 1640 | 1USC:B | oxygenase | 87 | 91 | GSHSG | 5 | 46.26 | TSSCT | 3 | -1.12 |
| 1641 | 1USC:B | oxygenase | 8 | 12 | GPLPG | 5 | 47.20 | SSCSC | 3 | -0.04 |
| 1642 | 1USC:B | oxygenase | 70 | 74 | FSASF | 5 | 0.18 | EEEEE | 9 | 1.16 |
| 1643 | 3DRF:A | peptide binding protein | 212 | 218 | PKTTTKP | 7 | 48.79 | GGGTTSC | 6 | -1.87 |
| 1644 | 3DRF:A | peptide binding protein | 213 | 217 | KTTTK | 5 | 53.22 | GGTTS | 7 | -1.98 |
| 1645 | 3PLU:B | peptide binding protein | 13 | 17 | KVRVK | 5 | 55.54 | EEEEE | 10 | -0.78 |
| 1646 | 3CZ1:A | pheromone binding protein | 47 | 51 | CYMYC | 5 | 13.68 | HHHHH | 11 | 0.86 |
| 1647 | 1H8P:A | phosphorylcholine-binding protein | 88 | 92 | SMWMS | 5 | 86.92 | CSSSC | 7 | 0.26 |
| 1648 | 1H8P:A | phosphorylcholine-binding protein | 25 | 29 | VFPFV | 5 | 46.16 | CSSEE | 7 | 2.48 |
| 1649 | 1OPD:A | phosphotransferase | 51 | 57 | QTLGLTQ | 7 | 62.09 | HHTCCCT | 7 | -0.17 |
| 1650 | 1OPD:A | phosphotransferase | 52 | 56 | TLGLT | 5 | 47.86 | HTCCC | 6 | 1.16 |
| 1651 | 1B33:O | photosynthesis | 14 | 18 | TRIRT | 5 | 66.44 | SSCCC | 5 | -1.18 |
| 1652 | 1JB0:E | photosynthesis | 50 | 54 | GSASG | 5 | 25.50 | TCCCC | 4 | -0.12 |
| 1653 | 1XG0:B | photosynthesis | 28 | 32 | GAKAG | 5 | 72.84 | SCCCS | 5 | -0.22 |
| 1654 | 1XG0:C | photosynthesis | 124 | 128 | ANSNA | 5 | 40.78 | HHHHH | 5 | -0.84 |
| 1655 | 3EOJ:A | photosynthesis | 153 | 158 | LDNNDL | 6 | 67.33 | CCSHHH | 4 | -1.07 |
| 1656 | 3EOJ:A | photosynthesis | 205 | 210 | VNSSNV | 6 | 66.58 | EEEEEE | 6 | -0.03 |
| 1657 | 3EOJ:A | photosynthesis | 133 | 137 | IPNPI | 5 | 72.80 | CSSCC | 4 | 0.46 |
| 1658 | 3IIS:M | photosynthesis | 71 | 75 | GSVSG | 5 | 61.72 | HTCBT | 8 | 0.36 |
| 1659 | 3EOJ:A | photosynthesis | 108 | 112 | FSHSF | 5 | 48.08 | EEEEE | 6 | 0.16 |
| 1660 | 1B33:O | photosynthesis | 13 | 19 | QTRIRTQ | 7 | 72.97 | CSSCCCS | 5 | -1.84 |
| 1661 | 2DS2:D | plant protein | 20 | 24 | PCVCP | 5 | 35.10 | GGHHH | 8 | 1.20 |
| 1662 | 2EA7:A | plant protein | 310 | 314 | QQKQQ | 5 | 118.44 | CCTTS | 8 | -3.58 |
| 1663 | 2EA7:A | plant protein | 210 | 214 | EQIQE | 5 | 69.18 | HHHHH | 11 | -1.90 |
| 1664 | 2EA7:A | plant protein | 174 | 178 | FDSDF | 5 | 33.18 | HTSCH | 8 | -0.44 |
| 1665 | 2EA7:A | plant protein | 93 | 97 | VLTLV | 5 | 4.42 | EEEEE | 11 | 3.06 |
| 1666 | 2FLH:B | plant protein | 135 | 139 | TSQST | 5 | 37.92 | HHHHH | 9 | -1.30 |
| 1667 | 2VU6:A | plant protein | 136 | 140 | AKLKA | 5 | 73.44 | GGGBC | 6 | -0.08 |
| 1668 | 2VU6:A | plant protein | 135 | 141 | PAKLKAP | 7 | 77.91 | CGGGBCT | 5 | -0.51 |
| 1669 | 2Q37:A | plant protein, lyase | 83 | 87 | TAFAT | 5 | 57.92 | HHHTS | 5 | 1.00 |
| 1670 | 1BI5:A | polyketide synthase | 316 | 320 | KLALK | 5 | 68.24 | HHTCC | 8 | 0.32 |
| 1671 | 3N1F:C | protein binding | 870 | 876 | DSDNDSD | 7 | 56.61 | TCCCGGG | 10 | -2.73 |
| 1672 | 3CZ6:A | protein binding | 777 | 783 | VNPPPNV | 7 | 51.27 | CSSCTTC | 6 | -0.49 |
| 1673 | 1KGD:A | protein binding | 789 | 794 | DQMMQD | 6 | 69.18 | HHHHHH | 6 | -1.70 |
| 1674 | 1K52:A | protein binding | 33 | 37 | AYAYA | 5 | 35.84 | HHHHH | 8 | 0.56 |
| 1675 | 1KGD:A | protein binding | 855 | 859 | PTITP | 5 | 75.68 | CSCCT | 7 | -0.02 |
| 1676 | 1KGD:A | protein binding | 826 | 830 | LIAIL | 5 | 6.20 | CEEEE | 7 | 3.68 |
| 1677 | 1OJH:L | protein binding | 6 | 10 | ELSLE | 5 | 88.82 | CCCHH | 5 | -0.04 |
| 1678 | 1R9L:A | protein binding | 58 | 62 | ATFTA | 5 | 3.38 | SCEEE | 11 | 1.00 |
| 1679 | 2IC2:B | protein binding | 467 | 471 | PPTPP | 5 | 34.82 | CCCCC | 4 | -1.42 |
| 1680 | 2OCT:A | protein binding | 79 | 83 | SLTLS | 5 | 52.68 | CEEEE | 6 | 1.06 |
| 1681 | 2OZF:A | protein binding | 150 | 154 | LRPRL | 5 | 110.98 | SSCEE | 4 | -0.60 |
| 1682 | 2Q8V:A | protein binding | 14 | 18 | FEQEF | 5 | 94.50 | SSHHH | 6 | -0.98 |
| 1683 | 2XFA:B | protein binding | 25 | 30 | KYIIYK | 6 | 18.03 | SEEEEE | 14 | -0.23 |
| 1684 | 2XUS:B | protein binding | 74 | 78 | LKEKL | 5 | 83.52 | HHHHH | 9 | -0.74 |
| 1685 | 2XUS:B | protein binding | 86 | 90 | LRLRL | 5 | 114.32 | HHHTC | 5 | 0.48 |
| 1686 | 3AA0:A | protein binding | 215 | 219 | SVQVS | 5 | 60.14 | EEECC | 5 | 0.66 |
| 1687 | 3CZ6:A | protein binding | 778 | 782 | NPPPN | 5 | 60.18 | SSCTT | 7 | -2.36 |
| 1688 | 3CZ6:A | protein binding | 703 | 707 | FLPLF | 5 | 41.54 | BCGGG | 7 | 2.32 |
| 1689 | 3FY5:B | protein binding | 280 | 284 | GIYIG | 5 | 23.26 | CEEEE | 8 | 1.38 |
| 1690 | 3GWR:A | protein binding | 99 | 103 | APRPA | 5 | 70.66 | SCCCC | 5 | -0.82 |
| 1691 | 3GWR:A | protein binding | 52 | 56 | AAVAA | 5 | 38.84 | HHHHH | 12 | 2.28 |
| 1692 | 3GWR:A | protein binding | 84 | 88 | VIRIV | 5 | 8.24 | EEEEE | 12 | 2.58 |
| 1693 | 3K0X:A | protein binding | 34 | 38 | ILRLI | 5 | 39.50 | EEEEE | 10 | 2.42 |
| 1694 | 3K7C:D | protein binding | 90 | 94 | NNDNN | 5 | 54.98 | TTSCE | 9 | -3.50 |
| 1695 | 3KAE:A | protein binding | 191 | 195 | LSPSL | 5 | 50.82 | CCHHH | 7 | 0.88 |
| 1696 | 3KAE:A | protein binding | 144 | 148 | SFLFS | 5 | 23.84 | CCCHH | 7 | 1.56 |
| 1697 | 3LLP:A | protein binding | 165 | 169 | VDRDV | 5 | 51.60 | EEESS | 11 | -0.62 |
| 1698 | 3MSX:B | protein binding | 410 | 414 | LKEKL | 5 | 55.94 | HHHHH | 8 | -0.74 |
| 1699 | 3MSX:B | protein binding | 510 | 514 | AVCVA | 5 | 2.38 | HHHHH | 8 | 2.90 |
| 1700 | 3N1F:C | protein binding | 871 | 875 | SDNDS | 5 | 53.58 | CCCGG | 11 | -2.42 |
| 1701 | 3OK8:A | protein binding | 203 | 207 | GRARG | 5 | 55.72 | HHHHH | 10 | -1.60 |
| 1702 | 3OK8:A | protein binding | 122 | 126 | YEIEY | 5 | 47.86 | HHHHH | 10 | -1.02 |
| 1703 | 3OK8:A | protein binding | 163 | 167 | AQMQA | 5 | 58.76 | HHHHH | 10 | -0.30 |
| 1704 | 3QN1:B | protein binding | 299 | 303 | GEIEG | 5 | 21.02 | HHHHT | 9 | -0.66 |
| 1705 | 3QOU:A | protein binding | 203 | 207 | QQLQQ | 5 | 78.08 | HHHHH | 10 | -2.04 |
| 1706 | 3QOU:A | protein binding | 221 | 225 | QLALQ | 5 | 48.02 | HHHHH | 10 | 0.48 |
| 1707 | 3U23:A | protein binding | 139 | 143 | EEVEE | 5 | 76.46 | EEEET | 6 | -1.96 |
| 1708 | 4A4Y:A | protein binding | 60 | 64 | DSKSD | 5 | 82.82 | TCSSC | 3 | -2.50 |
| 1709 | 3QOR:B | protein binding, cell cycle | 225 | 229 | EVKVE | 5 | 66.06 | CBCGG | 6 | -0.50 |
| 1710 | 3QA9:A | protein binding, de novo protein | 132 | 136 | REHER | 5 | 62.34 | HHHHH | 7 | -3.84 |
| 1711 | 3HTM:D | protein binding, ligase | 246 | 250 | VEPEV | 5 | 58.48 | SCHHH | 5 | -0.04 |
| 1712 | 1TXU:A | protein transport | 372 | 378 | QSLNLSQ | 7 | 65.10 | GGGTCCH | 8 | -0.64 |
| 1713 | 1TXU:A | protein transport | 373 | 377 | SLNLS | 5 | 61.94 | GGTCC | 8 | 0.50 |
| 1714 | 1ZB1:B | protein transport | 134 | 138 | ENINE | 5 | 85.70 | HTCSS | 8 | -1.90 |
| 1715 | 2C5K:T | protein transport | 65 | 69 | DENED | 5 | 117.44 | STTCC | 5 | -3.50 |
| 1716 | 2CW9:A | protein transport | 406 | 411 | GEVVEG | 6 | 75.08 | SCEEEE | 5 | 0.10 |
| 1717 | 2QTV:D | protein transport | 938 | 942 | AVSVA | 5 | 57.54 | CCCCC | 5 | 2.24 |
| 1718 | 2X49:A | protein transport | 608 | 612 | TSGST | 5 | 62.48 | CSSCE | 13 | -0.68 |
| 1719 | 2X49:A | protein transport | 540 | 544 | RVSVR | 5 | 32.40 | TCCCC | 13 | -0.28 |
| 1720 | 3CI0:I | protein transport | 73 | 77 | RWYWR | 5 | 51.46 | EEEEE | 10 | -2.42 |
| 1721 | 3HPC:X | protein transport | 110 | 114 | GEGEG | 5 | 74.62 | HHTGG | 5 | -1.64 |
| 1722 | 3IQ2:A | protein transport | 80 | 84 | LKGKL | 5 | 27.44 | HHHHH | 8 | -0.12 |
| 1723 | 3M1I:B | protein transport | 80 | 84 | EEDEE | 5 | 66.80 | CTTEE | 6 | -3.50 |
| 1724 | 3M1I:B | protein transport | 165 | 169 | AEGEA | 5 | 77.60 | TTSSC | 6 | -0.76 |
| 1725 | 3M1I:B | protein transport | 73 | 77 | KVDVK | 5 | 106.76 | CCCCC | 3 | -0.58 |
| 1726 | 3MNM:A | protein transport | 550 | 554 | NAPAN | 5 | 67.46 | SCCCC | 3 | -1.00 |
| 1727 | 3MNM:A | protein transport | 561 | 565 | VKWKV | 5 | 44.00 | EEEEE | 7 | -0.06 |
| 1728 | 3MNM:A | protein transport | 483 | 487 | IEFEI | 5 | 25.42 | EEEEE | 7 | 0.96 |
| 1729 | 3OV5:A | protein transport | 117 | 121 | VSVSV | 5 | 21.96 | EEEEE | 6 | 2.20 |
| 1730 | 4A56:A | protein transport | 94 | 98 | QQLQQ | 5 | 86.70 | HHHHH | 7 | -2.04 |
| 1731 | 2FAU:A | protein transport | 225 | 229 | TETET | 5 | 79.54 | EEEEE | 5 | -1.82 |
| 1732 | 3M1I:B | protein transport | 171 | 175 | AFTFA | 5 | 15.76 | EEEEE | 3 | 1.70 |
| 1733 | 3OV5:A | protein transport | 118 | 122 | SVSVS | 5 | 31.30 | EEEEE | 7 | 1.20 |
| 1734 | 3GGY:A | protein transport, endocytosis | 67 | 72 | IELLEI | 6 | 32.22 | HHHHHH | 9 | 1.60 |
| 1735 | 3KEP:B | protein transport, rna binding protein | 558 | 562 | KKTKK | 5 | 90.88 | TTTCC | 3 | -3.26 |
| 1736 | 1R8S:A | protein transport/exchange factor | 107 | 111 | LMRML | 5 | 34.48 | HHHHH | 8 | 1.38 |
| 1737 | 3TIW:A | protein transport/ligase | 92 | 96 | LRVRL | 5 | 72.10 | TTCCT | 8 | 0.56 |
| 1738 | 3TIW:A | protein transport/ligase | 85 | 90 | NRVVRN | 6 | 45.95 | CHHHHH | 8 | -1.27 |
| 1739 | 2VLQ:B | protein-binding | 126 | 132 | RHIDIHR | 7 | 80.11 | CCCCCCC | 6 | -1.41 |
| 1740 | 2VLQ:B | protein-binding | 127 | 131 | HIDIH | 5 | 62.32 | HHHHH | 7 | -0.18 |
| 1741 | 2VWR:A | protein-binding | 404 | 409 | AQIIQA | 6 | 73.90 | HHHHHH | 5 | 0.93 |
| 1742 | 2BKR:A | protein-binding/hydrolase | 77 | 81 | PLDLP | 5 | 47.48 | GGTGG | 5 | 0.18 |
| 1743 | 2BKR:A | protein-binding/hydrolase | 186 | 190 | LLQLL | 5 | 55.06 | HHHHC | 5 | 2.34 |
| 1744 | 2BKR:A | protein-binding/hydrolase | 45 | 50 | HDSSDH | 6 | 80.15 | GGGTTT | 5 | -2.50 |
| 1745 | 1A1X:A | proto-oncogene | 101 | 105 | LLKLL | 5 | 33.28 | EEEEC | 10 | 2.26 |
| 1746 | 1BEC:A | receptor | 27 | 31 | NNHNN | 5 | 64.38 | SCCSE | 8 | -3.44 |
| 1747 | 1GP0:A | receptor | 1596 | 1600 | SPCPS | 5 | 58.60 | SBCSS | 8 | -0.46 |
| 1748 | 1GP0:A | receptor | 1587 | 1591 | VLQLV | 5 | 25.04 | EEEEE | 9 | 2.50 |
| 1749 | 2UWI:B | receptor | 34 | 39 | LKCCKL | 6 | 60.98 | TEEEEC | 13 | 0.80 |
| 1750 | 3BVP:B | recombination | 43 | 47 | DTYTD | 5 | 33.96 | EEEEE | 10 | -1.94 |
| 1751 | 1U5K:A | recombination,replication | 180 | 184 | PPYPP | 5 | 71.56 | CCCCH | 4 | -1.54 |
| 1752 | 1U5K:A | recombination,replication | 46 | 50 | LSSSL | 5 | 30.34 | TTTTS | 4 | 1.04 |
| 1753 | 3HTK:C | recombination/replication/ligase | 52 | 56 | TSPST | 5 | 59.34 | CCCCS | 4 | -0.92 |
| 1754 | 2V6V:B | regulator | 406 | 410 | REFER | 5 | 111.20 | EEECC | 7 | -2.64 |
| 1755 | 2V6V:B | regulator | 324 | 328 | LQVQL | 5 | 44.22 | HHHHH | 10 | 0.96 |
| 1756 | 3KDF:C | replication | 74 | 78 | TAKAT | 5 | 69.36 | CTTSC | 4 | -0.34 |
| 1757 | 3JSY:A | ribosomal protein | 33 | 38 | VDMMDV | 6 | 45.70 | EECCSC | 5 | 0.87 |
| 1758 | 3JSY:A | ribosomal protein | 157 | 161 | KIAIK | 5 | 38.66 | EEEEC | 10 | 0.60 |
| 1759 | 3JSY:A | ribosomal protein | 164 | 168 | KVVVK | 5 | 57.60 | EEEEC | 10 | 0.96 |
| 1760 | 3OII:A | ribosomal protein | 96 | 100 | LLTLL | 5 | 6.16 | HHHHH | 9 | 2.90 |
| 1761 | 3ZZP:A | ribosomal protein | 52 | 56 | LNPNL | 5 | 47.22 | ECTTC | 5 | -0.20 |
| 1762 | 1J3A:A | ribosome | 8 | 12 | GLILG | 5 | 16.88 | TBBHH | 10 | 2.26 |
| 1763 | 1OA8:A | rna binding | 564 | 568 | PAAAP | 5 | 25.24 | CEECC | 5 | 0.44 |
| 1764 | 1OA8:A | rna binding | 644 | 648 | EVLVE | 5 | 28.62 | EEETT | 5 | 1.04 |
| 1765 | 3LRU:A | rna binding protein | 1935 | 1941 | RLILILR | 7 | 28.53 | CCCCCCC | 9 | 1.63 |
| 1766 | 1B34:A | rna binding protein | 27 | 31 | GTITG | 5 | 24.96 | EEEEE | 10 | 0.46 |
| 1767 | 1D3B:L | rna binding protein | 66 | 70 | VLGLV | 5 | 31.90 | EEEEE | 5 | 3.12 |
| 1768 | 1IQ4:B | rna binding protein | 169 | 173 | LLALL | 5 | 20.96 | HHHHH | 7 | 3.40 |
| 1769 | 2BTI:A | rna binding protein | 18 | 22 | VTVTV | 5 | 20.08 | EEEEE | 9 | 2.24 |
| 1770 | 2Z0T:A | rna binding protein | 48 | 52 | KVKVK | 5 | 44.80 | EEEEE | 8 | -0.66 |
| 1771 | 3LRU:A | rna binding protein | 1936 | 1940 | LILIL | 5 | 11.60 | HHHHH | 8 | 4.08 |
| 1772 | 3S6E:A | rna binding protein | 481 | 485 | IAAAI | 5 | 46.06 | HHHHH | 6 | 2.88 |
| 1773 | 3DA5:A | rna binding protein | 62 | 66 | DNSND | 5 | 81.14 | SCHHH | 5 | -2.96 |
| 1774 | 3ETI:A | rna binding protein | 143 | 147 | KAIAK | 5 | 62.64 | HHHHT | 5 | 0.06 |
| 1775 | 3GVO:A | rna binding protein | 714 | 719 | FRNNRF | 6 | 93.52 | HHTTCC | 8 | -1.73 |
| 1776 | 3TYT:A | rna binding protein | 547 | 551 | DSKSD | 5 | 63.04 | SSHHH | 8 | -2.50 |
| 1777 | 3S6E:A | rna binding protein | 483 | 487 | AAIAA | 5 | 21.74 | HHHHH | 8 | 2.34 |
| 1778 | 3NV0:A | rna binding/protein transport | 359 | 363 | FTRTF | 5 | 0.24 | EEEEE | 11 | -0.06 |
| 1779 | 2C5S:A | rna-binding protein | 266 | 270 | RMMMR | 5 | 24.44 | HHHHH | 13 | -0.66 |
| 1780 | 2WBM:A | rna-binding protein | 157 | 161 | KIPIK | 5 | 54.96 | TSCCE | 7 | -0.08 |
| 1781 | 1GCI:A | serine protease | 128 | 132 | SPSPS | 5 | 66.22 | BSSCC | 10 | -1.12 |
| 1782 | 1GCI:A | serine protease | 99 | 103 | SGSGS | 5 | 63.34 | TSCBC | 10 | -0.64 |
| 1783 | 1GCI:A | serine protease | 156 | 164 | SGAGS | 5 | 51.72 | SCCSS | 10 | -0.12 |
| 1784 | 1EAI:D | serine proteinase | 36 | 42 | PSCECSP | 7 | 22.07 | CEEECCG | 14 | -0.47 |
| 1785 | 1EAI:D | serine proteinase | 37 | 41 | SCECS | 5 | 15.26 | EEECC | 13 | -0.02 |
| 1786 | 1MAI:A | signal transduction protein | 112 | 116 | APSPA | 5 | 46.82 | CSSHH | 6 | -0.08 |
| 1787 | 2NTX:A | signaling protein | 162 | 168 | PPVKVPP | 7 | 51.16 | CCEECCT | 5 | -0.27 |
| 1788 | 1F35:B | signaling protein | 1017 | 1021 | LDQDL | 5 | 60.34 | ECHHH | 6 | -0.58 |
| 1789 | 1FC3:C | signaling protein | 140 | 144 | NKPKN | 5 | 94.48 | CHHHH | 4 | -3.28 |
| 1790 | 1FGY:A | signaling protein | 366 | 370 | EEKEE | 5 | 80.06 | HHHHH | 5 | -3.58 |
| 1791 | 1FQJ:C | signaling protein | 75 | 79 | HLELH | 5 | 78.34 | TSCHH | 5 | -0.46 |
| 1792 | 1G4Y:B | signaling protein | 422 | 426 | NAAAN | 5 | 46.24 | HHHHH | 8 | -0.32 |
| 1793 | 1I4D:A | signaling protein | 70 | 74 | DAFAD | 5 | 32.48 | HHHHH | 6 | -0.12 |
| 1794 | 1I4D:A | signaling protein | 28 | 32 | LELEL | 5 | 84.40 | HHHHH | 6 | 0.88 |
| 1795 | 1JUQ:D | signaling protein | 110 | 114 | VKTKV | 5 | 30.48 | HHHHH | 8 | -0.02 |
| 1796 | 1K66:B | signaling protein | 13 | 17 | EDSDE | 5 | 71.62 | CCCHH | 8 | -2.96 |
| 1797 | 1K66:B | signaling protein | 60 | 64 | APRPA | 5 | 26.30 | SCCCS | 8 | -0.82 |
| 1798 | 1T0H:A | signaling protein | 42 | 46 | REAER | 5 | 93.74 | THHHH | 5 | -2.84 |
| 1799 | 1T0H:A | signaling protein | 47 | 51 | QAQAQ | 5 | 43.94 | HHHHH | 9 | -1.38 |
| 1800 | 1T0H:A | signaling protein | 60 | 64 | VAFAV | 5 | 30.80 | CCEEE | 5 | 2.96 |
| 1801 | 1T0H:B | signaling protein | 249 | 253 | LFDFL | 5 | 21.82 | HHHHH | 8 | 1.94 |
| 1802 | 1V9Y:B | signaling protein | 98 | 102 | ELQLE | 5 | 60.50 | EEEEE | 7 | -0.58 |
| 1803 | 1WKO:A | signaling protein | 129 | 133 | RQKQR | 5 | 112.64 | ECCTT | 6 | -3.98 |
| 1804 | 1WKO:A | signaling protein | 49 | 53 | SSVSS | 5 | 54.74 | GGGSS | 6 | 0.20 |
| 1805 | 1XOD:A | signaling protein | 12 | 16 | ARVRA | 5 | 59.32 | EEEEE | 7 | -0.24 |
| 1806 | 1XOD:A | signaling protein | 38 | 42 | SVTVS | 5 | 14.74 | EEEEE | 7 | 1.22 |
| 1807 | 1YFQ:A | signaling protein | 235 | 239 | RFAFR | 5 | 66.76 | CEEEE | 6 | -0.32 |
| 1808 | 1ZCB:A | signaling protein | 74 | 78 | QDFDQ | 5 | 85.22 | CCCCH | 6 | -2.24 |
| 1809 | 2BBA:A | signaling protein | 158 | 162 | KVNVK | 5 | 69.68 | CCEEE | 6 | -0.58 |
| 1810 | 2FPE:A | signaling protein | 17 | 21 | ELELE | 5 | 36.72 | BCCBC | 10 | -0.58 |
| 1811 | 2GZV:A | signaling protein | 34 | 38 | GISIG | 5 | 24.56 | SEEEE | 6 | 1.48 |
| 1812 | 2NTX:A | signaling protein | 163 | 167 | PVKVP | 5 | 36.58 | CEECC | 6 | 0.26 |
| 1813 | 2OSA:A | signaling protein | 350 | 354 | LPIPL | 5 | 25.66 | CSSCS | 5 | 1.78 |
| 1814 | 2QMQ:A | signaling protein | 142 | 146 | GVGVG | 5 | 0.14 | EEEET | 11 | 1.44 |
| 1815 | 2QMQ:A | signaling protein | 50 | 54 | VTFTV | 5 | 6.18 | EEEEE | 9 | 1.96 |
| 1816 | 2VIF:A | signaling protein | 383 | 387 | GWYWG | 5 | 23.64 | TCBCC | 8 | -0.78 |
| 1817 | 2ZAY:A | signaling protein | 23 | 27 | ASISA | 5 | 43.02 | HHHHH | 4 | 1.30 |
| 1818 | 2ZAY:A | signaling protein | 44 | 48 | AVPVA | 5 | 27.26 | HHHHH | 4 | 2.08 |
| 1819 | 3HYN:A | signaling protein | 183 | 187 | KYYYK | 5 | 86.82 | EEECC | 6 | -2.34 |
| 1820 | 3K7I:B | signaling protein | 83 | 87 | PNYNP | 5 | 74.22 | ECCCT | 6 | -2.30 |
| 1821 | 3LQ9:A | signaling protein | 207 | 211 | SLTLS | 5 | 40.12 | CEECC | 7 | 1.06 |
| 1822 | 3NSU:A | signaling protein | 483 | 487 | KSYSK | 5 | 92.04 | SSEEE | 4 | -2.14 |
| 1823 | 3NSU:A | signaling protein | 514 | 518 | SLALS | 5 | 20.52 | EEEGG | 4 | 1.56 |
| 1824 | 3Q72:A | signaling protein | 165 | 169 | YVIVY | 5 | 3.98 | EEEEE | 10 | 2.06 |
| 1825 | 3SG0:A | signaling protein | 168 | 172 | YGEGY | 5 | 19.52 | HHHHH | 9 | -1.38 |
| 1826 | 3SNK:A | signaling protein | 102 | 106 | RVLVR | 5 | 88.26 | HHHHH | 4 | 0.64 |
| 1827 | 3TJY:A | signaling protein | 204 | 208 | LAIAL | 5 | 17.96 | HHHHH | 6 | 3.14 |
| 1828 | 4AFF:A | signaling protein | 34 | 38 | RGFGR | 5 | 109.38 | EECCC | 5 | -1.40 |
| 1829 | 1XOD:A | signaling protein | 31 | 36 | LGGGGL | 6 | 47.32 | GGGCCC | 5 | 1.00 |
| 1830 | 1MB3:A | signaling protein, cell cycle | 92 | 96 | ERIRE | 5 | 58.74 | HHHHH | 5 | -2.30 |
| 1831 | 3BQA:A | signaling protein, transferase | 130 | 134 | SLLLS | 5 | 43.70 | HHHHC | 8 | 1.96 |
| 1832 | 2ABS:A | signaling protein,transferase | 354 | 358 | FSLSF | 5 | 41.64 | CCCCC | 4 | 1.56 |
| 1833 | 2ABS:A | signaling protein,transferase | 165 | 169 | YATAY | 5 | 15.20 | EEEGG | 9 | 0.06 |
| 1834 | 2QIY:B | signaling protein/hydrolase | 56 | 60 | TVKVT | 5 | 58.38 | EEEEE | 6 | 0.62 |
| 1835 | 2QIY:B | signaling protein/hydrolase | 79 | 83 | LKLKL | 5 | 13.70 | EEEEE | 6 | 0.72 |
| 1836 | 2QIY:B | signaling protein/hydrolase | 28 | 32 | AYFYA | 5 | 49.22 | GGGEE | 9 | 0.76 |
| 1837 | 1GCQ:C | signaling protein/signaling protein | 641 | 645 | NTATN | 5 | 60.40 | ETTTT | 5 | -1.32 |
| 1838 | 3IOL:A | signaling protein/signaling protein | 44 | 48 | RQCQR | 5 | 78.40 | HHHHH | 9 | -2.70 |
| 1839 | 2ZYZ:C | splicing | 67 | 71 | VLALV | 5 | 0.00 | EEEEE | 8 | 3.56 |
| 1840 | 3EGN:A | splicing | 477 | 481 | KAAAK | 5 | 65.30 | HHHHH | 7 | -0.48 |
| 1841 | 3S6N:G | splicing | 16 | 20 | KLSLK | 5 | 56.44 | EEEEE | 7 | -0.20 |
| 1842 | 2BNL:F | stress-response | 13 | 17 | ENQNE | 5 | 92.96 | HTHHH | 8 | -3.50 |
| 1843 | 2BNL:F | stress-response | 30 | 34 | SEQES | 5 | 95.34 | HTTSS | 8 | -2.42 |
| 1844 | 2BNL:F | stress-response | 84 | 88 | LATAL | 5 | 14.14 | HHHHH | 6 | 2.10 |
| 1845 | 2Q3G:A | structural genomics | 43 | 47 | GVAVG | 5 | 41.82 | TCCTT | 6 | 1.88 |
| 1846 | 3H3M:B | structural genomics | 58 | 62 | ERLRE | 5 | 86.62 | HHHCC | 6 | -2.44 |
| 1847 | 1DM9:B | structural genomics | 24 | 28 | RALAR | 5 | 72.40 | HHHHH | 5 | -0.32 |
| 1848 | 2Q3G:A | structural genomics | 78 | 82 | SLGLS | 5 | 11.94 | EEEEE | 9 | 1.12 |
| 1849 | 3KEV:A | structural genomics, unknown function | 101 | 107 | YQFVFQY | 7 | 30.07 | HHHHHHH | 9 | 0.03 |
| 1850 | 1PBJ:A | structural genomics, unknown function | 13 | 17 | TIDIT | 5 | 56.92 | EEETT | 4 | 0.82 |
| 1851 | 2PD1:D | structural genomics, unknown function | 27 | 31 | ALPLA | 5 | 39.48 | THHHH | 8 | 1.92 |
| 1852 | 1V8D:C | structural genomics, unknown function | 96 | 101 | LLPPLL | 6 | 31.13 | HHHHHH | 7 | 2.00 |
| 1853 | 2B0A:A | structural genomics, unknown function | 150 | 154 | LNENL | 5 | 28.48 | BCGGG | 8 | -0.58 |
| 1854 | 2A5Z:C | structural genomics, unknown function | 142 | 148 | SIGLGIS | 7 | 37.99 | ECTTCCS | 8 | 1.49 |
| 1855 | 1ZWY:A | structural genomics, unknown function | 168 | 172 | ILALI | 5 | 2.54 | HHHTH | 10 | 3.68 |
| 1856 | 2O1M:B | structural genomics, unknown function | 88 | 92 | KEREK | 5 | 118.30 | HHHHH | 4 | -3.86 |
| 1857 | 3DR5:A | structural genomics, unknown function | 161 | 165 | LADAL | 5 | 20.36 | ETTTT | 8 | 1.54 |
| 1858 | 3G3L:A | structural genomics, unknown function | 132 | 137 | PPVVPP | 6 | 57.87 | CCSSCT | 5 | 0.33 |
| 1859 | 1G2R:A | structural genomics, unknown function | 93 | 97 | ELGLE | 5 | 97.38 | HTTCC | 6 | 0.04 |
| 1860 | 1IXL:A | structural genomics, unknown function | 49 | 53 | GFTFG | 5 | 40.58 | HHHHH | 6 | 0.82 |
| 1861 | 1JZT:B | structural genomics, unknown function | 39 | 43 | VAQAV | 5 | 1.80 | HHHHH | 9 | 1.70 |
| 1862 | 1M3S:A | structural genomics, unknown function | 85 | 89 | GSGSG | 5 | 18.50 | ECSSS | 7 | -0.56 |
| 1863 | 1MZG:B | structural genomics, unknown function | 38 | 42 | RDEDR | 5 | 99.30 | CTTTC | 5 | -3.90 |
| 1864 | 1NC7:C | structural genomics, unknown function | 74 | 78 | PKCKP | 5 | 64.96 | CTTCC | 4 | -1.70 |
| 1865 | 1PBJ:A | structural genomics, unknown function | 85 | 90 | KEAAEK | 6 | 72.32 | HHHHHH | 6 | -1.87 |
| 1866 | 1PBJ:A | structural genomics, unknown function | 9 | 13 | TDVDT | 5 | 76.32 | CSCCE | 4 | -0.84 |
| 1867 | 1PBJ:A | structural genomics, unknown function | 54 | 58 | EAIAE | 5 | 84.06 | HHHHH | 6 | 0.22 |
| 1868 | 1PBJ:A | structural genomics, unknown function | 41 | 45 | GVRVG | 5 | 58.04 | TEEEE | 4 | 0.62 |
| 1869 | 1R0U:A | structural genomics, unknown function | 97 | 101 | KTSTK | 5 | 51.36 | EEEEE | 10 | -2.00 |
| 1870 | 1R4V:A | structural genomics, unknown function | 40 | 44 | LDIDL | 5 | 34.22 | HCCCC | 5 | 1.02 |
| 1871 | 1RTT:A | structural genomics, unknown function | 64 | 68 | ERFRE | 5 | 76.06 | HHHHH | 11 | -2.64 |
| 1872 | 1RTT:A | structural genomics, unknown function | 115 | 119 | GASAG | 5 | 22.78 | EECSS | 8 | 0.40 |
| 1873 | 1S9U:A | structural genomics, unknown function | 42 | 46 | WQAQW | 5 | 79.38 | CGGGS | 3 | -1.40 |
| 1874 | 1S9U:A | structural genomics, unknown function | 187 | 191 | AQWQA | 5 | 63.16 | HHHHH | 7 | -0.86 |
| 1875 | 1SDI:A | structural genomics, unknown function | 116 | 120 | LQRQL | 5 | 80.56 | THHHH | 6 | -0.78 |
| 1876 | 1SFS:A | structural genomics, unknown function | 152 | 156 | QVAVQ | 5 | 37.92 | HHHHH | 5 | 0.64 |
| 1877 | 1T3U:D | structural genomics, unknown function | 82 | 86 | ERVRE | 5 | 95.40 | HHHHH | 9 | -2.36 |
| 1878 | 1TXJ:A | structural genomics, unknown function | 68 | 72 | VIDIV | 5 | 22.60 | EEHHH | 8 | 2.78 |
| 1879 | 1U69:D | structural genomics, unknown function | 132 | 136 | RAAAR | 5 | 42.30 | HHHHH | 9 | -0.72 |
| 1880 | 1U7I:A | structural genomics, unknown function | 93 | 97 | LAEAL | 5 | 38.34 | HHHHH | 5 | 1.54 |
| 1881 | 1UJ8:A | structural genomics, unknown function | 31 | 35 | PDLDP | 5 | 71.64 | TTSCG | 5 | -1.28 |
| 1882 | 1V8D:C | structural genomics, unknown function | 141 | 146 | KAGGAK | 6 | 21.97 | TBBCHH | 6 | -0.83 |
| 1883 | 1V8D:C | structural genomics, unknown function | 184 | 188 | PVAVP | 5 | 60.76 | SCEEE | 6 | 1.40 |
| 1884 | 1VH5:A | structural genomics, unknown function | 96 | 100 | GRVRG | 5 | 33.36 | SEEEE | 11 | -1.12 |
| 1885 | 1VHF:A | structural genomics, unknown function | 60 | 64 | EKEKE | 5 | 72.36 | GGHHH | 7 | -3.66 |
| 1886 | 1VHU:A | structural genomics, unknown function | 119 | 123 | LKEKL | 5 | 47.18 | HHHHH | 9 | -0.74 |
| 1887 | 1VK1:A | structural genomics, unknown function | 184 | 188 | DAKAD | 5 | 47.86 | HHHHH | 10 | -1.46 |
| 1888 | 1WV9:A | structural genomics, unknown function | 51 | 56 | LPRRPL | 6 | 41.48 | CCSSCE | 10 | -0.77 |
| 1889 | 1WWI:A | structural genomics, unknown function | 110 | 114 | LAGAL | 5 | 1.38 | HHHHH | 8 | 2.16 |
| 1890 | 1WWZ:B | structural genomics, unknown function | 47 | 51 | KWCWK | 5 | 89.94 | HHHHH | 7 | -1.42 |
| 1891 | 1WWZ:B | structural genomics, unknown function | 138 | 142 | KFGFK | 5 | 44.20 | HTTCE | 4 | -0.52 |
| 1892 | 1WWZ:B | structural genomics, unknown function | 3 | 7 | EIKIE | 5 | 82.44 | CEEEE | 4 | -0.38 |
| 1893 | 1X6I:B | structural genomics, unknown function | 233 | 237 | DSLSD | 5 | 65.98 | GGSCH | 8 | -0.96 |
| 1894 | 1X6I:B | structural genomics, unknown function | 241 | 245 | RIFIR | 5 | 41.62 | HHHHH | 10 | 0.56 |
| 1895 | 1Y7R:A | structural genomics, unknown function | 99 | 103 | SVYVS | 5 | 10.80 | TCEEE | 14 | 1.10 |
| 1896 | 1YBX:B | structural genomics, unknown function | 18 | 22 | KQAQK | 5 | 75.94 | HHHHH | 7 | -2.60 |
| 1897 | 1YBX:B | structural genomics, unknown function | 43 | 47 | AGGGA | 5 | 35.18 | ETTTT | 3 | 0.48 |
| 1898 | 1YOZ:A | structural genomics, unknown function | 120 | 124 | VEKEV | 5 | 74.92 | HHHTC | 3 | -0.50 |
| 1899 | 1YQH:A | structural genomics, unknown function | 47 | 51 | LEGEL | 5 | 20.60 | EEECH | 11 | 0.04 |
| 1900 | 1YRE:A | structural genomics, unknown function | 68 | 72 | ALPLA | 5 | 5.08 | EEEEE | 7 | 1.92 |
| 1901 | 1Z6M:A | structural genomics, unknown function | 44 | 48 | EESEE | 5 | 77.14 | HHHHH | 10 | -2.96 |
| 1902 | 1ZHV:A | structural genomics, unknown function | 56 | 60 | DVRVD | 5 | 87.22 | TSEEE | 7 | -0.62 |
| 1903 | 1ZHV:A | structural genomics, unknown function | 5 | 9 | IKLKI | 5 | 39.68 | CEEEE | 7 | 1.00 |
| 1904 | 1ZL0:A | structural genomics, unknown function | 149 | 154 | REQQER | 6 | 82.37 | HHHHHH | 9 | -3.83 |
| 1905 | 1ZWY:A | structural genomics, unknown function | 66 | 70 | NRVRN | 5 | 40.46 | HHHHH | 13 | -2.36 |
| 1906 | 1ZWY:A | structural genomics, unknown function | 166 | 170 | ALILA | 5 | 6.54 | HHHHH | 13 | 3.14 |
| 1907 | 1ZX3:A | structural genomics, unknown function | 73 | 77 | ETNTE | 5 | 81.46 | HHHHH | 8 | -2.38 |
| 1908 | 2A5Z:C | structural genomics, unknown function | 143 | 147 | IGLGI | 5 | 43.38 | CTTCC | 6 | 2.40 |
| 1909 | 2AH5:A | structural genomics, unknown function | 26 | 30 | FTYTF | 5 | 23.86 | HHHHH | 9 | 0.58 |
| 1910 | 2AO9:F | structural genomics, unknown function | 20 | 24 | LKQKL | 5 | 76.44 | HGGGS | 4 | -0.74 |
| 1911 | 2APL:A | structural genomics, unknown function | 90 | 94 | LPSPL | 5 | 58.50 | SCTTH | 5 | 0.72 |
| 1912 | 2APL:A | structural genomics, unknown function | 78 | 82 | LVSVL | 5 | 9.74 | HHHHH | 8 | 3.04 |
| 1913 | 2ATR:A | structural genomics, unknown function | 38 | 42 | LSHSL | 5 | 67.02 | HTSCS | 6 | 0.56 |
| 1914 | 2AUA:B | structural genomics, unknown function | 186 | 191 | IEVVEI | 6 | 46.55 | EEEEEE | 9 | 1.73 |
| 1915 | 2AZW:A | structural genomics, unknown function | 48 | 52 | GEIEG | 5 | 48.18 | EECCT | 8 | -0.66 |
| 1916 | 2B0A:A | structural genomics, unknown function | 148 | 152 | ENLNE | 5 | 23.98 | ECBCG | 5 | -2.04 |
| 1917 | 2B0A:A | structural genomics, unknown function | 5 | 9 | LTQTL | 5 | 44.92 | CBCCC | 5 | 0.54 |
| 1918 | 2CVI:A | structural genomics, unknown function | 68 | 72 | MTSTM | 5 | 35.88 | EEEEE | 6 | 0.32 |
| 1919 | 2D7V:A | structural genomics, unknown function | 152 | 156 | VETEV | 5 | 48.68 | BCSEE | 7 | 0.14 |
| 1920 | 2E6X:A | structural genomics, unknown function | 3 | 8 | KDLLDK | 6 | 99.15 | HHHHHH | 4 | -1.20 |
| 1921 | 2E8E:A | structural genomics, unknown function | 43 | 47 | GSCSG | 5 | 23.54 | HHHHH | 10 | 0.02 |
| 1922 | 2EGJ:B | structural genomics, unknown function | 85 | 89 | FTFTF | 5 | 30.66 | SEEEE | 8 | 1.40 |
| 1923 | 2FA8:A | structural genomics, unknown function | 7 | 11 | RIAIR | 5 | 47.04 | EEEEE | 8 | 0.36 |
| 1924 | 2FB6:A | structural genomics, unknown function | 55 | 59 | TQVQT | 5 | 67.16 | HHHHH | 7 | -0.84 |
| 1925 | 2FHP:A | structural genomics, unknown function | 88 | 92 | KEPEK | 5 | 96.22 | TCGGG | 6 | -3.28 |
| 1926 | 2FZV:A | structural genomics, unknown function | 7 | 11 | SDPDS | 5 | 76.62 | SSTTC | 4 | -2.04 |
| 1927 | 2FZV:A | structural genomics, unknown function | 47 | 51 | RSFSR | 5 | 39.90 | SCHHH | 4 | -1.56 |
| 1928 | 2FZV:A | structural genomics, unknown function | 6 | 12 | LSDPDSL | 7 | 58.99 | CSSTTCC | 4 | -0.37 |
| 1929 | 2FZV:A | structural genomics, unknown function | 24 | 30 | PALGLAP | 7 | 45.53 | TTTTTSC | 4 | 1.09 |
| 1930 | 2FZV:A | structural genomics, unknown function | 25 | 29 | ALGLA | 5 | 44.74 | TTTTS | 4 | 2.16 |
| 1931 | 2GDQ:B | structural genomics, unknown function | 223 | 227 | DQPQD | 5 | 87.60 | SCHHH | 7 | -3.12 |
| 1932 | 2GDQ:B | structural genomics, unknown function | 231 | 235 | LRSRL | 5 | 65.66 | HHTTC | 7 | -0.44 |
| 1933 | 2GDQ:B | structural genomics, unknown function | 104 | 108 | AAKAA | 5 | 33.50 | HHHHT | 7 | 0.66 |
| 1934 | 2GDQ:B | structural genomics, unknown function | 158 | 162 | IKVKI | 5 | 1.10 | EEEEC | 7 | 1.08 |
| 1935 | 2GFQ:C | structural genomics, unknown function | 266 | 270 | ELGLE | 5 | 75.26 | HHTCE | 10 | 0.04 |
| 1936 | 2GNP:A | structural genomics, unknown function | 234 | 238 | TKVKT | 5 | 88.42 | HHTTC | 5 | -1.00 |
| 1937 | 2HD9:A | structural genomics, unknown function | 48 | 52 | EKDKE | 5 | 108.22 | EECTT | 5 | -3.66 |
| 1938 | 2HEK:B | structural genomics, unknown function | 316 | 320 | VEKEV | 5 | 57.56 | EEECS | 7 | -0.50 |
| 1939 | 2HEK:B | structural genomics, unknown function | 217 | 221 | YVQVY | 5 | 33.16 | HHHTT | 7 | 0.46 |
| 1940 | 2HHG:A | structural genomics, unknown function | 48 | 52 | REIER | 5 | 108.84 | HHHHH | 5 | -2.30 |
| 1941 | 2HUJ:A | structural genomics, unknown function | 9 | 13 | QLLLQ | 5 | 36.86 | HHHHH | 9 | 0.88 |
| 1942 | 2I15:A | structural genomics, unknown function | 45 | 49 | DDYDD | 5 | 39.02 | SCCCS | 7 | -3.06 |
| 1943 | 2I15:A | structural genomics, unknown function | 109 | 113 | GVDVG | 5 | 53.32 | TCCTT | 7 | 0.82 |
| 1944 | 2I3D:A | structural genomics, unknown function | 20 | 24 | SKEKS | 5 | 115.02 | CSSTT | 6 | -2.58 |
| 1945 | 2I3D:A | structural genomics, unknown function | 62 | 66 | RFNFR | 5 | 15.00 | EECCT | 6 | -1.38 |
| 1946 | 2I5U:A | structural genomics, unknown function | 46 | 50 | AIEIA | 5 | 25.94 | HHHHH | 9 | 1.82 |
| 1947 | 2IMJ:D | structural genomics, unknown function | 9 | 13 | PPLPP | 5 | 60.22 | SSCSS | 3 | -0.52 |
| 1948 | 2NR7:A | structural genomics, unknown function | 46 | 50 | DGDGD | 5 | 65.48 | TTSTT | 6 | -2.26 |
| 1949 | 2O1M:B | structural genomics, unknown function | 85 | 89 | EKSKE | 5 | 98.04 | CCCHH | 3 | -3.12 |
| 1950 | 2O1M:B | structural genomics, unknown function | 57 | 61 | KFTFK | 5 | 66.60 | EEEEE | 7 | -0.58 |
| 1951 | 2OKQ:B | structural genomics, unknown function | 61 | 65 | EENEE | 5 | 100.36 | CTTEE | 6 | -3.50 |
| 1952 | 2OKQ:B | structural genomics, unknown function | 13 | 18 | ADKKDA | 6 | 74.37 | GGGHHH | 6 | -1.87 |
| 1953 | 2OKQ:B | structural genomics, unknown function | 23 | 27 | AAKAA | 5 | 39.34 | HHHHH | 9 | 0.66 |
| 1954 | 2PD1:D | structural genomics, unknown function | 54 | 58 | DAFAD | 5 | 26.86 | EEESS | 8 | -0.12 |
| 1955 | 2PD1:D | structural genomics, unknown function | 24 | 28 | LASAL | 5 | 32.28 | HHHTH | 8 | 2.08 |
| 1956 | 2PIH:A | structural genomics, unknown function | 80 | 84 | EELEE | 5 | 83.20 | HHHHH | 8 | -2.04 |
| 1957 | 2Q00:B | structural genomics, unknown function | 12 | 16 | EEAEE | 5 | 36.14 | HHHHH | 13 | -2.44 |
| 1958 | 2Q22:C | structural genomics, unknown function | 33 | 37 | SEKES | 5 | 47.50 | HHHHH | 9 | -2.50 |
| 1959 | 2Q3P:A | structural genomics, unknown function | 75 | 79 | EAVAE | 5 | 16.13 | HHHHH | 9 | 0.16 |
| 1960 | 2Q52:A | structural genomics, unknown function | 189 | 193 | QFFFQ | 5 | 10.33 | HHHHH | 14 | 0.28 |
| 1961 | 2Q52:A | structural genomics, unknown function | 49 | 53 | FGSGF | 5 | 2.47 | HCGGG | 15 | 0.80 |
| 1962 | 2QGS:A | structural genomics, unknown function | 120 | 124 | RDADR | 5 | 32.78 | HHHHH | 11 | -2.84 |
| 1963 | 2QGS:A | structural genomics, unknown function | 130 | 134 | AIGIA | 5 | 5.68 | HHHHH | 11 | 2.44 |
| 1964 | 2QIK:A | structural genomics, unknown function | 55 | 59 | GYIYG | 5 | 21.60 | SEEEE | 8 | 0.22 |
| 1965 | 2QKP:A | structural genomics, unknown function | 333 | 337 | NLILN | 5 | 16.64 | HHHHH | 9 | 1.02 |
| 1966 | 2QNT:A | structural genomics, unknown function | 77 | 81 | ADVDA | 5 | 59.32 | SCHHH | 6 | 0.16 |
| 1967 | 2QZI:D | structural genomics, unknown function | 73 | 77 | IDKDI | 5 | 47.14 | SCTTT | 7 | -0.38 |
| 1968 | 2R5X:A | structural genomics, unknown function | 115 | 119 | EKIKE | 5 | 108.86 | HTTTT | 4 | -2.06 |
| 1969 | 2RBG:A | structural genomics, unknown function | 6 | 10 | ILTLI | 5 | 2.12 | EEEEE | 10 | 3.18 |
| 1970 | 2YVT:A | structural genomics, unknown function | 68 | 72 | HENEH | 5 | 93.88 | HHHHH | 8 | -3.38 |
| 1971 | 3BS4:A | structural genomics, unknown function | 238 | 242 | FEYEF | 5 | 44.78 | EEEEE | 8 | -0.54 |
| 1972 | 3BT5:A | structural genomics, unknown function | 47 | 51 | ALDLA | 5 | 26.14 | HHHHH | 8 | 1.54 |
| 1973 | 3C4S:B | structural genomics, unknown function | 7 | 11 | TVRVT | 5 | 34.56 | EEEEC | 10 | 0.50 |
| 1974 | 3C8C:A | structural genomics, unknown function | 149 | 153 | PRVRP | 5 | 50.06 | GGGSH | 9 | -1.60 |
| 1975 | 3C8C:A | structural genomics, unknown function | 66 | 70 | VSDSV | 5 | 39.90 | HHHHH | 6 | 0.66 |
| 1976 | 3C9Q:A | structural genomics, unknown function | 71 | 75 | PGDGP | 5 | 67.52 | TTSCC | 2 | -1.50 |
| 1977 | 3CBN:A | structural genomics, unknown function | 77 | 81 | RAIAR | 5 | 37.08 | HHHSC | 7 | -0.18 |
| 1978 | 3DR5:A | structural genomics, unknown function | 203 | 207 | LGAGL | 5 | 23.98 | STTCE | 9 | 1.72 |
| 1979 | 3DR5:A | structural genomics, unknown function | 158 | 162 | ALVLA | 5 | 2.22 | EEEET | 9 | 3.08 |
| 1980 | 3DTZ:B | structural genomics, unknown function | 161 | 166 | SYTTYS | 6 | 22.78 | EEEEEC | 7 | -0.93 |
| 1981 | 3DTZ:B | structural genomics, unknown function | 113 | 118 | RLPPLR | 6 | 82.45 | HSCCCS | 7 | -0.77 |
| 1982 | 3DTZ:B | structural genomics, unknown function | 88 | 92 | SSFSS | 5 | 27.98 | EEEEC | 7 | -0.08 |
| 1983 | 3ERM:E | structural genomics, unknown function | 66 | 70 | FAAAF | 5 | 72.24 | HHHHT | 6 | 2.20 |
| 1984 | 3EYP:B | structural genomics, unknown function | 353 | 357 | AKVKA | 5 | 42.54 | CEEEE | 5 | 0.00 |
| 1985 | 3F14:A | structural genomics, unknown function | 67 | 71 | QNENQ | 5 | 80.34 | ECSSE | 5 | -3.50 |
| 1986 | 3F14:A | structural genomics, unknown function | 65 | 73 | VIQNENQIV | 9 | 62.64 | EEECSSEEE | 5 | -0.01 |
| 1987 | 3F2Z:A | structural genomics, unknown function | 310 | 314 | SGEGS | 5 | 55.78 | STTCT | 5 | -1.18 |
| 1988 | 3FLE:B | structural genomics, unknown function | 184 | 188 | ENVNE | 5 | 64.24 | BCTTC | 8 | -1.96 |
| 1989 | 3FUY:C | structural genomics, unknown function | 42 | 46 | DLCLD | 5 | 42.74 | HHHHH | 8 | 0.62 |
| 1990 | 3FUY:C | structural genomics, unknown function | 9 | 13 | LSPSL | 5 | 17.20 | EETTE | 5 | 0.88 |
| 1991 | 3FVV:A | structural genomics, unknown function | 181 | 185 | SYFYS | 5 | 9.50 | EEEEE | 12 | -0.28 |
| 1992 | 3FVV:A | structural genomics, unknown function | 172 | 176 | GLALG | 5 | 55.68 | TCCGG | 5 | 1.72 |
| 1993 | 3FXH:A | structural genomics, unknown function | 95 | 99 | ALLLA | 5 | 18.30 | HHHHH | 5 | 3.00 |
| 1994 | 3FYF:A | structural genomics, unknown function | 122 | 126 | GNVNG | 5 | 50.04 | EEEEE | 7 | -0.72 |
| 1995 | 3G3L:A | structural genomics, unknown function | 155 | 161 | KAVEVAK | 7 | 45.30 | HHHHHHS | 6 | 0.10 |
| 1996 | 3G3L:A | structural genomics, unknown function | 156 | 160 | AVEVA | 5 | 28.62 | HHHHH | 6 | 1.70 |
| 1997 | 3GMI:A | structural genomics, unknown function | 71 | 75 | EKGKE | 5 | 66.80 | HHHHT | 8 | -3.04 |
| 1998 | 3GMI:A | structural genomics, unknown function | 284 | 288 | LYEYL | 5 | 41.54 | GGGTS | 8 | 0.30 |
| 1999 | 3GMI:A | structural genomics, unknown function | 83 | 87 | LPGPL | 5 | 0.86 | ECCTT | 8 | 0.80 |
| 2000 | 3GNJ:A | structural genomics, unknown function | 93 | 98 | VEDDEV | 6 | 70.68 | CCHHHH | 5 | -0.93 |
| 2001 | 3GRD:B | structural genomics, unknown function | 100 | 104 | FEAEF | 5 | 46.26 | EEEEE | 8 | 0.08 |
| 2002 | 3GRD:B | structural genomics, unknown function | 24 | 28 | LAEAL | 5 | 41.12 | HHHHE | 7 | 1.54 |
| 2003 | 3H6P:C | structural genomics, unknown function | 54 | 58 | WQTQW | 5 | 46.64 | HHHHH | 12 | -1.90 |
| 2004 | 3H9W:A | structural genomics, unknown function | 105 | 109 | VVHVV | 5 | 38.66 | EEEEE | 9 | 2.72 |
| 2005 | 3HRG:A | structural genomics, unknown function | 217 | 221 | LTGTL | 5 | 33.80 | EEECC | 7 | 1.16 |
| 2006 | 3HTY:P | structural genomics, unknown function | 38 | 42 | GQVQG | 5 | 31.32 | EEEEE | 10 | -0.72 |
| 2007 | 3IJD:B | structural genomics, unknown function | 28 | 32 | EKIKE | 5 | 89.58 | HHHHH | 6 | -2.06 |
| 2008 | 3IJD:B | structural genomics, unknown function | 272 | 276 | GIPIG | 5 | 14.18 | TCCEE | 8 | 1.32 |
| 2009 | 3IJM:A | structural genomics, unknown function | 49 | 53 | EPLPE | 5 | 10.36 | EEEES | 10 | -1.28 |
| 2010 | 3IJM:A | structural genomics, unknown function | 121 | 125 | LRYRL | 5 | 47.74 | EEEET | 10 | -0.54 |
| 2011 | 3IRS:A | structural genomics, unknown function | 62 | 66 | GVCVG | 5 | 0.84 | EEEEC | 8 | 2.02 |
| 2012 | 3K4I:A | structural genomics, unknown function | 85 | 89 | SVIVS | 5 | 5.04 | EEEEE | 10 | 2.26 |
| 2013 | 3K67:B | structural genomics, unknown function | 68 | 72 | FDEDF | 5 | 52.00 | HCHHH | 7 | -0.98 |
| 2014 | 3KB2:B | structural genomics, unknown function | 93 | 97 | KAKAK | 5 | 60.34 | TTTEE | 10 | -1.62 |
| 2015 | 3KEV:A | structural genomics, unknown function | 80 | 84 | LKRKL | 5 | 56.96 | HHHHH | 9 | -0.94 |
| 2016 | 3KEV:A | structural genomics, unknown function | 102 | 106 | QFVFQ | 5 | 37.60 | HHHHH | 9 | 0.56 |
| 2017 | 3KGZ:B | structural genomics, unknown function | 79 | 83 | AHVHA | 5 | 18.90 | SSCEE | 7 | 0.28 |
| 2018 | 3KL2:L | structural genomics, unknown function | 101 | 105 | EGYGE | 5 | 60.18 | TTCTT | 6 | -1.82 |
| 2019 | 3KOP:E | structural genomics, unknown function | 57 | 61 | PAFAP | 5 | 67.16 | ECCSS | 3 | 0.64 |
| 2020 | 3KOP:E | structural genomics, unknown function | 118 | 122 | NLLLN | 5 | 6.70 | CCCEE | 3 | 0.88 |
| 2021 | 3KVP:B | structural genomics, unknown function | 39 | 43 | YIKIY | 5 | 39.42 | EEEEE | 11 | 0.50 |
| 2022 | 3KZP:B | structural genomics, unknown function | 76 | 80 | AINIA | 5 | 11.78 | EEEEC | 10 | 1.82 |
| 2023 | 3LMB:A | structural genomics, unknown function | 39 | 43 | TLILT | 5 | 11.26 | EEEEE | 10 | 2.14 |
| 2024 | 3M73:A | structural genomics, unknown function | 235 | 239 | FLQLF | 5 | 46.26 | HHHHH | 8 | 1.94 |
| 2025 | 3M73:A | structural genomics, unknown function | 22 | 26 | ALSLA | 5 | 7.92 | HHHHH | 8 | 2.08 |
| 2026 | 3M73:A | structural genomics, unknown function | 47 | 51 | VASAV | 5 | 35.20 | HHHHH | 8 | 2.24 |
| 2027 | 3M7A:B | structural genomics, unknown function | 31 | 35 | AAEAA | 5 | 50.80 | HHHHS | 3 | 0.74 |
| 2028 | 3MW6:B | structural genomics, unknown function | 103 | 107 | QAIAQ | 5 | 31.98 | HHHHH | 8 | 0.22 |
| 2029 | 3NJA:A | structural genomics, unknown function | 91 | 96 | RELLER | 6 | 57.93 | EEEEEE | 9 | -1.40 |
| 2030 | 3NNG:B | structural genomics, unknown function | 208 | 212 | ADDDA | 5 | 82.68 | GGCHH | 7 | -1.38 |
| 2031 | 3NNG:B | structural genomics, unknown function | 262 | 266 | YGSGY | 5 | 71.00 | SCGGG | 7 | -0.84 |
| 2032 | 3O12:A | structural genomics, unknown function | 65 | 69 | QVRVQ | 5 | 28.06 | EEEEE | 10 | -0.62 |
| 2033 | 3OBH:B | structural genomics, unknown function | 11 | 15 | EFTFE | 5 | 110.66 | CCCEE | 5 | -0.42 |
| 2034 | 3OGH:B | structural genomics, unknown function | 96 | 100 | GSISG | 5 | 30.96 | HHHHH | 8 | 0.42 |
| 2035 | 3OLQ:A | structural genomics, unknown function | 211 | 215 | PDFDP | 5 | 83.48 | TTCCH | 5 | -1.48 |
| 2036 | 3OMD:A | structural genomics, unknown function | 49 | 53 | FFSFF | 5 | 17.52 | HHHHH | 8 | 2.08 |
| 2037 | 3OMT:B | structural genomics, unknown function | 24 | 28 | LTETL | 5 | 60.48 | HHHHH | 6 | 0.54 |
| 2038 | 3Q63:A | structural genomics, unknown function | 98 | 102 | TPTPT | 5 | 87.22 | EECSS | 4 | -1.06 |
| 2039 | 3QOO:A | structural genomics, unknown function | 82 | 86 | VTFTV | 5 | 20.18 | EEEEE | 8 | 1.96 |
| 2040 | 3RH3:B | structural genomics, unknown function | 224 | 228 | LEKEL | 5 | 69.16 | HHHHH | 8 | -0.66 |
| 2041 | 3VCX:B | structural genomics, unknown function | 16 | 20 | AATAA | 5 | 36.52 | HHHHH | 6 | 1.30 |
| 2042 | 3F14:A | structural genomics, unknown function | 66 | 72 | IQNENQI | 7 | 70.86 | EECSSEE | 5 | -1.21 |
| 2043 | 2QIK:A | structural genomics, unknown function | 54 | 60 | EGYIYGE | 7 | 37.50 | CSEEEEE | 12 | -0.84 |
| 2044 | 2B06:A | structural genomics/unknown function | 125 | 129 | MLPLM | 5 | 29.26 | HHHHH | 6 | 1.96 |
| 2045 | 2B06:A | structural genomics/unknown function | 49 | 53 | EAFAE | 5 | 63.74 | SCHHH | 7 | -0.12 |
| 2046 | 2NZC:A | structural genomics/unknown function | 55 | 59 | TDNDT | 5 | 57.14 | ECHHH | 6 | -2.38 |
| 2047 | 1HQZ:4 | structural protein | 54 | 60 | DFLQLFD | 7 | 43.90 | HHTTCCC | 0 | 0.39 |
| 2048 | 2QF4:B | structural protein | 190 | 196 | YGIIIGY | 7 | 24.29 | EEEEEEE | 9 | 1.44 |
| 2049 | 1HQZ:4 | structural protein | 55 | 59 | FLQLF | 5 | 38.66 | HTTCC | 0 | 1.94 |
| 2050 | 1JXO:B | structural protein | 440 | 444 | DKTKD | 5 | 65.96 | CHHHH | 4 | -3.10 |
| 2051 | 1KTH:A | structural protein | 49 | 53 | KECEK | 5 | 88.74 | HHHHH | 6 | -2.46 |
| 2052 | 1KTH:A | structural protein | 36 | 40 | GGCGG | 5 | 35.86 | CSBSC | 7 | 0.18 |
| 2053 | 1T61:A | structural protein | 185 | 189 | YANAY | 5 | 8.34 | CTTCE | 6 | -0.50 |
| 2054 | 1T61:A | structural protein | 115 | 119 | AMVMA | 5 | 19.80 | SCEEE | 6 | 2.32 |
| 2055 | 1U7L:A | structural protein | 338 | 342 | SKCKS | 5 | 61.56 | HHHHH | 7 | -1.38 |
| 2056 | 1X6Z:A | structural protein | 35 | 39 | SALAS | 5 | 32.00 | HHHHH | 17 | 1.16 |
| 2057 | 1YA5:T | structural protein | 74 | 78 | LGRGL | 5 | 71.00 | TTSCE | 5 | 0.46 |
| 2058 | 1YA5:T | structural protein | 58 | 62 | QVLVQ | 5 | 13.68 | EEEEE | 10 | 1.04 |
| 2059 | 2E3H:A | structural protein | 255 | 259 | GAVAG | 5 | 52.76 | SEETT | 5 | 1.40 |
| 2060 | 2HD3:K | structural protein | 78 | 82 | VIGIV | 5 | 5.22 | EEEEC | 9 | 3.40 |
| 2061 | 2QF4:B | structural protein | 191 | 195 | GIIIG | 5 | 15.38 | EEEEE | 8 | 2.54 |
| 2062 | 2RIK:A | structural protein | 208 | 212 | LECEL | 5 | 34.06 | EEEEC | 6 | 0.62 |
| 2063 | 2W4S:A | structural protein | 464 | 468 | QITIQ | 5 | 77.22 | SSCHH | 7 | 0.26 |
| 2064 | 3BWZ:A | structural protein | 116 | 120 | VKFKV | 5 | 26.38 | EEEEE | 10 | 0.68 |
| 2065 | 3EDV:B | structural protein | 1997 | 2001 | EKRKE | 5 | 76.62 | HHHHH | 7 | -3.86 |
| 2066 | 3EDV:B | structural protein | 1738 | 1742 | ERFRE | 5 | 98.62 | HHHHH | 7 | -2.64 |
| 2067 | 3LPW:B | structural protein | 154 | 158 | TVKVT | 5 | 91.98 | EESSS | 4 | 0.62 |
| 2068 | 3LR2:B | structural protein | 91 | 95 | STKTS | 5 | 45.86 | HHHHH | 9 | -1.38 |
| 2069 | 3ENU:A | structural protein | 63 | 67 | SLFLS | 5 | 42.80 | EEEEE | 5 | 1.76 |
| 2070 | 2FTX:B | structural protein, protein binding | 202 | 206 | YKTKY | 5 | 57.90 | HHHHH | 7 | -2.22 |
| 2071 | 2FTX:B | structural protein, protein binding | 160 | 164 | LKLKL | 5 | 62.94 | HHHHH | 7 | 0.72 |
| 2072 | 2V51:F | structural protein/contractile protein | 75 | 79 | QLKLQ | 5 | 68.10 | HHHHH | 6 | -0.66 |
| 2073 | 3KNB:A | structural protein/structural protein | 14 | 18 | DISID | 5 | 37.62 | EEEEE | 9 | 0.24 |
| 2074 | 3BFQ:G | structural protein/structural protein | 85 | 89 | QLELQ | 5 | 8.14 | EEEEE | 11 | -0.58 |
| 2075 | 3KNB:A | structural protein/structural protein | 32 | 36 | EPTPE | 5 | 80.94 | ESCCE | 5 | -2.18 |
| 2076 | 3Q64:A | structure genomics, unknown function | 73 | 77 | YTATY | 5 | 25.20 | EEEEE | 9 | -0.44 |
| 2077 | 3Q64:A | structure genomics, unknown function | 142 | 146 | LLDLL | 5 | 38.60 | HHHHH | 9 | 2.34 |
| 2078 | 1MWP:A | sugar binding protein | 99 | 103 | KRGRK | 5 | 95.14 | EGGGE | 16 | -3.44 |
| 2079 | 1MWP:A | sugar binding protein | 42 | 46 | NMHMN | 5 | 14.50 | CEEEC | 16 | -1.28 |
| 2080 | 1OKO:A | sugar binding protein | 25 | 29 | VITIV | 5 | 8.36 | CEEEE | 9 | 3.34 |
| 2081 | 1WW7:A | sugar binding protein | 12 | 16 | SAGAS | 5 | 27.26 | ETTEE | 6 | 0.32 |
| 2082 | 1Y55:X | sugar binding protein | 61 | 65 | TFGFT | 5 | 0.36 | EEEEE | 14 | 0.76 |
| 2083 | 2A6Z:A | sugar binding protein | 48 | 52 | SNQNS | 5 | 73.22 | CSTTC | 4 | -2.42 |
| 2084 | 2OB5:A | sugar binding protein | 92 | 96 | QVEVQ | 5 | 42.54 | HHHHH | 9 | -0.42 |
| 2085 | 2OFC:A | sugar binding protein | 63 | 67 | FTATF | 5 | 0.00 | EEEEE | 10 | 1.20 |
| 2086 | 2OX8:A | sugar binding protein | 699 | 703 | GHGHG | 5 | 50.30 | TTCCC | 10 | -1.52 |
| 2087 | 2QSK:A | sugar binding protein | 13 | 18 | NPGGPN | 6 | 57.47 | CTTSTT | 6 | -1.83 |
| 2088 | 2QSK:A | sugar binding protein | 61 | 66 | NPGGPN | 6 | 57.47 | CTTSTT | 6 | -1.83 |
| 2089 | 2WQ4:B | sugar binding protein | 121 | 125 | ASLSA | 5 | 0.24 | EEEEE | 10 | 1.16 |
| 2090 | 2WQ4:B | sugar binding protein | 96 | 100 | VGSGV | 5 | 40.76 | CCTTC | 4 | 1.36 |
| 2091 | 2XFD:A | sugar binding protein | 60 | 64 | DVQVD | 5 | 22.46 | CEEEE | 11 | -0.42 |
| 2092 | 2XR6:A | sugar binding protein | 333 | 338 | SPLLPS | 6 | 62.92 | CBCCGG | 8 | 0.47 |
| 2093 | 3D02:A | sugar binding protein | 149 | 153 | GGKGG | 5 | 35.78 | TTCEE | 6 | -1.10 |
| 2094 | 3FLP:E | sugar binding protein | 206 | 210 | GVIVG | 5 | 12.24 | SEEEE | 7 | 2.42 |
| 2095 | 3HNX:A | sugar binding protein | 76 | 80 | LRARL | 5 | 28.40 | EEEEE | 11 | 0.08 |
| 2096 | 3P6B:B | sugar binding protein | 134 | 138 | WNNNW | 5 | 65.82 | ESSTT | 6 | -2.46 |
| 2097 | 3SEE:A | sugar binding protein | 165 | 169 | KDADK | 5 | 95.14 | CSTTC | 5 | -2.60 |
| 2098 | 3ZSJ:A | sugar binding protein | 131 | 135 | LITIL | 5 | 25.98 | EEEEE | 9 | 3.18 |
| 2099 | 2VUV:A | sugar-binding protein | 18 | 22 | SAKAS | 5 | 54.36 | EEEEC | 7 | -0.38 |
| 2100 | 1W5Q:A | synthase | 16 | 20 | RRNRR | 5 | 62.48 | TTTTS | 15 | -4.30 |
| 2101 | 1W5Q:A | synthase | 206 | 210 | YASAY | 5 | 12.44 | BCCGG | 15 | 0.04 |
| 2102 | 1OI7:A | synthetase | 77 | 81 | AADAA | 5 | 21.72 | HHHHH | 7 | 0.74 |
| 2103 | 2TPS:A | thiamin biosynthesis | 105 | 109 | GIHIG | 5 | 7.00 | EEEEC | 11 | 1.00 |
| 2104 | 1F0L:B | toxin | 177 | 183 | AMYEYMA | 7 | 22.51 | HHHHHHH | 6 | 0.19 |
| 2105 | 3SEB:A | toxin | 77 | 81 | YKDKY | 5 | 75.92 | HTTSE | 7 | -2.78 |
| 2106 | 3EA6:A | toxin | 98 | 102 | NIWIN | 5 | 43.50 | EEEET | 9 | 0.22 |
| 2107 | 2RDG:A | toxin | 55 | 59 | DKIKD | 5 | 93.34 | HHSCS | 7 | -2.06 |
| 2108 | 1F0L:B | toxin | 212 | 216 | KTKTK | 5 | 56.84 | HHHHH | 7 | -2.62 |
| 2109 | 1F0L:B | toxin | 178 | 182 | MYEYM | 5 | 27.44 | HHHHH | 7 | -0.46 |
| 2110 | 1F0L:B | toxin | 479 | 483 | VGNGV | 5 | 16.90 | EBTTB | 6 | 0.82 |
| 2111 | 1F0L:B | toxin | 282 | 286 | AVNVA | 5 | 5.56 | HHHHH | 7 | 1.70 |
| 2112 | 1I8N:A | toxin | 90 | 94 | NTETN | 5 | 60.50 | ETTTC | 5 | -2.38 |
| 2113 | 1IAZ:A | toxin | 154 | 158 | LGYGL | 5 | 39.08 | EETTE | 5 | 1.10 |
| 2114 | 1M1F:B | toxin | 40 | 44 | PVVVP | 5 | 2.00 | CEEEE | 8 | 1.88 |
| 2115 | 1MC2:A | toxin | 1026 | 1030 | GCNCG | 5 | 9.26 | TTTSS | 10 | 0.14 |
| 2116 | 1NPI:A | toxin | 50 | 54 | WVKVW | 5 | 80.08 | TCCCC | 6 | 0.54 |
| 2117 | 1PP0:A | toxin | 105 | 109 | NTFTN | 5 | 38.52 | HHHTS | 6 | -1.12 |
| 2118 | 1PP0:A | toxin | 148 | 152 | SYFYS | 5 | 29.48 | TEEEE | 6 | -0.28 |
| 2119 | 1PP0:A | toxin | 156 | 160 | TILIT | 5 | 17.44 | EEEEE | 9 | 2.28 |
| 2120 | 1R4P:A | toxin | 172 | 176 | RQIQR | 5 | 33.32 | HHHHH | 11 | -2.30 |
| 2121 | 1R4P:A | toxin | 83 | 87 | NTATN | 5 | 55.68 | ETTTT | 5 | -1.32 |
| 2122 | 1R4P:A | toxin | 217 | 221 | GVRVG | 5 | 5.60 | CEEET | 5 | 0.62 |
| 2123 | 1RC9:A | toxin | 190 | 194 | TNCNT | 5 | 73.36 | TTHHH | 6 | -1.18 |
| 2124 | 1SR4:C | toxin | 74 | 78 | KEYEK | 5 | 102.70 | CTTCT | 6 | -3.22 |
| 2125 | 1SR4:C | toxin | 151 | 155 | GSVSG | 5 | 38.30 | SCCTT | 6 | 0.36 |
| 2126 | 1VCL:A | toxin | 88 | 92 | QRWRQ | 5 | 69.18 | GCEEE | 10 | -3.38 |
| 2127 | 1VCL:A | toxin | 126 | 130 | DGTGD | 5 | 51.00 | SSCSB | 10 | -1.70 |
| 2128 | 1VCL:A | toxin | 164 | 168 | DLCLD | 5 | 28.10 | CCEEE | 10 | 0.62 |
| 2129 | 2A6S:D | toxin | 5 | 10 | WSEESW | 6 | 42.32 | ECHHHH | 9 | -1.73 |
| 2130 | 2AIB:A | toxin | 5 | 10 | ATQQTA | 6 | 67.77 | HHHHHH | 5 | -0.80 |
| 2131 | 2D42:B | toxin | 224 | 228 | TGEGT | 5 | 38.78 | EEEEE | 8 | -1.14 |
| 2132 | 2D42:B | toxin | 45 | 49 | IPDPI | 5 | 56.56 | CSCCE | 5 | 0.46 |
| 2133 | 2QV3:A | toxin | 523 | 527 | GVSVG | 5 | 82.70 | TTCSS | 3 | 1.36 |
| 2134 | 2RCI:A | toxin | 141 | 145 | NTFTN | 5 | 26.84 | HHHSS | 6 | -1.12 |
| 2135 | 2RCI:A | toxin | 54 | 58 | IAQAI | 5 | 44.78 | HHHHH | 9 | 1.82 |
| 2136 | 2RCI:A | toxin | 121 | 125 | VLGLV | 5 | 41.84 | HHCSC | 6 | 3.12 |
| 2137 | 2RDG:A | toxin | 52 | 56 | KDKDK | 5 | 78.12 | THHHH | 4 | -3.74 |
| 2138 | 2RK5:A | toxin | 73 | 77 | DKVKD | 5 | 97.16 | EEEET | 6 | -2.12 |
| 2139 | 2RK5:A | toxin | 45 | 49 | TGVGT | 5 | 52.44 | HHHCS | 6 | 0.40 |
| 2140 | 2SN3:A | toxin | 20 | 24 | GENEG | 5 | 84.02 | BSCHH | 10 | -2.26 |
| 2141 | 3AJ6:B | toxin | 127 | 131 | STQTS | 5 | 51.78 | EECCS | 6 | -1.30 |
| 2142 | 3AJ6:B | toxin | 154 | 158 | LQTQL | 5 | 27.60 | EEETT | 6 | -0.02 |
| 2143 | 3BON:A | toxin | 355 | 360 | VKFFKV | 6 | 73.72 | HHHHTC | 4 | 1.03 |
| 2144 | 3BPQ:A | toxin | 15 | 19 | KEYEK | 5 | 76.84 | HHHHH | 7 | -3.22 |
| 2145 | 3EA6:A | toxin | 94 | 98 | NIPIN | 5 | 24.44 | ECCEE | 5 | 0.08 |
| 2146 | 3EA6:A | toxin | 3 | 7 | DIGID | 5 | 65.42 | CHHHH | 5 | 0.32 |
| 2147 | 3SEB:A | toxin | 75 | 79 | DKYKD | 5 | 66.12 | HHHTT | 2 | -3.22 |
| 2148 | 3SEB:A | toxin | 5 | 9 | DPKPD | 5 | 78.14 | CSSSS | 2 | -2.82 |
| 2149 | 1C4Q:B | toxin | 238 | 242 | SLLLS | 5 | 20.14 | HHHHH | 6 | 1.96 |
| 2150 | 1PP0:A | toxin | 104 | 110 | LNTFTNL | 7 | 33.77 | HHHHTSH | 7 | 0.29 |
| 2151 | 3H87:C | toxin/antitoxin | 57 | 61 | GAVAG | 5 | 25.72 | HHSGG | 7 | 1.40 |
| 2152 | 3PA8:B | toxin/peptide inhibitor | 171 | 175 | KLLLK | 5 | 47.18 | HHHHH | 7 | 0.72 |
| 2153 | 3HPW:A | toxin/toxin repressor | 70 | 74 | SVPVS | 5 | 40.52 | EEEGG | 7 | 1.04 |
| 2154 | 3TGN:A | transcription | 47 | 52 | LSEESL | 6 | 61.13 | HTTCCC | 5 | -0.17 |
| 2155 | 1TH8:A | transcription | 122 | 126 | VESEV | 5 | 72.06 | EEEET | 6 | 0.12 |
| 2156 | 1TH8:A | transcription | 63 | 69 | IVSISVI | 7 | 20.71 | EEEEEEE | 11 | 2.90 |
| 2157 | 1G8E:A | transcription | 45 | 50 | TLAALT | 6 | 39.78 | HHHTCC | 9 | 1.63 |
| 2158 | 2Y2Z:A | transcription | 98 | 104 | AALTLAA | 7 | 23.01 | HHHHHHH | 8 | 2.01 |
| 2159 | 1YMT:A | transcription | 229 | 234 | LQLLQL | 6 | 56.90 | HHHHHH | 5 | 1.37 |
| 2160 | 1GXR:B | transcription | 467 | 471 | IGPGI | 5 | 44.26 | CSTTC | 2 | 1.32 |
| 2161 | 1OW1:A | transcription | 3537 | 3541 | SLPLS | 5 | 63.78 | HSCCC | 5 | 0.88 |
| 2162 | 1P4X:A | transcription | 123 | 127 | QSESQ | 5 | 115.98 | TCCSS | 3 | -2.42 |
| 2163 | 1T4W:A | transcription | 384 | 388 | ENGNE | 5 | 86.80 | TTSCE | 6 | -2.88 |
| 2164 | 1TH8:A | transcription | 13 | 17 | SENES | 5 | 35.28 | TTHHH | 8 | -2.42 |
| 2165 | 1YMT:A | transcription | 374 | 378 | FLILF | 5 | 4.78 | HHHHT | 6 | 3.54 |
| 2166 | 1Z3E:B | transcription | 306 | 310 | LGLGL | 5 | 51.80 | TTCCC | 4 | 2.12 |
| 2167 | 2FL7:A | transcription | 96 | 100 | LKPKL | 5 | 48.54 | SCHHH | 6 | -0.36 |
| 2168 | 2H98:A | transcription | 118 | 122 | EIHIE | 5 | 61.84 | TSEEE | 5 | -0.24 |
| 2169 | 2HIN:A | transcription | 18 | 22 | VGVGV | 5 | 48.46 | HHHTS | 5 | 2.36 |
| 2170 | 2IU1:A | transcription | 395 | 399 | EDEDE | 5 | 0.00 | CCCSC | 6 | -3.50 |
| 2171 | 2IU5:A | transcription | 43 | 47 | FYNYF | 5 | 60.96 | GGGTC | 4 | -0.10 |
| 2172 | 2O7T:A | transcription | 102 | 106 | PALAP | 5 | 36.20 | HHHCC | 4 | 0.84 |
| 2173 | 2O7T:A | transcription | 162 | 166 | TALAT | 5 | 90.28 | GGGCC | 4 | 1.20 |
| 2174 | 2OFY:B | transcription | 68 | 72 | DLSLD | 5 | 56.48 | TCCHH | 9 | -0.04 |
| 2175 | 2VT3:A | transcription | 186 | 190 | HIRIH | 5 | 57.44 | TSEEE | 7 | -0.38 |
| 2176 | 2YVE:B | transcription | 169 | 173 | ILELI | 5 | 50.98 | HHHHC | 5 | 2.62 |
| 2177 | 3CJW:A | transcription | 373 | 377 | VSSSV | 5 | 35.36 | SCHHH | 7 | 1.20 |
| 2178 | 3D3B:A | transcription | 56 | 60 | ATNTA | 5 | 59.52 | HHTHH | 8 | -0.26 |
| 2179 | 3D3B:J | transcription | 44 | 48 | TRSRT | 5 | 101.60 | EETTE | 3 | -2.24 |
| 2180 | 3EIK:A | transcription | 37 | 41 | KLDLK | 5 | 75.74 | CCCHH | 5 | -0.74 |
| 2181 | 3G3Z:A | transcription | 76 | 80 | QEGEQ | 5 | 117.68 | CCCSS | 6 | -2.88 |
| 2182 | 3G3Z:A | transcription | 27 | 31 | LNYNL | 5 | 44.54 | CCHHH | 6 | -0.14 |
| 2183 | 3KV1:A | transcription | 197 | 201 | TDADT | 5 | 61.26 | CBCCC | 7 | -1.32 |
| 2184 | 3KV1:A | transcription | 46 | 50 | LNNNL | 5 | 40.04 | HHHHC | 7 | -0.58 |
| 2185 | 3KV1:A | transcription | 116 | 120 | YAPAY | 5 | 49.90 | CSBSB | 7 | -0.12 |
| 2186 | 3PJP:B | transcription | 1296 | 1300 | LDKDL | 5 | 56.32 | EETTE | 4 | -0.66 |
| 2187 | 3Q7R:B | transcription | 105 | 109 | SLFLS | 5 | 29.30 | HHHHT | 6 | 1.76 |
| 2188 | 3R1F:A | transcription | 19 | 23 | PGRGP | 5 | 46.18 | TTSCC | 5 | -1.70 |
| 2189 | 3RSN:A | transcription | 163 | 167 | LDQDL | 5 | 59.90 | SCCCG | 5 | -0.58 |
| 2190 | 3S8S:A | transcription | 119 | 124 | EVEEVE | 6 | 65.77 | CEEEEE | 6 | -0.93 |
| 2191 | 3T5X:A | transcription | 222 | 226 | FDSDF | 5 | 82.70 | HTTCH | 14 | -0.44 |
| 2192 | 3U02:C | transcription | 246 | 250 | TNQNT | 5 | 48.42 | ESCCC | 6 | -2.38 |
| 2193 | 3U02:C | transcription | 67 | 71 | EKIKE | 5 | 93.82 | GGHHH | 6 | -2.06 |
| 2194 | 3U9Q:A | transcription | 308 | 312 | NLDLN | 5 | 70.10 | GSCHH | 7 | -0.58 |
| 2195 | 3UFE:B | transcription | 59 | 63 | KEPEK | 5 | 65.84 | CHHHH | 5 | -3.28 |
| 2196 | 3V2U:D | transcription | 234 | 238 | PFKFP | 5 | 50.94 | EECCC | 8 | -0.30 |
| 2197 | 3V2U:D | transcription | 100 | 104 | DLPLD | 5 | 39.00 | ECCTT | 8 | -0.20 |
| 2198 | 4A8X:B | transcription | 678 | 682 | EAIAE | 5 | 77.50 | HHHHC | 5 | 0.22 |
| 2199 | 1G8E:A | transcription | 73 | 77 | QTITQ | 5 | 66.48 | HHHHH | 5 | -0.78 |
| 2200 | 1H3L:A | transcription | 69 | 73 | YAKAY | 5 | 28.00 | HHHHH | 8 | -0.58 |
| 2201 | 1P4X:A | transcription | 72 | 76 | KVLVK | 5 | 72.00 | HHHHH | 6 | 0.88 |
| 2202 | 1R29:A | transcription | 94 | 98 | RLNLR | 5 | 91.60 | CCCCC | 6 | -0.98 |
| 2203 | 1R29:A | transcription | 106 | 110 | MATAM | 5 | 40.60 | HHHHH | 6 | 1.34 |
| 2204 | 1R29:A | transcription | 34 | 38 | VVIVV | 5 | 10.58 | EEEEE | 6 | 4.26 |
| 2205 | 1S3J:B | transcription | 12 | 16 | QLSLQ | 5 | 52.54 | HHHHH | 7 | -0.04 |
| 2206 | 1TH8:A | transcription | 119 | 123 | EVIVE | 5 | 55.64 | EEEEE | 9 | 1.18 |
| 2207 | 1TH8:A | transcription | 64 | 68 | VSISV | 5 | 5.96 | EEEEE | 9 | 2.26 |
| 2208 | 1YDL:A | transcription | 49 | 53 | LVNVL | 5 | 64.94 | HHHHH | 5 | 2.50 |
| 2209 | 1ZGZ:A | transcription | 28 | 32 | TVSVT | 5 | 38.16 | EEEEE | 7 | 1.24 |
| 2210 | 2FA5:B | transcription | 82 | 86 | RAVAR | 5 | 63.14 | HHHHH | 9 | -0.24 |
| 2211 | 2FBI:A | transcription | 8 | 12 | LTLTL | 5 | 88.16 | HHHHH | 5 | 2.00 |
| 2212 | 2FBI:A | transcription | 128 | 132 | LLELL | 5 | 95.36 | HHHHH | 5 | 2.34 |
| 2213 | 2HWV:A | transcription | 196 | 200 | TVDVT | 5 | 24.32 | HHHHH | 9 | 0.70 |
| 2214 | 2NML:A | transcription | 87 | 91 | IKEKI | 5 | 38.08 | HHHHH | 9 | -0.46 |
| 2215 | 2OKG:B | transcription | 154 | 158 | EAVAE | 5 | 50.48 | HHHHH | 10 | 0.16 |
| 2216 | 2OKG:B | transcription | 324 | 328 | TVLVT | 5 | 3.50 | EEEEE | 10 | 2.16 |
| 2217 | 2PN0:C | transcription | 61 | 65 | VRFRV | 5 | 33.48 | EEEEE | 10 | 0.44 |
| 2218 | 2PN0:C | transcription | 35 | 39 | LEAEL | 5 | 33.12 | HHHHH | 10 | 0.48 |
| 2219 | 2QSX:B | transcription | 207 | 211 | LKDKL | 5 | 54.10 | HHHHH | 6 | -0.74 |
| 2220 | 2XTC:A | transcription | 49 | 53 | LISIL | 5 | 29.56 | HHHHH | 7 | 3.16 |
| 2221 | 2Y2Z:A | transcription | 36 | 40 | AVKVA | 5 | 32.40 | HHHHH | 8 | 1.62 |
| 2222 | 2Y2Z:A | transcription | 99 | 103 | ALTLA | 5 | 23.42 | HHHHH | 8 | 2.10 |
| 2223 | 3BD1:C | transcription | 3 | 7 | AIDIA | 5 | 22.36 | HHHHH | 6 | 1.82 |
| 2224 | 3D06:A | transcription | 252 | 257 | LTIITL | 6 | 5.45 | EEEEEE | 13 | 2.53 |
| 2225 | 3D3B:A | transcription | 5 | 9 | ARRRA | 5 | 46.38 | HHHHH | 11 | -1.98 |
| 2226 | 3D3B:J | transcription | 5 | 9 | RIRIR | 5 | 51.00 | EEEEE | 9 | -0.90 |
| 2227 | 3G3Z:A | transcription | 125 | 129 | ADLDA | 5 | 35.64 | HHHHH | 10 | 0.08 |
| 2228 | 3LA7:B | transcription | 179 | 183 | IAEAI | 5 | 21.50 | HHHHH | 8 | 1.82 |
| 2229 | 3LFP:A | transcription | 9 | 13 | ARLRA | 5 | 62.12 | HHHHH | 8 | -0.32 |
| 2230 | 3LFP:A | transcription | 29 | 33 | ASASA | 5 | 49.68 | HHHHH | 8 | 0.76 |
| 2231 | 3LYI:A | transcription | 987 | 991 | FLVLF | 5 | 18.58 | EEEEE | 8 | 3.48 |
| 2232 | 3PMT:A | transcription | 575 | 579 | AEVEA | 5 | 28.82 | EEEEE | 7 | 0.16 |
| 2233 | 3TGN:A | transcription | 107 | 113 | EHHHHHE | 7 | 47.63 | HHHHHHH | 13 | -3.29 |
| 2234 | 3U9Q:A | transcription | 450 | 455 | VQLLQV | 6 | 61.78 | HHHHHH | 8 | 1.50 |
| 2235 | 3U9Q:A | transcription | 385 | 389 | AIFIA | 5 | 0.00 | HHHHH | 8 | 3.08 |
| 2236 | 3UFE:B | transcription | 82 | 86 | LIKIL | 5 | 32.74 | HHHHH | 8 | 2.54 |
| 2237 | 3V2U:D | transcription | 324 | 328 | IGTGI | 5 | 36.02 | HHHHH | 5 | 1.50 |
| 2238 | 3V46:A | transcription | 383 | 387 | LEKEL | 5 | 33.82 | HHHHH | 20 | -0.66 |
| 2239 | 4A69:C | transcription | 468 | 472 | LYYYL | 5 | 23.68 | HHHHH | 7 | 0.74 |
| 2240 | 4A8J:F | transcription | 218 | 222 | NLNLN | 5 | 5.02 | EEEEE | 11 | -0.58 |
| 2241 | 2O7T:A | transcription | 161 | 167 | ITALATI | 7 | 103.96 | CGGGCCS | 3 | 2.14 |
| 2242 | 1BGF:A | transcription factor | 84 | 88 | KRIRK | 5 | 99.86 | HHHHH | 7 | -2.46 |
| 2243 | 3HIM:A | transcription regulator | 18 | 24 | AARIRAA | 7 | 44.06 | HHHHHHH | 8 | 0.39 |
| 2244 | 3C8G:D | transcription regulator | 116 | 122 | ISNVNSI | 7 | 25.89 | HTTCHHH | 8 | 0.66 |
| 2245 | 3SQN:B | transcription regulator | 278 | 282 | LASAL | 5 | 26.36 | HHHHH | 8 | 2.08 |
| 2246 | 3SQN:B | transcription regulator | 258 | 262 | FFAFF | 5 | 0.72 | HHHHH | 8 | 2.60 |
| 2247 | 1Y7Y:B | transcription regulator | 61 | 65 | LATAL | 5 | 22.16 | HHHHT | 7 | 2.10 |
| 2248 | 2HKX:B | transcription regulator | 124 | 128 | LLKLL | 5 | 47.56 | HHHHH | 9 | 2.26 |
| 2249 | 2QQB:A | transcription regulator | 116 | 120 | LVKVL | 5 | 64.70 | HHHHS | 5 | 2.42 |
| 2250 | 3C8G:D | transcription regulator | 117 | 121 | SNVNS | 5 | 36.14 | TTCHH | 8 | -0.88 |
| 2251 | 3C8G:D | transcription regulator | 155 | 159 | ALTLA | 5 | 10.74 | HHHHH | 7 | 2.10 |
| 2252 | 3CDL:B | transcription regulator | 83 | 87 | LLELL | 5 | 42.10 | HHHHH | 7 | 2.34 |
| 2253 | 3CTP:A | transcription regulator | 102 | 106 | KEKEK | 5 | 60.64 | HHHHH | 10 | -3.74 |
| 2254 | 3CWR:A | transcription regulator | 194 | 198 | VADAV | 5 | 24.86 | HHHHH | 8 | 1.70 |
| 2255 | 3ELK:B | transcription regulator | 95 | 99 | ALQLA | 5 | 64.38 | HHHHH | 7 | 1.54 |
| 2256 | 3G7R:B | transcription regulator | 149 | 153 | GDGDG | 5 | 67.32 | SSSCS | 4 | -1.64 |
| 2257 | 3G7R:B | transcription regulator | 92 | 96 | RAVAR | 5 | 42.80 | HHHHH | 9 | -0.24 |
| 2258 | 3G7R:B | transcription regulator | 148 | 154 | VGDGDGV | 7 | 62.54 | TSSSCSH | 4 | 0.03 |
| 2259 | 3HIM:A | transcription regulator | 108 | 112 | RASAR | 5 | 41.04 | HHHHH | 9 | -1.24 |
| 2260 | 3HIM:A | transcription regulator | 19 | 23 | ARIRA | 5 | 56.42 | HHHHH | 9 | -0.18 |
| 2261 | 3HIM:A | transcription regulator | 147 | 151 | GATAG | 5 | 55.32 | HHHTT | 6 | 0.42 |
| 2262 | 3HIM:A | transcription regulator | 17 | 25 | AAARIRAAA | 9 | 38.08 | HHHHHHHHH | 9 | 0.70 |
| 2263 | 3KP7:B | transcription regulator | 128 | 132 | KEIEK | 5 | 91.92 | HHHHH | 6 | -2.06 |
| 2264 | 3KP7:B | transcription regulator | 123 | 127 | SDFDS | 5 | 73.40 | TTSCH | 7 | -1.16 |
| 2265 | 3KZ9:D | transcription regulator | 32 | 36 | RGIGR | 5 | 55.84 | HCTTT | 7 | -1.06 |
| 2266 | 3M1E:A | transcription regulator | 39 | 43 | LEEEL | 5 | 65.26 | HHHHH | 9 | -0.58 |
| 2267 | 3N5B:B | transcription regulator | 66 | 70 | NRLRN | 5 | 60.36 | HHHHH | 10 | -2.44 |
| 2268 | 3NRV:D | transcription regulator | 121 | 125 | EEFEE | 5 | 95.64 | TTCCH | 9 | -2.24 |
| 2269 | 3P1X:A | transcription regulator | 580 | 584 | AALAA | 5 | 13.54 | HHHHH | 7 | 2.20 |
| 2270 | 3QAO:A | transcription regulator | 42 | 46 | KDVDK | 5 | 77.42 | HHHHH | 7 | -2.12 |
| 2271 | 3QAO:A | transcription regulator | 98 | 102 | TLDLT | 5 | 66.68 | HHHHH | 7 | 0.54 |
| 2272 | 3KP7:B | transcription regulator | 94 | 99 | LKIIKL | 6 | 80.38 | CCCBEE | 7 | 1.47 |
| 2273 | 3CWR:A | transcription regulator | 193 | 199 | RVADAVR | 7 | 54.47 | HHHHHHH | 7 | -0.07 |
| 2274 | 3E19:C | transcription regulator, metal binding protein | 71 | 75 | RVMVR | 5 | 46.28 | TEEEE | 12 | 0.26 |
| 2275 | 1PK3:C | transcription repression | 28 | 32 | SNDNS | 5 | 70.44 | HHCGG | 6 | -2.42 |
| 2276 | 1PK3:C | transcription repression | 63 | 67 | GLKLG | 5 | 32.98 | CCCHH | 6 | 0.58 |
| 2277 | 3VHV:A | transcription/inhibitor | 745 | 749 | IEPEI | 5 | 80.28 | HCCCC | 4 | 0.08 |
| 2278 | 2VSH:B | transferase | 67 | 73 | KYLPLYK | 7 | 84.63 | HHCGGGG | 7 | -0.63 |
| 2279 | 2IMF:A | transferase | 55 | 61 | DLKVKLD | 7 | 91.30 | GCHHHHH | 6 | -0.43 |
| 2280 | 2WAS:B | transferase | 1879 | 1883 | VAVAV | 5 | 6.20 | EEEEE | 8 | 3.24 |
| 2281 | 2OH1:A | transferase | 73 | 79 | AGALAGA | 7 | 34.17 | TCCEEEE | 5 | 1.46 |
| 2282 | 2OFW:C | transferase | 94 | 100 | GLNKNLG | 7 | 28.36 | TTTTTCC | 9 | -0.59 |
| 2283 | 2IBP:B | transferase | 278 | 283 | KPGGPK | 6 | 102.60 | STTCCC | 2 | -1.97 |
| 2284 | 3NPK:A | transferase | 202 | 206 | GLKLG | 5 | 17.90 | HHHHH | 8 | 0.58 |
| 2285 | 3NPK:A | transferase | 201 | 207 | LGLKLGL | 7 | 18.39 | HHHHHHH | 9 | 1.50 |
| 2286 | 2GJ4:A | transferase | 123 | 127 | EEIEE | 5 | 44.32 | HTTSC | 8 | -1.90 |
| 2287 | 3US6:A | transferase | 29 | 34 | QLQQLQ | 6 | 73.87 | HHHHTC | 8 | -1.07 |
| 2288 | 1FG7:A | transferase | 47 | 52 | LTQQTL | 6 | 92.83 | CCCCCT | 4 | -0.13 |
| 2289 | 1H72:C | transferase | 97 | 102 | SSAASS | 6 | 7.42 | HHHHHH | 12 | 0.07 |
| 2290 | 3HUL:B | transferase | 23 | 29 | LTLYLTL | 7 | 19.77 | EEEEEEE | 10 | 1.79 |
| 2291 | 2POC:D | transferase | 392 | 398 | IRRCRRI | 7 | 29.69 | HTSSSEE | 17 | -0.93 |
| 2292 | 3BYJ:A | transferase | 143 | 149 | FKDSDKF | 7 | 62.14 | CCTTHHH | 4 | -1.43 |
| 2293 | 3NBM:A | transferase | 543 | 547 | KSPSK | 5 | 72.64 | TCHHH | 7 | -2.20 |
| 2294 | 1KJQ:B | transferase | 386 | 392 | GQVKVQG | 7 | 62.97 | HHCEEEC | 8 | -0.47 |
| 2295 | 2Q4H:B | transferase | 177 | 181 | SAGAS | 5 | 5.47 | TTTCS | 10 | 0.32 |
| 2296 | 3DNT:A | transferase | 40 | 46 | PLSLSLP | 7 | 25.21 | CSBTTBC | 6 | 0.94 |
| 2297 | 2P6W:A | transferase | 85 | 91 | PKNKNKP | 7 | 71.56 | GGGTTSC | 11 | -3.13 |
| 2298 | 3AJD:A | transferase | 216 | 222 | VEENEEV | 7 | 42.03 | TTSSHHH | 11 | -1.30 |
| 2299 | 3EVF:A | transferase | 228 | 234 | NVTFTVN | 7 | 48.57 | CHHHHHH | 6 | 0.40 |
| 2300 | 2VUW:A | transferase | 769 | 775 | QIKRKIQ | 7 | 66.70 | HHHHHHH | 15 | -1.47 |
| 2301 | 2IYV:A | transferase | 26 | 30 | LGVGL | 5 | 57.22 | HTCCE | 6 | 2.20 |
| 2302 | 3IJW:B | transferase | 23 | 27 | KLGLK | 5 | 61.82 | HHTCC | 6 | -0.12 |
| 2303 | 3IJW:B | transferase | 36 | 40 | SSLSS | 5 | 39.60 | ECSGG | 6 | 0.12 |
| 2304 | 1B8O:A | transferase | 265 | 270 | KQAAQK | 6 | 65.03 | HHHHHH | 6 | -1.87 |
| 2305 | 1B8O:A | transferase | 43 | 47 | TQAQT | 5 | 75.04 | EEEEE | 6 | -1.32 |
| 2306 | 1BB9:A | transferase | 76 | 80 | KELEK | 5 | 91.28 | CCHHH | 4 | -2.20 |
| 2307 | 1BTE:A | transferase | 48 | 52 | ISGSI | 5 | 93.42 | ETTEE | 2 | 1.40 |
| 2308 | 1BWN:B | transferase | 98 | 102 | FPYPF | 5 | 13.32 | CCEEE | 10 | 0.22 |
| 2309 | 1CXQ:A | transferase | 127 | 131 | TSKST | 5 | 64.00 | HSHHH | 10 | -1.38 |
| 2310 | 1D8D:A | transferase | 155 | 159 | IIAII | 5 | 22.20 | HHHHH | 8 | 3.96 |
| 2311 | 1DQP:B | transferase | 137 | 141 | IQEQI | 5 | 28.60 | HHHHS | 10 | -0.30 |
| 2312 | 1DQP:B | transferase | 72 | 76 | AYLYA | 5 | 0.40 | HHHHH | 10 | 0.96 |
| 2313 | 1EJ0:A | transferase | 193 | 197 | SRARS | 5 | 83.40 | SCTTC | 6 | -1.76 |
| 2314 | 1EJ0:A | transferase | 145 | 149 | ELALE | 5 | 39.42 | HHHHH | 8 | 0.48 |
| 2315 | 1EJ2:A | transferase | 126 | 130 | YRDRY | 5 | 106.40 | STTTS | 2 | -3.02 |
| 2316 | 1EJD:A | transferase | 297 | 301 | APHPA | 5 | 48.86 | CSTTS | 2 | -0.56 |
| 2317 | 1EJD:A | transferase | 323 | 327 | ITETI | 5 | 29.56 | EECCS | 2 | 0.82 |
| 2318 | 1EKQ:A | transferase | 217 | 221 | AAIAA | 5 | 0.30 | HHHHH | 8 | 2.34 |
| 2319 | 1FG7:A | transferase | 134 | 138 | QLDLQ | 5 | 52.06 | CCCHH | 10 | -0.58 |
| 2320 | 1FSG:A | transferase | 10 | 14 | GKGKG | 5 | 64.22 | TBCTT | 6 | -1.80 |
| 2321 | 1G60:B | transferase | 214 | 218 | DLVLD | 5 | 10.96 | CEEEE | 13 | 0.96 |
| 2322 | 1GA8:A | transferase | 46 | 50 | AAVAA | 5 | 23.40 | HHHHH | 8 | 2.28 |
| 2323 | 1H16:A | transferase | 79 | 83 | STITS | 5 | 28.80 | CCSSS | 4 | 0.30 |
| 2324 | 1H72:C | transferase | 6 | 10 | KVRVK | 5 | 55.26 | EEEEE | 7 | -0.78 |
| 2325 | 1H72:C | transferase | 249 | 253 | VKDKV | 5 | 57.00 | HTTTE | 7 | -0.58 |
| 2326 | 1H72:C | transferase | 292 | 298 | EVGKGVE | 7 | 48.97 | CBCCCCE | 7 | -0.47 |
| 2327 | 1H72:C | transferase | 82 | 86 | KITIK | 5 | 44.90 | EEEEE | 7 | 0.10 |
| 2328 | 1H72:C | transferase | 178 | 182 | NISIN | 5 | 71.40 | SSCCC | 7 | 0.24 |
| 2329 | 1H72:C | transferase | 93 | 97 | SGLGS | 5 | 6.28 | SSSCH | 7 | 0.28 |
| 2330 | 1H72:C | transferase | 293 | 297 | VGKGV | 5 | 35.32 | BCCCC | 7 | 0.74 |
| 2331 | 1H72:C | transferase | 45 | 49 | EIIIE | 5 | 56.16 | SEEEE | 7 | 1.30 |
| 2332 | 1HNJ:A | transferase | 304 | 308 | FGGGF | 5 | 12.50 | EETTT | 4 | 0.88 |
| 2333 | 1I1K:C | transferase | 1193 | 1197 | EGAGE | 5 | 15.98 | EESSS | 6 | -1.20 |
| 2334 | 1I1K:C | transferase | 1105 | 1109 | VGMGV | 5 | 58.32 | SCSSS | 6 | 1.90 |
| 2335 | 1I5N:B | transferase | 56 | 60 | TFGFT | 5 | 46.62 | HTTCH | 8 | 0.76 |
| 2336 | 1INL:A | transferase | 99 | 103 | GGDGG | 5 | 4.26 | CTTCH | 7 | -1.02 |
| 2337 | 1INL:A | transferase | 7 | 11 | LEREL | 5 | 76.18 | HCCCC | 7 | -0.78 |
| 2338 | 1KJQ:B | transferase | 387 | 391 | QVKVQ | 5 | 59.60 | HCEEE | 7 | -0.50 |
| 2339 | 1KNQ:B | transferase | 22 | 26 | SAVAS | 5 | 28.06 | HHHHH | 6 | 1.24 |
| 2340 | 1KZL:A | transferase | 158 | 163 | SIMMIS | 6 | 39.48 | EEEECH | 10 | 1.87 |
| 2341 | 1M4I:A | transferase | 30 | 34 | GAFAG | 5 | 66.20 | HHTTT | 6 | 1.12 |
| 2342 | 1MDO:A | transferase | 141 | 145 | ADLDA | 5 | 32.12 | CCHHH | 8 | 0.08 |
| 2343 | 1MDO:A | transferase | 284 | 288 | PLSLP | 5 | 53.26 | ECCCC | 8 | 0.72 |
| 2344 | 1MDO:A | transferase | 267 | 271 | AAIAA | 5 | 30.86 | HHHHH | 8 | 2.34 |
| 2345 | 1MGT:A | transferase | 115 | 119 | LAKAL | 5 | 43.84 | HHHHT | 6 | 1.46 |
| 2346 | 1O50:A | transferase | 35 | 39 | VTRTV | 5 | 29.16 | TCCEE | 6 | 0.50 |
| 2347 | 1O7Q:A | transferase | 244 | 248 | AQLQA | 5 | 6.52 | EEECT | 23 | 0.08 |
| 2348 | 1O9G:A | transferase | 108 | 112 | RELER | 5 | 47.04 | HHHHH | 12 | -2.44 |
| 2349 | 1O9G:A | transferase | 48 | 52 | PGDGP | 5 | 87.10 | SCCSC | 8 | -1.50 |
| 2350 | 1O9G:A | transferase | 134 | 138 | LRERL | 5 | 51.86 | HHHHH | 12 | -0.98 |
| 2351 | 1O9G:A | transferase | 42 | 46 | RALAR | 5 | 47.36 | HHHHT | 8 | -0.32 |
| 2352 | 1O9G:A | transferase | 127 | 131 | AAQAA | 5 | 16.00 | HHHHH | 12 | 0.74 |
| 2353 | 1O9G:A | transferase | 200 | 204 | LASAL | 5 | 24.90 | HHHHS | 8 | 2.08 |
| 2354 | 1OD6:A | transferase | 154 | 158 | LKAKL | 5 | 62.86 | HHHHT | 7 | 0.32 |
| 2355 | 1OMZ:A | transferase | 80 | 84 | LLRLL | 5 | 34.08 | HHHHH | 8 | 2.14 |
| 2356 | 1P5Z:B | transferase | 251 | 255 | EKVKE | 5 | 92.80 | HHHHH | 7 | -2.12 |
| 2357 | 1P5Z:B | transferase | 51 | 55 | VPEPV | 5 | 33.64 | ECCCH | 8 | 0.34 |
| 2358 | 1QGQ:A | transferase | 170 | 174 | EKVKE | 5 | 84.88 | HHHHH | 7 | -2.12 |
| 2359 | 1QSA:A | transferase | 331 | 335 | ERGRE | 5 | 125.64 | HTTCH | 7 | -3.28 |
| 2360 | 1QSA:A | transferase | 409 | 413 | KSKSK | 5 | 89.80 | TTCCH | 7 | -2.66 |
| 2361 | 1QSA:A | transferase | 535 | 539 | QYVYQ | 5 | 35.56 | HHHHH | 8 | -1.08 |
| 2362 | 1QSA:A | transferase | 37 | 41 | LYPYL | 5 | 17.22 | THHHH | 7 | 0.68 |
| 2363 | 1QSA:A | transferase | 187 | 191 | IASAI | 5 | 29.26 | HHHHH | 8 | 2.36 |
| 2364 | 1QSA:A | transferase | 172 | 176 | LVTVL | 5 | 30.54 | HHHHH | 8 | 3.06 |
| 2365 | 1R45:D | transferase | 95 | 99 | GNENG | 5 | 67.92 | TCTTS | 6 | -2.26 |
| 2366 | 1R7A:B | transferase | 444 | 448 | TDDDT | 5 | 67.74 | EETTT | 5 | -2.38 |
| 2367 | 1R7A:B | transferase | 48 | 52 | GADAG | 5 | 23.92 | SSSTT | 5 | -0.14 |
| 2368 | 1R7A:B | transferase | 228 | 232 | EILIE | 5 | 16.56 | EEEEC | 5 | 1.16 |
| 2369 | 1R7A:B | transferase | 381 | 385 | GALAG | 5 | 2.40 | HHTTC | 5 | 1.32 |
| 2370 | 1T82:D | transferase | 69 | 73 | LQQQL | 5 | 47.04 | HHHHH | 8 | -0.58 |
| 2371 | 1TC1:B | transferase | 18 | 22 | IRTRI | 5 | 53.06 | HHHHH | 10 | -0.14 |
| 2372 | 1TOJ:A | transferase | 331 | 335 | QRMRQ | 5 | 66.58 | HHHHH | 10 | -2.82 |
| 2373 | 1TU7:A | transferase | 135 | 139 | RGNGR | 5 | 77.50 | TGGGS | 5 | -2.66 |
| 2374 | 1TZD:B | transferase | 269 | 273 | LEEEL | 5 | 48.38 | CHHHH | 7 | -0.58 |
| 2375 | 1TZD:B | transferase | 449 | 453 | LIGIL | 5 | 11.90 | HHHHH | 9 | 3.24 |
| 2376 | 1UNQ:A | transferase | 94 | 98 | EEREE | 5 | 80.48 | HHHHH | 9 | -3.70 |
| 2377 | 1UNQ:A | transferase | 43 | 47 | QDVDQ | 5 | 108.96 | CSHHH | 4 | -1.96 |
| 2378 | 1VGW:E | transferase | 137 | 141 | AVPVA | 5 | 35.64 | EEECC | 7 | 2.08 |
| 2379 | 1VHT:C | transferase | 30 | 34 | IDADI | 5 | 47.72 | EEHHH | 7 | 0.76 |
| 2380 | 1W66:A | transferase | -1 | 3 | GAMAG | 5 | 0.00 | CTTSS | 5 | 0.94 |
| 2381 | 1W66:A | transferase | 203 | 207 | VAAAV | 5 | 16.36 | HHHHH | 9 | 2.76 |
| 2382 | 1W98:B | transferase | 338 | 342 | ADEDA | 5 | 46.56 | CGGGG | 6 | -1.38 |
| 2383 | 1W98:B | transferase | 258 | 262 | QIFIQ | 5 | 39.70 | HHHHH | 7 | 0.96 |
| 2384 | 1WWC:A | transferase | 314 | 318 | ELRLE | 5 | 141.42 | CCCCE | 4 | -0.78 |
| 2385 | 1ZRH:A | transferase | 212 | 216 | NFYFN | 5 | 45.42 | GEEEE | 9 | -0.54 |
| 2386 | 2A0J:A | transferase | 99 | 103 | LIFIL | 5 | 4.16 | EEEEE | 11 | 3.88 |
| 2387 | 2AML:B | transferase | 196 | 200 | AETEA | 5 | 42.66 | HHHHH | 10 | -0.82 |
| 2388 | 2B5G:A | transferase | 17 | 21 | ILRLI | 5 | 15.16 | HHHHH | 9 | 2.42 |
| 2389 | 2BU3:B | transferase | 137 | 141 | VKVKV | 5 | 71.80 | CEEEE | 6 | 0.96 |
| 2390 | 2BUE:A | transferase | 124 | 128 | LGKGL | 5 | 65.54 | TTSSH | 4 | 0.58 |
| 2391 | 2DY0:B | transferase | 67 | 71 | GFLFG | 5 | 0.72 | THHHH | 7 | 1.72 |
| 2392 | 2F6U:B | transferase | 2073 | 2077 | YDVDY | 5 | 60.16 | CCSSE | 6 | -1.08 |
| 2393 | 2FFU:A | transferase | 414 | 418 | RLELR | 5 | 49.04 | HHHHH | 10 | -0.98 |
| 2394 | 2FFU:A | transferase | 325 | 329 | DMMMD | 5 | 46.06 | CTTCC | 7 | -0.26 |
| 2395 | 2G3A:A | transferase | 85 | 89 | AEEEA | 5 | 40.28 | HHHHH | 9 | -1.38 |
| 2396 | 2G9Z:B | transferase | 185 | 189 | DVVVD | 5 | 87.76 | CCCCC | 5 | 1.12 |
| 2397 | 2GB4:B | transferase | 24 | 28 | WKEKW | 5 | 61.14 | HHHHH | 6 | -2.62 |
| 2398 | 2GB4:B | transferase | 176 | 180 | LVAVL | 5 | 8.24 | EEEEE | 6 | 3.56 |
| 2399 | 2GJ4:A | transferase | 120 | 124 | EELEE | 5 | 64.12 | HHHHT | 6 | -2.04 |
| 2400 | 2GVG:A | transferase | 368 | 372 | MKQKM | 5 | 73.62 | HHHTT | 7 | -1.50 |
| 2401 | 2GVG:A | transferase | 271 | 275 | SVPVS | 5 | 35.62 | SSCEE | 7 | 1.04 |
| 2402 | 2GVG:A | transferase | 204 | 208 | AGIGA | 5 | 3.30 | HHHHH | 7 | 1.46 |
| 2403 | 2H00:C | transferase | 69 | 73 | GIDIG | 5 | 11.08 | EEEES | 9 | 0.94 |
| 2404 | 2H00:C | transferase | 18 | 22 | LTCTL | 5 | 46.50 | HHHHH | 11 | 1.74 |
| 2405 | 2HCR:B | transferase | 24 | 28 | GLELG | 5 | 63.20 | TCCCC | 5 | 0.66 |
| 2406 | 2HF1:A | transferase | 21 | 25 | DKSKD | 5 | 70.28 | ETTTT | 4 | -3.12 |
| 2407 | 2HY7:A | transferase | 202 | 206 | VGHGV | 5 | 10.98 | CCCCB | 8 | 0.88 |
| 2408 | 2I9D:C | transferase | 38 | 42 | ARQRA | 5 | 66.36 | HHHHH | 10 | -1.78 |
| 2409 | 2IBP:B | transferase | 114 | 120 | EAAVAAE | 7 | 3.31 | HHHHHHH | 7 | 0.63 |
| 2410 | 2IBP:B | transferase | 67 | 71 | YLILY | 5 | 18.62 | HHHHH | 7 | 1.90 |
| 2411 | 2IBP:B | transferase | 115 | 119 | AAVAA | 5 | 0.36 | HHHHH | 7 | 2.28 |
| 2412 | 2IBP:B | transferase | 239 | 243 | AAIAA | 5 | 0.40 | HHHHH | 7 | 2.34 |
| 2413 | 2IBP:B | transferase | 322 | 326 | IASAI | 5 | 10.82 | HHHHH | 7 | 2.36 |
| 2414 | 2IDO:D | transferase | 62 | 66 | QLSLQ | 5 | 84.88 | HHHTT | 4 | -0.04 |
| 2415 | 2IFT:A | transferase | 120 | 124 | PQNQP | 5 | 60.36 | CCSSC | 5 | -2.74 |
| 2416 | 2IMF:A | transferase | 4 | 8 | DFYFD | 5 | 5.66 | EEEEC | 14 | -0.54 |
| 2417 | 2IMF:A | transferase | 56 | 60 | LKVKL | 5 | 92.22 | CHHHH | 14 | 0.80 |
| 2418 | 2IYV:A | transferase | 22 | 26 | LAKAL | 5 | 59.16 | HHHHH | 6 | 1.46 |
| 2419 | 2NPT:A | transferase | 83 | 87 | SYYYS | 5 | 49.78 | HHHHH | 9 | -1.10 |
| 2420 | 2NPT:A | transferase | 55 | 59 | ATTTA | 5 | 50.78 | SCCSE | 4 | 0.30 |
| 2421 | 2OEG:A | transferase | 135 | 139 | TSAST | 5 | 22.42 | HHHHH | 9 | -0.24 |
| 2422 | 2OFW:C | transferase | 95 | 99 | LNKNL | 5 | 36.30 | TTTTC | 8 | -0.66 |
| 2423 | 2OH1:A | transferase | 74 | 78 | GALAG | 5 | 22.66 | CCEEE | 7 | 1.32 |
| 2424 | 2ORD:B | transferase | 293 | 297 | EEVEE | 5 | 67.18 | HHHHH | 10 | -1.96 |
| 2425 | 2ORD:B | transferase | 101 | 105 | AIKIA | 5 | 1.68 | HHHHH | 10 | 1.74 |
| 2426 | 2P02:A | transferase | 205 | 209 | QVTVQ | 5 | 22.76 | EEEEE | 5 | 0.14 |
| 2427 | 2P02:A | transferase | 316 | 320 | AAYAA | 5 | 1.76 | HHHHH | 5 | 1.18 |
| 2428 | 2P02:A | transferase | 39 | 43 | TFLFT | 5 | 64.86 | EEEEE | 5 | 1.60 |
| 2429 | 2P6W:A | transferase | 86 | 90 | KNKNK | 5 | 77.50 | GGTTS | 9 | -3.74 |
| 2430 | 2P6W:A | transferase | 155 | 159 | VYEYV | 5 | 45.88 | HHHTH | 9 | 0.46 |
| 2431 | 2PC1:A | transferase | 20 | 24 | EARAE | 5 | 57.36 | HHHHH | 12 | -1.58 |
| 2432 | 2PKF:B | transferase | 282 | 286 | LVAVL | 5 | 11.24 | HHHHH | 7 | 3.56 |
| 2433 | 2POC:D | transferase | 393 | 397 | RRCRR | 5 | 41.32 | TSSSE | 15 | -3.10 |
| 2434 | 2Q4H:B | transferase | 81 | 85 | PDHDP | 5 | 12.44 | STTCS | 6 | -2.68 |
| 2435 | 2Q4H:B | transferase | 183 | 187 | SHSHS | 5 | 2.17 | CSCEE | 6 | -1.76 |
| 2436 | 2Q4H:B | transferase | 175 | 179 | GASAG | 5 | 5.26 | STTTT | 6 | 0.40 |
| 2437 | 2Q5R:C | transferase | 3 | 7 | LTLTL | 5 | 1.02 | EEEES | 10 | 2.00 |
| 2438 | 2Q7D:B | transferase | 17 | 21 | KKIKK | 5 | 94.22 | HHHHH | 7 | -2.22 |
| 2439 | 2QOL:A | transferase | 751 | 755 | NILIN | 5 | 16.46 | GEEEC | 14 | 1.16 |
| 2440 | 2QRD:C | transferase | 482 | 486 | VPKPV | 5 | 75.44 | CCCCB | 5 | 0.26 |
| 2441 | 2R4G:A | transferase | 480 | 484 | EKHKE | 5 | 110.32 | ESSST | 5 | -3.60 |
| 2442 | 2R8O:A | transferase | 314 | 318 | YAKAY | 5 | 63.54 | HHHHC | 7 | -0.58 |
| 2443 | 2UV4:A | transferase | 293 | 297 | VEAEV | 5 | 92.64 | HHHTC | 4 | 0.64 |
| 2444 | 2V8Q:B | transferase | 259 | 263 | YKKKY | 5 | 81.46 | ETTEE | 6 | -2.86 |
| 2445 | 2VDJ:A | transferase | 144 | 148 | GAQAG | 5 | 0.54 | HHHHH | 8 | -0.14 |
| 2446 | 2VDU:D | transferase | 311 | 316 | SKIIKS | 6 | 28.92 | EEEEEC | 6 | -0.07 |
| 2447 | 2VDU:D | transferase | 101 | 105 | IYSYI | 5 | 62.22 | CCCCE | 6 | 1.12 |
| 2448 | 2VDU:D | transferase | 154 | 158 | AISIA | 5 | 13.18 | EEEEC | 6 | 2.36 |
| 2449 | 2VGO:A | transferase | 294 | 298 | HTETH | 5 | 72.18 | HHHHH | 10 | -2.26 |
| 2450 | 2VGO:A | transferase | 200 | 204 | LADAL | 5 | 8.24 | HHHHH | 10 | 1.54 |
| 2451 | 2VSH:B | transferase | 68 | 72 | YLPLY | 5 | 70.12 | HCGGG | 6 | 0.68 |
| 2452 | 2VUW:A | transferase | 770 | 774 | IKRKI | 5 | 61.30 | HHHHH | 15 | -0.66 |
| 2453 | 2W4J:A | transferase | 101 | 105 | LFDFL | 5 | 45.18 | HHHHH | 5 | 1.94 |
| 2454 | 2WAS:B | transferase | 1878 | 1882 | AVAVA | 5 | 0.36 | EEEEE | 9 | 2.76 |
| 2455 | 2X61:A | transferase | 95 | 99 | FYDYF | 5 | 67.34 | HHHHS | 7 | -0.10 |
| 2456 | 2XZZ:A | transferase | 760 | 764 | RPGPR | 5 | 103.98 | SCSSC | 3 | -2.52 |
| 2457 | 2YC3:A | transferase | 138 | 142 | EEYEE | 5 | 102.78 | HTTTT | 5 | -3.06 |
| 2458 | 2Z1E:A | transferase | 275 | 279 | VVMVV | 5 | 0.28 | CEEEE | 9 | 3.74 |
| 2459 | 2ZPT:X | transferase | 181 | 185 | EKRKE | 5 | 99.26 | HHTTT | 4 | -3.86 |
| 2460 | 2ZPT:X | transferase | 57 | 61 | ILDLI | 5 | 2.60 | HHHHH | 10 | 2.62 |
| 2461 | 3A1B:A | transferase | 475 | 479 | LRERL | 5 | 61.06 | HHHHH | 11 | -0.98 |
| 2462 | 3A1B:A | transferase | 571 | 575 | AAQAA | 5 | 37.60 | HHHHH | 11 | 0.74 |
| 2463 | 3AAY:A | transferase | 39 | 43 | IAGAI | 5 | 15.16 | STTCE | 6 | 2.44 |
| 2464 | 3AJD:A | transferase | 217 | 221 | EENEE | 5 | 34.74 | TSSHH | 12 | -3.50 |
| 2465 | 3AJD:A | transferase | 118 | 122 | KTRTK | 5 | 94.96 | HHHHH | 7 | -2.74 |
| 2466 | 3AJD:A | transferase | 195 | 199 | DIGID | 5 | 17.74 | HHHHH | 7 | 0.32 |
| 2467 | 3ALN:C | transferase | 336 | 340 | FSPSF | 5 | 36.68 | CCHHH | 8 | 0.48 |
| 2468 | 3ALN:C | transferase | 374 | 378 | AVEVA | 5 | 46.92 | CCCTH | 8 | 1.70 |
| 2469 | 3AOW:C | transferase | 185 | 189 | EKLKE | 5 | 79.10 | HHHHH | 9 | -2.20 |
| 2470 | 3B33:A | transferase | 47 | 51 | QSLSQ | 5 | 56.60 | CBHHH | 9 | -0.96 |
| 2471 | 3BBD:A | transferase | 19 | 23 | IKNKI | 5 | 75.20 | GGGGC | 4 | -0.46 |
| 2472 | 3BF5:A | transferase | 234 | 238 | YLALY | 5 | 25.82 | HHHHH | 7 | 1.36 |
| 2473 | 3BPU:A | transferase | 689 | 694 | VNKKNV | 6 | 76.05 | ETTEEC | 6 | -1.07 |
| 2474 | 3BY9:A | transferase | 148 | 152 | GSTSG | 5 | 31.58 | CTTTC | 4 | -0.62 |
| 2475 | 3BY9:A | transferase | 234 | 238 | SPIPS | 5 | 50.02 | CCCCB | 4 | -0.06 |
| 2476 | 3BYJ:A | transferase | 144 | 148 | KDSDK | 5 | 61.80 | CTTHH | 5 | -3.12 |
| 2477 | 3C3Y:A | transferase | 65 | 69 | VLKLV | 5 | 27.62 | HHHHT | 6 | 2.42 |
| 2478 | 3C6K:C | transferase | 201 | 205 | GGDGG | 5 | 15.22 | CTTCH | 6 | -1.02 |
| 2479 | 3CI3:A | transferase | 128 | 132 | RTITR | 5 | 45.28 | HHHHH | 8 | -1.18 |
| 2480 | 3CI3:A | transferase | 120 | 124 | LASAL | 5 | 9.00 | HHHHH | 8 | 2.08 |
| 2481 | 3CZQ:A | transferase | 58 | 62 | EEYEE | 5 | 92.14 | HHHHH | 6 | -3.06 |
| 2482 | 3CZQ:A | transferase | 243 | 247 | ETHTE | 5 | 68.70 | HHCCS | 8 | -2.32 |
| 2483 | 3D3S:D | transferase | 30 | 34 | LYAYL | 5 | 25.14 | HHHHH | 8 | 1.36 |
| 2484 | 3DA8:B | transferase | 20 | 24 | SGTGS | 5 | 39.44 | CSCCH | 9 | -0.62 |
| 2485 | 3DA8:B | transferase | 83 | 87 | AATAA | 5 | 36.74 | HHHHT | 9 | 1.30 |
| 2486 | 3DA8:B | transferase | 189 | 193 | AAVAA | 5 | 21.84 | HHHHH | 8 | 2.28 |
| 2487 | 3DNS:B | transferase | 26 | 30 | GITIG | 5 | 43.46 | SCEEE | 8 | 1.50 |
| 2488 | 3DNT:A | transferase | 41 | 45 | LSLSL | 5 | 18.70 | SBTTB | 5 | 1.96 |
| 2489 | 3DRA:A | transferase | 28 | 32 | YDEDY | 5 | 64.74 | CCHHH | 8 | -2.62 |
| 2490 | 3E8M:A | transferase | 120 | 124 | PASAP | 5 | 20.64 | CTTSC | 17 | -0.08 |
| 2491 | 3EPZ:B | transferase | 479 | 483 | FSTSF | 5 | 44.28 | EECSS | 7 | 0.66 |
| 2492 | 3EVF:A | transferase | 242 | 246 | RRMRR | 5 | 103.42 | HHHHS | 6 | -3.22 |
| 2493 | 3EVF:A | transferase | 229 | 233 | VTFTV | 5 | 41.74 | HHHHH | 7 | 1.96 |
| 2494 | 3EVZ:A | transferase | 45 | 49 | KAIAK | 5 | 62.36 | HHHHH | 9 | 0.06 |
| 2495 | 3EYE:A | transferase | 35 | 40 | VVDDVV | 6 | 26.90 | EECHHH | 10 | 1.63 |
| 2496 | 3F0H:A | transferase | 159 | 163 | VCDCV | 5 | 14.42 | EEECT | 9 | 1.98 |
| 2497 | 3FOT:A | transferase | 211 | 215 | RSDSR | 5 | 77.16 | CCCGG | 10 | -2.82 |
| 2498 | 3FOT:A | transferase | 27 | 31 | RQVQR | 5 | 48.22 | CEEEE | 10 | -2.36 |
| 2499 | 3FRH:A | transferase | 175 | 179 | LLPLL | 5 | 16.38 | CHHHH | 7 | 2.72 |
| 2500 | 3FTT:A | transferase | 80 | 84 | NVYVN | 5 | 35.40 | SEEEC | 9 | 0.02 |
| 2501 | 3GJU:A | transferase | 309 | 313 | QVLVQ | 5 | 55.68 | HHHHH | 7 | 1.04 |
| 2502 | 3GJU:A | transferase | 71 | 75 | IADAI | 5 | 37.00 | HHHHH | 7 | 1.82 |
| 2503 | 3GSZ:B | transferase | 109 | 113 | RSLSR | 5 | 94.14 | HTTCH | 5 | -1.36 |
| 2504 | 3GSZ:B | transferase | 439 | 443 | LNQNL | 5 | 65.70 | TTSCE | 5 | -0.58 |
| 2505 | 3GSZ:B | transferase | 456 | 460 | PLDLP | 5 | 12.86 | GGGHH | 5 | 0.18 |
| 2506 | 3GSZ:B | transferase | 26 | 30 | LSNSL | 5 | 42.46 | HHHTT | 5 | 0.50 |
| 2507 | 3H4T:A | transferase | 58 | 62 | RAGAR | 5 | 60.30 | SGGGS | 5 | -1.16 |
| 2508 | 3H4T:A | transferase | 346 | 350 | LATAL | 5 | 32.22 | HHHHT | 5 | 2.10 |
| 2509 | 3H4T:A | transferase | 535 | 539 | LAAAL | 5 | 46.42 | HHHHH | 3 | 2.60 |
| 2510 | 3H5Z:A | transferase | 23 | 27 | TEDET | 5 | 64.26 | SHHHH | 7 | -2.38 |
| 2511 | 3H5Z:A | transferase | 409 | 413 | PKIKP | 5 | 48.22 | CCCCG | 7 | -1.30 |
| 2512 | 3H5Z:A | transferase | 18 | 22 | QPVPQ | 5 | 25.42 | SSSCC | 7 | -1.20 |
| 2513 | 3H5Z:A | transferase | 17 | 23 | TQPVPQT | 7 | 40.44 | GSSSCCS | 7 | -1.06 |
| 2514 | 3HM2:H | transferase | 121 | 125 | VANAV | 5 | 15.84 | EEEEC | 10 | 1.70 |
| 2515 | 3HUL:B | transferase | 60 | 64 | ALNLA | 5 | 45.10 | HHHHC | 6 | 1.54 |
| 2516 | 3HUL:B | transferase | 125 | 129 | VAPAV | 5 | 3.72 | HHHHH | 9 | 2.08 |
| 2517 | 3I1A:B | transferase | 1265 | 1269 | EGYGE | 5 | 51.80 | HHHCC | 9 | -1.82 |
| 2518 | 3IP0:A | transferase | 47 | 51 | PQDQP | 5 | 70.96 | CSSSC | 11 | -2.74 |
| 2519 | 3IU0:A | transferase | 24 | 28 | ANINA | 5 | 38.34 | HHHHH | 9 | 0.22 |
| 2520 | 3JUD:A | transferase | 63 | 67 | VEGEV | 5 | 10.30 | CEEEE | 10 | 0.20 |
| 2521 | 3JWI:A | transferase | 61 | 65 | VSYSV | 5 | 62.94 | SCHHH | 8 | 1.10 |
| 2522 | 3JWI:A | transferase | 175 | 179 | AVKVA | 5 | 16.92 | HHHHH | 6 | 1.62 |
| 2523 | 3JWI:A | transferase | 43 | 47 | LLSLL | 5 | 17.30 | HHHHH | 6 | 2.88 |
| 2524 | 3JYB:B | transferase | 91 | 95 | LQVQL | 5 | 24.42 | EEEEE | 7 | 0.96 |
| 2525 | 3K94:A | transferase | 209 | 213 | SSDSS | 5 | 67.00 | ECCTT | 6 | -1.34 |
| 2526 | 3KHF:B | transferase | 1011 | 1015 | VLGLV | 5 | 44.56 | CTTCC | 6 | 3.12 |
| 2527 | 3KKZ:B | transferase | 81 | 85 | FIDIF | 5 | 33.14 | HHHHH | 7 | 2.22 |
| 2528 | 3KTA:B | transferase | 1080 | 1084 | AFVFA | 5 | 0.80 | HHHHH | 8 | 2.68 |
| 2529 | 3LED:B | transferase | 32 | 36 | ANFNA | 5 | 60.38 | HHHHH | 8 | -0.12 |
| 2530 | 3LED:B | transferase | 76 | 80 | VDPDV | 5 | 60.32 | GCTTT | 2 | -0.04 |
| 2531 | 3LFJ:B | transferase | 56 | 60 | VKVKV | 5 | 42.62 | CCEEE | 5 | 0.96 |
| 2532 | 3LKM:A | transferase | 680 | 685 | KKPPKK | 6 | 89.45 | TCCSSC | 5 | -3.13 |
| 2533 | 3LLT:A | transferase | 608 | 613 | INNNNI | 6 | 43.10 | TTGGGB | 7 | -0.83 |
| 2534 | 3LLT:A | transferase | 696 | 701 | TVRRVT | 6 | 83.65 | EEECTT | 7 | -0.33 |
| 2535 | 3LRT:A | transferase | 91 | 95 | RQHQR | 5 | 53.04 | TCCSC | 10 | -3.84 |
| 2536 | 3LRT:A | transferase | 198 | 202 | NVDVN | 5 | 70.84 | CCCCT | 10 | -0.42 |
| 2537 | 3LRT:A | transferase | 260 | 264 | VTDTV | 5 | 6.56 | EESSS | 10 | 0.70 |
| 2538 | 3LW6:A | transferase | 83 | 87 | FRDRF | 5 | 71.54 | ESSCH | 9 | -1.38 |
| 2539 | 3LW6:A | transferase | 151 | 155 | LNDNL | 5 | 59.86 | CCTTS | 9 | -0.58 |
| 2540 | 3M0F:A | transferase | 141 | 145 | LEQEL | 5 | 38.52 | HHHHH | 13 | -0.58 |
| 2541 | 3ME5:A | transferase | 155 | 159 | EAAAE | 5 | 62.04 | HHHHH | 5 | -0.32 |
| 2542 | 3MK6:C | transferase | 37 | 41 | EEQEE | 5 | 72.84 | HHHHH | 8 | -3.50 |
| 2543 | 3MK6:C | transferase | 334 | 338 | LAYAL | 5 | 10.78 | HHHHH | 8 | 1.98 |
| 2544 | 3MQ2:A | transferase | 67 | 71 | KAAAK | 5 | 68.04 | HHTSC | 9 | -0.48 |
| 2545 | 3MQD:A | transferase | 284 | 288 | STVTS | 5 | 79.82 | TTCCS | 7 | 0.24 |
| 2546 | 3MQD:A | transferase | 139 | 143 | TASAT | 5 | 8.66 | HHHHH | 8 | 0.28 |
| 2547 | 3MSE:B | transferase | 141 | 145 | NDIDN | 5 | 60.72 | HHHHH | 9 | -1.90 |
| 2548 | 3MSE:B | transferase | 20 | 24 | NIIIN | 5 | 33.02 | HHHHH | 9 | 1.30 |
| 2549 | 3MTS:C | transferase | 93 | 97 | LEREL | 5 | 68.80 | HHHHH | 8 | -0.78 |
| 2550 | 3NDI:A | transferase | 373 | 377 | DPYPD | 5 | 66.34 | SSCCS | 8 | -2.30 |
| 2551 | 3NDI:A | transferase | 80 | 84 | SSGSS | 5 | 30.70 | GGGCH | 8 | -0.72 |
| 2552 | 3NPK:A | transferase | 252 | 257 | ENLLNE | 6 | 66.45 | HHHHHH | 9 | -1.07 |
| 2553 | 3NPK:A | transferase | 200 | 204 | KLGLK | 5 | 46.60 | HHHHH | 9 | -0.12 |
| 2554 | 3NVS:A | transferase | 135 | 139 | QAGAQ | 5 | 49.70 | HTTCE | 6 | -0.76 |
| 2555 | 3NVS:A | transferase | 102 | 106 | LAAAL | 5 | 0.46 | HHHHT | 6 | 2.60 |
| 2556 | 3NVS:A | transferase | 28 | 32 | ALLLA | 5 | 0.32 | HHHHH | 7 | 3.00 |
| 2557 | 3OAB:C | transferase | 237 | 241 | ELALE | 5 | 67.70 | HHHHH | 6 | 0.48 |
| 2558 | 3ORK:A | transferase | 253 | 257 | KALAK | 5 | 24.18 | HHTCS | 8 | -0.08 |
| 2559 | 3ORK:A | transferase | 245 | 249 | ADLDA | 5 | 25.90 | HHHHH | 7 | 0.08 |
| 2560 | 3PB6:X | transferase | 197 | 201 | LAQAL | 5 | 14.22 | HHHHT | 8 | 1.54 |
| 2561 | 3PB6:X | transferase | 267 | 271 | LLDLL | 5 | 3.46 | EEESC | 8 | 2.34 |
| 2562 | 3PPL:B | transferase | 235 | 239 | VIDIV | 5 | 24.04 | CCCHH | 6 | 2.78 |
| 2563 | 3QB8:B | transferase | 172 | 176 | GYQYG | 5 | 64.30 | TCCBT | 7 | -1.38 |
| 2564 | 3QHP:B | transferase | 299 | 303 | IVPVI | 5 | 0.14 | CCEEE | 8 | 3.16 |
| 2565 | 3QUV:A | transferase | 152 | 156 | VLRLV | 5 | 16.78 | HHTTS | 9 | 2.30 |
| 2566 | 3RPD:B | transferase | 23 | 27 | LPKPL | 5 | 21.32 | CCCCT | 9 | 0.10 |
| 2567 | 3SE2:A | transferase | 1690 | 1694 | DTVTD | 5 | 9.34 | SEEES | 12 | -0.84 |
| 2568 | 3TQE:A | transferase | 196 | 200 | PVSVP | 5 | 56.40 | SCSCC | 3 | 0.88 |
| 2569 | 3UJC:A | transferase | 228 | 232 | FLKLF | 5 | 70.42 | HHHHS | 5 | 1.86 |
| 2570 | 3US6:A | transferase | 40 | 44 | EFVFE | 5 | 65.20 | THHHH | 6 | 0.56 |
| 2571 | 3V8H:A | transferase | 84 | 88 | ANENA | 5 | 63.10 | HHTCH | 10 | -1.38 |
| 2572 | 3V8H:A | transferase | 120 | 124 | DAHAD | 5 | 73.68 | ETTCH | 10 | -1.32 |
| 2573 | 3VAA:B | transferase | 57 | 61 | RELER | 5 | 15.62 | HHHHH | 12 | -2.44 |
| 2574 | 3VAA:B | transferase | 154 | 158 | DELED | 5 | 62.68 | CCCSS | 8 | -2.04 |
| 2575 | 3VBL:E | transferase | 141 | 145 | GVAVG | 5 | 2.16 | TCEEC | 12 | 1.88 |
| 2576 | 4ADU:B | transferase | 203 | 207 | RKLKR | 5 | 106.98 | HHHTS | 9 | -2.60 |
| 2577 | 4ADU:B | transferase | 234 | 238 | GVFVG | 5 | 5.56 | EEEES | 9 | 2.08 |
| 2578 | 1NKI:A | transferase | 116 | 120 | LRSRL | 5 | 37.78 | HHHHH | 9 | -0.44 |
| 2579 | 1P0Z:J | transferase | 119 | 123 | VIGIV | 5 | 9.60 | EEEEE | 6 | 3.40 |
| 2580 | 2H00:C | transferase | 246 | 250 | WALAW | 5 | 5.24 | EEEEE | 11 | 1.12 |
| 2581 | 3HUL:B | transferase | 24 | 28 | TLYLT | 5 | 27.34 | EEEEE | 9 | 0.98 |
| 2582 | 3LED:B | transferase | 225 | 229 | TKLKT | 5 | 42.46 | EEEEE | 8 | -1.08 |
| 2583 | 3O0P:A | transferase , hydrolase | 170 | 174 | KKLKK | 5 | 79.84 | GGCCT | 7 | -2.36 |
| 2584 | 1G8M:A | transferase, hydrolase | 406 | 410 | TKNKT | 5 | 91.62 | SSSCC | 9 | -2.54 |
| 2585 | 1G8M:A | transferase, hydrolase | 28 | 32 | LGLGL | 5 | 21.12 | TTCEE | 9 | 2.12 |
| 2586 | 2GWM:A | transferase, toxin | 572 | 576 | DATAD | 5 | 71.70 | STTCC | 6 | -0.82 |
| 2587 | 3R24:A | transferase, viral protein | 105 | 109 | SDADS | 5 | 30.06 | CSSSE | 8 | -1.36 |
| 2588 | 1S5D:A | transferase,toxin | 145 | 149 | YRDRY | 5 | 35.02 | CCHHH | 6 | -3.02 |
| 2589 | 3ESS:A | transferase,toxin | 509 | 513 | EGTGE | 5 | 86.70 | SCCSG | 5 | -1.70 |
| 2590 | 3SG8:A | transferase/antibiotic | 155 | 159 | KKIKK | 5 | 69.56 | HHHHH | 9 | -2.22 |
| 2591 | 3SG8:A | transferase/antibiotic | 132 | 136 | SINIS | 5 | 69.58 | HSCCT | 6 | 0.78 |
| 2592 | 3JZ0:A | transferase/antibiotic | 67 | 71 | YLMLY | 5 | 32.80 | EEEEE | 7 | 1.38 |
| 2593 | 3MDY:A | transferase/isomerase | 187 | 191 | GSGSG | 5 | 36.42 | CSCSS | 3 | -0.56 |
| 2594 | 3MDY:A | transferase/isomerase | 186 | 190 | SGSGS | 5 | 47.20 | HCSCS | 2 | -0.64 |
| 2595 | 3MDY:A | transferase/isomerase | 287 | 291 | LYDYL | 5 | 33.52 | HHHHH | 9 | 0.30 |
| 2596 | 3MDY:A | transferase/isomerase | 227 | 231 | KVAVK | 5 | 26.66 | EEEEE | 9 | 0.48 |
| 2597 | 3CJS:C | transferase/ribosomal protein | 3 | 9 | KVVAVVK | 7 | 70.30 | CEEEEEE | 4 | 1.54 |
| 2598 | 3CJS:C | transferase/ribosomal protein | 4 | 8 | VVAVV | 5 | 57.76 | EEEEE | 4 | 3.72 |
| 2599 | 3P8B:D | transferase/transcription | 131 | 135 | VPIPV | 5 | 26.00 | SCCCE | 4 | 1.94 |
| 2600 | 3ACX:A | transferase/transferase inhibitor | 128 | 132 | GYCYG | 5 | 27.02 | HHHHH | 10 | -0.18 |
| 2601 | 3ACX:A | transferase/transferase inhibitor | 166 | 170 | LINIL | 5 | 10.64 | HHHHH | 10 | 2.62 |
| 2602 | 3D36:C | transferase/transferase inhibitor | 29 | 33 | IEKEI | 5 | 34.48 | HHHHH | 9 | -0.38 |
| 2603 | 3QWW:A | transferase/transferase inhibitor | 270 | 274 | KDKDK | 5 | 95.10 | CTTHH | 7 | -3.74 |
| 2604 | 3QWW:A | transferase/transferase inhibitor | 252 | 256 | DRLRD | 5 | 71.34 | HHHHH | 11 | -2.44 |
| 2605 | 3RQ7:A | transferase/transferase inhibitor | 421 | 425 | YGLGY | 5 | 15.36 | TEEEE | 25 | 0.08 |
| 2606 | 3THR:D | transferase/transferase inhibitor | 37 | 41 | TRSRT | 5 | 70.22 | CSCBC | 11 | -2.24 |
| 2607 | 3THR:D | transferase/transferase inhibitor | 48 | 52 | LLGLL | 5 | 13.88 | HHHHH | 7 | 2.96 |
| 2608 | 4DEM:F | transferase/transferase inhibitor | 307 | 311 | KEAEK | 5 | 91.72 | CCHHH | 5 | -2.60 |
| 2609 | 4DEM:F | transferase/transferase inhibitor | 227 | 231 | AMYMA | 5 | 18.26 | HHHHT | 5 | 1.22 |
| 2610 | 1F60:B | translation | 1161 | 1165 | IGFGI | 5 | 20.94 | EETTE | 3 | 2.20 |
| 2611 | 1G61:B | translation | 4092 | 4096 | NLDLN | 5 | 84.96 | TCCCE | 9 | -0.58 |
| 2612 | 1IS1:A | translation | 119 | 123 | GEAEG | 5 | 44.26 | HHHHH | 8 | -1.20 |
| 2613 | 1IS1:A | translation | 125 | 129 | RVAVR | 5 | 46.16 | HHHHH | 8 | 0.24 |
| 2614 | 1KL9:A | translation | 102 | 106 | TKSKT | 5 | 47.92 | HHHHH | 8 | -2.00 |
| 2615 | 2D1P:F | translation | 17 | 21 | LLRLL | 5 | 24.02 | HHHTC | 8 | 2.14 |
| 2616 | 3D3M:A | translation | 733 | 737 | LEKEL | 5 | 48.56 | HHHHH | 7 | -0.66 |
| 2617 | 3HXI:A | translation | 117 | 121 | LLMLL | 5 | 1.96 | HHHHH | 8 | 3.42 |
| 2618 | 3NS6:A | translation | 121 | 125 | GKTKG | 5 | 33.16 | TEECS | 9 | -1.86 |
| 2619 | 1M5Q:2 | translation | 132 | 137 | LFEEFL | 6 | 34.85 | HHHHHH | 7 | 1.03 |
| 2620 | 1DZK:B | transport | 130 | 134 | EKFKE | 5 | 65.12 | HHHHH | 9 | -2.40 |
| 2621 | 3RPW:A | transport protein | 338 | 344 | GAPSPAG | 7 | 53.96 | HSBCTTS | 8 | -0.17 |
| 2622 | 2ZFI:A | transport protein | 140 | 146 | YSVEVSY | 7 | 23.73 | EEEEEEE | 10 | 0.10 |
| 2623 | 1B0U:A | transport protein | 129 | 133 | ARERA | 5 | 47.22 | HHHHH | 9 | -1.78 |
| 2624 | 1B0U:A | transport protein | 91 | 95 | LRTRL | 5 | 40.88 | HHHHE | 8 | -0.42 |
| 2625 | 1I4U:A | transport protein | 133 | 137 | SFIFS | 5 | 9.46 | EEEEE | 11 | 1.70 |
| 2626 | 1JKG:A | transport protein | 109 | 113 | FNQNF | 5 | 6.46 | EEEEE | 12 | -0.98 |
| 2627 | 1JKG:A | transport protein | 56 | 60 | ESLSE | 5 | 66.08 | HHHHH | 12 | -0.96 |
| 2628 | 1S6C:A | transport protein | 183 | 187 | DKNKD | 5 | 75.04 | CTTCS | 7 | -3.66 |
| 2629 | 1U7G:A | transport protein | 95 | 99 | IYQYI | 5 | 51.62 | EEHHH | 8 | 0.58 |
| 2630 | 1U7G:A | transport protein | 264 | 268 | GAIAG | 5 | 2.24 | HHHHH | 9 | 1.46 |
| 2631 | 1URQ:C | transport protein | 72 | 76 | KEAEK | 5 | 52.38 | HHHHH | 8 | -2.60 |
| 2632 | 2BWQ:A | transport protein | 833 | 837 | EILIE | 5 | 51.58 | EEEEE | 5 | 1.16 |
| 2633 | 2FVY:A | transport protein | 248 | 252 | GALAG | 5 | 21.58 | TSSCB | 18 | 1.32 |
| 2634 | 2G1U:A | transport protein | 131 | 135 | EKVKE | 5 | 96.50 | HHHHH | 8 | -2.12 |
| 2635 | 2HEU:A | transport protein | 280 | 284 | GKEKG | 5 | 85.72 | CSSTT | 3 | -2.42 |
| 2636 | 2O6P:B | transport protein | 28 | 32 | GSDSG | 5 | 70.94 | CCEEE | 3 | -1.18 |
| 2637 | 2PFI:B | transport protein | 567 | 571 | VVKVV | 5 | 39.46 | HHHHH | 8 | 2.58 |
| 2638 | 2R5O:B | transport protein | 267 | 271 | DEIED | 5 | 102.26 | TCCCC | 6 | -1.90 |
| 2639 | 2V14:A | transport protein | 1237 | 1242 | ELAALE | 6 | 65.00 | GGGGCC | 3 | 0.70 |
| 2640 | 2VE8:E | transport protein | 785 | 789 | AMEMA | 5 | 63.12 | HHHHT | 6 | 0.78 |
| 2641 | 2VE8:E | transport protein | 763 | 767 | ASISA | 5 | 9.74 | CSHHH | 6 | 1.30 |
| 2642 | 2WCJ:A | transport protein | 102 | 106 | DTETD | 5 | 87.52 | TTCCC | 9 | -2.38 |
| 2643 | 2XE5:A | transport protein | 29 | 33 | KDGDK | 5 | 35.18 | GCEEC | 6 | -3.04 |
| 2644 | 2XE5:A | transport protein | 24 | 28 | SDNDS | 5 | 60.76 | ESSGG | 6 | -2.42 |
| 2645 | 2XE5:A | transport protein | 235 | 239 | YTQTY | 5 | 42.16 | EEEEE | 11 | -1.50 |
| 2646 | 2YLN:A | transport protein | 138 | 142 | AVLVA | 5 | 4.70 | EEEEE | 9 | 3.16 |
| 2647 | 2ZFI:A | transport protein | 141 | 145 | SVEVS | 5 | 21.08 | EEEEE | 8 | 0.66 |
| 2648 | 3B9W:A | transport protein | 330 | 334 | LALAL | 5 | 40.96 | HHHHH | 8 | 3.00 |
| 2649 | 3C1Q:A | transport protein | 118 | 122 | LSDSL | 5 | 24.34 | HHHHH | 7 | 0.50 |
| 2650 | 3CIJ:B | transport protein | 33 | 37 | VKLKV | 5 | 47.58 | CEEEE | 8 | 0.88 |
| 2651 | 3D32:B | transport protein | 66 | 70 | IRKRI | 5 | 64.60 | HHHHT | 9 | -0.78 |
| 2652 | 3E8T:A | transport protein | 61 | 65 | GKLKG | 5 | 45.92 | EEEEC | 6 | -0.96 |
| 2653 | 3E8T:A | transport protein | 115 | 119 | KLKLK | 5 | 68.96 | EEEEE | 9 | -0.82 |
| 2654 | 3E8T:A | transport protein | 46 | 50 | DFAFD | 5 | 65.88 | CEEEE | 6 | 0.08 |
| 2655 | 3EIX:A | transport protein | 168 | 172 | KEGEK | 5 | 106.42 | HHHHH | 6 | -3.04 |
| 2656 | 3EIX:A | transport protein | 297 | 301 | VDRDV | 5 | 34.12 | EEHHH | 8 | -0.62 |
| 2657 | 3K06:B | transport protein | 32 | 36 | ILTLI | 5 | 58.70 | HHHHH | 9 | 3.18 |
| 2658 | 3KCU:C | transport protein | 57 | 61 | TGTGT | 5 | 52.60 | TTCSS | 5 | -0.58 |
| 2659 | 3MD9:A | transport protein | 258 | 262 | LGFGL | 5 | 17.52 | HSCST | 7 | 1.92 |
| 2660 | 3PIK:A | transport protein | 91 | 95 | GSWSG | 5 | 73.76 | EEEEC | 4 | -0.66 |
| 2661 | 3PIK:A | transport protein | 115 | 119 | FDLDF | 5 | 73.72 | EECCT | 4 | 0.48 |
| 2662 | 3PIK:A | transport protein | 282 | 286 | AARAA | 5 | 28.24 | HHHHT | 4 | 0.54 |
| 2663 | 3PPQ:A | transport protein | 102 | 106 | KEAEK | 5 | 56.68 | CCCCC | 8 | -2.60 |
| 2664 | 3PPQ:A | transport protein | 188 | 192 | GFEFG | 5 | 47.78 | CCCCS | 8 | 0.26 |
| 2665 | 3PPQ:A | transport protein | 96 | 100 | LTSTL | 5 | 23.50 | HHTTS | 8 | 1.08 |
| 2666 | 3QM9:A | transport protein | 2 | 6 | ADFDA | 5 | 55.20 | CHHHH | 5 | -0.12 |
| 2667 | 3RPW:A | transport protein | 339 | 343 | APSPA | 5 | 62.22 | SBCTT | 7 | -0.08 |
| 2668 | 3SWF:C | transport protein | 35 | 39 | QKLKQ | 5 | 71.26 | HHHHH | 7 | -2.20 |
| 2669 | 1N7S:B | transport protein | 214 | 218 | DMFMD | 5 | 47.08 | HHHHH | 8 | -0.08 |
| 2670 | 1P28:A | transport protein | 45 | 49 | LACAL | 5 | 5.54 | HHHHH | 9 | 2.74 |
| 2671 | 3QQ8:B | transport protein/apoptosis | 599 | 603 | VFDFV | 5 | 21.94 | HHHHH | 7 | 2.10 |
| 2672 | 3FM8:B | transport protein/hydrolase activator | 518 | 522 | SSVSS | 5 | 68.74 | EECCS | 4 | 0.20 |
| 2673 | 1QGK:B | transport receptor | 33 | 37 | EVNVE | 5 | 74.28 | HHHHH | 7 | -0.42 |
| 2674 | 1VR9:B | unknown function | 55 | 59 | DLDLD | 5 | 96.00 | TSCTT | 3 | -0.58 |
| 2675 | 3FX7:B | unknown function | 34 | 38 | NHFHN | 5 | 71.64 | HHHHH | 7 | -2.12 |
| 2676 | 3P02:A | unknown function | 305 | 311 | NYEIEYN | 7 | 87.17 | TCCEEEE | 10 | -1.73 |
| 2677 | 1J7D:A | unknown function | 17 | 21 | EELEE | 5 | 45.54 | HHHHH | 13 | -2.04 |
| 2678 | 1O3U:A | unknown function | 89 | 93 | PSGSP | 5 | 67.08 | CTTHH | 5 | -1.04 |
| 2679 | 1SQW:A | unknown function | 5 | 9 | TEEET | 5 | 81.84 | CHHHH | 6 | -2.38 |
| 2680 | 1VMH:A | unknown function | 126 | 130 | KVFVK | 5 | 42.52 | EEEEE | 7 | 0.68 |
| 2681 | 1VR9:B | unknown function | 54 | 58 | LDLDL | 5 | 97.56 | TTSCT | 5 | 0.88 |
| 2682 | 1YKW:A | unknown function | 393 | 397 | KAGAK | 5 | 28.92 | HHHHH | 4 | -0.92 |
| 2683 | 1ZEE:A | unknown function | 233 | 237 | GANAG | 5 | 28.40 | CCCTT | 4 | -0.14 |
| 2684 | 2B8M:A | unknown function | 43 | 47 | YSNSY | 5 | 57.90 | ECSSC | 7 | -1.54 |
| 2685 | 2HUH:A | unknown function | 120 | 124 | YYLYY | 5 | 40.80 | SEEEE | 15 | -0.28 |
| 2686 | 2OD4:A | unknown function | 51 | 55 | SNENS | 5 | 58.48 | EETTE | 4 | -2.42 |
| 2687 | 2ODM:A | unknown function | 77 | 81 | LEKEL | 5 | 63.72 | HHHHH | 7 | -0.66 |
| 2688 | 2ODM:A | unknown function | 22 | 26 | ILHLI | 5 | 41.00 | HHHHH | 7 | 2.68 |
| 2689 | 2OZJ:B | unknown function | 48 | 52 | ESVSE | 5 | 69.14 | CEEEE | 6 | -0.88 |
| 2690 | 2P92:B | unknown function | 80 | 84 | QLILQ | 5 | 25.24 | HHHHH | 8 | 1.02 |
| 2691 | 2Q30:B | unknown function | 62 | 66 | EGEGE | 5 | 49.44 | ESCEE | 8 | -2.26 |
| 2692 | 2Q30:B | unknown function | 81 | 85 | AVLVA | 5 | 4.62 | EEEEE | 10 | 3.16 |
| 2693 | 2QML:A | unknown function | 117 | 121 | LGQGL | 5 | 62.32 | SSSST | 5 | 0.66 |
| 2694 | 2R4I:B | unknown function | 69 | 73 | IIRII | 5 | 38.72 | EEEEE | 7 | 2.70 |
| 2695 | 2RFR:A | unknown function | 61 | 65 | AAIAA | 5 | 37.88 | HHHHH | 6 | 2.34 |
| 2696 | 2UVP:D | unknown function | 134 | 138 | FLWLF | 5 | 10.38 | EEEES | 7 | 2.46 |
| 2697 | 2VH3:A | unknown function | 18 | 22 | LAEAL | 5 | 18.46 | HHHHH | 6 | 1.54 |
| 2698 | 2XDJ:F | unknown function | 34 | 38 | SDIDS | 5 | 53.44 | HHHHH | 9 | -0.82 |
| 2699 | 2XDJ:F | unknown function | 25 | 29 | LQQQL | 5 | 52.86 | HHHHH | 9 | -0.58 |
| 2700 | 2Y3C:A | unknown function | 233 | 237 | GSNSG | 5 | 46.46 | TTTSC | 8 | -1.18 |
| 2701 | 3BB9:F | unknown function | 105 | 109 | ISTSI | 5 | 11.46 | EEEEE | 12 | 1.34 |
| 2702 | 3BF4:A | unknown function | 48 | 52 | GSASG | 5 | 67.72 | CSSTT | 2 | -0.12 |
| 2703 | 3BF4:A | unknown function | 47 | 53 | AGSASGA | 7 | 55.56 | ECSSTTC | 2 | 0.43 |
| 2704 | 3BF4:A | unknown function | 100 | 104 | EVVVE | 5 | 52.54 | EEEES | 2 | 1.12 |
| 2705 | 3BL4:A | unknown function | 12 | 16 | GLTLG | 5 | 45.98 | TEEEC | 8 | 1.22 |
| 2706 | 3D7A:B | unknown function | 7 | 11 | EVEVE | 5 | 25.36 | EEEEE | 8 | -0.42 |
| 2707 | 3D7A:B | unknown function | 111 | 115 | ITITI | 5 | 8.38 | EEEEE | 8 | 2.42 |
| 2708 | 3FCN:A | unknown function | 138 | 142 | APLPA | 5 | 39.16 | CSSCH | 5 | 0.84 |
| 2709 | 3FX7:B | unknown function | 30 | 34 | NSLSN | 5 | 46.14 | HHHHH | 9 | -0.96 |
| 2710 | 3H3H:A | unknown function | 52 | 56 | GRLRG | 5 | 50.28 | CEEEH | 9 | -1.20 |
| 2711 | 3H3H:A | unknown function | 6 | 10 | QAFAQ | 5 | 51.14 | HHHHH | 7 | -0.12 |
| 2712 | 3OZY:A | unknown function | 50 | 54 | GSPSG | 5 | 1.46 | ECCCS | 6 | -0.80 |
| 2713 | 3OZY:A | unknown function | 292 | 297 | ASAASA | 6 | 49.67 | HHHHHT | 6 | 0.93 |
| 2714 | 3P02:A | unknown function | 306 | 310 | YEIEY | 5 | 83.90 | CCEEE | 9 | -1.02 |
| 2715 | 3P0T:B | unknown function | 124 | 128 | AKIKA | 5 | 60.62 | HHHHH | 8 | 0.06 |
| 2716 | 3P42:D | unknown function | 54 | 58 | LRDRL | 5 | 62.48 | TTTTS | 19 | -0.98 |
| 2717 | 3P42:D | unknown function | 70 | 74 | AKAKA | 5 | 57.66 | HHHHH | 13 | -0.48 |
| 2718 | 3P8A:B | unknown function | 185 | 189 | FQKQF | 5 | 48.80 | CCTTE | 8 | -1.06 |
| 2719 | 3QSZ:B | unknown function | 121 | 125 | AGDGA | 5 | 59.24 | CTTSC | 2 | -0.14 |
| 2720 | 3RQA:D | unknown function | 113 | 117 | PHAHP | 5 | 80.30 | SSCCC | 5 | -1.56 |
| 2721 | 3RQA:D | unknown function | 161 | 165 | LGTGL | 5 | 40.90 | CCHHH | 5 | 1.22 |
| 2722 | 3RQA:D | unknown function | 81 | 85 | ILALI | 5 | 9.48 | HHHHH | 9 | 3.68 |
| 2723 | 3RQT:A | unknown function | 192 | 196 | NRVRN | 5 | 38.88 | HHHHH | 10 | -2.36 |
| 2724 | 3RQT:A | unknown function | 312 | 316 | LKIKL | 5 | 38.24 | EEEEE | 10 | 0.86 |
| 2725 | 3S9J:A | unknown function | 350 | 354 | KYFYK | 5 | 33.70 | SCEEE | 9 | -1.52 |
| 2726 | 3TU8:A | unknown function | 193 | 197 | SSGSS | 5 | 75.90 | TTEEC | 4 | -0.72 |
| 2727 | 3TU8:A | unknown function | 202 | 206 | LTYTL | 5 | 49.12 | ECCEE | 4 | 0.98 |
| 2728 | 3U80:A | unknown function | 125 | 129 | GTITG | 5 | 11.26 | EEEES | 9 | 0.46 |
| 2729 | 4E0Q:B | unknown function | 87 | 91 | GKQKG | 5 | 79.52 | EEEET | 6 | -2.42 |
| 2730 | 4E0Q:B | unknown function | 151 | 155 | IQRQI | 5 | 102.04 | HHHHT | 6 | -0.50 |
| 2731 | 2B1Y:A | unknown function | 93 | 97 | AQPQA | 5 | 123.86 | CCCCC | 2 | -1.00 |
| 2732 | 3D33:A | unknown function | 78 | 82 | TIRIT | 5 | 23.52 | EEEEE | 9 | 0.62 |
| 2733 | 3MYU:B | vib binding protein | 121 | 125 | DLELD | 5 | 60.64 | BCSCC | 6 | -0.58 |
| 2734 | 3MYU:B | vib binding protein | 259 | 263 | INKNI | 5 | 83.40 | SSTTC | 6 | -0.38 |
| 2735 | 3MYU:B | vib binding protein | 326 | 330 | EIFIE | 5 | 40.64 | HHHHH | 9 | 0.96 |
| 2736 | 3KU3:B | viral protein | 3 | 9 | FGAIAGF | 7 | 36.63 | TSTBTTT | 5 | 1.84 |
| 2737 | 2WUX:A | viral protein | 18 | 23 | NKYYKN | 6 | 54.27 | TEEEEE | 12 | -2.90 |
| 2738 | 3GQH:A | viral protein | 777 | 783 | NPDFDPN | 7 | 92.14 | CTTCCSS | 4 | -2.06 |
| 2739 | 1QHV:A | viral protein | 542 | 546 | STETS | 5 | 95.18 | CCCTT | 4 | -1.30 |
| 2740 | 1M93:A | viral protein | 18 | 23 | ISPPSI | 6 | 9.97 | ECHHHH | 9 | 0.70 |
| 2741 | 2VBK:A | viral protein | 328 | 332 | SVSVS | 5 | 16.60 | EEEEE | 12 | 1.20 |
| 2742 | 1LUZ:B | viral protein | 63 | 67 | VKVKV | 5 | 28.72 | EEEEE | 11 | 0.96 |
| 2743 | 1LUZ:B | viral protein | 26 | 30 | LYIYL | 5 | 35.04 | EEEEE | 11 | 1.90 |
| 2744 | 1MN8:D | viral protein | 73 | 77 | PHGHP | 5 | 87.06 | GGCCG | 5 | -2.00 |
| 2745 | 1MN8:D | viral protein | 64 | 68 | VKIKV | 5 | 46.06 | HHHHH | 8 | 1.02 |
| 2746 | 1QHV:A | viral protein | 541 | 547 | ESTETSE | 7 | 79.23 | SCCCTTC | 5 | -1.93 |
| 2747 | 1QHV:A | viral protein | 507 | 511 | TQSQT | 5 | 92.30 | CSSCC | 5 | -1.84 |
| 2748 | 1QHV:A | viral protein | 539 | 543 | TSEST | 5 | 37.98 | GGSCC | 5 | -1.30 |
| 2749 | 1QHV:A | viral protein | 422 | 426 | TLVLT | 5 | 20.32 | EEEEE | 8 | 2.08 |
| 2750 | 1QHV:A | viral protein | 532 | 536 | LTITL | 5 | 8.06 | EEEEE | 8 | 2.14 |
| 2751 | 1SVF:C | viral protein | 130 | 134 | ANENA | 5 | 81.00 | HHHHH | 8 | -1.38 |
| 2752 | 1YU0:A | viral protein | 39 | 43 | GATAG | 5 | 51.04 | SSSTT | 5 | 0.42 |
| 2753 | 1ZVA:A | viral protein | 39 | 43 | GGRGG | 5 | 80.00 | TTSCC | 5 | -1.22 |
| 2754 | 2ACF:D | viral protein | 323 | 327 | QVCVQ | 5 | 42.14 | HHHHH | 6 | 0.78 |
| 2755 | 2CMP:A | viral protein | 51 | 55 | IRARI | 5 | 51.74 | HHHHH | 7 | 0.36 |
| 2756 | 2CMP:A | viral protein | 27 | 31 | AAIAA | 5 | 23.10 | HHHHT | 7 | 2.34 |
| 2757 | 2F0C:A | viral protein | 53 | 57 | NVEVN | 5 | 41.50 | CEEES | 7 | -0.42 |
| 2758 | 2F0C:A | viral protein | 52 | 58 | GNVEVNG | 7 | 34.97 | SCEEESS | 7 | -0.41 |
| 2759 | 2F0C:A | viral protein | 94 | 98 | ISSSI | 5 | 45.58 | CCSCB | 7 | 1.32 |
| 2760 | 2FKK:A | viral protein | 517 | 521 | GGTGG | 5 | 58.90 | CCCCC | 6 | -0.46 |
| 2761 | 2FQM:A | viral protein | 121 | 125 | TLRLT | 5 | 17.40 | EEEEE | 10 | 0.34 |
| 2762 | 2HNF:A | viral protein | 136 | 140 | ASDSA | 5 | 49.86 | CCTTC | 6 | -0.30 |
| 2763 | 2OJ6:A | viral protein | 374 | 379 | LLPPLL | 6 | 74.83 | BCTTCC | 5 | 2.00 |
| 2764 | 2V33:B | viral protein | 376 | 380 | CSASC | 5 | 41.18 | EECCC | 9 | 1.04 |
| 2765 | 2VBK:A | viral protein | 288 | 292 | NVMVN | 5 | 21.40 | SCEEE | 11 | 0.66 |
| 2766 | 2VBK:A | viral protein | 327 | 331 | VSVSV | 5 | 18.40 | EEEEE | 11 | 2.20 |
| 2767 | 2VVW:A | viral protein | 100 | 104 | LKQKL | 5 | 74.68 | HHHHT | 6 | -0.74 |
| 2768 | 2WUX:A | viral protein | 152 | 156 | VIRIV | 5 | 82.28 | EEECC | 5 | 2.58 |
| 2769 | 2X4J:A | viral protein | 112 | 116 | EVLVE | 5 | 27.18 | EEEEE | 9 | 1.04 |
| 2770 | 2X6W:A | viral protein | 398 | 402 | AAEAA | 5 | 36.88 | GGGCT | 7 | 0.74 |
| 2771 | 2X6W:A | viral protein | 431 | 435 | VTATV | 5 | 80.76 | BCSSC | 7 | 1.76 |
| 2772 | 2Y4Z:A | viral protein | 60 | 65 | GTLLTG | 6 | 41.58 | HHHSCH | 8 | 0.90 |
| 2773 | 3BJQ:A | viral protein | 153 | 157 | PDSDP | 5 | 51.50 | TTCCH | 7 | -2.20 |
| 2774 | 3C6A:A | viral protein | 385 | 389 | EKPKE | 5 | 99.42 | SCCCT | 7 | -3.28 |
| 2775 | 3FAV:C | viral protein | 50 | 54 | AAQAA | 5 | 24.30 | HHHHH | 8 | 0.74 |
| 2776 | 3GQH:A | viral protein | 778 | 782 | PDFDP | 5 | 98.16 | TTCCS | 3 | -1.48 |
| 2777 | 3GQH:A | viral protein | 820 | 825 | AVGGVA | 6 | 21.28 | EETTEE | 3 | 1.87 |
| 2778 | 3KU3:B | viral protein | 4 | 8 | GAIAG | 5 | 24.14 | STBTT | 6 | 1.46 |
| 2779 | 3LPH:D | viral protein | 38 | 42 | RRNRR | 5 | 87.00 | HHHHH | 8 | -4.30 |
| 2780 | 3LPH:D | viral protein | 8 | 12 | SDEDS | 5 | 97.04 | CTTHH | 4 | -2.42 |
| 2781 | 3LPH:D | viral protein | 26 | 30 | NPPPN | 5 | 61.54 | SCCCC | 4 | -2.36 |
| 2782 | 3O3X:A | viral protein | 78 | 83 | GSGGSG | 6 | 61.38 | TCCCTH | 8 | -0.53 |
| 2783 | 3O3X:A | viral protein | 38 | 42 | GGSGG | 5 | 45.42 | SSSTT | 8 | -0.48 |
| 2784 | 3O3X:A | viral protein | 77 | 81 | GGSGG | 5 | 45.42 | SSSTT | 8 | -0.48 |
| 2785 | 3O3X:A | viral protein | 119 | 123 | GGSGG | 5 | 45.42 | SSSTT | 8 | -0.48 |
| 2786 | 3O3X:A | viral protein | 158 | 162 | GGSGG | 5 | 45.42 | SSSTT | 8 | -0.48 |
| 2787 | 3ON9:B | viral protein | 195 | 199 | LTITL | 5 | 14.94 | EEEEE | 8 | 2.14 |
| 2788 | 3Q39:A | viral protein | 422 | 426 | LNMNL | 5 | 80.74 | CCCSC | 4 | 0.50 |
| 2789 | 3QR7:B | viral protein | 135 | 139 | VTATV | 5 | 104.22 | CCCCC | 6 | 1.76 |
| 2790 | 3VC8:A | viral protein | 32 | 36 | YLSLY | 5 | 45.20 | HHHTH | 9 | 0.84 |
| 2791 | 2NXY:B | viral protein/immune system | 1148 | 1152 | QLELQ | 5 | 74.04 | SCCGG | 5 | -0.58 |
| 2792 | 2NXY:B | viral protein/immune system | 1114 | 1118 | LTLTL | 5 | 22.82 | EEEEE | 8 | 2.00 |
| 2793 | 3SE8:G | viral protein/immune system | 102 | 106 | EQMQE | 5 | 49.14 | HHHHH | 17 | -2.42 |
| 2794 | 3SE8:G | viral protein/immune system | 347 | 351 | EKLKE | 5 | 55.94 | HHHHH | 17 | -2.20 |
| 2795 | 3SE8:G | viral protein/immune system | 428 | 432 | QGTGQ | 5 | 40.22 | TTTEE | 13 | -1.70 |
| 2796 | 2RHK:A | viral protein/nuclear protein | 161 | 165 | SPLPS | 5 | 62.70 | EECTT | 5 | -0.20 |
| 2797 | 3L4Q:D | viral protein/protein binding | 493 | 498 | EKSSKE | 6 | 85.67 | HHHTTT | 5 | -2.73 |
| 2798 | 3JRV:A | viral protein/protein binding | 11 | 15 | VFYFV | 5 | 69.78 | CCTTT | 6 | 2.54 |
| 2799 | 3L4Q:D | viral protein/protein binding | 536 | 540 | LEQEL | 5 | 60.70 | HHHHH | 7 | -0.58 |
| 2800 | 1AYM:1 | virus | 86 | 90 | DNYND | 5 | 79.16 | SCHHH | 0 | -3.06 |
| 2801 | 1AYM:1 | virus | 238 | 242 | HKAKH | 5 | 11.04 | EEEEE | 0 | -2.48 |
| 2802 | 1STM:A | virus | 140 | 144 | TVIVT | 5 | 8.96 | EEEEE | 9 | 2.30 |
| 2803 | 2BKF:A | zinc-finger protein | 51 | 55 | EENEE | 5 | 102.84 | TTSCE | 3 | -3.50 |
