# Supplementary material for: Analyses of the Sequence and Structural Properties Corresponding to Pentapeptide and Large Palindromes in Proteins
Source: PLoS One. 2015 Oct 14;10(10):e0139568. doi: 10.1371/journal.pone.0139568 (PMC4605511; doi:10.1371/journal.pone.0139568)
Supplement: S3 Appendix — (DOC) [file pone.0139568.s003.doc]

| **S3 Appendix. List of 2014 unique palindrome sequences (orange) in representative proteins of known three-dimensional structure.** | | | | | |
| --- | --- | --- | --- | --- | --- |
| S.No. | Unique PALIN S.No. | PALIN sequence | PDB code:chain | Start position | End position |
| 1 | 1 | AAARIRAAA | 3HIM:A | 17 | 25 |
| 2 | 2 | AADAA | 1OI7:A | 77 | 81 |
| 3 | 3 | AAEAA | 1FEW:A | 112 | 116 |
| 4 |  | AAEAA | 3LHI:A | 10 | 14 |
| 5 |  | AAEAA | 1EB6:A | 28 | 32 |
| 6 |  | AAEAA | 3A9S:C | 73 | 77 |
| 7 |  | AAEAA | 1X0G:D | 67 | 71 |
| 8 |  | AAEAA | 1H7E:A | 131 | 135 |
| 9 |  | AAEAA | 1WUI:S | 55 | 59 |
| 10 |  | AAEAA | 3M7A:B | 31 | 35 |
| 11 |  | AAEAA | 2X6W:A | 398 | 402 |
| 12 | 4 | AAEEAA | 2IJ2:A | 290 | 295 |
| 13 | 5 | AAEQEAA | 2D0B:A | 57 | 63 |
| 14 | 6 | AAGAA | 1NWW:A | 16 | 20 |
| 15 |  | AAGAA | 2BJI:B | 2204 | 2208 |
| 16 |  | AAGAA | 2JHF:A | 213 | 217 |
| 17 |  | AAGAA | 1SU7:A | 87 | 91 |
| 18 | 7 | AAGGAA | 3R0V:A | 81 | 86 |
| 19 | 8 | AAHAA | 3FSA:A | 113 | 117 |
| 20 | 9 | AAIAA | 2PNL:J | 674 | 678 |
| 21 |  | AAIAA | 1MJ5:A | 284 | 288 |
| 22 |  | AAIAA | 3PWK:A | 271 | 275 |
| 23 |  | AAIAA | 2IBP:B | 239 | 243 |
| 24 |  | AAIAA | 1EKQ:A | 217 | 221 |
| 25 |  | AAIAA | 1MDO:A | 267 | 271 |
| 26 |  | AAIAA | 2RFR:A | 61 | 65 |
| 27 |  | AAIAA | 2CMP:A | 27 | 31 |
| 28 |  | AAIAA | 3S6E:A | 483 | 487 |
| 29 | 10 | AAKAA | 3DMO:D | 9 | 13 |
| 30 |  | AAKAA | 1BYI:A | 25 | 29 |
| 31 |  | AAKAA | 2GDQ:B | 104 | 108 |
| 32 |  | AAKAA | 2OKQ:B | 23 | 27 |
| 33 | 11 | AALAA | 2XF3:A | 14 | 18 |
| 34 |  | AALAA | 3QPA:A | 123 | 127 |
| 35 |  | AALAA | 1GYX:B | 18 | 22 |
| 36 |  | AALAA | 1X8Q:A | 42 | 46 |
| 37 |  | AALAA | 2PVB:A | 13 | 17 |
| 38 |  | AALAA | 3P7X:A | 156 | 160 |
| 39 |  | AALAA | 3P1X:A | 580 | 584 |
| 40 | 12 | AALLAA | 3KUU:C | 134 | 139 |
| 41 | 13 | AALTLAA | 2Y2Z:A | 98 | 104 |
| 42 | 14 | AAMAA | 1GKP:E | 248 | 252 |
| 43 | 15 | AAMMAA | 3BZN:A | 208 | 213 |
| 44 | 16 | AANAA | 2VXN:A | 216 | 220 |
| 45 |  | AANAA | 3KUU:C | 131 | 135 |
| 46 |  | AANAA | 1Y7T:B | 245 | 249 |
| 47 | 17 | AAPAA | 2V6K:A | 203 | 207 |
| 48 |  | AAPAA | 2VFR:A | 240 | 244 |
| 49 | 18 | AAQAA | 3A1B:A | 571 | 575 |
| 50 |  | AAQAA | 1O9G:A | 127 | 131 |
| 51 |  | AAQAA | 3FAV:C | 50 | 54 |
| 52 | 19 | AARAA | 3B0B:C | 48 | 52 |
| 53 |  | AARAA | 1O04:G | 65 | 69 |
| 54 |  | AARAA | 3PIK:A | 282 | 286 |
| 55 |  | AARAA | 3AWU:B | 107 | 111 |
| 56 | 20 | AARIRAA | 3HIM:A | 18 | 24 |
| 57 | 21 | AASAA | 2VFO:A | 136 | 140 |
| 58 |  | AASAA | 1ITX:A | 300 | 304 |
| 59 |  | AASAA | 1Y7T:B | 242 | 246 |
| 60 | 22 | AATAA | 1L3P:A | 168 | 172 |
| 61 |  | AATAA | 1H41:B | 477 | 481 |
| 62 |  | AATAA | 1ITX:A | 174 | 178 |
| 63 |  | AATAA | 3VCX:B | 16 | 20 |
| 64 |  | AATAA | 3DA8:B | 83 | 87 |
| 65 | 23 | AATKKTAA | 2I5V:O | 208 | 215 |
| 66 | 24 | AAVAA | 2GMW:B | 163 | 167 |
| 67 |  | AAVAA | 3LYE:A | 269 | 273 |
| 68 |  | AAVAA | 3GWR:A | 52 | 56 |
| 69 |  | AAVAA | 2IBP:B | 115 | 119 |
| 70 |  | AAVAA | 1GA8:A | 46 | 50 |
| 71 |  | AAVAA | 3DA8:B | 189 | 193 |
| 72 |  | AAVAA | 1GK8:A | 55 | 59 |
| 73 | 25 | AAVVAA | 1HBN:A | 402 | 407 |
| 74 | 26 | AAYAA | 2BFF:A | 174 | 178 |
| 75 |  | AAYAA | 2P02:A | 316 | 320 |
| 76 | 27 | ADAVADA | 3LHI:A | 18 | 24 |
| 77 | 28 | ADDDA | 1RTQ:A | 115 | 119 |
| 78 |  | ADDDA | 3NNG:B | 208 | 212 |
| 79 | 29 | ADDDDA | 2VKN:A | 15 | 20 |
| 80 | 30 | ADDVDDA | 3SZ7:A | 242 | 248 |
| 81 | 31 | ADEDA | 2GHC:X | 226 | 230 |
| 82 |  | ADEDA | 1W98:B | 338 | 342 |
| 83 | 32 | ADFDA | 3QM9:A | 2 | 6 |
| 84 | 33 | ADGDA | 3F9S:A | 78 | 82 |
| 85 | 34 | ADKKDA | 2OKQ:B | 13 | 18 |
| 86 | 35 | ADLDA | 3PQS:A | 158 | 162 |
| 87 |  | ADLDA | 3G3Z:A | 125 | 129 |
| 88 |  | ADLDA | 3ORK:A | 245 | 249 |
| 89 |  | ADLDA | 1MDO:A | 141 | 145 |
| 90 | 36 | ADNDA | 2OSX:A | 220 | 224 |
| 91 | 37 | ADPDA | 2H0E:B | 98 | 102 |
| 92 | 38 | ADTDA | 2C0H:A | 244 | 248 |
| 93 |  | ADTDA | 3TX2:A | 10 | 14 |
| 94 | 39 | ADVDA | 2QNT:A | 77 | 81 |
| 95 | 40 | AEAEA | 1C75:A | 79 | 83 |
| 96 |  | AEAEA | 1EYQ:B | 127 | 131 |
| 97 | 41 | AEEEA | 3CB0:A | 156 | 160 |
| 98 |  | AEEEA | 2G3A:A | 85 | 89 |
| 99 | 42 | AEFEA | 3M0Z:A | 171 | 175 |
| 100 |  | AEFEA | 1WUI:L | 256 | 260 |
| 101 | 43 | AEGEA | 3M1I:B | 165 | 169 |
| 102 | 44 | AELEA | 2G9W:B | 120 | 124 |
| 103 | 45 | AENEA | 3HRQ:B | 1516 | 1520 |
| 104 | 46 | AEPEA | 2D0B:A | 194 | 198 |
| 105 | 47 | AEQEA | 2D0B:A | 58 | 62 |
| 106 | 48 | AEQKAKQEA | 1N62:E | 461 | 469 |
| 107 | 49 | AERREA | 1K0D:B | 264 | 269 |
| 108 | 50 | AETEA | 2AML:B | 196 | 200 |
| 109 | 51 | AEVEA | 2CKX:A | 583 | 587 |
| 110 |  | AEVEA | 3PMT:A | 575 | 579 |
| 111 | 52 | AEYEA | 3U3G:A | 48 | 52 |
| 112 | 53 | AFAFA | 1UG6:A | 138 | 142 |
| 113 |  | AFAFA | 2J8C:L | 120 | 124 |
| 114 | 54 | AFEFA | 1M1Q:A | 29 | 33 |
| 115 | 55 | AFGFA | 1M56:C | 190 | 194 |
| 116 | 56 | AFIFA | 3NYC:A | 1224 | 1228 |
| 117 | 57 | AFNFA | 2AS9:A | 104 | 108 |
| 118 | 58 | AFSFA | 3R9Z:A | 198 | 202 |
| 119 | 59 | AFTFA | 3M1I:B | 171 | 175 |
| 120 | 60 | AFVFA | 2BS2:F | 84 | 88 |
| 121 |  | AFVFA | 3KTA:B | 1080 | 1084 |
| 122 | 61 | AFYDYFA | 1XWT:A | 207 | 213 |
| 123 | 62 | AGAAGA | 1QOP:A | 226 | 231 |
| 124 | 63 | AGAGA | 1IFR:A | 500 | 504 |
| 125 | 64 | AGALAGA | 2OH1:A | 73 | 79 |
| 126 | 65 | AGDGA | 3QSZ:B | 121 | 125 |
| 127 | 66 | AGEEGA | 2Y0O:A | 141 | 146 |
| 128 | 67 | AGGGA | 3M5Q:A | 59 | 63 |
| 129 |  | AGGGA | 1YBX:B | 43 | 47 |
| 130 | 68 | AGHGA | 3QYJ:B | 22 | 26 |
| 131 | 69 | AGIGA | 1GKM:A | 85 | 89 |
| 132 |  | AGIGA | 2GVG:A | 204 | 208 |
| 133 | 70 | AGLGA | 3RQ5:A | 46 | 50 |
| 134 |  | AGLGA | 2AD7:A | 545 | 549 |
| 135 | 71 | AGMGA | 1O7J:C | 248 | 252 |
| 136 |  | AGMGA | 3PWK:A | 160 | 164 |
| 137 | 72 | AGNGA | 3S6L:D | 21 | 25 |
| 138 | 73 | AGQGA | 1I1W:A | 212 | 216 |
| 139 | 74 | AGSASGA | 3BF4:A | 47 | 53 |
| 140 | 75 | AGSGA | 2VFO:A | 459 | 463 |
| 141 | 76 | AGTGA | 1ESC:A | 247 | 251 |
| 142 | 77 | AGVVGA | 1QCX:A | 1 | 6 |
| 143 | 78 | AGWGA | 1PQ5:A | 136 | 140 |
| 144 | 79 | AHLHA | 1QZQ:B | 236 | 240 |
| 145 | 80 | AHNHA | 1YIS:A | 86 | 90 |
| 146 | 81 | AHVHA | 3KGZ:B | 79 | 83 |
| 147 | 82 | AIAIA | 2D0O:D | 9 | 13 |
| 148 |  | AIAIA | 1MG7:B | 328 | 332 |
| 149 | 83 | AIDIA | 3BF7:A | 111 | 115 |
| 150 |  | AIDIA | 3BD1:C | 3 | 7 |
| 151 | 84 | AIEIA | 2DVT:C | 73 | 77 |
| 152 |  | AIEIA | 3SF6:A | 342 | 346 |
| 153 |  | AIEIA | 3C24:B | 39 | 43 |
| 154 |  | AIEIA | 2I5U:A | 46 | 50 |
| 155 | 85 | AIFIA | 3U9Q:A | 385 | 389 |
| 156 | 86 | AIGIA | 2QGS:A | 130 | 134 |
| 157 | 87 | AIHIA | 1EUW:A | 56 | 60 |
| 158 | 88 | AIKIA | 2ORD:B | 101 | 105 |
| 159 | 89 | AINIA | 1RY9:D | 42 | 46 |
| 160 |  | AINIA | 1WUI:S | 144 | 148 |
| 161 |  | AINIA | 3KZP:B | 76 | 80 |
| 162 |  | AINIA | 3PKV:A | 62 | 66 |
| 163 | 90 | AISIA | 2VDU:D | 154 | 158 |
| 164 | 91 | AISVSIA | 2XZ4:A | 150 | 156 |
| 165 | 92 | AIVIA | 3EMU:A | 406 | 410 |
| 166 | 93 | AKAKA | 3P42:D | 70 | 74 |
| 167 | 94 | AKEKA | 2BW3:B | 155 | 159 |
| 168 | 95 | AKIKA | 3P0T:B | 124 | 128 |
| 169 | 96 | AKLKA | 2VU6:A | 136 | 140 |
| 170 | 97 | AKNKA | 3IVE:A | 67 | 71 |
| 171 |  | AKNKA | 3NUQ:A | 22 | 26 |
| 172 | 98 | AKQKA | 1FT5:A | 42 | 46 |
| 173 | 99 | AKTKA | 3MX7:A | 59 | 63 |
| 174 | 100 | AKVKA | 3EYP:B | 353 | 357 |
| 175 | 101 | ALAALA | 1LC5:A | 250 | 255 |
| 176 |  | ALAALA | 2RAF:C | 50 | 55 |
| 177 | 102 | ALADALA | 1SC6:A | 248 | 254 |
| 178 | 103 | ALALA | 2R31:A | 48 | 52 |
| 179 |  | ALALA | 2J8C:L | 184 | 188 |
| 180 |  | ALALA | 2GEF:A | 696 | 700 |
| 181 |  | ALALA | 2I5I:B | 143 | 147 |
| 182 |  | ALALA | 3TKT:A | 282 | 286 |
| 183 | 104 | ALDLA | 3C70:A | 35 | 39 |
| 184 |  | ALDLA | 3BT5:A | 47 | 51 |
| 185 | 105 | ALEAELA | 3OG4:A | 71 | 77 |
| 186 | 106 | ALELA | 3CPX:A | 154 | 158 |
| 187 |  | ALELA | 3ANU:A | 153 | 157 |
| 188 |  | ALELA | 2X9G:A | 189 | 193 |
| 189 | 107 | ALGLA | 2XHG:A | 266 | 270 |
| 190 |  | ALGLA | 3NW4:A | 85 | 89 |
| 191 |  | ALGLA | 2FZV:A | 25 | 29 |
| 192 | 108 | ALILA | 1ZWY:A | 166 | 170 |
| 193 | 109 | ALKLA | 256B:A | 75 | 79 |
| 194 | 110 | ALLLA | 3Q7H:N | 102 | 106 |
| 195 |  | ALLLA | 1UFO:D | 25 | 29 |
| 196 |  | ALLLA | 3RQ5:A | 30 | 34 |
| 197 |  | ALLLA | 3FXH:A | 95 | 99 |
| 198 |  | ALLLA | 3NVS:A | 28 | 32 |
| 199 | 111 | ALNLA | 1XMK:A | 315 | 319 |
| 200 |  | ALNLA | 3HUL:B | 60 | 64 |
| 201 | 112 | ALPLA | 3QIT:A | 43 | 47 |
| 202 |  | ALPLA | 1YRE:A | 68 | 72 |
| 203 |  | ALPLA | 2PD1:D | 27 | 31 |
| 204 | 113 | ALQLA | 3BY4:A | 249 | 253 |
| 205 |  | ALQLA | 2NT0:D | 164 | 168 |
| 206 |  | ALQLA | 3EAT:X | 147 | 151 |
| 207 |  | ALQLA | 3ELK:B | 95 | 99 |
| 208 | 114 | ALSLA | 3M73:A | 22 | 26 |
| 209 | 115 | ALTLA | 1X2I:A | 2 | 6 |
| 210 |  | ALTLA | 3C8G:D | 155 | 159 |
| 211 |  | ALTLA | 2Y2Z:A | 99 | 103 |
| 212 | 116 | ALVLA | 1R6D:A | 228 | 232 |
| 213 |  | ALVLA | 3DR5:A | 158 | 162 |
| 214 | 117 | ALWLA | 3UUW:D | 172 | 176 |
| 215 | 118 | AMEMA | 2VE8:E | 785 | 789 |
| 216 | 119 | AMTMA | 3HLX:A | 224 | 228 |
| 217 | 120 | AMVMA | 1T61:A | 115 | 119 |
| 218 | 121 | AMYEYMA | 1F0L:B | 177 | 183 |
| 219 | 122 | AMYMA | 4DEM:F | 227 | 231 |
| 220 | 123 | ANANA | 3MWZ:A | 84 | 88 |
| 221 | 124 | ANEKVKENA | 1V4P:A | 62 | 70 |
| 222 | 125 | ANENA | 3V8H:A | 84 | 88 |
| 223 |  | ANENA | 1SVF:C | 130 | 134 |
| 224 | 126 | ANFNA | 3LED:B | 32 | 36 |
| 225 | 127 | ANGNA | 2ADV:B | 12 | 16 |
| 226 | 128 | ANINA | 3IU0:A | 24 | 28 |
| 227 | 129 | ANKNA | 1DM1:A | 19 | 23 |
| 228 | 130 | ANSNA | 1XG0:C | 124 | 128 |
| 229 | 131 | ANTNA | 1Y7T:B | 132 | 136 |
| 230 | 132 | ANVNA | 2E6F:A | 231 | 235 |
| 231 | 133 | ANYNA | 1OC7:A | 309 | 313 |
| 232 | 134 | APDPA | 2HYK:A | 22 | 26 |
| 233 | 135 | APHPA | 1EJD:A | 297 | 301 |
| 234 | 136 | APIPA | 2X9Z:A | 357 | 361 |
| 235 | 137 | APLPA | 3FCN:A | 138 | 142 |
| 236 | 138 | APMPA | 1N4W:A | 363 | 367 |
| 237 | 139 | APRPA | 3GWR:A | 99 | 103 |
| 238 |  | APRPA | 1K66:B | 60 | 64 |
| 239 | 140 | APSPA | 1MAI:A | 112 | 116 |
| 240 |  | APSPA | 3RPW:A | 339 | 343 |
| 241 | 141 | AQDQA | 1EB6:A | 119 | 123 |
| 242 | 142 | AQIIQA | 2VWR:A | 404 | 409 |
| 243 | 143 | AQKQA | 1NOX:A | 111 | 115 |
| 244 | 144 | AQLALQA | 3S0R:B | 17 | 23 |
| 245 | 145 | AQLQA | 1O7Q:A | 244 | 248 |
| 246 | 146 | AQMQA | 3OK8:A | 163 | 167 |
| 247 | 147 | AQPQA | 2B1Y:A | 93 | 97 |
| 248 | 148 | AQSQA | 3LJK:A | 417 | 421 |
| 249 | 149 | AQWQA | 1S9U:A | 187 | 191 |
| 250 | 150 | ARERA | 1B0U:A | 129 | 133 |
| 251 | 151 | ARFRA | 2BK9:A | 56 | 60 |
| 252 | 152 | ARGQGRA | 3SX2:H | 20 | 26 |
| 253 | 153 | ARIIRA | 2D0B:A | 277 | 282 |
| 254 | 154 | ARIRA | 3HIM:A | 19 | 23 |
| 255 | 155 | ARKKRA | 1XQO:A | 104 | 109 |
| 256 | 156 | ARLRA | 3LFP:A | 9 | 13 |
| 257 | 157 | ARNRA | 1ZVT:A | 613 | 617 |
| 258 | 158 | ARPLPRA | 1B3A:A | 16 | 22 |
| 259 | 159 | ARQRA | 2I9D:C | 38 | 42 |
| 260 | 160 | ARRRA | 2BFF:A | 268 | 272 |
| 261 |  | ARRRA | 3D3B:A | 5 | 9 |
| 262 | 161 | ARVRA | 1IAR:B | 174 | 178 |
| 263 |  | ARVRA | 1XOD:A | 12 | 16 |
| 264 | 162 | ASAASA | 3OZY:A | 292 | 297 |
| 265 | 163 | ASALASA | 1QOP:B | 118 | 124 |
| 266 | 164 | ASASA | 3CKM:A | 466 | 470 |
| 267 |  | ASASA | 2OB3:B | 266 | 270 |
| 268 |  | ASASA | 3LFP:A | 29 | 33 |
| 269 |  | ASASA | 3AKH:A | 227 | 231 |
| 270 | 165 | ASDSA | 2HNF:A | 136 | 140 |
| 271 | 166 | ASESA | 3N79:A | 154 | 158 |
| 272 | 167 | ASGGSA | 1APY:A | 25 | 30 |
| 273 | 168 | ASGSA | 1GR3:A | 636 | 640 |
| 274 | 169 | ASISA | 2ZAY:A | 23 | 27 |
| 275 |  | ASISA | 2VE8:E | 763 | 767 |
| 276 | 170 | ASLLSA | 2HBG:A | 108 | 113 |
| 277 | 171 | ASLSA | 3BEM:A | 135 | 139 |
| 278 |  | ASLSA | 2WQ4:B | 121 | 125 |
| 279 | 172 | ASNSA | 3S46:B | 200 | 204 |
| 280 | 173 | ASRSA | 2WNK:A | 192 | 196 |
| 281 | 174 | ASTSA | 1DEU:A | 35 | 39 |
| 282 | 175 | ASWSA | 1UAI:A | 86 | 90 |
| 283 | 176 | ASYSA | 1W7C:A | 456 | 460 |
| 284 | 177 | ATAAATA | 1L3P:A | 169 | 175 |
| 285 | 178 | ATADATA | 1OQV:A | 59 | 65 |
| 286 | 179 | ATATA | 3BB7:A | 156 | 160 |
| 287 |  | ATATA | 3MK1:A | 94 | 98 |
| 288 |  | ATATA | 1N2F:A | 10 | 14 |
| 289 | 180 | ATFTA | 1R9L:A | 58 | 62 |
| 290 | 181 | ATITA | 1UAS:A | 311 | 315 |
| 291 | 182 | ATKKTA | 2I5V:O | 209 | 214 |
| 292 |  | ATKKTA | 3GFP:A | 338 | 343 |
| 293 | 183 | ATLTA | 1RA0:A | 128 | 132 |
| 294 |  | ATLTA | 1NKZ:F | 1 | 5 |
| 295 |  | ATLTA | 3AWU:A | 8 | 12 |
| 296 | 184 | ATNTA | 3D3B:A | 56 | 60 |
| 297 | 185 | ATQQTA | 2AIB:A | 5 | 10 |
| 298 | 186 | ATRTA | 1Q1F:A | 128 | 132 |
| 299 | 187 | ATTTA | 2XFG:A | 320 | 324 |
| 300 |  | ATTTA | 2NPT:A | 55 | 59 |
| 301 | 188 | ATVTA | 1RTQ:A | 8 | 12 |
| 302 |  | ATVTA | 2E4T:A | 36 | 40 |
| 303 | 189 | ATYTA | 2HDS:B | 110 | 114 |
| 304 | 190 | AVAVA | 3S46:B | 48 | 52 |
| 305 |  | AVAVA | 2WAS:B | 1878 | 1882 |
| 306 | 191 | AVCVA | 3MSX:B | 510 | 514 |
| 307 | 192 | AVDVA | 1NH9:A | 41 | 45 |
| 308 |  | AVDVA | 1UG6:A | 230 | 234 |
| 309 |  | AVDVA | 3FMF:D | 128 | 132 |
| 310 | 193 | AVEVA | 1Z9W:A | 92 | 96 |
| 311 |  | AVEVA | 3G3L:A | 156 | 160 |
| 312 |  | AVEVA | 3ALN:C | 374 | 378 |
| 313 | 194 | AVGGVA | 3GQH:A | 820 | 825 |
| 314 | 195 | AVGVA | 2WF7:A | 157 | 161 |
| 315 | 196 | AVKVA | 2JHF:A | 183 | 187 |
| 316 |  | AVKVA | 1US0:A | 30 | 34 |
| 317 |  | AVKVA | 2Y2Z:A | 36 | 40 |
| 318 |  | AVKVA | 3JWI:A | 175 | 179 |
| 319 | 197 | AVLVA | 2YLN:A | 138 | 142 |
| 320 |  | AVLVA | 2Q30:B | 81 | 85 |
| 321 | 198 | AVNVA | 1SU7:A | 253 | 257 |
| 322 |  | AVNVA | 1F0L:B | 282 | 286 |
| 323 | 199 | AVPVA | 2ZAY:A | 44 | 48 |
| 324 |  | AVPVA | 1VGW:E | 137 | 141 |
| 325 | 200 | AVQVA | 3ERJ:B | 21 | 25 |
| 326 | 201 | AVRVA | 1O7J:C | 138 | 142 |
| 327 |  | AVRVA | 3BB0:A | 260 | 264 |
| 328 | 202 | AVSVA | 2QTV:D | 938 | 942 |
| 329 | 203 | AVTVA | 3N79:A | 150 | 154 |
| 330 | 204 | AVVVA | 3TDN:B | 3 | 7 |
| 331 | 205 | AYAAYA | 3CLM:A | 173 | 178 |
| 332 | 206 | AYAYA | 4DFA:A | 90 | 94 |
| 333 |  | AYAYA | 1K52:A | 33 | 37 |
| 334 | 207 | AYFYA | 2QIY:B | 28 | 32 |
| 335 | 208 | AYGYA | 2XJP:A | 61 | 65 |
| 336 |  | AYGYA | 1F61:A | 362 | 366 |
| 337 | 209 | AYLYA | 1DQP:B | 72 | 76 |
| 338 | 210 | AYQQYA | 3HA2:A | 159 | 164 |
| 339 | 211 | CAKAC | 2W6P:B | 260 | 264 |
| 340 | 212 | CCSCC | 4MT2:A | 33 | 37 |
| 341 | 213 | CEGEC | 2ARP:A | 40 | 44 |
| 342 |  | CEGEC | 3ZWF:A | 28 | 32 |
| 343 | 214 | CEPEC | 2FDN:A | 14 | 18 |
| 344 |  | CEPEC | 7FD1:A | 45 | 49 |
| 345 | 215 | CFTFC | 3NO8:B | 498 | 502 |
| 346 | 216 | CGLGC | 3QSD:A | 137 | 141 |
| 347 | 217 | CHNHC | 3E7R:L | 15 | 19 |
| 348 | 218 | CISIC | 2WY3:D | 60 | 64 |
| 349 | 219 | CLFLC | 1LC5:A | 149 | 153 |
| 350 | 220 | CLRLC | 2Q0I:A | 241 | 245 |
| 351 | 221 | CMGMC | 2Z7F:I | 93 | 97 |
| 352 | 222 | CPRPC | 1P9G:A | 7 | 11 |
| 353 | 223 | CQCQC | 1RJU:V | 3 | 7 |
| 354 | 224 | CRIRC | 1BX7:A | 29 | 33 |
| 355 | 225 | CSASC | 2V33:B | 376 | 380 |
| 356 | 226 | CSGSC | 1AGQ:C | 69 | 73 |
| 357 |  | CSGSC | 2ASK:B | 43 | 47 |
| 358 | 227 | CVEVC | 7FD1:A | 16 | 20 |
| 359 | 228 | CVMKMVC | 3DK9:A | 417 | 423 |
| 360 | 229 | CVNVC | 1XER:A | 89 | 93 |
| 361 | 230 | CYMYC | 3CZ1:A | 47 | 51 |
| 362 | 231 | DACAD | 1N62:E | 343 | 347 |
| 363 | 232 | DADAD | 2F5V:A | 187 | 191 |
| 364 | 233 | DAFAD | 1I4D:A | 70 | 74 |
| 365 |  | DAFAD | 2PD1:D | 54 | 58 |
| 366 | 234 | DAFFAD | 2GHC:X | 229 | 234 |
| 367 | 235 | DAGAD | 3QU5:B | 202 | 206 |
| 368 |  | DAGAD | 3BMX:A | 260 | 264 |
| 369 |  | DAGAD | 1QOP:A | 42 | 46 |
| 370 | 236 | DAHAD | 3V8H:A | 120 | 124 |
| 371 | 237 | DAIAD | 1NF8:A | 175 | 179 |
| 372 |  | DAIAD | 2WF7:A | 133 | 137 |
| 373 | 238 | DAKAD | 1VK1:A | 184 | 188 |
| 374 | 239 | DALAD | 1OFW:B | 184 | 188 |
| 375 |  | DALAD | 1MSK:A | 1070 | 1074 |
| 376 | 240 | DANAD | 1GA6:A | 140 | 144 |
| 377 | 241 | DATAD | 3RYC:B | 437 | 441 |
| 378 |  | DATAD | 2GWM:A | 572 | 576 |
| 379 | 242 | DAVAD | 3LHI:A | 19 | 23 |
| 380 | 243 | DDADADD | 2F5V:A | 186 | 192 |
| 381 | 244 | DDIDD | 2OX0:B | 60 | 64 |
| 382 | 245 | DDTDD | 2OB3:B | 232 | 236 |
| 383 | 246 | DDVDD | 3SZ7:A | 243 | 247 |
| 384 | 247 | DDYDD | 2I15:A | 45 | 49 |
| 385 | 248 | DEEED | 1O8X:A | 71 | 75 |
| 386 | 249 | DEFED | 3QJG:L | 58 | 62 |
| 387 | 250 | DEIED | 2R5O:B | 267 | 271 |
| 388 | 251 | DEKED | 2F5V:A | 558 | 562 |
| 389 | 252 | DELED | 3VAA:B | 154 | 158 |
| 390 | 253 | DENED | 2C5K:T | 65 | 69 |
| 391 | 254 | DEPED | 1HBN:F | 60 | 64 |
| 392 | 255 | DFAFD | 1MSK:A | 913 | 917 |
| 393 |  | DFAFD | 3E8T:A | 46 | 50 |
| 394 | 256 | DFLKLFD | 1VJN:A | 176 | 182 |
| 395 | 257 | DFLQLFD | 1HQZ:4 | 54 | 60 |
| 396 | 258 | DFTFD | 3DY0:A | 28 | 32 |
| 397 | 259 | DFVVFD | 3MH9:C | 103 | 108 |
| 398 | 260 | DFYFD | 2IMF:A | 4 | 8 |
| 399 | 261 | DGAKAGD | 1HBN:F | 74 | 80 |
| 400 | 262 | DGDGD | 2NR7:A | 46 | 50 |
| 401 | 263 | DGDKDGD | 2PVB:A | 88 | 94 |
| 402 | 264 | DGKGD | 1UX6:A | 870 | 874 |
| 403 | 265 | DGRGD | 1UX6:A | 906 | 910 |
| 404 |  | DGRGD | 2QEB:A | 49 | 53 |
| 405 |  | DGRGD | 3DAS:A | 329 | 333 |
| 406 | 266 | DGTGD | 1VCL:A | 126 | 130 |
| 407 | 267 | DIGID | 3EA6:A | 3 | 7 |
| 408 |  | DIGID | 3AJD:A | 195 | 199 |
| 409 | 268 | DILID | 1IQZ:A | 48 | 52 |
| 410 |  | DILID | 3FCX:A | 217 | 221 |
| 411 | 269 | DIRID | 2E4T:A | 11 | 15 |
| 412 | 270 | DISID | 2XJP:A | 78 | 82 |
| 413 |  | DISID | 3KNB:A | 14 | 18 |
| 414 | 271 | DKEKD | 3OQ2:A | 66 | 70 |
| 415 | 272 | DKIKD | 2P4F:A | 108 | 112 |
| 416 |  | DKIKD | 2RDG:A | 55 | 59 |
| 417 | 273 | DKNKD | 1S6C:A | 183 | 187 |
| 418 | 274 | DKSKD | 2HF1:A | 21 | 25 |
| 419 | 275 | DKTKD | 1JXO:B | 440 | 444 |
| 420 | 276 | DKVKD | 2RK5:A | 73 | 77 |
| 421 | 277 | DKVSVKD | 2QIF:B | 49 | 55 |
| 422 | 278 | DKYKD | 3SEB:A | 75 | 79 |
| 423 | 279 | DLAALD | 3SZY:A | 366 | 371 |
| 424 | 280 | DLCLD | 3FUY:C | 42 | 46 |
| 425 |  | DLCLD | 1VCL:A | 164 | 168 |
| 426 | 281 | DLDLD | 1VR9:B | 55 | 59 |
| 427 | 282 | DLELD | 3MYU:B | 121 | 125 |
| 428 | 283 | DLFLD | 3ESL:A | 59 | 63 |
| 429 | 284 | DLILD | 1QLW:B | 311 | 315 |
| 430 | 285 | DLKLD | 1US0:A | 98 | 102 |
| 431 | 286 | DLKVKLD | 2IMF:A | 55 | 61 |
| 432 | 287 | DLLLD | 1JKV:F | 57 | 61 |
| 433 | 288 | DLPLD | 3F0D:D | 123 | 127 |
| 434 |  | DLPLD | 3V2U:D | 100 | 104 |
| 435 | 289 | DLSLD | 2OFY:B | 68 | 72 |
| 436 | 290 | DLVLD | 1JW9:B | 123 | 127 |
| 437 |  | DLVLD | 1G60:B | 214 | 218 |
| 438 | 291 | DMFMD | 1N7S:B | 214 | 218 |
| 439 | 292 | DMMMD | 2FFU:A | 325 | 329 |
| 440 | 293 | DMVVMD | 2YFO:A | 361 | 366 |
| 441 | 294 | DNCND | 1UAS:A | 130 | 134 |
| 442 | 295 | DNLND | 3PCT:C | 180 | 184 |
| 443 |  | DNLND | 3DAO:A | 216 | 220 |
| 444 |  | DNLND | 1JLY:B | 191 | 195 |
| 445 | 296 | DNPRPND | 3B7E:A | 324 | 330 |
| 446 | 297 | DNSND | 3DA5:A | 62 | 66 |
| 447 | 298 | DNYND | 1AYM:1 | 86 | 90 |
| 448 | 299 | DPAPD | 2QE8:B | 137 | 141 |
| 449 | 300 | DPKPD | 3SEB:A | 5 | 9 |
| 450 | 301 | DPLGLPD | 3BI1:A | 677 | 683 |
| 451 | 302 | DPLPD | 7FD1:A | 86 | 90 |
| 452 | 303 | DPNPD | 1Z2U:A | 112 | 116 |
| 453 | 304 | DPWPD | 1SJW:A | 134 | 138 |
| 454 | 305 | DPYPD | 3NDI:A | 373 | 377 |
| 455 | 306 | DQCQD | 3M7P:A | 559 | 563 |
| 456 | 307 | DQMMQD | 1KGD:A | 789 | 794 |
| 457 | 308 | DQPQD | 2GDQ:B | 223 | 227 |
| 458 | 309 | DQTQD | 1K5N:A | 223 | 227 |
| 459 | 310 | DRARD | 3G2B:A | 21 | 25 |
| 460 | 311 | DRIRD | 2Y4S:A | 541 | 545 |
| 461 | 312 | DRLRD | 3QWW:A | 252 | 256 |
| 462 | 313 | DRSRD | 3B1F:A | 38 | 42 |
| 463 | 314 | DSDNDSD | 3N1F:C | 870 | 876 |
| 464 | 315 | DSDSD | 1UX6:A | 828 | 832 |
| 465 | 316 | DSFSD | 2Y4S:A | 249 | 253 |
| 466 | 317 | DSISD | 3KZX:A | 164 | 168 |
| 467 | 318 | DSKSD | 4A4Y:A | 60 | 64 |
| 468 |  | DSKSD | 3TYT:A | 547 | 551 |
| 469 | 319 | DSLSD | 1X6I:B | 233 | 237 |
| 470 | 320 | DSRSD | 2WHL:A | 94 | 98 |
| 471 |  | DSRSD | 3I26:B | 36 | 40 |
| 472 | 321 | DSSSD | 2X27:X | 23 | 27 |
| 473 |  | DSSSD | 1K3I:A | 254 | 258 |
| 474 | 322 | DSWSD | 1GWE:A | 394 | 398 |
| 475 | 323 | DTETD | 2WCJ:A | 102 | 106 |
| 476 | 324 | DTLTD | 2WZ1:A | 458 | 462 |
| 477 |  | DTLTD | 3MM1:A | 295 | 299 |
| 478 | 325 | DTRSRTD | 3RL5:A | 65 | 71 |
| 479 | 326 | DTSTD | 2FUR:A | 170 | 174 |
| 480 | 327 | DTTTD | 3CM3:A | 1082 | 1086 |
| 481 | 328 | DTVTD | 3PQS:A | 71 | 75 |
| 482 |  | DTVTD | 3SE2:A | 1690 | 1694 |
| 483 | 329 | DTYTD | 1N7O:A | 172 | 176 |
| 484 |  | DTYTD | 3BVP:B | 43 | 47 |
| 485 | 330 | DVAVD | 1F00:I | 738 | 742 |
| 486 |  | DVAVD | 3FMF:D | 126 | 130 |
| 487 | 331 | DVDVD | 2X27:X | 194 | 198 |
| 488 | 332 | DVIVD | 3RYK:B | 83 | 87 |
| 489 | 333 | DVKVD | 3DB2:A | 291 | 295 |
| 490 | 334 | DVLVD | 1ZPS:B | 81 | 85 |
| 491 |  | DVLVD | 2X3H:C | 268 | 272 |
| 492 | 335 | DVPVD | 2I53:A | 241 | 245 |
| 493 | 336 | DVQVD | 2XFD:A | 60 | 64 |
| 494 | 337 | DVRVD | 1ZHV:A | 56 | 60 |
| 495 | 338 | DVVVD | 3EPW:A | 282 | 286 |
| 496 |  | DVVVD | 2R9G:P | 319 | 323 |
| 497 |  | DVVVD | 2G9Z:B | 185 | 189 |
| 498 | 339 | DVYVD | 1U58:A | 212 | 216 |
| 499 | 340 | DWEEWD | 1SAU:A | 21 | 26 |
| 500 | 341 | DYAYD | 1Y43:B | 47 | 51 |
| 501 | 342 | DYGYD | 2WJ6:D | 179 | 183 |
| 502 | 343 | DYKYD | 1KWF:A | 274 | 278 |
| 503 | 344 | DYNNYD | 3EDO:B | 72 | 77 |
| 504 | 345 | EAAAE | 3ME5:A | 155 | 159 |
| 505 | 346 | EAAEAAE | 3A9S:C | 72 | 78 |
| 506 | 347 | EAAVAAE | 2IBP:B | 114 | 120 |
| 507 | 348 | EACAE | 2XMJ:A | 13 | 17 |
| 508 | 349 | EAFAE | 1ZMT:A | 42 | 46 |
| 509 |  | EAFAE | 1MSK:A | 1097 | 1101 |
| 510 |  | EAFAE | 2B06:A | 49 | 53 |
| 511 | 350 | EAGAE | 1U83:A | 161 | 165 |
| 512 | 351 | EAIAE | 2ZKM:X | 389 | 393 |
| 513 |  | EAIAE | 1PBJ:A | 54 | 58 |
| 514 |  | EAIAE | 4A8X:B | 678 | 682 |
| 515 | 352 | EAKAE | 1AY7:B | 76 | 80 |
| 516 | 353 | EARAE | 1LYV:A | 192 | 196 |
| 517 |  | EARAE | 3U52:C | 312 | 316 |
| 518 |  | EARAE | 2PC1:A | 20 | 24 |
| 519 | 354 | EASAE | 2X9G:A | 231 | 235 |
| 520 | 355 | EAVAE | 2AA3:A | 317 | 321 |
| 521 |  | EAVAE | 2Q3P:A | 75 | 79 |
| 522 |  | EAVAE | 2OKG:B | 154 | 158 |
| 523 | 356 | EAWAE | 3I94:A | 1232 | 1236 |
| 524 | 357 | EDADE | 3O1C:A | 67 | 71 |
| 525 | 358 | EDDDE | 1QHH:D | 592 | 596 |
| 526 |  | EDDDE | 1T0I:B | 54 | 58 |
| 527 | 359 | EDEDE | 2IU1:A | 395 | 399 |
| 528 | 360 | EDIDE | 1UZK:A | 1605 | 1609 |
| 529 | 361 | EDSDE | 1K66:B | 13 | 17 |
| 530 | 362 | EDTDE | 2ZDP:A | 46 | 50 |
| 531 | 363 | EDVDE | 1M2D:A | 80 | 84 |
| 532 | 364 | EEAEE | 1H7C:A | 89 | 93 |
| 533 |  | EEAEE | 2Q00:B | 12 | 16 |
| 534 | 365 | EEDEE | 3M1I:B | 80 | 84 |
| 535 | 366 | EEFEE | 3NRV:D | 121 | 125 |
| 536 | 367 | EEGEE | 1FEW:A | 167 | 171 |
| 537 |  | EEGEE | 2CWZ:D | 56 | 60 |
| 538 |  | EEGEE | 3BT2:U | 33 | 37 |
| 539 | 368 | EEIEE | 2GJ4:A | 123 | 127 |
| 540 | 369 | EEKEE | 3A1Q:C | 103 | 107 |
| 541 |  | EEKEE | 1FGY:A | 366 | 370 |
| 542 | 370 | EELEE | 2NOG:A | 813 | 817 |
| 543 |  | EELEE | 1JKV:F | 190 | 194 |
| 544 |  | EELEE | 2PIH:A | 80 | 84 |
| 545 |  | EELEE | 2GJ4:A | 120 | 124 |
| 546 |  | EELEE | 1J7D:A | 17 | 21 |
| 547 | 371 | EENEE | 2OKQ:B | 61 | 65 |
| 548 |  | EENEE | 3AJD:A | 217 | 221 |
| 549 |  | EENEE | 2BKF:A | 51 | 55 |
| 550 | 372 | EEQEE | 3MK6:C | 37 | 41 |
| 551 | 373 | EEREE | 1UNQ:A | 94 | 98 |
| 552 | 374 | EESEE | 1WPA:A | 469 | 473 |
| 553 |  | EESEE | 1Z6M:A | 44 | 48 |
| 554 | 375 | EEVEE | 1S7Z:A | 106 | 110 |
| 555 |  | EEVEE | 1UGI:B | 27 | 31 |
| 556 |  | EEVEE | 3U23:A | 139 | 143 |
| 557 |  | EEVEE | 2ORD:B | 293 | 297 |
| 558 | 376 | EEYEE | 2YC3:A | 138 | 142 |
| 559 |  | EEYEE | 3CZQ:A | 58 | 62 |
| 560 | 377 | EFFFE | 3OJN:D | 127 | 131 |
| 561 | 378 | EFLFE | 2W6P:B | 276 | 280 |
| 562 | 379 | EFTFE | 3ZY7:B | 16 | 20 |
| 563 |  | EFTFE | 3OBH:B | 11 | 15 |
| 564 | 380 | EFVFE | 3US6:A | 40 | 44 |
| 565 | 381 | EGAGE | 1I1K:C | 1193 | 1197 |
| 566 | 382 | EGEGE | 3NED:A | 30 | 34 |
| 567 |  | EGEGE | 2Q30:B | 62 | 66 |
| 568 | 383 | EGFGE | 3QT9:A | 279 | 283 |
| 569 | 384 | EGIGE | 1X2I:A | 49 | 53 |
| 570 | 385 | EGKGE | 3H09:B | 463 | 467 |
| 571 | 386 | EGPGE | 1UI0:A | 41 | 45 |
| 572 | 387 | EGSSGE | 1LBU:A | 12 | 17 |
| 573 | 388 | EGTGE | 3ESS:A | 509 | 513 |
| 574 | 389 | EGYGE | 3KL2:L | 101 | 105 |
| 575 |  | EGYGE | 3I1A:B | 1265 | 1269 |
| 576 | 390 | EGYIYGE | 2QIK:A | 54 | 60 |
| 577 | 391 | EHHHHHE | 3TGN:A | 107 | 113 |
| 578 | 392 | EHSHE | 3KA8:A | 53 | 57 |
| 579 | 393 | EIAAIE | 1BB1:C | 23 | 28 |
| 580 | 394 | EIDIE | 2UWA:A | 94 | 98 |
| 581 | 395 | EIFIE | 3MYU:B | 326 | 330 |
| 582 | 396 | EIHIE | 2H98:A | 118 | 122 |
| 583 | 397 | EIIIE | 2OKF:A | 115 | 119 |
| 584 |  | EIIIE | 1H72:C | 45 | 49 |
| 585 | 398 | EIKIE | 1WWZ:B | 3 | 7 |
| 586 | 399 | EILIE | 2JE6:I | 205 | 209 |
| 587 |  | EILIE | 2FP8:B | 33 | 37 |
| 588 |  | EILIE | 1R7A:B | 228 | 232 |
| 589 |  | EILIE | 2BWQ:A | 833 | 837 |
| 590 | 400 | EISIE | 2XFR:A | 461 | 465 |
| 591 | 401 | EITIE | 2EB4:B | 33 | 37 |
| 592 | 402 | EIVGVIE | 3QY9:D | 25 | 31 |
| 593 | 403 | EKDKE | 2HD9:A | 48 | 52 |
| 594 | 404 | EKEKE | 2P64:B | 131 | 135 |
| 595 |  | EKEKE | 1VHF:A | 60 | 64 |
| 596 | 405 | EKFKE | 1DZK:B | 130 | 134 |
| 597 | 406 | EKGKE | 1ORJ:D | 4073 | 4077 |
| 598 |  | EKGKE | 2J8C:L | 201 | 205 |
| 599 |  | EKGKE | 3GMI:A | 71 | 75 |
| 600 | 407 | EKHKE | 2R4G:A | 480 | 484 |
| 601 | 408 | EKIKE | 3N72:B | 136 | 140 |
| 602 |  | EKIKE | 3TC7:A | 241 | 245 |
| 603 |  | EKIKE | 2R5X:A | 115 | 119 |
| 604 |  | EKIKE | 3IJD:B | 28 | 32 |
| 605 |  | EKIKE | 3U02:C | 67 | 71 |
| 606 | 409 | EKLKE | 1NQJ:B | 895 | 899 |
| 607 |  | EKLKE | 2AEB:B | 38 | 42 |
| 608 |  | EKLKE | 1QOP:A | 198 | 202 |
| 609 |  | EKLKE | 2ANU:A | 127 | 131 |
| 610 |  | EKLKE | 1W9M:A | 46 | 50 |
| 611 |  | EKLKE | 3AOW:C | 185 | 189 |
| 612 |  | EKLKE | 3SE8:G | 347 | 351 |
| 613 | 410 | EKPKE | 1VJN:A | 195 | 199 |
| 614 |  | EKPKE | 3C6A:A | 385 | 389 |
| 615 | 411 | EKRKE | 1ORJ:D | 4047 | 4051 |
| 616 |  | EKRKE | 3EDV:B | 1997 | 2001 |
| 617 |  | EKRKE | 2ZPT:X | 181 | 185 |
| 618 | 412 | EKSKE | 1FYH:E | 212 | 216 |
| 619 |  | EKSKE | 2O1M:B | 85 | 89 |
| 620 | 413 | EKSSKE | 3L4Q:D | 493 | 498 |
| 621 | 414 | EKSTSKE | 1TVG:A | 75 | 81 |
| 622 | 415 | EKVKE | 1V4P:A | 64 | 68 |
| 623 |  | EKVKE | 1P5Z:B | 251 | 255 |
| 624 |  | EKVKE | 1QGQ:A | 170 | 174 |
| 625 |  | EKVKE | 2G1U:A | 131 | 135 |
| 626 |  | EKVKE | 8A3H:A | 80 | 84 |
| 627 | 416 | ELAALE | 2V14:A | 1237 | 1242 |
| 628 | 417 | ELALE | 2IG6:A | 87 | 91 |
| 629 |  | ELALE | 3OAB:C | 237 | 241 |
| 630 |  | ELALE | 1EJ0:A | 145 | 149 |
| 631 | 418 | ELELE | 3BGY:A | 210 | 214 |
| 632 |  | ELELE | 2FPE:A | 17 | 21 |
| 633 | 419 | ELGLE | 1I24:A | 344 | 348 |
| 634 |  | ELGLE | 1G2R:A | 93 | 97 |
| 635 |  | ELGLE | 2GFQ:C | 266 | 270 |
| 636 | 420 | ELHLE | 2AXW:B | 2 | 6 |
| 637 | 421 | ELIKILE | 2ZP1:A | 294 | 300 |
| 638 | 422 | ELKLE | 1VKK:A | 56 | 60 |
| 639 | 423 | ELLLLE | 2Z72:A | 314 | 319 |
| 640 | 424 | ELNLE | 1S5P:A | 238 | 242 |
| 641 |  | ELNLE | 3H8D:D | 1208 | 1212 |
| 642 | 425 | ELPLE | 3P1V:B | 66 | 70 |
| 643 |  | ELPLE | 3NO0:C | 511 | 515 |
| 644 | 426 | ELQLE | 3AEI:B | 8 | 12 |
| 645 |  | ELQLE | 1V9Y:B | 98 | 102 |
| 646 | 427 | ELRLE | 3MMY:B | 202 | 206 |
| 647 |  | ELRLE | 1WWC:A | 314 | 318 |
| 648 | 428 | ELSLE | 1OJH:L | 6 | 10 |
| 649 | 429 | ELTLE | 2VQC:A | 23 | 27 |
| 650 | 430 | EMAME | 2GN4:A | 51 | 55 |
| 651 | 431 | ENENE | 3HE5:A | 14 | 18 |
| 652 | 432 | ENGNE | 1T4W:A | 384 | 388 |
| 653 | 433 | ENINE | 1ZB1:B | 134 | 138 |
| 654 | 434 | ENLLNE | 3NPK:A | 252 | 257 |
| 655 | 435 | ENLNE | 2B0A:A | 148 | 152 |
| 656 | 436 | ENNNE | 2UY2:A | 157 | 161 |
| 657 | 437 | ENQNE | 2BNL:F | 13 | 17 |
| 658 | 438 | ENVNE | 3FLE:B | 184 | 188 |
| 659 | 439 | EPAPE | 2Q4I:A | 174 | 178 |
| 660 | 440 | EPCPE | 2Y27:A | 375 | 379 |
| 661 |  | EPCPE | 2AG4:B | 94 | 98 |
| 662 | 441 | EPGPE | 3Q46:A | 8 | 12 |
| 663 | 442 | EPIPE | 1N67:A | 555 | 559 |
| 664 | 443 | EPLPE | 1W9M:A | 112 | 116 |
| 665 |  | EPLPE | 3IJM:A | 49 | 53 |
| 666 | 444 | EPPPE | 2CXY:A | 108 | 112 |
| 667 |  | EPPPE | 3SLZ:B | 11 | 15 |
| 668 |  | EPPPE | 2GHC:X | 124 | 128 |
| 669 | 445 | EPTFTPE | 1JFB:A | 102 | 108 |
| 670 | 446 | EPTPE | 1JR8:B | 32 | 36 |
| 671 |  | EPTPE | 3KNB:A | 32 | 36 |
| 672 | 447 | EQAQE | 2VXN:A | 181 | 185 |
| 673 | 448 | EQIQE | 2FP8:B | 309 | 313 |
| 674 |  | EQIQE | 2EA7:A | 210 | 214 |
| 675 | 449 | EQKAKQE | 1N62:E | 462 | 468 |
| 676 | 450 | EQLQE | 3EFY:B | 139 | 143 |
| 677 | 451 | EQMQE | 1ZWX:A | 204 | 208 |
| 678 |  | EQMQE | 3SE8:G | 102 | 106 |
| 679 | 452 | EQPQE | 3NRE:A | 213 | 217 |
| 680 | 453 | ERERE | 3PWT:A | 206 | 210 |
| 681 | 454 | ERFRE | 2D37:A | 86 | 90 |
| 682 |  | ERFRE | 1RTT:A | 64 | 68 |
| 683 |  | ERFRE | 3EDV:B | 1738 | 1742 |
| 684 | 455 | ERGRE | 1QSA:A | 331 | 335 |
| 685 | 456 | ERIRE | 3FPN:B | 222 | 226 |
| 686 |  | ERIRE | 3N0U:A | 9 | 13 |
| 687 |  | ERIRE | 1MB3:A | 92 | 96 |
| 688 | 457 | ERLRE | 3BMX:A | 245 | 249 |
| 689 |  | ERLRE | 3H3M:B | 58 | 62 |
| 690 | 458 | ERMRE | 3ANU:A | 27 | 31 |
| 691 | 459 | ERTRE | 1F61:A | 252 | 256 |
| 692 | 460 | ERVRE | 2XOD:A | 111 | 115 |
| 693 |  | ERVRE | 2J45:B | 53 | 57 |
| 694 |  | ERVRE | 3OLJ:A | 287 | 291 |
| 695 |  | ERVRE | 1T3U:D | 82 | 86 |
| 696 | 461 | ERWRE | 2WJ6:D | 147 | 151 |
| 697 | 462 | ERYRE | 1T1D:A | 157 | 161 |
| 698 | 463 | ESASE | 3R5G:B | 364 | 368 |
| 699 | 464 | ESLSE | 1JKG:A | 56 | 60 |
| 700 | 465 | ESSSE | 2C0H:A | 186 | 190 |
| 701 | 466 | ESTETSE | 1QHV:A | 541 | 547 |
| 702 | 467 | ESTTSE | 3NRE:A | 277 | 282 |
| 703 | 468 | ESVSE | 2OZJ:B | 48 | 52 |
| 704 | 469 | ETFTE | 2Z72:A | 263 | 267 |
| 705 | 470 | ETHTE | 3CZQ:A | 243 | 247 |
| 706 | 471 | ETITE | 1WMA:A | 160 | 164 |
| 707 |  | ETITE | 2GKM:B | 110 | 114 |
| 708 | 472 | ETLTE | 2GMW:B | 89 | 93 |
| 709 | 473 | ETNTE | 1ZX3:A | 73 | 77 |
| 710 | 474 | EVDVE | 3GOD:D | 96 | 114 |
| 711 | 475 | EVEEVE | 3S8S:A | 119 | 124 |
| 712 | 476 | EVEVE | 5CSM:A | 244 | 248 |
| 713 |  | EVEVE | 1WUI:L | 40 | 44 |
| 714 |  | EVEVE | 3D7A:B | 7 | 11 |
| 715 | 477 | EVGKGVE | 1H72:C | 292 | 298 |
| 716 | 478 | EVGVE | 2QJV:B | 124 | 128 |
| 717 | 479 | EVIVE | 1TH8:A | 119 | 123 |
| 718 | 480 | EVKVE | 3QOR:B | 225 | 229 |
| 719 | 481 | EVLVE | 3OD3:A | 282 | 286 |
| 720 |  | EVLVE | 1OA8:A | 644 | 648 |
| 721 |  | EVLVE | 2X4J:A | 112 | 116 |
| 722 | 482 | EVNVE | 3ELN:A | 27 | 31 |
| 723 |  | EVNVE | 1QGK:B | 33 | 37 |
| 724 | 483 | EVQVE | 3TDQ:B | 32 | 36 |
| 725 | 484 | EVSVE | 2I5V:O | 196 | 200 |
| 726 | 485 | EVVVE | 1CNU:A | 35 | 39 |
| 727 |  | EVVVE | 3H09:B | 735 | 739 |
| 728 |  | EVVVE | 2GKM:B | 26 | 30 |
| 729 |  | EVVVE | 3BF4:A | 100 | 104 |
| 730 | 486 | EVWVE | 3AKH:A | 385 | 389 |
| 731 |  | EVWVE | 3ALJ:A | 243 | 247 |
| 732 | 487 | EYEYE | 2PIE:A | 119 | 123 |
| 733 | 488 | FAAAF | 3ERM:E | 66 | 70 |
| 734 | 489 | FAEAF | 1EYQ:B | 135 | 139 |
| 735 | 490 | FAFAF | 2J8C:L | 119 | 123 |
| 736 | 491 | FAGAF | 3CX5:I | 22 | 26 |
| 737 | 492 | FAIAF | 1KLL:A | 73 | 77 |
| 738 | 493 | FAKAF | 3QC2:B | 188 | 192 |
| 739 |  | FAKAF | 3RF7:A | 211 | 215 |
| 740 | 494 | FDEDF | 3K67:B | 68 | 72 |
| 741 | 495 | FDLDF | 3PIK:A | 115 | 119 |
| 742 | 496 | FDPDF | 3RMI:A | 71 | 75 |
| 743 | 497 | FDSDF | 2EA7:A | 174 | 178 |
| 744 |  | FDSDF | 3T5X:A | 222 | 226 |
| 745 | 498 | FDTDF | 3SIB:A | 96 | 100 |
| 746 | 499 | FDYDF | 3ONH:A | 487 | 491 |
| 747 | 500 | FEAEF | 3GRD:B | 100 | 104 |
| 748 | 501 | FEQEF | 2Q8V:A | 14 | 18 |
| 749 | 502 | FESEF | 2PTV:A | 54 | 58 |
| 750 | 503 | FEYEF | 3BS4:A | 238 | 242 |
| 751 | 504 | FFAFF | 3SQN:B | 258 | 262 |
| 752 | 505 | FFGQGFF | 2WM9:A | 160 | 166 |
| 753 | 506 | FFSFF | 1FVK:A | 25 | 29 |
| 754 |  | FFSFF | 3OMD:A | 49 | 53 |
| 755 | 507 | FFYFF | 3N3M:A | 79 | 83 |
| 756 | 508 | FGAIAGF | 3KU3:B | 3 | 9 |
| 757 | 509 | FGGGF | 2J5I:A | 118 | 122 |
| 758 |  | FGGGF | 1HNJ:A | 304 | 308 |
| 759 | 510 | FGQGF | 2WM9:A | 161 | 165 |
| 760 | 511 | FGSGF | 3AG3:L | 29 | 33 |
| 761 |  | FGSGF | 2Q52:A | 49 | 53 |
| 762 | 512 | FGVGF | 3T0H:A | 134 | 138 |
| 763 | 513 | FIDIF | 3KKZ:B | 81 | 85 |
| 764 | 514 | FIVIF | 1V5I:B | 5 | 9 |
| 765 | 515 | FKDSDKF | 3BYJ:A | 143 | 149 |
| 766 | 516 | FLELF | 3V7P:A | 244 | 248 |
| 767 | 517 | FLGLF | 2BS2:F | 36 | 40 |
| 768 | 518 | FLILF | 1YMT:A | 374 | 378 |
| 769 | 519 | FLKLF | 1VJN:A | 177 | 181 |
| 770 |  | FLKLF | 3UJC:A | 228 | 232 |
| 771 | 520 | FLPLF | 3CZ6:A | 703 | 707 |
| 772 | 521 | FLQLF | 3M73:A | 235 | 239 |
| 773 |  | FLQLF | 1HQZ:4 | 55 | 59 |
| 774 | 522 | FLTLF | 1YIS:A | 204 | 208 |
| 775 | 523 | FLVLF | 3LYI:A | 987 | 991 |
| 776 | 524 | FLWLF | 2UVP:D | 134 | 138 |
| 777 | 525 | FNENF | 2DVT:C | 253 | 257 |
| 778 | 526 | FNPHPNF | 3QRA:A | 149 | 155 |
| 779 | 527 | FNQNF | 1JKG:A | 109 | 113 |
| 780 | 528 | FPDQDPF | 3I7M:A | 47 | 53 |
| 781 | 529 | FPVPF | 3U7Q:D | 179 | 183 |
| 782 | 530 | FPYPF | 1BWN:B | 98 | 102 |
| 783 | 531 | FQAQF | 1QOP:B | 41 | 45 |
| 784 | 532 | FQKQF | 3P8A:B | 185 | 189 |
| 785 | 533 | FRDRF | 3LW6:A | 83 | 87 |
| 786 | 534 | FRGRF | 2ZXE:B | 293 | 297 |
| 787 | 535 | FRKRF | 3NQA:B | 59 | 63 |
| 788 | 536 | FRNNRF | 3GVO:A | 714 | 719 |
| 789 | 537 | FRPRF | 2YVQ:A | 1370 | 1374 |
| 790 | 538 | FRRRF | 2I7D:A | 26 | 30 |
| 791 | 539 | FSASF | 1USC:B | 70 | 74 |
| 792 | 540 | FSCSF | 3CJJ:A | 206 | 210 |
| 793 | 541 | FSGSF | 3OLJ:A | 236 | 240 |
| 794 | 542 | FSHSF | 3EOJ:A | 108 | 112 |
| 795 | 543 | FSLSF | 2ABS:A | 354 | 358 |
| 796 | 544 | FSPSF | 3ALN:C | 336 | 340 |
| 797 | 545 | FSTSF | 2WAO:A | 226 | 230 |
| 798 |  | FSTSF | 2VU9:A | 941 | 945 |
| 799 |  | FSTSF | 3EPZ:B | 479 | 483 |
| 800 | 546 | FSVEVSF | 3AMN:B | 95 | 101 |
| 801 | 547 | FTATF | 2OFC:A | 63 | 67 |
| 802 | 548 | FTFTF | 2EGJ:B | 85 | 89 |
| 803 | 549 | FTPTF | 3DML:A | 59 | 63 |
| 804 | 550 | FTRTF | 2G6Y:B | 154 | 158 |
| 805 |  | FTRTF | 3NV0:A | 359 | 363 |
| 806 | 551 | FTYTF | 2AH5:A | 26 | 30 |
| 807 | 552 | FVVVF | 3GA3:A | 987 | 991 |
| 808 | 553 | FYDYF | 1XWT:A | 208 | 212 |
| 809 |  | FYDYF | 2X61:A | 95 | 99 |
| 810 | 554 | FYGYF | 1Y42:X | 345 | 349 |
| 811 | 555 | FYNYF | 2IU5:A | 43 | 47 |
| 812 | 556 | FYRYF | 2WTG:A | 34 | 38 |
| 813 | 557 | GAAAG | 1HX0:A | 106 | 110 |
| 814 |  | GAAAG | 3ZQU:A | 70 | 74 |
| 815 | 558 | GACAG | 2FDN:A | 41 | 45 |
| 816 | 559 | GADAG | 1HBN:F | 111 | 115 |
| 817 |  | GADAG | 1R7A:B | 48 | 52 |
| 818 | 560 | GAFAG | 1M4I:A | 30 | 34 |
| 819 | 561 | GAGAG | 2JJU:A | 41 | 45 |
| 820 | 562 | GAHAG | 1PZX:A | 268 | 272 |
| 821 |  | GAHAG | 2W72:A | 18 | 22 |
| 822 | 563 | GAIAG | 3D9S:D | 97 | 101 |
| 823 |  | GAIAG | 1U7G:A | 264 | 268 |
| 824 |  | GAIAG | 3KU3:B | 4 | 8 |
| 825 | 564 | GAKAG | 3PBT:A | 243 | 247 |
| 826 |  | GAKAG | 1HBN:F | 75 | 79 |
| 827 |  | GAKAG | 1XG0:B | 28 | 32 |
| 828 | 565 | GALAG | 2VFR:A | 395 | 399 |
| 829 |  | GALAG | 1R7A:B | 381 | 385 |
| 830 |  | GALAG | 2OH1:A | 74 | 78 |
| 831 |  | GALAG | 2FVY:A | 248 | 252 |
| 832 | 566 | GALLLAG | 3Q7H:N | 101 | 107 |
| 833 | 567 | GAMAG | 1W66:A | 0 | 0 |
| 834 | 568 | GANAG | 1ZEE:A | 233 | 237 |
| 835 | 569 | GAPIPAG | 2X9Z:A | 356 | 362 |
| 836 | 570 | GAPSPAG | 3RPW:A | 338 | 344 |
| 837 | 571 | GAQAG | 2VDJ:A | 144 | 148 |
| 838 | 572 | GASAG | 1RTT:A | 115 | 119 |
| 839 |  | GASAG | 2Q4H:B | 175 | 179 |
| 840 | 573 | GATAG | 3ZX3:A | 129 | 133 |
| 841 |  | GATAG | 3HIM:A | 147 | 151 |
| 842 |  | GATAG | 1YU0:A | 39 | 43 |
| 843 | 574 | GAVAG | 1DYO:A | 153 | 157 |
| 844 |  | GAVAG | 2E3H:A | 255 | 259 |
| 845 |  | GAVAG | 3H87:C | 57 | 61 |
| 846 | 575 | GAYAG | 3ARC:J | 31 | 35 |
| 847 | 576 | GCNCG | 1MC2:A | 1026 | 1030 |
| 848 | 577 | GCYCG | 3JQL:A | 26 | 30 |
| 849 | 578 | GDCDG | 2P26:A | 376 | 380 |
| 850 | 579 | GDEDG | 3OJN:D | 345 | 349 |
| 851 | 580 | GDGDG | 3G7R:B | 149 | 153 |
| 852 | 581 | GDKDG | 2PVB:A | 89 | 93 |
| 853 | 582 | GDNPRPNDG | 3B7E:A | 323 | 331 |
| 854 | 583 | GDSDG | 3EAT:X | 132 | 136 |
| 855 | 584 | GDTDG | 2Y8D:A | 2452 | 2456 |
| 856 | 585 | GDVDG | 1HW7:A | 161 | 165 |
| 857 |  | GDVDG | 3BB7:A | 326 | 330 |
| 858 |  | GDVDG | 2ZUX:B | 157 | 161 |
| 859 | 586 | GEAEG | 1IS1:A | 119 | 123 |
| 860 | 587 | GEEEG | 3H09:B | 663 | 667 |
| 861 | 588 | GEGEG | 2WUR:A | 31 | 35 |
| 862 |  | GEGEG | 2OS0:A | 109 | 113 |
| 863 |  | GEGEG | 3HPC:X | 110 | 114 |
| 864 |  | GEGEG | 3NED:A | 31 | 35 |
| 865 | 589 | GEIEG | 3QN1:B | 299 | 303 |
| 866 |  | GEIEG | 2AZW:A | 48 | 52 |
| 867 | 590 | GEKEG | 2GHC:X | 197 | 201 |
| 868 | 591 | GENEG | 2SN3:A | 20 | 24 |
| 869 | 592 | GEQEG | 2BHU:A | 340 | 344 |
| 870 |  | GEQEG | 3BAL:D | 82 | 86 |
| 871 | 593 | GEREG | 1UFO:D | 63 | 67 |
| 872 | 594 | GERTREG | 1F61:A | 251 | 257 |
| 873 | 595 | GEVEG | 2QHL:A | 94 | 98 |
| 874 | 596 | GEVVEG | 2CW9:A | 406 | 411 |
| 875 | 597 | GFDFG | 2WAO:A | 106 | 110 |
| 876 | 598 | GFEFG | 3PPQ:A | 188 | 192 |
| 877 | 599 | GFGFG | 1N7O:A | 561 | 565 |
| 878 |  | GFGFG | 1JFB:A | 344 | 348 |
| 879 | 600 | GFLFG | 2DY0:B | 67 | 71 |
| 880 | 601 | GFRFG | 3ERB:A | 144 | 148 |
| 881 | 602 | GFTFG | 1IXL:A | 49 | 53 |
| 882 | 603 | GFVFG | 1UX6:A | 1006 | 1010 |
| 883 | 604 | GGCGG | 1KTH:A | 36 | 40 |
| 884 | 605 | GGDGG | 3C6K:C | 201 | 205 |
| 885 |  | GGDGG | 1INL:A | 99 | 103 |
| 886 | 606 | GGEQEGG | 3BAL:D | 81 | 87 |
| 887 | 607 | GGFGG | 2X5X:A | 29 | 33 |
| 888 | 608 | GGHGG | 2PTH:A | 108 | 112 |
| 889 |  | GGHGG | 3PI6:A | 267 | 271 |
| 890 |  | GGHGG | 1T0I:B | 125 | 129 |
| 891 | 609 | GGIGG | 3A2O:B | 148 | 152 |
| 892 | 610 | GGKGG | 2WOJ:C | 24 | 28 |
| 893 |  | GGKGG | 3D02:A | 149 | 153 |
| 894 | 611 | GGLGG | 2E6F:A | 218 | 222 |
| 895 | 612 | GGPGG | 1U4G:A | 4 | 8 |
| 896 |  | GGPGG | 1SBY:B | 127 | 131 |
| 897 | 613 | GGQGG | 1N2F:A | 40 | 44 |
| 898 | 614 | GGRGG | 1ZVA:A | 39 | 43 |
| 899 | 615 | GGSGG | 2CBP:A | 6 | 10 |
| 900 |  | GGSGG | 3DK9:A | 28 | 32 |
| 901 |  | GGSGG | 3O3X:A | 38 | 42 |
| 902 |  | GGSGG | 3O3X:A | 77 | 81 |
| 903 |  | GGSGG | 3O3X:A | 119 | 123 |
| 904 |  | GGSGG | 3O3X:A | 158 | 162 |
| 905 | 616 | GGSSGG | 3CP7:A | 166 | 171 |
| 906 | 617 | GGSWSGG | 1K3I:A | 287 | 293 |
| 907 | 618 | GGTGG | 2FKK:A | 517 | 521 |
| 908 | 619 | GGVGG | 2H3L:B | 1338 | 1342 |
| 909 | 620 | GGWGG | 2YHG:A | 761 | 765 |
| 910 | 621 | GGYGG | 1URS:A | 186 | 190 |
| 911 | 622 | GHGHG | 2OX8:A | 699 | 703 |
| 912 | 623 | GHHHG | 3IB7:A | 138 | 142 |
| 913 | 624 | GHKHG | 1EUW:A | 75 | 79 |
| 914 | 625 | GIDIG | 2H00:C | 69 | 73 |
| 915 | 626 | GIFIG | 2ZXE:B | 49 | 53 |
| 916 | 627 | GIHIG | 3QZR:A | 159 | 163 |
| 917 |  | GIHIG | 2TPS:A | 105 | 109 |
| 918 | 628 | GIIIG | 2QF4:B | 191 | 195 |
| 919 | 629 | GILIG | 2YVQ:A | 1361 | 1365 |
| 920 | 630 | GIMIG | 1M0K:A | 116 | 120 |
| 921 | 631 | GIPIG | 3IJD:B | 272 | 276 |
| 922 | 632 | GIRIG | 3B7E:A | 105 | 109 |
| 923 | 633 | GISIG | 1K5C:A | 196 | 200 |
| 924 |  | GISIG | 3V5U:A | 68 | 72 |
| 925 |  | GISIG | 2GZV:A | 34 | 38 |
| 926 | 634 | GITIG | 3DNS:B | 26 | 30 |
| 927 | 635 | GIYIG | 3FY5:B | 280 | 284 |
| 928 | 636 | GKAAKG | 3QT9:A | 340 | 345 |
| 929 | 637 | GKDKG | 3MW4:A | 215 | 219 |
| 930 |  | GKDKG | 2VYO:A | 202 | 206 |
| 931 | 638 | GKEKG | 2HEU:A | 280 | 284 |
| 932 | 639 | GKGKG | 3H8T:A | 90 | 94 |
| 933 |  | GKGKG | 1FSG:A | 10 | 14 |
| 934 | 640 | GKLKG | 3E8T:A | 61 | 65 |
| 935 | 641 | GKLLKG | 1RZ4:A | 12 | 17 |
| 936 | 642 | GKLWLKG | 1ZPS:B | 64 | 70 |
| 937 | 643 | GKMKG | 1QIP:D | 155 | 159 |
| 938 | 644 | GKPKG | 3VMK:B | 145 | 149 |
| 939 | 645 | GKQKG | 4E0Q:B | 87 | 91 |
| 940 | 646 | GKTKG | 3NS6:A | 121 | 125 |
| 941 | 647 | GKVKG | 1GL4:A | 406 | 410 |
| 942 |  | GKVKG | 2JAE:A | 7 | 11 |
| 943 | 648 | GLALG | 3FVV:A | 172 | 176 |
| 944 | 649 | GLCLG | 1F5V:B | 154 | 158 |
| 945 | 650 | GLELG | 2HCR:B | 24 | 28 |
| 946 | 651 | GLFLG | 3NUL:A | 64 | 68 |
| 947 |  | GLFLG | 2BS2:F | 34 | 38 |
| 948 | 652 | GLGGLG | 1JW9:B | 38 | 43 |
| 949 | 653 | GLGLG | 1GVE:B | 58 | 62 |
| 950 | 654 | GLILG | 1J3A:A | 8 | 12 |
| 951 | 655 | GLKLG | 1UAS:A | 87 | 91 |
| 952 |  | GLKLG | 3C70:A | 145 | 149 |
| 953 |  | GLKLG | 1PK3:C | 63 | 67 |
| 954 |  | GLKLG | 3NPK:A | 202 | 206 |
| 955 | 656 | GLNKNLG | 2OFW:C | 94 | 100 |
| 956 | 657 | GLNLG | 3VNY:A | 138 | 142 |
| 957 | 658 | GLRLG | 1LC5:A | 222 | 226 |
| 958 |  | GLRLG | 3OA2:D | 91 | 95 |
| 959 |  | GLRLG | 3RHE:A | 45 | 49 |
| 960 | 659 | GLSLG | 1TQH:A | 92 | 96 |
| 961 | 660 | GLTLG | 2DFB:A | 163 | 167 |
| 962 |  | GLTLG | 3BL4:A | 12 | 16 |
| 963 | 661 | GLVLG | 1H4X:B | 61 | 65 |
| 964 |  | GLVLG | 3V5U:A | 83 | 87 |
| 965 | 662 | GNAANG | 1N62:F | 114 | 119 |
| 966 | 663 | GNENG | 1R45:D | 95 | 99 |
| 967 | 664 | GNLNG | 3H09:B | 111 | 115 |
| 968 | 665 | GNNNG | 3NO8:B | 506 | 510 |
| 969 | 666 | GNQNG | 1K3I:A | 76 | 80 |
| 970 | 667 | GNSNG | 3M7P:A | 353 | 357 |
| 971 |  | GNSNG | 3M7P:A | 414 | 417 |
| 972 | 668 | GNVEVNG | 2F0C:A | 52 | 58 |
| 973 | 669 | GNVNG | 3FYF:A | 122 | 126 |
| 974 | 670 | GPEPG | 2R01:A | 86 | 90 |
| 975 | 671 | GPKPG | 2JC9:A | 269 | 273 |
| 976 | 672 | GPLPG | 2HC1:A | 1777 | 1781 |
| 977 |  | GPLPG | 1USC:B | 8 | 12 |
| 978 | 673 | GPQPG | 3R8J:A | 32 | 36 |
| 979 | 674 | GQAQG | 2H5C:A | 207 | 211 |
| 980 | 675 | GQPGPQG | 1H41:B | 293 | 299 |
| 981 | 676 | GQRQG | 3LQB:A | 180 | 184 |
| 982 | 677 | GQTITQG | 3LGI:C | 165 | 171 |
| 983 | 678 | GQTQG | 1CV8:A | 74 | 78 |
| 984 | 679 | GQVKVQG | 1KJQ:B | 386 | 392 |
| 985 | 680 | GQVQG | 3HTY:P | 38 | 42 |
| 986 | 681 | GRARG | 3OQI:A | 157 | 161 |
| 987 |  | GRARG | 3OK8:A | 203 | 207 |
| 988 | 682 | GRLLRG | 1K5N:A | 107 | 112 |
| 989 | 683 | GRLRG | 2J45:B | 14 | 18 |
| 990 |  | GRLRG | 3H3H:A | 52 | 56 |
| 991 | 684 | GRRRG | 3IAR:A | 31 | 35 |
| 992 | 685 | GRTRG | 1R8N:A | 37 | 41 |
| 993 | 686 | GRVRG | 1VH5:A | 96 | 100 |
| 994 | 687 | GRWRG | 3LJK:A | 127 | 131 |
| 995 | 688 | GSASG | 1JB0:E | 50 | 54 |
| 996 |  | GSASG | 3BF4:A | 48 | 52 |
| 997 | 689 | GSCSG | 2E8E:A | 43 | 47 |
| 998 | 690 | GSDSG | 2MCM:A | 98 | 102 |
| 999 |  | GSDSG | 2O6P:B | 28 | 32 |
| 1000 | 691 | GSGGSG | 3O3X:A | 78 | 83 |
| 1001 | 692 | GSGSG | 1N67:A | 532 | 536 |
| 1002 |  | GSGSG | 1M3S:A | 85 | 89 |
| 1003 |  | GSGSG | 1MJU:L | 64 | 68 |
| 1004 |  | GSGSG | 3MDY:A | 187 | 191 |
| 1005 | 693 | GSHSG | 1USC:B | 87 | 91 |
| 1006 | 694 | GSISG | 2AEB:B | 106 | 110 |
| 1007 |  | GSISG | 3OGH:B | 96 | 100 |
| 1008 | 695 | GSLSG | 1DEU:A | 133 | 137 |
| 1009 | 696 | GSMSG | 1QCX:A | 320 | 324 |
| 1010 |  | GSMSG | 2X3H:C | 393 | 397 |
| 1011 | 697 | GSNSG | 3Q7Z:A | 357 | 361 |
| 1012 |  | GSNSG | 2Y3C:A | 233 | 237 |
| 1013 | 698 | GSPSG | 1OD3:A | 94 | 98 |
| 1014 |  | GSPSG | 3F9F:B | 120 | 124 |
| 1015 |  | GSPSG | 3OZY:A | 50 | 54 |
| 1016 | 699 | GSQSG | 1BXO:A | 201 | 205 |
| 1017 | 700 | GSTSG | 3SOJ:B | 72 | 76 |
| 1018 |  | GSTSG | 1EB6:A | 67 | 71 |
| 1019 |  | GSTSG | 1XG5:C | 109 | 113 |
| 1020 |  | GSTSG | 3BY9:A | 148 | 152 |
| 1021 | 701 | GSVSG | 3IIS:M | 71 | 75 |
| 1022 |  | GSVSG | 1SR4:C | 151 | 155 |
| 1023 | 702 | GSWSG | 1K3I:A | 288 | 292 |
| 1024 |  | GSWSG | 3PIK:A | 91 | 95 |
| 1025 | 703 | GSYSG | 2V3I:A | 319 | 323 |
| 1026 |  | GSYSG | 2X3H:C | 167 | 171 |
| 1027 | 704 | GTCTG | 2Y8N:B | 60 | 64 |
| 1028 | 705 | GTGTG | 1EK6:A | 270 | 274 |
| 1029 |  | GTGTG | 3FMF:D | 8 | 12 |
| 1030 | 706 | GTITG | 1B34:A | 27 | 31 |
| 1031 |  | GTITG | 3U80:A | 125 | 129 |
| 1032 | 707 | GTKTG | 2GHC:X | 50 | 54 |
| 1033 | 708 | GTLLTG | 2Y4Z:A | 60 | 65 |
| 1034 | 709 | GTQTG | 1RCF:A | 9 | 13 |
| 1035 | 710 | GTYTG | 2JC9:A | 315 | 319 |
| 1036 |  | GTYTG | 3DDC:B | 229 | 233 |
| 1037 |  | GTYTG | 3Q0H:A | 116 | 120 |
| 1038 |  | GTYTG | 3GE2:A | 75 | 79 |
| 1039 | 711 | GVAVG | 2Q3G:A | 43 | 47 |
| 1040 |  | GVAVG | 3VBL:E | 141 | 145 |
| 1041 | 712 | GVCVG | 3IRS:A | 62 | 66 |
| 1042 | 713 | GVDVG | 2I15:A | 109 | 113 |
| 1043 | 714 | GVFVG | 3MOE:A | 453 | 457 |
| 1044 |  | GVFVG | 4ADU:B | 234 | 238 |
| 1045 | 715 | GVGVG | 3JU4:A | 701 | 705 |
| 1046 |  | GVGVG | 2QMQ:A | 142 | 146 |
| 1047 | 716 | GVIVG | 3FLP:E | 206 | 210 |
| 1048 | 717 | GVLLVG | 1ME4:A | 160 | 165 |
| 1049 | 718 | GVLVG | 3GOD:D | 78 | 82 |
| 1050 |  | GVLVG | 3V5U:A | 174 | 178 |
| 1051 | 719 | GVNVG | 1AOH:A | 143 | 147 |
| 1052 | 720 | GVPVG | 4DZI:C | 223 | 227 |
| 1053 | 721 | GVQVG | 4DZI:C | 393 | 397 |
| 1054 | 722 | GVRVG | 1PBJ:A | 41 | 45 |
| 1055 |  | GVRVG | 1R4P:A | 217 | 221 |
| 1056 | 723 | GVSVG | 3K1W:B | 207 | 211 |
| 1057 |  | GVSVG | 2QV3:A | 523 | 527 |
| 1058 | 724 | GVTVG | 1W0N:A | 79 | 83 |
| 1059 | 725 | GWGWG | 4DNU:A | 195 | 199 |
| 1060 | 726 | GWYWG | 2VIF:A | 383 | 387 |
| 1061 | 727 | GYCYG | 3ACX:A | 128 | 132 |
| 1062 | 728 | GYDYG | 3CZ8:A | 257 | 261 |
| 1063 | 729 | GYGGYG | 1GA6:A | 335 | 340 |
| 1064 | 730 | GYIYG | 2QIK:A | 55 | 59 |
| 1065 | 731 | GYQYG | 2BI7:A | 241 | 245 |
| 1066 |  | GYQYG | 3QB8:B | 172 | 176 |
| 1067 | 732 | GYTYG | 2J8C:L | 161 | 165 |
| 1068 |  | GYTYG | 3QZX:A | 8 | 12 |
| 1069 | 733 | HAAAH | 3L2H:A | 100 | 104 |
| 1070 | 734 | HALAH | 1QOP:B | 353 | 357 |
| 1071 | 735 | HCPCH | 1JM1:A | 169 | 173 |
| 1072 | 736 | HDGGDH | 3NFW:A | 125 | 130 |
| 1073 | 737 | HDMDH | 4DGQ:C | 70 | 74 |
| 1074 | 738 | HDSSDH | 2BKR:A | 45 | 50 |
| 1075 | 739 | HDYDH | 1SO2:D | 821 | 825 |
| 1076 | 740 | HEFFEH | 3CZT:X | 85 | 90 |
| 1077 | 741 | HENEH | 2YVT:A | 68 | 72 |
| 1078 | 742 | HEWEH | 3HT1:A | 55 | 59 |
| 1079 | 743 | HGTGH | 3CTZ:A | 485 | 489 |
| 1080 | 744 | HHPHH | 2P2S:A | 207 | 211 |
| 1081 | 745 | HIDIH | 2VLQ:B | 127 | 131 |
| 1082 | 746 | HIRIH | 2VT3:A | 186 | 190 |
| 1083 | 747 | HKAKH | 1AYM:1 | 238 | 242 |
| 1084 | 748 | HLCLH | 2ZKM:X | 775 | 779 |
| 1085 | 749 | HLELH | 1FQJ:C | 75 | 79 |
| 1086 | 750 | HLGLH | 3D59:A | 395 | 399 |
| 1087 | 751 | HLILH | 2Z80:A | 202 | 206 |
| 1088 | 752 | HQGQH | 1MKK:A | 86 | 90 |
| 1089 | 753 | HSFSH | 2W3Z:A | 166 | 170 |
| 1090 | 754 | HSGSH | 1H4A:X | 83 | 88 |
| 1091 | 755 | HTETH | 2VGO:A | 294 | 298 |
| 1092 | 756 | HVEVH | 2WJ5:A | 35 | 39 |
| 1093 | 757 | HVPVH | 3BVU:A | 705 | 709 |
| 1094 | 758 | IAAAI | 1YD7:A | 69 | 73 |
| 1095 |  | IAAAI | 3S6E:A | 481 | 485 |
| 1096 | 759 | IADAI | 2QIF:B | 56 | 60 |
| 1097 |  | IADAI | 3GJU:A | 71 | 75 |
| 1098 | 760 | IAEAI | 3LA7:B | 179 | 183 |
| 1099 | 761 | IAFAI | 3LJK:A | 272 | 276 |
| 1100 | 762 | IAGAI | 3PJ0:D | 212 | 216 |
| 1101 |  | IAGAI | 3AAY:A | 39 | 43 |
| 1102 | 763 | IAGGAI | 2IMZ:B | 61 | 66 |
| 1103 | 764 | IAIAI | 1MG7:B | 327 | 331 |
| 1104 | 765 | IAMAI | 1N1J:B | 101 | 105 |
| 1105 | 766 | IANAI | 3LQB:A | 38 | 42 |
| 1106 |  | IANAI | 3G14:A | 59 | 63 |
| 1107 | 767 | IAPAI | 2D0B:A | 142 | 146 |
| 1108 | 768 | IAQAI | 3OKF:B | 356 | 360 |
| 1109 |  | IAQAI | 3VMK:B | 356 | 360 |
| 1110 |  | IAQAI | 2RCI:A | 54 | 58 |
| 1111 | 769 | IASAI | 3AJM:A | 134 | 138 |
| 1112 |  | IASAI | 2IBP:B | 322 | 326 |
| 1113 |  | IASAI | 1QSA:A | 187 | 191 |
| 1114 | 770 | IATAI | 3AMR:A | 123 | 127 |
| 1115 |  | IATAI | 2A25:A | 247 | 251 |
| 1116 | 771 | IDADI | 1VHT:C | 30 | 34 |
| 1117 | 772 | IDDDI | 4ADN:B | 60 | 64 |
| 1118 | 773 | IDIDI | 1CR5:A | 117 | 121 |
| 1119 | 774 | IDKDI | 2QZI:D | 73 | 77 |
| 1120 | 775 | IDLDI | 8A3H:A | 90 | 94 |
| 1121 |  | IDLDI | 2H9D:A | 20 | 24 |
| 1122 | 776 | IDNDI | 1JF8:A | 67 | 71 |
| 1123 | 777 | IDPDI | 1OU0:A | 25 | 29 |
| 1124 | 778 | IDVDI | 3F6C:B | 51 | 55 |
| 1125 |  | IDVDI | 3CXG:B | 76 | 80 |
| 1126 | 779 | IEDDEI | 3OD3:A | 162 | 167 |
| 1127 | 780 | IEDEI | 3V0S:A | 186 | 190 |
| 1128 | 781 | IEFEI | 3MNM:A | 483 | 487 |
| 1129 | 782 | IEKEI | 4A3P:A | 161 | 165 |
| 1130 |  | IEKEI | 3D36:C | 29 | 33 |
| 1131 | 783 | IELLEI | 3GGY:A | 67 | 72 |
| 1132 | 784 | IEPEI | 3VHV:A | 745 | 749 |
| 1133 | 785 | IETEI | 2A26:A | 36 | 40 |
| 1134 |  | IETEI | 3KT7:A | 74 | 78 |
| 1135 | 786 | IEVVEI | 2AUA:B | 186 | 191 |
| 1136 | 787 | IFKFI | 5CSM:A | 27 | 31 |
| 1137 |  | IFKFI | 4AJJ:A | 115 | 119 |
| 1138 | 788 | IGAGI | 3NYC:A | 1009 | 1013 |
| 1139 | 789 | IGFGI | 1F60:B | 1161 | 1165 |
| 1140 | 790 | IGGGI | 1EP3:B | 116 | 120 |
| 1141 | 791 | IGLGI | 2A5Z:C | 143 | 147 |
| 1142 | 792 | IGPGI | 1GXR:B | 467 | 471 |
| 1143 | 793 | IGRGI | 2OKV:D | 23 | 27 |
| 1144 | 794 | IGSGI | 3U7Q:A | 421 | 425 |
| 1145 | 795 | IGTGI | 3V2U:D | 324 | 328 |
| 1146 | 796 | IIAII | 1D8D:A | 155 | 159 |
| 1147 | 797 | IIGII | 1QZM:A | 528 | 532 |
| 1148 |  | IIGII | 3EN0:C | 38 | 42 |
| 1149 |  | IIGII | 3IGS:B | 69 | 73 |
| 1150 | 798 | IIRII | 2R4I:B | 69 | 73 |
| 1151 | 799 | IKAKI | 1O4Y:A | 124 | 128 |
| 1152 | 800 | IKEKI | 1XMK:A | 299 | 303 |
| 1153 |  | IKEKI | 2NML:A | 87 | 91 |
| 1154 | 801 | IKLKI | 1ZHV:A | 5 | 9 |
| 1155 | 802 | IKNKI | 3BBD:A | 19 | 23 |
| 1156 | 803 | IKQKI | 3DDC:B | 206 | 210 |
| 1157 |  | IKQKI | 2WCR:A | 101 | 105 |
| 1158 | 804 | IKRKI | 2VUW:A | 770 | 774 |
| 1159 | 805 | IKVKI | 2GDQ:B | 158 | 162 |
| 1160 | 806 | ILALI | 3RQA:D | 81 | 85 |
| 1161 |  | ILALI | 1ZWY:A | 168 | 172 |
| 1162 | 807 | ILDLI | 2ZPT:X | 57 | 61 |
| 1163 | 808 | ILELI | 2YVE:B | 169 | 173 |
| 1164 | 809 | ILHLI | 2ODM:A | 22 | 26 |
| 1165 | 810 | ILILI | 3AG3:B | 72 | 76 |
| 1166 | 811 | ILKPKLI | 2VSM:A | 197 | 203 |
| 1167 | 812 | ILRLI | 3K0X:A | 34 | 38 |
| 1168 |  | ILRLI | 2B5G:A | 17 | 21 |
| 1169 | 813 | ILSLI | 2OXL:A | 54 | 58 |
| 1170 | 814 | ILTLI | 2RBG:A | 6 | 10 |
| 1171 |  | ILTLI | 3K06:B | 32 | 36 |
| 1172 | 815 | IMSMI | 1EYH:A | 56 | 60 |
| 1173 | 816 | INKNI | 3MYU:B | 259 | 263 |
| 1174 | 817 | INNNNI | 3LLT:A | 608 | 613 |
| 1175 | 818 | IPAPI | 2OX0:B | 67 | 71 |
| 1176 | 819 | IPDPI | 2D42:B | 45 | 49 |
| 1177 | 820 | IPFPI | 3A2V:J | 97 | 101 |
| 1178 | 821 | IPNPI | 3EOJ:A | 133 | 137 |
| 1179 | 822 | IPSPI | 1PA2:A | 138 | 142 |
| 1180 | 823 | IPTPI | 3P1V:B | 366 | 370 |
| 1181 | 824 | IPYYPI | 2BI7:A | 311 | 316 |
| 1182 | 825 | IQEQI | 1DQP:B | 137 | 141 |
| 1183 | 826 | IQNENQI | 3F14:A | 66 | 72 |
| 1184 | 827 | IQRQI | 4E0Q:B | 151 | 155 |
| 1185 | 828 | IQSQI | 3LUM:D | 87 | 91 |
| 1186 |  | IQSQI | 1D9C:A | 45 | 49 |
| 1187 | 829 | IRARI | 2CMP:A | 51 | 55 |
| 1188 | 830 | IRGRI | 2R31:A | 186 | 190 |
| 1189 | 831 | IRKRI | 3D32:B | 66 | 70 |
| 1190 | 832 | IRRCRRI | 2POC:D | 392 | 398 |
| 1191 | 833 | IRTRI | 1TC1:B | 18 | 22 |
| 1192 | 834 | ISESI | 1S0P:A | 142 | 146 |
| 1193 |  | ISESI | 3BVF:F | 97 | 101 |
| 1194 | 835 | ISFSI | 2ZXE:B | 89 | 93 |
| 1195 | 836 | ISGSI | 1BTE:A | 48 | 52 |
| 1196 | 837 | ISNVNSI | 3C8G:D | 116 | 122 |
| 1197 | 838 | ISPPSI | 1M93:A | 18 | 23 |
| 1198 | 839 | ISSSI | 2F0C:A | 94 | 98 |
| 1199 | 840 | ISTSI | 3BB9:F | 105 | 109 |
| 1200 | 841 | ISVSI | 2XZ4:A | 151 | 155 |
| 1201 | 842 | ITALATI | 2O7T:A | 161 | 167 |
| 1202 | 843 | ITDTI | 3B7E:A | 211 | 215 |
| 1203 | 844 | ITETI | 1EJD:A | 323 | 327 |
| 1204 | 845 | ITGTI | 2J8C:L | 250 | 254 |
| 1205 |  | ITGTI | 1RTQ:A | 22 | 26 |
| 1206 | 846 | ITITI | 3D7A:B | 111 | 115 |
| 1207 | 847 | ITTTI | 1KQ3:A | 2 | 6 |
| 1208 | 848 | IVDDVI | 3CNE:D | 52 | 57 |
| 1209 | 849 | IVGVI | 3QY9:D | 26 | 30 |
| 1210 | 850 | IVKVI | 2IXD:B | 89 | 93 |
| 1211 | 851 | IVPVI | 3QHP:B | 299 | 303 |
| 1212 | 852 | IVSISVI | 1TH8:A | 63 | 69 |
| 1213 | 853 | IVSVI | 1X46:A | 68 | 72 |
| 1214 | 854 | IVTVI | 3MVS:A | 163 | 167 |
| 1215 | 855 | IYPYI | 3EGW:C | 11 | 15 |
| 1216 | 856 | IYQYI | 1U7G:A | 95 | 99 |
| 1217 | 857 | IYSYI | 1H41:B | 275 | 279 |
| 1218 |  | IYSYI | 2VDU:D | 101 | 105 |
| 1219 | 858 | KAAAAAK | 2PTZ:A | 118 | 124 |
| 1220 |  | KAAAAAK | 3ELF:A | 149 | 155 |
| 1221 | 859 | KAAAK | 2VYN:D | 260 | 264 |
| 1222 |  | KAAAK | 3EGN:A | 477 | 481 |
| 1223 |  | KAAAK | 3MQ2:A | 67 | 71 |
| 1224 | 860 | KAAEQEAAK | 2D0B:A | 56 | 64 |
| 1225 | 861 | KAGAK | 1RWJ:A | 43 | 47 |
| 1226 |  | KAGAK | 3GKM:A | 64 | 68 |
| 1227 |  | KAGAK | 1YKW:A | 393 | 397 |
| 1228 | 862 | KAGGAK | 1V8D:C | 141 | 146 |
| 1229 | 863 | KAHAK | 3GKM:A | 155 | 159 |
| 1230 | 864 | KAIAK | 3R46:F | 11 | 15 |
| 1231 |  | KAIAK | 3ETI:A | 143 | 147 |
| 1232 |  | KAIAK | 3EVZ:A | 45 | 49 |
| 1233 | 865 | KAKAK | 3KB2:B | 93 | 97 |
| 1234 | 866 | KALAK | 3ORK:A | 253 | 257 |
| 1235 | 867 | KARAK | 3GM5:A | 58 | 62 |
| 1236 | 868 | KATAK | 2ABK:A | 49 | 53 |
| 1237 | 869 | KAVAK | 3A9S:C | 40 | 44 |
| 1238 |  | KAVAK | 1EP3:B | 204 | 208 |
| 1239 | 870 | KAVEVAK | 3G3L:A | 155 | 161 |
| 1240 | 871 | KCICK | 1L5P:C | 46 | 50 |
| 1241 | 872 | KDADK | 3SEE:A | 165 | 169 |
| 1242 | 873 | KDGDK | 1XM8:B | 92 | 96 |
| 1243 |  | KDGDK | 2XE5:A | 29 | 33 |
| 1244 | 874 | KDKDK | 2QHL:A | 27 | 31 |
| 1245 |  | KDKDK | 2RDG:A | 52 | 56 |
| 1246 |  | KDKDK | 3QWW:A | 270 | 274 |
| 1247 | 875 | KDLDK | 3SGG:A | 256 | 260 |
| 1248 |  | KDLDK | 1O04:G | 434 | 438 |
| 1249 | 876 | KDLLDK | 2E6X:A | 3 | 8 |
| 1250 | 877 | KDMDK | 1Z7C:A | 168 | 172 |
| 1251 | 878 | KDSDK | 3BYJ:A | 144 | 148 |
| 1252 | 879 | KDVDK | 3QAO:A | 42 | 46 |
| 1253 | 880 | KDYDK | 2QEB:A | 136 | 140 |
| 1254 | 881 | KEAAEAAEK | 3A9S:C | 71 | 79 |
| 1255 | 882 | KEAAEK | 1PBJ:A | 85 | 90 |
| 1256 | 883 | KEAEK | 3DQG:A | 542 | 546 |
| 1257 |  | KEAEK | 1QYS:A | 58 | 62 |
| 1258 |  | KEAEK | 3I10:A | 201 | 205 |
| 1259 |  | KEAEK | 4DEM:F | 307 | 311 |
| 1260 |  | KEAEK | 3PPQ:A | 102 | 106 |
| 1261 |  | KEAEK | 1URQ:C | 72 | 76 |
| 1262 | 884 | KECEK | 1KTH:A | 49 | 53 |
| 1263 | 885 | KEDEK | 3NE8:A | 372 | 376 |
| 1264 | 886 | KEGEK | 3NGW:A | 106 | 110 |
| 1265 |  | KEGEK | 3EIX:A | 168 | 172 |
| 1266 | 887 | KEIEK | 1C44:A | 14 | 18 |
| 1267 |  | KEIEK | 3KP7:B | 128 | 132 |
| 1268 | 888 | KEKEK | 3CTP:A | 102 | 106 |
| 1269 | 889 | KELEK | 3PWT:A | 484 | 488 |
| 1270 |  | KELEK | 1BB9:A | 76 | 80 |
| 1271 | 890 | KENEK | 1T6F:A | 7 | 11 |
| 1272 | 891 | KEPEK | 2FHP:A | 88 | 92 |
| 1273 |  | KEPEK | 3UFE:B | 59 | 63 |
| 1274 | 892 | KEREK | 2O1M:B | 88 | 92 |
| 1275 | 893 | KEVEK | 2VOG:A | 46 | 50 |
| 1276 | 894 | KEYEK | 2PR5:B | 130 | 134 |
| 1277 |  | KEYEK | 1SR4:C | 74 | 78 |
| 1278 |  | KEYEK | 3BPQ:A | 15 | 19 |
| 1279 | 895 | KFAEAFK | 1EYQ:B | 134 | 140 |
| 1280 | 896 | KFGFK | 1WWZ:B | 138 | 142 |
| 1281 | 897 | KFIVIFK | 1V5I:B | 4 | 10 |
| 1282 | 898 | KFLFK | 2IP1:A | 200 | 204 |
| 1283 | 899 | KFTFK | 2O1M:B | 57 | 61 |
| 1284 | 900 | KGFGK | 1NRG:A | 100 | 104 |
| 1285 | 901 | KGIIGK | 1A92:D | 55 | 60 |
| 1286 | 902 | KGLGK | 3KX6:D | 64 | 68 |
| 1287 | 903 | KGNNGK | 1JLY:B | 168 | 173 |
| 1288 | 904 | KGQGK | 2W72:A | 56 | 60 |
| 1289 | 905 | KGTGK | 2WFI:A | 48 | 52 |
| 1290 | 906 | KGWGK | 1GKP:E | 446 | 450 |
| 1291 | 907 | KHLGLHK | 3D59:A | 394 | 400 |
| 1292 | 908 | KIAIK | 3JSY:A | 157 | 161 |
| 1293 | 909 | KIETEIK | 2A26:A | 35 | 41 |
| 1294 | 910 | KIHIK | 2ZX2:B | 19 | 23 |
| 1295 | 911 | KIIIIIK | 2I5V:O | 119 | 125 |
| 1296 | 912 | KIIIK | 1A78:B | 96 | 100 |
| 1297 | 913 | KILIK | 2ZKM:X | 457 | 461 |
| 1298 | 914 | KIPIK | 2WBM:A | 157 | 161 |
| 1299 | 915 | KITIK | 1H72:C | 82 | 86 |
| 1300 | 916 | KKGKK | 2YD6:A | 59 | 63 |
| 1301 |  | KKGKK | 3PCT:C | 53 | 57 |
| 1302 |  | KKGKK | 1Y42:X | 60 | 64 |
| 1303 |  | KKGKK | 2YWW:A | 51 | 55 |
| 1304 | 917 | KKIKK | 1A92:D | 39 | 43 |
| 1305 |  | KKIKK | 2Q7D:B | 17 | 21 |
| 1306 |  | KKIKK | 3SG8:A | 155 | 159 |
| 1307 | 918 | KKLKK | 1FIA:A | 90 | 94 |
| 1308 |  | KKLKK | 3O0P:A | 170 | 174 |
| 1309 | 919 | KKLLKK | 1SBY:B | 72 | 77 |
| 1310 | 920 | KKMKK | 2GRC:A | 1460 | 1464 |
| 1311 | 921 | KKPPKK | 3LKM:A | 680 | 685 |
| 1312 | 922 | KKTKK | 3NZN:B | 36 | 40 |
| 1313 |  | KKTKK | 3KEP:B | 558 | 562 |
| 1314 | 923 | KKVKK | 4ADN:B | 108 | 112 |
| 1315 | 924 | KLALK | 2CKW:A | 163 | 167 |
| 1316 |  | KLALK | 3BUX:B | 74 | 78 |
| 1317 |  | KLALK | 1BI5:A | 316 | 320 |
| 1318 | 925 | KLDLK | 1P1J:B | 57 | 61 |
| 1319 |  | KLDLK | 2P25:A | 44 | 48 |
| 1320 |  | KLDLK | 3EIK:A | 37 | 41 |
| 1321 | 926 | KLELK | 1MJU:L | 103 | 107 |
| 1322 | 927 | KLGLK | 3IJW:B | 23 | 27 |
| 1323 |  | KLGLK | 3NPK:A | 200 | 204 |
| 1324 | 928 | KLILK | 2VXT:I | 171 | 175 |
| 1325 | 929 | KLKLK | 3I10:A | 112 | 116 |
| 1326 |  | KLKLK | 3E8T:A | 115 | 119 |
| 1327 | 930 | KLLLK | 3PA8:B | 171 | 175 |
| 1328 | 931 | KLLNLLK | 1NZ0:A | 107 | 113 |
| 1329 | 932 | KLQLK | 2WM9:A | 334 | 338 |
| 1330 | 933 | KLSLK | 3S6N:G | 16 | 20 |
| 1331 | 934 | KLTLK | 2WUR:A | 41 | 45 |
| 1332 | 935 | KLVLK | 1GL4:A | 500 | 504 |
| 1333 | 936 | KLWLK | 1ZPS:B | 65 | 69 |
| 1334 | 937 | KNGGNK | 3OJ0:A | 15 | 20 |
| 1335 | 938 | KNKNK | 2P6W:A | 86 | 90 |
| 1336 | 939 | KNTIITNK | 2AS9:A | 32 | 39 |
| 1337 | 940 | KPAPK | 1LY2:A | 57 | 61 |
| 1338 | 941 | KPGGPK | 2IBP:B | 278 | 283 |
| 1339 | 942 | KPNPK | 1OFW:B | 22 | 26 |
| 1340 | 943 | KQAAQK | 1B8O:A | 265 | 270 |
| 1341 | 944 | KQAQK | 1YBX:B | 18 | 22 |
| 1342 | 945 | KQIQK | 2IP1:A | 365 | 369 |
| 1343 | 946 | KRGRK | 1MWP:A | 99 | 103 |
| 1344 | 947 | KRIRK | 1BGF:A | 84 | 88 |
| 1345 | 948 | KRLRK | 3PP2:A | 513 | 517 |
| 1346 |  | KRLRK | 3RMI:A | 60 | 64 |
| 1347 | 949 | KSISK | 1LQT:A | 61 | 65 |
| 1348 | 950 | KSKSK | 1QSA:A | 409 | 413 |
| 1349 | 951 | KSPSK | 3BPT:A | 233 | 237 |
| 1350 |  | KSPSK | 3NBM:A | 543 | 547 |
| 1351 | 952 | KSSSK | 1K55:A | 45 | 49 |
| 1352 | 953 | KSTSK | 1TVG:A | 76 | 80 |
| 1353 | 954 | KSVSK | 3ALJ:A | 89 | 93 |
| 1354 | 955 | KSYSK | 3NSU:A | 483 | 487 |
| 1355 | 956 | KTFTK | 2WUJ:B | 11 | 15 |
| 1356 | 957 | KTKTK | 1F0L:B | 212 | 216 |
| 1357 | 958 | KTLTK | 2BLA:A | 138 | 142 |
| 1358 | 959 | KTRTK | 3AJD:A | 118 | 122 |
| 1359 | 960 | KTSTK | 1R0U:A | 97 | 101 |
| 1360 | 961 | KTTTK | 1QWZ:A | 174 | 178 |
| 1361 |  | KTTTK | 3DRF:A | 213 | 217 |
| 1362 | 962 | KTWTK | 3PCT:C | 88 | 92 |
| 1363 | 963 | KVAVK | 3MDY:A | 227 | 231 |
| 1364 | 964 | KVDVK | 1I1J:B | 94 | 98 |
| 1365 |  | KVDVK | 3M1I:B | 73 | 77 |
| 1366 | 965 | KVFVK | 1VMH:A | 126 | 130 |
| 1367 | 966 | KVHVK | 3RTL:A | 432 | 436 |
| 1368 | 967 | KVKVK | 1OB9:A | 60 | 64 |
| 1369 |  | KVKVK | 2Z0T:A | 48 | 52 |
| 1370 | 968 | KVLVK | 1P4X:A | 72 | 76 |
| 1371 | 969 | KVNVK | 2BBA:A | 158 | 162 |
| 1372 | 970 | KVRVK | 3PLU:B | 13 | 17 |
| 1373 |  | KVRVK | 1H72:C | 6 | 10 |
| 1374 | 971 | KVSVK | 2QIF:B | 50 | 54 |
| 1375 | 972 | KVVAVVK | 3CJS:C | 3 | 9 |
| 1376 | 973 | KVVVK | 3H43:A | 105 | 109 |
| 1377 |  | KVVVK | 1HT6:A | 371 | 375 |
| 1378 |  | KVVVK | 3O0A:B | 385 | 389 |
| 1379 |  | KVVVK | 3JSY:A | 164 | 168 |
| 1380 | 974 | KWCWK | 1WWZ:B | 47 | 51 |
| 1381 | 975 | KWFWK | 1ZVD:A | 654 | 658 |
| 1382 | 976 | KYFYK | 3S9J:A | 350 | 354 |
| 1383 | 977 | KYIIYK | 2XFA:B | 25 | 30 |
| 1384 | 978 | KYLPLYK | 2VSH:B | 67 | 73 |
| 1385 | 979 | KYYYK | 3HYN:A | 183 | 187 |
| 1386 | 980 | LAAAL | 2AXW:B | 123 | 127 |
| 1387 |  | LAAAL | 1H41:B | 311 | 315 |
| 1388 |  | LAAAL | 3VNY:A | 477 | 481 |
| 1389 |  | LAAAL | 3PBT:A | 99 | 103 |
| 1390 |  | LAAAL | 1WP5:A | 314 | 318 |
| 1391 |  | LAAAL | 1N4W:A | 233 | 237 |
| 1392 |  | LAAAL | 3H4T:A | 535 | 539 |
| 1393 |  | LAAAL | 3NVS:A | 102 | 106 |
| 1394 | 981 | LACAL | 1P28:A | 45 | 49 |
| 1395 | 982 | LADAL | 1SC6:A | 249 | 253 |
| 1396 |  | LADAL | 2GKM:B | 98 | 102 |
| 1397 |  | LADAL | 2VGO:A | 200 | 204 |
| 1398 |  | LADAL | 3DR5:A | 161 | 165 |
| 1399 | 983 | LADAVADAL | 3LHI:A | 17 | 25 |
| 1400 | 984 | LAEAL | 1VMG:A | 39 | 43 |
| 1401 |  | LAEAL | 2WZV:B | 73 | 77 |
| 1402 |  | LAEAL | 1U7I:A | 93 | 97 |
| 1403 |  | LAEAL | 3GRD:B | 24 | 28 |
| 1404 |  | LAEAL | 2VH3:A | 18 | 22 |
| 1405 | 985 | LAERREAL | 1K0D:B | 263 | 270 |
| 1406 | 986 | LAGAL | 1WWI:A | 110 | 114 |
| 1407 |  | LAGAL | 2VFR:A | 397 | 401 |
| 1408 | 987 | LAHAL | 1QOP:B | 355 | 359 |
| 1409 | 988 | LAIAL | 1M56:C | 170 | 174 |
| 1410 |  | LAIAL | 3TJY:A | 204 | 208 |
| 1411 | 989 | LAKAL | 2GN4:A | 242 | 246 |
| 1412 |  | LAKAL | 1Z6O:A | 126 | 130 |
| 1413 |  | LAKAL | 2IYV:A | 22 | 26 |
| 1414 |  | LAKAL | 1MGT:A | 115 | 119 |
| 1415 |  | LAKAL | 1GK8:A | 314 | 318 |
| 1416 | 990 | LALAL | 1GUQ:A | 255 | 259 |
| 1417 |  | LALAL | 3B9W:A | 330 | 334 |
| 1418 |  | LALAL | 2J8C:L | 185 | 189 |
| 1419 | 991 | LAMAL | 3KAN:C | 45 | 49 |
| 1420 | 992 | LANAL | 2OXC:A | 250 | 254 |
| 1421 |  | LANAL | 1ZMT:A | 155 | 159 |
| 1422 | 993 | LAQAL | 3O7I:B | 98 | 102 |
| 1423 |  | LAQAL | 3D9S:D | 56 | 60 |
| 1424 |  | LAQAL | 3PB6:X | 197 | 201 |
| 1425 | 994 | LAQQAL | 2CI1:A | 24 | 29 |
| 1426 | 995 | LASAL | 1WMG:F | 923 | 927 |
| 1427 |  | LASAL | 2CWZ:D | 71 | 75 |
| 1428 |  | LASAL | 3GA7:A | 172 | 176 |
| 1429 |  | LASAL | 2W7Z:A | 202 | 206 |
| 1430 |  | LASAL | 2V9L:A | 268 | 272 |
| 1431 |  | LASAL | 3SQN:B | 278 | 282 |
| 1432 |  | LASAL | 2PD1:D | 24 | 28 |
| 1433 |  | LASAL | 3CI3:A | 120 | 124 |
| 1434 |  | LASAL | 1O9G:A | 200 | 204 |
| 1435 |  | LASAL | 1QOP:B | 121 | 125 |
| 1436 | 996 | LASGGSAL | 1APY:A | 24 | 31 |
| 1437 | 997 | LATAL | 2BNL:F | 84 | 88 |
| 1438 |  | LATAL | 1Y7Y:B | 61 | 65 |
| 1439 |  | LATAL | 3H4T:A | 346 | 350 |
| 1440 | 998 | LAYAL | 3MK6:C | 334 | 338 |
| 1441 | 999 | LCTCL | 3M7P:A | 332 | 336 |
| 1442 | 1000 | LDAGADL | 3QU5:B | 201 | 207 |
| 1443 | 1001 | LDDDL | 1GKM:A | 91 | 95 |
| 1444 |  | LDDDL | 2Q0I:A | 10 | 14 |
| 1445 | 1002 | LDEDL | 3NKE:B | 107 | 111 |
| 1446 | 1003 | LDFDL | 2WNO:A | 178 | 182 |
| 1447 | 1004 | LDGDL | 4ACJ:A | 764 | 768 |
| 1448 | 1005 | LDIDL | 1R4V:A | 40 | 44 |
| 1449 | 1006 | LDKDL | 3PJP:B | 1296 | 1300 |
| 1450 | 1007 | LDKIKDL | 2P4F:A | 107 | 113 |
| 1451 | 1008 | LDLDL | 1VR9:B | 54 | 58 |
| 1452 | 1009 | LDNDL | 3P7X:A | 30 | 34 |
| 1453 | 1010 | LDNNDL | 3EOJ:A | 153 | 158 |
| 1454 | 1011 | LDQDL | 1F35:B | 1017 | 1021 |
| 1455 |  | LDQDL | 3RSN:A | 163 | 167 |
| 1456 | 1012 | LDRDL | 3AQI:B | 594 | 598 |
| 1457 | 1013 | LEAEL | 3OG4:A | 72 | 76 |
| 1458 |  | LEAEL | 2I74:A | 611 | 615 |
| 1459 |  | LEAEL | 2PN0:C | 35 | 39 |
| 1460 | 1014 | LECEL | 2RIK:A | 208 | 212 |
| 1461 | 1015 | LEDEL | 1VKK:A | 45 | 49 |
| 1462 |  | LEDEL | 2E6F:A | 285 | 289 |
| 1463 | 1016 | LEEEL | 1KQ3:A | 21 | 25 |
| 1464 |  | LEEEL | 3M1E:A | 39 | 43 |
| 1465 |  | LEEEL | 1TZD:B | 269 | 273 |
| 1466 | 1017 | LEFEL | 3HE4:B | 34 | 38 |
| 1467 | 1018 | LEGEL | 1YQH:A | 47 | 51 |
| 1468 | 1019 | LEIEL | 3G91:A | 253 | 257 |
| 1469 | 1020 | LEIIEL | 1JLY:B | 294 | 299 |
| 1470 | 1021 | LEKEL | 1YDX:A | 30 | 34 |
| 1471 |  | LEKEL | 3PJ0:D | 354 | 358 |
| 1472 |  | LEKEL | 3RH3:B | 224 | 228 |
| 1473 |  | LEKEL | 3V46:A | 383 | 387 |
| 1474 |  | LEKEL | 3D3M:A | 733 | 737 |
| 1475 |  | LEKEL | 2ODM:A | 77 | 81 |
| 1476 | 1022 | LELEL | 1I4D:A | 28 | 32 |
| 1477 |  | LELEL | 3BGY:A | 211 | 215 |
| 1478 | 1023 | LEQEL | 3M0F:A | 141 | 145 |
| 1479 |  | LEQEL | 3L4Q:D | 536 | 540 |
| 1480 | 1024 | LEREL | 1INL:A | 7 | 11 |
| 1481 |  | LEREL | 3MTS:C | 93 | 97 |
| 1482 | 1025 | LESEL | 3PW3:F | 65 | 69 |
| 1483 |  | LESEL | 3BGY:A | 224 | 228 |
| 1484 | 1026 | LEVEL | 2J8C:H | 179 | 183 |
| 1485 |  | LEVEL | 1N2F:A | 97 | 101 |
| 1486 | 1027 | LFDFL | 1T0H:B | 249 | 253 |
| 1487 |  | LFDFL | 2W4J:A | 101 | 105 |
| 1488 | 1028 | LFEEFL | 1M5Q:2 | 132 | 137 |
| 1489 | 1029 | LFIFL | 2ZKM:X | 791 | 795 |
| 1490 | 1030 | LGAGL | 3DR5:A | 203 | 207 |
| 1491 | 1031 | LGFGL | 3CT5:A | 63 | 67 |
| 1492 |  | LGFGL | 3AMR:A | 291 | 295 |
| 1493 |  | LGFGL | 3MD9:A | 258 | 262 |
| 1494 | 1032 | LGGGGL | 2ZPU:A | 182 | 187 |
| 1495 |  | LGGGGL | 1XOD:A | 31 | 36 |
| 1496 | 1033 | LGGGL | 2CLB:A | 84 | 88 |
| 1497 | 1034 | LGHGL | 1UG6:A | 197 | 201 |
| 1498 | 1035 | LGIGL | 2E10:B | 126 | 130 |
| 1499 |  | LGIGL | 1ZJZ:A | 16 | 20 |
| 1500 | 1036 | LGKGL | 2H8G:B | 56 | 60 |
| 1501 |  | LGKGL | 3EEI:A | 83 | 87 |
| 1502 |  | LGKGL | 3SZY:A | 137 | 141 |
| 1503 |  | LGKGL | 1WZD:A | 211 | 215 |
| 1504 |  | LGKGL | 2BUE:A | 124 | 128 |
| 1505 | 1037 | LGLGL | 1GVE:B | 57 | 61 |
| 1506 |  | LGLGL | 1Z3E:B | 306 | 310 |
| 1507 |  | LGLGL | 1G8M:A | 28 | 32 |
| 1508 | 1038 | LGLKLGL | 3NPK:A | 201 | 207 |
| 1509 | 1039 | LGNGL | 3EY6:A | 40 | 44 |
| 1510 | 1040 | LGQGL | 1PZX:A | 124 | 128 |
| 1511 |  | LGQGL | 2QML:A | 117 | 121 |
| 1512 | 1041 | LGRGL | 3C7X:A | 406 | 410 |
| 1513 |  | LGRGL | 1YA5:T | 74 | 78 |
| 1514 | 1042 | LGTCTGL | 2Y8N:B | 59 | 65 |
| 1515 | 1043 | LGTGL | 3RQA:D | 161 | 165 |
| 1516 | 1044 | LGVGL | 2IYV:A | 26 | 30 |
| 1517 | 1045 | LGYGL | 3BMX:A | 630 | 634 |
| 1518 |  | LGYGL | 1M0K:A | 62 | 66 |
| 1519 |  | LGYGL | 1IAZ:A | 154 | 158 |
| 1520 | 1046 | LGYYGL | 3HHT:A | 91 | 96 |
| 1521 | 1047 | LHSHL | 3UXJ:D | 185 | 189 |
| 1522 | 1048 | LHVHL | 3IAR:A | 14 | 18 |
| 1523 | 1049 | LIAIL | 1KGD:A | 826 | 830 |
| 1524 | 1050 | LIDNDIL | 1JF8:A | 66 | 72 |
| 1525 | 1051 | LIEIL | 3BQP:B | 58 | 62 |
| 1526 |  | LIEIL | 2ZHJ:A | 109 | 113 |
| 1527 | 1052 | LIFIL | 2A0J:A | 99 | 103 |
| 1528 | 1053 | LIGIL | 1TZD:B | 449 | 453 |
| 1529 | 1054 | LIHIL | 3RMI:A | 24 | 28 |
| 1530 | 1055 | LIKIL | 2ZP1:A | 295 | 299 |
| 1531 |  | LIKIL | 3UFE:B | 82 | 86 |
| 1532 | 1056 | LILIL | 3LRU:A | 1936 | 1940 |
| 1533 | 1057 | LINIL | 1UOC:A | 170 | 174 |
| 1534 |  | LINIL | 3ACX:A | 166 | 170 |
| 1535 | 1058 | LIRIL | 3ELN:A | 15 | 19 |
| 1536 |  | LIRIL | 2XF3:A | 113 | 117 |
| 1537 | 1059 | LISIL | 2XTC:A | 49 | 53 |
| 1538 | 1060 | LITIL | 3ZSJ:A | 131 | 135 |
| 1539 | 1061 | LKAKL | 1OD6:A | 154 | 158 |
| 1540 | 1062 | LKCCKL | 2UWI:B | 34 | 39 |
| 1541 | 1063 | LKDKL | 3FZ4:A | 69 | 73 |
| 1542 |  | LKDKL | 2QSX:B | 207 | 211 |
| 1543 | 1064 | LKEKL | 1VKK:A | 134 | 138 |
| 1544 |  | LKEKL | 1RRE:E | 719 | 723 |
| 1545 |  | LKEKL | 3IOF:A | 223 | 231 |
| 1546 |  | LKEKL | 3MSX:B | 410 | 414 |
| 1547 |  | LKEKL | 2XUS:B | 74 | 78 |
| 1548 |  | LKEKL | 1VHU:A | 119 | 123 |
| 1549 | 1065 | LKENEKL | 1T6F:A | 6 | 12 |
| 1550 | 1066 | LKGKL | 2RH0:A | 62 | 66 |
| 1551 |  | LKGKL | 3IQ2:A | 80 | 84 |
| 1552 | 1067 | LKIIKL | 3KP7:B | 94 | 99 |
| 1553 | 1068 | LKIKL | 3RQT:A | 312 | 316 |
| 1554 | 1069 | LKKKL | 1MXR:B | 189 | 193 |
| 1555 | 1070 | LKLKL | 2QIY:B | 79 | 83 |
| 1556 |  | LKLKL | 2FTX:B | 160 | 164 |
| 1557 | 1071 | LKPKL | 2VSM:A | 198 | 202 |
| 1558 |  | LKPKL | 2FL7:A | 96 | 100 |
| 1559 | 1072 | LKQKL | 3PT3:A | 2783 | 2787 |
| 1560 |  | LKQKL | 2AO9:F | 20 | 24 |
| 1561 |  | LKQKL | 2VVW:A | 100 | 104 |
| 1562 | 1073 | LKQQKL | 3CU2:B | 10 | 15 |
| 1563 | 1074 | LKRKL | 3KEV:A | 80 | 84 |
| 1564 | 1075 | LKSKL | 1WPA:A | 503 | 507 |
| 1565 |  | LKSKL | 1KD8:B | 12 | 16 |
| 1566 | 1076 | LKVKL | 2IMF:A | 56 | 60 |
| 1567 | 1077 | LKYKL | 3G91:A | 122 | 126 |
| 1568 | 1078 | LLALL | 3EAR:B | 414 | 418 |
| 1569 |  | LLALL | 1QQF:A | 1237 | 1241 |
| 1570 |  | LLALL | 1W9M:A | 501 | 505 |
| 1571 |  | LLALL | 1IQ4:B | 169 | 173 |
| 1572 | 1079 | LLDAGADLL | 3QU5:B | 200 | 208 |
| 1573 | 1080 | LLDLL | 3IAR:A | 343 | 347 |
| 1574 |  | LLDLL | 3Q64:A | 142 | 146 |
| 1575 |  | LLDLL | 3PB6:X | 267 | 271 |
| 1576 | 1081 | LLELL | 2P5M:C | 145 | 149 |
| 1577 |  | LLELL | 2FBI:A | 128 | 132 |
| 1578 |  | LLELL | 3CDL:B | 83 | 87 |
| 1579 | 1082 | LLGLL | 3THR:D | 48 | 52 |
| 1580 | 1083 | LLKLL | 2AXI:A | 34 | 38 |
| 1581 |  | LLKLL | 2GN4:A | 329 | 333 |
| 1582 |  | LLKLL | 1A1X:A | 101 | 105 |
| 1583 |  | LLKLL | 2HKX:B | 124 | 128 |
| 1584 | 1084 | LLMLL | 1UOC:A | 252 | 256 |
| 1585 |  | LLMLL | 3HXI:A | 117 | 121 |
| 1586 | 1085 | LLNLL | 1NZ0:A | 108 | 112 |
| 1587 | 1086 | LLPLL | 3GE3:B | 246 | 250 |
| 1588 |  | LLPLL | 3F1L:B | 135 | 139 |
| 1589 |  | LLPLL | 3FRH:A | 175 | 179 |
| 1590 | 1087 | LLPPLL | 2OJ6:A | 374 | 379 |
| 1591 |  | LLPPLL | 1V8D:C | 96 | 101 |
| 1592 | 1088 | LLQLL | 2BKR:A | 186 | 190 |
| 1593 | 1089 | LLRLL | 1ES9:A | 212 | 216 |
| 1594 |  | LLRLL | 3U52:C | 119 | 123 |
| 1595 |  | LLRLL | 1OMZ:A | 80 | 84 |
| 1596 |  | LLRLL | 2D1P:F | 17 | 21 |
| 1597 | 1090 | LLSLL | 1XL3:A | 106 | 110 |
| 1598 |  | LLSLL | 3JWI:A | 43 | 47 |
| 1599 | 1091 | LLTLL | 2Z72:A | 86 | 90 |
| 1600 |  | LLTLL | 1KWF:A | 378 | 382 |
| 1601 |  | LLTLL | 1W9H:A | 324 | 328 |
| 1602 |  | LLTLL | 3OII:A | 96 | 100 |
| 1603 | 1092 | LMRML | 1R8S:A | 107 | 111 |
| 1604 | 1093 | LNDNL | 256B:A | 10 | 14 |
| 1605 |  | LNDNL | 3LW6:A | 151 | 155 |
| 1606 | 1094 | LNENL | 2B0A:A | 150 | 154 |
| 1607 | 1095 | LNGNL | 2VPT:A | 128 | 132 |
| 1608 | 1096 | LNINL | 1LC5:A | 178 | 182 |
| 1609 | 1097 | LNKNL | 1W7C:A | 186 | 190 |
| 1610 |  | LNKNL | 2OFW:C | 95 | 99 |
| 1611 | 1098 | LNMNL | 3Q39:A | 422 | 426 |
| 1612 | 1099 | LNNNL | 2IN5:B | 82 | 86 |
| 1613 |  | LNNNL | 3KV1:A | 46 | 50 |
| 1614 | 1100 | LNPNL | 3ZZP:A | 52 | 56 |
| 1615 | 1101 | LNQNL | 3GSZ:B | 439 | 443 |
| 1616 | 1102 | LNTFTNL | 1PP0:A | 104 | 110 |
| 1617 | 1103 | LNYNL | 3G3Z:A | 27 | 31 |
| 1618 | 1104 | LPASAPL | 2D0O:D | 77 | 83 |
| 1619 | 1105 | LPEPL | 1OIH:D | 147 | 151 |
| 1620 | 1106 | LPGPL | 3GMI:A | 83 | 87 |
| 1621 | 1107 | LPIPL | 2NWF:A | 171 | 175 |
| 1622 |  | LPIPL | 2OSA:A | 350 | 354 |
| 1623 | 1108 | LPKPL | 1K5N:A | 266 | 270 |
| 1624 |  | LPKPL | 3RPD:B | 23 | 27 |
| 1625 | 1109 | LPQPL | 1LTZ:A | 29 | 33 |
| 1626 | 1110 | LPRRPL | 1WV9:A | 51 | 56 |
| 1627 | 1111 | LPSNSPL | 2VPA:A | 99 | 105 |
| 1628 | 1112 | LPSPL | 2APL:A | 90 | 94 |
| 1629 | 1113 | LPYPL | 1YRB:A | 122 | 126 |
| 1630 | 1114 | LQKQL | 1FO0:B | 39 | 43 |
| 1631 |  | LQKQL | 2WY8:Q | 62 | 66 |
| 1632 | 1115 | LQLLQL | 1YMT:A | 229 | 234 |
| 1633 | 1116 | LQNNQL | 2FP8:B | 111 | 116 |
| 1634 | 1117 | LQQQL | 2RAF:C | 94 | 98 |
| 1635 |  | LQQQL | 1T82:D | 69 | 73 |
| 1636 |  | LQQQL | 2XDJ:F | 25 | 29 |
| 1637 | 1118 | LQRQL | 1SDI:A | 116 | 120 |
| 1638 | 1119 | LQSQL | 1LYV:A | 263 | 267 |
| 1639 |  | LQSQL | 1UCD:A | 61 | 65 |
| 1640 |  | LQSQL | 2ZXK:A | 77 | 81 |
| 1641 | 1120 | LQTQL | 3AJ6:B | 154 | 158 |
| 1642 | 1121 | LQVQL | 2V6V:B | 324 | 328 |
| 1643 |  | LQVQL | 3JYB:B | 91 | 95 |
| 1644 | 1122 | LQVVQL | 2CI1:A | 59 | 64 |
| 1645 | 1123 | LRARL | 3HNA:B | 1093 | 1097 |
| 1646 |  | LRARL | 3HNX:A | 76 | 80 |
| 1647 | 1124 | LRCRL | 2ECU:A | 103 | 107 |
| 1648 | 1125 | LRDEDRL | 1EYH:A | 134 | 140 |
| 1649 | 1126 | LRDRL | 3CZ8:A | 229 | 233 |
| 1650 |  | LRDRL | 3P42:D | 54 | 58 |
| 1651 | 1127 | LRERL | 3A1B:A | 475 | 479 |
| 1652 |  | LRERL | 1O9G:A | 134 | 138 |
| 1653 | 1128 | LRGRL | 2Q0I:A | 225 | 229 |
| 1654 | 1129 | LRHRL | 1G5H:C | 230 | 234 |
| 1655 | 1130 | LRLRL | 2ILK:A | 101 | 105 |
| 1656 |  | LRLRL | 3OD3:A | 231 | 235 |
| 1657 |  | LRLRL | 2XUS:B | 86 | 90 |
| 1658 |  | LRLRL | 1PRZ:A | 224 | 228 |
| 1659 | 1131 | LRNRL | 1I24:A | 226 | 230 |
| 1660 | 1132 | LRPRL | 2OZF:A | 150 | 154 |
| 1661 | 1133 | LRRRL | 3CXK:B | 19 | 23 |
| 1662 | 1134 | LRSRL | 2GDQ:B | 231 | 235 |
| 1663 |  | LRSRL | 1NKI:A | 116 | 120 |
| 1664 | 1135 | LRTRL | 1B0U:A | 91 | 95 |
| 1665 | 1136 | LRVRL | 3TIW:A | 92 | 96 |
| 1666 | 1137 | LRYRL | 3IJM:A | 121 | 125 |
| 1667 | 1138 | LSASL | 1RTQ:A | 56 | 60 |
| 1668 |  | LSASL | 2VWS:A | 180 | 184 |
| 1669 | 1139 | LSDPDSL | 2FZV:A | 6 | 12 |
| 1670 | 1140 | LSDSL | 1I24:A | 352 | 356 |
| 1671 |  | LSDSL | 1EXZ:B | 252 | 256 |
| 1672 |  | LSDSL | 3C1Q:A | 118 | 122 |
| 1673 | 1141 | LSEESL | 3TGN:A | 47 | 52 |
| 1674 | 1142 | LSHSL | 2ATR:A | 38 | 42 |
| 1675 | 1143 | LSLSL | 1MUN:A | 126 | 130 |
| 1676 |  | LSLSL | 1A79:C | 43 | 47 |
| 1677 |  | LSLSL | 3DNT:A | 41 | 45 |
| 1678 | 1144 | LSNSL | 3GSZ:B | 26 | 30 |
| 1679 | 1145 | LSPSL | 3KAE:A | 191 | 195 |
| 1680 |  | LSPSL | 3FUY:C | 9 | 13 |
| 1681 | 1146 | LSRSL | 3CLM:A | 19 | 23 |
| 1682 |  | LSRSL | 1KMV:A | 89 | 93 |
| 1683 | 1147 | LSSSL | 1Z4V:A | 545 | 549 |
| 1684 |  | LSSSL | 3Q7Z:A | 509 | 513 |
| 1685 |  | LSSSL | 1U5K:A | 46 | 50 |
| 1686 | 1148 | LSTSL | 3CEG:A | 4557 | 4561 |
| 1687 | 1149 | LSVSL | 2HZY:A | 299 | 303 |
| 1688 | 1150 | LTATL | 1HW7:A | 57 | 61 |
| 1689 |  | LTATL | 3F5O:H | 59 | 63 |
| 1690 | 1151 | LTCTL | 3BJ9:1 | 20 | 24 |
| 1691 |  | LTCTL | 2H00:C | 18 | 22 |
| 1692 | 1152 | LTETL | 3OMT:B | 24 | 28 |
| 1693 | 1153 | LTGTL | 3HRG:A | 217 | 221 |
| 1694 | 1154 | LTHTL | 2WHL:A | 153 | 157 |
| 1695 | 1155 | LTIITL | 3D06:A | 252 | 257 |
| 1696 | 1156 | LTITL | 2CPG:A | 5 | 9 |
| 1697 |  | LTITL | 1QHV:A | 532 | 536 |
| 1698 |  | LTITL | 3ON9:B | 195 | 199 |
| 1699 | 1157 | LTKKTL | 1TQJ:C | 60 | 65 |
| 1700 | 1158 | LTLTL | 2I5I:B | 64 | 68 |
| 1701 |  | LTLTL | 3P2U:B | 3 | 7 |
| 1702 |  | LTLTL | 2FBI:A | 8 | 12 |
| 1703 |  | LTLTL | 2Q5R:C | 3 | 7 |
| 1704 |  | LTLTL | 2NXY:B | 1114 | 1118 |
| 1705 |  | LTLTL | 1QVE:A | 113 | 117 |
| 1706 | 1159 | LTLYLTL | 3HUL:B | 23 | 29 |
| 1707 | 1160 | LTQQTL | 1FG7:A | 47 | 52 |
| 1708 | 1161 | LTQTL | 2B0A:A | 5 | 9 |
| 1709 | 1162 | LTSTL | 3PPQ:A | 96 | 100 |
| 1710 | 1163 | LTTTL | 3G2B:A | 80 | 84 |
| 1711 |  | LTTTL | 1UF5:B | 260 | 264 |
| 1712 | 1164 | LTVTL | 3NRE:A | 127 | 131 |
| 1713 | 1165 | LTYTL | 3TU8:A | 202 | 206 |
| 1714 | 1166 | LVAVL | 2GB4:B | 176 | 180 |
| 1715 |  | LVAVL | 2PKF:B | 282 | 286 |
| 1716 | 1167 | LVDVL | 3ARC:K | 21 | 25 |
| 1717 | 1168 | LVEEVL | 1QQ5:B | 155 | 160 |
| 1718 | 1169 | LVGVL | 3M0Z:A | 36 | 40 |
| 1719 | 1170 | LVKVL | 3BNJ:A | 428 | 432 |
| 1720 |  | LVKVL | 2QQB:A | 116 | 120 |
| 1721 | 1171 | LVNVL | 3LL8:A | 365 | 369 |
| 1722 |  | LVNVL | 3QZR:A | 53 | 57 |
| 1723 |  | LVNVL | 1VL7:A | 66 | 70 |
| 1724 |  | LVNVL | 1YDL:A | 49 | 53 |
| 1725 | 1172 | LVPVL | 3O0A:B | 288 | 292 |
| 1726 |  | LVPVL | 3KUU:C | 90 | 94 |
| 1727 |  | LVPVL | 3V5A:A | 407 | 411 |
| 1728 | 1173 | LVQQVL | 2XFR:A | 360 | 365 |
| 1729 | 1174 | LVRVL | 3RQZ:C | 48 | 52 |
| 1730 |  | LVRVL | 1MKF:B | 104 | 108 |
| 1731 | 1175 | LVSVL | 3PA6:C | 70 | 74 |
| 1732 |  | LVSVL | 3CTZ:A | 107 | 111 |
| 1733 |  | LVSVL | 1QDD:A | 50 | 54 |
| 1734 |  | LVSVL | 2APL:A | 78 | 82 |
| 1735 | 1176 | LVTVL | 1Z9M:B | 133 | 137 |
| 1736 |  | LVTVL | 1QSA:A | 172 | 176 |
| 1737 | 1177 | LVVVL | 1M56:C | 48 | 52 |
| 1738 | 1178 | LVWVL | 1LKE:A | 127 | 131 |
| 1739 | 1179 | LWIWL | 1YIS:A | 37 | 41 |
| 1740 | 1180 | LYAYL | 3D3S:D | 30 | 34 |
| 1741 | 1181 | LYDYL | 3MDY:A | 287 | 291 |
| 1742 | 1182 | LYEYL | 2X3H:C | 19 | 23 |
| 1743 |  | LYEYL | 3GMI:A | 284 | 288 |
| 1744 | 1183 | LYFYL | 3D9S:D | 220 | 224 |
| 1745 | 1184 | LYIIYL | 2HEW:F | 101 | 106 |
| 1746 | 1185 | LYIYL | 1LUZ:B | 26 | 30 |
| 1747 | 1186 | LYKYL | 3CM3:A | 1008 | 1012 |
| 1748 |  | LYKYL | 3CPX:A | 294 | 298 |
| 1749 | 1187 | LYPYL | 1QSA:A | 37 | 41 |
| 1750 | 1188 | LYRYL | 2PTZ:A | 128 | 132 |
| 1751 | 1189 | LYVDDVYL | 2ZEX:A | 136 | 143 |
| 1752 | 1190 | LYYYL | 4A69:C | 468 | 472 |
| 1753 | 1191 | MAKAM | 2UXY:A | 203 | 207 |
| 1754 | 1192 | MATAM | 1R29:A | 106 | 110 |
| 1755 | 1193 | MELEM | 2HZY:A | 198 | 202 |
| 1756 | 1194 | MIVIM | 1WL8:A | 2 | 6 |
| 1757 | 1195 | MKIKM | 3R8J:A | 81 | 85 |
| 1758 | 1196 | MKQKM | 2GVG:A | 368 | 372 |
| 1759 | 1197 | MLPLM | 2B06:A | 125 | 129 |
| 1760 | 1198 | MRLLRM | 3ZX3:A | 134 | 139 |
| 1761 | 1199 | MTSTM | 2CVI:A | 68 | 72 |
| 1762 | 1200 | MVLVM | 2V3I:A | 360 | 364 |
| 1763 | 1201 | MVMVM | 3GDC:C | 176 | 180 |
| 1764 | 1202 | MYEYM | 1F0L:B | 178 | 182 |
| 1765 | 1203 | NAAAN | 1OQV:A | 101 | 105 |
| 1766 |  | NAAAN | 1G4Y:B | 422 | 426 |
| 1767 | 1204 | NAKAN | 1SO2:D | 906 | 910 |
| 1768 | 1205 | NALAN | 3QFH:B | 74 | 78 |
| 1769 | 1206 | NALLAN | 2TNF:C | 34 | 39 |
| 1770 | 1207 | NANAN | 1QWY:A | 98 | 102 |
| 1771 | 1208 | NAPAN | 3MNM:A | 550 | 554 |
| 1772 | 1209 | NASAN | 2WAO:A | 167 | 171 |
| 1773 | 1210 | NDGDN | 3IMH:A | 93 | 97 |
| 1774 | 1211 | NDIDN | 3MSE:B | 141 | 145 |
| 1775 | 1212 | NEAEN | 3HRQ:B | 1514 | 1518 |
| 1776 |  | NEAEN | 3JQL:A | 53 | 57 |
| 1777 |  | NEAEN | 1G1S:A | 71 | 75 |
| 1778 | 1213 | NEEEEN | 3LUM:D | 76 | 81 |
| 1779 | 1214 | NEIEN | 2QL8:A | 114 | 118 |
| 1780 | 1215 | NEKEN | 3N3M:A | 35 | 39 |
| 1781 | 1216 | NEKVKEN | 1V4P:A | 63 | 69 |
| 1782 | 1217 | NELEN | 1F00:I | 872 | 876 |
| 1783 | 1218 | NELLEN | 1G5H:C | 378 | 383 |
| 1784 | 1219 | NESEN | 2BOU:A | 87 | 91 |
| 1785 | 1220 | NFDFN | 3H9M:A | 374 | 378 |
| 1786 | 1221 | NFYFN | 1ZRH:A | 212 | 216 |
| 1787 | 1222 | NGFGN | 1UUQ:A | 100 | 104 |
| 1788 | 1223 | NGGGGN | 1GXM:B | 378 | 383 |
| 1789 | 1224 | NGPGN | 1YB0:A | 79 | 83 |
| 1790 | 1225 | NGQQGN | 1FNF:A | 1183 | 1188 |
| 1791 | 1226 | NGVGN | 1M70:A | 126 | 130 |
| 1792 |  | NGVGN | 2Y6H:A | 62 | 66 |
| 1793 | 1227 | NHFHN | 3FX7:B | 34 | 38 |
| 1794 | 1228 | NHPHN | 2O71:A | 151 | 155 |
| 1795 | 1229 | NIIIN | 2W39:A | 246 | 250 |
| 1796 |  | NIIIN | 3MSE:B | 20 | 24 |
| 1797 | 1230 | NIKIN | 3MW4:A | 246 | 250 |
| 1798 | 1231 | NILIN | 2QOL:A | 751 | 755 |
| 1799 | 1232 | NIPIN | 3EA6:A | 94 | 98 |
| 1800 | 1233 | NISIN | 1H72:C | 178 | 182 |
| 1801 | 1234 | NITIN | 3S8K:A | 84 | 88 |
| 1802 | 1235 | NIWIN | 3EA6:A | 98 | 102 |
| 1803 | 1236 | NKPKN | 1FC3:C | 140 | 144 |
| 1804 | 1237 | NKVKN | 3BI1:A | 212 | 216 |
| 1805 | 1238 | NKYKN | 2Y8D:A | 2332 | 2336 |
| 1806 | 1239 | NKYYKN | 2WUX:A | 18 | 23 |
| 1807 | 1240 | NLALN | 2NT0:D | 382 | 386 |
| 1808 | 1241 | NLDLN | 3U9Q:A | 308 | 312 |
| 1809 |  | NLDLN | 1G61:B | 4092 | 4096 |
| 1810 | 1242 | NLELN | 1O6V:B | 321 | 325 |
| 1811 |  | NLELN | 2A0M:A | 71 | 75 |
| 1812 | 1243 | NLILN | 2QKP:A | 333 | 337 |
| 1813 | 1244 | NLLLN | 1G6G:A | 98 | 102 |
| 1814 |  | NLLLN | 3KOP:E | 118 | 122 |
| 1815 | 1245 | NLNLN | 1MID:A | 60 | 64 |
| 1816 |  | NLNLN | 4A8J:F | 218 | 222 |
| 1817 | 1246 | NLRLN | 3EN0:C | 247 | 251 |
| 1818 | 1247 | NLVLN | 2F5V:A | 220 | 224 |
| 1819 | 1248 | NMHMN | 1MWP:A | 42 | 46 |
| 1820 | 1249 | NNDNN | 3D9X:A | 392 | 396 |
| 1821 |  | NNDNN | 3K7C:D | 90 | 94 |
| 1822 | 1250 | NNGNN | 3RLG:A | 119 | 123 |
| 1823 | 1251 | NNHNN | 1BEC:A | 27 | 31 |
| 1824 | 1252 | NNSNN | 3OYV:A | 238 | 242 |
| 1825 | 1253 | NNTNN | 3F02:A | 157 | 161 |
| 1826 | 1254 | NNVNN | 3GWL:B | 177 | 181 |
| 1827 | 1255 | NNYNN | 3N3M:A | 39 | 43 |
| 1828 | 1256 | NPDFDPN | 3GQH:A | 777 | 783 |
| 1829 | 1257 | NPGGPN | 2QSK:A | 13 | 18 |
| 1830 |  | NPGGPN | 2QSK:A | 61 | 66 |
| 1831 | 1258 | NPHPN | 3QRA:A | 150 | 154 |
| 1832 | 1259 | NPPPN | 3CZ6:A | 778 | 782 |
| 1833 |  | NPPPN | 3LPH:D | 26 | 30 |
| 1834 | 1260 | NPRPN | 3B7E:A | 325 | 329 |
| 1835 | 1261 | NQLQN | 3G9K:S | 388 | 392 |
| 1836 | 1262 | NQQQN | 2POF:B | 45 | 49 |
| 1837 | 1263 | NRLRN | 3NUA:B | 231 | 235 |
| 1838 |  | NRLRN | 3N5B:B | 66 | 70 |
| 1839 | 1264 | NRVRN | 1ZWY:A | 66 | 70 |
| 1840 |  | NRVRN | 3RQT:A | 192 | 196 |
| 1841 | 1265 | NRVVRN | 3TIW:A | 85 | 90 |
| 1842 | 1266 | NSLSN | 3FX7:B | 30 | 34 |
| 1843 | 1267 | NSPSN | 3JU4:A | 561 | 565 |
| 1844 | 1268 | NTATN | 2J9O:D | 124 | 128 |
| 1845 |  | NTATN | 1GCQ:C | 641 | 645 |
| 1846 |  | NTATN | 1R4P:A | 83 | 87 |
| 1847 | 1269 | NTETN | 1I8N:A | 90 | 94 |
| 1848 | 1270 | NTFTN | 1PP0:A | 105 | 109 |
| 1849 |  | NTFTN | 2RCI:A | 141 | 145 |
| 1850 | 1271 | NTIITN | 2AS9:A | 33 | 38 |
| 1851 | 1272 | NTLTN | 1I1W:A | 102 | 106 |
| 1852 | 1273 | NVDVN | 3LRT:A | 198 | 202 |
| 1853 | 1274 | NVEVN | 2F0C:A | 53 | 57 |
| 1854 | 1275 | NVMVN | 2VBK:A | 288 | 292 |
| 1855 | 1276 | NVTFTVN | 3EVF:A | 228 | 234 |
| 1856 | 1277 | NVVVVN | 2CBP:A | 40 | 45 |
| 1857 | 1278 | NVYVN | 3FTT:A | 80 | 84 |
| 1858 | 1279 | NWGWN | 3BB7:A | 321 | 325 |
| 1859 | 1280 | NWQWN | 2CDO:C | 126 | 130 |
| 1860 | 1281 | NYEIEYN | 3P02:A | 305 | 311 |
| 1861 | 1282 | NYFYN | 1QCX:A | 228 | 232 |
| 1862 | 1283 | NYLYN | 1IAR:B | 126 | 130 |
| 1863 |  | NYLYN | 1K3I:A | 531 | 535 |
| 1864 | 1284 | NYVYN | 2QX3:B | 192 | 196 |
| 1865 | 1285 | PAAAP | 3CP7:A | 131 | 135 |
| 1866 |  | PAAAP | 1W9A:A | 91 | 95 |
| 1867 |  | PAAAP | 1OA8:A | 564 | 568 |
| 1868 | 1286 | PAFAP | 3KOP:E | 57 | 61 |
| 1869 | 1287 | PAKLKAP | 2VU6:A | 135 | 141 |
| 1870 | 1288 | PALAP | 2O7T:A | 102 | 106 |
| 1871 | 1289 | PALGLAP | 2FZV:A | 24 | 30 |
| 1872 | 1290 | PASAP | 2D0O:D | 78 | 82 |
| 1873 |  | PASAP | 3E8M:A | 120 | 124 |
| 1874 | 1291 | PATAP | 3Q6X:B | 171 | 175 |
| 1875 |  | PATAP | 1JS3:B | 43 | 47 |
| 1876 |  | PATAP | 3DB2:A | 170 | 174 |
| 1877 | 1292 | PAWAP | 2HDS:B | 140 | 144 |
| 1878 | 1293 | PAYAP | 1GVP:A | 54 | 58 |
| 1879 | 1294 | PCHCP | 2AG4:B | 106 | 110 |
| 1880 | 1295 | PCVCP | 2DS2:D | 20 | 24 |
| 1881 | 1296 | PDDDP | 2QFA:A | 69 | 73 |
| 1882 | 1297 | PDEDP | 3PT5:A | 271 | 275 |
| 1883 | 1298 | PDFDP | 3OLQ:A | 211 | 215 |
| 1884 |  | PDFDP | 3GQH:A | 778 | 782 |
| 1885 | 1299 | PDGDP | 3QZB:A | 62 | 66 |
| 1886 | 1300 | PDHDP | 2Q4H:B | 81 | 85 |
| 1887 | 1301 | PDLDP | 1UJ8:A | 31 | 35 |
| 1888 | 1302 | PDQDP | 3I7M:A | 48 | 52 |
| 1889 | 1303 | PDSDP | 3BJQ:A | 153 | 157 |
| 1890 | 1304 | PEDEP | 3TKT:A | 176 | 180 |
| 1891 | 1305 | PEEEP | 2FVV:A | 55 | 59 |
| 1892 | 1306 | PEKEP | 1NYK:A | 47 | 51 |
| 1893 | 1307 | PEWEP | 2O71:A | 129 | 133 |
| 1894 | 1308 | PFGFP | 1LWB:A | 52 | 56 |
| 1895 | 1309 | PFKFP | 3V2U:D | 234 | 238 |
| 1896 | 1310 | PFNFP | 1YFU:A | 7 | 11 |
| 1897 | 1311 | PGAGP | 2HC1:A | 1895 | 1899 |
| 1898 | 1312 | PGDGP | 3C9Q:A | 71 | 75 |
| 1899 |  | PGDGP | 1O9G:A | 48 | 52 |
| 1900 | 1313 | PGFGP | 1O04:G | 222 | 226 |
| 1901 | 1314 | PGRGP | 3C8Z:B | 13 | 17 |
| 1902 |  | PGRGP | 3R1F:A | 19 | 23 |
| 1903 | 1315 | PGSGP | 3R8J:A | 95 | 99 |
| 1904 | 1316 | PGYGP | 2J8Q:B | 206 | 210 |
| 1905 | 1317 | PHAHP | 3RQA:D | 113 | 117 |
| 1906 | 1318 | PHDHP | 2NRR:A | 471 | 475 |
| 1907 | 1319 | PHGHP | 1MN8:D | 73 | 77 |
| 1908 | 1320 | PHHHP | 3HHT:A | 15 | 19 |
| 1909 | 1321 | PHWHP | 2IUW:A | 152 | 156 |
| 1910 | 1322 | PIHIP | 3S7O:A | 28 | 32 |
| 1911 | 1323 | PILIP | 3K6I:A | 6 | 10 |
| 1912 | 1324 | PISIP | 2HEW:F | 126 | 130 |
| 1913 |  | PISIP | 3B79:A | 102 | 106 |
| 1914 | 1325 | PKCKP | 1NC7:C | 74 | 78 |
| 1915 | 1326 | PKIKP | 3H5Z:A | 409 | 413 |
| 1916 | 1327 | PKLKP | 3QQQ:B | 65 | 69 |
| 1917 | 1328 | PKNKNKP | 2P6W:A | 85 | 91 |
| 1918 | 1329 | PKTTTKP | 3DRF:A | 212 | 218 |
| 1919 | 1330 | PKWKP | 3BPT:A | 357 | 361 |
| 1920 | 1331 | PLDLP | 2BKR:A | 77 | 81 |
| 1921 |  | PLDLP | 3GSZ:B | 456 | 460 |
| 1922 | 1332 | PLGKGLP | 2H8G:B | 55 | 61 |
| 1923 | 1333 | PLGLP | 3BI1:A | 678 | 682 |
| 1924 | 1334 | PLRLP | 3TK9:A | 121 | 125 |
| 1925 |  | PLRLP | 2R31:A | 41 | 45 |
| 1926 | 1335 | PLSLP | 1MDO:A | 284 | 288 |
| 1927 | 1336 | PLSLSLP | 3DNT:A | 40 | 46 |
| 1928 | 1337 | PLTLP | 3IAR:A | 55 | 59 |
| 1929 | 1338 | PLTTLP | 3TDN:B | 71 | 76 |
| 1930 | 1339 | PLVLP | 3IOF:A | 168 | 172 |
| 1931 | 1340 | PLYLP | 3S5B:A | 207 | 211 |
| 1932 |  | PLYLP | 3ZVL:A | 350 | 354 |
| 1933 | 1341 | PNFNP | 2P0B:A | 149 | 153 |
| 1934 | 1342 | PNPNP | 3BB0:A | 121 | 125 |
| 1935 | 1343 | PNYNP | 3PQS:A | 165 | 169 |
| 1936 |  | PNYNP | 3K7I:B | 83 | 87 |
| 1937 | 1344 | PPLPP | 2IMJ:D | 9 | 13 |
| 1938 | 1345 | PPTPP | 2IC2:B | 467 | 471 |
| 1939 | 1346 | PPVKVPP | 2NTX:A | 162 | 168 |
| 1940 | 1347 | PPVPP | 3R0V:A | 125 | 129 |
| 1941 | 1348 | PPVVPP | 3G3L:A | 132 | 137 |
| 1942 | 1349 | PPYPP | 1U5K:A | 180 | 184 |
| 1943 | 1350 | PQDQP | 3IP0:A | 47 | 51 |
| 1944 | 1351 | PQNQP | 2IFT:A | 120 | 124 |
| 1945 | 1352 | PRMRP | 3R2R:A | 69 | 73 |
| 1946 | 1353 | PRVRP | 2IG6:A | 24 | 28 |
| 1947 |  | PRVRP | 3C24:B | 88 | 92 |
| 1948 |  | PRVRP | 3C8C:A | 149 | 153 |
| 1949 | 1354 | PSCECSP | 1EAI:D | 36 | 42 |
| 1950 | 1355 | PSESP | 2OV0:A | 6 | 10 |
| 1951 | 1356 | PSGSP | 1O3U:A | 89 | 93 |
| 1952 | 1357 | PSNSP | 2VPA:A | 100 | 104 |
| 1953 | 1358 | PTDTP | 3SF6:A | 203 | 207 |
| 1954 | 1359 | PTFTP | 1JFB:A | 103 | 107 |
| 1955 | 1360 | PTGGTP | 3AWU:A | 237 | 242 |
| 1956 | 1361 | PTGTP | 3M0Z:A | 119 | 123 |
| 1957 | 1362 | PTITP | 3SK2:A | 7 | 11 |
| 1958 |  | PTITP | 1KGD:A | 855 | 859 |
| 1959 | 1363 | PTNTP | 2CKW:A | 386 | 390 |
| 1960 | 1364 | PTPTP | 3DLQ:R | 141 | 145 |
| 1961 | 1365 | PVAVP | 1V8D:C | 184 | 188 |
| 1962 | 1366 | PVDVP | 3TX2:A | 220 | 224 |
| 1963 | 1367 | PVKVP | 2JE6:I | 161 | 165 |
| 1964 |  | PVKVP | 2NTX:A | 163 | 167 |
| 1965 | 1368 | PVNVP | 1T1D:A | 134 | 138 |
| 1966 | 1369 | PVSVP | 3TQE:A | 196 | 200 |
| 1967 | 1370 | PVTVP | 3DDC:B | 244 | 248 |
| 1968 | 1371 | PVVVP | 1M1F:B | 40 | 44 |
| 1969 | 1372 | PYFYP | 1ZMT:A | 184 | 188 |
| 1970 | 1373 | QAFAQ | 1PA2:A | 273 | 277 |
| 1971 |  | QAFAQ | 3H3H:A | 6 | 10 |
| 1972 | 1374 | QAGAQ | 3NVS:A | 135 | 139 |
| 1973 | 1375 | QAIAQ | 3MW6:B | 103 | 107 |
| 1974 | 1376 | QALAQ | 1QQF:A | 1277 | 1281 |
| 1975 | 1377 | QAQAQ | 1UI0:A | 7 | 11 |
| 1976 |  | QAQAQ | 1T0H:A | 47 | 51 |
| 1977 | 1378 | QATAQ | 2V9L:A | 195 | 199 |
| 1978 | 1379 | QCGGCQ | 3H31:A | 49 | 54 |
| 1979 | 1380 | QDFDQ | 1ZCB:A | 74 | 78 |
| 1980 | 1381 | QDVDQ | 1UNQ:A | 43 | 47 |
| 1981 | 1382 | QEGEQ | 3G3Z:A | 76 | 80 |
| 1982 | 1383 | QEIAAIEQ | 1BB1:C | 22 | 29 |
| 1983 | 1384 | QEKEQ | 3ZQO:J | 84 | 88 |
| 1984 | 1385 | QELLEQ | 1WU3:I | 18 | 23 |
| 1985 | 1386 | QFFFQ | 2Q52:A | 189 | 193 |
| 1986 | 1387 | QFVFQ | 3KEV:A | 102 | 106 |
| 1987 | 1388 | QGNGQ | 3U7I:D | 65 | 69 |
| 1988 | 1389 | QGTGQ | 3SE8:G | 428 | 432 |
| 1989 | 1390 | QIDIQ | 1U0A:D | 1005 | 1009 |
| 1990 | 1391 | QIFIQ | 1W98:B | 258 | 262 |
| 1991 | 1392 | QIKRKIQ | 2VUW:A | 769 | 775 |
| 1992 | 1393 | QITIQ | 2W4S:A | 464 | 468 |
| 1993 | 1394 | QIVIQ | 3DQG:A | 523 | 527 |
| 1994 | 1395 | QKAKQ | 1N62:E | 463 | 467 |
| 1995 | 1396 | QKLKQ | 3SWF:C | 35 | 39 |
| 1996 | 1397 | QKQKQ | 1NOW:A | 475 | 479 |
| 1997 | 1398 | QKVKQ | 2BO9:B | 35 | 39 |
| 1998 |  | QKVKQ | 3GE2:A | 106 | 110 |
| 1999 | 1399 | QLALQ | 3S0R:B | 18 | 22 |
| 2000 |  | QLALQ | 2Y1K:A | 172 | 176 |
| 2001 |  | QLALQ | 3QOU:A | 221 | 225 |
| 2002 | 1400 | QLDLQ | 1FG7:A | 134 | 138 |
| 2003 | 1401 | QLELQ | 3BFQ:G | 85 | 89 |
| 2004 |  | QLELQ | 2NXY:B | 1148 | 1152 |
| 2005 |  | QLELQ | 3AEI:B | 10 | 14 |
| 2006 | 1402 | QLFLQ | 2Y6U:A | 303 | 307 |
| 2007 | 1403 | QLILQ | 2P92:B | 80 | 84 |
| 2008 | 1404 | QLKLQ | 2V51:F | 75 | 79 |
| 2009 |  | QLKLQ | 2WM9:A | 336 | 340 |
| 2010 | 1405 | QLLLQ | 2HUJ:A | 9 | 13 |
| 2011 | 1406 | QLQLQ | 1WU3:I | 5 | 9 |
| 2012 | 1407 | QLQQLQ | 3US6:A | 29 | 34 |
| 2013 | 1408 | QLRDRLQ | 3CZ8:A | 228 | 234 |
| 2014 | 1409 | QLSLQ | 1S3J:B | 12 | 16 |
| 2015 |  | QLSLQ | 2IDO:D | 62 | 66 |
| 2016 | 1410 | QLVLQ | 1ORY:B | 2511 | 2515 |
| 2017 | 1411 | QMVMQ | 3BVU:A | 498 | 502 |
| 2018 | 1412 | QNENQ | 3F14:A | 67 | 71 |
| 2019 | 1413 | QPGPQ | 1H41:B | 294 | 298 |
| 2020 | 1414 | QPHPQ | 3M7P:A | 297 | 301 |
| 2021 | 1415 | QPSPQ | 2P0B:A | 59 | 63 |
| 2022 | 1416 | QPVPQ | 3H5Z:A | 18 | 22 |
| 2023 | 1417 | QQFQQ | 3RYD:A | 52 | 56 |
| 2024 | 1418 | QQIQQ | 2J9O:D | 22 | 26 |
| 2025 | 1419 | QQKQQ | 2EA7:A | 310 | 314 |
| 2026 | 1420 | QQLQQ | 3QOU:A | 203 | 207 |
| 2027 |  | QQLQQ | 4A56:A | 94 | 98 |
| 2028 | 1421 | QQVQQ | 1JM1:A | 65 | 69 |
| 2029 | 1422 | QRMRQ | 1TOJ:A | 331 | 335 |
| 2030 | 1423 | QRWRQ | 1VCL:A | 88 | 92 |
| 2031 | 1424 | QSESQ | 1P4X:A | 123 | 127 |
| 2032 | 1425 | QSLNLSQ | 1TXU:A | 372 | 378 |
| 2033 | 1426 | QSLSQ | 3B33:A | 47 | 51 |
| 2034 | 1427 | QSWSQ | 2XHG:A | 184 | 188 |
| 2035 | 1428 | QTITQ | 3LGI:C | 166 | 170 |
| 2036 |  | QTITQ | 1G8E:A | 73 | 77 |
| 2037 | 1429 | QTLGLTQ | 1OPD:A | 51 | 57 |
| 2038 | 1430 | QTPTQ | 2RE9:C | 33 | 37 |
| 2039 | 1431 | QTRIRTQ | 1B33:O | 13 | 19 |
| 2040 | 1432 | QVAVQ | 1SFS:A | 152 | 156 |
| 2041 | 1433 | QVCVQ | 2ACF:D | 323 | 327 |
| 2042 | 1434 | QVEVQ | 1CNU:A | 112 | 116 |
| 2043 |  | QVEVQ | 2OB5:A | 92 | 96 |
| 2044 | 1435 | QVFVQ | 2V5T:A | 297 | 301 |
| 2045 | 1436 | QVKVQ | 1KJQ:B | 387 | 391 |
| 2046 | 1437 | QVLVQ | 1K4Z:A | 1408 | 1412 |
| 2047 |  | QVLVQ | 1YA5:T | 58 | 62 |
| 2048 |  | QVLVQ | 3GJU:A | 309 | 313 |
| 2049 | 1438 | QVRVQ | 3O12:A | 65 | 69 |
| 2050 | 1439 | QVTVQ | 2P02:A | 205 | 209 |
| 2051 | 1440 | QYGYQ | 1QWY:A | 131 | 135 |
| 2052 | 1441 | QYRGRYQ | 2YH6:A | 132 | 138 |
| 2053 | 1442 | QYVYQ | 1QSA:A | 535 | 539 |
| 2054 | 1443 | RAAAR | 2YB1:A | 25 | 29 |
| 2055 |  | RAAAR | 3AWU:B | 105 | 109 |
| 2056 |  | RAAAR | 1U69:D | 132 | 136 |
| 2057 | 1444 | RADAR | 3MXO:A | 214 | 218 |
| 2058 | 1445 | RAGAR | 3H4T:A | 58 | 62 |
| 2059 | 1446 | RAIAR | 3CBN:A | 77 | 81 |
| 2060 | 1447 | RAKAR | 1N62:F | 281 | 285 |
| 2061 | 1448 | RALAR | 3E1I:A | 167 | 171 |
| 2062 |  | RALAR | 2QSA:A | 58 | 62 |
| 2063 |  | RALAR | 3L77:A | 19 | 23 |
| 2064 |  | RALAR | 1DM9:B | 24 | 28 |
| 2065 |  | RALAR | 1O9G:A | 42 | 46 |
| 2066 | 1449 | RASAR | 3HIM:A | 108 | 112 |
| 2067 | 1450 | RAVAR | 1DCS:A | 131 | 135 |
| 2068 |  | RAVAR | 2FA5:B | 82 | 86 |
| 2069 |  | RAVAR | 3G7R:B | 92 | 96 |
| 2070 | 1451 | RCPCR | 3GV3:A | 8 | 12 |
| 2071 | 1452 | RCQCR | 1P9G:A | 36 | 40 |
| 2072 | 1453 | RDADR | 2QGS:A | 120 | 124 |
| 2073 | 1454 | RDEDR | 1EYH:A | 135 | 139 |
| 2074 |  | RDEDR | 1MZG:B | 38 | 42 |
| 2075 | 1455 | RDFDR | 3GDC:C | 29 | 33 |
| 2076 | 1456 | RDPDR | 3ZW5:B | 133 | 137 |
| 2077 | 1457 | REAER | 1T0H:A | 42 | 46 |
| 2078 | 1458 | REFER | 1MKY:A | 124 | 128 |
| 2079 |  | REFER | 2V6V:B | 406 | 410 |
| 2080 | 1459 | REHER | 3QA9:A | 132 | 136 |
| 2081 | 1460 | REIER | 1V4P:A | 56 | 60 |
| 2082 |  | REIER | 2HHG:A | 48 | 52 |
| 2083 | 1461 | REKER | 1XJU:A | 171 | 175 |
| 2084 | 1462 | RELER | 1O9G:A | 108 | 112 |
| 2085 |  | RELER | 3VAA:B | 57 | 61 |
| 2086 | 1463 | RELLER | 3NJA:A | 91 | 96 |
| 2087 | 1464 | RENER | 2JK1:A | 128 | 132 |
| 2088 | 1465 | REPER | 3B7F:A | 64 | 68 |
| 2089 | 1466 | REQER | 2HFE:D | 117 | 121 |
| 2090 | 1467 | REQQER | 1ZL0:A | 149 | 154 |
| 2091 | 1468 | REVVER | 2XF3:A | 248 | 253 |
| 2092 | 1469 | RFAFR | 1YFQ:A | 235 | 239 |
| 2093 | 1470 | RFNFR | 3D7R:B | 68 | 72 |
| 2094 |  | RFNFR | 2I3D:A | 62 | 66 |
| 2095 | 1471 | RGAGAGR | 2JJU:A | 40 | 46 |
| 2096 | 1472 | RGFGR | 4AFF:A | 34 | 38 |
| 2097 | 1473 | RGHGR | 4DGQ:C | 58 | 62 |
| 2098 | 1474 | RGIGR | 3PWT:A | 489 | 493 |
| 2099 |  | RGIGR | 3KZ9:D | 32 | 36 |
| 2100 | 1475 | RGLLGR | 1Q33:A | 159 | 164 |
| 2101 | 1476 | RGNGR | 1TU7:A | 135 | 139 |
| 2102 | 1477 | RGPGR | 3OJN:D | 243 | 247 |
| 2103 | 1478 | RGQGR | 3SX2:H | 21 | 25 |
| 2104 | 1479 | RGRGR | 2J45:B | 17 | 21 |
| 2105 | 1480 | RHIDIHR | 2VLQ:B | 126 | 132 |
| 2106 | 1481 | RIAIR | 2FA8:A | 7 | 11 |
| 2107 | 1482 | RIFIR | 1X6I:B | 241 | 245 |
| 2108 | 1483 | RIGIR | 1VYR:A | 229 | 233 |
| 2109 | 1484 | RILIR | 3KAN:C | 94 | 98 |
| 2110 |  | RILIR | 1LC5:A | 319 | 323 |
| 2111 | 1485 | RIRIR | 3D3B:J | 5 | 9 |
| 2112 | 1486 | RKIKR | 3V3L:A | 163 | 167 |
| 2113 | 1487 | RKLKR | 4ADU:B | 203 | 207 |
| 2114 | 1488 | RKTKR | 3IMH:A | 218 | 222 |
| 2115 | 1489 | RLAQALR | 3O7I:B | 97 | 103 |
| 2116 | 1490 | RLELR | 2FFU:A | 414 | 418 |
| 2117 | 1491 | RLGLR | 1U83:A | 242 | 246 |
| 2118 |  | RLGLR | 1M56:C | 76 | 80 |
| 2119 | 1492 | RLILILR | 3LRU:A | 1935 | 1941 |
| 2120 | 1493 | RLNLR | 1R29:A | 94 | 98 |
| 2121 | 1494 | RLPPLR | 3DTZ:B | 113 | 118 |
| 2122 | 1495 | RLRLR | 1NZ0:A | 10 | 14 |
| 2123 |  | RLRLR | 1PRZ:A | 223 | 227 |
| 2124 |  | RLRLR | 2ILK:A | 102 | 106 |
| 2125 | 1496 | RLTLR | 1KYF:A | 916 | 920 |
| 2126 | 1497 | RLTTLR | 1LYV:A | 200 | 205 |
| 2127 | 1498 | RLVLR | 2VYN:D | 13 | 17 |
| 2128 | 1499 | RLYLR | 3V9O:A | 20 | 24 |
| 2129 | 1500 | RMMMR | 2C5S:A | 266 | 270 |
| 2130 | 1501 | RNPNR | 1YKS:A | 467 | 471 |
| 2131 | 1502 | RPGPR | 2XZZ:A | 760 | 764 |
| 2132 | 1503 | RPLPR | 1B3A:A | 17 | 21 |
| 2133 | 1504 | RQCQR | 3IOL:A | 44 | 48 |
| 2134 | 1505 | RQDQR | 1MKY:A | 279 | 283 |
| 2135 | 1506 | RQHQR | 3LRT:A | 91 | 95 |
| 2136 | 1507 | RQIQR | 2I7H:D | 102 | 106 |
| 2137 |  | RQIQR | 1R4P:A | 172 | 176 |
| 2138 | 1508 | RQKQR | 1WKO:A | 129 | 133 |
| 2139 | 1509 | RQLQR | 3M9Q:A | 80 | 84 |
| 2140 | 1510 | RQVQR | 3FOT:A | 27 | 31 |
| 2141 |  | RQVQR | 1B1U:A | 87 | 91 |
| 2142 | 1511 | RRCRR | 2POC:D | 393 | 397 |
| 2143 | 1512 | RRIIRR | 3HXW:A | 310 | 315 |
| 2144 | 1513 | RRLLRR | 3YGS:P | 7 | 12 |
| 2145 | 1514 | RRLRR | 3OQ2:A | 24 | 28 |
| 2146 | 1515 | RRMRR | 3EVF:A | 242 | 246 |
| 2147 | 1516 | RRNRR | 1W5Q:A | 16 | 20 |
| 2148 |  | RRNRR | 3LPH:D | 38 | 42 |
| 2149 | 1517 | RRSRR | 1Z9W:A | 116 | 120 |
| 2150 | 1518 | RSDSR | 3FOT:A | 211 | 215 |
| 2151 | 1519 | RSFSR | 2FZV:A | 47 | 51 |
| 2152 | 1520 | RSGSR | 2JC9:A | 442 | 446 |
| 2153 | 1521 | RSISR | 1N7O:A | 462 | 466 |
| 2154 | 1522 | RSLSR | 3GSZ:B | 109 | 113 |
| 2155 | 1523 | RSMSR | 3CZ8:A | 378 | 382 |
| 2156 | 1524 | RSWSR | 3GMO:A | 21 | 25 |
| 2157 |  | RSWSR | 1T0I:B | 76 | 80 |
| 2158 | 1525 | RTATR | 2Y6U:A | 48 | 52 |
| 2159 | 1526 | RTITR | 3CI3:A | 128 | 132 |
| 2160 | 1527 | RTVTR | 1O4Y:A | 225 | 229 |
| 2161 | 1528 | RVADAVR | 3CWR:A | 193 | 199 |
| 2162 | 1529 | RVAVR | 1IS1:A | 125 | 129 |
| 2163 | 1530 | RVDVR | 2FP7:A | 74 | 78 |
| 2164 | 1531 | RVEVR | 3CXK:B | 99 | 103 |
| 2165 | 1532 | RVFFVR | 3H8T:A | 178 | 183 |
| 2166 | 1533 | RVGGGVR | 2VFR:A | 80 | 86 |
| 2167 | 1534 | RVLVR | 3SNK:A | 102 | 106 |
| 2168 | 1535 | RVMVR | 3E19:C | 71 | 75 |
| 2169 | 1536 | RVSVR | 2X49:A | 540 | 544 |
| 2170 | 1537 | RVTVR | 2XF3:A | 220 | 224 |
| 2171 | 1538 | RWFWR | 3C7X:A | 339 | 343 |
| 2172 | 1539 | RWYWR | 3CI0:I | 73 | 77 |
| 2173 | 1540 | RYVEEVYR | 1UFO:D | 76 | 83 |
| 2174 | 1541 | SAAGAAS | 1NWW:A | 15 | 21 |
| 2175 | 1542 | SAEAS | 2ZXK:A | 99 | 103 |
| 2176 | 1543 | SAGAS | 4DNU:A | 21 | 25 |
| 2177 |  | SAGAS | 1WW7:A | 12 | 16 |
| 2178 |  | SAGAS | 2Q4H:B | 177 | 181 |
| 2179 | 1544 | SAGQGAS | 1I1W:A | 211 | 217 |
| 2180 | 1545 | SAIAS | 2P8I:A | 7 | 11 |
| 2181 | 1546 | SAKAS | 2VUV:A | 18 | 22 |
| 2182 | 1547 | SALAS | 2Q0S:A | 169 | 173 |
| 2183 |  | SALAS | 1QOP:B | 119 | 123 |
| 2184 |  | SALAS | 1X6Z:A | 35 | 39 |
| 2185 | 1548 | SAQAS | 2WJ5:A | 89 | 93 |
| 2186 | 1549 | SASAS | 3AKH:A | 226 | 230 |
| 2187 | 1550 | SAVAS | 1KNQ:B | 22 | 26 |
| 2188 | 1551 | SAVVAS | 1NLS:A | 185 | 190 |
| 2189 | 1552 | SAYAS | 2E4T:A | 433 | 437 |
| 2190 |  | SAYAS | 3V5U:A | 58 | 62 |
| 2191 | 1553 | SCCCS | 1UZK:A | 1561 | 1565 |
| 2192 | 1554 | SCECS | 1EAI:D | 37 | 41 |
| 2193 | 1555 | SDADS | 3R24:A | 105 | 109 |
| 2194 | 1556 | SDEDS | 3LPH:D | 8 | 12 |
| 2195 | 1557 | SDFDS | 3KP7:B | 123 | 127 |
| 2196 | 1558 | SDIDS | 3FCD:A | 73 | 77 |
| 2197 |  | SDIDS | 2XDJ:F | 34 | 38 |
| 2198 | 1559 | SDNDS | 3N1F:C | 871 | 875 |
| 2199 |  | SDNDS | 2XE5:A | 24 | 28 |
| 2200 | 1560 | SDPDS | 2FZV:A | 7 | 11 |
| 2201 | 1561 | SDTDS | 3AWU:A | 44 | 48 |
| 2202 | 1562 | SEIES | 2F5V:A | 293 | 297 |
| 2203 | 1563 | SEKES | 2Q22:C | 33 | 37 |
| 2204 | 1564 | SENES | 1TH8:A | 13 | 17 |
| 2205 | 1565 | SEQES | 2BNL:F | 30 | 34 |
| 2206 | 1566 | SETES | 1R8N:A | 181 | 185 |
| 2207 |  | SETES | 1K4Z:A | 1423 | 1427 |
| 2208 | 1567 | SEVES | 1TY0:B | 54 | 58 |
| 2209 | 1568 | SFCFS | 1Z7C:A | 51 | 55 |
| 2210 | 1569 | SFEFS | 2ZKM:X | 556 | 560 |
| 2211 | 1570 | SFGFS | 3ZWF:A | 186 | 190 |
| 2212 | 1571 | SFIFS | 1I4U:A | 133 | 137 |
| 2213 | 1572 | SFLFS | 1GO4:G | 490 | 494 |
| 2214 |  | SFLFS | 3KAE:A | 144 | 148 |
| 2215 | 1573 | SFPFS | 1JFB:A | 7 | 11 |
| 2216 | 1574 | SFRFS | 3FAU:A | 74 | 78 |
| 2217 | 1575 | SFTRTFS | 2G6Y:B | 153 | 159 |
| 2218 | 1576 | SGAGS | 1GCI:A | 156 | 164 |
| 2219 | 1577 | SGDGS | 1BXO:A | 277 | 281 |
| 2220 |  | SGDGS | 3U7Q:A | 254 | 258 |
| 2221 | 1578 | SGEGS | 3F2Z:A | 310 | 314 |
| 2222 | 1579 | SGLGS | 1H72:C | 93 | 97 |
| 2223 | 1580 | SGNGS | 3H09:B | 901 | 905 |
| 2224 | 1581 | SGQGS | 1QLW:B | 16 | 20 |
| 2225 | 1582 | SGSGS | 3S6L:D | 70 | 74 |
| 2226 |  | SGSGS | 1MJU:L | 63 | 67 |
| 2227 |  | SGSGS | 1GCI:A | 99 | 103 |
| 2228 |  | SGSGS | 3MDY:A | 186 | 190 |
| 2229 | 1583 | SGSMSGS | 1QCX:A | 319 | 325 |
| 2230 | 1584 | SGTGS | 2OPI:A | 41 | 45 |
| 2231 |  | SGTGS | 3DA8:B | 20 | 24 |
| 2232 | 1585 | SGVGVGS | 3JU4:A | 700 | 706 |
| 2233 | 1586 | SHPHS | 1GKM:A | 399 | 403 |
| 2234 | 1587 | SHSHS | 2Q4H:B | 183 | 187 |
| 2235 | 1588 | SIAIS | 2END:A | 110 | 114 |
| 2236 | 1589 | SICIS | 2FM8:A | 42 | 46 |
| 2237 | 1590 | SIDIDIS | 1CR5:A | 116 | 122 |
| 2238 | 1591 | SIEEIS | 3A35:B | 146 | 151 |
| 2239 | 1592 | SIEIS | 3PXL:A | 379 | 383 |
| 2240 | 1593 | SIGLGIS | 2A5Z:C | 142 | 148 |
| 2241 | 1594 | SIMMIS | 1KZL:A | 158 | 163 |
| 2242 | 1595 | SINIS | 3QJG:L | 15 | 19 |
| 2243 |  | SINIS | 3SG8:A | 132 | 136 |
| 2244 | 1596 | SISIS | 1Z9M:B | 104 | 108 |
| 2245 |  | SISIS | 3MOL:B | 2 | 6 |
| 2246 |  | SISIS | 2JJU:A | 75 | 79 |
| 2247 | 1597 | SIYIS | 2Q3E:A | 350 | 354 |
| 2248 | 1598 | SKAKS | 3Q6X:B | 213 | 217 |
| 2249 | 1599 | SKCKS | 1U7L:A | 338 | 342 |
| 2250 | 1600 | SKEKS | 2I3D:A | 20 | 24 |
| 2251 | 1601 | SKGKS | 2C0H:A | 55 | 59 |
| 2252 | 1602 | SKIIKS | 2VDU:D | 311 | 316 |
| 2253 | 1603 | SKLKS | 1NLS:A | 113 | 117 |
| 2254 | 1604 | SLALS | 3NSU:A | 514 | 518 |
| 2255 | 1605 | SLDLS | 2Z80:A | 56 | 60 |
| 2256 | 1606 | SLFLS | 3ENU:A | 63 | 67 |
| 2257 |  | SLFLS | 3Q7R:B | 105 | 109 |
| 2258 | 1607 | SLGLS | 2O71:A | 137 | 141 |
| 2259 |  | SLGLS | 2Q3G:A | 78 | 82 |
| 2260 | 1608 | SLKLS | 2VFO:A | 124 | 128 |
| 2261 | 1609 | SLLLS | 2OXC:A | 66 | 70 |
| 2262 |  | SLLLS | 3BQA:A | 130 | 134 |
| 2263 |  | SLLLS | 1C4Q:B | 238 | 242 |
| 2264 | 1610 | SLLMLLS | 1UOC:A | 251 | 257 |
| 2265 | 1611 | SLNLS | 1TXU:A | 373 | 377 |
| 2266 | 1612 | SLPLS | 1OW1:A | 3537 | 3541 |
| 2267 |  | SLPLS | 3CEG:A | 4560 | 4564 |
| 2268 | 1613 | SLQQLS | 1O6V:B | 187 | 192 |
| 2269 | 1614 | SLRLS | 3QXV:E | 19 | 23 |
| 2270 | 1615 | SLSLS | 3D9S:D | 229 | 233 |
| 2271 |  | SLSLS | 2H88:C | 76 | 80 |
| 2272 | 1616 | SLTLS | 2JKS:A | 32 | 36 |
| 2273 |  | SLTLS | 2OCT:A | 79 | 83 |
| 2274 |  | SLTLS | 3LQ9:A | 207 | 211 |
| 2275 | 1617 | SLVLS | 1GKM:A | 78 | 82 |
| 2276 | 1618 | SMWMS | 1H8P:A | 88 | 92 |
| 2277 | 1619 | SNDNS | 1PK3:C | 28 | 32 |
| 2278 | 1620 | SNENS | 2OD4:A | 51 | 55 |
| 2279 | 1621 | SNQNS | 3O79:A | 170 | 174 |
| 2280 |  | SNQNS | 2A6Z:A | 48 | 52 |
| 2281 | 1622 | SNTNS | 2W91:A | 230 | 234 |
| 2282 | 1623 | SNVNS | 3C8G:D | 117 | 121 |
| 2283 | 1624 | SNYNS | 1U4G:A | 263 | 267 |
| 2284 | 1625 | SPCPS | 1GP0:A | 1596 | 1600 |
| 2285 | 1626 | SPIPS | 3BY9:A | 234 | 238 |
| 2286 | 1627 | SPLLPS | 2XR6:A | 333 | 338 |
| 2287 | 1628 | SPLPS | 2RHK:A | 161 | 165 |
| 2288 | 1629 | SPSPS | 1GCI:A | 128 | 132 |
| 2289 | 1630 | SQTQS | 1ARB:A | 97 | 101 |
| 2290 | 1631 | SQWQS | 2V3G:A | 195 | 199 |
| 2291 | 1632 | SRARS | 1EJ0:A | 193 | 197 |
| 2292 | 1633 | SRDRS | 2P26:A | 431 | 435 |
| 2293 | 1634 | SRGRS | 1N7O:A | 459 | 463 |
| 2294 | 1635 | SSAASS | 1H72:C | 97 | 102 |
| 2295 |  | SSAASS | 2CXY:A | 82 | 87 |
| 2296 | 1636 | SSASS | 3ED1:C | 127 | 131 |
| 2297 | 1637 | SSCSS | 1BGC:A | 63 | 67 |
| 2298 | 1638 | SSDSS | 3K94:A | 209 | 213 |
| 2299 | 1639 | SSFSS | 3DTZ:B | 88 | 92 |
| 2300 | 1640 | SSGSS | 3NDI:A | 80 | 84 |
| 2301 |  | SSGSS | 3TU8:A | 193 | 197 |
| 2302 | 1641 | SSHSS | 3BVU:A | 1041 | 1045 |
| 2303 | 1642 | SSISS | 1N7O:A | 214 | 218 |
| 2304 | 1643 | SSKSS | 3I26:B | 61 | 65 |
| 2305 | 1644 | SSLSS | 1PQ5:A | 82 | 86 |
| 2306 |  | SSLSS | 3IJW:B | 36 | 40 |
| 2307 | 1645 | SSVSS | 1WKO:A | 49 | 53 |
| 2308 |  | SSVSS | 3FM8:B | 518 | 522 |
| 2309 | 1646 | SSYYSS | 1Z4V:A | 346 | 351 |
| 2310 | 1647 | STCTS | 2QJV:B | 6 | 10 |
| 2311 | 1648 | STETS | 1QHV:A | 542 | 546 |
| 2312 | 1649 | STFTS | 3CDX:D | 196 | 200 |
| 2313 | 1650 | STGTS | 3TMP:E | 302 | 306 |
| 2314 | 1651 | STITS | 3AJI:D | 346 | 350 |
| 2315 |  | STITS | 1H16:A | 79 | 83 |
| 2316 | 1652 | STKTS | 3LR2:B | 91 | 95 |
| 2317 | 1653 | STPTS | 2BFF:A | 227 | 231 |
| 2318 | 1654 | STQTS | 3AJ6:B | 127 | 131 |
| 2319 | 1655 | STVTS | 3MQD:A | 284 | 288 |
| 2320 | 1656 | STYTS | 1ZMT:A | 143 | 147 |
| 2321 | 1657 | SVDDVS | 1KW4:A | 18 | 23 |
| 2322 | 1658 | SVEVS | 3AMN:B | 96 | 100 |
| 2323 |  | SVEVS | 2ZFI:A | 141 | 145 |
| 2324 | 1659 | SVHVS | 2D0B:A | 233 | 237 |
| 2325 | 1660 | SVIVS | 3K4I:A | 85 | 89 |
| 2326 | 1661 | SVKVS | 1LKE:A | 53 | 57 |
| 2327 | 1662 | SVMVS | 2PTZ:A | 366 | 370 |
| 2328 | 1663 | SVPVS | 1URS:A | 135 | 139 |
| 2329 |  | SVPVS | 3HPW:A | 70 | 74 |
| 2330 |  | SVPVS | 2GVG:A | 271 | 275 |
| 2331 | 1664 | SVQVS | 3AA0:A | 215 | 219 |
| 2332 | 1665 | SVSSVS | 1MG7:B | 338 | 343 |
| 2333 | 1666 | SVSVS | 2Y7L:A | 99 | 103 |
| 2334 |  | SVSVS | 2VBK:A | 328 | 332 |
| 2335 |  | SVSVS | 3OV5:A | 118 | 122 |
| 2336 | 1667 | SVTVS | 2EEY:A | 33 | 37 |
| 2337 |  | SVTVS | 1XOD:A | 38 | 42 |
| 2338 | 1668 | SVYVS | 3PJ0:D | 200 | 204 |
| 2339 |  | SVYVS | 1Y7R:A | 99 | 103 |
| 2340 | 1669 | SYEYS | 3QRA:A | 160 | 164 |
| 2341 | 1670 | SYFYS | 3LL8:A | 257 | 261 |
| 2342 |  | SYFYS | 3FVV:A | 181 | 185 |
| 2343 |  | SYFYS | 1PP0:A | 148 | 152 |
| 2344 | 1671 | SYKYS | 2P5K:A | 59 | 63 |
| 2345 | 1672 | SYTTYS | 3DTZ:B | 161 | 166 |
| 2346 | 1673 | SYTYS | 2ZUX:B | 146 | 150 |
| 2347 |  | SYTYS | 3KA8:A | 27 | 31 |
| 2348 | 1674 | SYYYS | 1YKS:A | 475 | 479 |
| 2349 |  | SYYYS | 2NPT:A | 83 | 87 |
| 2350 | 1675 | TAAAAT | 2DWU:C | 79 | 84 |
| 2351 | 1676 | TAAAT | 1L3P:A | 170 | 174 |
| 2352 | 1677 | TACAT | 3NO8:B | 466 | 470 |
| 2353 | 1678 | TADAT | 1OQV:A | 60 | 64 |
| 2354 | 1679 | TAFAT | 1HBN:A | 413 | 417 |
| 2355 |  | TAFAT | 2Q37:A | 83 | 87 |
| 2356 | 1680 | TAKAT | 3CLM:A | 298 | 302 |
| 2357 |  | TAKAT | 3KDF:C | 74 | 78 |
| 2358 | 1681 | TALAT | 2O7T:A | 162 | 166 |
| 2359 | 1682 | TASAT | 3MQD:A | 139 | 143 |
| 2360 | 1683 | TATAT | 1N2F:A | 9 | 13 |
| 2361 |  | TATAT | 1N2F:A | 9 | 13 |
| 2362 | 1684 | TATATAT | 1N2F:A | 9 | 15 |
| 2363 | 1685 | TDADT | 3KV1:A | 197 | 201 |
| 2364 | 1686 | TDDDT | 1R7A:B | 444 | 448 |
| 2365 | 1687 | TDLDT | 2Z80:A | 232 | 236 |
| 2366 | 1688 | TDNDT | 2RA2:F | 24 | 28 |
| 2367 |  | TDNDT | 2NZC:A | 55 | 59 |
| 2368 | 1689 | TDPDT | 7ODC:A | 203 | 207 |
| 2369 | 1690 | TDVDT | 1PBJ:A | 9 | 13 |
| 2370 | 1691 | TEDDET | 1QIP:D | 106 | 111 |
| 2371 | 1692 | TEDET | 3H5Z:A | 23 | 27 |
| 2372 | 1693 | TEEET | 1SQW:A | 5 | 9 |
| 2373 | 1694 | TEKET | 2D48:A | 40 | 44 |
| 2374 | 1695 | TELET | 2VZC:A | 284 | 288 |
| 2375 |  | TELET | 1JS3:B | 112 | 116 |
| 2376 | 1696 | TENET | 1Z9T:A | 219 | 223 |
| 2377 | 1697 | TETET | 2FAU:A | 225 | 229 |
| 2378 | 1698 | TEVET | 3ARC:u | 85 | 89 |
| 2379 | 1699 | TFDFT | 1QCX:A | 48 | 52 |
| 2380 | 1700 | TFEFT | 2Y6H:A | 129 | 133 |
| 2381 | 1701 | TFGFT | 1GK9:A | 121 | 125 |
| 2382 |  | TFGFT | 1Y55:X | 61 | 65 |
| 2383 |  | TFGFT | 1I5N:B | 56 | 60 |
| 2384 | 1702 | TFLFT | 4ACJ:A | 722 | 726 |
| 2385 |  | TFLFT | 2P02:A | 39 | 43 |
| 2386 | 1703 | TFQFT | 2XXN:A | 70 | 74 |
| 2387 | 1704 | TFSFT | 2VYO:A | 56 | 60 |
| 2388 |  | TFSFT | 3F02:A | 201 | 205 |
| 2389 | 1705 | TGAAGT | 3FYQ:A | 2044 | 2049 |
| 2390 | 1706 | TGAGT | 3LYE:A | 113 | 117 |
| 2391 | 1707 | TGDGT | 1QAU:A | 112 | 116 |
| 2392 | 1708 | TGEGT | 2PW8:I | 41 | 45 |
| 2393 |  | TGEGT | 2D42:B | 224 | 228 |
| 2394 | 1709 | TGFGT | 1YPF:A | 181 | 185 |
| 2395 | 1710 | TGGGT | 3TX2:A | 42 | 46 |
| 2396 | 1711 | TGSGT | 2CHH:A | 41 | 45 |
| 2397 | 1712 | TGTGT | 3FMF:D | 7 | 11 |
| 2398 |  | TGTGT | 3KCU:C | 57 | 61 |
| 2399 | 1713 | TGTTGT | 3GE2:A | 91 | 96 |
| 2400 | 1714 | TGVGT | 2RK5:A | 45 | 49 |
| 2401 | 1715 | TIDIT | 1PBJ:A | 13 | 17 |
| 2402 | 1716 | TIEEIT | 3KLQ:B | 36 | 41 |
| 2403 | 1717 | TIHIT | 3EPB:A | 241 | 245 |
| 2404 | 1718 | TILIT | 1BJA:A | 21 | 25 |
| 2405 |  | TILIT | 2GN4:A | 12 | 16 |
| 2406 |  | TILIT | 1PP0:A | 156 | 160 |
| 2407 | 1719 | TIQIT | 2WQR:A | 263 | 267 |
| 2408 |  | TIQIT | 3QY9:D | 203 | 207 |
| 2409 | 1720 | TIRIT | 3D33:A | 78 | 82 |
| 2410 | 1721 | TIVIT | 3PG6:D | 632 | 636 |
| 2411 | 1722 | TIYIT | 3L8W:A | 63 | 67 |
| 2412 | 1723 | TKIKT | 1YPF:A | 177 | 181 |
| 2413 | 1724 | TKLKT | 3LED:B | 225 | 229 |
| 2414 | 1725 | TKNKT | 1G8M:A | 406 | 410 |
| 2415 | 1726 | TKRKT | 3DK9:A | 411 | 415 |
| 2416 | 1727 | TKSKT | 1KL9:A | 102 | 106 |
| 2417 | 1728 | TKVKT | 2GNP:A | 234 | 238 |
| 2418 | 1729 | TLAALT | 1G8E:A | 45 | 50 |
| 2419 | 1730 | TLALT | 1JY5:A | 9 | 13 |
| 2420 | 1731 | TLCLT | 3KT7:A | 537 | 541 |
| 2421 | 1732 | TLDLT | 3QAO:A | 98 | 102 |
| 2422 | 1733 | TLELT | 1QIP:D | 97 | 101 |
| 2423 | 1734 | TLGLT | 1OPD:A | 52 | 56 |
| 2424 | 1735 | TLILT | 3LMB:A | 39 | 43 |
| 2425 | 1736 | TLKLT | 3RYC:B | 216 | 220 |
| 2426 | 1737 | TLLLT | 1IAR:B | 113 | 117 |
| 2427 | 1738 | TLRLT | 1XT5:A | 93 | 97 |
| 2428 |  | TLRLT | 3BZN:A | 122 | 126 |
| 2429 |  | TLRLT | 2FQM:A | 121 | 125 |
| 2430 | 1739 | TLTLT | 1QVE:A | 112 | 116 |
| 2431 |  | TLTLT | 1MJU:L | 178 | 182 |
| 2432 |  | TLTLT | 3P2U:B | 4 | 8 |
| 2433 | 1740 | TLVLT | 3PL0:A | 239 | 243 |
| 2434 |  | TLVLT | 2IQY:A | 65 | 69 |
| 2435 |  | TLVLT | 3GIY:A | 152 | 156 |
| 2436 |  | TLVLT | 1QHV:A | 422 | 426 |
| 2437 | 1741 | TLYLT | 3HUL:B | 24 | 28 |
| 2438 | 1742 | TMNMT | 1Y42:X | 160 | 164 |
| 2439 | 1743 | TNCNT | 1RC9:A | 190 | 194 |
| 2440 | 1744 | TNDNT | 3D2Q:D | 213 | 217 |
| 2441 | 1745 | TNINT | 3D59:A | 420 | 424 |
| 2442 | 1746 | TNQNT | 3U02:C | 246 | 250 |
| 2443 | 1747 | TNRNT | 2VB1:A | 43 | 47 |
| 2444 | 1748 | TNSSNT | 3CFU:A | 193 | 198 |
| 2445 | 1749 | TNTNT | 3CKM:A | 471 | 475 |
| 2446 | 1750 | TPDPT | 1ALU:A | 138 | 142 |
| 2447 | 1751 | TPGPT | 3C8Z:B | 33 | 37 |
| 2448 | 1752 | TPNNPT | 1LC5:A | 154 | 159 |
| 2449 | 1753 | TPNPT | 1QLW:B | 182 | 186 |
| 2450 | 1754 | TPTPT | 3Q63:A | 98 | 102 |
| 2451 | 1755 | TPWPT | 1URS:A | 270 | 274 |
| 2452 | 1756 | TQAQT | 1B8O:A | 43 | 47 |
| 2453 | 1757 | TQEQT | 3UO3:B | 89 | 93 |
| 2454 | 1758 | TQPQT | 1GXM:B | 545 | 549 |
| 2455 |  | TQPQT | 2BS2:F | 149 | 153 |
| 2456 | 1759 | TQPVPQT | 3H5Z:A | 17 | 23 |
| 2457 | 1760 | TQSQT | 1QHV:A | 507 | 511 |
| 2458 | 1761 | TQVQT | 2FB6:A | 55 | 59 |
| 2459 | 1762 | TRERT | 3O0A:B | 319 | 323 |
| 2460 | 1763 | TRIRT | 1B33:O | 14 | 18 |
| 2461 | 1764 | TRSRT | 3RL5:A | 66 | 70 |
| 2462 |  | TRSRT | 3D3B:J | 44 | 48 |
| 2463 |  | TRSRT | 3THR:D | 37 | 41 |
| 2464 | 1765 | TSAST | 2OEG:A | 135 | 139 |
| 2465 | 1766 | TSCST | 1K4Z:A | 1481 | 1485 |
| 2466 | 1767 | TSDST | 3H09:B | 421 | 425 |
| 2467 |  | TSDST | 1X9D:A | 390 | 394 |
| 2468 |  | TSDST | 3D6M:A | 226 | 230 |
| 2469 | 1768 | TSEST | 1QHV:A | 539 | 543 |
| 2470 | 1769 | TSGGST | 1Y43:B | 135 | 140 |
| 2471 |  | TSGGST | 1EB6:A | 69 | 74 |
| 2472 | 1770 | TSGST | 2X49:A | 608 | 612 |
| 2473 | 1771 | TSKST | 1CXQ:A | 127 | 131 |
| 2474 | 1772 | TSLLST | 3EQA:A | 96 | 101 |
| 2475 | 1773 | TSPST | 3HTK:C | 52 | 56 |
| 2476 | 1774 | TSQST | 2FLH:B | 135 | 139 |
| 2477 | 1775 | TSSST | 1GL4:A | 552 | 556 |
| 2478 |  | TSSST | 1MJU:H | 73 | 77 |
| 2479 |  | TSSST | 1TY0:B | 178 | 182 |
| 2480 | 1776 | TTGGTT | 2CWS:A | 134 | 139 |
| 2481 | 1777 | TTLVLTT | 3GIY:A | 151 | 157 |
| 2482 | 1778 | TTPDPTT | 1ALU:A | 137 | 143 |
| 2483 | 1779 | TTVTVTT | 1QFO:C | 112 | 118 |
| 2484 | 1780 | TVCVT | 2C29:F | 7 | 11 |
| 2485 | 1781 | TVDVT | 2CAK:A | 71 | 75 |
| 2486 |  | TVDVT | 2HWV:A | 196 | 200 |
| 2487 | 1782 | TVEVEVT | 2PTZ:A | 19 | 25 |
| 2488 | 1783 | TVEVT | 2JIL:A | 150 | 154 |
| 2489 | 1784 | TVGGVT | 1BXO:A | 91 | 96 |
| 2490 | 1785 | TVGVT | 1U4G:A | 292 | 296 |
| 2491 | 1786 | TVIVT | 1STM:A | 140 | 144 |
| 2492 | 1787 | TVKVT | 2X9Z:A | 253 | 257 |
| 2493 |  | TVKVT | 2QIY:B | 56 | 60 |
| 2494 |  | TVKVT | 3LPW:B | 154 | 158 |
| 2495 | 1788 | TVLVT | 2JKS:A | 302 | 306 |
| 2496 |  | TVLVT | 2OKG:B | 324 | 328 |
| 2497 | 1789 | TVPVT | 2VN5:A | 29 | 33 |
| 2498 |  | TVPVT | 3R9Z:A | 184 | 188 |
| 2499 |  | TVPVT | 2WY3:D | 118 | 122 |
| 2500 | 1790 | TVRRVT | 3LLT:A | 696 | 701 |
| 2501 | 1791 | TVRVT | 2VZP:B | 97 | 101 |
| 2502 |  | TVRVT | 3C4S:B | 7 | 11 |
| 2503 | 1792 | TVSVT | 2BSY:A | 149 | 153 |
| 2504 |  | TVSVT | 1TT8:A | 44 | 48 |
| 2505 |  | TVSVT | 1ZGZ:A | 28 | 32 |
| 2506 | 1793 | TVTVT | 3KUV:B | 85 | 89 |
| 2507 |  | TVTVT | 1QFO:C | 113 | 117 |
| 2508 |  | TVTVT | 1K3I:A | 218 | 222 |
| 2509 |  | TVTVT | 2OSX:A | 483 | 487 |
| 2510 | 1794 | TWLWT | 3F9S:A | 86 | 90 |
| 2511 | 1795 | TYGYT | 2RG9:B | 67 | 71 |
| 2512 | 1796 | TYTYT | 2Y6H:A | 85 | 89 |
| 2513 | 1797 | TYVYT | 2QVP:A | 176 | 180 |
| 2514 | 1798 | TYYYYT | 2CLB:A | 40 | 45 |
| 2515 | 1799 | VAAAV | 2CAK:A | 41 | 45 |
| 2516 |  | VAAAV | 3TX2:A | 211 | 215 |
| 2517 |  | VAAAV | 3MDP:A | 132 | 136 |
| 2518 |  | VAAAV | 1W66:A | 203 | 207 |
| 2519 | 1800 | VADAV | 3CWR:A | 194 | 198 |
| 2520 | 1801 | VAEAV | 1XG5:C | 232 | 236 |
| 2521 | 1802 | VAFAV | 1T0H:A | 60 | 64 |
| 2522 | 1803 | VAGAV | 3TKT:A | 214 | 218 |
| 2523 | 1804 | VAHAV | 3PBT:A | 161 | 165 |
| 2524 | 1805 | VAKAV | 3PIW:A | 56 | 60 |
| 2525 |  | VAKAV | 3CU2:B | 83 | 87 |
| 2526 | 1806 | VALAV | 3Q6X:B | 113 | 117 |
| 2527 |  | VALAV | 2RH0:A | 52 | 56 |
| 2528 |  | VALAV | 2ECU:A | 45 | 49 |
| 2529 | 1807 | VANAV | 3SX2:H | 247 | 251 |
| 2530 |  | VANAV | 3HM2:H | 121 | 125 |
| 2531 | 1808 | VAPAV | 3HUL:B | 125 | 129 |
| 2532 | 1809 | VAQAV | 1JZT:B | 39 | 43 |
| 2533 | 1810 | VASAV | 1G66:A | 68 | 72 |
| 2534 |  | VASAV | 3M0Z:A | 47 | 51 |
| 2535 |  | VASAV | 3M73:A | 47 | 51 |
| 2536 | 1811 | VAVAV | 1ME4:A | 130 | 134 |
| 2537 |  | VAVAV | 2WAS:B | 1879 | 1883 |
| 2538 | 1812 | VCDCV | 3F0H:A | 159 | 163 |
| 2539 | 1813 | VCNCV | 2J4W:D | 448 | 452 |
| 2540 |  | VCNCV | 3F9T:B | 363 | 367 |
| 2541 | 1814 | VDIDV | 3CXG:B | 74 | 78 |
| 2542 | 1815 | VDLDV | 1W7C:A | 536 | 540 |
| 2543 | 1816 | VDMMDV | 3JSY:A | 33 | 38 |
| 2544 | 1817 | VDPDV | 3LED:B | 76 | 80 |
| 2545 | 1818 | VDRDV | 1NUY:A | 1196 | 1200 |
| 2546 |  | VDRDV | 2OS0:A | 117 | 121 |
| 2547 |  | VDRDV | 3LLP:A | 165 | 169 |
| 2548 |  | VDRDV | 3EIX:A | 297 | 301 |
| 2549 | 1819 | VDVDV | 2X27:X | 193 | 197 |
| 2550 | 1820 | VEAEV | 2UV4:A | 293 | 297 |
| 2551 | 1821 | VEDDEV | 3GNJ:A | 93 | 98 |
| 2552 | 1822 | VEEEV | 1UOW:A | 409 | 413 |
| 2553 | 1823 | VEENEEV | 3AJD:A | 216 | 222 |
| 2554 | 1824 | VEGEV | 3JUD:A | 63 | 67 |
| 2555 | 1825 | VEKEV | 1YOZ:A | 120 | 124 |
| 2556 |  | VEKEV | 2HEK:B | 316 | 320 |
| 2557 | 1826 | VELEV | 1OI0:A | 116 | 120 |
| 2558 | 1827 | VENEV | 3NUQ:A | 248 | 252 |
| 2559 |  | VENEV | 1HZT:A | 113 | 117 |
| 2560 | 1828 | VEPEV | 3HTM:D | 246 | 250 |
| 2561 | 1829 | VEQEV | 2OKF:A | 129 | 133 |
| 2562 | 1830 | VESEV | 1TH8:A | 122 | 126 |
| 2563 | 1831 | VETEV | 2QSW:A | 337 | 341 |
| 2564 |  | VETEV | 2D7V:A | 152 | 156 |
| 2565 | 1832 | VEVEV | 3P1V:B | 124 | 128 |
| 2566 |  | VEVEV | 2Q87:A | 101 | 105 |
| 2567 |  | VEVEV | 2PTZ:A | 20 | 24 |
| 2568 | 1833 | VFDFV | 3QQ8:B | 599 | 603 |
| 2569 | 1834 | VFPFV | 1H8P:A | 25 | 29 |
| 2570 | 1835 | VFYFV | 3JRV:A | 11 | 15 |
| 2571 | 1836 | VGAGV | 3LQW:A | 75 | 79 |
| 2572 |  | VGAGV | 1FXK:C | 80 | 84 |
| 2573 | 1837 | VGDGDGV | 3G7R:B | 148 | 154 |
| 2574 | 1838 | VGDGV | 2NL9:A | 216 | 220 |
| 2575 | 1839 | VGEGV | 1Q6O:B | 40 | 44 |
| 2576 | 1840 | VGFFGV | 2J8C:L | 31 | 36 |
| 2577 | 1841 | VGFGV | 1DM1:A | 96 | 100 |
| 2578 | 1842 | VGGGV | 2BDQ:B | 183 | 187 |
| 2579 |  | VGGGV | 2VFR:A | 81 | 85 |
| 2580 | 1843 | VGGKGGV | 2WOJ:C | 23 | 29 |
| 2581 | 1844 | VGHGV | 2HY7:A | 202 | 206 |
| 2582 | 1845 | VGKGV | 1H72:C | 293 | 297 |
| 2583 | 1846 | VGMGV | 1I1K:C | 1105 | 1109 |
| 2584 | 1847 | VGNGV | 1F0L:B | 479 | 483 |
| 2585 | 1848 | VGRGV | 1N4W:A | 108 | 112 |
| 2586 | 1849 | VGSGV | 2WQ4:B | 96 | 100 |
| 2587 | 1850 | VGTGV | 1F00:I | 764 | 768 |
| 2588 |  | VGTGV | 1E7S:A | 245 | 249 |
| 2589 | 1851 | VGVGV | 1G6G:A | 132 | 136 |
| 2590 |  | VGVGV | 2HIN:A | 18 | 22 |
| 2591 | 1852 | VGWGV | 3QO4:A | 152 | 156 |
| 2592 | 1853 | VHLHV | 3O1C:A | 111 | 115 |
| 2593 | 1854 | VHWHV | 2OIK:D | 96 | 100 |
| 2594 | 1855 | VIDIV | 1TXJ:A | 68 | 72 |
| 2595 |  | VIDIV | 3PPL:B | 235 | 239 |
| 2596 | 1856 | VIGIV | 2HD3:K | 78 | 82 |
| 2597 |  | VIGIV | 1P0Z:J | 119 | 123 |
| 2598 | 1857 | VIKIV | 3I38:J | 269 | 273 |
| 2599 | 1858 | VILIV | 2RI0:B | 221 | 225 |
| 2600 | 1859 | VINIV | 2RG9:B | 202 | 206 |
| 2601 | 1860 | VIQNENQIV | 3F14:A | 65 | 73 |
| 2602 | 1861 | VIRIV | 3GWR:A | 84 | 88 |
| 2603 |  | VIRIV | 2WUX:A | 152 | 156 |
| 2604 | 1862 | VISIV | 3L41:A | 792 | 796 |
| 2605 | 1863 | VITIV | 1OKO:A | 25 | 29 |
| 2606 | 1864 | VIVIV | 3ARC:J/3WU2 | 21 | 25 |
| 2607 | 1865 | VKAKV | 2QX3:B | 337 | 341 |
| 2608 | 1866 | VKCKV | 2P09:A | 24 | 28 |
| 2609 | 1867 | VKDKV | 1H72:C | 249 | 253 |
| 2610 | 1868 | VKEKV | 3NO6:A | 101 | 105 |
| 2611 |  | VKEKV | 8A3H:A | 78 | 82 |
| 2612 |  | VKEKV | 2BLA:A | 145 | 149 |
| 2613 | 1869 | VKFFKV | 3BON:A | 355 | 360 |
| 2614 | 1870 | VKFKV | 3BWZ:A | 116 | 120 |
| 2615 | 1871 | VKGKV | 3N0U:A | 201 | 205 |
| 2616 | 1872 | VKIKV | 1MN8:D | 64 | 68 |
| 2617 | 1873 | VKKKV | 1ORJ:D | 4120 | 4124 |
| 2618 |  | VKKKV | 3G9K:S | 488 | 492 |
| 2619 | 1874 | VKLKV | 3CIJ:B | 33 | 37 |
| 2620 | 1875 | VKTKV | 1JUQ:D | 110 | 114 |
| 2621 | 1876 | VKVKV | 3LFJ:B | 56 | 60 |
| 2622 |  | VKVKV | 2BU3:B | 137 | 141 |
| 2623 |  | VKVKV | 1LUZ:B | 63 | 67 |
| 2624 | 1877 | VKWKV | 3MNM:A | 561 | 565 |
| 2625 | 1878 | VLALV | 1E29:A | 69 | 73 |
| 2626 |  | VLALV | 3I7M:A | 41 | 45 |
| 2627 |  | VLALV | 2ZKM:X | 130 | 134 |
| 2628 |  | VLALV | 2ZYZ:C | 67 | 71 |
| 2629 | 1879 | VLDLV | 3O2R:B | 46 | 50 |
| 2630 | 1880 | VLELV | 3EAT:X | 88 | 92 |
| 2631 |  | VLELV | 3AG3:B | 210 | 214 |
| 2632 | 1881 | VLGLV | 1D3B:L | 66 | 70 |
| 2633 |  | VLGLV | 2RCI:A | 121 | 125 |
| 2634 |  | VLGLV | 3KHF:B | 1011 | 1015 |
| 2635 | 1882 | VLHLV | 1XU1:D | 108 | 112 |
| 2636 | 1883 | VLIEILV | 3BQP:B | 57 | 63 |
| 2637 | 1884 | VLKLV | 3C3Y:A | 65 | 69 |
| 2638 | 1885 | VLLLV | 2QSQ:B | 17 | 21 |
| 2639 |  | VLLLV | 2XTM:B | 108 | 112 |
| 2640 | 1886 | VLNLV | 2Q3E:A | 281 | 285 |
| 2641 | 1887 | VLPLV | 2QVP:A | 103 | 107 |
| 2642 |  | VLPLV | 3CG7:A | 239 | 243 |
| 2643 | 1888 | VLQLV | 3D1B:C | 476 | 480 |
| 2644 |  | VLQLV | 1GP0:A | 1587 | 1591 |
| 2645 | 1889 | VLRLV | 3QUV:A | 152 | 156 |
| 2646 | 1890 | VLTLV | 2EA7:A | 93 | 97 |
| 2647 | 1891 | VMKMV | 3DK9:A | 418 | 422 |
| 2648 | 1892 | VNKKNV | 3BPU:A | 689 | 694 |
| 2649 | 1893 | VNNNV | 3OMY:A | 8 | 12 |
| 2650 | 1894 | VNPPPNV | 3CZ6:A | 777 | 783 |
| 2651 | 1895 | VNSSNV | 3EOJ:A | 205 | 210 |
| 2652 | 1896 | VPDPV | 2D1S:A | 474 | 478 |
| 2653 | 1897 | VPEPV | 1FX4:A | 1082 | 1086 |
| 2654 |  | VPEPV | 1P5Z:B | 51 | 55 |
| 2655 | 1898 | VPIPV | 3P8B:D | 131 | 135 |
| 2656 | 1899 | VPKPV | 2QRD:C | 482 | 486 |
| 2657 | 1900 | VPLPV | 1W4S:A | 1088 | 1092 |
| 2658 | 1901 | VPVPV | 1JR7:A | 275 | 279 |
| 2659 | 1902 | VQDQV | 1UWC:A | 107 | 111 |
| 2660 | 1903 | VQLLQV | 3U9Q:A | 450 | 455 |
| 2661 | 1904 | VQMQV | 2CKW:A | 224 | 228 |
| 2662 | 1905 | VQPQV | 1TCA:A | 190 | 194 |
| 2663 | 1906 | VQQQV | 3CP7:A | 105 | 109 |
| 2664 | 1907 | VQSQV | 2O71:A | 156 | 160 |
| 2665 | 1908 | VQTQV | 2XGT:A | 273 | 277 |
| 2666 |  | VQTQV | 3HD5:C | 154 | 158 |
| 2667 | 1909 | VRDRV | 1YIS:A | 429 | 433 |
| 2668 | 1910 | VRFRV | 2PN0:C | 61 | 65 |
| 2669 | 1911 | VRWRV | 2B5I:B | 88 | 92 |
| 2670 | 1912 | VSDSV | 3C8C:A | 66 | 70 |
| 2671 | 1913 | VSISV | 3AON:A | 88 | 92 |
| 2672 |  | VSISV | 1TH8:A | 64 | 68 |
| 2673 | 1914 | VSNSV | 2ZX2:B | 172 | 176 |
| 2674 | 1915 | VSSSV | 3CJW:A | 373 | 377 |
| 2675 | 1916 | VSTSV | 3R8J:A | 49 | 53 |
| 2676 | 1917 | VSVSV | 3A72:A | 367 | 371 |
| 2677 |  | VSVSV | 3OV5:A | 117 | 121 |
| 2678 |  | VSVSV | 2VBK:A | 327 | 331 |
| 2679 | 1918 | VSYSV | 3JWI:A | 61 | 65 |
| 2680 | 1919 | VTATV | 3QR7:B | 135 | 139 |
| 2681 |  | VTATV | 2X6W:A | 431 | 435 |
| 2682 | 1920 | VTDTV | 1O6V:B | 66 | 70 |
| 2683 |  | VTDTV | 3LRT:A | 260 | 264 |
| 2684 | 1921 | VTFTV | 2QMQ:A | 50 | 54 |
| 2685 |  | VTFTV | 3QOO:A | 82 | 86 |
| 2686 |  | VTFTV | 3EVF:A | 229 | 233 |
| 2687 | 1922 | VTGTV | 1W2W:J | 340 | 344 |
| 2688 | 1923 | VTHTV | 3LQK:A | 40 | 44 |
| 2689 |  | VTHTV | 3BB0:A | 36 | 40 |
| 2690 | 1924 | VTITV | 3RL5:A | 11 | 15 |
| 2691 |  | VTITV | 3RY4:A | 168 | 172 |
| 2692 | 1925 | VTKTV | 3H8T:A | 37 | 41 |
| 2693 | 1926 | VTLTV | 2BSY:A | 152 | 156 |
| 2694 | 1927 | VTPTV | 1P1J:B | 47 | 51 |
| 2695 | 1928 | VTRTV | 2AEB:B | 289 | 293 |
| 2696 |  | VTRTV | 1O50:A | 35 | 39 |
| 2697 | 1929 | VTVTV | 1P5U:C | 142 | 146 |
| 2698 |  | VTVTV | 2OSX:A | 482 | 486 |
| 2699 |  | VTVTV | 1KZQ:A | 124 | 128 |
| 2700 |  | VTVTV | 2QZT:B | 61 | 65 |
| 2701 |  | VTVTV | 2BTI:A | 18 | 22 |
| 2702 | 1930 | VTYTV | 1V05:A | 2694 | 2698 |
| 2703 | 1931 | VVAVV | 3CJS:C | 4 | 8 |
| 2704 | 1932 | VVDDVV | 3EYE:A | 35 | 40 |
| 2705 | 1933 | VVEVV | 3LUU:A | 65 | 69 |
| 2706 | 1934 | VVHVV | 3LUM:D | 70 | 74 |
| 2707 |  | VVHVV | 3H9W:A | 105 | 109 |
| 2708 | 1935 | VVIVV | 1R29:A | 34 | 38 |
| 2709 | 1936 | VVKVV | 1ST9:A | 151 | 155 |
| 2710 |  | VVKVV | 2PFI:B | 567 | 571 |
| 2711 | 1937 | VVLVV | 1MJ5:A | 102 | 106 |
| 2712 |  | VVLVV | 3CTZ:A | 539 | 543 |
| 2713 |  | VVLVV | 2EV1:B | 127 | 131 |
| 2714 | 1938 | VVMVV | 2Z1E:A | 275 | 279 |
| 2715 | 1939 | VYAYV | 2BW4:A | 300 | 304 |
| 2716 | 1940 | VYDYV | 2XTS:D | 113 | 117 |
| 2717 | 1941 | VYEYV | 2P6W:A | 155 | 159 |
| 2718 | 1942 | VYLYV | 2Y0O:A | 91 | 95 |
| 2719 | 1943 | WALAW | 2H00:C | 246 | 250 |
| 2720 | 1944 | WAMAW | 3QXV:E | 34 | 38 |
| 2721 | 1945 | WGEGW | 1HX0:A | 280 | 284 |
| 2722 | 1946 | WGNGW | 1QNR:A | 253 | 257 |
| 2723 | 1947 | WKEKW | 3BB0:A | 350 | 354 |
| 2724 |  | WKEKW | 2GB4:B | 24 | 28 |
| 2725 | 1948 | WNNNW | 3P6B:B | 134 | 138 |
| 2726 | 1949 | WNVVNW | 1IX9:A | 189 | 194 |
| 2727 | 1950 | WQAQW | 1S9U:A | 42 | 46 |
| 2728 | 1951 | WQTQW | 3H6P:C | 54 | 58 |
| 2729 | 1952 | WSASW | 3PP2:A | 520 | 524 |
| 2730 | 1953 | WSEESW | 2A6S:D | 5 | 10 |
| 2731 | 1954 | WVKVW | 1NPI:A | 50 | 54 |
| 2732 | 1955 | WWQWW | 2J8C:L | 262 | 266 |
| 2733 | 1956 | YAKAY | 1H3L:A | 69 | 73 |
| 2734 |  | YAKAY | 2R8O:A | 314 | 318 |
| 2735 | 1957 | YANAY | 1T61:A | 185 | 189 |
| 2736 | 1958 | YAPAY | 3KV1:A | 116 | 120 |
| 2737 | 1959 | YASAY | 1W5Q:A | 206 | 210 |
| 2738 | 1960 | YATAY | 2ABS:A | 165 | 169 |
| 2739 | 1961 | YDEDY | 3DRA:A | 28 | 32 |
| 2740 | 1962 | YDRRDY | 1EJF:B | 9 | 14 |
| 2741 | 1963 | YDVDY | 2F6U:B | 2073 | 2077 |
| 2742 | 1964 | YEEEY | 1XIY:A | 67 | 71 |
| 2743 | 1965 | YEIEY | 3OK8:A | 122 | 126 |
| 2744 |  | YEIEY | 3P02:A | 306 | 310 |
| 2745 | 1966 | YETEY | 3KIZ:B | 196 | 200 |
| 2746 | 1967 | YEYEY | 3OJ0:A | 61 | 65 |
| 2747 | 1968 | YGEGY | 3SG0:A | 168 | 172 |
| 2748 | 1969 | YGGGY | 1QQF:A | 1262 | 1266 |
| 2749 |  | YGGGY | 3L77:A | 146 | 150 |
| 2750 | 1970 | YGIIIGY | 2QF4:B | 190 | 196 |
| 2751 | 1971 | YGKGY | 3O8Q:A | 219 | 223 |
| 2752 | 1972 | YGLGY | 3RF7:A | 262 | 266 |
| 2753 |  | YGLGY | 3RQ7:A | 421 | 425 |
| 2754 | 1973 | YGRGY | 1QOP:A | 169 | 173 |
| 2755 | 1974 | YGSGY | 3NNG:B | 262 | 266 |
| 2756 | 1975 | YGTGY | 2V3I:A | 167 | 171 |
| 2757 | 1976 | YGYGY | 1GK9:A | 29 | 33 |
| 2758 | 1977 | YIKIY | 3KVP:B | 39 | 43 |
| 2759 | 1978 | YKDKY | 3SEB:A | 77 | 81 |
| 2760 | 1979 | YKKKY | 2V8Q:B | 259 | 263 |
| 2761 | 1980 | YKTKY | 1VJN:A | 162 | 166 |
| 2762 |  | YKTKY | 2FTX:B | 202 | 206 |
| 2763 | 1981 | YLALY | 3BF5:A | 234 | 238 |
| 2764 | 1982 | YLCLY | 2BF6:A | 370 | 374 |
| 2765 | 1983 | YLDLY | 1US0:A | 103 | 107 |
| 2766 | 1984 | YLELY | 2I5I:B | 166 | 170 |
| 2767 | 1985 | YLILY | 3L4R:A | 100 | 104 |
| 2768 |  | YLILY | 2IBP:B | 67 | 71 |
| 2769 | 1986 | YLMLY | 3JZ0:A | 67 | 71 |
| 2770 | 1987 | YLPLY | 2VSH:B | 68 | 72 |
| 2771 | 1988 | YLSLY | 3IVV:A | 83 | 87 |
| 2772 |  | YLSLY | 2XHF:B | 89 | 93 |
| 2773 |  | YLSLY | 3VC8:A | 32 | 36 |
| 2774 | 1989 | YLTLY | 1O6V:B | 343 | 347 |
| 2775 | 1990 | YLVLY | 1SJW:A | 80 | 84 |
| 2776 | 1991 | YNTNY | 3BB0:A | 18 | 22 |
| 2777 | 1992 | YNYVYNY | 2QX3:B | 191 | 197 |
| 2778 | 1993 | YPPPY | 1DQT:A | 98 | 102 |
| 2779 | 1994 | YQFVFQY | 3KEV:A | 101 | 107 |
| 2780 | 1995 | YQQQY | 2ZHJ:A | 128 | 132 |
| 2781 | 1996 | YRDRY | 1S5D:A | 145 | 149 |
| 2782 |  | YRDRY | 1EJ2:A | 126 | 130 |
| 2783 | 1997 | YRGRY | 2YH6:A | 133 | 137 |
| 2784 | 1998 | YSNSY | 2B8M:A | 43 | 47 |
| 2785 | 1999 | YSVEVSY | 2ZFI:A | 140 | 146 |
| 2786 | 2000 | YTATY | 3Q64:A | 73 | 77 |
| 2787 | 2001 | YTGTY | 1GUQ:A | 69 | 73 |
| 2788 | 2002 | YTLTY | 2Y7L:A | 270 | 274 |
| 2789 | 2003 | YTQTY | 2XE5:A | 235 | 239 |
| 2790 | 2004 | YVDDVY | 2ZEX:A | 137 | 142 |
| 2791 | 2005 | YVEEVY | 1UFO:D | 77 | 82 |
| 2792 | 2006 | YVIVY | 3Q72:A | 165 | 169 |
| 2793 | 2007 | YVNNNVY | 3OMY:A | 7 | 13 |
| 2794 | 2008 | YVQVY | 2XFR:A | 9 | 13 |
| 2795 |  | YVQVY | 2HEK:B | 217 | 221 |
| 2796 | 2009 | YVRVY | 2HYK:A | 238 | 242 |
| 2797 | 2010 | YYGYY | 1KWF:A | 368 | 372 |
| 2798 |  | YYGYY | 3I26:B | 226 | 230 |
| 2799 | 2011 | YYLYY | 3CU9:A | 95 | 99 |
| 2800 |  | YYLYY | 2HUH:A | 120 | 124 |
| 2801 | 2012 | YYPYY | 2ZXE:B | 244 | 248 |
| 2802 | 2013 | YYQYY | 2QSA:A | 115 | 119 |
| 2803 | 2014 | YYSSYY | 1G1S:A | 44 | 49 |
