# Supplementary material for: Analyses of the Sequence and Structural Properties Corresponding to Pentapeptide and Large Palindromes in Proteins
Source: PLoS One. 2015 Oct 14;10(10):e0139568. doi: 10.1371/journal.pone.0139568 (PMC4605511; doi:10.1371/journal.pone.0139568)
Supplement: S4 Appendix — (DOC) [file pone.0139568.s004.doc]

| **S4 Appendix. Distribution of the number of palindrome peptides in representative proteins.** | | |
| --- | --- | --- |
| S.No. | **Protein name classification** | **No. of PALINs** |
| 1 | activation domain | 1 |
| 2 | actin-binding protein | 2 |
| 3 | agglutinin | 2 |
| 4 | allergen | 4 |
| 5 | antibiotic resistance | 4 |
| 6 | anti-coagulant | 1 |
| 7 | antifungal protein | 2 |
| 8 | anti-hiv protein | 2 |
| 9 | antimicrobial protein | 2 |
| 10 | apoprotein | 1 |
| 11 | apoptosis | 19 |
| 12 | basement membrane | 3 |
| 13 | biosynthetic protein | 14 |
| 14 | blood clotting | 2 |
| 15 | carbohydrate-binding module | 1 |
| 16 | cell adhesion | 53 |
| 17 | cell cycle/ hydrolase | 24 |
| 18 | cell differentiation | 1 |
| 19 | cell invasion | 6 |
| 20 | cellulosome subunit | 1 |
| 21 | chaperone | 35 |
| 22 | chaperone regulator | 1 |
| 23 | collagen | 1 |
| 24 | complex (isomerase/peptide) | 4 |
| 25 | contractile | 2 |
| 26 | cysteine protease | 1 |
| 27 | cytokine | 25 |
| 28 | de novo protein | 16 |
| 29 | disulfide oxidoreductase | 1 |
| 30 | dna recombination | 1 |
| 31 | dna binding protein | 28 |
| 32 | DNA repair | 1 |
| 33 | electron transport | 47 |
| 34 | endocytosis/exocytosis | 4 |
| 35 | endonuclease | 3 |
| 36 | epimerase/reductase | 1 |
| 37 | eye lens protein | 1 |
| 38 | flavoenzyme | 4 |
| 39 | fluorescent protein | 5 |
| 40 | fmn-binding protein | 1 |
| 41 | gene regulation | 11 |
| 42 | glycoprotein | 4 |
| 43 | growth factor | 1 |
| 44 | griseoluteate binding protein | 1 |
| 45 | heme binding protein | 4 |
| 46 | hormone/growth factor | 10 |
| 47 | hydrolase | 526 |
| 48 | immune system | 65 |
| 49 | inhibitor | 1 |
| 50 | ion transport | 2 |
| 51 | isomerase | 63 |
| 52 | lectin | 5 |
| 53 | leucine zipper | 2 |
| 54 | ligand binding protein | 7 |
| 55 | ligase | 49 |
| 56 | lipid binding protein/lipoprotein | 21 |
| 57 | luminescent protein | 3 |
| 58 | lyase | 145 |
| 59 | maltose-binding protein | 3 |
| 60 | membrane protein | 22 |
| 61 | metal binding protein | 21 |
| 62 | metal transport | 6 |
| 63 | metallothionein | 1 |
| 64 | methanogenesis | 6 |
| 65 | motor protein/signaling protein | 1 |
| 66 | methyltransferase | 3 |
| 67 | nuclear protein | 8 |
| 68 | nucleotide binding protein | 5 |
| 69 | nucleotidyltransferase | 3 |
| 70 | oxidoreductase | 292 |
| 71 | oxygen binding | 2 |
| 72 | oxygen storage/transport | 12 |
| 73 | oxygenase | 3 |
| 74 | peptide binding protein | 3 |
| 75 | phosphorylcholine-binding protein | 2 |
| 76 | phosphotransferase | 2 |
| 77 | pheromone binding protein | 1 |
| 78 | photosynthesis | 10 |
| 79 | plant protein | 9 |
| 80 | polyketide synthase | 1 |
| 81 | protein binding | 47 |
| 82 | protein transport | 28 |
| 83 | proto-oncogene | 1 |
| 84 | receptor | 4 |
| 85 | recombination | 4 |
| 86 | regulator | 2 |
| 87 | replication | 1 |
| 88 | ribosomal protein | 6 |
| 89 | RNA Binding Protein | 18 |
| 90 | serine protease | 5 |
| 91 | signaling protein | 55 |
| 92 | splicing | 3 |
| 93 | stress-response | 3 |
| 94 | structural genomics, unknown function | 206 |
| 95 | structural protein | 29 |
| 96 | sugar binding protein | 23 |
| 97 | synthetase | 3 |
| 98 | thiamin biosynthesis | 1 |
| 99 | toxin | 50 |
| 100 | transcription | 124 |
| 101 | transferase | 330 |
| 102 | translation | 10 |
| 103 | transport protein | 57 |
| 104 | unknown function | 59 |
| 105 | vib binding protein | 3 |
| 106 | viral protein | 67 |
| 107 | zinc-finger protein | 1 |
|  | Total number of PALINs | **2803** |
